# Investigation on the organic–inorganic hybrid crystal [NH_2_(CH_3_)_2_]_2_CuBr_4_: structure, phase transition, thermal property, structural geometry, and dynamics

**DOI:** 10.1038/s41598-023-48015-6

**Published:** 2023-11-29

**Authors:** Changyub Na, Ae Ran Lim

**Affiliations:** 1 Graduate School of Carbon Convergence Engineering, Jeonju University, Jeonju, 55069 South Korea; 2 Department of Science Education, Jeonju University, Jeonju, 55069 South Korea

**Keywords:** Organic chemistry, Materials science

## Abstract

Understanding the physical properties of the organic–inorganic hybrid [NH_2_(CH_3_)_2_]_2_CuBr_4_ is essential to expand its applications. The single [NH_2_(CH_3_)_2_]_2_CuBr_4_ crystals were grown and their comprehensive properties were investigated. The crystals had a monoclinic structure with the space group *P2*_*1*_*/n* and lattice constants of* a* = 8.8651 (5) Å, *b* = 11.9938 (6) Å, *c* = 13.3559 (7) Å, and *β* = 91.322°. The transition temperature from phase I to phase II was determined to be 388 K. Variations in the ^1^H nuclear magnetic resonance chemical shifts of NH_2_ and ^14^N NMR chemical shifts according to the temperature changes in the cation were attributed to vibrations of NH_2_ groups at their localization sites. The ^1^H and ^13^C spin–lattice relaxation times (T_1ρ_) in phase II changed significantly with temperature, indicating that these values are governed by molecular motion. The T_1ρ_ values were much longer in phase I than in phase II, which means energy transfer was difficult. Finally, the activation energies for phases I and II were considered. According to the basic mechanism of [NH_2_(CH_3_)_2_]_2_CuBr_4_ crystals, organic–inorganic materials may have potential applications in various fields.

## Introduction

Significant attention has been paid to organic–inorganic hybrid compounds owing to their diverse applications as catalysts, sensors, functional smart coatings, fuel and solar cells, light-emitting diodes, light-emitting transistors, and perovskite photovoltaic cells^1–12^. Perovskite type organic–inorganic metal halides are lead-based CH_3_NH_3_Pb*X*_3_ (*X* = Cl, Br, I) compounds with a 3D structure. However, CH_3_NH_3_Pb*X*_3_-based photovoltaics demonstrate high instability under typical environmental conditions, notably moisture, as well as high toxicity owing to the bioaccumulation of Pb^13–18^. Thus, researchers have suggested the substitution of Pb with other low-toxicity or eco-friendly metals such as Cu and Zn to develop Pb-free photovoltaic devices^19^. The urgent need to develop eco-friendly hybrid perovskite solar cells has recently been highlighted. For example, two-dimensional [NH_3_(CH_2_)_*n*_NH_3_]*BX*_4_ (*n* = 2, 3, …, *B* = Mn, Co, Cu, Zn, Cd; *X* = Cl, Br, I) and [(C_*n*_H_2*n*+1_NH_3_)]_2_*BX*_4_ organic–inorganic hybrids have recently been proposed as an alternative to these materials^20–27^. In addition, it is needed to study for [NH_2_(CH_3_)_*n*_]_2_*BX*_4_^28–33^, which has a different hydrogen-bond structure from [NH_3_(CH_2_)_*n*_NH_3_]*BX*_4_, which has three H atoms bonded to one N. The overall structure of these materials consists of a 1D inorganic network. The isolated building blocks in these 1D perovskites provide a large degree of freedom for the dynamic motion of organic ammonium cations, which can trigger a disorder-to-order transition. Such materials are expected to act as proton conductors owing to the availability of hydrogen bonds.

Dimethylammonium tetrabromocuprate (II) [NH_2_(CH_3_)_2_]_2_CuBr_4_ is a member of the [NH_2_(CH_3_)_*n*_]_2_*BX*_4_ family. In this group of compounds, the individual *BX*_4_ tetrahedral anions are isolated and surrounded by [NH_2_(CH_3_)_2_]^+^ cations. However, the properties and characteristics of [NH_2_(CH_3_)_2_]_2_CuBr_4_ have not yet been reported.

In this study, the single crystals of [NH_2_(CH_3_)_2_]_2_CuBr_4_ were grown using an aqueous solution method, and their structure, the phase-transition temperature (T_C_), and the thermal property was considered. The coordination geometries around ^1^H, ^13^C, and ^14^N atoms were investigated by obtaining the chemical shifts of the ^1^H magic-angle spinning (MAS) nuclear magnetic resonance (NMR), ^13^C MAS NMR, and static ^14^N NMR as functions of temperature. In addition, the ^1^H and ^13^C NMR spin–lattice relaxation times (T_1ρ_), which represent the energy transfer surrounding the ^1^H and ^13^C atoms of the cation, respectively, were discussed, and their activation energies (E_a_) were determined. The single-crystal structure and physical properties observed in this study are expected to important information on the basic mechanism for various applications.

## Methods

### Crystal growth

Single crystals of [NH_2_(CH_3_)_2_]_2_CuBr_4_ were prepared by dissolving dimethylammonium bromide (Aldrich, 98%) and CuBr_2_ (Aldrich, 98%) at a ratio of 2:1 in tertiary distilled water. To make a saturated solution, the mixed material was heated, stirred, and filtered through filter paper. The prepared saturated solution was placed in a beaker, covered with filter paper, to let natural evaporation in an apparatus with a constant temperature of 300 K. Several dark brown colored single crystals with sizes of 6 × 3 × 1 mm were obtained after a few days.

### Characterization

The structure and lattice parameters of the [NH_2_(CH_3_)_2_]_2_CuBr_4_ crystals were determined at 300 K using the single-crystal X-ray diffraction (SCXRD) system of the Seoul Western Center at the Korea Basic Science Institute (KBSI). SCXRD measurements were performed using a diffractometer with a graphite-monochromated Mo-Kα target with a wavelength of 0.71073 Å under a cold nitrogen flow (− 50 °C) (Bruker D8 Venture PHOTON III M14). The data was collected using SMART APEX3 (Bruker 2016) and SAINT (Bruker, 2016). The crystal structure was solved using direct methods and refined using the full-matrix least-squares method^34^. All hydrogen atoms are presented in their geometric positions. Additionally, powder X-ray diffraction (PXRD) patterns were measured using an XRD system at 300 K with the same target used for SCXRD.

Differential scanning calorimetry (DSC) results were performed in the temperature range of 200–423 K using a DSC instrument (TA Instruments, DSC 25) under a nitrogen atmosphere. The thermogram was measured using the sample of 6.1 mg at a heating rate of 10 °C/min.

Thermogravimetric analysis (TGA) results were performed at a heating rate of 10 °C/min under dry nitrogen gas. The thermogram was collected using a 15.92 mg sample while heating from room temperature to 900 K.

The MAS NMR chemical shifts and spin–lattice relaxation time T_1ρ_ of the [NH_2_(CH_3_)_2_]_2_CuBr_4_ crystals were measured using a solid-state NMR spectrometer (AVANCE II + , Bruker) at the Seoul Western Center of the KBSI. The Larmor frequency for the ^1^H NMR experiment was 400.13 MHz, while that for the ^13^C NMR experiment was 100.61 MHz. The samples were placed in a cylindrical zirconia rotor and then subjected to MAS NMR measurements at a spinning rate of 10 kHz to reduce the spinning sidebands. Chemical shifts were measured using adamantane and tetramethylsilane as the standards for ^1^H and ^13^C, respectively. The 1D NMR spectra of ^1^H and ^13^C were obtained with a delay time of 0.2‒2 s. To obtain T_1ρ_ values, the one-pulse method was used, and the delay times were within 0.4‒4 s, and the 90° pulse for ^1^H and ^13^C was used to 3.45–7.5 μs and 5–5.5 μs, respectively. The ^13^C T_1ρ_ values were obtained by varying the duration of the ^13^C spin-locking pulse applied after cross-polarization (CP) preparation. Static ^14^N NMR chemical shifts were recorded using the one-pulse method at a Larmor frequency of 28.90 MHz with NH_4_NO_3_ as the standard sample. NMR experiments above 430 K were not possible because of the limitations of the NMR instrument. The temperature was maintained nearly constant within the error range of ± 0.5 °C, even when the N_2_ gas flow rate and heater current were adjusted.

## Results and discussions

### Crystal structure

**Figure 1:**
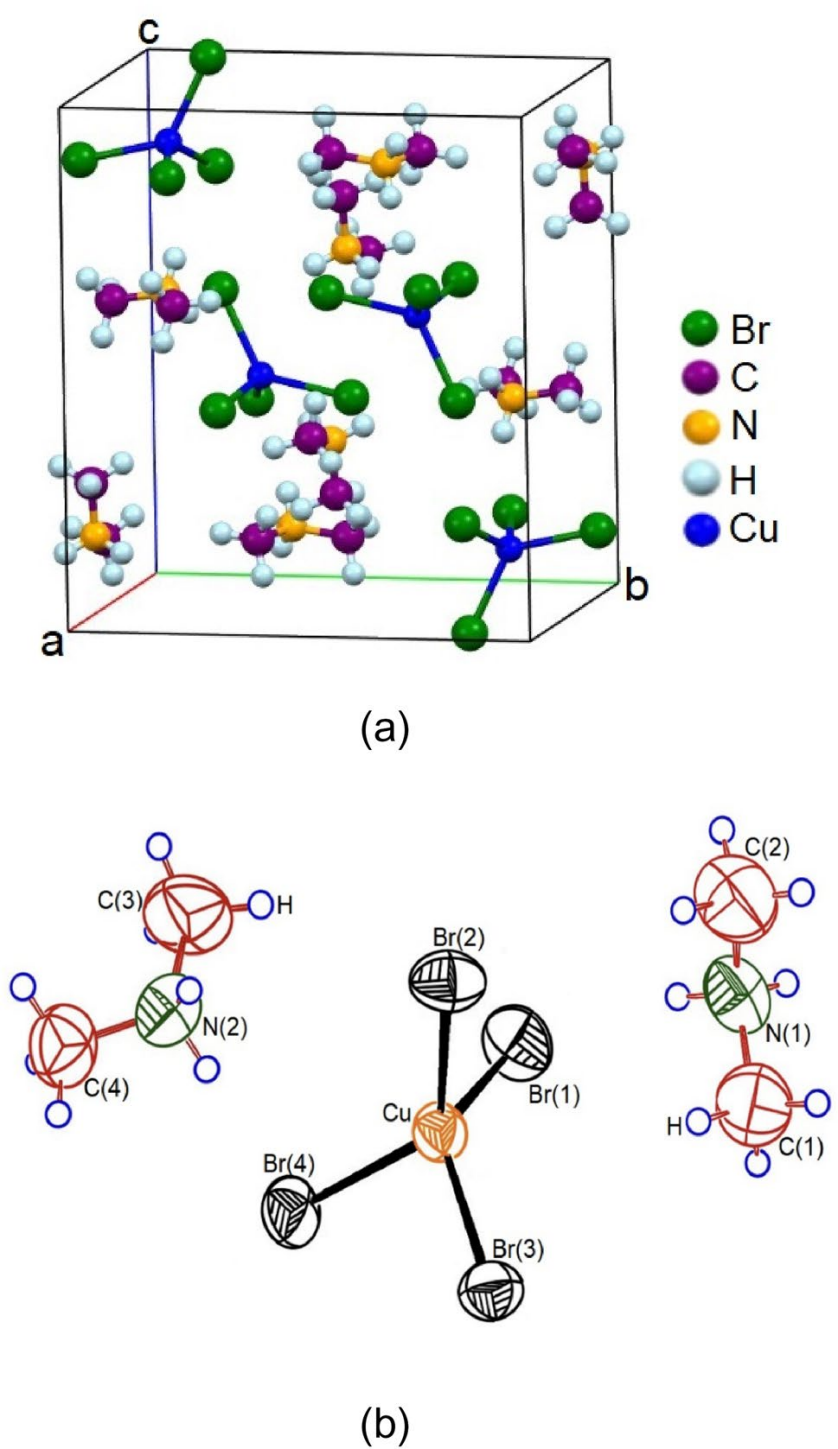
(**a**) Structure of the [NH_2_(CH_3_)_2_]_2_CuBr_4_ crystal at 300 K (CCDC No. 2290529). (**b**) Thermal ellipsoid plot for [NH_2_(CH_3_)_2_]_2_CuBr_4_ crystal structure at 300 K.

The SCXRD results of the [NH_2_(CH_3_)_2_]_2_CuBr_4_ crystals were obtained at 300 K. The crystals had a monoclinic structure with the *P2*_*1*_*/n* space group and lattice constants of *a* = 8.8651 (5) Å, *b* = 11.9938 (6) Å, *c* = 13.3559 (7) Å, *β* = 91.322 (2)°, and *Z* = 4. A perspective view of the atomic arrangement in the unit cell of a [NH_2_(CH_3_)_2_]_2_CuBr_4_ crystal is shown in Fig. 1a,b. Here, the [CuBr_4_]^2−^ tetrahedra and [NH_2_(CH_3_)_2_] cations are linked by hydrogen bonds. Specifically, the crystal structure consists of discrete, slightly deformed CuBr_4_ tetrahedra linked to the organic cations through N‒H∙∙∙Br hydrogen bonds^28‒30,33^. The SCXRD data for the [NH_2_(CH_3_)_2_]_2_CuBr_4_ crystals are shown in Table 1, and the corresponding bond lengths and angles are presented in Table 2. The CIF file result of SCXRD for crystal structure at 300 K is shown in the Supplementary information S1.

**Table 1:** Structure of single crystal for [NH_2_(CH_3_)_2_]_2_CuBr_4_ at 300 K (CCDC No. 2290529).

Chemical formula	C_4_H_16_N_2_CuBr_4_
Weight	475.37
Crystal structure	Monoclinic
Space group	*P2*_*1*_*/n*
T (K)	300
*a* (Å)	8.8651 (5)
*b* (Å)	11.9938 (6)
*c* (Å)	13.3559 (7)
α (°)	90
β (°)	91.322 (2)
γ (°)	90
Z	4
V (Å^3^)	1419.70
Wavelength (Å)	0.71073
θ range for data collection (°)	2.283–28.331
Reflections collected	29,993
Independent reflections	3535 (*R*_int_ = 0.0951)
Goodness-of-fit on *F*^2^	0.990
Final *R* indices [I > 2sigma(I)]	*R*_1_ = 0.0408, *wR*_2_ = 0.0690
*R* indices (all data)	*R*_1_ = 0.1156, *wR*_2_ = 0.0861

**Table 2:** Bond-lengths (Å) and bond-angles (°) for [NH_2_(CH_3_)_2_]_2_CuBr_4_ at 300 K.

Cu–Br(1)	2.3511 (8)
Cu–Br(2)	2.3772 (8)
Cu–Br(3)	2.3917 (9)
Cu–Br(4)	2.3918 (8)
N(1)–C(1)	1.447 (7)
N(1)–C(2)	1.456 (7)
N(2)–C(3)	1.437 (7)
N(2)–C(4)	1.461 (7)
N–H	0.89
C–H	0.96
Br(1)–Cu–Br(2)	100.02 (3)
Br(1)–Cu–Br(3)	131.86 (4)
Br(2)–Cu–Br(3)	101.03 (3)
Br(1)–Cu–Br(4)	100.43 (3)
Br(2)–Cu–Br(4)	128.13 (4)
Br(3)–Cu–Br(4)	99.62 (3)

**Figure 2:**
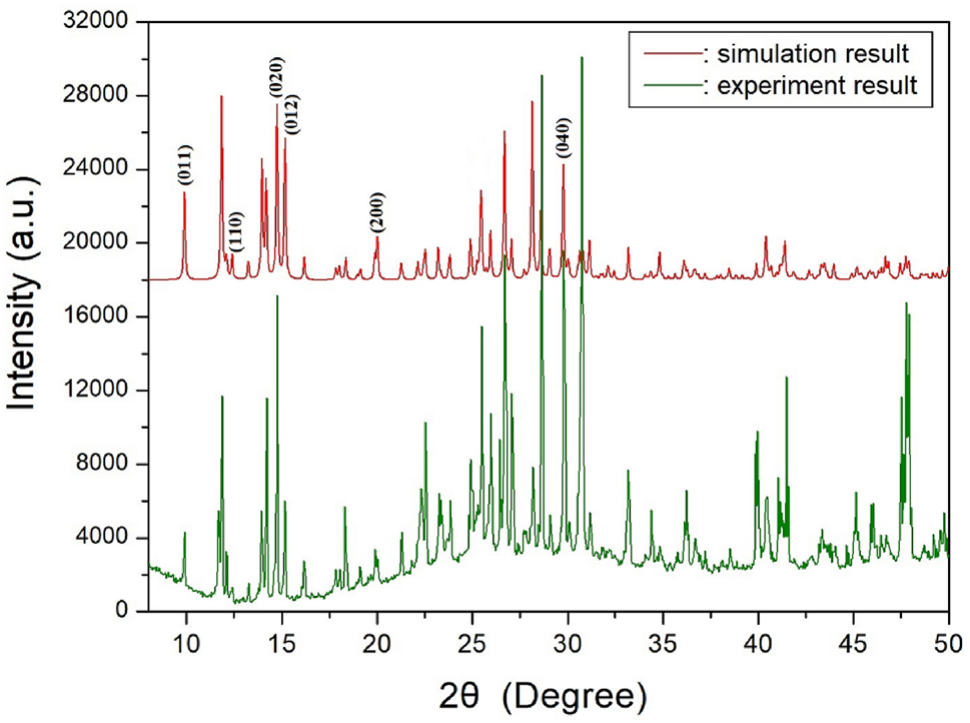
Powder XRD and simulated XRD patterns of [NH_2_(CH_3_)_2_]_2_CuBr_4_ at 300 K.

In addition, PXRD experiments were performed at 300 K. The PXRD patterns obtained in the 2θ measurement range of 8°–50° are shown in red color in Fig. 2. The PXRD pattern simulated by SCXRD structural parameters was consistent with that determined from the PXRD experiment at 300 K. The peaks observed in this diffractogram were indexed using the Mercury program as shown in Fig. 2.

### Phase transition temperature

**Figure 3:**
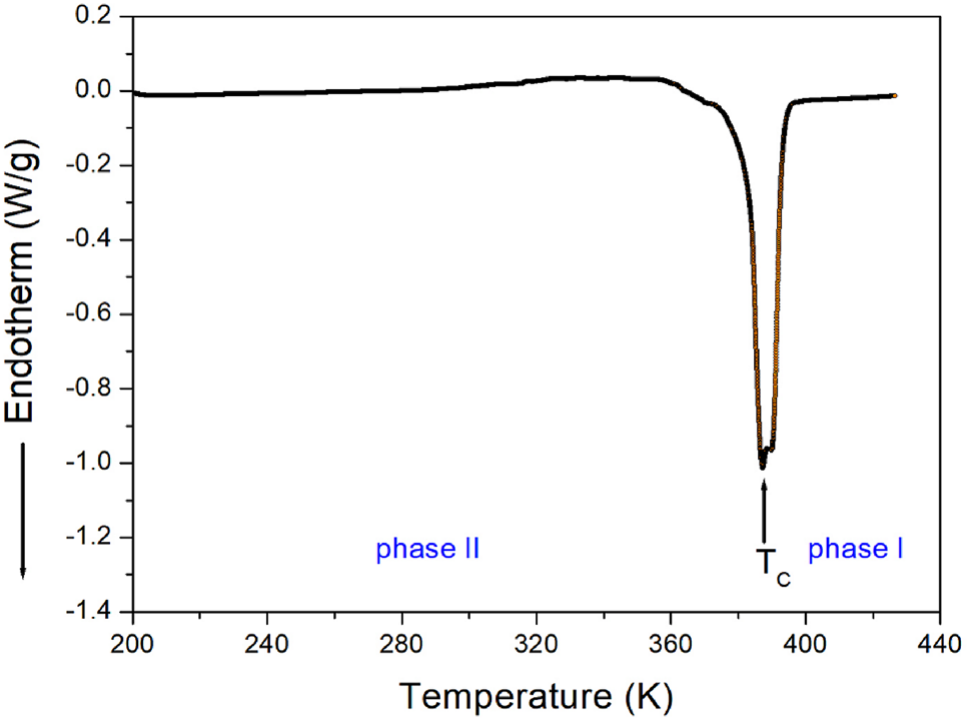
Differential scanning calorimetry (DSC) curve of [NH_2_(CH_3_)_2_]_2_CuBr_4_ measured at a heating rate of 10 °C/min.

The DSC thermogram of the crystals was measured in the temperature range of 200–423 K at a heating rate of 10 °C/min. Figure 3 shows a strong endothermic peak at 388 K with an enthalpy of 22.26 kJ/mol. The two phases were denoted as phase I, which refers to the region above 388 K, and phase II, which refers to the region below 388 K.

### Thermal property

**Figure 4:**
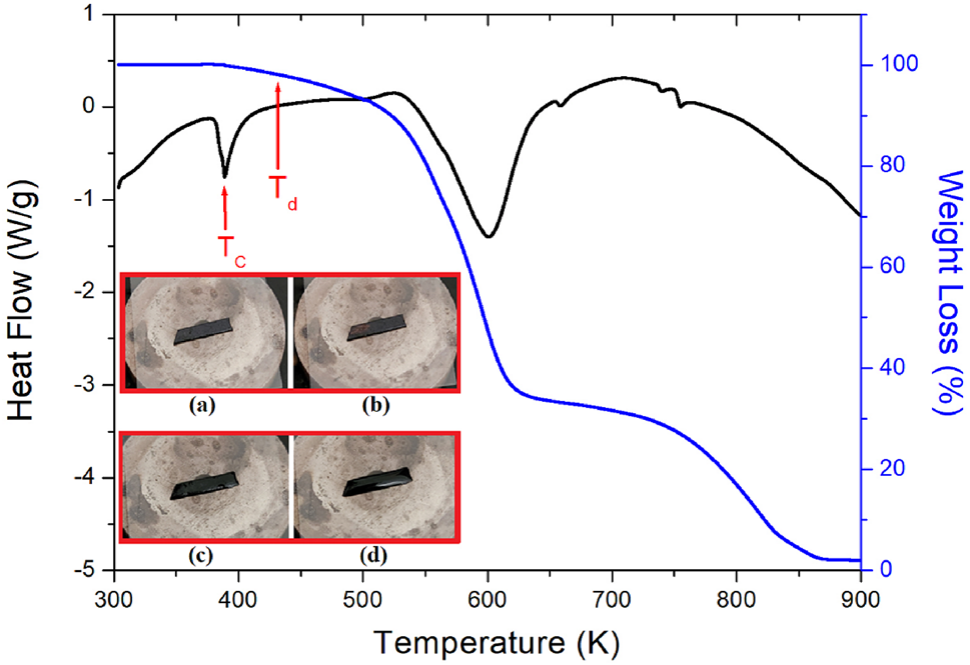
Thermogravimetric analysis (TGA) and differential thermal analysis (DTA) results of [NH_2_(CH_3_)_2_]_2_CuBr_4_ (Inset: Morphology in crystal by polarizing microscopy at (**a**) 300 K, (**b**) 373 K, (**c**) 430 K, and (**d**) 490 K for [NH_2_(CH_3_)_2_]_2_CuBr_4_).

The TGA curves shown in Fig. 4 were measured as the temperature increased. In the TGA curve, the partial decomposition temperature (T_d_) represents a weight loss of 2% at 432 K, which means the material is thermally stable up to 432 K. The molecular weight of the [NH_2_(CH_3_)_2_]_2_CuBr_4_ crystal decreased abruptly as the temperature increased owing to partial decomposition. The amounts of the sample remaining from the partial decomposition of HBr and 2HBr were obtained from a total molecular weight of 475.37 mg using the TGA data and following chemical reactions^35,36^.

1 $$\left\{ {\left[ {{\text{NH}}\left( {{\text{CH}}_{{3}} } \right)_{{2}} } \right]_{{2}} {\text{HBr}} \cdot {\text{CuBr}}_{{2}} \left( {\text{s}} \right) + {\text{HBr}}\left( {\text{g}} \right)} \right\}/\left[ {{\text{NH}}_{{2}} \left( {{\text{CH}}_{{3}} } \right)_{{2}} } \right]_{{2}} {\text{CuBr}}_{{4}} = { 83}.0{4}\%$$

2 $$\left\{ {\left[ {{\text{NH}}\left( {{\text{CH}}_{{3}} } \right)_{{2}} } \right]_{{2}} {\text{CuBr}}_{{2}} \left( {\text{s}} \right) + {\text{2HBr}}\left( {\text{g}} \right)} \right\}/\left[ {{\text{NH}}_{{2}} \left( {{\text{CH}}_{{3}} } \right)_{{2}} } \right]_{{2}} {\text{CuBr}}_{{4}} = {66}.0{5}\%$$

Molecular weight losses of 27% and 34%, which were attributed to the decomposition of HBr and 2HBr, respectively, were observed. The initial weight loss of 27% occurred at 545 K, while the second weight loss of 34% occurred in the range of 577 K.

The endothermic peak at 390 K observed in the DTA curve, which is presented as the differential form of the TGA curve, was in good agreement with the T_C_ shown in the DSC results. Complete weight loss occurred at temperatures above 850 K. Morphology in the crystals with increasing temperature were confirmed by optical polarizing microscopy to understand their thermal properties based on the TGA results. As shown in the inset in Fig. 4, as the temperature increases from 300 to 373 K, the crystal morphology does not change. However, when the temperature reaches 430 K, the single crystal begins to melt, and a considerable amount of it melts at 490 K. From the DSC, TGA, and polarizing microscopy experiments, the T_C_, T_d_, and melting temperature of the crystals were determined to be T_C_ = 388 K, T_d_ = 432 K, and T_m_ = 490 K, respectively.

### ^1^H and ^13^C MAS NMR chemical shifts

**Figure 5:**
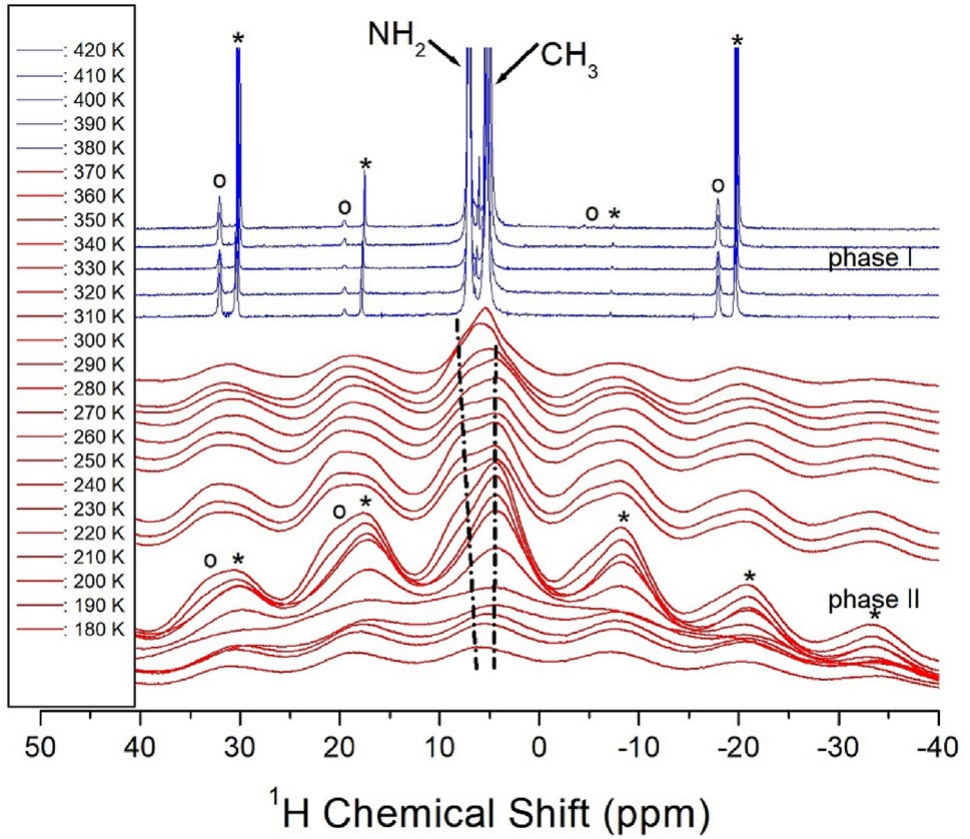
^1^H MAS NMR chemical shifts of NH_2_ and CH_3_ in [NH_2_(CH_3_)_2_]_2_CuBr_4_ at phases II and I. The open circles and asterisks are denoted sidebands for ^1^H in NH_2_ and CH_3_, respectively.

The ^1^H NMR chemical shifts of the [NH_2_(CH_3_)_2_]_2_CuBr_4_ crystals were recorded at phases I and II as shown in Fig. 5. As expected, the two ^1^H signals of NH_2_ and CH_3_ in the cation were detected. At low temperatures, the ^1^H NMR spectra of NH_2_ and CH_3_ completely overlapped and only one signal was obtained. The two ^1^H signals corresponding to NH_2_ and CH_3_ began to separate at temperatures above 260 K. The ^1^H chemical shift for NH_2_ at 300 K was recorded at 6.81 ppm, while that for CH_3_ was obtained at 4.67 ppm. In phase II below T_C_, changes in the chemical shifts with increasing temperature are indicated by dotted lines. The ^1^H chemical shifts for CH_3_ were independent of temperature, whereas those for NH_2_ shifted positively with increasing temperature. The two ^1^H chemical shifts for NH_2_ and CH_3_ changed discontinuously near T_C_.

Furthermore, the linewidths of the ^1^H spectrum in phase II were very broad because the two signals overlapped, whereas those in phase I were relatively thin owing to their complete separation. The sidebands for ^1^H in NH_2_ and CH_3_ are represented by open circles and asterisks, respectively. A disadvantage of spinning is that it may lead to the presence of spinning sidebands. These are spurious signals (i.e. peaks) that result from the modulation of the magnetic field at the spinning frequency. The peaks always appear on either side of any large genuine peak at a separation equal to the spinning rate. Near T_C_, the linewidths rapidly changed from a Gaussian shape to a Lorentzian one.

**Figure 6:**
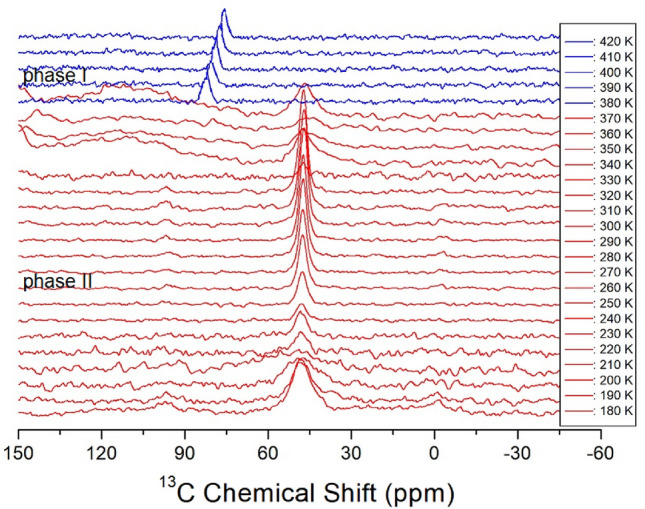
^13^C MAS NMR chemical shifts in [NH_2_(CH_3_)_2_]_2_CuBr_4_ at phases II and I.

The ^13^C NMR chemical shifts of [NH_2_(CH_3_)_2_]_2_CuBr_4_ were also measured in phases I and II as a function of temperature, as shown in Fig. 6. Because the signals above 340 K could not be well detected, chemical shifts above this temperature were measured using the one-pulse method. In phases I and II, only one ^13^C NMR signal was observed, and one signal of ^13^C at 48.10 ppm was observed at 180 K. At T_C_ values above 380 K, this signal suddenly shifted to 82.32 ppm. This abrupt shift is related to the phase transition caused by structural changes between phases I and II. The changes in the ^13^C NMR chemical shifts reflect changes in the coordination geometry around ^13^C.

### Static ^14^N NMR chemical shifts

**Figure 7:**
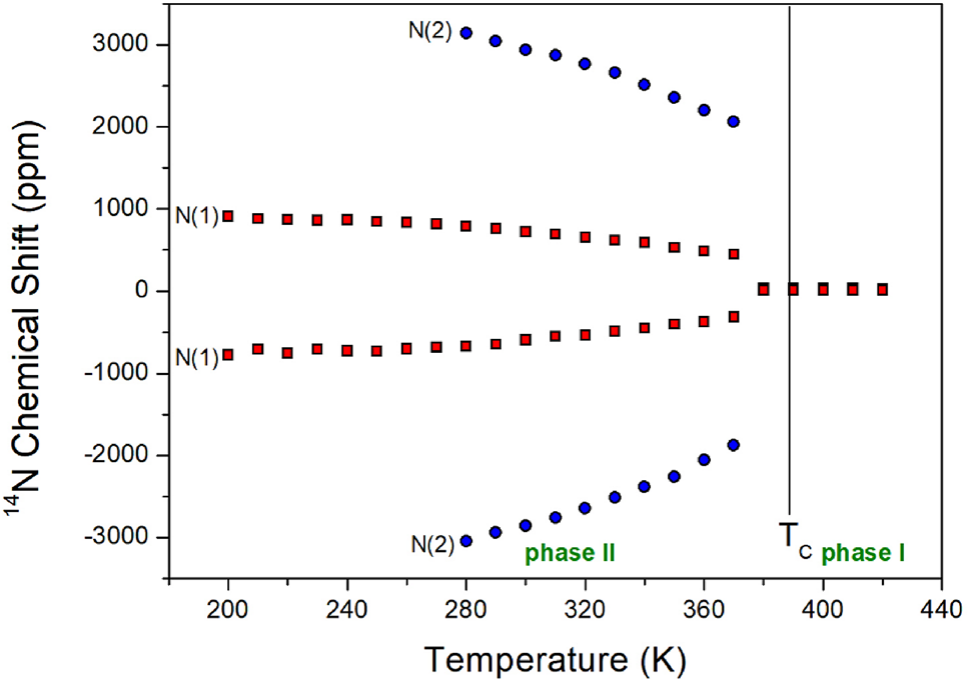
Static ^14^N NMR chemical shifts in [NH_2_(CH_3_)_2_]_2_CuBr_4_ at phases II and I.

The static NMR spectra of ^14^N in NH_2_ at the center of the cation in the [NH_2_(CH_3_)_2_]_2_CuBr_4_ single crystals were recorded as a function of temperature in the range of 200–430 K, and the longest direction of the single crystal and the applied magnetic field of 9.4 T were measured in the directions perpendicular to each other. ^14^N has a spin number of 1, and its signals are expected to show two resonances owing to its quadrupole interactions^37^. The ^14^N NMR spectrum was challenging to obtain because of the low Larmor frequency (28.90 MHz). The chemical shifts in the ^14^N NMR spectra at various temperatures are shown in Fig. 7. The structural geometries of N(1) and N(2) in the two [NH_2_(CH_3_)_2_]^+^ groups were determined based on the ^14^N NMR chemical shifts. The resonance pairs for ^14^N are indicated by the same symbol. The N(1) chemical shifts represented by red squares decrease slightly with increasing temperature, whereas the N(2) chemical shifts represented by blue circles decrease abruptly. The [NH_2_(CH_3_)_2_]_2_CuBr_4_ structure consists of complex CuBr_4_ anions and two [NH_2_(CH_3_)_2_] cations, as shown in Fig. 1. The ^14^N NMR spectrum of N(2) was difficult to detect at low temperatures because of the wide area outside the observed chemical shift range. In phase I, the four ^14^N spectra of the two sets of signals were reduced to two ^14^N spectra of only one set. The abrupt changes in the N(1) and N(2) chemical shifts between phases I and II indicate changes in the coordination geometry of the surrounding environments around ^14^N; N(1) and N(2) with different surrounding environments exist in phase II, whereas only one N site with the same surroundings exists in phase I; temperature changes in ^14^N NMR static chemical shifts may be associated with vibrations of NH_2_ groups at their localization sites.

### ^1^H and ^13^C NMR spin–lattice relaxation times

**Figure 8:**
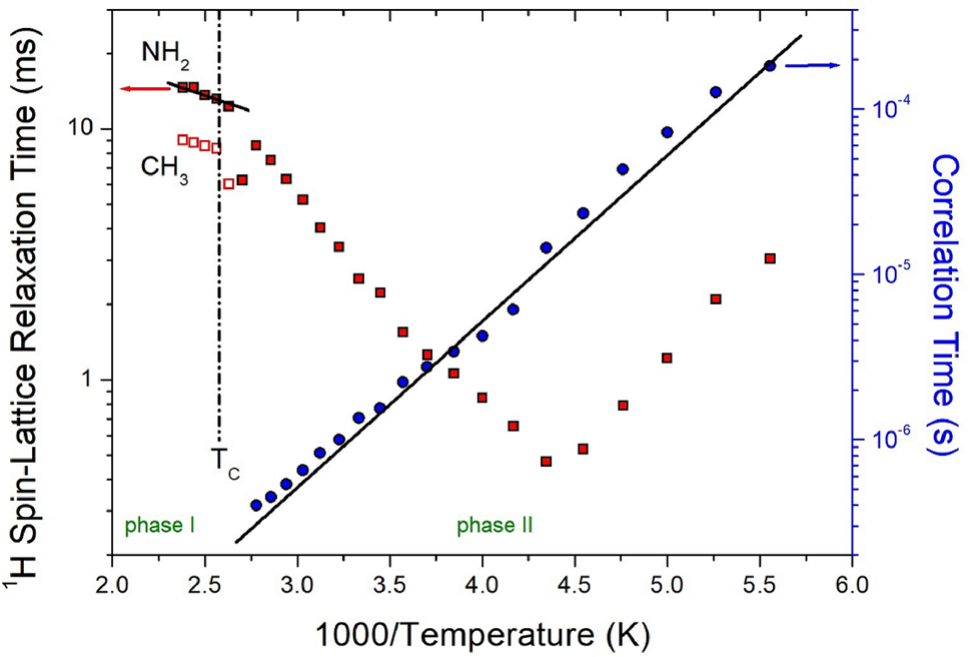
^1^H NMR spin–lattice relaxation times in [NH_2_(CH_3_)_2_]_2_CuBr_4_ at phases II and I. The slope of solid line at phases II and I is represented the activation energy by the correlation times and the relaxation times as a function of inverse temperature, respectively.

**Figure 9:**
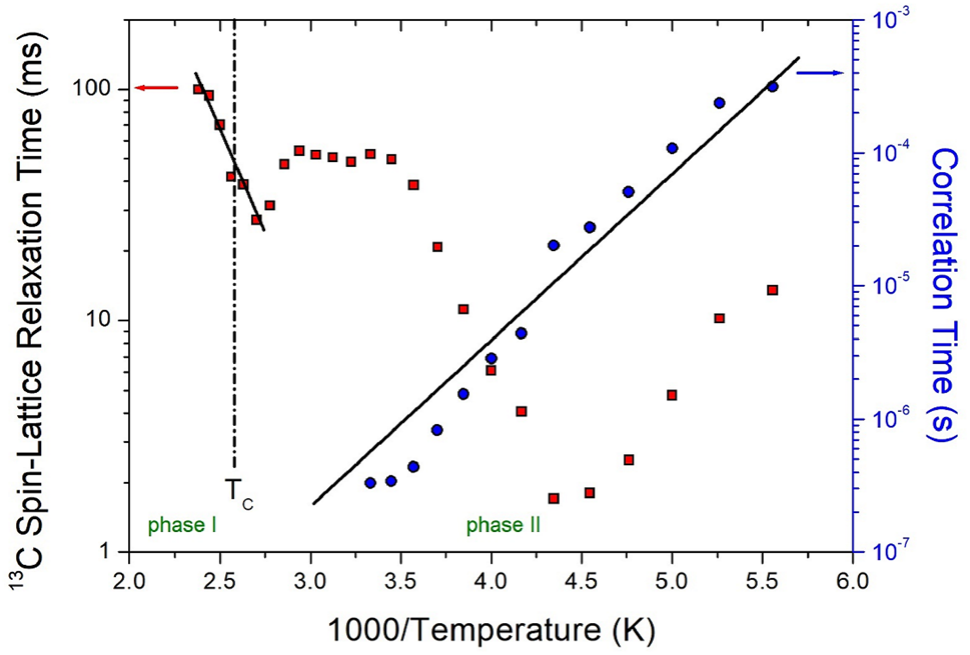
^13^C NMR spin–lattice relaxation times in [NH_2_(CH_3_)_2_]_2_CuBr_4_ at phases II and I. The slope of solid line at phases II and I is represented the activation energy by the correlation times and the relaxation times as a function of inverse temperature, respectively.

The intensities of the ^1^H and ^13^C NMR spectral peaks were measured according to the increase in delay time to obtain T_1ρ_. The decay curves of the changes in intensities and delay times are represented by the following equation^37–39^:

3 $$I\left( t \right) \, = I\left( 0 \right){\text{exp}}( - t/{\text{T}}_{{{1}\rho }} ),$$

where *I*(*t*) is the intensities of the peaks at time *t* and *I*(0) is the intensities of the peaks at time *t* = 0. The T_1ρ_ values for ^1^H and ^13^C in [NH_2_(CH_3_)_2_]_2_CuBr_4_ were obtained using Eq. (3), and the results are shown in Figs. 8 and 9, respectively, as functions of the inverse temperature.

In the case of ^1^H, the chemical shifts for ^1^H in NH_2_ and CH_3_ in phase II were not completely separated, thus, their T_1ρ_ was obtained as a single value. The T_1ρ_ values were strongly dependent on temperature, and T_1ρ_ rapidly decreased as the temperature increased, reaching a minimum value of 0.47 ms at 230 K, as shown in Fig. 8. T_1ρ_ is lengthened again with further increases in temperature, indicating molecular motion according to Bloembergen–Purcell–Pound theory^38^. The minimum value of T_1ρ_ is clearly due to the reorientational motion of ^1^H in NH_2_ and CH_3_. In the case of ^13^C, the T_1ρ_ values shown in Fig. 9 decrease as temperature increases and increase abruptly at temperatures above 230 K. Compared with the T_1ρ_ of ^1^H, the T_1ρ_ of ^13^C showed a minimum value of 1.70 ms at 230 K, and the ^13^C T_1ρ_ pattern indicated active molecular motion. In addition, the T_1ρ_ values of ^1^H and ^13^C rapidly increased in phase I. However, the T_1ρ_ of ^13^C was approximately 10 times longer than that of ^1^H. In phase II, the experimental values of T_1ρ_ can be expressed by the correlation time τ_C_ for reorientational motion and are given by^39,40^:

4 $$\begin{gathered} ({1}/{\text{T}}_{{{1}\rho }} ) \, = {\text{ R}}\{ {4}\tau_{{\text{C}}} /[{1 } + \omega_{{1}}^{{2}} \tau_{{\text{C}}}^{{2}} ] \, + \tau_{{\text{C}}} /[{1 } + \, (\omega_{{\text{C}}} - \omega_{{\text{H}}} )^{{2}} \tau_{{\text{C}}}^{{2}} ] \, + { 3}\tau_{{\text{C}}} /[{1 } + \omega_{{\text{C}}}^{{2}} \tau_{{\text{C}}}^{{2}} ] \, \hfill \\ + { 6}\tau_{{\text{C}}} /[{1 } + \, (\omega_{{\text{C}}} + \omega_{{\text{H}}} )^{{2}} \tau_{{\text{C}}}^{{2}} ] \, + { 6}\tau_{{\text{C}}} /[{1 } + \omega_{{\text{H}}}^{{2}} \tau_{{\text{C}}}^{{2}} ]\} , \hfill \\ \end{gathered}$$

where R is a constant; ω_1_ is the spin-lock field; and ω_C_ and ω_H_ are the Larmor frequencies for carbon and protons, respectively. The τ_C_ value can be obtained from the condition that T_1ρ_ is at a minimum when ω_1_ is 1. As the T_1ρ_ curves exhibited minima, the coefficient R in Eq. (4) can be obtained. Using this R, based on the T_1ρ_ values observed over the temperature range investigated and the frequency power of ω_1_ given in the experiment, the τ_C_ value at various temperatures could be obtained. The ω_1_ values for ^1^H and ^13^C were 69.44 and 50 kHz, respectively. Local field fluctuations are caused by thermal motion, which is activated by thermal energy. τ_C_ is generally expressed as an Arrhenius-type equation based on the E_a_ for molecular motion and temperature as follows^36,37^:

5 $$\tau_{{\text{C}}} = \tau_{{\text{C}}}^{{\text{o}}} {\text{exp}}( - {\text{E}}_{{\text{a}}} /{\text{k}}_{{\text{B}}} {\text{T}})$$

where E_a_ is the activation energy and k_B_ is the Boltzmann constant. The magnitude of E_a_ depends on molecular dynamics. The logarithmic scale of τ_C_ represented by blue circles versus 1000/T is shown in Figs. 8 and 9 to determine the molecular dynamics of the crystal. In phase II, the activation barriers are 19.38 ± 1.70 kJ/mol (^1^H) and 23.88 ± 4.48 kJ/mol (^13^C), and their values are the same within the experimental error. The phenomenon showing the minimum relaxation time for both T_1ρ_ (^1^H) and T_1ρ_ (^13^C) is related to the same relaxation process, namely the reorientation of CH_3_ around its own C3 axis. On the other hand, the changes in the relaxation times for Arrhenius-type random motions as functions of T_1ρ_ for ^1^H and ^13^C in phase I are described in terms of slow motions; for τ_C_ <  < ω_C_ (or ω_H_), T_1ρ_ ~ τ_C_ = τ_0_exp(‒E_a_/k_B_T), where ω_C_ (or ω_H_) denotes the Larmor frequency. The E_a_ values for ^1^H and ^13^C obtained from the logarithmic scale of T_1ρ_ in phase I were 5.11 ± 1.24 and 33.36 ± 9.90 kJ/mol, respectively. The difference in E_a_ values between phases I and II was greater for ^1^H than for ^13^C.

## Conclusion

The physicochemical properties of organic–inorganic hybrid [NH_2_(CH_3_)_2_]_2_CuBr_4_ crystals were investigated in this study. The monoclinic structure of this crystal was determined using SCXRD, and its phase transition temperature T_C_ was determined as 388 K. Its thermal stability at approximately 432 K was not good. ^1^H, ^13^C, and ^14^N NMR spectroscopy provided valuable information on the hydrogen, carbon, and nitrogen environments, as well as their connectivity within the crystal molecule; the ^13^C and ^14^N NMR chemical shifts abruptly changed near the T_C_, thus suggesting that the surrounding environment changes with temperature. By contrast, the ^1^H chemical shifts for NH_2_ rather than CH_3_ changed rapidly over the temperature range investigated. The variations in the ^1^H NMR chemical shifts for NH_2_ and ^14^N NMR chemical shifts according to the changes in temperature in the cation were associated to vibrations of NH_2_ groups at their localization sites. Finally, the ^1^H and ^13^C T_1ρ_ values, which represent the extent of energy transfer surrounding the ^1^H and ^13^C atoms of the cation, changed significantly with the temperature in phase II, indicating that these values are governed by the large degree of freedom for the molecular motions of organic cations. The T_1ρ_ values for ^1^H and ^13^C indicated similar molecular motions, but the T_1ρ_ values in phase I were much longer than those in phase II, indicating difficulties in energy transfer. The ^13^C E_a_ determined from the T_1ρ_ results for molecular motion was larger than the ^1^H E_a_, and the E_a_ for ^1^H and ^13^C near the T_C_ changes greater. Based on their basic mechanism of [NH_2_(CH_3_)_2_]_2_CuBr_4_ crystals, it is expected that the potential applications in various fields will be possible.

### Supplementary Information

Supplementary Information.

## Data Availability

The datasets used and/or analysed during the current study are available from the corresponding author on reasonable request.
