# Supplementary material for: Investigation on the organic–inorganic hybrid crystal [NH2(CH3)2]2CuBr4: structure, phase transition, thermal property, structural geometry, and dynamics
Source: Sci Rep. 2023 Nov 29;13:21008. doi: 10.1038/s41598-023-48015-6 (PMC10687088; doi:10.1038/s41598-023-48015-6)
Supplement: Supplementary file 1 — Supplementary Information. [file 41598_2023_48015_MOESM1_ESM.pdf]

```
data_NH2CH32CuBr4_300K_a

_audit_creation_method            'SHELXL-2018/3'
_shelx_SHELXL_version_number      '2018/3'
_chemical_name_systematic         ?
_chemical_name_common             ?
_chemical_melting_point           ?
_chemical_formula_moiety
'Br4 Cu, 2(C2 H8 N) '
_chemical_formula_sum
 'C4 H16 Br4 Cu N2'
_chemical_formula_weight          475.37

loop_
 _atom_type_symbol
 _atom_type_description
 _atom_type_scat_dispersion_real
 _atom_type_scat_dispersion_imag
 _atom_type_scat_source
 'C'  'C'   0.0033   0.0016
 'International Tables Vol C Tables 4.2.6.8 and 6.1.1.4'
 'H'  'H'   0.0000   0.0000
 'International Tables Vol C Tables 4.2.6.8 and 6.1.1.4'
 'Br'  'Br'  -0.2901   2.4595
 'International Tables Vol C Tables 4.2.6.8 and 6.1.1.4'
 'Cu'  'Cu'   0.3201   1.2651
 'International Tables Vol C Tables 4.2.6.8 and 6.1.1.4'
 'N'  'N'   0.0061   0.0033
 'International Tables Vol C Tables 4.2.6.8 and 6.1.1.4'
```

```
_space_group_crystal_system       monoclinic
_space_group_IT_number            14
_space_group_name_H-M_alt         'P 21/n'
_space_group_name_Hall            '-P 2yn'

_shelx_space_group_comment
;
The symmetry employed for this shelxl refinement is uniquely defined
by the following loop, which should always be used as a source of
symmetry information in preference to the above space-group names.
They are only intended as comments.
;

loop_
 _space_group_symop_operation_xyz
 'x, y, z'
 '-x+1/2, y+1/2, -z+1/2'
 '-x, -y, -z'
 'x-1/2, -y-1/2, z-1/2'

_cell_length_a                    8.8651(5)
_cell_length_b                    11.9938(6)
_cell_length_c                    13.3559(7)
_cell_angle_alpha                 90
_cell_angle_beta                  91.322(2)
_cell_angle_gamma                 90
_cell_volume                      1419.70(13)
_cell_formula_units_Z             4
_cell_measurement_temperature     300(2)
_cell_measurement_reflns_used     4243
_cell_measurement_theta_min       2.28
```

_cell_measurement_theta_max	22.44
_exptl_crystal_description	BLOCK
_exptl_crystal_colour	black
_exptl_crystal_density_meas	?
_exptl_crystal_density_method	?
_exptl_crystal_density_diffrn	2.224
_exptl_crystal_F_000	892
_exptl_transmission_factor_min	?
_exptl_transmission_factor_max	?
_exptl_crystal_size_max	0.263
_exptl_crystal_size_mid	0.122
_exptl_crystal_size_min	0.050
_exptl_absorpt_coefficient_mu	12.756
_shelx_estimated_absorpt_T_min	0.134
_shelx_estimated_absorpt_T_max	0.568
_exptl_absorpt_correction_type	multi-scan
_exptl_absorpt_correction_T_min	0.5387
_exptl_absorpt_correction_T_max	0.7457
_exptl_absorpt_process_details	SADABS
_exptl_absorpt_special_details	?
_diffrn_ambient_temperature	300(2)
_diffrn_radiation_wavelength	0.71073
_diffrn_radiation_type	MoK₩a
_diffrn_source	?
_diffrn_measurement_device_type	'Bruker APEX-II CCD'
_diffrn_measurement_method	'₩f and ₩w scans'
_diffrn_detector_area_resol_mean	?
_diffrn_reflns_number	29993
_diffrn_reflns_av_unetI/netI	0.0605
_diffrn_reflns_av_R_equivalents	0.0951

_diffrn_reflns_limit_h_min -11

_diffrn_reflns_limit_h_max 11

_diffrn_reflns_limit_k_min -15

_diffrn_reflns_limit_k_max 16

_diffrn_reflns_limit_l_min -17

_diffrn_reflns_limit_l_max 17

_diffrn_reflns_theta_min 2.283

_diffrn_reflns_theta_max 28.331

_diffrn_reflns_theta_full 25.242

_diffrn_measured_fraction_theta_max 0.999

_diffrn_measured_fraction_theta_full 1.000

_diffrn_reflns_Laue_measured_fraction_max 0.999

_diffrn_reflns_Laue_measured_fraction_full 1.000

_diffrn_reflns_point_group_measured_fraction_max 0.999

_diffrn_reflns_point_group_measured_fraction_full 1.000

_reflns_number_total 3535

_reflns_number_gt 1823

_reflns_threshold_expression 'I > 2₩s(I)'

_reflns_Friedel_coverage 0.000

_reflns_Friedel_fraction_max .

_reflns_Friedel_fraction_full .

_reflns_special_details

;

Reflections were merged by SHELXL according to the crystal class for the calculation of statistics and refinement.

_reflns_Friedel_fraction is defined as the number of unique Friedel pairs measured divided by the number that would be possible theoretically, ignoring centric projections and systematic absences.

```
;

_computing_data_collection        'Bruker APEX2'
_computing_cell_refinement        'Bruker SAINT'
_computing_data_reduction         'Bruker SAINT'
_computing_structure_solution     'SHELXT 2014/5 (Sheldrick, 2014)'
_computing_structure_refinement 'SHELXL-2018/3 (Sheldrick, 2018)'
_computing_molecular_graphics     'Bruker SHELXTL'
_computing_publication_material   'Bruker SHELXTL'
_refine_special_details           ?
_refine_ls_structure_factor_coef  Fsqd
_refine_ls_matrix_type            full
_refine_ls_weighting_scheme       calc
_refine_ls_weighting_details
'w=1/[₩s^2^(Fo^2^)+(0.0328P)^2^] where P=(Fo^2^+2Fc^2^)/3'
_atom_sites_solution_primary      ?
_atom_sites_solution_secondary    ?
_atom_sites_solution_hydrogens    geom
_refine_ls_hydrogen_treatment     constr
_refine_ls_extinction_method      none
_refine_ls_extinction_coef        .
_refine_ls_number_reflns          3535
_refine_ls_number_parameters      104
_refine_ls_number_restraints      0
_refine_ls_R_factor_all           0.1156
_refine_ls_R_factor_gt            0.0408
_refine_ls_wR_factor_ref          0.0861
_refine_ls_wR_factor_gt           0.0690
_refine_ls_goodness_of_fit_ref    0.990
_refine_ls_restrained_S_all       0.990
_refine_ls_shift/su_max           0.001
```

```
_refine_ls_shift/su_mean          0.000

loop_
 _atom_site_label
 _atom_site_type_symbol
 _atom_site_fract_x
 _atom_site_fract_y
 _atom_site_fract_z
 _atom_site_U_iso_or_equiv
 _atom_site_adp_type
 _atom_site_occupancy
 _atom_site_site_symmetry_order
 _atom_site_calc_flag
 _atom_site_refinement_flags_posn
 _atom_site_refinement_flags_adp
 _atom_site_refinement_flags_occupancy
 _atom_site_disorder_assembly
 _atom_site_disorder_group
Br1 Br 0.09980(7) 0.24101(5) 0.34867(5) 0.0772(2) Uani 1 1 d . . . . .
Br2 Br 0.32557(7) 0.49077(5) 0.38089(5) 0.0749(2) Uani 1 1 d . . . . .
Br3 Br 0.58686(7) 0.25231(5) 0.37992(5) 0.06649(19) Uani 1 1 d . . . . .
Br4 Br 0.31988(7) 0.21123(5) 0.58103(4) 0.0678(2) Uani 1 1 d . . . . .
Cu1 Cu 0.33247(7) 0.29743(5) 0.41995(5) 0.05165(19) Uani 1 1 d . . . . .
N1 N 0.2031(6) 0.3343(4) 0.1171(4) 0.0780(15) Uani 1 1 d . . . . .
H1NA H 0.143022 0.298923 0.073237 0.094 Uiso 1 1 calc R U . . .
H1NB H 0.169547 0.319718 0.178065 0.094 Uiso 1 1 calc R U . . .
N2 N 0.2876(5) 0.4788(4) 0.6590(4) 0.0737(14) Uani 1 1 d . . . . .
H2NA H 0.319405 0.415533 0.631844 0.088 Uiso 1 1 calc R U . . .
H2NB H 0.337112 0.534534 0.630423 0.088 Uiso 1 1 calc R U . . .
C1 C 0.3551(8) 0.2894(6) 0.1097(5) 0.100(2) Uani 1 1 d . . . . .
H1A H 0.393414 0.306002 0.044685 0.149 Uiso 1 1 calc R U . . .
```

H1B H 0.352653 0.210131 0.119118 0.149 Uiso 1 1 calc R U . . .

H1C H 0.419288 0.322679 0.160226 0.149 Uiso 1 1 calc R U . . .

C2 C 0.1911(9) 0.4529(6) 0.0992(6) 0.116(3) Uani 1 1 d . . . . .

H2A H 0.221042 0.469193 0.032167 0.175 Uiso 1 1 calc R U . . .

H2B H 0.255752 0.492071 0.145945 0.175 Uiso 1 1 calc R U . . .

H2C H 0.088608 0.476277 0.107688 0.175 Uiso 1 1 calc R U . . .

C3 C 0.1267(7) 0.4915(7) 0.6357(6) 0.123(3) Uani 1 1 d . . . . .

H3A H 0.108192 0.477863 0.565702 0.184 Uiso 1 1 calc R U . . .

H3B H 0.095601 0.565977 0.651801 0.184 Uiso 1 1 calc R U . . .

H3C H 0.070508 0.439151 0.674411 0.184 Uiso 1 1 calc R U . . .

C4 C 0.3261(8) 0.4776(5) 0.7641(5) 0.090(2) Uani 1 1 d . . . . .

H4A H 0.431398 0.460719 0.773145 0.136 Uiso 1 1 calc R U . . .

H4B H 0.267232 0.421785 0.796850 0.136 Uiso 1 1 calc R U . . .

H4C H 0.305356 0.549302 0.792517 0.136 Uiso 1 1 calc R U . . .

loop_

 _atom_site_aniso_label

 _atom_site_aniso_U_11

 _atom_site_aniso_U_22

 _atom_site_aniso_U_33

 _atom_site_aniso_U_23

 _atom_site_aniso_U_13

 _atom_site_aniso_U_12

Br1 0.0642(4) 0.0819(5) 0.0847(5) -0.0010(3) -0.0124(3) -0.0119(3)

Br2 0.0725(4) 0.0523(4) 0.1001(5) 0.0186(3) 0.0072(4) 0.0067(3)

Br3 0.0610(4) 0.0678(4) 0.0712(4) 0.0093(3) 0.0114(3) 0.0155(3)

Br4 0.0875(5) 0.0613(4) 0.0550(4) 0.0113(3) 0.0087(3) -0.0027(3)

Cu1 0.0560(4) 0.0494(4) 0.0495(4) 0.0034(3) 0.0004(3) 0.0004(3)

N1 0.072(4) 0.091(4) 0.071(3) 0.012(3) -0.008(3) -0.009(3)

N2 0.076(4) 0.067(3) 0.079(4) -0.007(3) 0.013(3) -0.001(3)

C1 0.081(5) 0.115(6) 0.102(6) -0.004(4) -0.003(4) 0.001(5)

C2 0.158(8) 0.067(5) 0.122(6) -0.004(4) -0.033(6) -0.005(5)

C3 0.064(5) 0.174(8) 0.131(7) 0.018(6) 0.009(5) 0.032(5)

C4 0.103(6) 0.091(5) 0.078(5) 0.007(4) 0.016(4) -0.002(4)

```
_geom_special_details
;
 All esds (except the esd in the dihedral angle between two l.s. planes)
 are estimated using the full covariance matrix.  The cell esds are taken
 into account individually in the estimation of esds in distances, angles
 and torsion angles; correlations between esds in cell parameters are only
 used when they are defined by crystal symmetry.  An approximate (isotropic)
 treatment of cell esds is used for estimating esds involving l.s. planes.
;

loop_
 _geom_bond_atom_site_label_1
 _geom_bond_atom_site_label_2
 _geom_bond_distance
 _geom_bond_site_symmetry_2
 _geom_bond_publ_flag
Br1 Cu1 2.3511(8) . ?
Br2 Cu1 2.3772(8) . ?
Br3 Cu1 2.3917(9) . ?
Br4 Cu1 2.3918(8) . ?
N1 C2 1.447(7) . ?
N1 C1 1.456(7) . ?
N1 H1NA 0.8900 . ?
N1 H1NB 0.8900 . ?
N2 C4 1.437(7) . ?
N2 C3 1.461(7) . ?
N2 H2NA 0.8900 . ?
```

N2 H2NB 0.8900 . ?

C1 H1A 0.9600 . ?

C1 H1B 0.9600 . ?

C1 H1C 0.9600 . ?

C2 H2A 0.9600 . ?

C2 H2B 0.9600 . ?

C2 H2C 0.9600 . ?

C3 H3A 0.9600 . ?

C3 H3B 0.9600 . ?

C3 H3C 0.9600 . ?

C4 H4A 0.9600 . ?

C4 H4B 0.9600 . ?

C4 H4C 0.9600 . ?

loop_

_geom_angle_atom_site_label_1

_geom_angle_atom_site_label_2

_geom_angle_atom_site_label_3

_geom_angle

_geom_angle_site_symmetry_1

_geom_angle_site_symmetry_3

_geom_angle_publ_flag

Br1 Cu1 Br2 100.02(3) . . ?

Br1 Cu1 Br3 131.86(4) . . ?

Br2 Cu1 Br3 101.03(3) . . ?

Br1 Cu1 Br4 100.43(3) . . ?

Br2 Cu1 Br4 128.13(4) . . ?

Br3 Cu1 Br4 99.62(3) . . ?

C2 N1 C1 114.6(6) . . ?

C2 N1 H1NA 108.6 . . ?

C1 N1 H1NA 108.6 . . ?

C2 N1 H1NB 108.6 . . ?

C1 N1 H1NB 108.6 . . ?

H1NA N1 H1NB 107.6 . . ?

C4 N2 C3 114.7(5) . . ?

C4 N2 H2NA 108.6 . . ?

C3 N2 H2NA 108.6 . . ?

C4 N2 H2NB 108.6 . . ?

C3 N2 H2NB 108.6 . . ?

H2NA N2 H2NB 107.6 . . ?

N1 C1 H1A 109.5 . . ?

N1 C1 H1B 109.5 . . ?

H1A C1 H1B 109.5 . . ?

N1 C1 H1C 109.5 . . ?

H1A C1 H1C 109.5 . . ?

H1B C1 H1C 109.5 . . ?

N1 C2 H2A 109.5 . . ?

N1 C2 H2B 109.5 . . ?

H2A C2 H2B 109.5 . . ?

N1 C2 H2C 109.5 . . ?

H2A C2 H2C 109.5 . . ?

H2B C2 H2C 109.5 . . ?

N2 C3 H3A 109.5 . . ?

N2 C3 H3B 109.5 . . ?

H3A C3 H3B 109.5 . . ?

N2 C3 H3C 109.5 . . ?

H3A C3 H3C 109.5 . . ?

H3B C3 H3C 109.5 . . ?

N2 C4 H4A 109.5 . . ?

N2 C4 H4B 109.5 . . ?

H4A C4 H4B 109.5 . . ?

N2 C4 H4C 109.5 . . ?

```
H4A C4 H4C 109.5 . . ?

H4B C4 H4C 109.5 . . ?


_refine_diff_density_max    0.409

_refine_diff_density_min   -0.613

_refine_diff_density_rms    0.118


_shelx_res_file

;

TITL NH2CH32CuBr4_300K_a.res in P2(1)/n

    NH2CH32CuBr4_300K_a.res

    created by SHELXL-2018/3 at 15:11:14 on 12-Dec-2022

REM Old TITL NH2CH32CuBr4_300K in P2(1)/n

REM SHELXT solution in P2(1)/n

REM R1 0.171,  Rweak 0.037,  Alpha 0.029,  Orientation as input

REM Formula found by SHELXT:  C4 Br5 N2

CELL  0.71073   8.8651  11.9938  13.3559   90.000   91.322   90.000

ZERR    4.000   0.0005   0.0006   0.0007    0.000    0.002    0.000

LATT  1

SYMM 1/2-X, 1/2+Y, 1/2-Z

SFAC C H BR CU N

UNIT 16 64 16 4 8

TEMP 27.000

SIZE 0.05 0.122 0.263

L.S. 10

BOND $H

LIST 4

FMAP 2

PLAN 20

ACTA
```

```
WGHT    0.032800

FVAR       0.24411

BR1    3      0.099802      0.241013      0.348669      11.00000      0.06421
0.08195 =

         0.08470   -0.00100   -0.01237   -0.01189

BR2    3      0.325572      0.490766      0.380895      11.00000      0.07255
0.05234 =

         0.10007    0.01863    0.00717    0.00667

BR3    3      0.586855      0.252306      0.379919      11.00000      0.06097
0.06777 =

         0.07119    0.00933    0.01138    0.01553

BR4    3      0.319883      0.211229      0.581029      11.00000      0.08748
0.06132 =

         0.05503    0.01131    0.00867   -0.00273

CU1    4      0.332467      0.297431      0.419948      11.00000      0.05605
0.04939 =

         0.04947    0.00337    0.00037    0.00042

N1     5      0.203051      0.334259      0.117131      11.00000      0.07231
0.09070 =

         0.07053    0.01184   -0.00827   -0.00860

AFIX  23

H1NA  2    0.143022    0.298923    0.073237    11.00000   -1.20000

H1NB  2    0.169547    0.319718    0.178065    11.00000   -1.20000

AFIX   0

N2     5      0.287638      0.478836      0.659008      11.00000      0.07574
0.06663 =

         0.07927   -0.00704    0.01257   -0.00063

AFIX  23

H2NA  2    0.319405    0.415533    0.631844    11.00000   -1.20000

H2NB  2    0.337112    0.534534    0.630423    11.00000   -1.20000

AFIX   0

C1     1      0.355051      0.289450      0.109653      11.00000      0.08138
0.11474 =

         0.10236   -0.00363   -0.00252    0.00102
```

```
AFIX 137
H1A   2    0.393414    0.306002    0.044685    11.00000   -1.50000
H1B   2    0.352653    0.210131    0.119118    11.00000   -1.50000
H1C   2    0.419288    0.322679    0.160226    11.00000   -1.50000
AFIX   0
C2     1     0.191109     0.452932     0.099227     11.00000     0.15819
0.06730 =
         0.12189   -0.00409   -0.03260   -0.00540
AFIX 137
H2A   2    0.221042    0.469193    0.032167    11.00000   -1.50000
H2B   2    0.255752    0.492071    0.145945    11.00000   -1.50000
H2C   2    0.088608    0.476277    0.107688    11.00000   -1.50000
AFIX   0
C3     1     0.126680     0.491547     0.635734     11.00000     0.06426
0.17363 =
         0.13057    0.01822    0.00876    0.03194
AFIX 137
H3A   2    0.108192    0.477863    0.565702    11.00000   -1.50000
H3B   2    0.095601    0.565977    0.651801    11.00000   -1.50000
H3C   2    0.070508    0.439151    0.674411    11.00000   -1.50000
AFIX   0
C4     1     0.326097     0.477554     0.764098     11.00000     0.10278
0.09149 =
         0.07766    0.00705    0.01646   -0.00225
AFIX 137
H4A   2    0.431398    0.460719    0.773145    11.00000   -1.50000
H4B   2    0.267232    0.421785    0.796850    11.00000   -1.50000
H4C   2    0.305356    0.549302    0.792517    11.00000   -1.50000
AFIX   0
HKLF 4
```

```
REM  NH2CH32CuBr4_300K_a.res in P2(1)/n

REM wR2 = 0.086077, GooF = S = 0.98952, Restrained GooF = 0.98952 for all
data

REM R1 = 0.040753 for 1823 Fo > 4sig(Fo) and 0.115629 for all 3535 data

REM 104 parameters refined using 0 restraints


END


WGHT      0.0328      0.0000


REM Highest difference peak  0.409,  deepest hole -0.613,  1-sigma level
0.118

Q1    1   0.3277  0.3986  0.4196  11.00000  0.05    0.41

Q2    1   0.3381  0.4165  0.0322  11.00000  0.05    0.40

Q3    1   0.2421  0.2212  0.5440  11.00000  0.05    0.39

Q4    1   0.3751  0.1475  0.5168  11.00000  0.05    0.39

Q5    1   0.2805  0.4176  0.2732  11.00000  0.05    0.38

Q6    1   0.1852  0.2097  0.3113  11.00000  0.05    0.37

Q7    1   0.3064  0.3827  0.7368  11.00000  0.05    0.36

Q8    1   0.0205  0.6163  0.0225  11.00000  0.05    0.36

Q9    1   0.2596  0.4188  0.0052  11.00000  0.05    0.35

Q10   1   0.2801  0.6133  0.6800  11.00000  0.05    0.35

Q11   1   0.3838  0.6132  0.5030  11.00000  0.05    0.34

Q12   1   0.6421  0.2805  0.4602  11.00000  0.05    0.34

Q13   1   0.3051  0.4130  0.7361  11.00000  0.05    0.34

Q14   1   0.0356  0.3961  0.7564  11.00000  0.05    0.33

Q15   1   0.4209  0.1773  0.5411  11.00000  0.05    0.33

Q16   1   0.1931  0.1562  0.6029  11.00000  0.05    0.33

Q17   1   0.4242  0.2821  0.4960  11.00000  0.05    0.33

Q18   1   0.2706  0.3900  0.3587  11.00000  0.05    0.33
```

```
Q19   1   0.3569  0.4069  0.4957  11.00000  0.05    0.33
Q20   1   0.3506  0.6232  0.0172  11.00000  0.05    0.32
;
_shelx_res_checksum   54457

_shelx_hkl_file
;
   1   0   0   -0.50    0.70
   1   0   0   -0.40    0.80
   1   0   0   -0.10    0.50
  -1   0   0    2.00    0.90
  -1   0   0    0.30    0.90
  -1   0   0    1.80    0.70
  -2   0   0 1848.72   47.10
  -2   0   0 1854.91   48.60
   2   0   0 1867.51   47.00
   2   0   0 1809.82   48.10
  -2   0   0 1835.72   41.80
   2   0   0 1760.52   42.90
  -3   0   0    3.30    2.50
  -3   0   0   -3.70    2.60
   3   0   0    2.60    3.00
  -3   0   0   -0.50    1.60
   3   0   0    5.90    4.30
   3   0   0    0.80    1.70
   4   0   0 2301.37   55.19
   4   0   0 2361.16   70.39
  -4   0   0 2440.96   69.19
  -4   0   0 2337.47   66.29
  -4   0   0 2333.77   57.19
  -5   0   0   -6.30    4.40
```

5	0	0	1.50	2.90
5	0	0	3.60	2.80
-5	0	0	-0.40	3.20
-5	0	0	3.50	5.10
6	0	0	114.09	8.90
-6	0	0	83.79	13.90
-6	0	0	98.49	17.00
7	0	0	3.70	4.90
-7	0	0	-4.10	8.50
-7	0	0	4.10	7.80
7	0	0	3.10	4.70
8	0	0	157.48	13.60
8	0	0	174.88	13.70
-8	0	0	189.98	28.00
-8	0	0	178.58	26.20
-9	0	0	-6.50	10.60
9	0	0	23.40	8.70
9	0	0	5.30	6.90
9	0	0	5.80	6.70
-9	0	0	6.10	12.40
10	0	0	41.40	10.80
-10	0	0	40.40	17.90
-10	0	0	1.60	14.90
10	0	0	29.10	9.10
-11	0	0	-0.30	16.50
11	0	0	8.30	9.70
-11	0	0	-21.20	16.30
1	-1	0	215.38	7.20
-1	-1	0	221.78	6.80
-1	-1	0	220.28	7.40
-1	-1	0	216.78	9.00

-1	-1	0	220.68	9.10
1	-1	0	214.58	8.10
1	-1	0	210.98	6.20
-1	-1	0	194.58	8.00
1	1	0	219.48	8.30
1	1	0	202.18	6.60
1	1	0	225.28	9.20
1	1	0	209.98	7.30
-1	1	0	223.18	8.40
-1	1	0	212.78	6.30
-1	1	0	219.88	8.20
1	1	0	220.58	9.10
-1	1	0	212.28	7.30
2	-1	0	70.69	4.40
-2	-1	0	73.99	6.60
-2	-1	0	68.59	6.00
2	-1	0	60.29	6.00
-2	-1	0	79.69	4.60
-2	-1	0	70.19	4.40
2	-1	0	87.59	7.00
2	-1	0	77.89	4.10
-2	-1	0	61.19	6.20
2	1	0	71.39	6.90
2	1	0	65.69	4.40
2	1	0	69.09	4.30
-2	1	0	69.49	6.00
-2	1	0	75.89	4.30
2	1	0	84.39	7.20
3	-1	0	642.54	26.10
-3	-1	0	656.03	26.30
-3	-1	0	653.73	19.70

3	-1	0	652.63	19.00
3	-1	0	664.43	24.80
-3	-1	0	639.84	25.50
-3	1	0	700.83	25.50
3	1	0	665.23	19.80
3	1	0	663.63	19.60
3	1	0	670.53	26.70
3	1	0	649.04	25.50
-3	1	0	633.34	19.10
-3	1	0	686.33	26.20
4	-1	0	3098.19	87.49
4	-1	0	3416.56	76.09
-4	-1	0	3269.27	77.09
4	-1	0	3183.08	96.19
-4	-1	0	3307.57	89.39
-4	-1	0	3356.76	87.89
4	1	0	3210.88	89.99
4	1	0	3299.97	76.29
-4	1	0	3344.07	76.59
4	1	0	3378.26	92.69
-4	1	0	3364.16	86.69
4	1	0	3353.06	76.89
-4	1	0	3327.07	88.49
-5	-1	0	149.59	17.90
5	-1	0	125.59	8.60
-5	-1	0	132.89	14.70
-5	1	0	122.59	16.00
5	1	0	128.39	9.00
-5	1	0	122.19	8.90
5	1	0	117.89	8.90
-5	1	0	121.39	15.50

6	−1	0	153.58	10.80
6	−1	0	151.88	9.80
−6	−1	0	155.18	19.10
−6	−1	0	143.49	19.50
−6	1	0	156.18	19.60
6	1	0	134.09	10.30
6	1	0	128.09	10.00
−6	1	0	143.09	19.00
7	−1	0	0.70	4.70
−7	−1	0	−1.50	8.40
7	−1	0	4.70	4.50
7	−1	0	7.20	5.10
−7	−1	0	9.90	9.40
7	1	0	1.80	5.00
−7	1	0	−0.60	7.40
7	1	0	6.40	5.20
−7	1	0	5.80	9.10
8	−1	0	399.26	20.80
−8	−1	0	374.86	33.00
−8	−1	0	430.86	33.70
8	−1	0	385.76	20.00
8	−1	0	439.16	23.10
−8	1	0	443.96	33.50
8	1	0	437.36	21.50
8	1	0	420.06	21.50
−8	1	0	454.35	35.40
9	−1	0	5.80	7.30
−9	−1	0	25.70	14.30
9	−1	0	7.10	8.10
−9	−1	0	21.90	14.10
9	−1	0	22.10	7.60

9	1	0	18.20	7.50
-9	1	0	16.60	12.30
-9	1	0	12.90	13.20
-10	-1	0	7.80	15.50
-10	-1	0	-4.70	15.60
10	-1	0	12.30	9.60
-10	1	0	29.30	15.00
-10	1	0	-6.30	13.50
10	1	0	-6.10	7.90
-11	-1	0	-6.90	16.60
11	-1	0	10.20	10.50
-11	-1	0	22.40	20.00
11	1	0	-20.80	11.80
11	1	0	8.20	10.30
-11	1	0	10.30	19.40
-11	1	0	-9.00	17.80
-11	1	0	5.00	16.60
0	-2	0	3517.85	83.39
0	-2	0	3838.82	79.29
0	2	0	3334.87	83.19
0	2	0	3328.87	76.49
0	2	0	3932.61	79.29
0	2	0	3722.33	76.19
-1	-2	0	188.58	9.20
1	-2	0	205.68	8.20
1	-2	0	200.98	11.50
1	-2	0	212.58	5.50
-1	-2	0	190.58	6.90
1	-2	0	213.58	10.00
-1	-2	0	188.58	9.50
-1	-2	0	199.18	8.50

−1	−2	0	211.38	11.00
1	−2	0	222.98	8.20
1	2	0	221.68	10.00
1	2	0	209.58	7.20
−1	2	0	218.38	9.20
1	2	0	211.88	9.30
−1	2	0	211.58	8.10
−1	2	0	206.28	11.60
1	2	0	217.28	9.40
1	2	0	208.48	8.60
−1	2	0	202.38	6.30
−2	−2	0	333.17	12.50
−2	−2	0	344.67	15.70
−2	−2	0	319.87	15.50
2	−2	0	331.37	10.30
2	−2	0	336.27	14.70
−2	−2	0	369.76	14.60
−2	−2	0	327.77	11.20
2	−2	0	328.47	13.80
2	−2	0	337.47	11.90
−2	2	0	319.77	13.70
−2	2	0	313.67	10.50
2	2	0	359.76	16.00
2	2	0	360.46	16.00
−2	2	0	348.07	17.30
2	2	0	347.47	12.70
−2	2	0	362.36	16.60
2	2	0	335.97	11.50
−2	2	0	341.37	12.70
3	−2	0	11.80	2.70
−3	−2	0	8.80	4.10

−3	−2	0	12.70	3.10
−3	−2	0	20.00	5.30
−3	−2	0	10.20	4.10
3	−2	0	11.10	3.10
−3	−2	0	11.60	3.00
3	−2	0	15.40	4.50
3	−2	0	7.90	4.30
3	2	0	8.40	4.50
−3	2	0	14.50	3.10
−3	2	0	8.80	3.80
3	2	0	18.70	5.00
−3	2	0	13.80	4.30
3	2	0	10.90	3.00
3	2	0	7.20	2.40
4	−2	0	822.62	26.50
−4	−2	0	845.12	32.50
4	−2	0	877.61	24.80
−4	−2	0	858.91	35.50
4	−2	0	773.42	31.40
−4	−2	0	887.91	26.20
4	−2	0	845.52	34.50
−4	−2	0	856.31	34.50
−4	2	0	864.21	34.00
4	2	0	894.51	26.40
4	2	0	853.41	34.60
−4	2	0	848.42	25.40
4	2	0	840.22	26.50
−4	2	0	868.21	32.80
4	2	0	891.31	35.90
−5	−2	0	13.60	7.10
5	−2	0	12.90	3.80

−5	−2	0	3.10	5.90
−5	−2	0	13.60	6.80
5	−2	0	14.00	4.60
5	−2	0	−5.20	7.10
−5	2	0	7.50	6.30
−5	2	0	10.60	3.70
5	2	0	8.00	4.00
−5	2	0	1.60	5.40
5	2	0	7.40	3.90
−6	−2	0	257.27	22.50
6	−2	0	230.38	14.20
−6	−2	0	251.07	22.20
6	−2	0	238.08	13.10
6	2	0	243.48	14.20
−6	2	0	256.87	21.60
−6	2	0	260.47	21.30
6	2	0	244.08	14.20
7	−2	0	21.20	5.90
−7	−2	0	4.80	10.20
7	−2	0	22.30	6.30
−7	−2	0	7.50	9.80
7	2	0	12.00	6.60
−7	2	0	12.00	8.90
7	2	0	35.50	7.20
−7	2	0	20.00	9.60
8	−2	0	9.10	5.60
−8	−2	0	−4.00	9.10
−8	−2	0	8.00	12.20
8	−2	0	0.90	5.00
−8	2	0	1.90	9.80
8	2	0	3.20	5.50

−8	2	0	6.50	11.00
9	−2	0	0.10	7.70
9	−2	0	−5.00	6.20
−9	−2	0	21.60	15.10
−9	−2	0	3.50	11.10
9	2	0	12.90	8.20
−9	2	0	15.40	12.00
−9	2	0	−7.30	10.80
−10	−2	0	21.30	20.10
10	−2	0	18.30	8.10
10	−2	0	38.80	10.20
−10	−2	0	17.80	15.80
−10	2	0	49.10	19.00
10	2	0	11.10	8.50
−10	2	0	46.50	17.50
−10	2	0	21.90	20.00
11	−2	0	14.90	10.70
−11	−2	0	−5.00	18.30
−11	−2	0	10.60	17.30
11	2	0	−5.30	10.20
−11	2	0	−29.60	17.30
−11	2	0	−2.70	20.40
−11	2	0	−14.80	15.00
0	−3	0	−0.10	2.60
0	−3	0	0.00	1.40
0	−3	0	2.00	1.60
0	−3	0	−1.50	1.40
0	3	0	−0.60	0.90
0	3	0	0.40	1.10
0	3	0	−2.70	2.20
0	3	0	−0.50	1.50

-1	-3	0	27.00	4.80
-1	-3	0	26.70	4.40
-1	-3	0	35.50	5.10
-1	-3	0	27.00	4.50
1	-3	0	23.10	4.10
1	-3	0	29.40	4.10
-1	-3	0	29.40	3.10
1	-3	0	34.10	3.60
1	-3	0	34.70	6.30
1	3	0	39.10	6.40
1	3	0	30.10	4.30
1	3	0	28.60	4.40
-1	3	0	32.40	2.40
-1	3	0	29.80	3.00
-1	3	0	44.00	7.50
-1	3	0	36.80	5.60
-1	3	0	36.80	4.60
-2	-3	0	386.96	13.00
2	-3	0	389.96	16.00
-2	-3	0	391.26	18.10
-2	-3	0	397.16	18.20
2	-3	0	399.16	14.50
2	-3	0	368.56	15.90
-2	-3	0	402.56	15.50
2	-3	0	401.56	10.60
-2	-3	0	401.76	16.70
-2	3	0	387.36	20.40
-2	3	0	390.76	12.50
-2	3	0	389.96	15.40
-2	3	0	407.76	15.00
2	3	0	396.36	13.40

2	3	0	405.66	17.80
2	3	0	412.26	18.30
2	3	0	373.86	14.80
2	3	0	365.76	16.20
−2	3	0	398.06	16.30
−3	−3	0	349.77	17.20
3	−3	0	330.57	17.50
−3	−3	0	360.16	15.20
3	−3	0	345.27	14.10
−3	−3	0	380.16	19.90
−3	−3	0	344.37	13.20
3	−3	0	321.67	16.40
−3	−3	0	377.16	20.00
−3	3	0	341.07	14.90
3	3	0	350.86	15.00
−3	3	0	335.67	12.50
−3	3	0	339.07	16.80
3	3	0	348.87	19.20
3	3	0	378.06	19.70
−3	3	0	355.36	17.50
3	3	0	332.87	17.10
−4	−3	0	1666.53	56.29
4	−3	0	1642.04	45.00
−4	−3	0	1716.53	44.90
−4	−3	0	1704.83	55.69
4	−3	0	1684.43	54.49
−4	−3	0	1836.02	53.49
4	−3	0	1723.73	42.50
4	−3	0	1732.63	51.99
4	3	0	1683.73	46.60
4	3	0	1622.64	55.19

4	3	0	1692.83	45.20
4	3	0	1645.14	54.59
-4	3	0	1726.03	43.80
-4	3	0	1856.91	60.29
-4	3	0	1811.52	60.19
-4	3	0	1751.62	52.39
-5	-3	0	334.27	23.10
-5	-3	0	341.27	21.50
5	-3	0	332.27	23.90
-5	-3	0	356.76	24.70
5	-3	0	358.66	16.40
-5	3	0	373.46	23.40
-5	3	0	332.47	15.40
-5	3	0	344.37	21.70
5	3	0	356.46	17.20
6	-3	0	84.79	7.90
-6	-3	0	78.49	15.60
-6	-3	0	140.99	20.00
6	-3	0	88.59	10.10
6	3	0	85.89	10.60
6	3	0	97.99	11.00
-6	3	0	83.89	13.30
-6	3	0	75.39	14.50
-6	3	0	91.59	17.50
7	-3	0	16.60	5.60
7	-3	0	7.30	5.30
-7	-3	0	33.70	11.70
-7	-3	0	14.40	11.40
-7	3	0	27.70	11.50
-7	3	0	15.90	9.40
-7	3	0	9.70	9.40

7	3	0	10.90	5.90
-8	-3	0	287.27	29.00
8	-3	0	246.08	15.20
-8	-3	0	154.78	23.70
8	3	0	230.88	17.40
-8	3	0	294.27	26.80
-8	3	0	251.17	26.90
-8	3	0	250.67	25.90
-9	-3	0	7.60	12.60
9	-3	0	3.60	7.40
9	-3	0	21.40	7.30
-9	-3	0	3.80	12.40
-9	3	0	3.50	13.90
9	3	0	17.70	8.30
-9	3	0	-16.70	12.00
-9	3	0	-0.50	12.00
-10	-3	0	-8.20	13.70
-10	-3	0	-12.10	13.20
10	-3	0	2.10	7.90
-10	3	0	1.10	17.20
10	3	0	1.00	7.30
-10	3	0	-2.80	12.50
-10	3	0	23.80	15.10
11	-3	0	-3.90	10.60
-11	-3	0	-9.20	31.60
-11	3	0	16.90	17.10
-11	3	0	-4.30	18.00
-11	3	0	-27.70	17.80
11	3	0	12.30	10.40
0	-4	0	9999.00	203.18
0	-4	0	9870.81	202.58

0	-4	0	9158.38	211.48
0	-4	0	9376.36	201.68
0	4	0	8492.85	210.28
0	4	0	9364.56	196.58
0	4	0	9987.40	202.68
0	4	0	9638.04	195.88
0	4	0	8606.54	196.48
-1	-4	0	139.89	11.00
-1	-4	0	146.09	6.50
-1	-4	0	143.59	10.10
-1	-4	0	148.89	9.30
1	-4	0	148.59	8.80
-1	-4	0	128.49	9.00
1	-4	0	137.39	9.40
1	-4	0	127.89	11.60
-1	4	0	142.29	8.80
-1	4	0	159.48	9.10
1	4	0	173.98	10.90
-1	4	0	149.69	6.60
1	4	0	126.99	10.10
1	4	0	141.29	6.90
-1	4	0	148.29	5.80
1	4	0	135.89	8.90
-1	4	0	165.68	13.90
1	4	0	142.69	8.40
2	-4	0	145.09	13.40
-2	-4	0	136.19	9.30
-2	-4	0	134.19	10.80
-2	-4	0	125.69	10.00
-2	-4	0	131.69	7.10
2	-4	0	127.39	8.40

2	−4	0	131.19	9.80
−2	−4	0	132.09	11.20
2	−4	0	129.59	9.10
2	4	0	117.89	9.10
2	4	0	124.59	9.80
−2	4	0	142.49	9.00
2	4	0	136.69	10.50
−2	4	0	124.79	8.20
−2	4	0	130.49	9.80
−2	4	0	135.59	13.20
2	4	0	131.19	7.40
2	4	0	125.19	10.90
−2	4	0	131.99	6.70
−3	−4	0	1.10	2.60
3	−4	0	1.80	1.40
3	−4	0	4.70	4.00
3	−4	0	0.30	2.20
3	−4	0	−8.60	3.10
−3	−4	0	−1.80	3.60
−3	−4	0	5.40	5.10
−3	−4	0	0.80	2.20
−3	−4	0	3.50	4.00
−3	4	0	−0.10	2.00
3	4	0	1.10	3.90
−3	4	0	1.00	5.60
−3	4	0	−2.20	3.70
3	4	0	0.10	3.20
−3	4	0	1.30	3.50
−3	4	0	−5.60	2.30
3	4	0	1.90	2.60
3	4	0	−5.00	4.30

3	4	0	4.80	3.80
4	−4	0	1036.40	38.90
−4	−4	0	1002.40	33.10
−4	−4	0	1004.20	40.60
−4	−4	0	1020.00	41.50
4	−4	0	1037.30	31.50
4	−4	0	983.90	35.60
−4	−4	0	1008.10	30.00
4	−4	0	1039.60	27.00
−4	−4	0	1005.30	42.80
4	4	0	982.90	39.80
−4	4	0	942.41	36.00
4	4	0	1027.10	40.50
−4	4	0	1074.99	45.70
−4	4	0	955.50	28.90
4	4	0	999.00	32.70
4	4	0	1022.60	31.10
−4	4	0	966.20	32.10
−5	−4	0	3.10	3.80
5	−4	0	2.80	3.60
−5	−4	0	0.70	6.10
−5	−4	0	12.40	6.90
5	−4	0	−14.50	7.40
5	−4	0	3.90	3.10
−5	4	0	6.00	5.30
5	4	0	1.40	3.80
−5	4	0	−1.70	6.30
5	4	0	−1.40	4.90
−5	4	0	−1.10	3.60
−5	4	0	1.50	5.60
6	−4	0	−3.10	4.20

−6	−4	0	−0.30	7.80
−6	−4	0	0.20	7.90
6	−4	0	−1.50	3.20
−6	4	0	4.30	8.10
−6	4	0	−10.40	7.60
−6	4	0	2.20	7.00
6	4	0	−2.60	4.40
7	−4	0	4.70	6.00
−7	−4	0	3.40	9.40
7	−4	0	11.80	7.90
7	−4	0	3.00	4.00
−7	−4	0	0.50	9.30
−7	4	0	5.10	9.20
−7	4	0	15.60	10.30
7	4	0	7.20	6.10
−7	4	0	3.60	8.30
−8	−4	0	71.09	18.80
8	−4	0	50.49	9.00
8	−4	0	58.99	8.90
−8	−4	0	44.10	18.30
−8	4	0	61.79	16.10
−8	4	0	37.70	15.40
−8	4	0	82.29	19.10
8	4	0	44.30	8.90
9	−4	0	−2.00	8.50
−9	−4	0	5.90	15.00
−9	−4	0	0.30	13.10
9	−4	0	4.80	6.80
−9	4	0	7.20	15.50
−9	4	0	14.90	12.10
−9	4	0	10.20	16.30

9	4	0	5.70	8.50
10	-4	0	21.90	9.50
-10	-4	0	28.20	20.50
-10	-4	0	14.50	19.20
-10	4	0	10.30	17.60
-10	4	0	38.10	18.80
-10	4	0	9.80	15.00
10	4	0	15.40	9.90
-11	-4	0	-14.80	21.30
11	-4	0	14.30	11.30
-11	-4	0	-13.40	20.90
-11	4	0	-12.80	17.70
11	4	0	8.30	11.10
-11	4	0	5.80	19.30
-11	4	0	-1.00	17.20
0	-5	0	-0.60	4.30
0	-5	0	1.00	2.60
0	-5	0	7.80	3.80
0	5	0	1.20	1.50
0	5	0	3.60	1.80
0	5	0	0.30	1.70
0	5	0	6.50	4.00
0	5	0	4.80	2.90
-1	-5	0	240.08	13.50
1	-5	0	284.97	19.70
-1	-5	0	295.27	15.50
1	-5	0	272.57	13.70
-1	-5	0	254.57	13.30
1	-5	0	229.08	13.30
-1	-5	0	265.17	9.90
-1	-5	0	265.27	17.30

-1	5	0	268.47	9.10
-1	5	0	238.38	18.70
1	5	0	259.67	14.80
1	5	0	269.17	12.10
1	5	0	298.67	17.00
1	5	0	252.57	10.60
-1	5	0	264.27	13.40
1	5	0	275.37	14.30
-1	5	0	254.47	9.30
2	-5	0	427.46	19.20
2	-5	0	423.36	17.80
-2	-5	0	440.46	21.70
2	-5	0	400.96	28.70
-2	-5	0	422.46	21.00
2	-5	0	417.86	17.10
-2	-5	0	422.06	19.20
-2	-5	0	414.06	21.10
-2	-5	0	426.86	14.90
-2	5	0	416.86	13.80
-2	5	0	455.05	26.80
-2	5	0	427.06	18.20
-2	5	0	411.96	18.90
-2	5	0	411.86	15.80
2	5	0	433.06	20.70
2	5	0	459.05	22.20
2	5	0	429.96	19.50
2	5	0	438.46	18.90
2	5	0	438.56	15.90
-3	-5	0	480.25	25.50
-3	-5	0	501.75	19.10
3	-5	0	490.25	19.50

−3	−5	0	473.75	23.00
3	−5	0	487.65	25.60
−3	−5	0	499.45	21.70
3	−5	0	469.55	21.70
3	5	0	492.75	18.30
−3	5	0	542.45	30.70
−3	5	0	462.85	16.70
3	5	0	448.16	24.40
3	5	0	479.85	23.60
−3	5	0	478.35	20.40
−3	5	0	436.06	26.30
3	5	0	483.15	23.50
3	5	0	470.55	21.00
−3	5	0	466.35	20.40
−4	−5	0	970.80	41.60
4	−5	0	943.51	37.50
4	−5	0	945.01	22.60
−4	−5	0	984.80	34.20
4	−5	0	1021.40	36.90
4	−5	0	989.30	23.20
−4	−5	0	993.70	41.40
−4	−5	0	948.91	36.90
4	−5	0	947.01	31.30
−4	5	0	1046.80	37.80
4	5	0	964.30	39.70
4	5	0	974.20	37.40
−4	5	0	969.40	29.10
−4	5	0	965.90	53.09
−4	5	0	1045.60	34.50
4	5	0	918.61	40.00
4	5	0	964.60	30.90

-5	-5	0	5.30	8.40
5	-5	0	13.90	4.50
-5	-5	0	2.90	4.60
5	-5	0	1.90	9.10
5	-5	0	7.90	2.70
5	-5	0	2.40	2.70
-5	5	0	1.10	4.50
-5	5	0	-1.80	7.20
-5	5	0	7.80	6.60
5	5	0	1.40	3.80
-5	5	0	4.10	5.30
-6	-5	0	75.59	16.60
-6	-5	0	96.09	12.40
6	-5	0	103.59	8.40
6	-5	0	111.99	7.60
-6	-5	0	109.49	19.20
-6	5	0	125.29	18.30
6	5	0	72.39	10.30
-6	5	0	111.99	21.60
-6	5	0	67.39	14.10
-7	-5	0	37.90	15.00
7	-5	0	32.90	7.80
7	-5	0	30.60	6.60
7	-5	0	34.60	6.20
-7	-5	0	37.40	14.00
7	5	0	18.50	8.10
-7	5	0	32.50	11.60
-7	5	0	39.80	15.50
-7	5	0	25.20	9.10
-8	-5	0	110.19	22.20
8	-5	0	87.69	11.80

8	−5	0	109.49	11.00
−8	−5	0	165.48	29.10
8	−5	0	107.79	10.80
−8	5	0	132.29	21.70
8	5	0	123.09	15.60
−8	5	0	74.99	21.80
−8	5	0	122.29	22.00
9	−5	0	14.30	7.70
−9	−5	0	−14.80	14.40
−9	−5	0	8.60	17.60
−9	5	0	−6.00	11.80
−9	5	0	−4.20	16.00
9	5	0	13.10	8.80
−9	5	0	12.80	14.00
10	−5	0	−1.90	8.40
−10	−5	0	−3.90	18.40
−10	−5	0	12.90	18.00
−10	5	0	−3.30	17.50
−10	5	0	5.20	13.50
10	5	0	4.60	11.00
−10	5	0	6.10	15.50
−11	−5	0	−13.50	20.40
−11	−5	0	7.70	22.10
−11	5	0	−46.10	18.50
−11	5	0	−38.30	16.20
11	5	0	−0.20	12.10
0	−6	0	56.09	12.30
0	−6	0	57.59	8.20
0	−6	0	61.99	8.80
0	−6	0	58.29	8.40
0	6	0	70.19	4.90

0	6	0	56.69	13.00
0	6	0	61.39	5.10
0	6	0	63.59	8.60
0	6	0	51.89	5.70
1	-6	0	26.00	8.90
1	-6	0	29.10	6.00
1	-6	0	43.00	8.00
-1	-6	0	32.10	8.90
-1	-6	0	29.50	5.60
-1	-6	0	30.50	6.70
-1	-6	0	39.90	4.60
-1	6	0	29.80	3.60
-1	6	0	19.00	8.20
1	6	0	39.70	6.20
1	6	0	32.80	4.90
1	6	0	25.50	6.00
1	6	0	45.50	9.10
-1	6	0	47.70	7.60
1	6	0	31.20	7.20
-1	6	0	24.90	3.70
1	6	0	37.80	5.20
-2	-6	0	623.34	27.20
2	-6	0	649.34	34.70
-2	-6	0	637.24	28.30
-2	-6	0	628.94	19.50
-2	-6	0	620.04	29.00
-2	-6	0	614.24	26.00
2	-6	0	639.94	26.20
2	-6	0	597.84	24.20
2	6	0	648.74	28.10
-2	6	0	600.94	18.60

2	6	0	565.74	29.90
-2	6	0	691.43	25.60
2	6	0	619.24	24.70
-2	6	0	617.34	20.20
2	6	0	627.24	21.10
-2	6	0	661.23	35.10
2	6	0	624.34	29.10
3	-6	0	10.00	5.80
3	-6	0	5.40	5.60
3	-6	0	18.20	5.50
-3	-6	0	11.10	3.50
-3	-6	0	24.00	6.30
-3	6	0	23.90	4.80
-3	6	0	20.90	5.20
-3	6	0	12.60	4.70
-3	6	0	16.40	7.00
-3	6	0	18.40	8.00
3	6	0	26.00	9.20
3	6	0	10.30	5.50
3	6	0	18.00	5.00
3	6	0	2.80	5.80
-4	-6	0	95.49	14.20
-4	-6	0	89.59	16.20
4	-6	0	100.79	10.10
-4	-6	0	96.99	15.80
-4	-6	0	108.79	11.30
4	-6	0	114.79	15.40
4	6	0	92.29	13.10
-4	6	0	126.29	12.00
4	6	0	122.19	16.50
-4	6	0	114.89	14.60

4	6	0	95.79	8.80
−4	6	0	94.09	17.70
−4	6	0	126.99	9.60
4	6	0	86.39	13.90
−5	−6	0	13.60	9.00
−5	−6	0	9.50	5.50
−5	−6	0	1.60	8.80
5	−6	0	0.80	9.80
5	−6	0	3.50	4.40
−5	6	0	11.70	11.00
−5	6	0	6.30	6.80
5	6	0	7.70	5.20
−5	6	0	10.10	6.80
−5	6	0	6.60	5.10
−5	6	0	9.90	7.80
6	−6	0	491.35	22.70
−6	−6	0	548.25	35.60
6	−6	0	503.35	16.50
−6	−6	0	476.05	35.00
−6	−6	0	501.35	25.70
−6	6	0	531.55	38.80
−6	6	0	437.96	28.60
6	6	0	489.25	23.60
−6	6	0	532.65	30.30
−7	−6	0	19.00	11.90
7	−6	0	37.30	8.40
7	−6	0	41.70	5.30
−7	−6	0	43.90	16.60
7	6	0	32.20	8.60
−7	6	0	20.90	11.70
−7	6	0	39.70	13.10

-7	6	0	36.80	17.90
8	-6	0	11.60	4.80
-8	-6	0	2.60	14.30
8	-6	0	1.10	6.90
8	-6	0	6.20	4.80
-8	6	0	6.60	10.90
-8	6	0	8.10	12.40
-8	6	0	5.30	11.70
8	6	0	2.80	7.60
-9	-6	0	-15.40	15.50
-9	-6	0	-1.30	16.10
9	-6	0	-7.50	7.30
-9	6	0	-2.20	12.60
9	6	0	-0.20	9.60
-9	6	0	1.00	15.90
-9	6	0	9.80	15.80
-10	-6	0	18.20	19.40
-10	-6	0	20.70	21.30
-10	6	0	3.30	19.50
10	6	0	10.60	11.60
-10	6	0	-2.30	15.90
-10	6	0	-17.40	14.90
0	-7	0	0.90	3.60
0	-7	0	-5.40	3.60
0	-7	0	-7.40	6.70
0	-7	0	4.90	5.90
0	7	0	-0.50	2.30
0	7	0	-4.10	5.80
0	7	0	4.40	2.10
0	7	0	-0.30	2.30
1	-7	0	2.20	8.00

-1	-7	0	2.20	4.10
1	-7	0	14.10	5.70
-1	-7	0	-3.90	7.60
1	-7	0	6.30	4.10
1	-7	0	3.10	3.50
-1	-7	0	0.80	4.70
-1	7	0	-0.30	2.10
1	7	0	3.10	3.60
-1	7	0	0.20	1.60
-1	7	0	10.70	7.50
1	7	0	0.00	5.70
1	7	0	8.10	3.60
-1	7	0	-0.40	2.40
-1	7	0	0.50	2.70
2	-7	0	1131.39	39.50
-2	-7	0	1140.39	45.00
2	-7	0	1130.79	38.10
-2	-7	0	1137.59	40.50
-2	-7	0	1132.99	30.90
-2	-7	0	1118.79	40.00
-2	-7	0	1083.79	41.60
2	7	0	1146.39	33.20
2	7	0	1104.39	42.40
-2	7	0	1129.19	37.90
2	7	0	1163.58	36.80
-2	7	0	1121.99	30.50
-2	7	0	1094.89	51.29
-2	7	0	1094.89	30.00
2	7	0	1130.99	32.90
2	7	0	1094.39	41.10
-3	-7	0	97.29	14.30

−3	−7	0	96.19	15.20
−3	−7	0	86.59	7.60
−3	−7	0	85.39	11.80
3	−7	0	76.79	12.30
3	−7	0	76.09	10.70
3	−7	0	104.99	12.30
−3	−7	0	71.29	13.30
3	7	0	77.79	12.80
3	7	0	66.69	11.80
3	7	0	94.19	15.40
−3	7	0	73.19	11.00
−3	7	0	82.89	8.80
3	7	0	74.09	8.80
−3	7	0	99.99	12.00
−3	7	0	79.79	7.50
−3	7	0	61.99	14.30
−4	−7	0	2.80	6.60
4	−7	0	29.60	9.10
−4	−7	0	34.90	12.00
−4	−7	0	−7.60	8.20
4	−7	0	16.80	6.30
−4	−7	0	21.20	10.00
−4	7	0	39.00	8.50
−4	7	0	46.20	12.80
4	7	0	21.20	10.10
−4	7	0	14.70	8.50
4	7	0	6.40	6.70
4	7	0	29.80	10.90
−4	7	0	17.90	5.00
4	7	0	9.10	4.70
−4	7	0	18.70	6.20

-5	-7	0	190.88	22.60
5	-7	0	184.58	14.10
-5	-7	0	229.88	23.10
-5	-7	0	170.28	15.70
5	-7	0	169.68	21.50
-5	7	0	182.18	16.80
-5	7	0	190.78	18.30
-5	7	0	176.08	26.90
5	7	0	167.88	13.10
-5	7	0	181.28	16.60
-6	-7	0	3.10	11.10
-6	-7	0	9.30	6.50
6	-7	0	3.70	5.40
-6	-7	0	11.00	12.60
6	7	0	0.90	5.80
-6	7	0	8.30	8.60
-6	7	0	7.20	7.90
-6	7	0	17.30	10.20
-6	7	0	-3.00	10.40
-7	-7	0	12.50	7.70
7	-7	0	8.60	6.50
-7	-7	0	11.90	13.20
-7	-7	0	7.70	11.30
-7	7	0	17.30	10.00
-7	7	0	-11.60	9.10
-8	-7	0	50.59	19.00
-8	-7	0	50.79	19.50
8	-7	0	19.40	8.20
-8	7	0	31.40	14.50
-8	7	0	9.80	17.80
8	7	0	14.20	9.70

-8	7	0	22.80	12.60
-9	-7	0	51.69	22.50
-9	-7	0	3.30	18.50
9	-7	0	14.50	9.10
-9	7	0	25.80	19.80
-9	7	0	10.40	14.90
9	7	0	-5.90	9.50
-9	7	0	45.10	17.80
-10	-7	0	22.00	19.00
-10	-7	0	42.90	22.70
-10	7	0	-22.30	16.10
10	7	0	-9.10	17.60
-10	7	0	24.90	38.20
-10	7	0	-0.90	17.20
0	-8	0	388.66	20.90
0	-8	0	386.56	29.60
0	-8	0	447.36	23.10
0	8	0	424.26	29.10
0	8	0	388.76	12.20
0	8	0	408.96	12.70
0	8	0	437.26	16.60
0	8	0	396.76	18.00
-1	-8	0	291.37	19.70
-1	-8	0	272.57	18.40
-1	-8	0	302.67	24.40
1	-8	0	276.27	16.90
1	-8	0	302.57	18.60
1	8	0	280.17	13.90
1	8	0	269.17	13.40
-1	8	0	233.68	23.40
-1	8	0	298.27	13.30

1	8	0	295.97	13.60
−1	8	0	285.37	12.50
1	8	0	251.27	21.50
−2	−8	0	86.29	12.30
−2	−8	0	80.79	12.80
−2	−8	0	93.09	7.30
−2	−8	0	88.29	15.40
−2	−8	0	92.09	13.60
2	8	0	87.49	9.60
2	8	0	76.09	10.10
−2	8	0	50.29	14.10
−2	8	0	72.39	7.50
−2	8	0	75.49	5.90
2	8	0	95.29	14.80
2	8	0	56.79	11.60
−3	−8	0	−5.20	3.50
−3	−8	0	3.80	5.20
−3	−8	0	−1.50	10.00
−3	−8	0	−4.70	6.00
−3	−8	0	5.50	7.70
−3	8	0	−2.20	4.60
−3	8	0	1.10	3.60
3	8	0	7.70	6.80
3	8	0	4.30	3.30
−3	8	0	4.20	8.70
−3	8	0	−4.50	4.20
3	8	0	−14.20	7.40
4	−8	0	128.79	15.60
−4	−8	0	91.09	16.80
−4	−8	0	89.69	13.40
−4	−8	0	137.89	21.20

4	8	0	119.29	17.00
-4	8	0	97.39	13.90
4	8	0	113.19	18.60
-4	8	0	102.19	20.70
4	8	0	94.39	11.80
-4	8	0	112.69	11.90
-4	8	0	98.59	11.00
-5	-8	0	11.90	10.30
5	-8	0	15.70	10.80
-5	-8	0	12.10	6.90
-5	-8	0	18.80	10.40
5	8	0	7.00	6.10
-5	8	0	7.90	9.10
-5	8	0	12.10	11.00
5	8	0	12.40	9.30
-5	8	0	8.50	7.20
-6	-8	0	89.49	20.10
-6	-8	0	92.79	14.80
-6	-8	0	149.09	25.40
-6	8	0	164.48	21.80
-6	8	0	138.19	20.50
6	8	0	102.89	13.60
-6	8	0	143.59	26.70
-7	-8	0	2.30	7.30
-7	-8	0	-2.90	14.70
-7	-8	0	20.80	14.20
7	-8	0	-4.10	11.80
-7	8	0	-18.20	10.10
-7	8	0	1.30	16.10
7	8	0	-2.20	6.30
-7	8	0	-4.30	10.90

-8	-8	0	24.60	17.30
-8	-8	0	8.00	15.50
8	8	0	-0.50	8.50
-8	8	0	-3.60	16.70
-8	8	0	10.80	13.30
-8	8	0	-15.10	13.40
-9	-8	0	26.40	20.80
-9	-8	0	31.30	20.10
9	-8	0	-3.30	14.50
-9	8	0	-37.10	14.40
-9	8	0	9.90	15.20
-9	8	0	-19.00	20.20
9	8	0	2.60	9.30
-10	-8	0	8.40	20.50
-10	-8	0	21.70	23.60
-10	8	0	-34.00	21.30
-10	8	0	-20.50	17.10
-10	8	0	13.30	19.80
10	8	0	6.60	10.30
0	-9	0	1.60	4.20
0	-9	0	-3.60	5.10
0	9	0	1.30	4.00
0	9	0	4.30	3.50
0	9	0	24.70	14.20
-1	-9	0	57.29	10.80
-1	-9	0	65.99	12.40
1	-9	0	60.99	11.00
1	-9	0	47.40	9.10
1	9	0	59.99	8.50
1	9	0	88.89	16.90
1	9	0	86.69	8.10

-1	9	0	65.59	7.60
-1	9	0	78.09	19.40
-2	-9	0	434.76	25.50
2	-9	0	449.16	25.00
-2	-9	0	452.95	16.90
-2	-9	0	453.35	28.10
2	-9	0	447.26	22.80
2	9	0	486.95	21.80
-2	9	0	443.26	17.60
2	9	0	447.16	28.80
2	9	0	418.46	28.20
-2	9	0	471.55	36.20
-3	-9	0	108.89	15.10
-3	-9	0	105.89	9.10
3	-9	0	97.29	14.30
-3	-9	0	114.09	18.80
-3	-9	0	111.99	16.90
-3	9	0	104.99	10.70
3	9	0	95.49	18.00
3	9	0	108.09	14.20
-3	9	0	61.09	19.10
3	9	0	131.49	11.30
-4	-9	0	30.90	12.10
-4	-9	0	16.00	7.70
4	-9	0	30.80	7.60
-4	-9	0	23.50	11.10
4	9	0	13.80	8.10
-4	9	0	8.30	7.70
-4	9	0	17.20	7.80
4	9	0	19.30	7.10
-4	9	0	15.80	7.00

−4	9	0	12.40	14.70
5	−9	0	3.80	6.80
−5	−9	0	9.80	5.90
−5	−9	0	12.90	13.40
−5	−9	0	13.10	12.50
5	−9	0	23.40	12.90
−5	9	0	0.20	7.40
−5	9	0	24.80	9.00
5	9	0	19.70	9.10
−5	9	0	−4.30	10.90
5	9	0	0.90	5.40
−6	−9	0	−1.40	6.90
−6	−9	0	−9.70	14.10
−6	−9	0	26.10	14.90
6	−9	0	8.90	7.60
−6	9	0	18.50	10.00
−6	9	0	16.60	14.50
−7	−9	0	−1.80	16.60
−7	−9	0	13.20	10.00
−7	−9	0	−8.30	14.30
7	−9	0	8.70	8.80
−7	9	0	8.80	11.60
−7	9	0	17.10	13.80
7	9	0	−3.00	7.00
−7	9	0	6.60	16.30
−8	−9	0	−15.30	19.00
8	−9	0	17.50	10.30
−8	−9	0	5.00	18.90
−8	9	0	6.30	13.80
8	9	0	4.70	10.40
−8	9	0	30.40	17.80

-9	-9	0	-21.00	22.30
9	-9	0	10.20	11.80
-9	-9	0	-37.70	19.10
-9	9	0	-13.90	16.60
-9	9	0	15.30	16.80
-9	9	0	-1.20	24.10
0	-10	0	86.39	13.90
0	10	0	97.39	18.90
0	10	0	97.39	10.50
1	-10	0	-4.60	6.30
1	-10	0	9.10	7.70
-1	-10	0	1.20	7.60
-1	-10	0	7.40	6.10
1	10	0	-5.90	3.60
-1	10	0	-8.70	9.50
1	10	0	-21.00	7.70
-1	10	0	3.30	4.70
1	10	0	5.80	4.10
-2	-10	0	275.37	28.00
2	-10	0	249.78	18.20
2	-10	0	253.17	19.80
-2	-10	0	268.47	21.40
2	10	0	283.27	15.90
-2	10	0	283.77	15.10
2	10	0	256.97	24.10
2	10	0	224.88	15.90
-2	10	0	272.27	14.80
-2	10	0	245.98	32.60
3	-10	0	12.70	8.10
-3	-10	0	24.30	10.70
-3	-10	0	17.80	8.90

-3	-10	0	18.30	5.90
-3	-10	0	19.10	11.20
3	10	0	28.20	6.70
3	10	0	11.00	13.10
-3	10	0	15.30	3.90
-3	10	0	7.40	13.40
3	10	0	-4.60	8.60
3	10	0	10.20	8.30
-4	-10	0	10.10	11.30
4	-10	0	-3.70	6.00
-4	-10	0	-4.70	6.70
-4	10	0	-6.80	5.50
-4	10	0	5.50	13.90
4	10	0	-1.30	5.70
4	10	0	6.70	8.60
-5	-10	0	-10.90	12.50
-5	-10	0	-0.20	12.40
5	-10	0	-11.10	6.80
-5	10	0	-9.40	12.40
-5	10	0	-11.90	6.70
-5	10	0	-6.20	8.00
5	10	0	7.70	9.70
5	10	0	10.20	7.20
-6	-10	0	302.77	37.30
-6	-10	0	242.18	33.30
6	-10	0	276.47	22.40
-6	-10	0	231.68	22.30
-6	10	0	227.48	23.80
-6	10	0	335.07	44.90
-6	10	0	234.68	21.60
6	10	0	225.28	27.20

6	10	0	290.77	21.80
-7	-10	0	28.00	11.30
7	-10	0	13.20	10.30
-7	-10	0	5.70	17.40
-7	-10	0	28.20	18.10
-7	10	0	27.40	13.00
7	10	0	10.10	9.10
-7	10	0	-0.50	12.70
-7	10	0	-6.80	16.40
-8	-10	0	7.30	22.50
-8	-10	0	14.50	23.60
8	-10	0	6.50	10.70
-8	10	0	7.70	14.60
-8	10	0	6.90	15.10
-8	10	0	0.60	20.60
8	10	0	8.00	10.90
-9	-10	0	6.20	23.70
-9	-10	0	6.10	25.10
-9	10	0	7.80	21.40
-9	10	0	23.70	18.00
-9	10	0	18.30	18.30
9	10	0	-9.90	10.50
0	-11	0	5.60	8.70
0	-11	0	2.10	7.50
0	11	0	12.30	5.40
0	11	0	-6.90	10.30
-1	-11	0	14.00	9.70
-1	-11	0	17.50	8.40
1	-11	0	4.00	8.30
1	-11	0	-7.20	5.80
1	11	0	-2.30	4.90

-1	11	0	-0.10	4.90
1	11	0	-0.70	11.00
-1	11	0	-6.20	12.50
-2	-11	0	470.15	29.30
2	-11	0	488.65	28.40
2	-11	0	434.96	25.80
-2	11	0	493.35	42.30
2	11	0	529.95	23.40
2	11	0	450.45	21.30
2	11	0	463.85	34.40
-2	11	0	448.96	21.00
3	-11	0	39.70	10.50
-3	-11	0	44.40	15.20
3	-11	0	5.00	7.10
3	11	0	50.29	16.60
-3	11	0	44.30	18.80
3	11	0	28.90	8.50
3	11	0	63.99	12.10
-4	-11	0	61.19	14.00
4	-11	0	52.09	13.30
-4	-11	0	69.59	19.60
-4	-11	0	13.90	12.10
-4	11	0	70.79	23.70
4	11	0	40.80	13.50
4	11	0	35.50	9.80
-5	-11	0	75.09	18.30
-5	-11	0	51.29	16.10
-5	-11	0	67.69	15.40
5	-11	0	35.90	11.30
5	11	0	30.60	9.00
-5	11	0	38.10	19.80

-5	11	0	57.29	11.50
5	11	0	31.10	13.50
6	-11	0	-4.90	10.10
-6	-11	0	-15.50	17.00
-6	-11	0	1.30	10.30
-6	-11	0	-1.30	17.60
-6	11	0	6.20	18.40
-6	11	0	14.00	9.70
-6	11	0	-22.50	8.60
6	11	0	19.90	13.90
6	11	0	-8.00	8.10
7	-11	0	-21.90	9.90
-7	-11	0	6.50	10.50
-7	-11	0	5.60	19.90
7	11	0	-15.80	7.90
-7	11	0	-20.40	18.60
-7	11	0	-14.50	10.50
-7	11	0	8.40	12.80
-8	-11	0	15.00	24.10
8	-11	0	-4.20	13.50
-8	-11	0	25.60	23.20
-8	11	0	-6.50	15.60
-8	11	0	-38.20	34.60
-8	11	0	1.80	20.00
0	-12	0	4.30	8.90
0	12	0	1.50	14.50
0	12	0	5.70	6.50
1	-12	0	86.29	16.40
1	-12	0	94.59	14.30
-1	-12	0	90.39	17.30
-1	-12	0	80.79	16.00

1	12	0	123.09	23.20
1	12	0	91.49	11.90
-1	12	0	89.29	12.80
-1	12	0	105.09	23.10
2	-12	0	1.10	8.70
-2	-12	0	29.50	14.20
2	-12	0	27.50	9.80
-2	-12	0	30.10	12.00
2	12	0	48.80	8.40
2	12	0	42.90	15.50
-2	12	0	38.10	18.60
-2	12	0	22.40	8.00
2	12	0	21.30	7.80
-3	-12	0	8.00	9.90
3	-12	0	4.30	9.20
-3	-12	0	-2.10	13.60
3	-12	0	-6.40	17.70
-3	12	0	-0.80	17.10
-3	12	0	0.60	5.80
3	12	0	-3.60	6.70
3	12	0	20.30	12.70
4	-12	0	12.00	10.20
-4	-12	0	0.50	16.50
-4	-12	0	14.40	10.70
-4	12	0	32.50	16.30
4	12	0	13.20	11.40
-5	-12	0	11.00	10.70
5	-12	0	35.70	12.80
-5	-12	0	25.50	18.70
-5	-12	0	5.70	16.30
5	12	0	4.30	8.10

-5	12	0	33.40	20.30
-5	12	0	11.20	9.30
5	12	0	23.50	14.00
-6	-12	0	89.59	21.90
6	-12	0	31.30	14.00
-6	-12	0	18.30	13.50
-6	-12	0	31.70	22.50
6	12	0	7.00	15.30
-6	12	0	-1.70	23.40
6	12	0	32.40	11.80
-6	12	0	34.30	12.10
-7	-12	0	-18.50	22.10
7	-12	0	23.50	13.40
-7	-12	0	38.10	26.50
-7	-12	0	9.70	12.70
-7	12	0	16.00	25.10
7	12	0	-0.20	11.20
0	-13	0	1.90	12.00
0	-13	0	8.10	9.70
0	13	0	21.20	16.70
0	13	0	17.10	7.60
-1	-13	0	-2.20	8.70
1	-13	0	-6.20	11.20
-1	-13	0	-23.60	12.60
1	-13	0	13.70	10.60
1	13	0	20.30	17.60
1	13	0	0.30	6.90
-1	13	0	13.30	7.70
-1	13	0	15.20	17.10
2	-13	0	43.20	12.30
2	-13	0	0.40	10.60

-2	-13	0	44.00	18.60
-2	-13	0	41.50	13.90
-2	13	0	43.50	10.20
2	13	0	50.19	10.90
2	13	0	33.20	15.20
-3	-13	0	-4.80	8.90
-3	-13	0	-6.00	15.70
3	-13	0	4.40	10.00
3	13	0	-8.20	7.10
-3	13	0	16.20	8.30
3	13	0	14.10	14.40
-3	13	0	16.50	20.60
-4	-13	0	-1.10	17.60
4	-13	0	4.70	10.50
-4	-13	0	-1.60	10.00
-4	13	0	-24.70	17.40
4	13	0	23.90	14.80
-4	13	0	-2.90	7.90
4	13	0	-5.20	8.00
5	-13	0	3.30	10.70
-5	-13	0	-2.40	21.80
-5	-13	0	8.40	12.30
-5	13	0	10.40	22.50
5	13	0	-10.20	8.40
5	13	0	33.70	17.70
-6	-13	0	-16.40	13.70
6	-13	0	27.50	15.00
-6	-13	0	-34.30	20.60
-6	-13	0	15.00	23.70
6	13	0	-16.80	16.90
6	13	0	7.60	11.30

-6	13	0	24.10	21.10
0	-14	0	13.00	12.30
0	14	0	18.80	9.00
-1	-14	0	-16.60	9.70
-1	-14	0	-16.80	15.90
1	-14	0	1.50	9.00
1	14	0	-3.10	7.50
1	14	0	13.30	16.60
-1	14	0	-19.10	17.60
-1	14	0	3.80	7.90
-2	-14	0	38.80	14.20
-2	-14	0	-8.50	17.90
2	-14	0	41.20	14.80
2	14	0	15.00	9.90
-2	14	0	30.70	10.40
2	14	0	50.39	20.00
-2	14	0	68.39	22.90
-3	-14	0	-13.00	9.90
3	-14	0	13.70	11.70
3	14	0	36.10	17.10
3	14	0	9.00	9.00
-3	14	0	-15.30	22.00
-3	14	0	15.60	8.30
-4	-14	0	-21.50	10.10
4	-14	0	-7.80	12.70
-4	-14	0	-22.10	24.30
-4	14	0	-5.30	9.60
4	14	0	33.00	20.80
4	14	0	-8.50	8.90
-4	14	0	-6.10	22.10
-5	-14	0	8.40	12.90

5	-14	0	-16.70	13.80
-5	-14	0	25.40	26.60
5	14	0	2.40	10.70
5	14	0	-2.50	17.10
-5	14	0	32.00	26.70
-5	14	0	23.40	11.70
0	-15	0	-8.00	12.30
0	15	0	17.40	8.90
0	15	0	9.60	20.30
1	-15	0	5.10	11.50
-1	-15	0	3.20	12.30
-1	15	0	49.10	23.20
1	15	0	-24.00	20.80
-1	15	0	-8.70	9.50
2	-15	0	52.29	16.80
-2	15	0	24.70	22.40
2	15	0	26.80	11.40
2	15	0	19.80	19.60
-2	15	0	44.60	12.50
3	-15	0	18.80	13.70
-3	-15	0	9.50	14.00
3	15	0	16.80	11.10
-3	15	0	13.10	11.20
3	15	0	16.60	18.90
-3	15	0	18.70	24.30
-4	-15	0	1.70	14.90
4	-15	0	41.60	18.50
-4	15	0	11.70	14.00
4	15	0	9.20	18.60
-4	15	0	-33.00	23.40
4	15	0	1.10	11.50

0	16	0	27.70	23.20
11	0	−1	43.20	14.10
−11	0	1	14.40	19.20
−11	0	1	2.30	18.40
−11	0	1	50.09	22.70
11	0	−1	13.20	10.10
10	0	−1	10.40	7.90
−10	0	1	−2.80	13.60
10	0	−1	−16.00	8.60
−10	0	1	3.20	14.30
9	0	−1	62.39	10.80
−9	0	1	102.69	22.80
−9	0	1	63.39	18.00
−8	0	1	3.40	9.20
−8	0	1	0.10	9.20
8	0	−1	−10.10	8.20
8	0	−1	5.00	6.10
−7	0	1	0.50	7.50
7	0	−1	10.50	5.10
−7	0	1	2.30	7.80
7	0	−1	5.50	5.20
6	0	−1	6.40	3.90
6	0	−1	−0.30	4.00
−6	0	1	2.10	6.80
−6	0	1	−1.20	7.10
5	0	−1	195.18	11.20
−5	0	1	167.78	16.50
−5	0	1	153.88	16.00
4	0	−1	−2.00	4.80
4	0	−1	−1.80	2.10
−4	0	1	1.70	4.60

−4	0	1	−2.80	3.50
−3	0	1	2557.84	69.49
−3	0	1	2795.12	72.29
3	0	−1	2593.94	71.39
3	0	−1	2622.14	69.59
−3	0	1	2663.83	61.59
3	0	−1	2700.33	61.59
2	0	−1	−1.20	2.00
2	0	−1	0.80	1.20
−2	0	1	1.10	2.20
−2	0	1	1.50	2.10
−2	0	1	2.20	1.50
2	0	−1	3.40	2.50
−1	0	1	2685.23	60.49
1	0	−1	2762.42	61.89
−1	0	1	2708.13	62.99
−1	0	1	2566.74	57.69
−1	0	1	2751.52	61.89
1	0	−1	2616.24	62.59
1	0	−1	2675.53	57.89
−1	0	−1	418.66	14.40
1	0	1	404.86	12.80
−1	0	−1	406.66	10.50
1	0	1	417.26	14.30
1	0	1	410.16	10.50
−1	0	−1	414.16	10.70
−1	0	−1	406.76	12.80
−1	0	−1	431.76	13.90
−1	0	−1	403.36	9.40
−2	0	−1	2.70	2.30
2	0	1	0.60	0.70

2	0	1	5.70	2.80
−2	0	−1	−0.20	0.90
−2	0	−1	0.10	1.80
2	0	1	−1.00	1.20
2	0	1	3.80	2.60
2	0	1	−0.60	1.00
−3	0	−1	2946.81	67.19
3	0	1	2976.20	74.59
−3	0	−1	2973.90	74.69
−3	0	−1	2957.80	77.09
3	0	1	2980.30	67.09
3	0	1	2981.80	77.89
3	0	1	2994.80	64.69
4	0	1	−1.80	3.90
−4	0	−1	0.40	2.30
−4	0	−1	−1.70	3.70
−4	0	−1	−1.90	3.00
4	0	1	1.00	1.80
4	0	1	−3.10	5.40
5	0	1	1586.24	39.60
−5	0	−1	1585.34	56.69
5	0	1	1550.14	42.10
−5	0	−1	1575.74	43.20
−5	0	−1	1640.64	53.69
−6	0	−1	3.00	7.30
6	0	1	3.00	4.00
6	0	1	−0.80	3.30
−6	0	−1	−3.90	6.60
7	0	1	31.70	5.50
−7	0	−1	15.10	9.70
−7	0	−1	19.50	10.00

7	0	1	18.40	5.20
-8	0	-1	9.50	9.90
8	0	1	-14.00	5.20
8	0	1	1.90	5.00
-8	0	-1	-12.20	8.80
-9	0	-1	60.89	18.40
9	0	1	108.89	11.60
-9	0	-1	69.39	19.60
9	0	1	114.59	13.60
10	0	1	10.40	7.60
-10	0	-1	-11.10	12.80
-10	0	-1	-4.80	14.30
10	0	1	7.60	10.70
11	0	1	2.00	9.40
-11	0	-1	52.29	20.30
-11	0	-1	36.70	21.20
11	-1	-1	16.90	13.30
-11	-1	1	-12.60	16.20
11	-1	-1	-9.10	9.90
-11	-1	1	-9.90	15.50
-11	1	1	19.80	21.10
-11	1	1	-4.50	17.80
11	1	-1	3.00	10.20
11	1	-1	-9.90	12.40
-11	1	1	3.00	17.60
-10	-1	1	18.20	15.70
-10	-1	1	-7.40	14.40
10	-1	-1	-4.80	7.40
-10	1	1	-0.30	13.50
-10	1	1	-0.50	15.30
-10	1	1	6.00	16.50

10	1	-1	5.40	8.10
-9	-1	1	11.30	14.50
-9	-1	1	12.80	13.10
9	-1	-1	7.80	6.40
9	-1	-1	17.80	8.90
9	1	-1	4.10	6.30
-9	1	1	13.70	12.90
-9	1	1	4.90	11.10
8	-1	-1	2.50	4.40
-8	-1	1	-6.20	8.70
8	-1	-1	-1.20	6.60
8	-1	-1	-10.80	6.00
-8	-1	1	-2.90	9.60
8	1	-1	13.90	6.70
-8	1	1	-5.40	8.50
-8	1	1	15.00	11.50
-7	-1	1	127.49	19.60
7	-1	-1	128.29	11.40
7	-1	-1	146.99	11.70
7	-1	-1	130.89	12.80
-7	-1	1	110.59	19.80
-7	1	1	137.09	22.20
-7	1	1	170.58	22.70
7	1	-1	121.19	12.00
7	1	-1	126.19	11.20
6	-1	-1	71.89	10.00
-6	-1	1	76.29	15.40
-6	-1	1	51.29	13.10
6	1	-1	65.79	8.70
6	1	-1	57.19	7.80
-6	1	1	51.19	12.20

-6	1	1	56.49	11.90
5	-1	-1	360.76	15.60
-5	-1	1	352.26	22.90
-5	-1	1	350.76	24.20
5	1	-1	368.26	16.40
5	1	-1	349.67	15.90
-5	1	1	365.96	23.10
-5	1	1	335.27	23.50
-5	1	1	371.26	24.10
4	-1	-1	255.07	17.30
-4	-1	1	273.07	12.60
-4	-1	1	269.77	17.90
4	-1	-1	261.77	21.70
-4	-1	1	269.47	19.30
4	1	-1	275.97	13.00
4	1	-1	272.37	19.40
4	1	-1	265.97	12.30
-4	1	1	273.57	18.10
-4	1	1	274.77	14.10
-4	1	1	248.28	17.00
3	-1	-1	283.97	15.80
3	-1	-1	279.67	10.90
-3	-1	1	260.17	15.40
-3	-1	1	244.68	10.70
3	-1	-1	257.07	15.80
-3	-1	1	252.27	16.10
3	1	-1	273.07	16.70
3	1	-1	253.27	10.80
-3	1	1	274.77	11.00
-3	1	1	226.18	14.40
-3	1	1	260.17	15.40

3	1	−1	280.77	16.00
3	1	−1	270.87	11.60
−2	−1	1	64.49	4.30
−2	−1	1	59.49	7.50
2	−1	−1	58.79	6.30
2	−1	−1	58.09	4.60
−2	−1	1	58.79	7.90
2	−1	−1	57.59	4.90
2	−1	−1	58.99	7.70
−2	1	1	62.19	6.40
2	1	−1	59.29	4.50
2	1	−1	60.59	4.20
2	1	−1	51.19	7.20
2	1	−1	69.89	8.20
−2	1	1	64.69	4.40
−2	1	1	57.09	7.20
1	−1	−1	1217.48	32.00
−1	−1	1	1229.38	34.50
−1	−1	1	1282.07	32.50
1	−1	−1	1218.98	31.80
1	−1	−1	1279.57	35.80
−1	−1	1	1270.97	28.70
1	−1	−1	1250.97	29.50
−1	1	1	1234.78	31.60
1	1	−1	1267.27	31.80
1	1	−1	1337.17	32.70
−1	1	1	1266.67	32.10
−1	1	1	1325.67	35.60
1	1	−1	1277.37	28.80
−1	1	1	1263.17	30.80
−1	1	1	1245.18	33.30

0	-1	-1	483.25	13.60
0	-1	-1	500.25	12.40
0	1	1	475.35	12.30
0	1	1	431.86	12.10
0	1	1	456.05	15.30
0	1	-1	472.15	12.30
0	1	-1	488.45	11.80
0	1	1	493.85	15.20
-1	-1	-1	1097.49	26.00
1	-1	1	1076.29	25.70
-1	-1	-1	1107.89	25.90
-1	-1	-1	1116.59	31.00
-1	-1	-1	1148.89	29.40
1	-1	1	1098.09	29.50
-1	-1	-1	1115.59	29.60
-1	-1	-1	1071.99	25.60
-1	1	-1	1104.79	24.00
-1	1	-1	1083.99	23.70
1	1	1	1075.39	25.60
-1	1	-1	1120.79	29.60
-1	1	-1	1085.69	23.70
1	1	1	1113.79	29.40
1	1	1	1084.29	29.10
1	1	1	1110.49	26.10
-1	1	-1	1113.69	25.70
1	1	1	1099.49	26.80
2	-1	1	679.63	17.10
2	-1	1	694.13	21.30
-2	-1	-1	700.93	23.40
-2	-1	-1	655.53	23.10
2	-1	1	688.93	17.10

−2	−1	−1	684.23	18.20
2	−1	1	718.13	24.50
−2	−1	−1	695.33	24.10
−2	−1	−1	652.33	18.40
2	1	1	669.03	19.50
−2	1	−1	684.63	23.80
2	1	1	677.83	18.60
2	1	1	701.23	18.90
−2	1	−1	730.43	21.50
−2	1	−1	679.73	17.50
2	1	1	698.63	24.20
2	1	1	684.53	23.40
2	1	1	698.33	23.60
3	−1	1	10.80	3.60
3	−1	1	8.00	2.30
−3	−1	−1	11.10	4.30
−3	−1	−1	12.10	4.00
3	−1	1	10.50	2.10
−3	−1	−1	11.10	4.20
3	−1	1	7.70	3.60
−3	−1	−1	8.70	2.20
3	−1	1	10.10	2.30
−3	−1	−1	8.50	2.30
3	1	1	15.20	5.70
3	1	1	12.10	4.20
−3	1	−1	9.70	3.70
3	1	1	9.80	2.90
3	1	1	12.00	2.50
−3	1	−1	12.30	5.00
−3	1	−1	10.70	2.40
4	−1	1	8.60	2.50

4	−1	1	8.20	2.60
−4	−1	−1	3.70	4.50
−4	−1	−1	5.90	4.80
4	−1	1	9.70	6.30
4	−1	1	14.60	6.00
−4	−1	−1	6.20	3.00
−4	1	−1	9.40	4.60
4	1	1	9.30	2.60
4	1	1	11.00	5.80
4	1	1	3.30	5.30
−4	1	−1	10.00	3.30
4	1	1	9.10	3.30
−4	1	−1	11.50	5.30
5	−1	1	413.76	13.80
−5	−1	−1	389.46	25.20
−5	−1	−1	359.56	27.80
5	−1	1	397.36	15.10
−5	−1	−1	406.36	24.10
5	−1	1	402.16	16.00
5	1	1	429.06	17.20
−5	1	−1	403.06	16.60
−5	1	−1	415.46	23.20
5	1	1	421.06	15.80
−5	1	−1	449.46	26.10
−6	−1	−1	68.99	14.20
−6	−1	−1	86.09	15.20
6	−1	1	77.59	8.20
6	−1	1	72.99	6.50
6	−1	1	70.99	8.80
6	−1	1	83.99	8.80
−6	1	−1	65.49	12.40

−6	1	−1	89.29	16.10
6	1	1	83.39	9.50
6	1	1	71.19	7.70
−7	−1	−1	121.69	19.80
7	−1	1	125.99	10.60
7	−1	1	134.59	9.20
7	−1	1	143.29	10.20
−7	−1	−1	93.99	17.80
7	1	1	148.49	12.10
−7	1	−1	137.39	20.10
7	1	1	123.39	10.60
−7	1	−1	139.19	20.50
8	−1	1	34.60	8.10
8	−1	1	26.70	6.70
−8	−1	−1	41.10	14.20
−8	−1	−1	39.10	14.30
8	−1	1	48.20	7.10
−8	1	−1	33.20	12.80
8	1	1	54.19	12.70
−8	1	−1	61.69	16.70
8	1	1	45.70	9.60
−9	−1	−1	17.90	14.10
9	−1	1	19.00	8.00
−9	−1	−1	7.40	13.20
9	−1	1	24.20	7.40
9	−1	1	15.50	6.70
−9	1	−1	21.90	11.90
9	1	1	22.70	8.80
−9	1	−1	10.20	12.50
9	1	1	3.50	7.80
10	−1	1	−3.70	7.60

10	−1	1	2.40	7.90
−10	−1	−1	−0.40	14.20
−10	−1	−1	5.40	14.80
−10	1	−1	−7.90	14.40
−10	1	−1	8.70	15.00
10	1	1	4.10	8.70
−11	−1	−1	−1.70	20.10
−11	−1	−1	−28.10	15.90
11	−1	1	8.70	9.00
−11	1	−1	21.50	19.50
11	1	1	0.50	10.10
−11	1	−1	−7.70	16.80
−11	−2	1	−7.80	19.10
−11	−2	1	−6.60	19.20
11	−2	−1	15.40	10.20
−11	2	1	30.20	18.90
−11	2	1	7.40	18.60
−11	2	1	14.70	16.50
11	2	−1	34.40	12.40
10	−2	−1	−18.30	9.90
−10	−2	1	5.20	14.00
10	−2	−1	2.40	8.50
−10	−2	1	21.80	17.10
10	2	−1	3.20	7.50
−10	2	1	8.50	15.20
−10	2	1	11.20	15.10
−10	2	1	−20.60	14.80
9	−2	−1	7.90	8.30
−9	−2	1	−5.50	12.00
−9	−2	1	−16.70	11.10
9	−2	−1	7.70	5.80

−9	2	1	1.50	13.20
−9	2	1	−12.90	10.20
9	2	−1	−3.60	6.10
−9	2	1	−4.70	10.40
8	−2	−1	11.50	7.80
8	−2	−1	17.50	7.40
−8	−2	1	6.50	11.90
8	−2	−1	18.20	7.00
−8	−2	1	17.00	11.70
−8	2	1	14.20	10.80
−8	2	1	7.90	10.60
−8	2	1	3.60	10.50
8	2	−1	21.90	8.20
7	−2	−1	68.89	9.60
7	−2	−1	72.69	11.90
−7	−2	1	75.19	16.80
−7	−2	1	72.39	16.50
−7	2	1	92.29	18.80
7	2	−1	105.29	11.80
−7	2	1	82.39	17.00
−7	2	1	86.59	17.40
6	−2	−1	34.90	6.70
−6	−2	1	22.50	9.60
6	−2	−1	31.50	8.20
−6	−2	1	33.50	11.10
6	2	−1	50.69	7.80
−6	2	1	30.20	9.90
−6	2	1	47.10	12.30
6	2	−1	42.00	7.70
−5	−2	1	7.30	6.30
5	−2	−1	2.80	3.40

5	−2	−1	6.30	3.50
5	−2	−1	8.80	7.10
−5	−2	1	7.10	5.90
−5	−2	1	11.00	6.30
5	2	−1	4.40	3.60
−5	2	1	17.90	7.20
−5	2	1	8.20	4.00
−5	2	1	3.80	5.50
−5	2	1	3.80	6.40
−4	−2	1	10.10	4.90
4	−2	−1	4.40	4.90
4	−2	−1	5.20	2.60
−4	−2	1	10.50	5.60
−4	−2	1	−1.50	3.60
4	−2	−1	6.60	4.70
4	−2	−1	3.90	3.20
−4	−2	1	0.90	2.90
4	2	−1	3.90	2.60
−4	2	1	1.80	4.40
4	2	−1	−1.40	2.90
4	2	−1	7.30	6.40
−4	2	1	0.70	3.00
−4	2	1	4.70	5.60
−4	2	1	5.00	4.60
3	−2	−1	13.40	4.70
−3	−2	1	22.80	6.00
3	−2	−1	11.40	3.50
3	−2	−1	10.10	2.60
−3	−2	1	20.50	6.30
−3	−2	1	14.50	4.00
−3	−2	1	16.60	3.30

3	−2	−1	16.90	5.20
−3	2	1	13.70	4.90
−3	2	1	18.20	3.60
3	2	−1	12.40	3.10
−3	2	1	24.80	6.90
−3	2	1	11.90	4.70
3	2	−1	18.20	5.50
3	2	−1	18.30	3.90
3	2	−1	20.10	6.40
−2	−2	1	275.67	11.60
−2	−2	1	259.17	14.90
−2	−2	1	295.77	16.00
−2	−2	1	277.97	11.60
2	−2	−1	288.37	16.30
−2	−2	1	259.57	9.70
2	−2	−1	291.57	13.50
2	−2	−1	278.27	14.30
−2	2	1	265.77	12.20
−2	2	1	276.57	14.20
2	2	−1	297.27	11.90
−2	2	1	300.77	10.70
2	2	−1	274.87	15.20
−2	2	1	287.27	16.20
−2	2	1	288.97	13.20
2	2	−1	284.07	10.30
2	2	−1	248.08	13.50
1	−2	−1	92.59	7.60
1	−2	−1	93.19	9.20
1	−2	−1	99.89	5.00
1	−2	−1	102.19	5.40
1	−2	−1	100.59	6.40

-1	-2	1	98.79	6.50
-1	-2	1	109.69	8.00
-1	-2	1	101.89	5.20
-1	2	1	113.59	5.80
1	2	-1	105.29	6.00
1	2	-1	113.89	8.00
1	2	-1	118.19	7.00
1	2	-1	104.89	4.50
-1	2	1	109.19	6.70
1	2	-1	105.69	5.20
-1	2	1	112.99	5.40
-1	2	1	102.99	7.80
0	-2	1	368.46	11.70
0	-2	-1	368.96	16.10
0	-2	-1	343.87	13.50
0	-2	-1	368.26	10.50
0	-2	1	336.07	12.30
0	-2	-1	382.26	12.70
0	2	1	359.66	15.70
0	2	-1	368.86	10.10
0	2	1	367.06	12.50
0	2	1	374.26	10.90
0	2	1	367.26	14.20
0	2	-1	357.96	8.20
0	2	1	349.07	11.20
0	2	-1	363.46	13.10
0	2	-1	365.76	11.70
-1	-2	-1	205.08	8.90
1	-2	1	236.38	8.10
-1	-2	-1	220.38	11.00
1	-2	1	226.88	6.90

−1	−2	−1	217.98	11.10
1	−2	1	231.88	11.90
−1	−2	−1	242.28	10.00
−1	−2	−1	231.28	8.00
−1	−2	−1	218.68	9.20
1	2	1	236.78	11.30
1	2	1	226.08	8.20
−1	2	−1	230.68	10.20
−1	2	−1	245.38	8.10
1	2	1	244.38	11.50
1	2	1	233.78	8.30
−1	2	−1	236.78	11.70
−1	2	−1	241.38	7.00
1	2	1	228.78	10.00
1	2	1	225.98	9.20
2	−2	1	27.40	2.50
−2	−2	−1	25.50	3.50
−2	−2	−1	27.20	4.00
2	−2	1	22.40	5.00
2	−2	1	25.80	1.90
−2	−2	−1	24.30	5.40
2	−2	1	39.60	4.90
−2	−2	−1	26.30	5.00
−2	−2	−1	23.30	5.00
2	−2	1	23.70	1.60
2	2	1	37.20	6.40
2	2	1	29.80	3.90
2	2	1	22.40	3.30
2	2	1	25.10	4.80
−2	2	−1	25.20	3.90
2	2	1	27.30	5.30

-2	2	-1	23.40	5.10
2	2	1	25.40	3.50
-2	2	-1	28.80	2.90
-3	-2	-1	271.67	11.00
3	-2	1	265.27	9.20
3	-2	1	247.58	12.30
3	-2	1	242.18	12.90
-3	-2	-1	242.18	14.80
-3	-2	-1	246.48	11.00
-3	-2	-1	232.28	15.10
3	-2	1	255.47	8.70
3	-2	1	261.67	15.70
-3	-2	-1	273.97	16.20
3	2	1	256.87	11.10
3	2	1	253.07	15.40
-3	2	-1	259.57	13.50
-3	2	-1	241.78	15.00
3	2	1	267.97	16.10
-3	2	-1	270.57	9.90
-4	-2	-1	46.10	9.40
-4	-2	-1	55.99	6.70
4	-2	1	54.29	4.80
-4	-2	-1	32.30	7.90
4	-2	1	45.70	4.50
4	-2	1	48.00	8.50
4	-2	1	48.60	5.10
-4	-2	-1	49.00	6.00
4	2	1	40.90	9.00
4	2	1	37.10	5.60
-4	2	-1	43.20	5.30
4	2	1	47.00	9.50

4	2	1	47.70	6.10
−4	2	−1	59.69	10.60
−4	2	−1	34.70	6.70
5	−2	1	15.50	3.80
5	−2	1	18.70	3.90
−5	−2	−1	13.50	6.90
−5	−2	−1	6.80	5.70
−5	2	−1	18.70	7.90
5	2	1	17.30	4.80
−5	2	−1	18.30	4.90
5	2	1	14.10	3.90
−5	2	−1	12.50	5.70
6	−2	1	51.19	7.00
6	−2	1	46.70	5.10
−6	−2	−1	43.50	12.10
−6	−2	−1	80.19	16.00
6	−2	1	33.90	6.30
6	−2	1	44.60	6.40
−6	2	−1	44.50	9.80
−6	2	−1	56.09	12.60
6	2	1	48.30	7.20
6	2	1	46.20	7.40
7	−2	1	41.00	5.90
−7	−2	−1	39.30	13.50
7	−2	1	40.40	6.90
−7	−2	−1	36.10	13.20
7	2	1	42.70	7.70
−7	2	−1	44.50	12.50
−7	2	−1	28.30	10.60
7	2	1	54.19	7.60
8	−2	1	5.60	4.30

8	−2	1	2.40	4.80
−8	−2	−1	1.50	8.70
8	−2	1	11.20	5.20
−8	−2	−1	−2.40	9.50
−8	2	−1	9.40	9.70
8	2	1	−13.30	6.50
−8	2	−1	7.20	9.80
8	2	1	0.70	5.70
9	−2	1	59.99	10.30
9	−2	1	31.40	6.90
9	−2	1	41.60	7.80
−9	−2	−1	38.40	15.90
−9	−2	−1	49.70	17.10
−9	2	−1	31.40	15.10
−9	2	−1	40.50	16.00
10	−2	1	8.50	7.80
−10	−2	−1	−14.00	13.00
10	−2	1	−0.10	6.30
−10	−2	−1	28.00	17.80
−10	2	−1	2.10	16.80
−10	2	−1	−2.20	14.30
10	2	1	−1.40	8.10
11	−2	1	8.20	10.10
−11	−2	−1	6.30	23.20
−11	−2	−1	8.30	22.40
−11	2	−1	11.50	16.20
−11	2	−1	3.60	19.90
−11	2	−1	18.40	18.00
11	2	1	−4.40	9.70
−11	−3	1	−7.00	22.50
11	−3	−1	−8.10	10.20

-11	3	1	14.70	19.40
-11	3	1	8.30	23.00
-11	3	1	17.40	18.90
11	3	-1	4.20	9.80
-10	-3	1	1.70	16.70
-10	-3	1	-4.60	14.50
10	-3	-1	4.50	7.90
10	-3	-1	-1.30	10.90
-10	3	1	6.30	15.00
-10	3	1	8.50	17.50
-10	3	1	-1.40	14.20
10	3	-1	2.30	9.20
-9	-3	1	20.90	14.70
9	-3	-1	21.10	9.60
-9	-3	1	-26.50	13.50
9	-3	-1	-7.80	10.00
9	-3	-1	9.70	6.70
-9	3	1	5.10	11.40
9	3	-1	7.70	6.60
-9	3	1	-11.20	11.70
-9	3	1	32.90	16.30
8	-3	-1	24.90	8.50
-8	-3	1	-6.30	10.80
8	-3	-1	12.30	6.70
-8	-3	1	25.60	11.80
-8	3	1	7.10	9.10
-8	3	1	9.90	11.40
-8	3	1	26.40	12.70
8	3	-1	10.40	7.00
7	-3	-1	53.19	10.10
7	-3	-1	56.49	8.80

−7	−3	1	62.59	16.30
−7	−3	1	56.79	15.80
−7	3	1	29.70	10.10
−7	3	1	54.59	14.70
−7	3	1	27.30	12.40
7	3	−1	64.09	9.70
−6	−3	1	15.60	8.80
−6	−3	1	39.20	12.80
6	−3	−1	22.40	5.60
−6	3	1	28.50	8.90
6	3	−1	20.30	6.40
−6	3	1	17.40	9.10
−6	3	1	14.90	7.90
−5	−3	1	194.18	15.00
5	−3	−1	174.08	18.30
−5	−3	1	175.28	17.70
5	−3	−1	155.48	12.40
5	−3	−1	151.18	9.90
−5	−3	1	198.68	19.00
−5	3	1	153.58	18.00
−5	3	1	144.79	15.00
5	3	−1	168.18	12.50
−5	3	1	156.78	11.00
5	3	−1	150.08	12.20
4	−3	−1	−4.10	2.10
−4	−3	1	5.20	3.00
4	−3	−1	1.90	5.60
−4	−3	1	4.60	4.60
−4	−3	1	2.90	4.10
4	−3	−1	5.50	5.10
4	−3	−1	5.20	3.60

-4	-3	1	6.20	5.00
4	3	-1	2.90	2.90
4	3	-1	11.40	6.10
-4	3	1	6.90	4.70
-4	3	1	-1.30	3.10
-4	3	1	3.30	4.50
4	3	-1	6.30	5.90
3	-3	-1	158.08	10.60
3	-3	-1	177.38	8.50
-3	-3	1	158.98	8.40
-3	-3	1	161.18	14.00
-3	-3	1	159.28	9.90
-3	-3	1	157.68	10.60
3	-3	-1	158.78	12.90
3	-3	-1	176.58	12.80
-3	3	1	161.98	15.40
3	3	-1	184.58	13.90
-3	3	1	171.18	11.80
3	3	-1	168.98	10.20
-3	3	1	162.68	8.90
-3	3	1	163.88	10.80
3	3	-1	170.88	13.70
-3	3	1	147.29	13.60
-2	-3	1	324.47	16.10
-2	-3	1	338.57	13.90
-2	-3	1	338.17	18.00
2	-3	-1	337.67	14.30
2	-3	-1	360.66	17.20
-2	-3	1	318.37	13.80
-2	-3	1	333.47	11.60
2	-3	-1	348.27	20.40

2	-3	-1	341.57	14.80
-2	3	1	354.56	12.80
2	3	-1	349.07	14.00
-2	3	1	311.47	16.70
-2	3	1	338.27	20.40
2	3	-1	342.37	12.20
-2	3	1	329.77	13.90
2	3	-1	380.56	17.10
2	3	-1	339.97	17.30
-2	3	1	347.87	15.30
2	3	-1	345.27	14.20
-1	-3	1	498.95	20.00
1	-3	-1	503.35	14.10
-1	-3	1	494.85	19.90
-1	-3	1	516.65	17.60
1	-3	-1	504.95	17.90
1	-3	-1	497.55	23.80
-1	-3	1	530.85	18.40
1	-3	-1	511.45	20.20
-1	-3	1	481.65	13.60
1	-3	-1	499.65	14.10
-1	3	1	521.65	24.50
-1	3	1	530.25	18.10
-1	3	1	490.75	19.80
1	3	-1	508.85	14.20
1	3	-1	533.75	17.70
1	3	-1	547.05	18.30
1	3	-1	496.65	19.30
-1	3	1	530.35	15.00
1	3	-1	520.75	16.80
-1	3	1	508.15	14.40

0	-3	-1	969.90	23.70
0	-3	1	980.80	26.80
0	-3	-1	957.70	27.60
0	-3	-1	938.31	33.40
0	-3	1	955.70	30.00
0	-3	-1	949.90	29.00
0	3	-1	984.60	21.80
0	3	-1	967.40	30.30
0	3	1	980.40	24.80
0	3	-1	1005.40	24.70
0	3	1	973.00	27.40
0	3	1	911.61	32.80
0	3	-1	985.00	26.60
0	3	1	965.00	23.90
1	-3	1	461.75	13.50
-1	-3	-1	470.75	19.30
-1	-3	-1	465.55	16.50
-1	-3	-1	450.55	13.80
-1	-3	-1	463.65	15.90
-1	-3	-1	455.65	18.50
1	-3	1	449.56	16.40
1	-3	1	460.15	19.60
1	3	1	461.85	18.50
1	3	1	452.65	17.30
1	3	1	494.35	14.40
-1	3	-1	469.25	16.20
-1	3	-1	464.35	19.80
1	3	1	471.95	16.50
1	3	1	486.15	19.60
-1	3	-1	471.05	15.10
1	3	1	458.75	14.30

-2	-3	-1	90.09	8.50
2	-3	1	102.89	5.90
-2	-3	-1	109.09	9.80
-2	-3	-1	94.29	6.90
2	-3	1	83.59	6.20
-2	-3	-1	111.09	8.90
-2	-3	-1	93.69	6.00
2	-3	1	112.39	8.80
-2	3	-1	99.49	6.00
2	3	1	88.49	8.30
-2	3	-1	103.29	8.40
-2	3	-1	109.89	6.70
2	3	1	98.99	6.20
2	3	1	98.89	7.00
-2	3	-1	95.09	9.70
2	3	1	95.89	9.50
2	3	1	98.99	6.50
-2	3	-1	103.09	4.60
2	3	1	79.49	7.90
-3	-3	-1	19.20	5.30
3	-3	1	11.60	1.60
3	-3	1	10.40	1.90
-3	-3	-1	11.40	4.80
3	-3	1	15.30	2.80
-3	-3	-1	17.60	6.30
3	-3	1	15.90	4.80
3	-3	1	11.10	3.60
-3	-3	-1	14.90	3.60
-3	-3	-1	11.50	3.60
-3	3	-1	5.50	3.70
3	3	1	25.70	6.60

−3	3	−1	14.50	4.20
−3	3	−1	14.70	3.70
−3	3	−1	9.30	2.60
3	3	1	10.60	5.00
3	3	1	14.70	4.00
3	3	1	10.20	5.20
3	3	1	13.70	3.50
−4	−3	−1	12.70	6.50
4	−3	1	12.90	4.90
−4	−3	−1	9.90	5.20
4	−3	1	7.20	2.40
−4	−3	−1	9.90	5.70
4	−3	1	14.40	2.70
4	−3	1	10.60	2.80
−4	−3	−1	8.40	3.10
−4	−3	−1	8.70	3.90
−4	3	−1	15.40	3.60
4	3	1	0.70	5.00
4	3	1	9.50	3.50
−4	3	−1	11.90	4.90
4	3	1	−3.40	5.00
4	3	1	6.00	3.30
−4	3	−1	10.30	5.10
−5	−3	−1	159.58	10.70
5	−3	1	138.29	8.10
5	−3	1	132.89	8.00
−5	−3	−1	143.49	15.60
−5	−3	−1	169.68	17.10
−5	3	−1	121.09	16.10
5	3	1	153.38	11.20
−5	3	−1	134.79	9.50

−5	3	−1	156.18	14.60
5	3	1	132.29	11.60
−6	−3	−1	19.40	10.10
−6	−3	−1	33.80	11.40
6	−3	1	24.80	4.80
6	−3	1	22.70	4.80
6	−3	1	17.70	4.80
−6	3	−1	25.30	8.70
6	3	1	20.90	5.80
6	3	1	22.40	5.90
−6	3	−1	11.70	8.40
−7	−3	−1	30.70	12.80
7	−3	1	24.60	6.40
−7	−3	−1	40.40	13.50
7	−3	1	26.30	5.70
7	−3	1	18.90	5.90
7	3	1	27.60	7.90
−7	3	−1	39.90	11.10
7	3	1	41.80	8.20
−7	3	−1	35.80	13.60
8	−3	1	8.30	4.90
8	−3	1	4.70	4.60
−8	−3	−1	10.60	11.80
−8	−3	−1	7.00	13.90
8	−3	1	10.30	5.70
−8	3	−1	8.20	9.00
−8	3	−1	23.70	13.10
8	3	1	−0.40	6.20
9	−3	1	7.70	5.70
−9	−3	−1	−5.80	11.00
9	−3	1	18.90	5.30

9	-3	1	-7.90	5.30
-9	-3	-1	-9.70	12.30
9	3	1	12.10	7.30
-9	3	-1	20.30	14.60
-9	3	-1	21.50	14.60
-10	-3	-1	-17.40	15.00
10	-3	1	6.50	7.20
-10	-3	-1	6.50	15.00
-10	3	-1	13.90	18.30
10	3	1	-6.20	8.60
-10	3	-1	-7.30	14.20
-10	3	-1	3.70	15.40
11	-3	1	-1.90	9.50
-11	3	-1	27.80	21.40
11	3	1	11.10	11.30
-11	3	-1	-1.60	15.60
-11	-4	1	-3.60	25.00
11	-4	-1	-6.20	10.20
-11	-4	1	51.99	23.20
-11	4	1	31.60	20.60
-10	-4	1	-6.70	17.80
10	-4	-1	-20.10	9.60
10	-4	-1	-5.80	8.40
-10	-4	1	7.70	18.10
10	4	-1	5.10	8.80
-10	4	1	-15.00	14.10
-10	4	1	0.10	15.40
-10	4	1	0.10	18.50
-9	-4	1	18.40	15.70
-9	-4	1	16.90	16.60
9	-4	-1	29.40	9.40

9	−4	−1	27.00	8.80
−9	4	1	23.70	13.60
9	4	−1	30.70	9.40
−9	4	1	49.10	18.20
−9	4	1	37.20	18.00
8	−4	−1	40.50	9.20
8	−4	−1	27.10	7.40
−8	−4	1	16.70	12.50
−8	−4	1	5.00	12.60
8	4	−1	32.50	9.50
−8	4	1	11.50	11.20
−8	4	1	15.30	13.50
−8	4	1	43.50	15.50
−7	−4	1	14.40	9.60
7	−4	−1	1.20	4.70
−7	−4	1	0.50	8.50
7	−4	−1	−3.00	6.20
7	4	−1	−6.20	5.20
−7	4	1	3.80	10.20
−7	4	1	−2.80	8.10
−7	4	1	−2.20	8.50
−6	−4	1	79.49	15.50
6	−4	−1	71.99	10.90
−6	−4	1	92.09	17.90
6	−4	−1	95.59	8.90
−6	4	1	100.29	19.80
6	4	−1	104.99	12.10
−6	4	1	104.69	17.50
−6	4	1	88.49	15.60
5	−4	−1	5.10	8.00
−5	−4	1	6.60	6.60

−5	−4	1	9.00	5.70
5	−4	−1	2.60	6.40
−5	−4	1	−5.40	5.90
−5	4	1	14.30	9.00
5	4	−1	−0.40	3.30
−5	4	1	4.10	6.00
−4	−4	1	18.30	8.60
4	−4	−1	40.00	6.90
−4	−4	1	43.20	7.40
−4	−4	1	54.69	9.60
4	−4	−1	32.10	9.60
4	−4	−1	29.70	5.10
−4	−4	1	50.59	10.90
4	−4	−1	44.90	10.00
−4	−4	1	34.80	5.40
4	4	−1	46.30	10.70
4	4	−1	44.50	7.30
4	4	−1	47.60	10.70
−4	4	1	32.80	7.50
−4	4	1	42.70	7.40
4	4	−1	35.10	6.00
−4	4	1	28.60	10.50
−3	−4	1	487.45	24.20
3	−4	−1	521.25	24.70
−3	−4	1	485.15	20.30
3	−4	−1	493.45	20.60
3	−4	−1	530.65	22.40
3	−4	−1	515.55	16.50
−3	−4	1	486.25	17.10
−3	−4	1	541.55	21.60
−3	4	1	527.15	22.10

-3	4	1	517.95	24.60
3	4	-1	515.15	18.20
3	4	-1	513.45	24.50
-3	4	1	525.55	21.40
-3	4	1	457.55	27.20
3	4	-1	485.75	20.70
3	4	-1	508.55	25.10
-3	4	1	517.25	18.20
3	4	-1	537.25	23.20
-2	-4	1	90.19	5.60
2	-4	-1	88.89	8.00
-2	-4	1	63.49	8.40
2	-4	-1	85.49	7.30
-2	-4	1	92.09	7.90
2	-4	-1	78.59	12.30
2	-4	-1	75.79	6.80
-2	-4	1	84.39	8.80
-2	-4	1	98.49	10.30
2	-4	-1	71.79	9.70
-2	4	1	82.59	6.50
2	4	-1	75.29	7.20
-2	4	1	83.29	6.20
2	4	-1	80.29	9.60
2	4	-1	110.69	10.10
2	4	-1	80.19	7.30
-2	4	1	102.49	10.00
2	4	-1	93.79	6.00
-2	4	1	77.09	7.60
-1	-4	1	708.03	25.70
1	-4	-1	697.63	23.90
1	-4	-1	702.23	17.30

−1	−4	1	701.13	23.70
1	−4	−1	668.73	31.00
1	−4	−1	664.03	18.20
−1	−4	1	742.13	24.30
−1	−4	1	707.23	28.20
−1	−4	1	709.63	18.30
1	−4	−1	683.23	26.00
1	4	−1	710.23	23.50
1	4	−1	702.03	24.60
1	4	−1	733.93	24.60
1	4	−1	724.43	19.40
−1	4	1	697.03	32.00
−1	4	1	717.13	23.90
−1	4	1	711.13	25.90
−1	4	1	714.83	19.70
1	4	−1	746.63	24.10
−1	4	1	721.23	17.70
0	−4	1	353.96	14.60
0	−4	−1	350.96	11.00
0	−4	−1	324.27	15.50
0	−4	−1	327.67	14.60
0	−4	−1	338.07	15.90
0	−4	−1	314.67	19.60
0	−4	1	357.16	17.90
0	4	1	356.16	15.00
0	4	1	331.47	12.00
0	4	1	326.27	11.30
0	4	−1	354.76	9.90
0	4	−1	321.17	13.80
0	4	1	344.47	20.00
0	4	−1	335.77	18.10

-1	-4	-1	115.79	6.20
-1	-4	-1	118.29	7.50
1	-4	1	115.09	7.40
-1	-4	-1	127.79	11.20
-1	-4	-1	122.19	8.60
1	-4	1	116.29	11.20
1	-4	1	124.69	7.00
1	4	1	104.09	9.60
-1	4	-1	121.89	7.70
-1	4	-1	110.59	7.50
1	4	1	103.99	6.30
1	4	1	111.49	10.20
1	4	1	122.39	8.50
1	4	1	108.09	6.40
-1	4	-1	115.39	10.80
-2	-4	-1	137.19	7.70
2	-4	1	141.59	10.20
-2	-4	-1	120.59	8.90
2	-4	1	144.89	8.30
-2	-4	-1	162.98	12.70
2	-4	1	128.09	8.20
-2	-4	-1	145.09	10.50
-2	-4	-1	134.59	10.80
-2	4	-1	148.19	13.10
2	4	1	123.29	7.40
-2	4	-1	151.88	5.70
2	4	1	137.49	10.30
2	4	1	140.79	9.50
2	4	1	136.19	8.10
-2	4	-1	136.79	8.30
-2	4	-1	141.59	8.30

2	4	1	122.49	11.40
2	4	1	144.69	11.60
-2	4	-1	129.59	9.40
-3	-4	-1	665.13	27.60
3	-4	1	641.34	20.50
3	-4	1	645.34	22.60
-3	-4	-1	606.94	27.30
3	-4	1	654.23	27.40
-3	-4	-1	629.84	22.60
-3	4	-1	654.43	26.20
3	4	1	662.73	29.00
-3	4	-1	605.84	30.80
-3	4	-1	659.63	18.80
3	4	1	648.64	27.60
3	4	1	642.04	21.60
3	4	1	662.53	22.00
-3	4	-1	610.34	22.80
-3	4	-1	677.43	21.90
3	4	1	662.73	23.60
4	-4	1	3.50	7.10
-4	-4	-1	2.50	3.70
4	-4	1	-3.00	2.20
4	-4	1	4.10	4.70
-4	-4	-1	6.80	6.40
-4	-4	-1	10.10	5.70
4	-4	1	3.60	1.50
4	-4	1	0.10	1.30
-4	-4	-1	-1.80	5.80
-4	-4	-1	-0.70	2.70
-4	4	-1	1.10	4.30
-4	4	-1	3.10	3.80

4	4	1	−0.30	3.60
4	4	1	−6.70	5.10
4	4	1	0.50	3.00
4	4	1	8.00	7.20
−4	4	−1	2.40	5.90
−4	4	−1	2.80	4.20
4	4	1	−2.90	4.50
−4	4	−1	−4.30	2.30
−5	−4	−1	414.36	19.60
5	−4	1	467.45	16.70
5	−4	1	423.46	14.20
5	−4	1	419.66	13.30
−5	4	−1	458.45	26.50
5	4	1	412.66	18.90
−5	4	−1	407.36	22.50
5	4	1	408.66	19.90
−5	4	−1	441.66	30.50
−5	4	−1	408.66	16.70
−6	−4	−1	14.80	6.00
−6	−4	−1	14.20	8.00
−6	−4	−1	3.90	7.00
6	−4	1	9.80	3.80
6	−4	1	13.20	4.30
6	−4	1	13.40	4.50
−6	4	−1	2.50	10.20
6	4	1	12.30	5.50
−6	4	−1	−1.40	7.00
−6	4	−1	15.50	6.60
7	−4	1	19.20	5.20
−7	−4	−1	2.20	9.00
7	−4	1	8.20	4.50

-7	-4	-1	9.40	11.00
7	-4	1	11.40	4.40
-7	4	-1	15.30	8.30
-7	4	-1	8.10	10.50
-7	4	-1	3.40	10.80
7	4	1	10.70	5.30
8	-4	1	4.30	5.60
-8	-4	-1	-12.70	12.20
8	-4	1	-2.00	6.30
8	-4	1	10.50	4.40
-8	-4	-1	-9.50	10.50
8	4	1	-6.20	6.40
-8	4	-1	-14.60	10.60
-8	4	-1	8.80	9.70
-8	4	-1	14.50	12.70
9	-4	1	29.10	6.50
9	-4	1	33.40	13.10
9	-4	1	29.50	6.20
-9	-4	-1	42.10	17.50
9	-4	1	30.00	7.80
-9	-4	-1	16.50	14.70
-9	4	-1	45.60	17.40
-9	4	-1	47.50	16.30
9	4	1	46.50	11.00
-9	4	-1	23.60	14.60
-10	-4	-1	18.50	19.80
10	-4	1	1.80	8.40
-10	-4	-1	4.30	17.70
10	4	1	-3.20	8.50
-10	4	-1	25.70	17.20
-10	4	-1	4.40	14.10

−10	4	−1	−0.30	15.10
11	−4	1	1.50	10.40
−11	−4	−1	4.50	21.30
−11	−4	−1	−3.60	20.40
−11	4	−1	19.90	17.40
11	4	1	13.90	9.40
−11	4	−1	2.20	22.90
−11	4	−1	41.30	19.10
−11	−5	1	−35.60	20.90
−11	−5	1	7.00	22.70
11	−5	−1	2.70	11.40
−11	5	1	8.40	20.70
−11	5	1	−12.40	20.30
−11	5	1	6.00	22.70
11	5	−1	4.30	12.40
−10	−5	1	72.29	22.90
10	−5	−1	−2.50	9.90
−10	−5	1	20.90	21.50
10	−5	−1	4.10	8.60
−10	5	1	12.80	15.80
−10	5	1	35.30	20.20
10	5	−1	16.60	10.50
−10	5	1	11.20	18.40
9	−5	−1	18.80	9.10
−9	−5	1	17.90	15.00
9	−5	−1	1.20	7.20
−9	−5	1	26.70	17.10
−9	5	1	7.40	17.20
−9	5	1	8.20	14.90
9	5	−1	11.30	7.90
−9	5	1	−11.00	12.00

-8	-5	1	-0.70	11.50
8	-5	-1	8.30	7.60
-8	-5	1	-16.70	12.60
-8	5	1	5.70	11.40
8	5	-1	12.90	6.80
-8	5	1	20.90	13.40
-8	5	1	-10.60	11.30
-7	-5	1	8.00	11.20
7	-5	-1	16.40	8.00
-7	-5	1	21.50	12.50
7	-5	-1	21.50	6.50
-7	5	1	41.10	12.90
-7	5	1	29.10	12.70
-7	5	1	26.80	14.00
7	5	-1	26.20	7.80
-6	-5	1	64.79	16.10
-6	-5	1	53.09	14.10
6	-5	-1	92.19	9.50
6	-5	-1	72.79	11.30
-6	5	1	73.89	14.00
-6	5	1	80.59	18.40
-6	5	1	73.59	14.10
6	5	-1	88.09	11.70
-5	-5	1	66.29	14.90
-5	-5	1	35.70	7.80
5	-5	-1	48.50	8.60
5	-5	-1	52.99	6.80
5	-5	-1	33.10	9.40
-5	-5	1	53.69	12.30
-5	-5	1	58.49	10.60
-5	5	1	56.29	11.20

−5	5	1	47.90	11.50
5	5	−1	56.19	8.70
−5	5	1	55.09	15.20
−4	−5	1	525.05	29.10
4	−5	−1	490.45	22.50
4	−5	−1	521.55	17.30
−4	−5	1	534.25	24.70
−4	−5	1	515.35	23.40
4	−5	−1	524.35	25.50
−4	−5	1	525.05	30.40
−4	−5	1	510.45	19.50
−4	5	1	516.35	20.20
4	5	−1	528.65	29.40
−4	5	1	536.55	28.10
4	5	−1	549.95	29.10
−4	5	1	519.35	27.40
4	5	−1	528.55	20.60
−4	5	1	513.85	28.20
3	−5	−1	100.39	16.20
−3	−5	1	98.29	12.60
−3	−5	1	80.79	10.00
−3	−5	1	98.99	7.30
3	−5	−1	95.29	12.70
−3	−5	1	78.79	10.30
3	−5	−1	96.29	11.20
3	−5	−1	82.09	5.40
−3	−5	1	94.99	16.40
3	5	−1	93.19	13.30
−3	5	1	80.49	7.60
3	5	−1	105.89	13.50
3	5	−1	94.79	9.10

-3	5	1	80.49	8.50
3	5	-1	83.89	10.30
-3	5	1	98.59	11.60
3	5	-1	98.29	7.90
-3	5	1	88.89	15.90
-3	5	1	83.39	12.30
2	-5	-1	400.66	12.20
-2	-5	1	410.06	20.90
-2	-5	1	421.96	19.20
-2	-5	1	416.56	14.00
-2	-5	1	388.96	21.50
2	-5	-1	407.26	25.80
-2	-5	1	429.66	20.00
2	-5	-1	438.06	21.60
2	-5	-1	404.96	21.20
-2	5	1	418.16	27.30
-2	5	1	414.56	15.20
2	5	-1	419.86	18.80
-2	5	1	402.16	18.60
2	5	-1	397.36	20.10
2	5	-1	418.16	20.30
2	5	-1	448.16	15.40
-2	5	1	419.46	15.20
2	5	-1	410.26	18.60
1	-5	-1	119.59	13.00
-1	-5	1	109.39	12.80
1	-5	-1	76.39	12.50
-1	-5	1	109.39	9.30
-1	-5	1	88.79	8.90
-1	-5	1	97.89	5.10
-1	-5	1	96.69	8.20

−1	5	1	93.89	8.40
−1	5	1	99.29	6.10
−1	5	1	99.59	9.80
1	5	−1	93.59	9.60
1	5	−1	105.69	9.30
1	5	−1	112.19	9.20
1	5	−1	95.59	8.60
1	5	−1	85.19	5.50
0	−5	1	127.79	12.70
0	−5	−1	129.79	10.70
0	−5	−1	111.09	9.90
0	−5	1	103.79	9.80
0	−5	−1	120.99	15.50
0	5	−1	114.59	5.60
0	5	−1	122.09	9.10
0	5	1	104.79	6.90
0	5	1	138.19	9.90
0	5	1	137.69	13.80
0	5	−1	118.79	9.10
0	5	−1	110.49	11.80
0	5	1	123.59	6.90
−1	−5	−1	75.29	6.90
−1	−5	−1	82.89	5.80
1	−5	1	74.19	7.00
−1	−5	−1	108.49	11.80
1	−5	1	95.69	12.90
1	−5	1	85.09	7.70
−1	−5	−1	71.39	9.40
−1	−5	−1	88.29	8.30
−1	5	−1	84.89	6.90
1	5	1	83.19	11.30

1	5	1	93.09	6.50
-1	5	-1	73.09	7.00
1	5	1	83.99	6.20
1	5	1	87.79	6.60
1	5	1	105.49	10.30
-1	5	-1	87.59	12.90
2	-5	1	974.00	30.40
-2	-5	-1	1016.00	37.30
-2	-5	-1	1018.70	34.20
-2	-5	-1	1031.70	37.50
-2	-5	-1	1031.70	28.20
-2	-5	-1	1042.30	33.60
2	-5	1	1000.00	29.60
2	5	1	950.80	35.80
2	5	1	1074.09	33.50
2	5	1	991.90	31.50
-2	5	-1	1019.80	24.20
-2	5	-1	1084.69	48.20
-2	5	-1	990.30	31.20
-2	5	-1	1011.90	30.90
2	5	1	986.70	37.00
2	5	1	1029.50	32.80
-2	5	-1	966.50	27.70
3	-5	1	18.90	6.70
3	-5	1	20.10	4.40
-3	-5	-1	4.30	9.10
-3	-5	-1	18.00	4.00
3	-5	1	19.70	5.00
-3	-5	-1	16.10	6.20
-3	-5	-1	18.60	5.20
3	5	1	15.30	4.10

3	5	1	9.30	5.80
−3	5	−1	14.30	5.50
3	5	1	22.20	5.40
3	5	1	20.30	7.50
3	5	1	9.70	5.40
−3	5	−1	16.70	4.50
3	5	1	14.20	5.30
−3	5	−1	19.70	8.50
−3	5	−1	18.40	3.50
−3	5	−1	11.50	4.10
4	−5	1	117.19	8.70
−4	−5	−1	133.79	18.70
−4	−5	−1	119.39	15.60
−4	−5	−1	133.99	11.50
4	−5	1	152.38	20.10
−4	−5	−1	114.89	15.90
4	−5	1	104.39	11.80
−4	5	−1	132.39	13.10
4	5	1	121.89	11.20
−4	5	−1	119.69	7.60
−4	5	−1	100.19	17.00
4	5	1	101.79	11.80
−4	5	−1	129.79	10.50
4	5	1	132.49	13.80
4	5	1	101.69	14.80
−4	5	−1	106.49	10.90
4	5	1	127.99	17.10
5	−5	1	21.60	4.80
−5	−5	−1	19.70	8.60
5	−5	1	12.50	9.80
−5	−5	−1	14.70	5.80

-5	-5	-1	26.60	10.40
-5	5	-1	10.70	4.50
-5	5	-1	22.10	5.30
-5	5	-1	19.20	8.10
-5	5	-1	29.00	11.60
5	5	1	16.20	5.80
-5	5	-1	20.00	7.70
-6	-5	-1	74.59	17.20
-6	-5	-1	61.09	15.60
-6	-5	-1	46.70	8.50
6	-5	1	50.79	4.80
6	-5	1	52.79	6.70
-6	5	-1	51.89	11.40
-6	5	-1	42.20	11.80
-6	5	-1	45.90	13.90
6	5	1	42.30	8.30
7	-5	1	-0.40	3.70
7	-5	1	-0.60	3.90
-7	-5	-1	7.60	11.10
-7	-5	-1	3.50	10.60
7	-5	1	-6.70	3.90
7	5	1	-1.70	5.40
-7	5	-1	1.40	9.40
-7	5	-1	7.50	9.40
-7	5	-1	5.30	9.00
8	-5	1	38.40	7.00
-8	-5	-1	33.10	15.50
8	-5	1	36.10	6.90
-8	-5	-1	49.30	18.10
-8	5	-1	32.00	12.60
-8	5	-1	77.09	23.20

8	5	1	54.49	10.30
-8	5	-1	53.19	16.70
-9	-5	-1	24.60	14.00
-9	-5	-1	-5.00	13.10
9	-5	1	1.20	5.30
9	-5	1	1.90	5.90
-9	5	-1	-7.90	12.30
-9	5	-1	5.10	11.00
9	5	1	-9.40	6.60
-9	5	-1	-3.00	12.10
10	-5	1	-1.40	7.90
-10	-5	-1	-3.80	16.90
-10	-5	-1	-4.80	18.20
-10	5	-1	28.60	21.20
-10	5	-1	13.00	13.40
10	5	1	14.40	10.20
-10	5	-1	5.90	16.50
-11	-5	-1	14.70	21.70
-11	-5	-1	-22.60	22.10
-11	5	-1	19.70	22.50
-11	5	-1	-3.00	19.60
11	5	1	-1.00	11.70
-11	5	-1	38.30	18.40
10	-6	-1	-2.50	11.10
10	-6	-1	-2.40	17.00
-10	-6	1	17.80	18.80
-10	-6	1	10.50	18.80
-10	6	1	21.10	20.50
10	6	-1	-12.70	9.60
-10	6	1	6.80	17.90
-10	6	1	-14.60	15.30

9	-6	-1	19.30	10.00
9	-6	-1	14.40	7.80
-9	-6	1	-7.70	14.20
-9	-6	1	28.40	19.10
-9	6	1	10.40	16.00
-9	6	1	23.90	17.10
-9	6	1	21.50	14.90
9	6	-1	18.20	8.70
8	-6	-1	41.70	8.60
-8	-6	1	18.10	15.00
8	-6	-1	21.00	7.20
8	-6	-1	21.20	9.10
-8	-6	1	34.20	19.80
-8	6	1	21.60	14.20
-8	6	1	38.90	17.40
-8	6	1	40.00	17.30
8	6	-1	19.10	8.10
-7	-6	1	114.89	25.30
7	-6	-1	78.69	12.30
7	-6	-1	92.59	9.40
-7	-6	1	130.09	22.90
7	6	-1	99.29	13.10
-7	6	1	101.09	21.80
-7	6	1	80.09	17.70
-7	6	1	86.49	17.90
-6	-6	1	83.29	16.30
6	-6	-1	99.39	8.50
-6	-6	1	107.79	20.20
6	-6	-1	109.79	13.80
-6	6	1	76.49	19.40
-6	6	1	90.09	15.60

6	6	−1	104.39	12.60
−6	6	1	130.59	20.10
−5	−6	1	21.90	7.10
5	−6	−1	26.50	4.30
−5	−6	1	38.50	10.20
5	−6	−1	35.50	10.30
5	−6	−1	17.90	7.60
−5	−6	1	16.60	12.50
−5	−6	1	31.40	12.20
−5	6	1	17.10	11.60
5	6	−1	29.00	6.50
−5	6	1	36.90	9.40
−5	6	1	16.70	7.90
−5	6	1	35.70	8.80
4	−6	−1	−1.90	2.30
−4	−6	1	−0.60	6.50
−4	−6	1	−7.60	6.10
4	−6	−1	2.90	4.40
−4	−6	1	4.00	3.80
4	−6	−1	13.30	6.70
−4	−6	1	5.00	7.30
−4	6	1	−3.20	5.60
4	6	−1	5.60	9.30
−4	6	1	6.70	5.40
4	6	−1	−4.30	3.50
4	6	−1	11.50	8.40
−4	6	1	1.40	4.40
−4	6	1	5.70	9.30
4	6	−1	5.80	5.30
−4	6	1	2.30	4.40
−3	−6	1	224.28	15.80

3	−6	−1	237.88	14.60
−3	−6	1	219.28	16.20
3	−6	−1	239.18	7.90
3	−6	−1	261.47	15.90
−3	−6	1	232.98	19.40
−3	−6	1	233.78	18.10
3	−6	−1	221.38	18.90
−3	6	1	241.38	12.80
−3	6	1	220.58	22.00
3	6	−1	258.17	13.00
3	6	−1	252.17	19.10
−3	6	1	258.87	16.70
3	6	−1	207.98	19.60
3	6	−1	249.08	16.00
−3	6	1	222.58	18.00
−3	6	1	233.88	13.50
2	−6	−1	273.97	8.40
2	−6	−1	258.87	11.60
−2	−6	1	276.87	18.60
2	−6	−1	283.37	16.30
2	−6	−1	307.27	24.00
2	−6	−1	277.07	18.60
−2	−6	1	274.07	17.80
−2	−6	1	253.77	17.40
−2	−6	1	309.77	17.10
−2	−6	1	247.98	10.70
−2	6	1	265.57	11.00
−2	6	1	279.57	16.40
2	6	−1	243.98	16.30
2	6	−1	295.07	17.90
−2	6	1	263.37	17.50

-2	6	1	264.77	23.10
-2	6	1	271.47	12.10
2	6	-1	263.67	15.80
1	-6	-1	202.38	16.70
-1	-6	1	204.08	8.00
-1	-6	1	209.48	16.70
1	-6	-1	205.88	20.50
1	-6	-1	207.28	13.70
-1	-6	1	187.18	13.20
-1	-6	1	194.38	13.30
1	6	-1	182.98	9.10
-1	6	1	181.08	12.70
1	6	-1	211.38	15.20
1	6	-1	181.78	14.60
1	6	-1	183.08	12.10
-1	6	1	201.68	9.50
-1	6	1	188.38	18.60
1	6	-1	207.68	13.30
0	-6	1	321.37	20.90
0	-6	1	291.87	14.60
0	-6	-1	303.37	16.60
0	-6	1	240.98	13.20
0	-6	-1	299.77	17.20
0	-6	1	310.87	16.20
0	-6	-1	296.77	17.80
0	6	-1	287.37	15.20
0	6	1	291.47	16.30
0	6	-1	308.97	10.90
0	6	1	310.77	12.90
0	6	1	295.37	11.90
0	6	-1	289.97	14.10

0	6	−1	319.87	21.30
0	6	1	275.57	21.00
−1	−6	−1	46.60	5.40
−1	−6	−1	65.29	9.00
1	−6	1	53.39	7.50
−1	−6	−1	55.79	7.00
−1	−6	−1	58.69	12.80
1	−6	1	51.69	7.30
−1	−6	−1	38.90	8.10
−1	−6	−1	39.90	8.30
1	−6	1	33.00	10.00
1	6	1	55.79	5.70
−1	6	−1	44.00	5.90
1	6	1	39.00	7.00
1	6	1	47.30	10.00
−1	6	−1	47.70	3.70
1	6	1	40.00	5.70
1	6	1	45.10	6.60
−1	6	−1	56.69	7.90
2	−6	1	38.30	6.70
−2	−6	−1	48.70	8.20
2	−6	1	35.60	6.50
−2	−6	−1	44.70	8.70
2	−6	1	49.50	11.70
−2	−6	−1	42.80	7.90
−2	−6	−1	24.40	8.80
−2	−6	−1	46.50	5.90
2	6	1	32.60	8.00
−2	6	−1	43.30	3.90
2	6	1	26.20	8.30
−2	6	−1	39.60	8.00

-2	6	-1	41.70	11.00
2	6	1	31.00	4.90
2	6	1	49.40	6.50
2	6	1	56.79	10.20
2	6	1	44.20	8.00
-2	6	-1	47.60	7.00
-3	-6	-1	158.38	12.80
3	-6	1	164.98	13.80
-3	-6	-1	150.18	15.40
-3	-6	-1	166.48	16.50
-3	-6	-1	142.59	9.60
3	-6	1	141.39	13.30
3	6	1	144.19	9.90
-3	6	-1	147.19	8.40
-3	6	-1	123.19	10.80
3	6	1	124.09	16.00
3	6	1	151.88	14.00
3	6	1	153.78	14.20
-3	6	-1	145.89	11.60
-3	6	-1	155.28	20.50
-4	-6	-1	35.00	12.20
4	-6	1	24.50	9.50
-4	-6	-1	22.80	8.90
4	-6	1	22.70	6.50
-4	6	-1	22.90	9.40
4	6	1	21.30	8.00
-4	6	-1	35.10	7.70
4	6	1	15.10	9.20
-4	6	-1	23.70	7.00
4	6	1	38.20	11.70
4	6	1	27.70	6.20

−4	6	−1	24.00	5.30
−4	6	−1	25.10	6.90
−5	−6	−1	123.49	19.10
−5	−6	−1	110.09	18.60
5	−6	1	122.59	20.40
−5	−6	−1	97.89	12.80
−5	6	−1	121.09	12.50
−5	6	−1	99.89	14.30
5	6	1	106.19	16.80
−5	6	−1	100.29	9.30
5	6	1	95.99	11.40
−5	6	−1	102.29	18.80
−5	6	−1	80.19	13.30
−6	−6	−1	29.00	12.20
−6	−6	−1	70.79	16.50
−6	−6	−1	54.29	10.50
−6	6	−1	51.99	15.80
−6	6	−1	70.79	13.20
−6	6	−1	92.39	15.90
6	6	1	72.19	10.90
−7	−6	−1	−1.80	6.00
−7	−6	−1	−6.80	10.10
−7	−6	−1	4.10	11.70
7	6	1	5.80	6.20
−7	6	−1	26.50	14.20
−7	6	−1	0.90	8.60
−7	6	−1	16.20	11.30
8	−6	1	−8.50	4.60
−8	6	−1	4.50	15.30
−8	6	−1	−5.80	10.00
8	6	1	−3.50	5.90

-8	6	-1	3.10	11.00
-9	-6	-1	6.40	17.30
-9	-6	-1	13.40	17.20
-9	6	-1	0.30	16.10
-9	6	-1	-3.80	13.50
9	6	1	4.40	8.40
-9	6	-1	4.40	11.60
-10	-6	-1	-20.30	18.60
-10	-6	-1	-40.00	16.50
-10	6	-1	20.80	17.60
-10	6	-1	15.00	21.80
-10	6	-1	-7.50	13.70
10	6	1	10.00	11.50
-10	-7	1	-28.70	20.50
-10	-7	1	4.90	22.30
-10	7	1	11.10	35.10
-10	7	1	15.10	18.10
10	7	-1	-1.60	10.60
-10	7	1	8.20	16.70
-9	-7	1	1.20	17.50
-9	-7	1	-23.00	15.20
9	-7	-1	7.00	9.90
9	7	-1	17.80	10.70
-9	7	1	-8.90	18.20
-9	7	1	33.30	16.40
-9	7	1	17.10	15.80
8	-7	-1	6.60	8.00
-8	-7	1	-6.90	13.00
8	-7	-1	11.20	4.40
8	7	-1	-1.80	7.80
-8	7	1	8.40	12.50

-8	7	1	3.20	15.90
7	-7	-1	6.00	7.40
-7	-7	1	-4.00	12.20
7	-7	-1	1.90	3.40
-7	-7	1	-1.50	12.60
-7	7	1	3.60	13.60
-7	7	1	-10.80	10.00
-7	7	1	4.00	11.00
6	-7	-1	23.40	8.10
-6	-7	1	17.30	6.50
-6	-7	1	24.10	13.00
6	-7	-1	13.90	2.90
-6	-7	1	1.00	11.00
-6	7	1	24.00	14.70
-6	7	1	15.20	9.30
6	7	-1	4.30	5.20
-5	-7	1	11.00	6.10
-5	-7	1	0.50	8.00
5	-7	-1	16.80	6.20
-5	-7	1	4.70	10.20
5	-7	-1	-6.10	10.40
-5	7	1	3.40	10.20
-5	7	1	20.20	9.80
5	7	-1	16.80	6.10
-5	7	1	38.60	9.60
4	-7	-1	4.50	7.40
-4	-7	1	-3.20	7.60
-4	-7	1	-0.80	7.10
-4	-7	1	2.90	5.30
-4	-7	1	-5.00	8.10
4	-7	-1	0.30	5.20

-4	7	1	8.80	5.50
-4	7	1	-11.00	8.90
4	7	-1	1.20	5.40
-4	7	1	8.60	5.30
4	7	-1	-2.70	4.00
4	7	-1	5.70	8.30
-3	-7	1	59.39	13.20
-3	-7	1	68.39	7.30
-3	-7	1	75.49	15.30
-3	-7	1	43.70	8.30
-3	-7	1	61.79	13.80
3	-7	-1	84.59	10.40
3	-7	-1	57.99	10.20
-3	7	1	48.10	10.50
3	7	-1	48.50	10.90
-3	7	1	81.19	17.20
3	7	-1	59.59	8.30
-3	7	1	68.29	8.50
3	7	-1	49.00	9.50
3	7	-1	46.80	11.20
-3	7	1	64.99	8.90
-2	-7	1	215.48	16.70
-2	-7	1	197.98	15.30
-2	-7	1	216.68	16.70
-2	-7	1	225.28	18.50
2	-7	-1	207.08	14.80
-2	-7	1	213.18	9.80
2	-7	-1	218.08	18.80
-2	7	1	214.78	11.00
-2	7	1	239.98	24.30
-2	7	1	198.48	8.40

2	7	-1	185.38	15.70
2	7	-1	196.18	14.60
-2	7	1	208.18	16.20
2	7	-1	198.98	11.20
1	-7	-1	63.89	11.30
-1	-7	1	83.49	11.30
1	-7	-1	77.49	16.60
1	-7	-1	85.69	11.10
-1	-7	1	86.49	14.60
-1	-7	1	83.29	11.10
-1	7	1	78.79	6.60
1	7	-1	94.49	9.10
-1	7	1	80.89	17.00
1	7	-1	77.49	6.80
1	7	-1	109.89	20.60
-1	7	1	63.69	7.30
0	-7	-1	309.37	24.30
0	-7	1	319.27	23.20
0	-7	1	296.87	16.40
0	-7	-1	289.57	19.00
0	-7	-1	303.17	17.90
0	-7	1	278.97	15.20
0	-7	1	262.07	16.30
0	7	-1	273.07	22.00
0	7	-1	283.27	11.20
0	7	-1	307.07	11.80
0	7	1	278.37	13.50
0	7	1	305.77	24.20
0	7	-1	285.47	14.60
1	-7	1	135.49	11.50
1	-7	1	169.98	17.80

−1	−7	−1	137.39	13.40
−1	−7	−1	184.18	18.30
−1	−7	−1	132.49	9.10
1	−7	1	139.49	13.40
−1	−7	−1	137.39	12.00
−1	7	−1	142.79	6.90
1	7	1	131.59	9.40
−1	7	−1	182.38	19.80
1	7	1	135.29	8.70
1	7	1	136.69	9.30
−1	7	−1	128.09	12.10
1	7	1	147.59	18.80
−1	7	−1	145.79	9.30
2	−7	1	4.50	8.30
−2	−7	−1	6.70	7.20
−2	−7	−1	2.60	4.40
−2	−7	−1	−3.50	4.70
−2	−7	−1	26.10	9.50
−2	−7	−1	2.00	3.30
2	−7	1	2.60	3.50
2	−7	1	6.80	5.90
2	7	1	4.60	4.60
−2	7	−1	2.60	2.10
2	7	1	−1.00	6.20
−2	7	−1	15.90	8.70
2	7	1	3.40	5.50
2	7	1	−1.30	3.50
−2	7	−1	3.10	3.70
−2	7	−1	−1.30	3.90
3	−7	1	11.80	4.90
−3	−7	−1	5.10	4.40

3	−7	1	9.90	6.00
−3	−7	−1	4.50	7.60
−3	−7	−1	28.90	11.70
−3	−7	−1	7.00	3.90
3	7	1	12.90	7.30
−3	7	−1	15.10	9.00
3	7	1	13.70	6.70
−3	7	−1	9.50	4.90
−3	7	−1	10.40	4.50
−3	7	−1	7.30	4.00
−3	7	−1	13.80	5.40
−4	−7	−1	15.10	5.60
4	−7	1	2.80	8.60
−4	−7	−1	18.90	10.60
4	−7	1	9.50	4.50
−4	−7	−1	3.00	7.60
4	7	1	5.60	4.70
4	7	1	16.40	8.10
−4	7	−1	5.10	5.40
−4	7	−1	28.70	12.70
−4	7	−1	12.30	7.10
−4	7	−1	8.40	6.30
−4	7	−1	10.60	4.80
4	7	1	15.40	8.60
5	−7	1	0.80	10.30
−5	−7	−1	1.50	7.70
−5	−7	−1	0.00	10.70
5	−7	1	7.80	4.90
−5	−7	−1	9.40	6.40
5	7	1	3.90	6.80
−5	7	−1	10.10	7.40

-5	7	-1	2.80	7.00
5	7	1	1.10	4.90
-5	7	-1	-7.70	4.40
-5	7	-1	4.10	8.20
-6	-7	-1	19.30	13.10
-6	-7	-1	1.10	6.20
6	-7	1	22.10	6.60
-6	-7	-1	20.80	12.00
-6	7	-1	8.30	7.80
-6	7	-1	14.00	8.00
6	7	1	9.00	6.30
-6	7	-1	27.80	13.20
-6	7	-1	17.20	9.30
-7	-7	-1	37.30	9.60
-7	-7	-1	40.50	15.90
-7	-7	-1	25.10	14.50
7	-7	1	37.60	7.90
7	7	1	46.40	11.80
-7	7	-1	47.00	12.30
-7	7	-1	55.29	15.40
-8	-7	-1	14.60	15.40
8	-7	1	14.60	6.40
-8	-7	-1	2.50	16.80
-8	7	-1	-9.00	10.80
-8	7	-1	3.20	13.20
-8	7	-1	26.60	14.40
8	7	1	4.20	8.00
9	-7	1	-8.20	9.90
-9	-7	-1	24.70	18.20
-9	-7	-1	2.40	17.30
-9	7	-1	7.90	20.00

9	7	1	18.70	10.50
-9	7	-1	7.60	16.00
-9	7	-1	-2.30	13.20
-10	-7	-1	-22.40	21.20
-10	-7	-1	-9.60	20.70
-10	7	-1	-13.00	19.50
-10	7	-1	-27.90	15.90
-10	7	-1	18.30	18.50
10	7	1	17.00	19.90
-10	-8	1	-53.89	25.80
-10	-8	1	15.20	22.70
-10	8	1	-16.80	18.10
-10	8	1	-14.00	23.40
10	8	-1	-2.20	12.60
-10	8	1	-10.40	17.80
-9	-8	1	-5.00	16.90
9	-8	-1	8.30	10.80
-9	-8	1	-3.90	18.90
-9	8	1	-14.30	20.60
-9	8	1	-26.00	15.90
9	8	-1	15.00	11.30
-8	-8	1	-2.70	15.40
8	-8	-1	7.90	9.60
-8	-8	1	20.90	17.80
-8	8	1	2.00	13.40
-8	8	1	18.50	17.30
8	8	-1	0.30	9.20
-8	8	1	5.40	12.50
7	-8	-1	-4.00	7.80
-7	-8	1	-26.10	11.00
-7	-8	1	13.20	13.90

-7	8	1	4.40	12.00
7	8	-1	9.20	7.00
-7	8	1	-3.00	13.10
-7	8	1	-6.30	10.00
-6	-8	1	49.99	11.60
-6	-8	1	44.90	14.80
6	-8	-1	32.10	9.70
-6	-8	1	25.90	16.30
6	8	-1	45.90	10.40
-6	8	1	43.90	11.50
-6	8	1	42.20	17.50
-6	8	1	19.10	11.50
5	-8	-1	40.00	10.10
5	-8	-1	6.80	11.90
-5	-8	1	24.00	12.40
-5	-8	1	31.80	9.30
-5	-8	1	61.49	15.70
-5	8	1	70.89	15.70
-5	8	1	21.10	14.10
5	8	-1	36.50	10.60
5	8	-1	34.70	7.70
-5	8	1	29.20	9.60
-4	-8	1	50.39	13.00
4	-8	-1	57.39	10.70
-4	-8	1	85.19	17.00
-4	-8	1	53.09	10.10
4	-8	-1	76.09	12.70
-4	-8	1	56.69	13.70
-4	8	1	77.59	14.50
4	8	-1	38.00	9.80
-4	8	1	58.39	9.40

4	8	−1	45.30	13.40
−4	8	1	70.99	17.30
4	8	−1	57.19	8.90
−3	−8	1	11.10	7.50
−3	−8	1	−1.40	8.60
−3	−8	1	11.60	3.90
−3	−8	1	10.00	7.20
3	−8	−1	2.70	5.10
3	8	−1	21.10	8.00
−3	8	1	14.50	5.20
−3	8	1	5.40	7.30
−3	8	1	6.20	4.10
3	8	−1	−0.20	7.70
−3	8	1	1.40	7.40
3	8	−1	14.00	6.90
3	8	−1	7.40	7.60
−2	−8	1	6.40	5.60
−2	−8	1	1.30	2.90
−2	−8	1	−23.00	7.80
−2	−8	1	−4.20	5.30
2	−8	−1	−2.10	3.80
−2	8	1	5.10	8.40
2	8	−1	−1.20	3.00
−2	8	1	−2.10	3.60
2	8	−1	2.10	6.60
2	8	−1	4.20	5.10
2	8	−1	3.70	5.40
1	−8	−1	18.60	7.10
−1	−8	1	14.10	9.30
−1	−8	1	22.30	6.80
−1	−8	1	25.50	7.30

1	-8	-1	28.10	7.80
1	8	-1	25.50	5.70
1	8	-1	21.60	6.30
1	8	-1	25.40	5.10
1	8	-1	34.40	9.80
-1	8	1	18.10	4.40
0	-8	-1	13.60	5.60
0	-8	1	16.60	5.70
0	-8	1	5.40	4.60
0	-8	-1	14.50	6.90
0	-8	1	9.70	7.20
0	-8	1	-2.50	9.40
0	8	-1	13.20	4.80
0	8	-1	14.40	3.60
0	8	-1	-3.00	7.00
0	8	-1	12.10	3.40
0	8	1	17.50	4.90
0	8	1	9.80	9.30
0	8	1	6.40	4.30
1	-8	1	8.80	5.50
1	-8	1	-4.70	9.90
1	-8	1	9.20	5.80
-1	-8	-1	-3.50	5.80
-1	-8	-1	0.60	4.80
1	-8	1	-3.10	3.30
-1	8	-1	1.10	2.70
1	8	1	0.60	3.80
-1	8	-1	5.70	4.70
-1	8	-1	5.20	7.80
-1	8	-1	7.90	3.50
1	8	1	1.50	7.20

-1	8	-1	8.20	3.20
2	-8	1	107.79	12.90
-2	-8	-1	85.39	12.60
-2	-8	-1	80.39	17.00
-2	-8	-1	57.59	10.30
2	-8	1	53.89	9.70
-2	-8	-1	62.79	8.10
-2	8	-1	78.79	19.50
-2	8	-1	70.99	7.80
2	8	1	56.99	11.90
-2	8	-1	67.59	6.00
2	8	1	77.69	9.50
2	8	1	82.49	10.40
-3	-8	-1	17.10	9.80
3	-8	1	26.10	7.80
3	-8	1	20.90	6.60
-3	-8	-1	30.90	8.10
-3	-8	-1	13.70	9.00
3	8	1	23.70	10.40
-3	8	-1	23.20	12.10
3	8	1	34.40	10.30
3	8	1	15.40	5.50
-3	8	-1	34.30	5.50
-3	8	-1	19.50	5.20
3	8	1	36.00	9.30
4	-8	1	51.49	9.30
-4	-8	-1	56.99	14.00
-4	-8	-1	25.80	9.20
-4	-8	-1	45.80	13.40
-4	8	-1	51.79	16.80
-4	8	-1	32.90	7.30

-4	8	-1	36.10	7.90
4	8	1	36.50	7.70
-4	8	-1	38.00	9.90
4	8	1	42.20	11.50
-5	-8	-1	6.90	10.30
5	-8	1	-20.90	11.40
5	-8	1	3.80	5.60
-5	-8	-1	-6.70	5.10
-5	-8	-1	6.50	10.40
-5	8	-1	0.90	5.50
-5	8	-1	8.30	11.90
5	8	1	6.90	8.00
5	8	1	1.00	5.80
-5	8	-1	-3.20	6.50
-6	-8	-1	15.90	14.10
-6	-8	-1	-3.30	11.70
6	-8	1	-0.50	5.90
-6	-8	-1	-7.70	6.80
6	8	1	-3.70	5.80
-6	8	-1	-3.30	7.60
-6	8	-1	10.10	8.30
-6	8	-1	-12.80	9.60
6	8	1	4.20	8.90
-7	-8	-1	36.00	17.50
-7	-8	-1	10.20	15.20
7	-8	1	1.80	6.50
-7	-8	-1	21.30	9.80
7	8	1	0.90	8.00
-7	8	-1	25.10	15.20
-7	8	-1	-2.70	10.10
-7	8	-1	11.40	11.30

-8	-8	-1	0.80	15.60
-8	-8	-1	-9.50	14.80
8	-8	1	-7.10	7.10
-8	8	-1	-13.60	15.20
-8	8	-1	-20.20	11.40
-8	8	-1	-2.40	12.00
8	8	1	0.00	7.30
-9	-8	-1	19.00	16.50
-9	-8	-1	-3.50	18.20
-9	8	-1	-13.50	14.00
9	8	1	9.80	9.20
-9	8	-1	-21.40	15.90
-9	8	-1	4.70	15.70
-10	-8	-1	-6.40	26.30
-10	-8	-1	11.40	24.90
-10	8	-1	0.50	25.00
-10	8	-1	10.10	17.70
10	8	1	-5.00	11.70
-10	8	-1	-0.40	20.30
-9	-9	1	53.79	26.80
-9	-9	1	-18.50	20.10
9	-9	-1	-8.70	12.00
-9	9	1	18.10	17.20
-8	-9	1	3.30	18.70
8	-9	-1	5.30	10.90
-8	-9	1	-3.00	17.10
-8	9	1	8.20	14.20
8	9	-1	6.70	9.30
-8	9	1	0.30	13.30
-8	9	1	9.60	18.60
-7	-9	1	-25.50	15.10

-7	-9	1	-9.20	14.40
-7	-9	1	-4.70	9.10
7	-9	-1	-3.40	8.10
7	9	-1	4.10	7.30
-7	9	1	-10.70	10.60
-7	9	1	17.40	19.90
-7	9	1	5.50	11.20
6	-9	-1	22.70	10.30
-6	-9	1	18.00	9.00
-6	-9	1	45.10	17.40
-6	-9	1	43.10	16.90
-6	9	1	12.00	10.10
6	9	-1	25.30	10.80
-6	9	1	-1.60	13.90
-6	9	1	22.90	12.40
-5	-9	1	-0.20	10.00
-5	-9	1	4.50	11.10
5	-9	-1	-11.20	12.90
5	-9	-1	1.80	8.10
-5	-9	1	2.10	7.70
5	9	-1	13.00	9.60
-5	9	1	3.60	12.10
-5	9	1	5.90	7.70
5	9	-1	12.60	6.80
-5	9	1	7.60	9.70
4	-9	-1	78.29	12.80
-4	-9	1	80.59	14.30
-4	-9	1	91.69	19.30
-4	-9	1	107.69	18.40
-4	9	1	111.09	11.30
4	9	-1	96.79	13.00

-4	9	1	97.99	22.30
4	9	-1	87.79	17.40
4	9	-1	101.99	15.00
-4	9	1	89.49	16.00
3	-9	-1	9.20	6.70
-3	-9	1	2.10	8.90
-3	-9	1	7.80	8.80
-3	-9	1	3.00	6.70
-3	-9	1	11.90	4.70
3	9	-1	8.60	7.30
-3	9	1	15.40	13.40
-3	9	1	7.10	3.70
3	9	-1	-2.60	9.60
3	9	-1	16.90	8.70
3	9	-1	3.50	4.70
-3	9	1	-9.00	5.80
-2	-9	1	117.69	19.30
-2	-9	1	112.69	15.30
2	-9	-1	96.99	13.80
-2	-9	1	89.49	15.20
-2	9	1	78.79	9.40
-2	9	1	151.78	27.20
2	9	-1	84.49	9.50
2	9	-1	94.99	12.50
2	9	-1	93.39	15.50
2	9	-1	91.39	15.00
1	-9	-1	93.49	14.10
1	-9	-1	114.79	16.70
-1	-9	1	107.59	14.60
-1	-9	1	111.79	21.80
-1	-9	1	125.09	20.10

1	9	-1	86.19	8.90
1	9	-1	120.39	10.70
1	9	-1	81.09	15.20
-1	9	1	108.39	21.20
-1	9	1	95.79	10.50
-1	9	1	95.99	10.20
0	-9	-1	10.80	6.50
0	-9	1	13.90	7.00
0	-9	1	16.10	6.00
0	-9	-1	13.50	6.60
0	9	1	18.80	6.00
0	9	-1	12.30	4.70
0	9	-1	21.70	5.10
0	9	-1	17.80	4.70
0	9	-1	19.90	10.90
0	9	1	4.60	10.60
-1	-9	-1	69.29	12.50
-1	-9	-1	45.10	11.20
1	-9	1	47.80	9.80
-1	9	-1	53.99	6.40
1	9	1	70.49	9.40
1	9	1	65.39	6.00
-1	9	-1	62.79	6.40
-1	9	-1	61.49	10.80
1	9	1	51.99	14.70
-1	9	-1	51.99	6.80
2	-9	1	214.18	17.00
-2	-9	-1	238.68	12.50
-2	-9	-1	198.78	14.80
-2	-9	-1	212.48	24.00
-2	-9	-1	211.68	18.10

-2	9	-1	237.68	26.00
-2	9	-1	218.88	10.90
2	9	1	197.68	14.30
2	9	1	241.08	23.00
2	9	1	242.48	14.30
-3	-9	-1	4.20	6.70
3	-9	1	6.10	5.00
-3	-9	-1	-0.90	8.70
-3	-9	-1	8.30	8.70
-3	9	-1	10.90	4.60
3	9	1	-4.40	10.40
3	9	1	4.90	7.60
-3	9	-1	13.00	11.60
3	9	1	18.10	6.20
-3	9	-1	6.50	4.90
3	9	1	11.60	8.10
-4	-9	-1	30.70	9.00
-4	-9	-1	28.90	11.30
-4	-9	-1	41.50	13.40
4	-9	1	40.30	9.40
-4	9	-1	34.20	7.10
-4	9	-1	23.30	13.80
4	9	1	34.30	12.20
4	9	1	44.10	9.20
-5	-9	-1	2.10	6.30
-5	-9	-1	16.50	11.80
5	-9	1	4.40	6.70
-5	-9	-1	23.30	14.30
-5	9	-1	14.80	14.00
5	9	1	38.70	12.60
5	9	1	21.10	8.10

-5	9	-1	8.80	8.50
-5	9	-1	10.70	8.30
6	-9	1	1.10	7.00
-6	-9	-1	6.10	13.90
-6	-9	-1	23.60	14.40
-6	-9	-1	2.50	7.30
-6	9	-1	21.00	19.70
6	9	1	6.00	6.50
-6	9	-1	7.40	9.30
6	9	1	-1.00	10.00
-6	9	-1	12.50	9.30
-7	-9	-1	30.90	17.00
-7	-9	-1	41.20	17.80
-7	-9	-1	0.10	9.20
7	-9	1	13.60	8.20
-7	9	-1	21.10	12.10
7	9	1	14.90	9.30
-7	9	-1	36.00	25.00
-7	9	-1	-5.70	11.40
-8	-9	-1	-1.40	10.30
-8	-9	-1	30.10	19.10
-8	-9	-1	12.40	17.00
-8	9	-1	12.90	21.00
-8	9	-1	35.50	15.10
-8	9	-1	-11.60	12.00
8	9	1	-8.20	9.30
-9	-9	-1	8.80	23.80
-9	-9	-1	-9.60	20.70
-9	9	-1	30.00	16.60
-9	9	-1	3.40	16.60
-9	-10	1	33.40	26.30

9	-10	-1	15.40	13.70
-9	-10	1	49.30	26.10
9	10	-1	-4.80	12.30
-9	10	1	-9.30	21.00
-9	10	1	25.90	19.30
-9	10	1	8.70	26.10
-8	-10	1	19.70	21.10
8	-10	-1	9.40	11.90
-8	-10	1	16.50	19.50
-8	10	1	0.90	15.80
-8	10	1	-9.10	19.00
-8	10	1	14.40	15.20
8	10	-1	-4.60	11.00
-7	-10	1	21.70	18.10
-7	-10	1	18.40	10.70
-7	-10	1	40.00	19.80
7	-10	-1	15.80	11.30
-7	10	1	35.00	23.70
7	10	-1	37.90	11.50
-7	10	1	19.10	13.40
-7	10	1	29.90	14.30
-6	-10	1	-3.70	14.20
-6	-10	1	31.50	17.30
-6	-10	1	-1.20	9.30
6	-10	-1	10.30	10.20
-6	10	1	-6.90	15.90
6	10	-1	11.40	8.60
-6	10	1	14.40	11.00
-6	10	1	8.00	10.40
-5	-10	1	30.20	15.70
5	-10	-1	47.60	12.20

-5	-10	1	38.80	11.90
-5	-10	1	38.20	14.80
-5	10	1	23.70	12.00
-5	10	1	26.20	15.50
-5	10	1	-7.20	24.20
5	10	-1	25.50	11.80
5	10	-1	33.00	9.70
4	-10	-1	-4.30	6.50
-4	-10	1	6.20	10.10
-4	-10	1	-3.20	6.20
-4	10	1	4.60	12.40
4	10	-1	-8.90	13.00
4	10	-1	8.30	6.30
4	10	-1	9.10	8.20
-4	10	1	2.90	7.50
-3	-10	1	165.08	22.30
3	-10	-1	212.88	18.60
-3	-10	1	185.58	19.00
-3	10	1	160.38	28.90
3	10	-1	165.08	13.30
3	10	-1	202.98	23.80
3	10	-1	174.28	18.80
2	-10	-1	19.40	7.80
-2	-10	1	22.90	9.10
-2	-10	1	36.90	12.10
2	-10	-1	39.90	10.60
-2	10	1	22.70	6.60
2	10	-1	30.50	8.40
2	10	-1	14.90	9.30
2	10	-1	26.40	5.90
-2	10	1	33.00	13.20

1	-10	-1	106.89	15.60
1	-10	-1	117.89	18.00
-1	-10	1	108.69	15.70
-1	-10	1	116.29	15.60
1	10	-1	101.89	9.70
-1	10	1	118.99	12.70
-1	10	1	111.89	22.90
1	10	-1	111.29	11.20
0	-10	-1	21.00	8.30
0	-10	-1	20.10	9.00
0	-10	1	8.30	6.50
0	-10	1	23.40	8.30
0	10	-1	21.60	5.90
0	10	1	9.80	12.20
0	10	-1	24.20	5.60
0	10	1	14.60	5.70
0	10	-1	8.90	10.40
0	10	-1	9.50	4.40
-1	-10	-1	3.20	6.50
-1	-10	-1	1.40	8.60
1	-10	1	-7.00	5.30
1	-10	1	-5.20	5.40
1	10	1	-16.10	8.70
-1	10	-1	5.00	4.60
1	10	1	3.10	4.90
-1	10	-1	0.90	9.70
-2	-10	-1	18.40	9.20
2	-10	1	2.30	6.80
-2	-10	-1	11.80	9.20
-2	10	-1	-4.90	10.70
-2	10	-1	16.10	5.10

-2	10	-1	11.40	4.90
-2	10	-1	15.80	4.00
2	10	1	19.30	6.60
2	10	1	-3.30	9.70
2	10	1	11.50	6.20
-3	-10	-1	238.58	26.70
3	-10	1	264.97	19.50
3	-10	1	248.38	17.00
-3	-10	-1	284.17	24.10
-3	-10	-1	205.08	19.60
-3	10	-1	240.68	14.00
-3	10	-1	247.68	13.70
3	10	1	207.48	26.10
-3	10	-1	304.77	38.50
3	10	1	189.88	18.00
3	10	1	246.98	16.70
-4	-10	-1	15.50	13.40
-4	-10	-1	9.10	8.20
4	-10	1	5.10	6.70
-4	-10	-1	10.30	10.10
4	10	1	-3.10	8.10
4	10	1	3.00	5.50
-4	10	-1	3.20	5.40
-4	10	-1	10.10	6.90
-4	10	-1	9.80	14.80
5	-10	1	110.69	15.00
-5	-10	-1	110.19	28.20
-5	-10	-1	108.39	21.50
-5	-10	-1	88.19	20.90
5	10	1	99.49	14.30
5	10	1	73.69	17.00

-5	10	-1	86.29	14.00
-5	10	-1	62.49	21.30
-6	-10	-1	12.10	9.40
6	-10	1	13.50	8.60
-6	-10	-1	9.40	17.20
-6	-10	-1	21.40	15.60
-6	10	-1	20.90	16.50
-6	10	-1	-7.40	9.70
6	10	1	8.30	8.30
-6	10	-1	12.60	8.50
6	10	1	-1.50	11.40
-7	-10	-1	-2.40	8.30
7	-10	1	6.50	9.30
-7	-10	-1	33.00	25.60
-7	-10	-1	-10.50	17.00
-7	10	-1	9.10	12.20
-7	10	-1	-8.60	9.30
-7	10	-1	3.70	18.30
7	10	1	6.50	9.20
-8	-10	-1	13.30	20.30
-8	-10	-1	-2.10	10.70
8	-10	1	10.40	11.30
-8	-10	-1	-24.60	19.80
-8	10	-1	22.50	12.80
-8	10	-1	24.90	19.50
8	10	1	-8.10	9.50
-8	10	-1	-18.00	13.90
-9	-10	-1	-39.50	24.80
-9	-10	-1	27.90	25.20
9	10	1	-3.00	12.00
-9	10	-1	22.10	16.40

-9	10	-1	-22.30	18.10
-9	10	-1	22.10	26.30
-8	-11	1	-42.10	22.50
8	-11	-1	-1.40	13.20
-8	-11	1	12.10	22.30
-8	11	1	-4.30	14.90
-8	11	1	-22.90	23.70
-8	11	1	-13.20	14.40
-7	-11	1	-16.10	19.80
-7	-11	1	37.10	25.50
7	-11	-1	9.10	11.00
-7	-11	1	6.70	11.80
-7	11	1	50.59	14.40
7	11	-1	10.20	10.40
-7	11	1	43.20	25.20
6	-11	-1	21.40	11.10
-6	-11	1	24.60	16.90
-6	-11	1	21.30	12.60
-6	-11	1	30.70	19.20
-6	11	1	37.00	10.90
-6	11	1	2.60	16.60
-6	11	1	30.50	14.00
6	11	-1	21.90	11.00
-5	-11	1	31.80	11.40
5	-11	-1	34.10	11.90
-5	-11	1	52.09	17.70
-5	-11	1	64.09	17.90
-5	11	1	34.30	9.50
5	11	-1	49.60	14.10
-5	11	1	43.10	21.80
-5	11	1	23.20	10.80

-4	-11	1	9.70	13.30
-4	-11	1	27.90	12.50
-4	-11	1	6.20	8.60
4	-11	-1	16.00	8.70
4	11	-1	34.70	9.50
4	11	-1	35.00	11.40
4	11	-1	-5.60	17.10
3	-11	-1	10.10	8.30
-3	-11	1	18.50	10.90
3	-11	-1	21.80	10.60
-3	11	1	-2.10	15.50
3	11	-1	6.30	9.20
3	11	-1	13.00	5.90
-3	11	1	8.80	6.90
3	11	-1	11.50	9.80
2	-11	-1	4.00	9.10
-2	-11	1	8.80	7.20
2	-11	-1	16.20	8.80
-2	-11	1	-0.70	10.30
2	11	-1	2.70	5.30
-2	11	1	4.70	5.50
2	11	-1	11.80	10.10
-2	11	1	18.00	16.00
2	11	-1	14.90	7.90
-1	-11	1	163.28	20.30
1	-11	-1	151.48	19.50
-1	-11	1	152.78	19.90
1	-11	-1	202.18	19.10
-1	11	1	199.88	31.40
1	11	-1	172.88	14.10
1	11	-1	197.08	25.60

1	11	-1	146.99	11.90
0	-11	1	84.09	14.80
0	-11	-1	89.59	15.50
0	-11	1	81.39	13.20
0	-11	-1	77.99	12.70
0	11	1	96.29	11.70
0	11	1	108.59	22.90
0	11	-1	91.19	10.40
0	11	-1	48.10	18.10
1	-11	1	265.97	21.30
-1	-11	-1	229.58	21.00
-1	-11	-1	219.48	22.10
1	11	1	225.78	16.10
-1	11	-1	211.28	13.80
-1	11	-1	225.18	32.20
1	11	1	215.88	29.00
-2	-11	-1	28.90	10.50
2	-11	1	17.60	8.10
-2	-11	-1	26.00	11.70
-2	11	-1	23.60	6.80
2	11	1	36.00	7.50
-2	11	-1	33.40	18.00
2	11	1	19.30	6.50
2	11	1	10.50	11.90
3	-11	1	9.50	8.00
-3	-11	-1	15.50	12.80
3	-11	1	31.60	8.50
3	11	1	29.80	9.60
3	11	1	14.50	7.60
-3	11	-1	13.30	4.60
-3	11	-1	10.80	9.10

3	11	1	19.40	11.70
-3	11	-1	5.80	14.50
-4	-11	-1	28.20	12.80
-4	-11	-1	-14.50	13.50
-4	-11	-1	-2.80	6.60
4	-11	1	6.10	9.00
4	-11	1	-4.10	7.60
4	11	1	-8.20	10.50
-4	11	-1	-8.30	5.70
4	11	1	14.50	7.00
-4	11	-1	-4.70	14.40
-5	-11	-1	-4.60	8.80
5	-11	1	19.50	9.80
-5	-11	-1	8.20	16.00
-5	-11	-1	3.30	14.10
5	11	1	-1.10	11.40
-5	11	-1	14.80	8.80
5	11	1	12.70	7.40
-5	11	-1	17.00	18.80
-6	-11	-1	14.30	11.00
-6	-11	-1	39.60	18.00
-6	-11	-1	29.50	16.20
6	-11	1	10.80	10.10
6	11	1	5.10	13.00
-6	11	-1	7.70	16.60
6	11	1	-1.70	7.10
-6	11	-1	17.40	11.90
-7	-11	-1	47.80	22.70
-7	-11	-1	42.00	21.40
7	-11	1	29.60	12.20
-7	-11	-1	53.59	15.60

7	11	1	47.70	13.40
-7	11	-1	4.90	18.50
-7	11	-1	37.10	15.40
-8	-11	-1	2.70	11.70
-8	-11	-1	6.70	22.30
8	-11	1	-15.20	12.70
-8	-11	-1	21.50	22.80
-8	11	-1	-9.30	10.80
-8	11	-1	-23.50	15.70
8	11	1	6.70	10.60
-7	-12	1	21.20	24.10
-7	-12	1	11.30	23.90
7	-12	-1	-24.00	12.80
-7	-12	1	10.40	12.40
7	12	-1	11.80	10.30
-7	12	1	-24.70	27.10
-6	-12	1	10.10	12.00
-6	-12	1	-6.50	19.10
-6	-12	1	11.10	20.90
6	-12	-1	1.40	12.30
-6	12	1	-32.50	17.50
6	12	-1	5.30	9.30
-5	-12	1	30.00	19.50
-5	-12	1	36.60	17.40
-5	-12	1	37.70	13.40
5	12	-1	50.19	14.40
-5	12	1	28.40	20.90
5	12	-1	23.70	10.60
4	-12	-1	4.40	14.40
-4	-12	1	15.30	14.10
4	-12	-1	16.70	11.90

-4	-12	1	3.50	9.60
4	12	-1	11.00	7.90
4	12	-1	15.30	12.00
-4	12	1	22.20	19.60
-3	-12	1	-2.00	13.60
3	-12	-1	0.20	11.00
-3	-12	1	-2.60	8.50
3	-12	-1	-2.20	7.60
3	12	-1	-8.40	10.60
-3	12	1	12.50	7.70
3	12	-1	-6.10	6.10
-3	12	1	14.80	18.40
3	12	-1	16.30	12.40
2	-12	-1	3.40	9.70
-2	-12	1	11.80	12.60
2	-12	-1	17.60	11.40
-2	-12	1	25.10	10.70
-2	12	1	15.90	7.70
2	12	-1	36.40	12.50
2	12	-1	16.60	8.50
2	12	-1	9.90	6.60
-2	12	1	28.70	18.20
-1	-12	1	-5.60	9.80
1	-12	-1	-0.10	10.20
-1	-12	1	8.00	7.30
1	12	-1	13.00	6.20
1	12	-1	15.10	12.60
1	12	-1	8.00	8.10
-1	12	1	-3.40	14.60
1	12	-1	9.10	5.30
-1	12	1	4.30	7.10

0	-12	-1	15.80	10.20
0	-12	1	27.20	16.50
0	-12	-1	18.20	11.40
0	-12	1	14.90	10.50
0	12	1	23.50	8.00
0	12	1	33.10	14.70
0	12	-1	20.50	13.90
1	-12	1	-7.90	8.20
-1	-12	-1	21.30	9.20
1	-12	1	19.40	8.90
-1	12	-1	7.00	6.10
-1	12	-1	35.60	16.80
1	12	1	25.90	14.30
1	12	1	19.20	7.20
2	-12	1	23.10	8.70
-2	-12	-1	7.90	13.40
2	-12	1	2.70	9.10
-2	-12	-1	26.20	13.10
-2	12	-1	3.90	14.10
2	12	1	19.70	14.40
-2	12	-1	12.00	6.30
2	12	1	7.00	7.30
3	-12	1	24.90	10.70
3	-12	1	33.50	10.50
-3	-12	-1	36.60	13.30
-3	12	-1	49.90	22.60
-3	12	-1	27.30	7.40
3	12	1	16.60	10.30
3	12	1	24.90	13.80
3	12	1	38.90	9.90
4	-12	1	15.70	10.50

-4	-12	-1	22.70	12.90
-4	-12	-1	1.50	14.60
-4	-12	-1	36.10	12.00
-4	12	-1	25.30	16.80
4	12	1	57.69	11.80
4	12	1	21.50	13.90
5	-12	1	-2.40	9.70
-5	-12	-1	-5.60	8.10
-5	-12	-1	10.30	13.90
-5	-12	-1	19.00	17.80
5	12	1	20.10	8.80
5	12	1	-3.70	12.70
-5	12	-1	-3.80	19.60
-5	12	-1	11.90	9.20
-6	-12	-1	-1.90	20.20
-6	-12	-1	-4.30	20.10
6	-12	1	-7.00	10.20
-6	-12	-1	-2.80	10.10
-6	12	-1	17.60	23.40
6	12	1	22.70	11.40
-6	12	-1	3.70	10.50
-7	-12	-1	31.70	22.60
7	-12	1	6.00	13.60
-7	-12	-1	-8.90	10.30
-7	-12	-1	20.00	24.60
-7	12	-1	26.10	15.70
7	12	1	16.60	13.10
-7	12	-1	23.90	25.80
-6	-13	1	6.90	22.50
6	-13	-1	0.70	14.30
6	13	-1	2.30	15.80

-6	13	1	15.70	24.30
6	13	-1	-3.90	8.60
5	-13	-1	10.90	13.00
-5	-13	1	-2.70	10.10
-5	-13	1	11.70	19.70
5	13	-1	21.00	14.60
-5	13	1	-0.90	23.50
5	13	-1	-16.70	9.00
-4	-13	1	6.20	18.30
4	-13	-1	16.80	12.40
-4	-13	1	-9.90	9.30
4	13	-1	18.90	13.90
-4	13	1	10.20	17.90
-4	13	1	8.70	7.80
4	13	-1	5.10	7.70
-3	-13	1	-10.60	10.20
3	-13	-1	12.90	19.00
-3	-13	1	2.90	15.40
3	-13	-1	-9.20	9.40
-3	13	1	19.50	19.00
3	13	-1	-0.70	7.90
3	13	-1	11.40	13.00
-3	13	1	-0.10	8.40
-2	-13	1	9.50	9.10
2	-13	-1	15.70	13.70
2	-13	-1	-3.80	8.10
-2	13	1	7.20	20.20
2	13	-1	-1.10	6.50
-2	13	1	-3.10	7.00
2	13	-1	16.60	12.80
-1	-13	1	20.90	11.30

1	-13	-1	13.40	10.90
-1	-13	1	26.50	15.80
1	-13	-1	31.80	14.60
-1	13	1	22.10	17.80
1	13	-1	41.00	16.20
1	13	-1	28.00	8.10
-1	13	1	37.80	9.40
0	-13	-1	-8.50	8.60
0	-13	1	-7.90	11.90
0	-13	1	-3.30	9.10
0	-13	-1	-1.40	11.20
0	13	1	-0.70	13.90
0	13	-1	14.80	14.90
0	13	-1	-3.30	6.10
0	13	1	0.30	7.80
1	-13	1	-12.20	10.40
-1	-13	-1	9.00	10.00
1	-13	1	8.60	9.70
-1	-13	-1	6.00	14.20
-1	13	-1	0.50	15.60
1	13	1	17.30	7.70
-1	13	-1	15.40	7.30
1	13	1	8.60	15.30
-2	-13	-1	-0.90	10.80
-2	-13	-1	-27.90	15.50
2	-13	1	14.20	10.00
-2	13	-1	-32.70	15.80
2	13	1	25.20	15.40
2	13	1	-6.30	7.10
-2	13	-1	22.20	7.70
-3	-13	-1	22.30	19.20

3	-13	1	20.80	10.90
-3	-13	-1	6.70	12.20
3	13	1	7.60	8.50
-3	13	-1	8.50	19.20
3	13	1	11.90	14.80
-3	13	-1	-0.80	7.50
-4	-13	-1	-19.10	17.20
-4	-13	-1	-6.80	8.70
4	-13	1	7.00	10.40
-4	13	-1	-36.90	19.60
4	13	1	-3.50	14.80
4	13	1	-6.80	8.10
-5	-13	-1	-0.20	18.00
-5	-13	-1	31.30	21.40
-5	-13	-1	6.40	12.30
5	-13	1	9.50	11.60
-5	13	-1	34.00	22.10
5	13	1	-15.30	14.80
5	13	1	4.80	9.40
6	-13	1	3.00	13.30
-6	-13	-1	-12.70	20.90
-6	-13	-1	4.70	11.60
-6	-13	-1	23.60	33.90
6	13	1	-1.40	16.20
-6	13	-1	-12.30	24.90
6	13	1	15.10	10.40
5	-14	-1	33.20	15.60
-5	-14	1	-2.20	14.90
-5	-14	1	38.60	26.50
5	14	-1	22.60	17.10
-5	14	1	0.00	26.60

-5	14	1	17.70	12.00
5	14	-1	-0.70	10.90
-4	-14	1	9.80	12.40
4	-14	-1	22.70	14.10
-4	-14	1	15.00	22.70
4	14	-1	-6.70	9.70
4	14	-1	29.70	16.50
-4	14	1	3.20	22.70
-4	14	1	-1.40	10.70
3	-14	-1	15.60	12.40
-3	-14	1	1.50	12.60
-3	-14	1	-3.90	21.40
-3	14	1	16.10	21.90
3	14	-1	6.20	9.40
3	14	-1	0.10	15.90
-3	14	1	17.90	10.80
-2	-14	1	-8.00	9.40
2	-14	-1	-4.50	11.10
2	14	-1	8.20	15.60
-2	14	1	11.60	19.40
2	14	-1	-6.40	8.00
-2	14	1	4.20	9.60
1	-14	-1	16.10	12.00
-1	-14	1	22.20	12.50
1	14	-1	9.80	16.30
-1	14	1	20.00	9.50
1	14	-1	14.70	8.90
-1	14	1	20.30	21.40
0	-14	-1	-18.80	13.30
0	-14	-1	11.20	11.10
0	-14	1	12.70	12.20

0	14	1	-40.40	16.10
0	14	1	-3.70	8.40
0	14	-1	-6.80	6.80
0	14	-1	-9.30	17.90
-1	-14	-1	-1.20	11.50
-1	-14	-1	2.00	16.10
1	-14	1	0.40	10.80
-1	14	-1	5.30	8.00
1	14	1	6.10	17.80
1	14	1	-5.10	7.60
2	-14	1	-7.30	9.80
-2	-14	-1	2.90	19.60
-2	-14	-1	0.80	10.80
-2	14	-1	9.90	7.50
2	14	1	6.10	8.90
-2	14	-1	20.70	19.00
2	14	1	-6.20	16.40
-3	-14	-1	27.60	24.40
3	-14	1	41.90	14.10
-3	-14	-1	25.50	13.90
3	14	1	10.50	10.70
-3	14	-1	35.40	24.40
-3	14	-1	39.00	9.90
3	14	1	63.29	21.40
4	-14	1	18.30	13.10
-4	-14	-1	-19.50	22.20
-4	-14	-1	-8.60	10.40
-4	14	-1	11.30	23.60
4	14	1	12.30	16.80
4	14	1	5.80	10.40
-4	14	-1	6.70	9.40

-5	-14	-1	-0.20	24.60
5	-14	1	18.20	15.10
-5	-14	-1	22.10	16.00
5	14	1	-35.10	17.10
5	14	1	-3.80	11.10
-5	14	-1	-27.80	25.50
4	-15	-1	-12.00	16.50
-4	-15	1	-5.60	15.40
4	15	-1	-3.60	12.30
-4	15	1	-11.50	25.30
-4	15	1	17.30	12.90
-3	-15	1	-4.30	12.70
3	-15	-1	2.10	13.50
3	15	-1	4.80	20.80
3	15	-1	-11.10	9.90
-3	15	1	53.79	26.30
-3	15	1	20.90	11.30
2	-15	-1	-10.40	13.30
-2	15	1	10.00	11.50
2	15	-1	-11.20	19.80
2	15	-1	-6.60	9.60
-2	15	1	0.20	22.60
-1	-15	1	27.20	14.60
1	-15	-1	35.20	14.90
-1	15	1	12.10	21.20
1	15	-1	-25.40	19.50
1	15	-1	-3.10	9.50
-1	15	1	28.70	11.10
0	-15	1	5.60	14.70
0	-15	-1	-2.30	14.10
0	15	-1	3.60	21.30

0	15	1	2.70	22.70
0	15	1	13.60	12.70
0	15	-1	1.00	8.60
1	-15	1	17.40	14.30
-1	-15	-1	25.00	13.20
-1	15	-1	49.40	24.30
-1	15	-1	18.00	9.90
1	15	1	-13.70	20.00
1	15	1	0.10	10.30
-2	-15	-1	-10.60	19.10
2	-15	1	4.00	14.40
2	15	1	-4.60	10.50
2	15	1	13.60	22.10
-2	15	-1	13.60	10.10
-2	15	-1	34.40	26.20
-3	-15	-1	-8.50	14.40
3	-15	1	0.90	15.00
-3	15	-1	22.00	10.90
3	15	1	4.10	10.90
-3	15	-1	-32.20	22.50
3	15	1	20.00	20.90
-4	-15	-1	6.30	13.80
4	-15	1	7.90	14.90
4	15	1	10.10	12.50
-4	15	-1	-23.80	24.80
4	15	1	34.40	21.50
-11	0	2	-13.40	16.00
-11	0	2	16.90	18.40
11	0	-2	1.10	10.10
-11	0	2	27.50	21.30
11	0	-2	26.30	13.80

10	0	-2	2.20	7.40
-10	0	2	18.20	18.20
-10	0	2	-3.30	15.10
-10	0	2	2.70	14.50
10	0	-2	-12.40	10.50
-9	0	2	5.90	13.20
9	0	-2	-6.90	5.70
-9	0	2	-13.10	13.10
-8	0	2	208.28	30.80
8	0	-2	206.38	15.20
-8	0	2	189.98	26.40
-7	0	2	1.10	9.40
-7	0	2	-7.50	7.80
7	0	-2	-1.60	4.40
6	0	-2	388.26	18.50
-6	0	2	387.26	27.80
-6	0	2	424.56	28.40
5	0	-2	-0.30	3.00
-5	0	2	4.10	6.30
-5	0	2	1.80	4.90
4	0	-2	2001.90	50.99
-4	0	2	2029.60	63.79
-4	0	2	2005.60	51.09
4	0	-2	1998.30	61.69
-4	0	2	2050.69	62.09
4	0	-2	2007.70	63.99
3	0	-2	4.60	4.00
-3	0	2	2.60	3.50
3	0	-2	-0.10	4.30
3	0	-2	-2.40	1.50
-3	0	2	-1.40	2.90

-3	0	2	1.40	1.70
2	0	-2	1481.65	36.60
-2	0	2	1508.95	43.50
-2	0	2	1492.45	36.50
-2	0	2	1515.75	41.50
2	0	-2	1529.55	43.50
2	0	-2	1458.75	44.70
-2	0	2	1543.85	45.30
-1	0	2	2.40	1.90
1	0	-2	-1.10	0.70
-1	0	2	-0.10	1.00
1	0	-2	-1.40	1.90
-1	0	2	0.60	1.80
-1	0	2	0.90	1.40
1	0	-2	-0.20	2.40
1	0	-2	0.50	1.50
0	0	-2	357.76	11.90
0	0	-2	379.86	14.30
0	0	2	361.36	14.20
0	0	2	367.46	11.90
0	0	2	353.26	10.20
0	0	-2	362.86	10.30
0	0	-2	369.96	10.80
-1	0	-2	1.80	1.30
1	0	2	0.00	0.90
1	0	2	1.20	1.70
1	0	2	0.00	1.30
-1	0	-2	0.00	1.30
-1	0	-2	0.20	1.70
1	0	2	-1.90	1.30
-1	0	-2	0.50	0.80

-1	0	-2	-0.90	1.50
-1	0	-2	-0.30	0.90
1	0	2	1.30	0.80
1	0	2	0.00	0.90
-1	0	-2	-1.00	0.80
-2	0	-2	55.39	2.60
2	0	2	57.69	3.80
2	0	2	48.00	6.50
2	0	2	55.19	4.20
-2	0	-2	54.89	7.00
2	0	2	51.09	7.60
-2	0	-2	58.19	6.30
-2	0	-2	54.09	3.70
2	0	2	55.09	3.00
-2	0	-2	59.39	8.20
3	0	2	1.40	1.00
-3	0	-2	-1.40	3.00
-3	0	-2	2.90	3.60
-3	0	-2	-1.60	3.10
3	0	2	1.20	4.10
3	0	2	-3.70	2.90
3	0	2	1.60	1.70
3	0	2	-0.70	1.50
-3	0	-2	0.70	1.60
4	0	2	1518.75	39.30
-4	0	-2	1568.44	52.39
4	0	2	1532.45	52.59
-4	0	-2	1541.15	48.40
4	0	2	1486.15	47.80
4	0	2	1586.24	35.80
-4	0	-2	1594.94	43.10

5	0	2	-0.60	3.40
5	0	2	4.40	2.80
5	0	2	-2.00	1.80
-5	0	-2	1.40	4.90
-5	0	-2	-5.50	4.70
6	0	2	24.60	5.90
-6	0	-2	8.50	7.20
-6	0	-2	23.80	9.30
6	0	2	19.90	3.90
-7	0	-2	2.00	8.60
7	0	2	-7.90	3.70
-7	0	-2	-4.20	7.10
7	0	2	-6.90	3.10
8	0	2	515.95	23.10
-8	0	-2	456.85	37.10
8	0	2	509.85	18.10
-8	0	-2	523.15	35.60
9	0	2	0.10	5.10
9	0	2	13.70	5.70
-9	0	-2	-7.00	10.90
9	0	2	-3.80	5.50
-9	0	-2	4.30	12.90
10	0	2	14.20	7.30
-10	0	-2	-6.80	14.00
-10	0	-2	-0.40	13.70
-11	0	-2	-3.60	19.00
-11	0	-2	3.30	18.60
11	0	2	12.30	9.60
-11	-1	2	11.20	17.40
-11	-1	2	-6.30	19.00
11	-1	-2	10.20	13.70

-11	-1	2	17.40	19.20
11	-1	-2	14.00	11.00
11	1	-2	-2.10	9.50
-11	1	2	12.80	19.60
11	1	-2	19.90	14.20
-11	1	2	0.70	17.00
-11	1	2	-7.10	18.10
-10	-1	2	28.50	18.00
10	-1	-2	28.10	10.60
-10	-1	2	17.30	16.40
10	-1	-2	35.40	13.50
-10	1	2	14.90	16.20
10	1	-2	30.10	9.30
-10	1	2	58.49	21.90
-10	1	2	42.20	17.40
-9	-1	2	10.70	11.90
-9	-1	2	4.90	13.10
9	-1	-2	29.00	10.20
9	-1	-2	18.00	6.80
-9	1	2	4.30	12.40
9	1	-2	10.90	7.10
-9	1	2	-2.50	11.00
-9	1	2	15.50	15.40
-8	-1	2	-5.20	10.20
8	-1	-2	8.90	6.60
-8	-1	2	-14.80	11.40
8	-1	-2	-0.70	5.00
-8	1	2	10.40	9.90
-8	1	2	-12.00	7.90
-8	1	2	13.00	10.70
8	1	-2	6.20	6.40

−7	−1	2	182.28	26.50
7	−1	−2	205.38	14.40
−7	−1	2	156.98	21.90
−7	1	2	185.38	24.40
−7	1	2	131.59	19.90
7	1	−2	196.98	14.00
−7	1	2	212.78	22.30
6	−1	−2	410.66	18.70
−6	−1	2	372.36	28.00
−6	−1	2	439.16	28.40
6	1	−2	395.76	18.70
−6	1	2	399.46	27.50
−6	1	2	375.96	26.40
−6	1	2	412.76	28.40
6	1	−2	366.66	18.90
5	−1	−2	212.28	11.90
−5	−1	2	222.48	20.10
−5	−1	2	201.68	18.60
−5	1	2	222.68	19.10
5	1	−2	237.68	12.90
−5	1	2	188.88	18.10
5	1	−2	198.88	13.50
−5	1	2	241.18	19.20
−4	−1	2	234.58	17.30
−4	−1	2	209.48	11.00
−4	−1	2	198.28	17.40
4	−1	−2	217.58	17.20
4	−1	−2	195.88	10.70
−4	1	2	194.18	15.50
4	1	−2	207.08	17.70
4	1	−2	192.88	10.70

−4	1	2	207.58	11.00
4	1	−2	184.68	16.80
−4	1	2	180.58	15.50
−4	1	2	220.08	16.60
−3	−1	2	8.60	4.30
3	−1	−2	−0.80	4.20
3	−1	−2	5.80	2.50
3	−1	−2	3.60	3.80
−3	−1	2	4.90	2.50
−3	−1	2	3.20	3.80
−3	1	2	7.40	3.50
3	1	−2	3.60	2.20
−3	1	2	2.90	2.60
−3	1	2	0.90	3.30
−3	1	2	3.20	3.70
3	1	−2	−0.10	3.50
3	1	−2	6.20	4.40
−2	−1	2	12.00	2.50
2	−1	−2	4.60	3.30
2	−1	−2	6.50	2.30
2	−1	−2	8.90	4.00
−2	−1	2	12.60	3.50
2	−1	−2	5.20	3.60
−2	−1	2	7.60	3.60
−2	1	2	8.00	3.10
2	1	−2	3.90	3.70
−2	1	2	9.80	4.30
−2	1	2	6.60	2.30
2	1	−2	7.00	2.10
2	1	−2	11.60	3.70
−2	1	2	5.00	2.90

-1	-1	2	265.17	14.00
1	-1	-2	245.18	13.40
1	-1	-2	246.48	9.60
1	-1	-2	269.97	13.70
1	-1	-2	281.07	11.80
1	-1	-2	250.67	13.50
-1	-1	2	268.77	8.50
-1	-1	2	260.87	11.90
-1	1	2	250.17	12.30
1	1	-2	258.07	8.60
-1	1	2	250.07	13.30
-1	1	2	274.87	9.90
1	1	-2	260.37	13.80
-1	1	2	266.37	14.00
1	1	-2	289.67	12.10
0	-1	2	1768.52	37.70
0	-1	2	1828.82	37.90
0	-1	-2	1751.32	40.90
0	-1	-2	1740.23	45.10
0	-1	-2	1822.72	49.30
0	-1	-2	1691.73	43.10
0	1	2	1744.33	44.90
0	1	2	1806.12	41.00
0	1	2	1821.22	46.70
0	1	-2	1768.32	41.90
0	1	-2	1807.42	38.30
0	1	2	1714.43	43.20
0	1	-2	1758.52	39.20
0	1	2	1728.83	40.40
-1	-1	-2	420.86	16.10
1	-1	2	413.16	13.00

1	-1	2	420.86	9.80
1	-1	2	427.86	10.20
-1	-1	-2	447.66	13.40
-1	-1	-2	449.56	13.00
1	-1	2	415.96	16.70
-1	-1	-2	445.36	16.60
-1	-1	-2	443.86	16.60
-1	-1	-2	424.46	16.40
-1	-1	-2	413.76	12.30
-1	1	-2	426.16	11.10
-1	1	-2	453.75	17.50
1	1	2	411.36	12.40
1	1	2	432.56	12.80
1	1	2	430.46	14.80
-1	1	-2	433.26	13.70
1	1	2	406.96	15.90
-1	1	-2	448.56	13.10
-1	1	-2	428.16	10.40
1	1	2	422.66	16.40
1	1	2	417.66	16.00
-2	-1	-2	2992.80	67.09
-2	-1	-2	3024.90	66.59
-2	-1	-2	3017.80	75.19
2	-1	2	2944.61	64.09
2	-1	2	2946.21	75.29
-2	-1	-2	3200.68	74.79
2	-1	2	2919.11	64.39
2	-1	2	2958.20	69.39
-2	-1	-2	3001.40	65.79
-2	-1	-2	2937.41	73.39
-2	1	-2	3082.39	75.79

-2	1	-2	2924.71	69.29
2	1	2	2949.60	66.49
2	1	2	3028.30	73.59
2	1	2	2962.20	74.19
-2	1	-2	2958.10	64.69
2	1	2	3002.20	66.99
2	1	2	3012.30	74.99
-2	1	-2	2982.40	64.99
-3	-1	-2	56.79	8.30
3	-1	2	42.70	3.70
-3	-1	-2	65.99	10.00
3	-1	2	40.30	7.00
3	-1	2	56.29	4.30
-3	-1	-2	50.19	4.50
-3	-1	-2	47.30	3.90
-3	-1	-2	41.70	7.70
3	-1	2	51.59	8.90
3	1	2	48.30	4.50
3	1	2	44.80	3.90
-3	1	-2	53.59	7.50
3	1	2	44.40	8.00
3	1	2	51.49	9.20
-3	1	-2	55.29	8.80
3	1	2	48.90	4.40
-3	1	-2	48.70	4.20
-4	-1	-2	1613.44	50.99
-4	-1	-2	1574.64	52.79
-4	-1	-2	1576.04	51.19
-4	-1	-2	1460.95	39.90
4	-1	2	1559.44	39.00
-4	-1	-2	1563.04	41.10

4	-1	2	1551.64	47.80
4	-1	2	1561.74	39.20
4	-1	2	1655.23	54.09
4	1	2	1607.64	41.20
-4	1	-2	1616.94	40.30
4	1	2	1506.75	49.90
4	1	2	1625.44	38.80
-4	1	-2	1619.34	48.30
-4	1	-2	1640.34	53.59
4	1	2	1556.94	52.99
4	1	2	1580.84	40.90
5	-1	2	120.79	5.40
5	-1	2	123.39	8.20
5	-1	2	114.59	8.10
-5	-1	-2	105.39	16.30
5	-1	2	118.89	6.30
-5	-1	-2	153.88	15.40
5	1	2	119.49	7.60
-5	1	-2	150.68	9.80
5	1	2	144.49	9.70
5	1	2	139.49	9.50
-5	1	-2	151.08	15.90
-5	1	-2	140.99	14.10
-6	-1	-2	375.06	27.60
6	-1	2	353.86	16.20
6	-1	2	373.36	16.10
-6	-1	-2	356.06	25.30
6	-1	2	351.86	13.20
-6	1	-2	360.46	24.20
6	1	2	356.06	14.70
6	1	2	406.66	17.70

−6	1	−2	351.66	26.50
7	−1	2	102.99	7.80
7	−1	2	120.39	10.20
−7	−1	−2	123.69	21.20
−7	−1	−2	70.09	14.30
7	−1	2	109.09	6.70
7	1	2	102.89	11.30
7	1	2	119.79	9.00
−7	1	−2	99.69	18.70
−7	1	−2	57.19	12.80
8	−1	2	−2.90	4.70
−8	−1	−2	4.60	11.00
−8	−1	−2	9.70	10.90
8	−1	2	10.60	3.60
8	−1	2	3.00	5.50
8	1	2	9.80	6.40
8	1	2	9.90	5.20
−8	1	−2	5.30	11.30
−8	1	−2	6.30	10.20
9	−1	2	1.10	4.30
9	−1	2	−8.20	5.50
9	−1	2	7.10	6.20
−9	−1	−2	−8.90	10.50
−9	−1	−2	5.10	13.10
9	1	2	4.30	6.60
−9	1	−2	31.90	16.20
−9	1	−2	4.00	11.10
9	1	2	5.20	6.90
−10	−1	−2	−0.60	14.70
10	−1	2	−3.50	7.20
−10	−1	−2	−3.80	12.70

-10	1	-2	-4.50	16.40
-10	1	-2	4.70	13.70
10	1	2	3.40	8.30
-11	-1	-2	18.20	22.00
11	-1	2	2.30	9.70
-11	-1	-2	4.40	20.80
-11	1	-2	12.20	18.60
11	1	2	14.50	11.00
-11	1	-2	5.00	16.90
11	-2	-2	-17.00	14.80
-11	-2	2	3.10	34.90
-11	-2	2	-7.40	19.20
11	-2	-2	18.60	10.30
11	2	-2	-4.40	10.10
-11	2	2	-8.80	16.00
-11	2	2	3.30	15.90
-11	2	2	12.60	20.40
10	-2	-2	18.10	9.40
-10	-2	2	32.00	18.60
10	-2	-2	47.40	12.40
-10	-2	2	36.80	17.40
10	2	-2	19.20	10.10
-10	2	2	-4.50	15.00
-10	2	2	15.80	17.60
-10	2	2	38.60	17.70
9	-2	-2	6.10	7.30
-9	-2	2	19.30	14.80
9	-2	-2	15.00	9.70
-9	-2	2	5.60	13.10
-9	2	2	-1.70	10.90
-9	2	2	16.90	15.70

9	2	−2	6.10	6.70
−9	2	2	1.70	15.70
8	−2	−2	23.20	7.80
−8	−2	2	14.10	12.50
8	−2	−2	36.60	9.60
−8	−2	2	28.30	11.10
−8	2	2	36.10	15.30
8	2	−2	35.30	8.10
−8	2	2	39.00	16.40
−8	2	2	30.60	13.50
−7	−2	2	3.60	10.10
7	−2	−2	3.30	4.20
−7	−2	2	13.80	8.90
7	−2	−2	9.90	6.70
7	2	−2	0.50	4.90
−7	2	2	2.20	8.70
−7	2	2	−2.60	8.20
−7	2	2	−5.00	7.60
−6	−2	2	196.08	23.10
−6	−2	2	195.88	20.80
6	−2	−2	179.98	14.80
−6	2	2	196.78	20.00
−6	2	2	207.68	21.10
6	2	−2	179.08	12.50
−6	2	2	197.48	18.90
5	−2	−2	61.99	7.80
5	−2	−2	110.59	19.60
−5	−2	2	79.79	14.20
−5	−2	2	81.69	14.30
−5	2	2	84.39	13.60
−5	2	2	64.39	11.70

5	2	−2	93.99	9.70
−5	2	2	79.39	13.30
5	2	−2	95.59	8.70
4	−2	−2	62.89	11.50
−4	−2	2	79.89	8.10
4	−2	−2	78.89	9.40
4	−2	−2	70.19	6.60
−4	−2	2	91.09	14.30
−4	−2	2	89.19	13.40
4	−2	−2	53.09	10.20
−4	2	2	102.09	14.70
−4	2	2	77.09	11.50
−4	2	2	81.49	8.40
4	2	−2	77.49	9.10
4	2	−2	83.49	15.00
−4	2	2	66.09	11.40
4	2	−2	83.19	7.00
3	−2	−2	655.13	20.80
3	−2	−2	657.33	28.20
3	−2	−2	616.34	26.50
−3	−2	2	676.43	30.90
−3	−2	2	665.63	27.30
−3	−2	2	617.84	19.90
3	−2	−2	651.43	24.50
3	2	−2	656.63	22.70
−3	2	2	649.14	29.40
3	2	−2	633.84	20.40
3	2	−2	690.33	30.40
−3	2	2	639.84	21.00
−3	2	2	677.63	27.00
3	2	−2	641.94	27.30

2	−2	−2	7214.28	157.48
−2	−2	2	6998.10	149.59
2	−2	−2	7007.80	163.88
2	−2	−2	6819.42	157.08
−2	−2	2	6825.62	162.88
−2	−2	2	7109.89	158.38
2	−2	−2	7054.49	161.08
−2	−2	2	7113.89	150.58
2	−2	−2	7291.37	151.78
−2	2	2	6894.81	157.88
2	2	−2	7026.10	157.88
−2	2	2	7253.07	164.98
2	2	−2	7254.97	153.98
−2	2	2	7205.88	151.98
2	2	−2	6803.22	149.88
−2	2	2	6967.80	160.68
2	2	−2	6923.61	162.28
−2	2	2	6994.10	156.28
1	−2	−2	237.68	11.40
1	−2	−2	235.18	12.00
1	−2	−2	264.57	16.10
−1	−2	2	267.07	7.30
1	−2	−2	270.77	10.50
1	−2	−2	257.27	14.80
−1	−2	2	259.37	11.70
−1	−2	2	254.07	16.50
1	2	−2	273.97	11.00
1	2	−2	249.97	11.40
1	2	−2	262.67	8.50
−1	2	2	294.07	13.20
−1	2	2	280.37	16.50

1	2	-2	258.47	14.50
-1	2	2	260.87	11.60
-1	2	2	264.97	10.40
-1	2	2	263.47	14.70
0	-2	-2	568.64	22.10
0	-2	-2	587.54	21.10
0	-2	2	546.15	16.50
0	-2	-2	547.55	21.10
0	2	2	563.94	18.60
0	2	-2	562.14	15.90
0	2	2	563.64	23.50
0	2	2	577.14	22.60
0	2	2	558.44	16.30
0	2	-2	567.04	15.30
0	2	-2	551.54	12.70
0	2	-2	579.34	16.80
-1	-2	-2	238.28	11.50
-1	-2	-2	256.67	13.30
-1	-2	-2	253.57	9.30
1	-2	2	248.48	8.50
-1	-2	-2	261.97	13.40
-1	-2	-2	278.97	13.50
-1	-2	-2	254.37	9.60
1	2	2	257.07	13.10
1	2	2	259.17	9.60
-1	2	-2	235.78	14.40
-1	2	-2	250.17	7.60
1	2	2	247.58	10.40
-1	2	-2	264.57	6.90
1	2	2	246.58	12.90
1	2	2	235.58	11.30

1	2	2	251.87	9.50
-1	2	-2	255.57	8.80
-2	-2	-2	3000.80	70.19
-2	-2	-2	3197.58	69.79
2	-2	2	3093.79	69.79
-2	-2	-2	3122.29	77.99
-2	-2	-2	3096.39	70.29
2	-2	2	3099.79	78.09
-2	-2	-2	3028.10	76.89
-2	-2	-2	3019.20	77.09
2	-2	2	3036.80	65.99
2	2	2	3005.80	77.19
-2	2	-2	3091.09	65.79
-2	2	-2	3118.89	66.19
2	2	2	3104.09	69.99
2	2	2	3071.89	76.89
-2	2	-2	3088.29	69.49
-2	2	-2	3088.39	65.89
2	2	2	3068.89	69.69
2	2	2	3145.59	70.59
-2	2	-2	3154.58	77.89
2	2	2	3146.59	78.19
-3	-2	-2	455.05	16.10
3	-2	2	471.95	23.00
3	-2	2	487.05	19.00
3	-2	2	483.75	13.20
-3	-2	-2	448.36	22.40
-3	-2	-2	472.55	22.00
-3	-2	-2	472.35	26.50
3	-2	2	471.05	13.00
-3	-2	-2	485.65	16.40

−3	2	−2	509.35	23.60
3	2	2	480.75	16.70
3	2	2	479.95	23.00
−3	2	−2	493.95	14.60
3	2	2	489.45	22.80
3	2	2	495.05	16.50
−3	2	−2	487.05	18.80
3	2	2	485.35	22.90
−4	−2	−2	67.19	10.60
−4	−2	−2	54.69	5.60
−4	−2	−2	57.69	10.30
4	−2	2	58.09	8.90
−4	−2	−2	45.40	9.10
4	−2	2	50.89	4.60
4	−2	2	57.39	4.70
−4	−2	−2	60.79	7.10
4	−2	2	53.79	11.70
−4	2	−2	61.59	5.50
4	2	2	53.29	5.60
−4	2	−2	60.39	10.20
4	2	2	61.99	11.00
−4	2	−2	51.49	8.20
4	2	2	52.09	10.20
4	2	2	63.89	7.40
4	2	2	47.00	6.20
5	−2	2	34.10	2.70
5	−2	2	24.00	4.40
−5	−2	−2	36.30	12.50
−5	−2	−2	32.30	5.00
5	−2	2	33.60	2.70
−5	−2	−2	37.80	9.80

5	−2	2	39.20	5.20
5	2	2	39.00	6.20
−5	2	−2	42.40	6.40
−5	2	−2	20.70	7.80
5	2	2	38.40	5.80
−5	2	−2	40.70	8.50
6	−2	2	31.20	3.20
6	−2	2	33.10	3.40
6	−2	2	24.30	5.00
6	−2	2	27.80	5.60
−6	−2	−2	28.90	10.20
−6	−2	−2	37.00	10.20
6	2	2	36.90	6.90
6	2	2	30.10	5.40
−6	2	−2	30.30	9.70
−6	2	−2	22.70	8.90
−7	−2	−2	3.70	8.40
7	−2	2	1.10	2.40
−7	−2	−2	1.60	9.00
7	−2	2	−2.50	3.30
7	−2	2	−0.90	2.20
7	−2	2	5.10	5.10
−7	2	−2	6.10	8.20
−7	2	−2	0.90	6.30
7	2	2	2.50	4.40
7	2	2	2.30	5.10
8	−2	2	179.88	12.90
−8	−2	−2	149.99	23.70
−8	−2	−2	175.08	27.50
8	2	2	179.98	13.70
8	2	2	161.48	14.60

−8	2	−2	148.89	24.00
−8	2	−2	193.18	25.70
−9	−2	−2	−4.40	13.20
−9	−2	−2	9.10	14.90
9	−2	2	4.30	6.00
−9	2	−2	21.90	16.10
9	2	2	7.20	10.80
9	2	2	3.50	8.00
−9	2	−2	2.40	12.30
−10	−2	−2	33.90	19.10
−10	−2	−2	24.80	19.50
10	−2	2	37.30	9.20
−10	2	−2	16.20	14.90
10	2	2	35.70	10.30
−10	2	−2	37.40	17.00
11	−2	2	−4.60	9.90
−11	2	−2	1.90	19.70
−11	2	−2	8.00	15.60
11	2	2	12.70	11.50
−11	−3	2	5.90	21.90
11	−3	−2	11.60	14.30
11	−3	−2	4.50	11.40
−11	3	2	23.70	20.10
11	3	−2	30.80	12.10
−11	3	2	−15.50	21.20
−10	−3	2	44.00	21.10
10	−3	−2	25.90	9.00
−10	−3	2	38.20	18.50
10	−3	−2	31.10	11.80
−10	3	2	33.50	16.50
−10	3	2	50.59	20.30

-10	3	2	29.90	19.50
10	3	-2	16.40	10.10
9	-3	-2	-6.00	6.10
-9	-3	2	5.40	13.60
-9	-3	2	-13.60	13.00
9	-3	-2	-2.00	9.10
-9	3	2	-7.10	13.60
9	3	-2	-5.90	7.20
-9	3	2	25.60	14.30
-9	3	2	15.40	14.30
8	-3	-2	-2.90	7.30
8	-3	-2	1.70	5.40
-8	-3	2	-5.30	9.90
-8	-3	2	7.20	11.40
-8	3	2	3.00	9.90
-8	3	2	6.30	12.70
8	3	-2	-2.70	5.30
-8	3	2	1.70	12.30
7	-3	-2	5.90	6.50
-7	-3	2	-0.80	10.00
7	-3	-2	11.00	6.30
-7	-3	2	8.30	10.10
-7	3	2	15.80	9.80
-7	3	2	29.70	12.70
-7	3	2	17.00	10.80
7	3	-2	16.50	5.80
6	-3	-2	420.86	19.30
6	-3	-2	449.56	23.50
-6	-3	2	450.85	31.30
-6	-3	2	367.56	28.10
6	3	-2	402.66	19.70

−6	3	2	430.16	30.80
−6	3	2	388.96	26.90
−6	3	2	415.66	28.30
−5	−3	2	−1.20	5.60
−5	−3	2	−6.80	5.30
5	−3	−2	2.10	4.90
5	−3	−2	−1.80	3.40
−5	−3	2	−10.50	5.10
5	−3	−2	7.60	8.50
−5	3	2	7.40	6.50
5	3	−2	1.50	3.60
5	3	−2	−5.80	3.70
−5	3	2	3.60	4.60
−4	−3	2	136.79	11.30
4	−3	−2	144.99	11.00
−4	−3	2	164.88	16.70
−4	−3	2	180.68	16.00
4	−3	−2	150.18	14.30
−4	−3	2	136.89	9.30
4	−3	−2	147.49	17.40
4	−3	−2	132.69	11.40
−4	3	2	149.79	14.50
4	3	−2	138.69	9.30
−4	3	2	132.39	18.30
4	3	−2	143.89	10.80
4	3	−2	141.59	15.50
−4	3	2	156.38	16.10
−4	3	2	163.98	10.40
3	−3	−2	21.10	6.40
3	−3	−2	30.10	5.00
3	−3	−2	24.30	9.40

3	-3	-2	35.10	8.30
-3	-3	2	32.40	4.60
3	-3	-2	35.50	6.40
-3	-3	2	24.60	5.00
-3	-3	2	39.00	8.10
-3	3	2	46.40	6.30
3	3	-2	27.00	4.90
-3	3	2	42.60	9.40
-3	3	2	17.50	6.10
3	3	-2	22.10	6.50
3	3	-2	31.00	8.00
-3	3	2	26.60	6.20
-3	3	2	43.00	7.30
-2	-3	2	2.80	2.40
-2	-3	2	-1.20	3.00
2	-3	-2	2.10	2.70
-2	-3	2	2.30	1.60
-2	-3	2	3.10	3.40
2	-3	-2	2.00	4.50
2	-3	-2	1.50	2.50
2	-3	-2	3.30	2.60
2	-3	-2	18.80	7.00
2	3	-2	6.70	2.40
-2	3	2	2.80	3.30
2	3	-2	-2.10	2.90
-2	3	2	8.80	3.80
-2	3	2	4.40	5.40
2	3	-2	6.80	4.20
-2	3	2	2.40	3.50
2	3	-2	4.10	2.90
-2	3	2	2.30	2.50

1	−3	−2	18.50	3.30
−1	−3	2	23.40	3.60
1	−3	−2	6.20	4.90
−1	−3	2	14.00	3.50
−1	−3	2	17.10	3.70
−1	−3	2	15.70	3.40
1	−3	−2	9.80	3.10
1	−3	−2	18.40	5.60
1	−3	−2	20.10	3.70
−1	3	2	16.70	4.30
1	3	−2	16.80	2.20
−1	3	2	20.10	6.30
1	3	−2	18.20	3.70
−1	3	2	20.00	5.70
−1	3	2	23.60	3.80
1	3	−2	24.50	6.10
1	3	−2	15.70	2.40
1	3	−2	15.80	3.40
0	−3	−2	1772.82	41.80
0	−3	2	1680.73	43.20
0	−3	−2	1731.43	49.10
0	−3	−2	1722.43	45.80
0	−3	−2	1795.52	41.40
0	−3	2	1766.92	43.70
0	−3	−2	1729.63	52.59
0	3	2	1758.92	50.39
0	3	2	1749.43	41.90
0	3	−2	1794.62	45.30
0	3	2	1701.63	52.29
0	3	2	1784.12	45.80
0	3	−2	1775.92	43.60

0	3	2	1777.02	42.80
0	3	-2	1783.52	40.10
0	3	-2	1787.82	37.30
-1	-3	-2	1.50	2.00
-1	-3	-2	-3.20	2.20
-1	-3	-2	-1.40	1.30
-1	-3	-2	0.40	3.00
-1	-3	-2	1.10	1.90
-1	-3	-2	-5.00	2.60
-1	-3	-2	0.50	1.90
-1	3	-2	2.30	1.80
1	3	2	-1.30	1.80
1	3	2	2.40	2.20
1	3	2	2.50	2.90
1	3	2	-2.60	1.50
-1	3	-2	0.40	1.80
1	3	2	-0.60	1.40
-1	3	-2	1.80	2.10
-2	-3	-2	1802.92	49.99
2	-3	2	1809.12	42.10
2	-3	2	1827.22	51.39
-2	-3	-2	1794.72	52.49
-2	-3	-2	1803.62	43.80
-2	-3	-2	1827.12	51.89
-2	-3	-2	1741.03	45.90
2	-3	2	1783.02	41.70
2	3	2	1866.71	46.30
-2	3	-2	1839.52	42.20
-2	3	-2	1844.72	38.20
2	3	2	1722.03	44.00
2	3	2	1684.83	51.09

2	3	2	1784.22	52.19
−2	3	−2	1774.42	50.39
2	3	2	1801.12	49.90
−2	3	−2	1753.02	38.40
2	3	2	1810.02	44.30
−3	−3	−2	4.80	2.80
3	−3	2	3.20	4.20
−3	−3	−2	4.30	3.60
3	−3	2	3.60	1.60
−3	−3	−2	1.30	3.20
3	−3	2	0.10	2.90
3	3	2	5.10	4.60
−3	3	−2	−0.80	3.20
−3	3	−2	4.10	3.00
3	3	2	6.20	4.50
3	3	2	7.20	2.90
−3	3	−2	3.90	1.80
3	3	2	−0.20	2.10
−4	−3	−2	960.60	29.00
4	−3	2	960.60	23.60
4	−3	2	1008.90	33.10
−4	−3	−2	954.60	37.50
4	−3	2	990.80	23.70
4	−3	2	980.30	39.10
−4	−3	−2	964.50	39.20
−4	−3	−2	1022.70	40.30
4	−3	2	938.71	23.10
−4	−3	−2	899.01	29.10
4	3	2	915.51	37.90
−4	3	−2	980.60	32.90
4	3	2	927.81	29.30

4	3	2	912.11	38.00
4	3	2	898.61	29.20
4	3	2	942.81	37.90
−4	3	−2	1018.00	39.10
−4	3	−2	950.00	26.40
4	3	2	993.20	29.90
−5	−3	−2	−6.10	5.70
5	−3	2	−2.00	1.30
5	−3	2	0.10	2.10
−5	−3	−2	0.70	5.80
5	−3	2	2.80	2.30
−5	3	−2	2.40	3.50
5	3	2	−2.30	3.40
5	3	2	1.30	3.40
−5	3	−2	2.20	5.30
−5	3	−2	0.80	5.10
6	−3	2	370.16	11.20
−6	−3	−2	367.36	27.80
6	−3	2	365.76	14.40
6	−3	2	383.66	11.20
−6	−3	−2	395.76	28.60
6	3	2	384.96	18.30
−6	3	−2	382.86	23.90
6	3	2	358.76	17.70
−6	3	−2	360.16	26.00
7	−3	2	−1.10	4.00
−7	−3	−2	−3.20	7.90
−7	−3	−2	−2.80	10.70
7	−3	2	−2.40	4.40
−7	3	−2	−3.20	7.20
7	3	2	−2.80	5.00

7	3	2	3.00	5.80
−7	3	−2	5.60	8.10
8	−3	2	9.40	5.80
−8	−3	−2	10.20	11.50
8	−3	2	−0.30	5.50
−8	−3	−2	−8.30	9.60
8	3	2	5.20	6.70
−8	3	−2	−7.50	10.10
−8	3	−2	1.80	9.60
9	−3	2	11.00	6.50
−9	−3	−2	−0.10	13.50
−9	−3	−2	−17.00	11.50
−9	3	−2	−1.00	10.70
9	3	2	10.90	8.70
−9	3	−2	9.70	12.70
10	−3	2	1.90	7.10
−10	−3	−2	0.20	15.50
−10	−3	−2	−5.60	17.00
−10	3	−2	21.50	15.20
−10	3	−2	−2.60	14.70
10	3	2	0.30	8.60
11	−3	2	13.60	10.30
−11	−3	−2	3.40	17.00
−11	−3	−2	−4.40	19.10
−11	3	−2	24.60	17.90
11	3	2	−5.20	9.80
−11	3	−2	−0.40	25.40
−11	3	−2	−4.50	17.80
−11	−4	2	−18.00	21.50
−11	−4	2	18.90	37.70
11	−4	−2	−16.30	10.60

-11	4	2	37.40	19.20
-11	4	2	-2.50	20.40
11	4	-2	4.10	11.80
-10	-4	2	-5.70	17.00
-10	-4	2	15.30	17.90
10	-4	-2	-7.40	10.20
10	-4	-2	8.70	8.60
-10	4	2	25.60	18.20
-10	4	2	19.90	19.60
-10	4	2	-5.80	13.90
10	4	-2	6.70	10.40
-9	-4	2	7.90	17.20
9	-4	-2	16.10	8.40
9	-4	-2	10.80	9.60
-9	-4	2	-12.90	13.10
-9	4	2	2.10	12.80
9	4	-2	-0.60	7.80
-9	4	2	-19.50	11.20
-9	4	2	5.70	13.60
8	-4	-2	118.19	13.80
-8	-4	2	116.69	20.90
8	-4	-2	133.19	16.90
-8	-4	2	141.89	25.20
-8	4	2	135.69	21.70
8	4	-2	128.49	17.20
-8	4	2	120.19	22.40
-8	4	2	125.79	25.60
7	-4	-2	1.70	6.80
-7	-4	2	1.90	8.90
7	-4	-2	-9.00	5.20
-7	-4	2	7.90	10.80

−7	4	2	5.50	10.00
−7	4	2	9.80	8.70
−7	4	2	−10.40	8.50
7	4	−2	−2.00	4.50
−6	−4	2	124.69	21.90
6	−4	−2	120.09	14.90
6	−4	−2	104.49	9.90
−6	−4	2	100.99	17.80
6	4	−2	120.99	10.80
−6	4	2	83.79	17.90
−6	4	2	94.59	16.40
−6	4	2	117.09	17.50
5	−4	−2	96.49	12.30
−5	−4	2	92.69	13.20
5	−4	−2	76.09	14.90
5	−4	−2	90.39	8.60
−5	−4	2	105.09	17.80
−5	−4	2	81.39	14.70
5	4	−2	82.19	9.90
−5	4	2	108.89	15.80
−5	4	2	102.69	16.30
−5	4	2	151.88	22.60
−4	−4	2	805.22	25.80
−4	−4	2	850.91	36.60
4	−4	−2	784.82	31.10
−4	−4	2	777.82	38.50
−4	−4	2	826.92	29.90
4	−4	−2	793.22	33.40
−4	4	2	809.42	27.10
4	4	−2	792.62	29.70
4	4	−2	810.72	37.50

−4	4	2	799.02	35.80
4	4	−2	806.72	26.40
−4	4	2	801.02	35.80
−4	4	2	816.42	40.70
3	−4	−2	696.33	26.60
3	−4	−2	685.03	30.60
−3	−4	2	668.63	23.70
3	−4	−2	700.43	22.40
−3	−4	2	683.53	33.10
−3	−4	2	671.83	21.10
−3	−4	2	687.53	29.40
3	−4	−2	692.53	35.80
−3	−4	2	715.23	25.90
−3	4	2	686.73	27.40
3	4	−2	691.43	25.90
3	4	−2	643.14	28.50
−3	4	2	710.53	31.40
−3	4	2	682.13	23.20
−3	4	2	694.53	35.30
3	4	−2	656.93	31.60
3	4	−2	678.13	22.10
−3	4	2	718.83	26.90
2	−4	−2	785.02	36.60
2	−4	−2	764.72	24.70
2	−4	−2	795.72	28.40
2	−4	−2	797.62	23.20
−2	−4	2	759.82	29.60
−2	−4	2	839.52	29.70
2	−4	−2	801.82	32.20
−2	−4	2	768.22	32.90
−2	−4	2	786.82	21.50

-2	-4	2	787.22	24.50
2	4	-2	794.52	31.60
-2	4	2	784.42	28.70
2	4	-2	790.32	26.70
-2	4	2	803.72	25.70
-2	4	2	813.62	37.80
-2	4	2	748.53	23.90
2	4	-2	821.02	22.80
2	4	-2	739.63	27.90
2	4	-2	764.22	23.50
1	-4	-2	158.98	6.90
1	-4	-2	163.58	11.10
1	-4	-2	178.58	13.60
-1	-4	2	155.58	10.00
1	-4	-2	170.18	15.70
-1	-4	2	143.99	9.60
1	-4	-2	169.18	8.10
-1	-4	2	168.58	6.10
-1	4	2	161.48	10.80
1	4	-2	149.59	8.50
1	4	-2	149.29	12.20
-1	4	2	160.58	15.70
-1	4	2	159.18	8.40
-1	4	2	181.48	14.00
1	4	-2	156.78	9.80
1	4	-2	164.88	9.80
0	-4	2	16.40	4.30
0	-4	-2	22.00	7.10
0	-4	-2	19.50	5.70
0	-4	2	15.80	4.40
0	-4	-2	16.90	3.20

0	−4	−2	12.00	2.20
0	−4	−2	16.80	3.80
0	4	2	6.40	3.80
0	4	2	6.90	2.30
0	4	2	15.60	3.40
0	4	2	12.80	5.40
0	4	−2	12.30	4.60
0	4	−2	19.40	3.00
0	4	2	11.10	3.50
0	4	−2	13.50	1.60
0	4	−2	13.10	3.70
−1	−4	−2	295.87	14.30
−1	−4	−2	292.67	17.70
−1	−4	−2	313.17	12.10
−1	−4	−2	297.17	17.20
1	−4	2	310.37	13.00
−1	−4	−2	286.87	11.50
−1	−4	−2	315.37	13.00
−1	−4	−2	292.37	16.70
−1	4	−2	336.67	15.70
1	4	2	341.07	15.20
1	4	2	306.87	12.30
1	4	2	299.87	18.20
1	4	2	304.07	13.00
1	4	2	299.17	12.00
−1	4	−2	295.17	16.70
−1	4	−2	301.27	12.70
2	−4	2	244.68	8.70
−2	−4	−2	233.18	16.10
2	−4	2	252.67	11.20
−2	−4	−2	251.07	14.10

−2	−4	−2	237.78	10.70
2	−4	2	255.67	15.30
−2	−4	−2	250.27	13.00
2	4	2	262.57	14.30
2	4	2	244.78	16.30
−2	4	−2	254.57	10.90
2	4	2	227.28	15.60
2	4	2	250.17	13.20
−2	4	−2	264.17	17.10
−2	4	−2	245.88	14.50
2	4	2	232.18	11.40
−3	−4	−2	448.76	23.10
3	−4	2	445.76	16.60
3	−4	2	446.56	15.20
−3	−4	−2	483.05	25.20
−3	−4	−2	449.95	16.90
−3	−4	−2	432.16	23.30
3	−4	2	435.66	23.00
3	4	2	451.75	17.70
3	4	2	448.16	17.30
−3	4	−2	441.96	16.70
−3	4	−2	494.35	26.40
3	4	2	470.15	25.20
3	4	2	448.46	18.70
3	4	2	468.55	22.90
3	4	2	437.96	23.50
−3	4	−2	501.05	23.20
−3	4	−2	452.75	16.00
−3	4	−2	448.26	13.30
4	−4	2	870.21	37.10
−4	−4	−2	912.41	37.20

−4	−4	−2	866.71	37.40
−4	−4	−2	897.71	30.20
4	−4	2	861.91	29.90
−4	−4	−2	884.11	38.60
4	4	2	869.51	37.30
4	4	2	914.31	28.90
−4	4	−2	857.41	26.60
4	4	2	922.21	39.30
−4	4	−2	917.71	30.80
4	4	2	877.81	31.60
4	4	2	863.91	37.20
−4	4	−2	903.71	36.70
−4	4	−2	895.71	24.80
−5	−4	−2	8.20	7.90
5	−4	2	15.60	3.00
−5	−4	−2	9.90	11.60
5	−4	2	11.50	2.10
5	−4	2	14.50	2.20
−5	−4	−2	13.70	5.10
5	4	2	12.80	5.20
5	4	2	5.90	4.90
−5	4	−2	12.10	4.20
−5	4	−2	15.90	8.40
−5	4	−2	16.60	6.50
−6	−4	−2	−4.30	6.90
−6	−4	−2	0.90	3.90
6	−4	2	−1.10	2.70
6	−4	2	0.90	2.70
6	−4	2	−1.30	2.20
−6	−4	−2	−8.40	7.40
6	4	2	−10.10	4.80

−6	4	−2	1.60	6.20
−6	4	−2	−4.80	6.80
6	4	2	−1.10	3.50
7	−4	2	4.90	4.20
7	−4	2	10.40	2.70
−7	−4	−2	−10.50	10.30
−7	−4	−2	6.30	9.30
−7	4	−2	16.70	9.20
7	4	2	4.70	5.70
−7	4	−2	7.50	7.80
8	−4	2	266.77	14.00
−8	−4	−2	293.77	36.60
8	−4	2	251.17	13.60
−8	−4	−2	299.97	37.60
8	4	2	261.17	18.50
−8	4	−2	286.27	28.90
−8	4	−2	287.57	26.70
−9	−4	−2	6.00	13.60
9	−4	2	−8.10	5.90
−9	−4	−2	−14.30	13.40
−9	4	−2	11.70	11.40
−9	4	−2	0.90	12.40
−9	4	−2	−4.00	13.90
9	4	2	2.10	7.10
−10	−4	−2	5.20	16.30
−10	−4	−2	4.70	15.40
10	−4	2	0.40	7.80
−10	4	−2	2.20	12.10
−10	4	−2	−9.90	15.80
−10	4	−2	−17.60	14.10
10	4	2	−5.90	7.80

−11	−4	−2	−4.60	18.80
−11	−4	−2	10.20	19.30
−11	4	−2	19.20	16.60
−11	4	−2	−2.30	19.10
11	4	2	−6.20	10.90
−11	4	−2	−14.40	17.60
−11	−5	2	−36.50	21.20
−11	−5	2	−30.00	21.90
11	−5	−2	−20.90	11.30
−11	5	2	−35.90	16.80
11	5	−2	16.10	12.80
−11	5	2	4.60	23.90
−11	5	2	56.39	29.30
−10	−5	2	−0.20	18.10
10	−5	−2	−5.00	8.80
10	−5	−2	−2.70	11.70
−10	−5	2	10.40	18.60
−10	5	2	31.00	19.90
10	5	−2	15.20	10.20
−10	5	2	38.30	16.80
−10	5	2	25.80	20.60
9	−5	−2	−1.20	9.20
−9	−5	2	25.50	17.50
9	−5	−2	15.10	7.60
−9	−5	2	8.70	14.90
−9	5	2	24.50	15.10
9	5	−2	27.80	10.90
−9	5	2	18.40	17.70
−9	5	2	24.70	16.90
8	−5	−2	−1.30	6.10
−8	−5	2	−5.20	11.40

8	−5	−2	11.70	8.40
8	5	−2	9.00	7.90
−8	5	2	0.20	14.10
−8	5	2	6.60	14.80
−8	5	2	11.00	11.70
−7	−5	2	429.36	36.50
7	−5	−2	393.26	23.80
−7	−5	2	368.26	32.40
−7	5	2	375.76	30.50
−7	5	2	396.16	30.30
7	5	−2	417.06	22.60
−7	5	2	468.15	38.20
6	−5	−2	192.28	12.90
−6	−5	2	228.98	25.50
−6	−5	2	161.08	24.10
6	−5	−2	159.08	15.20
−6	5	2	148.69	20.10
−6	5	2	172.68	19.10
6	5	−2	211.38	15.00
−6	5	2	143.79	23.20
5	−5	−2	288.57	18.80
−5	−5	2	325.57	24.90
5	−5	−2	320.87	15.70
−5	−5	2	316.07	20.40
−5	−5	2	274.57	24.90
5	−5	−2	298.47	23.20
−5	5	2	298.47	27.20
−5	5	2	334.57	22.10
−5	5	2	303.97	23.60
5	5	−2	327.07	16.70
−4	−5	2	305.57	14.30

−4	−5	2	314.77	22.50
4	−5	−2	291.87	19.20
4	−5	−2	307.57	18.50
4	−5	−2	326.97	15.10
−4	−5	2	297.47	17.30
−4	−5	2	310.77	17.60
−4	−5	2	273.27	23.30
−4	5	2	308.67	15.90
−4	5	2	295.27	18.70
−4	5	2	312.77	19.00
4	5	−2	317.17	15.30
−4	5	2	285.87	21.50
4	5	−2	288.27	24.00
−4	5	2	296.97	25.90
3	−5	−2	79.79	10.60
−3	−5	2	90.99	13.90
−3	−5	2	86.19	6.70
−3	−5	2	70.19	9.40
3	−5	−2	89.79	14.10
3	−5	−2	90.89	7.00
3	−5	−2	86.79	10.80
−3	−5	2	72.49	11.50
−3	−5	2	80.89	8.60
−3	5	2	103.49	11.60
−3	5	2	67.19	13.60
−3	5	2	87.49	9.10
3	5	−2	96.59	12.70
3	5	−2	71.29	11.10
3	5	−2	71.09	13.50
3	5	−2	84.09	8.20
−3	5	2	81.09	11.10

3	5	-2	76.69	7.00
-3	5	2	59.19	10.70
-2	-5	2	89.29	11.00
-2	-5	2	75.59	5.30
2	-5	-2	76.59	5.90
2	-5	-2	97.59	16.30
2	-5	-2	58.59	8.60
-2	-5	2	83.49	12.90
-2	-5	2	91.19	9.10
-2	-5	2	86.99	8.60
2	-5	-2	79.89	12.20
2	5	-2	80.89	7.20
2	5	-2	73.69	5.90
-2	5	2	82.09	12.80
2	5	-2	85.39	12.50
2	5	-2	67.19	9.30
-2	5	2	79.29	7.20
-2	5	2	83.79	11.40
-2	5	2	89.59	15.60
2	5	-2	83.99	8.40
-2	5	2	78.49	7.00
-1	-5	2	1123.59	25.90
-1	-5	2	1115.09	36.20
1	-5	-2	1067.99	35.80
1	-5	-2	1081.29	39.40
-1	-5	2	1126.59	35.10
-1	-5	2	1085.79	33.70
1	-5	-2	1066.39	29.10
1	5	-2	1114.19	28.10
1	5	-2	1133.69	37.60
1	5	-2	1085.09	37.80

1	5	-2	1084.49	33.20
1	5	-2	1070.19	36.80
-1	5	2	1140.29	30.70
-1	5	2	1184.28	44.50
-1	5	2	1176.58	48.10
0	-5	-2	1302.87	48.60
0	-5	-2	1260.67	39.10
0	-5	2	1273.37	40.30
0	-5	-2	1236.28	32.60
0	-5	-2	1258.77	41.90
0	5	2	1255.47	33.40
0	5	-2	1214.08	41.80
0	5	-2	1220.58	40.00
0	5	-2	1313.77	33.20
0	5	2	1296.27	35.00
0	5	-2	1307.37	37.20
0	5	-2	1283.27	29.30
-1	-5	-2	1202.68	40.40
-1	-5	-2	1130.99	30.70
-1	-5	-2	1199.38	44.90
1	-5	2	1119.99	28.20
-1	-5	-2	1106.99	35.80
-1	-5	-2	1127.19	42.40
1	-5	2	1141.89	33.80
1	-5	2	1122.09	27.10
-1	-5	-2	1082.79	31.30
1	5	2	1174.88	36.20
1	5	2	1118.19	31.30
-1	5	-2	1058.89	34.80
1	5	2	1086.09	31.00
1	5	2	1132.39	32.00

-1	5	-2	1144.99	33.40
-1	5	-2	1202.88	41.90
2	-5	2	311.37	14.20
-2	-5	-2	309.57	19.80
-2	-5	-2	328.97	16.70
-2	-5	-2	336.07	13.60
-2	-5	-2	328.77	17.00
2	-5	2	312.27	10.70
2	5	2	335.97	16.70
2	5	2	331.27	20.40
-2	5	-2	351.26	15.00
2	5	2	340.37	14.40
2	5	2	348.77	17.00
2	5	2	348.87	20.60
-2	5	-2	338.47	17.20
2	5	2	326.17	14.30
-3	-5	-2	80.59	12.60
3	-5	2	106.49	14.70
-3	-5	-2	79.69	9.80
3	-5	2	80.99	7.00
-3	-5	-2	96.09	7.80
3	-5	2	93.49	7.40
-3	-5	-2	84.89	12.90
3	5	2	116.39	16.60
-3	5	-2	93.69	5.50
3	5	2	98.89	9.20
3	5	2	95.89	8.00
3	5	2	105.39	13.10
-3	5	-2	70.79	10.60
-3	5	-2	83.79	7.40
-3	5	-2	77.29	13.70

3	5	2	96.09	13.80
−3	5	−2	72.09	6.80
4	−5	2	247.18	11.80
−4	−5	−2	254.07	21.30
−4	−5	−2	250.07	20.00
4	−5	2	242.48	20.20
4	−5	2	246.68	14.00
−4	−5	−2	284.87	16.30
−4	5	−2	267.47	14.70
−4	5	−2	254.67	10.80
−4	5	−2	235.88	13.20
−4	5	−2	281.77	23.50
4	5	2	220.08	19.00
4	5	2	256.27	14.20
4	5	2	250.07	25.60
4	5	2	275.07	16.00
−4	5	−2	240.88	18.40
4	5	2	270.07	19.70
−5	−5	−2	267.07	22.80
−5	−5	−2	265.47	24.20
−5	−5	−2	235.68	16.00
5	−5	2	242.48	11.50
−5	5	−2	228.78	13.70
−5	5	−2	261.87	20.80
−5	5	−2	242.38	16.80
5	5	2	214.48	20.30
−5	5	−2	239.38	12.20
−5	5	−2	278.77	24.50
5	5	2	257.17	15.00
−6	−5	−2	219.68	26.70
6	−5	2	239.18	11.20

−6	−5	−2	257.57	25.50
6	5	2	233.18	15.30
−6	5	−2	210.68	20.10
−6	5	−2	218.18	17.90
−6	5	−2	238.48	30.80
−7	−5	−2	268.67	32.60
−7	−5	−2	243.28	17.90
−7	−5	−2	262.77	26.60
−7	5	−2	281.97	26.30
7	5	2	257.47	18.10
−7	5	−2	264.57	28.80
−7	5	−2	254.47	22.10
−8	−5	−2	4.50	16.50
8	−5	2	13.40	5.70
−8	5	−2	9.20	10.30
−8	5	−2	−8.60	11.30
−8	5	−2	3.50	10.50
8	5	2	1.50	6.40
−9	−5	−2	−17.90	12.30
−9	−5	−2	−7.40	14.00
9	−5	2	−8.00	4.50
−9	5	−2	8.10	14.70
−9	5	−2	1.00	14.30
9	5	2	1.80	8.00
−9	5	−2	13.20	11.60
−10	−5	−2	4.60	19.60
−10	−5	−2	2.20	17.60
−10	5	−2	30.70	20.70
−10	5	−2	−2.20	15.00
10	5	2	6.10	11.90
−10	5	−2	−5.80	13.30

-11	-5	-2	-10.10	20.40
-11	-5	-2	14.00	23.10
-11	5	-2	18.00	17.20
-11	5	-2	8.00	20.70
11	5	2	0.30	12.10
-11	5	-2	-3.50	22.60
-10	-6	2	-6.00	19.20
-10	-6	2	10.80	21.90
10	-6	-2	-7.00	12.50
10	-6	-2	13.40	10.50
-10	6	2	10.50	16.10
-10	6	2	-11.80	17.70
10	6	-2	2.50	10.10
-10	6	2	13.80	21.70
-9	-6	2	-5.20	16.70
9	-6	-2	31.70	11.20
-9	-6	2	-6.90	15.00
9	-6	-2	16.30	9.30
-9	6	2	-0.90	13.00
-9	6	2	59.49	22.40
-9	6	2	43.50	26.40
9	6	-2	1.80	7.50
-8	-6	2	35.10	16.70
-8	-6	2	30.60	20.00
8	-6	-2	17.90	8.20
8	-6	-2	29.50	10.00
-8	6	2	33.80	13.90
-8	6	2	21.10	15.00
8	6	-2	36.70	9.70
-8	6	2	18.20	15.20
-7	-6	2	11.30	13.60

7	−6	−2	7.60	6.60
7	−6	−2	10.30	6.10
−7	−6	2	1.90	11.10
−7	6	2	−8.90	10.00
−7	6	2	12.30	12.60
−7	6	2	12.20	10.50
7	6	−2	10.80	7.40
−6	−6	2	13.10	13.20
6	−6	−2	6.90	6.50
6	−6	−2	2.70	4.60
−6	−6	2	21.70	11.80
−6	6	2	−3.40	8.00
−6	6	2	8.20	11.70
6	6	−2	10.60	5.90
−6	6	2	5.10	9.50
−5	−6	2	129.49	22.80
−5	−6	2	87.29	16.90
−5	−6	2	106.49	13.70
5	−6	−2	91.19	12.50
5	−6	−2	118.19	16.40
−5	−6	2	111.79	14.50
−5	6	2	99.69	20.90
−5	6	2	94.09	14.40
−5	6	2	88.09	16.30
5	6	−2	93.59	11.00
−4	−6	2	3.30	5.90
4	−6	−2	13.90	6.50
−4	−6	2	1.20	3.60
4	−6	−2	8.80	3.10
−4	−6	2	−4.00	7.30
4	−6	−2	3.30	6.00

-4	-6	2	2.80	6.00
-4	-6	2	3.50	4.00
4	6	-2	0.80	4.40
4	6	-2	5.80	7.30
-4	6	2	4.50	6.00
-4	6	2	-5.90	7.40
-4	6	2	8.20	7.90
-4	6	2	0.10	5.20
-3	-6	2	1028.80	29.40
-3	-6	2	1027.90	44.30
3	-6	-2	1063.49	33.70
-3	-6	2	1027.10	40.10
3	-6	-2	1029.10	28.40
-3	-6	2	1070.49	36.50
3	-6	-2	968.30	36.90
3	6	-2	1126.39	42.50
3	6	-2	1057.89	31.50
-3	6	2	1058.89	43.60
-3	6	2	1062.89	34.00
3	6	-2	1045.20	39.70
-3	6	2	957.90	32.00
-3	6	2	1066.09	38.90
3	6	-2	1030.60	34.20
-2	-6	2	1932.01	60.89
-2	-6	2	1931.01	56.69
-2	-6	2	1927.11	55.89
-2	-6	2	1884.01	45.10
2	-6	-2	1837.22	55.89
2	-6	-2	1925.41	61.89
-2	-6	2	1936.51	56.59
2	-6	-2	1944.21	46.00

2	−6	−2	1912.01	45.50
−2	6	2	1911.71	56.19
−2	6	2	1949.70	71.59
−2	6	2	1822.02	60.19
2	6	−2	1949.01	55.99
2	6	−2	1892.91	57.99
−2	6	2	1881.11	46.40
2	6	−2	1898.61	52.19
−2	6	2	1844.12	49.40
−1	−6	2	460.85	21.10
1	−6	−2	441.36	15.10
1	−6	−2	443.76	24.50
−1	−6	2	449.46	19.80
−1	−6	2	476.15	23.80
1	−6	−2	419.76	29.00
1	−6	−2	437.16	21.60
1	6	−2	454.75	22.40
−1	6	2	496.25	17.40
1	6	−2	458.45	15.10
−1	6	2	501.05	25.20
−1	6	2	473.35	21.90
1	6	−2	466.45	20.80
1	6	−2	452.15	19.90
−1	6	2	514.75	32.20
1	6	−2	429.96	20.80
0	−6	−2	37.30	11.30
0	−6	−2	23.70	4.10
0	−6	2	23.50	7.70
0	−6	−2	40.20	8.90
0	−6	2	31.40	6.30
0	−6	−2	20.20	5.40

0	−6	−2	50.19	9.40
0	6	−2	17.80	6.10
0	6	2	27.80	6.50
0	6	−2	18.00	7.30
0	6	2	34.00	10.60
0	6	−2	35.60	3.80
0	6	−2	15.00	4.50
0	6	2	38.20	5.50
−1	−6	−2	338.57	23.90
−1	−6	−2	347.07	21.80
1	−6	2	359.56	21.50
1	−6	2	377.16	13.90
−1	−6	−2	361.26	21.40
−1	−6	−2	351.26	14.30
1	−6	2	344.07	16.90
−1	−6	−2	359.76	14.70
−1	−6	−2	324.77	18.20
1	−6	2	318.37	12.10
−1	6	−2	343.07	22.40
1	6	2	369.26	19.00
1	6	2	379.16	24.20
−1	6	−2	353.36	16.80
−1	6	−2	313.07	16.40
1	6	2	364.36	14.80
1	6	2	345.27	15.20
1	6	2	343.37	14.00
−2	−6	−2	1926.61	61.39
−2	−6	−2	1830.92	51.69
−2	−6	−2	1792.92	52.99
−2	−6	−2	1774.22	45.90
2	−6	2	1796.12	53.29

−2	−6	−2	1830.02	65.29
2	−6	2	1765.42	42.70
2	6	2	1749.32	47.10
−2	6	−2	1740.03	48.90
2	6	2	1730.73	49.99
2	6	2	1756.12	59.39
−2	6	−2	1816.52	62.89
2	6	2	1722.03	57.09
−2	6	−2	1796.12	38.60
2	6	2	1831.52	48.10
−2	6	−2	1788.72	51.19
3	−6	2	721.63	22.30
−3	−6	−2	709.93	26.90
−3	−6	−2	746.53	40.50
−3	−6	−2	718.13	29.00
−3	−6	−2	737.63	35.00
3	−6	2	697.83	27.30
3	6	2	681.73	24.80
−3	6	−2	728.13	37.20
−3	6	−2	733.53	25.60
3	6	2	656.73	33.00
3	6	2	725.93	30.00
−3	6	−2	709.03	29.10
−3	6	−2	701.33	19.60
−3	6	−2	693.23	19.20
3	6	2	718.13	34.10
4	−6	2	15.00	9.70
−4	−6	−2	15.60	7.80
4	−6	2	22.80	4.90
4	−6	2	33.70	6.40
−4	−6	−2	3.60	8.20

4	6	2	6.20	8.40
−4	6	−2	14.50	8.90
−4	6	−2	31.20	8.30
4	6	2	26.50	10.40
−4	6	−2	24.20	6.20
4	6	2	29.90	6.30
−4	6	−2	28.70	4.70
−4	6	−2	24.40	5.70
5	−6	2	42.10	6.70
−5	−6	−2	35.10	8.50
−5	−6	−2	35.00	12.30
−5	−6	−2	32.10	10.60
−5	6	−2	36.30	8.40
−5	6	−2	45.80	10.50
−5	6	−2	35.40	5.70
−5	6	−2	25.40	10.70
5	6	2	30.20	6.50
5	6	2	34.10	11.80
−5	6	−2	50.09	8.50
−6	−6	−2	5.20	9.10
−6	−6	−2	13.20	6.20
6	−6	2	−2.30	3.90
−6	−6	−2	14.10	10.80
−6	6	−2	10.20	8.50
−6	6	−2	−5.40	6.40
6	6	2	8.60	5.50
−6	6	−2	7.90	11.80
−7	−6	−2	−2.70	9.80
−7	−6	−2	3.70	11.20
7	−6	2	12.50	4.50
−7	−6	−2	11.40	6.60

−7	6	−2	1.00	8.30
−7	6	−2	10.60	12.90
7	6	2	−7.30	5.90
−7	6	−2	10.10	11.10
−8	−6	−2	9.50	13.50
−8	−6	−2	36.80	16.40
−8	6	−2	20.10	17.30
−8	6	−2	12.00	10.10
−8	6	−2	53.59	16.80
8	6	2	21.50	8.60
−9	−6	−2	0.30	16.90
−9	−6	−2	10.40	15.10
9	6	2	−0.40	8.80
−9	6	−2	16.80	13.70
−9	6	−2	9.30	18.60
−9	6	−2	−5.10	11.30
−10	−6	−2	22.80	20.00
−10	−6	−2	22.00	21.30
−10	6	−2	9.10	14.00
−10	6	−2	19.40	19.10
10	6	2	24.30	13.40
−10	6	−2	26.90	24.50
−10	−7	2	21.40	24.00
−10	−7	2	5.70	20.90
10	−7	−2	35.10	13.50
−10	7	2	−10.50	26.60
10	7	−2	10.10	11.20
−10	7	2	−24.00	17.40
−10	7	2	−9.40	17.60
−9	−7	2	−3.10	16.50
9	−7	−2	14.70	8.40

9	−7	−2	−1.60	10.30
−9	−7	2	2.50	17.30
9	7	−2	−5.40	10.00
−9	7	2	−6.70	17.30
−9	7	2	12.50	16.10
8	−7	−2	−2.00	9.30
8	−7	−2	18.60	6.50
−8	−7	2	13.90	18.30
−8	−7	2	11.60	15.10
−8	7	2	−8.10	13.30
−8	7	2	20.40	12.50
−8	7	2	8.20	12.50
8	7	−2	14.60	9.30
7	−7	−2	43.40	7.70
−7	−7	2	42.10	19.30
−7	−7	2	37.00	14.60
7	−7	−2	28.70	9.80
−7	7	2	57.39	14.50
−7	7	2	49.80	15.90
−7	7	2	46.40	18.30
6	−7	−2	245.18	20.30
−6	−7	2	271.37	27.90
−6	−7	2	206.28	26.20
−6	7	2	292.37	31.50
−6	7	2	217.68	28.00
−6	7	2	249.28	29.10
6	7	−2	213.68	15.90
−5	−7	2	1.60	10.40
5	−7	−2	−16.00	8.50
−5	−7	2	12.50	8.40
5	−7	−2	7.30	3.40

−5	−7	2	5.60	6.00
5	−7	−2	5.70	6.50
−5	7	2	−7.30	7.60
−5	7	2	12.90	7.20
−5	7	2	1.20	10.20
5	7	−2	4.70	4.80
4	−7	−2	156.48	8.30
−4	−7	2	169.88	24.10
4	−7	−2	173.88	15.80
−4	−7	2	160.98	16.30
−4	−7	2	141.79	13.70
−4	−7	2	124.79	17.20
4	7	−2	122.69	14.40
−4	7	2	138.09	20.40
4	7	−2	140.19	18.80
−4	7	2	129.69	23.40
−4	7	2	122.99	12.60
4	7	−2	134.09	11.60
−3	−7	2	26.10	8.30
3	−7	−2	34.80	8.80
−3	−7	2	22.60	7.70
−3	−7	2	28.60	7.50
−3	−7	2	29.80	5.00
3	−7	−2	31.00	6.70
−3	−7	2	15.10	8.60
3	−7	−2	25.10	3.90
−3	7	2	9.70	9.90
3	7	−2	32.60	7.20
−3	7	2	29.80	6.20
3	7	−2	20.30	8.40
3	7	−2	27.60	5.80

3	7	-2	26.70	9.60
-3	7	2	32.00	11.60
-2	-7	2	132.29	16.10
2	-7	-2	153.68	7.60
-2	-7	2	167.48	13.90
-2	-7	2	134.19	7.40
-2	-7	2	119.89	14.40
2	-7	-2	158.68	14.00
2	7	-2	155.48	9.70
2	7	-2	161.38	14.80
-2	7	2	147.79	24.90
2	7	-2	152.68	14.90
-2	7	2	133.99	9.90
-2	7	2	139.49	17.70
2	7	-2	163.78	12.40
-1	-7	2	22.50	8.40
1	-7	-2	10.20	4.80
-1	-7	2	11.70	4.70
1	-7	-2	11.50	2.90
-1	-7	2	14.90	5.20
1	7	-2	2.60	2.30
-1	7	2	14.10	8.50
1	7	-2	11.00	6.50
-1	7	2	8.80	4.30
1	7	-2	29.50	7.80
-1	7	2	14.70	4.80
1	7	-2	8.20	4.80
0	-7	-2	355.96	21.90
0	-7	2	333.07	18.60
0	-7	-2	375.96	21.00
0	-7	2	356.96	22.80

0	7	-2	384.66	19.90
0	7	2	395.36	28.60
0	7	-2	383.06	24.20
0	7	-2	372.86	12.70
0	7	2	383.36	15.70
0	7	2	377.46	16.70
1	-7	2	23.30	5.00
1	-7	2	32.90	7.40
1	-7	2	31.30	5.60
-1	-7	-2	21.50	11.20
-1	-7	-2	29.30	5.30
-1	-7	-2	36.60	9.40
-1	7	-2	34.60	6.90
-1	7	-2	21.70	3.40
1	7	2	24.50	10.10
1	7	2	18.60	4.70
-1	7	-2	38.20	11.40
-1	7	-2	26.30	6.30
1	7	2	38.10	6.50
2	-7	2	140.69	11.90
-2	-7	-2	146.19	13.30
2	-7	2	158.58	8.30
-2	-7	-2	178.88	21.10
-2	-7	-2	187.18	10.40
-2	-7	-2	169.18	13.00
2	-7	2	149.39	9.60
-2	7	-2	143.89	11.80
2	7	2	139.99	11.60
2	7	2	141.49	10.90
-2	7	-2	158.18	6.10
-2	7	-2	150.48	12.10

−2	7	−2	171.58	22.20
2	7	2	188.98	19.00
−3	−7	−2	67.69	10.70
3	−7	2	75.09	9.50
3	−7	2	65.99	7.50
−3	−7	−2	54.09	12.60
3	7	2	57.89	8.80
−3	7	−2	80.09	20.70
−3	7	−2	60.99	5.40
−3	7	−2	86.69	11.10
3	7	2	59.49	12.50
−3	7	−2	55.59	8.70
3	7	2	86.09	12.20
3	7	2	47.80	11.80
4	−7	2	32.00	10.30
−4	−7	−2	27.90	9.60
−4	−7	−2	36.00	12.50
−4	−7	−2	26.20	7.60
4	−7	2	37.80	6.80
4	−7	2	45.10	8.10
−4	7	−2	39.00	5.60
−4	7	−2	34.70	13.30
4	7	2	27.60	10.20
4	7	2	23.30	10.60
−4	7	−2	34.90	5.70
−4	7	−2	34.70	7.40
−4	7	−2	37.80	9.00
4	7	2	39.40	7.20
5	−7	2	9.00	4.40
−5	−7	−2	4.50	8.80
−5	−7	−2	−0.60	5.30

−5	−7	−2	−5.60	7.50
−5	7	−2	−4.00	4.10
−5	7	−2	3.40	10.10
5	7	2	−0.90	7.40
−5	7	−2	−3.60	6.00
−5	7	−2	−2.20	6.20
−5	7	−2	−4.00	5.50
5	7	2	−0.80	5.00
−6	−7	−2	224.78	30.30
−6	−7	−2	293.77	27.80
−6	−7	−2	256.37	19.10
−6	7	−2	283.97	23.90
−6	7	−2	218.48	19.10
6	7	2	240.58	17.40
6	7	2	261.67	24.90
−6	7	−2	217.38	17.10
−6	7	−2	276.37	29.30
−7	−7	−2	23.10	9.00
−7	−7	−2	42.70	16.70
−7	−7	−2	8.10	13.10
7	−7	2	32.90	6.60
7	7	2	23.70	9.10
−7	7	−2	7.40	8.80
−7	7	−2	14.90	11.80
−8	−7	−2	−27.60	13.10
−8	−7	−2	−9.80	8.00
8	−7	2	−5.90	5.10
−8	−7	−2	2.70	15.50
−8	7	−2	10.90	13.50
−8	7	−2	14.10	11.30
8	7	2	1.80	7.40

−8	7	−2	−7.60	14.40
−9	−7	−2	0.00	16.50
−9	−7	−2	−5.60	16.80
−9	7	−2	−20.00	14.80
−9	7	−2	−14.50	14.30
−9	7	−2	−14.20	12.80
9	7	2	−11.90	8.50
−10	−7	−2	14.00	19.30
−10	−7	−2	23.40	21.50
10	7	2	−3.10	12.10
−10	7	−2	29.20	25.50
−10	7	−2	9.70	17.30
−10	7	−2	28.30	19.50
−10	−8	2	−1.20	23.10
−10	8	2	−14.30	19.80
−10	8	2	−16.80	18.70
10	8	−2	−6.00	11.50
−10	8	2	19.80	25.90
−9	−8	2	−10.30	19.00
−9	−8	2	21.80	22.20
9	−8	−2	8.10	11.70
−9	8	2	4.30	15.60
9	8	−2	5.70	9.00
−9	8	2	23.10	21.80
−8	−8	2	32.70	19.20
−8	−8	2	84.59	22.90
8	−8	−2	57.19	14.20
8	8	−2	57.89	12.60
−8	8	2	80.19	26.80
−8	8	2	41.00	16.90
−8	8	2	64.59	18.80

7	−8	−2	−0.70	5.40
−7	−8	2	31.30	15.60
−7	−8	2	9.00	13.40
7	−8	−2	9.10	9.60
−7	8	2	−1.80	10.30
−7	8	2	7.00	12.50
−7	8	2	31.40	13.30
7	8	−2	3.60	8.20
6	−8	−2	8.30	8.50
−6	−8	2	−11.30	5.80
−6	−8	2	−4.20	10.90
−6	−8	2	11.80	13.40
6	8	−2	1.10	5.40
−6	8	2	22.70	13.20
−6	8	2	3.10	8.80
−6	8	2	7.10	14.40
−5	−8	2	−2.70	9.00
−5	−8	2	−5.00	11.20
−5	−8	2	0.00	5.80
5	−8	−2	−2.00	6.40
−5	−8	2	7.20	9.90
−5	8	2	19.20	10.80
5	8	−2	5.30	6.20
−5	8	2	−4.50	6.60
−5	8	2	8.30	12.20
−4	−8	2	161.68	21.70
−4	−8	2	169.68	17.90
−4	−8	2	132.79	16.90
4	−8	−2	167.98	18.50
4	−8	−2	154.88	15.40
4	8	−2	158.28	13.00

4	8	−2	147.19	14.00
−4	8	2	203.48	31.60
4	8	−2	151.48	19.70
−4	8	2	156.78	22.00
−4	8	2	166.48	13.60
−3	−8	2	161.68	21.00
−3	−8	2	138.89	8.90
−3	−8	2	152.38	15.30
−3	−8	2	157.38	19.00
3	−8	−2	140.09	16.80
3	−8	−2	131.99	6.40
−3	−8	2	166.98	19.70
−3	8	2	138.39	23.60
3	8	−2	149.88	18.70
−3	8	2	130.09	9.50
−3	8	2	138.99	18.50
3	8	−2	149.69	12.60
3	8	−2	166.28	14.30
3	8	−2	127.29	18.30
−2	−8	2	44.60	9.30
2	−8	−2	64.39	11.20
−2	−8	2	58.29	12.00
−2	−8	2	63.69	5.70
−2	−8	2	62.09	11.70
−2	8	2	86.39	15.20
2	8	−2	68.09	12.20
2	8	−2	67.69	8.20
2	8	−2	64.69	11.90
−2	8	2	67.69	8.80
−2	8	2	87.89	19.10
−1	−8	2	71.99	10.60

1	−8	−2	63.09	10.70
1	−8	−2	57.19	11.80
−1	−8	2	37.30	12.10
−1	−8	2	52.79	9.80
−1	8	2	61.59	8.80
1	8	−2	57.89	9.90
−1	8	2	58.99	7.30
1	8	−2	51.29	11.70
1	8	−2	60.89	7.60
1	8	−2	73.19	10.20
−1	8	2	82.99	17.80
0	−8	−2	90.49	13.10
0	−8	2	98.09	11.30
0	−8	−2	90.99	14.50
0	−8	2	99.19	13.30
0	−8	2	96.19	15.40
0	8	2	70.29	17.60
0	8	−2	80.89	14.30
0	8	−2	86.49	11.40
0	8	2	99.09	10.50
0	8	−2	86.19	6.60
1	−8	2	52.39	8.60
−1	−8	−2	39.70	10.80
1	−8	2	66.09	14.00
−1	−8	−2	52.89	9.80
1	−8	2	61.69	7.50
−1	8	−2	72.79	5.20
1	8	2	61.39	5.80
−1	8	−2	87.89	10.50
1	8	2	67.19	8.90
−1	8	−2	59.09	9.90

−1	8	−2	91.89	17.20
−2	−8	−2	204.88	14.60
2	−8	2	187.18	10.40
2	−8	2	226.28	15.50
−2	−8	−2	223.78	20.20
−2	−8	−2	201.28	11.70
−2	−8	−2	191.98	16.40
−2	8	−2	188.28	13.80
−2	8	−2	201.68	8.20
2	8	2	194.88	13.30
−2	8	−2	211.28	23.10
2	8	2	204.58	13.60
2	8	2	235.78	22.10
−3	−8	−2	61.69	11.30
3	−8	2	68.59	8.00
−3	−8	−2	76.69	17.20
3	−8	2	62.59	9.70
3	8	2	101.89	17.20
−3	8	−2	76.49	6.70
3	8	2	97.89	11.20
−3	8	−2	72.19	10.70
3	8	2	74.99	14.20
−3	8	−2	88.19	17.20
3	8	2	87.69	12.80
4	−8	2	354.96	17.20
−4	−8	−2	330.37	21.80
−4	−8	−2	343.77	25.80
−4	−8	−2	280.57	26.90
4	−8	2	341.77	18.80
−4	8	−2	341.07	31.30
−4	8	−2	343.67	21.40

4	8	2	350.86	26.10
4	8	2	292.37	18.20
4	8	2	364.46	28.80
-4	8	-2	350.26	14.90
5	-8	2	2.50	5.10
-5	-8	-2	15.90	10.70
-5	-8	-2	3.10	5.70
-5	-8	-2	9.30	10.40
-5	8	-2	6.00	4.80
-5	8	-2	-8.50	7.10
-5	8	-2	-10.10	11.40
-5	8	-2	0.20	6.80
5	8	2	0.90	6.00
5	8	2	3.00	7.80
-5	8	-2	13.60	5.90
6	-8	2	41.00	7.90
-6	-8	-2	52.09	16.40
-6	-8	-2	34.70	10.30
-6	-8	-2	42.00	15.20
-6	8	-2	9.00	14.20
-6	8	-2	43.80	12.20
-6	8	-2	21.30	10.10
-6	8	-2	32.60	9.70
6	8	2	10.10	10.80
6	8	2	19.10	8.20
-7	-8	-2	-9.10	7.30
-7	-8	-2	3.90	13.10
-7	-8	-2	0.40	14.60
7	-8	2	-6.00	6.00
-7	8	-2	-2.00	10.10
-7	8	-2	20.20	10.10

7	8	2	5.20	6.60
-7	8	-2	19.50	17.40
-8	-8	-2	19.80	9.80
-8	-8	-2	50.09	18.20
-8	-8	-2	15.20	17.40
8	8	2	46.00	11.80
-8	8	-2	26.80	20.30
-8	8	-2	46.70	15.10
-8	8	-2	22.20	14.30
-9	-8	-2	1.70	20.70
-9	-8	-2	-35.40	18.00
-9	8	-2	4.50	20.60
-9	8	-2	-16.00	13.80
-9	8	-2	17.50	13.50
9	8	2	2.90	9.40
-10	-8	-2	16.70	25.40
-10	-8	-2	14.50	29.00
10	8	2	-2.80	10.20
-10	8	-2	10.90	20.90
-10	8	-2	7.60	24.30
-10	8	-2	11.70	16.90
-9	-9	2	5.00	19.90
9	-9	-2	-4.20	13.40
-9	-9	2	6.80	22.60
-9	9	2	-13.00	17.00
-9	9	2	10.60	17.90
-8	-9	2	-27.40	16.90
-8	-9	2	-14.90	19.70
8	-9	-2	-19.30	10.50
8	9	-2	-9.90	9.10
-8	9	2	-5.80	18.60

−8	9	2	9.30	14.00
−8	9	2	−2.40	15.00
−7	−9	2	111.89	26.10
−7	−9	2	100.59	23.60
7	−9	−2	65.69	15.00
−7	9	2	53.29	22.10
−7	9	2	105.19	27.50
−7	9	2	100.49	19.40
7	9	−2	110.39	16.40
−6	−9	2	−9.40	12.90
6	−9	−2	47.10	12.70
−6	−9	2	22.20	17.50
−6	9	2	48.60	14.00
−6	9	2	45.20	13.30
−6	9	2	12.70	16.00
6	9	−2	34.80	9.10
5	−9	−2	63.29	13.20
−5	−9	2	37.90	9.90
5	−9	−2	40.50	10.00
−5	−9	2	43.00	16.60
−5	−9	2	39.20	14.60
5	9	−2	48.30	11.50
−5	9	2	25.50	16.30
−5	9	2	39.90	12.40
5	9	−2	41.00	10.20
−4	−9	2	202.98	23.10
−4	−9	2	197.18	24.30
−4	−9	2	180.78	18.40
4	−9	−2	210.68	16.10
4	−9	−2	173.48	17.00
−4	−9	2	231.78	28.60

4	9	−2	155.08	16.00
−4	9	2	159.78	21.00
4	9	−2	190.78	22.70
4	9	−2	202.88	15.00
−4	9	2	183.88	13.20
−4	9	2	185.18	30.80
−3	−9	2	28.90	5.30
−3	−9	2	41.70	10.70
3	−9	−2	30.60	9.80
−3	−9	2	−5.50	10.50
−3	9	2	23.40	10.00
3	9	−2	65.89	13.30
3	9	−2	−1.70	9.70
3	9	−2	42.20	10.20
−3	9	2	51.49	18.70
3	9	−2	42.10	8.30
−2	−9	2	111.59	15.00
2	−9	−2	73.59	13.10
−2	−9	2	122.59	16.40
−2	−9	2	96.19	16.70
2	9	−2	122.29	15.80
−2	9	2	108.39	12.30
−2	9	2	88.09	21.60
2	9	−2	109.59	15.00
2	9	−2	107.19	17.50
2	9	−2	101.99	9.20
1	−9	−2	202.38	17.30
1	−9	−2	217.38	19.00
−1	−9	2	171.68	15.80
−1	−9	2	182.78	23.30
−1	−9	2	203.58	16.70

-1	9	2	208.98	13.80
1	9	-2	196.98	18.60
-1	9	2	225.28	30.40
1	9	-2	211.18	11.70
1	9	-2	207.78	16.10
0	-9	-2	334.97	23.40
0	-9	2	305.27	20.30
0	-9	-2	342.27	22.20
0	-9	2	290.07	25.50
0	9	-2	326.57	26.40
0	9	-2	318.97	18.70
0	9	2	303.87	29.00
0	9	-2	325.57	13.60
0	9	2	315.57	16.80
-1	-9	-2	204.88	17.90
1	-9	2	219.18	17.50
1	-9	2	213.78	15.30
1	-9	2	208.68	13.20
-1	9	-2	236.48	10.30
1	9	2	227.28	16.30
-1	9	-2	233.28	14.20
-1	9	-2	212.98	23.20
1	9	2	251.27	26.10
-1	9	-2	215.38	10.30
-1	9	-2	202.48	15.80
2	-9	2	-0.70	4.30
-2	-9	-2	-3.20	5.40
-2	-9	-2	6.40	4.70
2	-9	2	5.30	5.80
2	-9	2	8.30	6.00
-2	-9	-2	-3.40	7.80

−2	−9	−2	−1.50	5.40
2	9	2	−4.40	7.50
2	9	2	3.90	4.90
−2	9	−2	1.80	3.30
2	9	2	8.50	5.50
−2	9	−2	−2.20	9.80
−2	9	−2	8.90	5.30
3	−9	2	63.29	9.00
−3	−9	−2	56.59	15.60
3	−9	2	61.29	10.70
−3	−9	−2	60.49	11.70
3	−9	2	44.10	7.90
−3	9	−2	69.49	10.60
−3	9	−2	56.79	16.20
3	9	2	67.09	10.40
−3	9	−2	57.49	6.50
3	9	2	62.79	11.70
3	9	2	43.00	13.80
−4	−9	−2	8.40	9.70
4	−9	2	−7.40	5.10
−4	−9	−2	−1.10	10.60
−4	−9	−2	−6.60	5.30
4	9	2	2.30	5.90
−4	9	−2	7.60	5.30
4	9	2	−0.50	6.80
−4	9	−2	5.30	12.20
−4	9	−2	0.40	6.80
−5	−9	−2	29.90	13.90
5	−9	2	30.20	10.10
−5	−9	−2	28.30	10.10
−5	−9	−2	44.00	15.30

5	9	2	39.30	14.00
−5	9	−2	30.90	7.70
−5	9	−2	41.50	11.40
−5	9	−2	34.70	15.40
5	9	2	38.40	9.20
6	−9	2	79.09	11.80
−6	−9	−2	68.99	20.80
−6	−9	−2	69.59	14.70
−6	−9	−2	105.09	22.30
6	9	2	67.69	18.50
−6	9	−2	74.29	16.60
6	9	2	52.79	11.10
−7	−9	−2	57.09	20.80
7	−9	2	86.19	12.40
−7	9	−2	76.49	16.10
−7	9	−2	88.79	14.40
−7	9	−2	21.30	18.80
7	9	2	89.09	14.70
8	−9	2	19.40	8.30
−8	−9	−2	26.90	19.00
−8	−9	−2	32.00	12.10
−8	−9	−2	−8.40	17.80
−8	9	−2	8.80	14.30
−8	9	−2	11.00	20.30
−8	9	−2	20.80	12.10
8	9	2	12.00	9.10
−9	−9	−2	12.10	23.40
−9	−9	−2	26.80	22.00
9	9	2	18.30	12.40
−9	9	−2	13.80	14.70
−9	9	−2	0.50	15.40

-9	-10	2	-11.40	24.20
-9	-10	2	-39.10	23.70
9	-10	-2	-16.80	15.60
-9	10	2	7.50	18.10
-9	10	2	9.90	26.60
-9	10	2	7.40	19.30
9	10	-2	19.10	12.80
-8	-10	2	-31.70	20.00
8	-10	-2	-2.00	11.10
-8	-10	2	34.60	19.00
-8	10	2	21.70	17.30
8	10	-2	-3.80	10.30
-8	10	2	-6.80	17.90
-8	10	2	5.60	14.90
7	-10	-2	11.00	10.50
-7	-10	2	-3.80	16.10
-7	-10	2	12.70	19.80
-7	10	2	5.80	17.70
-7	10	2	17.70	12.40
-7	10	2	12.90	13.80
7	10	-2	15.50	9.90
-6	-10	2	-18.90	12.50
-6	-10	2	1.50	8.60
6	-10	-2	21.70	10.30
-6	-10	2	22.40	16.60
-6	10	2	-11.60	9.00
-6	10	2	14.00	12.80
6	10	-2	-5.70	7.10
-6	10	2	8.00	14.70
5	-10	-2	27.30	12.70
-5	-10	2	-14.40	6.30

-5	-10	2	9.80	13.30
5	-10	-2	0.60	8.10
-5	-10	2	12.90	13.40
-5	10	2	11.30	10.70
5	10	-2	-4.40	6.80
5	10	-2	-1.70	7.80
-5	10	2	-20.80	12.30
-5	10	2	5.70	6.90
-4	-10	2	7.70	10.40
4	-10	-2	-7.40	7.20
-4	-10	2	-1.20	7.40
-4	-10	2	2.80	5.80
-4	-10	2	4.70	9.90
4	10	-2	-4.20	7.80
4	10	-2	-4.30	13.10
-4	10	2	19.30	17.10
-4	10	2	5.60	9.90
4	10	-2	-1.30	5.70
-3	-10	2	108.39	16.20
-3	-10	2	134.19	9.30
3	-10	-2	113.09	16.30
-3	10	2	153.98	28.70
3	10	-2	173.38	22.60
3	10	-2	133.29	12.00
3	10	-2	144.19	18.40
-2	-10	2	162.48	18.70
2	-10	-2	158.18	19.00
2	-10	-2	172.28	20.70
-2	-10	2	200.58	26.80
-2	-10	2	188.98	18.20
-2	10	2	161.08	13.40

2	10	-2	196.48	18.50
-2	10	2	158.18	27.60
-1	-10	2	101.59	14.30
1	-10	-2	94.49	15.90
-1	-10	2	115.69	15.30
1	-10	-2	82.89	13.80
1	10	-2	122.19	19.20
-1	10	2	112.99	15.10
1	10	-2	100.69	9.00
-1	10	2	124.89	25.30
1	10	-2	79.29	11.70
0	-10	-2	111.09	18.10
0	-10	-2	98.89	15.20
0	-10	2	71.39	12.60
0	10	2	102.89	12.30
0	10	-2	71.99	17.60
0	10	2	84.49	22.10
0	10	-2	108.29	9.20
0	10	-2	96.29	11.40
0	10	-2	108.79	11.90
0	10	-2	96.89	8.90
-1	-10	-2	21.10	11.60
1	-10	2	22.70	8.90
1	-10	2	27.40	7.80
-1	-10	-2	36.50	9.70
-1	10	-2	17.00	7.30
1	10	2	35.10	15.00
-1	10	-2	13.80	11.80
-1	10	-2	32.90	7.30
1	10	2	28.50	8.30
-1	10	-2	39.30	6.20

-2	-10	-2	444.06	28.10
2	-10	2	388.86	18.60
-2	-10	-2	427.86	31.30
2	-10	2	412.96	24.70
-2	10	-2	449.95	37.00
-2	10	-2	403.76	22.00
2	10	2	442.46	21.50
-2	10	-2	369.96	16.00
2	10	2	458.15	34.30
2	10	2	416.26	18.50
-2	10	-2	423.16	15.80
-2	10	-2	378.46	18.50
-3	-10	-2	82.69	16.60
-3	-10	-2	79.09	14.10
3	-10	2	89.09	12.50
3	-10	2	79.69	8.80
-3	10	-2	98.89	11.90
3	10	2	89.29	13.10
-3	10	-2	83.59	8.40
3	10	2	92.69	11.60
3	10	2	51.29	15.60
-3	10	-2	60.89	19.80
-4	-10	-2	30.00	12.10
-4	-10	-2	26.80	10.30
-4	-10	-2	59.99	19.00
4	-10	2	21.50	8.90
4	-10	2	32.90	8.10
-4	10	-2	20.80	8.30
4	10	2	19.90	7.60
-4	10	-2	20.30	7.20
-4	10	-2	55.79	19.50

4	10	2	12.40	10.80
-5	-10	-2	4.00	12.60
-5	-10	-2	0.90	13.70
-5	-10	-2	-2.10	9.60
5	-10	2	-3.10	6.90
-5	10	-2	4.30	8.90
5	10	2	-12.40	10.60
-5	10	-2	-14.90	14.40
-6	-10	-2	57.69	18.90
-6	-10	-2	17.90	10.80
6	-10	2	24.90	10.50
-6	-10	-2	36.30	17.10
6	10	2	18.80	13.10
-6	10	-2	21.20	11.90
-6	10	-2	18.40	17.90
6	10	2	31.20	8.90
-7	-10	-2	19.80	16.90
-7	-10	-2	-4.70	8.50
-7	-10	-2	39.30	19.00
7	-10	2	6.20	8.20
7	10	2	-5.80	7.20
-7	10	-2	-4.80	10.90
-7	10	-2	-16.80	16.70
-8	-10	-2	6.30	19.50
-8	-10	-2	19.90	12.30
-8	-10	-2	24.30	21.10
-8	10	-2	-15.60	9.20
-8	10	-2	-11.90	14.40
8	10	2	-12.30	8.60
-8	10	-2	18.90	22.40
-9	-10	-2	34.20	26.50

−9	−10	−2	16.40	23.50
−9	10	−2	16.50	25.00
9	10	2	5.60	12.40
−9	10	−2	1.00	14.00
−9	10	−2	59.79	21.20
−8	−11	2	52.09	27.30
−8	−11	2	3.40	22.20
−8	11	2	39.80	24.80
−8	11	2	32.40	17.00
−7	−11	2	31.50	18.60
−7	−11	2	7.50	19.90
7	−11	−2	0.90	12.70
7	11	−2	−5.50	9.60
−7	11	2	−6.50	21.60
−7	11	2	−2.80	14.10
6	−11	−2	51.59	15.30
−6	−11	2	31.80	10.70
−6	−11	2	41.60	20.80
−6	−11	2	53.19	22.20
−6	11	2	59.09	12.90
−6	11	2	48.20	25.30
6	11	−2	50.99	12.70
−6	11	2	44.70	17.20
−5	−11	2	19.00	15.10
5	−11	−2	−0.40	9.60
−5	−11	2	−22.40	14.30
−5	−11	2	8.20	9.70
−5	11	2	18.70	12.30
−5	11	2	37.50	20.80
5	11	−2	1.80	10.60
−4	−11	2	37.20	8.30

-4	-11	2	60.69	17.60
-4	-11	2	55.99	13.50
4	-11	-2	58.19	13.10
4	11	-2	41.60	10.10
-4	11	2	44.60	19.50
4	11	-2	34.10	12.60
4	11	-2	45.60	18.10
-3	-11	2	0.40	8.60
-3	-11	2	4.30	11.10
3	-11	-2	13.60	9.30
-3	11	2	-7.30	5.60
3	11	-2	1.70	5.90
3	11	-2	-6.80	12.00
-3	11	2	-13.00	13.60
-2	-11	2	90.09	16.40
-2	-11	2	95.69	16.00
2	-11	-2	76.59	14.90
2	-11	-2	109.79	19.30
-2	11	2	85.59	12.30
-2	11	2	161.78	30.20
2	11	-2	97.19	11.80
-1	-11	2	60.39	13.10
1	-11	-2	40.40	11.70
-1	-11	2	52.89	12.30
1	-11	-2	65.79	14.00
-1	11	2	45.60	16.90
-1	11	2	57.59	10.20
1	11	-2	66.99	9.10
1	11	-2	44.20	14.80
1	11	-2	75.19	10.70
0	-11	2	15.80	7.20

0	-11	-2	33.60	13.20
0	-11	-2	14.30	7.90
0	-11	2	2.30	7.30
0	11	-2	36.30	7.60
0	11	2	13.80	7.00
0	11	-2	10.00	6.30
0	11	2	24.50	14.10
0	11	-2	22.90	6.40
0	11	-2	6.90	11.80
1	-11	2	2.90	6.20
1	-11	2	28.30	8.70
-1	-11	-2	12.90	8.30
-1	-11	-2	8.70	9.90
-1	11	-2	14.60	5.80
-1	11	-2	27.10	6.00
1	11	2	31.30	8.30
-1	11	-2	24.30	6.30
1	11	2	35.20	15.60
-1	11	-2	18.70	14.50
-2	-11	-2	-1.10	7.70
-2	-11	-2	-4.70	10.00
2	-11	2	16.50	7.70
-2	11	-2	10.80	7.40
2	11	2	12.90	5.50
-2	11	-2	-2.60	13.40
-2	11	-2	13.50	5.00
-2	11	-2	3.60	5.20
-2	11	-2	-5.80	4.90
2	11	2	9.70	12.00
2	11	2	7.70	6.40
-3	-11	-2	36.50	14.70

-3	-11	-2	51.09	19.20
3	-11	2	19.10	6.90
3	-11	2	41.90	11.20
-3	-11	-2	41.40	10.70
-3	11	-2	28.50	7.60
3	11	2	28.90	13.40
3	11	2	40.20	9.60
-3	11	-2	49.50	19.80
-3	11	-2	30.30	7.50
3	11	2	26.10	9.50
-4	-11	-2	11.20	13.90
4	-11	2	17.20	9.10
-4	-11	-2	-15.50	10.50
-4	-11	-2	-3.50	7.10
4	-11	2	7.90	6.60
-4	11	-2	-0.20	16.40
4	11	2	2.00	7.40
4	11	2	3.10	11.30
-4	11	-2	-3.00	7.40
-5	-11	-2	15.20	9.90
-5	-11	-2	38.90	17.50
5	-11	2	18.60	10.20
-5	-11	-2	35.50	17.90
5	11	2	10.10	13.70
5	11	2	10.00	8.90
-5	11	-2	32.80	19.90
-5	11	-2	11.30	9.30
6	-11	2	58.59	14.20
-6	-11	-2	85.29	24.70
-6	-11	-2	65.89	22.00
-6	-11	-2	77.79	17.30

6	11	2	83.59	14.90
-6	11	-2	52.39	24.10
-6	11	-2	42.50	14.20
-7	-11	-2	19.20	20.40
-7	-11	-2	-25.40	20.00
-7	-11	-2	-7.00	9.70
7	-11	2	11.90	10.80
-7	11	-2	-18.90	12.50
-7	11	-2	-25.50	17.40
7	11	2	18.00	11.20
-8	-11	-2	-15.60	22.90
-8	-11	-2	20.60	14.30
-8	-11	-2	-29.00	22.40
8	11	2	-8.20	8.80
-8	11	-2	23.60	17.30
7	-12	-2	15.20	14.80
-7	-12	2	-58.59	21.90
-7	-12	2	39.80	31.50
-7	12	2	0.00	15.80
7	12	-2	-13.90	9.80
-6	-12	2	19.60	21.30
-6	-12	2	0.90	11.50
-6	-12	2	36.10	19.70
6	-12	-2	30.30	13.00
-6	12	2	-15.20	12.60
6	12	-2	5.10	8.50
-6	12	2	-5.40	19.60
-5	-12	2	3.20	17.30
5	-12	-2	35.50	13.50
-5	-12	2	39.70	12.60
-5	12	2	14.50	19.80

5	12	-2	8.50	12.00
5	12	-2	23.20	9.30
4	-12	-2	37.00	13.20
-4	-12	2	43.40	16.30
-4	-12	2	22.30	11.40
4	12	-2	48.90	11.70
-4	12	2	17.50	22.60
4	12	-2	24.40	12.60
-3	-12	2	-4.40	12.20
3	-12	-2	-1.60	7.80
3	-12	-2	31.90	13.60
-3	-12	2	13.80	8.90
3	12	-2	1.40	10.00
-3	12	2	19.60	19.00
-3	12	2	-8.80	6.90
3	12	-2	1.00	6.90
2	-12	-2	-8.10	8.20
2	-12	-2	-11.10	10.50
-2	-12	2	8.60	10.80
-2	-12	2	-1.40	8.00
-2	12	2	0.10	7.10
2	12	-2	-7.90	5.60
-2	12	2	-17.70	15.40
2	12	-2	19.50	11.80
-1	-12	2	18.40	9.30
1	-12	-2	-1.00	11.40
1	-12	-2	2.10	8.80
-1	-12	2	3.20	8.30
-1	12	2	-4.70	7.40
1	12	-2	-3.10	7.20
1	12	-2	10.50	11.80

-1	12	2	-19.10	12.10
1	12	-2	-6.90	4.90
0	-12	2	17.90	11.10
0	-12	-2	10.60	9.00
0	-12	2	8.30	8.70
0	12	-2	27.90	7.30
0	12	2	34.10	15.90
0	12	2	21.30	7.40
0	12	-2	17.60	6.50
0	12	-2	14.40	13.10
1	-12	2	-12.50	7.10
-1	-12	-2	16.60	11.40
-1	-12	-2	-7.90	8.70
1	-12	2	3.10	8.50
1	12	2	-6.40	6.50
-1	12	-2	9.90	13.40
-1	12	-2	-3.20	4.90
1	12	2	13.60	14.20
-2	-12	-2	32.40	11.50
2	-12	2	13.90	9.90
2	-12	2	-5.60	7.70
-2	-12	-2	-2.00	13.40
2	12	2	23.80	15.30
-2	12	-2	21.90	17.60
2	12	2	11.60	7.90
-2	12	-2	19.50	6.60
-3	-12	-2	11.80	15.90
3	-12	2	-7.20	7.30
3	-12	2	7.80	9.50
-3	-12	-2	6.90	9.70
3	12	2	31.10	9.30

3	12	2	7.60	14.00
−3	12	−2	−3.10	9.10
3	12	2	9.40	8.00
−3	12	−2	−6.80	8.00
−3	12	−2	−12.00	14.80
−3	12	−2	6.40	6.10
4	−12	2	87.99	15.80
−4	−12	−2	98.59	18.90
−4	−12	−2	127.99	19.50
−4	−12	−2	75.99	22.10
4	12	2	76.69	14.60
−4	12	−2	69.59	20.70
−4	12	−2	83.79	11.40
4	12	2	68.49	20.00
−5	−12	−2	19.60	18.60
−5	−12	−2	7.10	12.50
5	−12	2	27.80	11.50
−5	−12	−2	−19.00	15.20
5	12	2	8.20	9.80
−5	12	−2	−7.00	8.90
−5	12	−2	4.60	20.80
−6	−12	−2	10.10	14.10
−6	−12	−2	16.20	18.60
6	−12	2	18.10	12.60
6	12	2	8.90	8.40
6	12	2	−7.70	15.50
−6	12	−2	29.20	20.50
7	−12	2	1.20	12.50
−7	−12	−2	−2.00	11.20
−7	−12	−2	−17.50	21.50
7	12	2	45.90	20.10

−7	12	−2	21.90	15.10
−7	12	−2	9.40	23.20
−6	−13	2	23.30	20.70
−6	−13	2	44.40	24.70
6	13	−2	−2.30	10.90
−5	−13	2	3.60	20.50
−5	−13	2	4.60	11.70
5	−13	−2	−5.40	12.30
5	13	−2	−18.90	12.90
5	13	−2	2.80	9.20
−5	13	2	−26.40	19.90
−4	−13	2	19.40	13.80
4	−13	−2	23.30	10.80
4	13	−2	33.50	10.40
4	13	−2	1.80	12.20
−4	13	2	33.10	24.10
−4	13	2	14.60	10.20
3	−13	−2	−6.20	15.10
−3	−13	2	−8.80	8.50
−3	−13	2	14.80	13.80
3	−13	−2	6.70	11.80
−3	13	2	9.00	9.20
3	13	−2	−5.10	11.30
3	13	−2	4.20	7.80
−3	13	2	−25.40	17.20
2	−13	−2	−2.60	9.10
−2	−13	2	22.50	13.10
2	−13	−2	−10.00	14.00
−2	−13	2	4.60	10.40
−2	13	2	20.60	19.50
2	13	−2	4.70	7.60

-2	13	2	3.40	8.60
2	13	-2	22.00	13.20
-1	-13	2	15.00	10.70
1	-13	-2	-16.00	12.80
1	-13	-2	12.60	10.30
-1	-13	2	12.10	11.90
-1	13	2	-13.00	15.20
1	13	-2	-4.90	6.90
-1	13	2	10.60	7.60
1	13	-2	19.00	13.70
1	13	-2	5.70	6.60
0	-13	-2	63.59	15.60
0	-13	2	86.79	17.20
0	-13	-2	72.79	19.10
0	13	-2	69.09	18.50
0	13	2	89.29	14.20
0	13	-2	67.79	10.20
-1	-13	-2	6.40	9.60
1	-13	2	-3.60	7.70
1	-13	2	-2.40	13.70
-1	-13	-2	-1.90	12.80
1	13	2	-15.90	15.90
-1	13	-2	8.20	6.00
1	13	2	27.50	9.00
-1	13	-2	20.80	16.50
2	-13	2	8.40	8.60
-2	-13	-2	-3.90	17.60
-2	-13	-2	-6.80	8.50
-2	13	-2	10.30	16.00
-2	13	-2	8.40	7.00
2	13	2	21.60	8.60

2	13	2	−1.80	16.60
3	−13	2	6.60	10.50
−3	−13	−2	−4.40	11.00
−3	−13	−2	−4.50	15.50
3	13	2	−2.30	8.20
−3	13	−2	5.90	7.10
−3	13	−2	−3.00	18.40
−4	−13	−2	7.70	11.30
−4	−13	−2	19.70	20.30
4	−13	2	−0.80	9.60
4	13	2	−42.80	13.00
4	13	2	−10.00	7.80
−4	13	−2	−14.20	6.70
−4	13	−2	−2.20	18.40
5	−13	2	−4.30	12.30
−5	−13	−2	2.70	14.60
−5	−13	−2	−23.90	18.40
−5	−13	−2	3.60	17.00
−5	13	−2	33.20	24.30
5	13	2	0.10	10.20
5	13	2	−31.40	14.30
−6	−13	−2	16.00	12.50
−6	−13	−2	16.30	24.90
−6	−13	−2	−1.80	22.10
6	13	2	9.30	18.80
6	13	2	5.40	11.40
−6	13	−2	8.20	21.90
−5	−14	2	−0.20	13.10
5	−14	−2	2.50	15.50
5	14	−2	−38.60	14.10
−5	14	2	6.50	12.10

5	14	-2	10.70	10.90
4	-14	-2	6.70	15.10
-4	-14	2	-22.00	11.70
-4	-14	2	18.80	27.50
4	14	-2	7.20	10.80
-4	14	2	-23.50	9.90
-4	14	2	-10.40	19.50
4	14	-2	-33.80	14.00
-3	-14	2	3.50	10.90
3	-14	-2	-3.60	12.60
3	14	-2	-25.90	14.00
-3	14	2	10.80	11.10
-3	14	2	1.60	20.10
3	14	-2	6.70	9.30
-2	-14	2	10.50	11.30
2	-14	-2	16.70	14.10
-2	14	2	-16.30	20.60
-2	14	2	20.40	10.20
2	14	-2	-0.40	8.60
2	14	-2	-25.90	13.30
1	-14	-2	1.10	11.50
-1	-14	2	13.10	11.40
1	-14	-2	5.40	15.50
-1	14	2	2.60	19.60
1	14	-2	17.40	16.50
-1	14	2	3.20	10.00
1	14	-2	-4.00	7.10
0	-14	2	11.10	12.20
0	-14	-2	-19.80	16.20
0	-14	-2	-2.30	12.00
0	14	-2	2.10	18.10

0	14	-2	6.30	7.60
0	14	2	19.40	10.00
0	14	2	-6.20	19.60
-1	-14	-2	0.10	10.20
-1	-14	-2	-31.10	18.10
1	-14	2	6.40	10.40
-1	14	-2	3.10	7.00
-1	14	-2	-53.19	16.70
1	14	2	8.00	16.70
2	-14	2	46.50	15.90
-2	-14	-2	17.50	12.70
-2	-14	-2	-1.60	19.20
2	14	2	30.60	11.10
2	14	2	52.09	19.50
-2	14	-2	36.50	20.90
3	-14	2	-0.60	11.30
-3	-14	-2	-15.70	19.60
-3	-14	-2	-0.40	13.50
-3	14	-2	7.30	8.10
3	14	2	-0.30	8.80
-3	14	-2	5.40	19.90
3	14	2	9.80	18.80
-4	-14	-2	-10.50	23.60
-4	-14	-2	3.40	10.80
4	-14	2	-14.20	12.00
-4	14	-2	8.60	24.90
4	14	2	14.60	17.40
4	14	2	-13.60	9.30
-5	-14	-2	-6.30	26.70
-5	-14	-2	-14.70	13.70
5	-14	2	-24.60	12.70

5	14	2	-15.50	10.20
-5	14	-2	1.50	25.50
5	14	2	-31.80	17.00
-3	-15	2	15.60	14.00
3	-15	-2	8.80	15.70
-3	15	2	-8.60	22.90
-3	15	2	15.50	13.80
3	15	-2	-0.30	11.00
3	15	-2	-32.00	16.20
-2	-15	2	0.40	13.80
2	-15	-2	-11.60	13.70
-2	15	2	17.20	12.00
2	15	-2	-32.20	16.30
-2	15	2	-11.60	22.50
-1	-15	2	42.40	16.70
1	-15	-2	17.30	17.50
1	15	-2	28.30	22.90
-1	15	2	27.30	23.00
1	15	-2	21.80	11.10
0	-15	2	10.90	14.00
0	-15	-2	28.40	16.00
0	15	2	-7.30	20.20
0	15	2	16.70	11.50
0	15	-2	10.10	8.70
1	-15	2	25.60	15.70
-1	15	-2	54.29	23.80
-1	15	-2	21.70	9.60
1	15	2	6.30	10.60
1	15	2	27.60	24.90
2	-15	2	9.60	12.20
-2	-15	-2	-7.70	12.10

-2	15	-2	-12.10	23.00
-2	15	-2	12.90	8.50
2	15	2	-3.00	10.40
2	15	2	-2.30	21.50
-3	-15	-2	-0.70	13.60
3	-15	2	-8.40	14.80
-3	15	-2	-28.60	22.80
3	15	2	14.50	11.20
3	15	2	21.80	21.80
-11	0	3	30.90	22.90
11	0	-3	1.60	10.20
11	0	-3	10.70	13.40
-11	0	3	1.50	22.50
-11	0	3	-3.70	21.30
-10	0	3	-4.20	14.20
10	0	-3	2.70	8.90
10	0	-3	0.00	10.40
-10	0	3	-16.90	16.30
-10	0	3	-8.00	15.50
9	0	-3	35.00	15.10
-9	0	3	38.10	15.70
-9	0	3	66.29	18.50
-9	0	3	47.60	16.00
-8	0	3	-1.00	9.00
-8	0	3	-7.20	10.10
8	0	-3	13.50	6.80
-8	0	3	10.40	13.60
7	0	-3	178.08	13.60
-7	0	3	190.88	27.00
-7	0	3	195.78	25.30
-7	0	3	179.58	23.80

−6	0	3	2.20	6.70
6	0	−3	0.30	4.10
−6	0	3	−4.90	6.00
−6	0	3	10.90	8.20
5	0	−3	926.91	30.30
−5	0	3	899.51	38.70
−5	0	3	928.81	42.20
−5	0	3	903.11	40.70
−4	0	3	−2.30	3.60
−4	0	3	−3.30	4.10
4	0	−3	−6.70	6.00
4	0	−3	1.10	2.80
4	0	−3	7.80	6.50
−4	0	3	1.80	3.40
−4	0	3	9.20	5.10
−3	0	3	25.70	7.20
−3	0	3	26.00	6.30
−3	0	3	21.20	5.50
−3	0	3	19.70	3.70
3	0	−3	29.40	7.90
3	0	−3	17.40	6.30
2	0	−3	4.20	2.80
2	0	−3	−0.30	2.90
−2	0	3	3.50	3.20
2	0	−3	1.60	1.40
−2	0	3	1.40	3.10
−2	0	3	−0.90	1.50
−2	0	3	−0.40	3.70
2	0	−3	−1.10	3.30
−1	0	3	527.05	21.20
−1	0	3	528.55	15.80

1	0	−3	521.95	21.30
1	0	−3	521.65	16.10
1	0	−3	520.15	22.40
−1	0	3	523.15	19.30
0	0	3	1.20	1.90
0	0	−3	−0.30	1.60
0	0	−3	−1.10	2.40
0	0	3	0.10	1.10
0	0	3	0.90	1.50
0	0	−3	−0.10	1.40
0	0	3	2.10	1.30
1	0	3	377.46	11.20
−1	0	−3	385.06	15.30
−1	0	−3	378.66	11.40
−1	0	−3	385.16	15.60
−1	0	−3	367.66	17.50
−1	0	−3	392.26	12.50
−1	0	−3	357.86	12.80
1	0	3	386.46	17.80
1	0	3	370.06	12.10
−1	0	−3	405.96	16.40
1	0	3	357.66	15.40
−2	0	−3	−0.20	1.40
−2	0	−3	0.30	1.30
2	0	3	−0.10	1.30
2	0	3	−1.20	3.00
−2	0	−3	0.90	2.50
2	0	3	0.70	1.40
2	0	3	−0.90	2.60
2	0	3	−0.90	1.00
−2	0	−3	1.70	1.20

-2	0	-3	1.10	3.00
-2	0	-3	-0.50	2.80
3	0	3	255.07	10.10
-3	0	-3	263.87	7.80
3	0	3	248.88	10.40
-3	0	-3	242.38	17.10
-3	0	-3	254.27	10.80
3	0	3	250.67	14.90
-3	0	-3	269.27	16.50
3	0	3	262.57	17.60
-3	0	-3	273.07	10.70
3	0	3	270.67	8.80
4	0	3	1.40	1.40
4	0	3	-8.00	4.70
4	0	3	1.60	4.90
-4	0	-3	-0.60	2.50
-4	0	-3	-1.90	5.60
4	0	3	-1.40	2.30
-4	0	-3	2.40	4.90
-4	0	-3	1.40	3.90
5	0	3	3.90	1.70
5	0	3	-3.90	3.10
-5	0	-3	5.00	5.10
5	0	3	7.00	3.20
6	0	3	1.00	3.80
6	0	3	-4.70	4.50
-6	0	-3	2.10	6.50
-6	0	-3	-1.80	5.70
6	0	3	0.40	3.50
-7	0	-3	54.09	14.90
-7	0	-3	51.69	13.90

7	0	3	49.99	8.40
8	0	3	1.90	3.50
8	0	3	0.50	5.60
-8	0	-3	-0.30	10.00
-8	0	-3	-4.60	10.90
-9	0	-3	7.50	12.40
9	0	3	5.30	6.70
-9	0	-3	4.60	12.50
-10	0	-3	-21.00	14.00
-10	0	-3	10.50	18.00
10	0	3	-8.40	7.80
11	0	3	11.30	10.20
-11	0	-3	3.10	20.60
-11	-1	3	16.80	21.00
-11	-1	3	0.90	20.70
11	-1	-3	3.80	10.40
-11	-1	3	-9.90	19.20
11	-1	-3	-33.90	14.20
11	1	-3	49.20	15.10
-11	1	3	15.60	18.50
-11	1	3	6.30	18.90
11	1	-3	13.80	12.20
-11	1	3	4.30	24.60
10	-1	-3	19.30	12.90
-10	-1	3	11.90	17.90
-10	-1	3	-10.50	15.10
-10	-1	3	-16.30	13.50
10	-1	-3	10.50	8.70
-10	1	3	10.20	14.80
-10	1	3	21.50	17.80
10	1	-3	12.30	9.30

-10	1	3	34.70	15.90
-9	-1	3	20.50	14.20
-9	-1	3	-4.20	11.80
9	-1	-3	-0.40	7.40
9	-1	-3	3.00	9.10
-9	-1	3	-6.00	12.70
9	1	-3	-0.90	6.00
-9	1	3	7.00	12.50
-9	1	3	17.60	13.60
-9	1	3	-1.20	13.20
-8	-1	3	58.99	16.00
8	-1	-3	53.39	9.60
-8	-1	3	30.90	14.50
8	-1	-3	49.70	11.50
-8	1	3	68.59	16.00
8	1	-3	50.99	9.60
-8	1	3	74.19	18.60
-8	1	3	40.00	15.30
-7	-1	3	16.10	9.30
7	-1	-3	9.90	4.90
-7	-1	3	13.40	12.50
-7	1	3	23.00	9.90
7	1	-3	18.10	6.70
-7	1	3	24.00	11.40
-7	1	3	20.40	11.20
-6	-1	3	11.50	8.70
-6	-1	3	14.90	8.80
6	-1	-3	17.00	5.80
-6	1	3	18.20	10.20
-6	1	3	8.40	7.10
-6	1	3	16.60	8.20

6	1	−3	11.00	5.30
−5	−1	3	203.58	27.80
−5	−1	3	187.98	22.00
5	−1	−3	219.08	12.80
5	1	−3	192.68	13.90
−5	1	3	211.28	26.20
−5	1	3	189.58	17.50
−5	1	3	243.88	20.40
4	−1	−3	308.77	14.10
4	−1	−3	257.97	19.70
−4	−1	3	274.97	19.80
4	−1	−3	268.47	20.00
−4	−1	3	280.87	13.50
−4	−1	3	303.37	22.50
−4	1	3	295.27	20.60
−4	1	3	263.67	19.80
−4	1	3	272.87	19.30
−4	1	3	287.47	14.00
4	1	−3	288.87	13.80
4	1	−3	281.57	21.10
4	1	−3	230.68	18.50
3	−1	−3	51.79	10.30
−3	−1	3	32.50	6.50
−3	−1	3	38.60	6.40
3	−1	−3	38.30	9.20
−3	−1	3	36.00	5.30
3	1	−3	35.10	8.50
3	1	−3	28.40	6.90
−3	1	3	44.00	9.20
−3	1	3	28.60	4.90
−3	1	3	42.70	8.30

−2	−1	3	1452.25	48.10
2	−1	−3	1394.06	44.70
−2	−1	3	1476.15	36.30
−2	−1	3	1451.05	38.70
−2	−1	3	1444.76	44.20
2	−1	−3	1491.15	46.30
2	−1	−3	1462.05	45.80
2	−1	−3	1443.96	37.60
−2	1	3	1522.75	46.50
2	1	−3	1495.95	36.70
2	1	−3	1494.05	44.40
−2	1	3	1428.06	45.60
2	1	−3	1417.16	47.40
−2	1	3	1473.45	45.50
−2	1	3	1441.66	37.50
1	−1	−3	209.08	12.80
1	−1	−3	203.88	9.30
−1	−1	3	193.58	11.40
1	−1	−3	213.58	13.40
1	−1	−3	218.88	14.10
−1	−1	3	198.38	8.20
−1	−1	3	202.18	7.80
1	1	−3	193.08	13.50
1	1	−3	212.88	8.90
−1	1	3	199.38	9.10
1	1	−3	209.38	13.30
0	−1	3	425.36	16.80
0	−1	−3	430.66	14.60
0	−1	−3	438.56	19.50
0	−1	−3	414.26	18.40
0	−1	−3	446.16	17.60

0	−1	3	419.06	11.60
0	−1	3	422.96	13.30
0	−1	3	436.96	11.70
0	1	−3	418.46	12.20
0	1	3	400.06	14.00
0	1	3	415.06	16.10
0	1	3	441.36	19.80
0	1	−3	440.96	13.50
0	1	−3	433.16	16.60
0	1	3	414.06	13.70
−1	−1	−3	171.08	7.90
−1	−1	−3	148.49	10.50
−1	−1	−3	155.28	7.00
−1	−1	−3	155.08	10.70
−1	−1	−3	163.28	10.10
1	−1	3	155.48	8.70
1	−1	3	161.98	5.70
−1	−1	−3	164.58	8.50
−1	−1	−3	146.69	10.10
1	−1	3	150.28	5.00
−1	1	−3	163.48	11.50
1	1	3	152.38	8.40
1	1	3	151.38	7.00
−1	1	−3	153.98	6.50
−1	1	−3	157.18	6.00
1	1	3	165.48	12.40
−1	1	−3	154.38	9.10
−1	1	−3	157.08	9.20
1	1	3	146.99	10.10
−1	1	−3	156.68	8.80
1	1	3	165.78	7.70

1	1	3	165.28	10.40
−2	−1	−3	303.17	15.80
2	−1	3	300.67	13.10
−2	−1	−3	279.37	15.20
−2	−1	−3	320.87	11.80
2	−1	3	287.87	8.80
−2	−1	−3	298.67	11.10
2	−1	3	280.27	16.30
−2	−1	−3	278.87	16.10
−2	−1	−3	306.37	10.90
2	−1	3	283.67	9.60
2	1	3	296.27	15.30
2	1	3	308.67	11.50
−2	1	−3	279.87	8.80
2	1	3	277.87	16.00
2	1	3	301.97	11.30
2	1	3	295.77	10.90
−2	1	−3	301.37	17.00
−2	1	−3	286.87	14.90
−2	1	−3	289.47	13.00
−2	1	−3	294.07	9.50
2	1	3	277.27	15.40
−2	1	−3	287.17	9.90
3	−1	3	37.10	4.20
3	−1	3	53.79	9.40
−3	−1	−3	47.80	5.80
−3	−1	−3	42.00	5.20
−3	−1	−3	38.30	3.60
3	−1	3	38.20	3.90
−3	−1	−3	36.40	7.40
3	−1	3	30.20	5.80

3	1	3	50.29	5.80
3	1	3	29.40	4.10
3	1	3	41.20	4.10
-3	1	-3	39.00	4.40
3	1	3	43.10	8.40
-3	1	-3	38.10	6.40
3	1	3	29.60	6.70
-3	1	-3	40.90	4.50
-4	-1	-3	430.66	23.70
-4	-1	-3	456.85	24.20
-4	-1	-3	444.46	14.40
4	-1	3	467.75	15.30
4	-1	3	439.16	15.10
-4	-1	-3	460.85	26.10
4	-1	3	405.76	25.00
-4	-1	-3	441.66	16.80
4	-1	3	453.05	21.80
-4	1	-3	459.35	16.10
4	1	3	461.95	24.20
-4	1	-3	455.75	21.60
4	1	3	460.45	26.10
4	1	3	473.35	16.80
-4	1	-3	477.35	26.10
4	1	3	443.66	17.10
4	1	3	452.95	14.60
5	-1	3	65.89	6.70
5	-1	3	60.89	6.80
-5	1	-3	66.39	7.90
5	1	3	61.79	7.50
5	1	3	58.19	5.40
5	1	3	72.59	8.10

−5	1	−3	59.49	10.60
−6	−1	−3	33.80	10.70
6	−1	3	58.79	7.60
6	−1	3	49.90	6.80
−6	−1	−3	58.99	14.80
6	1	3	54.79	7.90
6	1	3	45.00	5.60
−6	1	−3	59.89	13.70
−6	1	−3	44.20	11.60
7	−1	3	37.40	7.10
−7	−1	−3	37.40	12.70
−7	−1	−3	47.30	12.40
−7	1	−3	50.89	11.70
7	1	3	36.10	5.80
7	1	3	51.29	8.90
−7	1	−3	29.80	13.50
−8	−1	−3	3.00	9.80
−8	−1	−3	2.70	10.70
8	−1	3	5.30	5.80
8	1	3	14.80	5.00
−8	1	−3	−2.20	11.20
−8	1	−3	0.60	9.00
8	1	3	8.10	6.00
−9	−1	−3	2.30	14.80
9	−1	3	13.20	7.10
−9	−1	−3	−9.20	10.40
9	1	3	10.60	6.80
9	1	3	−2.30	6.00
−9	1	−3	−5.90	13.30
−9	1	−3	16.40	12.80
10	−1	3	30.20	8.70

-10	-1	-3	19.40	15.70
-10	-1	-3	23.40	16.40
-10	1	-3	16.50	15.50
10	1	3	4.90	8.90
-10	1	-3	12.60	15.80
11	-1	3	2.60	9.60
-11	-1	-3	-16.70	29.00
11	1	3	6.20	10.60
-11	1	-3	18.80	20.00
11	-2	-3	-16.60	13.90
-11	-2	3	-14.60	18.50
11	-2	-3	-6.10	10.00
11	2	-3	0.50	10.50
-11	2	3	13.30	21.00
-11	2	3	-23.50	20.30
-10	-2	3	6.30	16.10
-10	-2	3	4.40	18.40
10	-2	-3	4.90	7.60
-10	-2	3	19.10	14.90
10	-2	-3	15.40	12.70
-10	2	3	-21.30	15.40
10	2	-3	4.50	9.40
-10	2	3	-23.20	13.00
-10	2	3	12.60	14.90
9	-2	-3	49.80	9.80
-9	-2	3	79.29	22.20
9	-2	-3	28.40	12.90
-9	-2	3	37.20	18.70
-9	2	3	46.00	16.00
-9	2	3	14.90	13.20
9	2	-3	55.19	11.50

−9	2	3	26.20	14.20
8	−2	−3	19.70	9.80
8	−2	−3	29.20	8.50
−8	−2	3	46.30	17.70
−8	−2	3	26.20	15.80
−8	2	3	2.30	11.00
−8	2	3	7.70	11.20
−8	2	3	37.30	15.70
8	2	−3	24.50	7.30
−7	−2	3	−6.30	8.40
−7	−2	3	2.40	8.70
7	−2	−3	6.40	5.50
7	−2	−3	5.60	6.30
7	2	−3	11.80	6.10
−7	2	3	−4.50	9.40
−7	2	3	12.30	7.90
−7	2	3	15.60	10.90
−6	−2	3	131.59	20.90
6	−2	−3	158.08	12.10
6	−2	−3	147.49	14.00
−6	−2	3	164.38	22.50
−6	2	3	162.78	22.50
−6	2	3	148.39	20.60
6	2	−3	133.99	11.70
−6	2	3	116.99	18.70
5	−2	−3	16.00	4.90
−5	−2	3	11.30	7.00
−5	−2	3	10.00	6.90
5	−2	−3	14.50	5.80
5	2	−3	14.20	5.30
5	2	−3	18.90	6.10

−5	2	3	18.80	7.60
−5	2	3	18.00	8.70
−5	2	3	23.10	7.60
−4	−2	3	80.69	12.30
−4	−2	3	76.49	7.10
4	−2	−3	86.49	13.90
−4	−2	3	65.99	13.00
4	−2	−3	88.89	7.70
4	−2	−3	110.59	10.80
4	−2	−3	61.19	11.60
−4	2	3	83.39	13.90
4	2	−3	96.79	7.90
4	2	−3	76.49	14.40
−4	2	3	59.79	11.00
−4	2	3	96.49	9.90
−4	2	3	106.69	15.40
4	2	−3	112.79	10.00
4	2	−3	95.59	16.00
3	−2	−3	489.65	26.10
−3	−2	3	487.25	27.10
3	−2	−3	514.85	22.20
−3	−2	3	489.05	17.10
3	−2	−3	490.75	24.70
−3	−2	3	495.25	24.30
3	−2	−3	515.45	26.90
−3	2	3	521.65	25.60
3	2	−3	485.25	26.80
3	2	−3	490.45	24.10
3	2	−3	545.35	20.80
−3	2	3	507.15	18.70
−3	2	3	519.35	24.70

2	-2	-3	169.18	12.50
2	-2	-3	180.78	14.40
-2	-2	3	184.58	15.00
2	-2	-3	195.68	12.50
-2	-2	3	172.38	12.10
-2	-2	3	179.08	8.10
2	-2	-3	158.58	14.20
-2	-2	3	165.38	7.90
2	-2	-3	164.28	9.20
2	2	-3	180.88	8.50
-2	2	3	179.88	14.70
-2	2	3	163.68	13.70
2	2	-3	174.48	12.30
2	2	-3	161.08	10.00
-2	2	3	150.28	12.00
-2	2	3	174.18	9.70
2	2	-3	161.18	14.60
1	-2	-3	257.57	14.00
1	-2	-3	251.77	16.70
1	-2	-3	242.48	11.20
-1	-2	3	252.77	13.00
1	-2	-3	265.57	13.90
-1	-2	3	266.17	8.70
1	-2	-3	268.97	17.10
-1	2	3	273.27	11.60
-1	2	3	240.08	13.60
1	2	-3	270.07	12.00
1	2	-3	254.67	12.90
1	2	-3	281.97	17.10
-1	2	3	288.97	17.30
1	2	-3	267.87	9.30

-1	2	3	250.77	16.60
0	-2	-3	1269.87	41.00
0	-2	-3	1259.77	40.30
0	-2	-3	1236.78	36.80
0	-2	-3	1302.37	34.00
0	-2	3	1312.67	27.80
0	-2	3	1295.07	27.80
0	2	-3	1326.17	33.60
0	2	3	1310.77	33.70
0	2	-3	1323.57	33.70
0	2	3	1257.57	33.50
0	2	-3	1312.97	31.20
0	2	-3	1342.57	29.50
0	2	3	1380.96	41.70
0	2	3	1305.97	41.50
0	2	3	1339.87	37.60
-1	-2	-3	1650.93	40.10
-1	-2	-3	1653.93	40.90
-1	-2	-3	1666.73	48.00
-1	-2	-3	1691.13	47.60
-1	-2	-3	1675.43	46.30
-1	-2	-3	1701.23	43.20
-1	-2	-3	1697.43	48.60
-1	2	-3	1659.53	35.50
-1	2	-3	1650.93	36.00
1	2	3	1666.23	40.80
-1	2	-3	1654.53	38.70
1	2	3	1646.14	47.60
1	2	3	1597.74	45.00
1	2	3	1636.64	43.00
1	2	3	1667.63	40.50

−1	2	−3	1680.73	39.20
−1	2	−3	1628.34	40.50
−1	2	−3	1658.13	48.50
−2	−2	−3	552.74	23.90
−2	−2	−3	520.85	17.70
−2	−2	−3	559.54	17.40
−2	−2	−3	541.85	23.10
−2	−2	−3	542.25	23.30
2	−2	3	506.85	17.40
−2	−2	−3	555.74	18.00
2	−2	3	535.55	23.80
2	2	3	547.45	23.70
2	2	3	533.95	23.10
−2	2	−3	547.95	14.50
−2	2	−3	524.65	14.20
−2	2	−3	555.24	24.60
2	2	3	544.85	18.30
−2	2	−3	512.75	16.90
−2	2	−3	549.65	18.00
2	2	3	524.35	22.70
2	2	3	557.74	17.60
2	2	3	540.55	17.50
−2	2	−3	555.74	14.00
−3	−2	−3	299.57	13.20
−3	−2	−3	271.97	18.00
3	−2	3	308.57	9.40
3	−2	3	307.17	9.00
−3	−2	−3	301.87	12.40
−3	−2	−3	329.07	22.60
3	−2	3	296.17	14.30
3	2	3	312.57	19.00

3	2	3	316.67	18.60
3	2	3	349.27	21.20
-3	2	-3	313.37	10.70
-3	2	-3	309.97	14.30
-3	2	-3	304.67	10.80
3	2	3	320.37	13.10
3	2	3	288.87	12.90
3	2	3	301.97	12.70
4	-2	3	15.50	6.70
-4	-2	-3	4.10	5.90
4	-2	3	7.60	2.50
4	-2	3	10.00	4.60
-4	-2	-3	12.40	3.70
4	-2	3	15.10	2.80
-4	-2	-3	12.90	6.50
-4	-2	-3	12.70	3.30
-4	-2	-3	6.80	5.40
4	2	3	4.20	6.40
4	2	3	5.60	3.00
4	2	3	20.20	8.50
4	2	3	6.50	3.40
-4	2	-3	11.00	3.30
-4	2	-3	8.40	4.90
4	2	3	16.80	3.60
-4	2	-3	-1.50	4.40
-5	-2	-3	3.60	6.60
5	-2	3	7.20	2.90
-5	-2	-3	12.10	7.20
5	-2	3	2.20	3.00
-5	-2	-3	4.20	3.00
-5	2	-3	9.80	6.70

5	2	3	1.90	3.20
5	2	3	9.20	3.90
−5	2	−3	10.70	4.00
6	−2	3	75.89	7.80
−6	−2	−3	71.49	14.40
6	−2	3	88.29	8.70
−6	−2	−3	79.99	15.50
−6	2	−3	77.79	15.60
6	2	3	76.89	7.90
6	2	3	84.79	9.80
−6	2	−3	65.49	12.10
−7	−2	−3	19.50	11.40
7	−2	3	36.10	6.30
7	−2	3	23.30	6.00
−7	−2	−3	45.50	13.00
−7	2	−3	24.40	11.00
−7	2	−3	36.70	12.40
7	2	3	32.40	6.30
7	2	3	33.40	7.90
−8	−2	−3	6.70	13.10
−8	−2	−3	14.60	12.80
8	−2	3	20.50	6.40
8	2	3	15.60	6.10
8	2	3	10.80	6.80
−8	2	−3	14.90	11.50
−8	2	−3	13.60	9.90
−9	−2	−3	9.30	15.60
−9	−2	−3	27.20	17.60
9	−2	3	22.80	6.80
9	2	3	9.50	8.00
9	2	3	9.80	12.50

−9	2	−3	6.00	12.20
−9	2	−3	1.00	13.00
−10	−2	−3	−13.60	13.90
−10	−2	−3	−13.70	13.00
10	−2	3	2.20	7.40
10	2	3	6.80	8.60
−10	2	−3	−13.10	13.70
−10	2	−3	10.40	15.30
−11	−2	−3	−28.50	18.80
−11	−2	−3	−12.40	17.80
11	−2	3	11.20	10.00
11	2	3	21.60	11.80
−11	2	−3	−2.60	16.70
−11	−3	3	−16.00	18.90
−11	−3	3	5.90	17.00
11	−3	−3	−13.80	10.50
11	−3	−3	21.90	16.30
−11	3	3	−23.40	17.20
−11	3	3	−4.20	20.50
−11	3	3	22.00	20.90
10	−3	−3	−6.80	7.70
10	−3	−3	−9.80	11.30
−10	−3	3	−14.40	14.90
−10	−3	3	2.20	16.10
−10	3	3	4.80	13.40
−10	3	3	−16.30	15.80
−10	3	3	8.80	18.80
10	3	−3	0.00	8.50
9	−3	−3	1.90	6.90
−9	−3	3	−17.40	14.40
−9	−3	3	−10.80	14.30

9	−3	−3	−15.20	10.10
9	3	−3	0.90	6.70
−9	3	3	7.00	13.10
−9	3	3	14.60	13.90
−9	3	3	−1.30	13.30
−8	−3	3	15.50	12.80
−8	−3	3	17.80	13.80
8	−3	−3	−0.60	5.80
8	−3	−3	−1.50	7.40
−8	3	3	−2.40	11.40
8	3	−3	6.40	6.80
−8	3	3	3.70	10.90
−8	3	3	−1.10	13.00
−7	−3	3	35.20	14.30
7	−3	−3	20.60	6.60
−7	−3	3	48.10	15.60
7	−3	−3	33.70	12.00
−7	3	3	38.10	11.70
7	3	−3	50.39	10.00
−7	3	3	24.90	11.00
−7	3	3	24.30	13.50
−6	−3	3	45.00	10.40
−6	−3	3	45.20	12.20
6	−3	−3	23.10	8.10
6	−3	−3	47.20	8.10
−6	−3	3	44.60	14.50
6	3	−3	44.40	8.10
−6	3	3	45.10	12.50
−6	3	3	48.50	15.80
−6	3	3	18.40	10.20
5	−3	−3	154.28	14.30

−5	−3	3	145.29	13.40
5	−3	−3	185.48	26.40
−5	−3	3	141.99	19.00
−5	−3	3	119.39	17.90
5	−3	−3	152.88	11.30
5	3	−3	151.88	11.20
−5	3	3	186.98	23.90
−5	3	3	147.69	18.90
−5	3	3	116.99	16.60
−4	−3	3	152.68	15.50
4	−3	−3	162.08	10.60
4	−3	−3	140.29	12.50
4	−3	−3	123.19	18.00
−4	−3	3	151.18	20.40
−4	−3	3	139.79	10.90
−4	−3	3	124.09	8.90
−4	3	3	133.29	10.10
4	3	−3	144.59	20.40
4	3	−3	147.59	11.70
−4	3	3	125.19	13.50
4	3	−3	136.29	9.80
−4	3	3	131.89	16.40
−4	3	3	144.29	19.80
3	−3	−3	71.59	11.90
−3	−3	3	78.99	6.60
3	−3	−3	80.89	13.50
−3	−3	3	58.29	9.50
3	−3	−3	55.89	9.30
−3	−3	3	65.59	5.90
−3	−3	3	60.29	12.10
3	3	−3	61.89	12.00

3	3	−3	49.50	7.50
3	3	−3	75.59	6.30
3	3	−3	63.29	10.10
−3	3	3	66.59	10.00
−3	3	3	71.69	12.40
−3	3	3	58.69	11.70
2	−3	−3	4.40	3.60
−2	−3	3	3.20	1.70
−2	−3	3	4.90	5.10
2	−3	−3	12.60	6.20
−2	−3	3	9.80	4.00
2	−3	−3	1.00	5.00
2	−3	−3	7.90	2.90
2	−3	−3	7.40	3.80
−2	−3	3	4.50	2.10
2	3	−3	3.70	4.30
2	3	−3	6.40	3.40
2	3	−3	3.00	4.00
2	3	−3	6.50	2.10
−2	3	3	5.90	3.50
−2	3	3	0.90	3.90
−2	3	3	1.90	3.50
−2	3	3	1.10	4.10
−2	3	3	6.80	5.20
1	−3	−3	73.79	9.20
1	−3	−3	74.09	11.10
−1	−3	3	71.89	7.20
1	−3	−3	75.09	7.40
1	−3	−3	70.19	6.20
1	−3	−3	89.69	12.40
1	3	−3	71.49	11.00

-1	3	3	53.59	9.00
-1	3	3	66.59	6.00
1	3	-3	85.09	6.90
1	3	-3	72.99	6.80
-1	3	3	69.99	11.50
1	3	-3	65.39	4.80
-1	3	3	81.69	9.60
0	-3	-3	689.73	22.80
0	-3	-3	718.23	28.30
0	-3	-3	663.33	28.70
0	-3	-3	717.33	21.60
0	-3	-3	670.83	24.50
0	-3	3	657.43	21.40
0	3	3	722.73	30.00
0	3	-3	724.93	16.50
0	3	-3	715.13	19.70
0	3	-3	691.03	20.90
0	3	3	672.63	28.50
0	3	3	694.53	21.70
0	3	3	733.93	24.90
0	3	-3	730.83	22.20
0	3	3	675.43	21.30
-1	-3	-3	118.39	10.90
1	-3	3	93.49	4.60
1	-3	3	83.69	5.90
-1	-3	-3	104.69	8.00
-1	-3	-3	90.39	11.70
-1	-3	-3	106.39	10.30
-1	-3	-3	91.59	6.60
-1	-3	-3	92.69	6.00
-1	-3	-3	89.09	8.10

-1	3	-3	99.29	6.30
-1	3	-3	104.89	9.80
1	3	3	104.09	10.20
1	3	3	87.09	6.30
1	3	3	97.49	7.60
1	3	3	97.49	7.90
1	3	3	97.29	6.60
-2	-3	-3	374.16	18.70
-2	-3	-3	392.66	20.70
2	-3	3	361.66	12.10
2	-3	3	365.76	12.10
-2	-3	-3	351.96	19.30
-2	-3	-3	332.87	14.80
-2	-3	-3	347.17	13.60
-2	3	-3	366.56	12.10
-2	3	-3	375.06	12.00
-2	3	-3	358.86	9.40
2	3	3	343.97	19.00
-2	3	-3	382.26	19.90
-2	3	-3	365.46	11.20
2	3	3	356.26	18.20
2	3	3	353.66	14.10
2	3	3	347.37	15.20
-2	3	-3	365.66	9.00
-3	-3	-3	11.50	3.90
-3	-3	-3	16.60	5.60
3	-3	3	11.70	6.20
-3	-3	-3	10.00	5.80
3	-3	3	10.40	2.00
-3	-3	-3	16.30	3.90
3	-3	3	10.30	4.00

−3	3	−3	15.60	4.00
3	3	3	25.00	7.30
−3	3	−3	13.50	2.60
3	3	3	13.90	3.70
3	3	3	15.90	3.90
3	3	3	10.60	5.50
3	3	3	11.70	5.00
−3	3	−3	13.30	2.60
3	3	3	17.00	3.90
−3	3	−3	13.70	2.40
−3	3	−3	8.00	5.20
−4	−3	−3	0.60	3.80
4	−3	3	5.70	3.80
−4	−3	−3	5.40	3.10
−4	−3	−3	2.90	5.40
4	−3	3	−5.10	5.70
−4	3	−3	−0.10	5.40
4	3	3	1.50	4.60
−4	3	−3	5.60	3.90
4	3	3	8.30	5.60
−4	3	−3	8.50	2.60
4	3	3	2.00	5.90
4	3	3	5.40	3.60
4	3	3	−17.40	6.20
4	3	3	0.80	2.90
5	−3	3	45.70	4.30
−5	−3	−3	52.09	8.50
−5	−3	−3	55.39	12.50
−5	−3	−3	39.20	10.00
5	−3	3	44.50	4.50
5	3	3	37.00	6.50

−5	3	−3	48.90	6.20
5	3	3	35.20	6.30
−5	3	−3	36.80	8.50
6	−3	3	24.40	4.50
−6	−3	−3	12.90	8.40
6	−3	3	19.80	4.00
−6	−3	−3	19.40	9.30
−6	−3	−3	21.20	5.30
−6	3	−3	7.40	6.00
−6	3	−3	19.80	9.40
6	3	3	18.50	5.60
6	3	3	12.00	5.30
7	−3	3	13.30	4.70
−7	−3	−3	13.70	11.30
−7	−3	−3	31.80	12.10
7	−3	3	14.40	4.50
−7	3	−3	19.60	8.80
−7	3	−3	9.10	9.70
7	3	3	9.40	6.50
7	3	3	23.90	7.40
−8	−3	−3	12.80	11.40
−8	−3	−3	26.40	13.50
8	−3	3	18.50	5.90
8	3	3	27.20	8.10
8	3	3	15.50	7.70
−8	3	−3	22.90	11.10
−8	3	−3	38.10	15.20
−9	−3	−3	−17.20	10.70
9	−3	3	4.30	6.20
−9	−3	−3	16.40	14.10
−9	3	−3	−7.30	10.80

−9	3	−3	6.80	14.40
9	3	3	4.30	7.70
10	−3	3	−7.00	7.50
−10	−3	−3	−5.10	16.70
−10	−3	−3	22.40	15.50
10	3	3	9.50	8.50
−10	3	−3	15.20	17.30
−10	3	−3	1.30	13.20
−11	−3	−3	−27.60	17.70
11	−3	3	−6.30	9.40
−11	−3	−3	−18.80	18.20
11	3	3	−9.60	11.60
−11	3	−3	1.90	16.30
11	−4	−3	−21.40	14.40
−11	−4	3	28.60	24.30
−11	−4	3	−9.90	22.60
11	−4	−3	0.30	11.40
11	4	−3	0.50	11.60
−11	4	3	−12.00	20.00
−11	4	3	19.70	20.60
−10	−4	3	−22.10	16.50
10	−4	−3	8.70	12.60
−10	−4	3	−8.10	15.80
10	−4	−3	−11.10	9.40
−10	4	3	−1.30	15.20
10	4	−3	−5.10	8.40
−10	4	3	25.40	19.10
−10	4	3	2.60	18.80
−9	−4	3	23.40	19.30
−9	−4	3	46.70	18.70
9	−4	−3	36.70	11.70

9	−4	−3	28.10	9.30
9	4	−3	43.10	10.80
−9	4	3	27.60	16.20
−9	4	3	52.89	16.10
−9	4	3	37.50	17.80
−8	−4	3	4.60	13.50
8	−4	−3	16.30	9.20
8	−4	−3	14.40	7.70
−8	4	3	44.70	16.30
8	4	−3	19.60	9.50
−8	4	3	20.70	13.00
−8	4	3	19.50	14.20
7	−4	−3	50.29	9.60
−7	−4	3	94.09	20.70
7	−4	−3	74.29	12.80
−7	−4	3	65.39	17.30
−7	4	3	66.79	15.80
7	4	−3	67.29	10.70
−7	4	3	84.69	17.50
−7	4	3	89.09	21.00
6	−4	−3	158.28	15.40
−6	−4	3	143.89	22.30
−6	−4	3	117.39	21.60
6	−4	−3	133.79	12.00
−6	−4	3	96.09	14.60
−6	4	3	113.39	21.30
6	4	−3	124.29	13.80
−6	4	3	111.29	18.10
−6	4	3	125.49	19.00
−5	−4	3	410.86	29.60
−5	−4	3	337.47	25.40

5	−4	−3	316.97	20.50
5	−4	−3	386.76	25.40
−5	−4	3	336.07	20.20
5	−4	−3	387.16	18.00
5	4	−3	373.96	17.90
−5	4	3	400.46	27.10
−5	4	3	383.16	25.10
−5	4	3	387.36	31.00
−4	−4	3	27.60	5.10
−4	−4	3	20.00	9.00
4	−4	−3	17.20	4.90
4	−4	−3	21.00	7.10
−4	−4	3	35.60	6.50
4	−4	−3	16.50	8.10
−4	−4	3	26.90	8.20
−4	4	3	12.00	7.20
−4	4	3	3.80	6.40
4	4	−3	22.90	5.00
−4	4	3	13.00	5.10
−4	4	3	29.40	9.00
−3	−4	3	11.00	3.30
3	−4	−3	22.60	6.40
−3	−4	3	7.70	3.50
3	−4	−3	4.80	4.10
3	−4	−3	1.20	7.60
−3	−4	3	16.40	7.70
−3	−4	3	11.40	5.50
3	−4	−3	4.70	7.00
−3	4	3	−3.80	4.70
−3	4	3	−10.30	5.70
3	4	−3	9.00	3.40

3	4	−3	−0.30	5.60
−3	4	3	7.10	3.70
3	4	−3	2.60	4.50
−3	4	3	7.90	5.10
−3	4	3	6.10	4.90
3	4	−3	10.40	5.70
2	−4	−3	291.27	15.20
−2	−4	3	312.77	11.20
2	−4	−3	312.17	24.00
2	−4	−3	319.37	17.80
2	−4	−3	338.77	23.90
−2	−4	3	312.17	12.10
−2	−4	3	303.97	15.50
−2	−4	3	323.47	19.20
−2	−4	3	310.17	20.50
2	−4	−3	327.27	14.10
−2	4	3	290.47	22.70
−2	4	3	315.77	21.30
2	4	−3	303.77	16.80
−2	4	3	324.97	18.30
2	4	−3	300.17	20.40
−2	4	3	327.17	18.20
−2	4	3	286.97	14.00
2	4	−3	318.77	15.80
2	4	−3	308.57	12.00
1	−4	−3	24.70	4.20
−1	−4	3	18.00	4.30
1	−4	−3	28.40	7.10
−1	−4	3	25.90	3.70
−1	−4	3	24.00	2.60
1	−4	−3	34.10	10.10

1	−4	−3	27.00	8.20
1	4	−3	22.50	5.10
1	4	−3	27.90	3.50
1	4	−3	29.20	3.80
−1	4	3	18.90	5.30
1	4	−3	20.00	4.30
1	4	−3	23.30	7.60
−1	4	3	28.00	4.30
0	−4	−3	1197.08	44.60
0	−4	−3	1208.38	41.20
0	−4	−3	1199.18	32.20
0	−4	3	1152.18	33.60
0	−4	−3	1094.69	39.90
0	−4	−3	1221.78	32.20
0	4	3	1164.98	32.90
0	4	3	1172.98	32.40
0	4	−3	1191.88	26.30
0	4	−3	1168.48	33.70
0	4	3	1164.98	49.30
0	4	3	1183.38	36.60
0	4	−3	1144.09	34.80
0	4	−3	1177.68	29.30
−1	−4	−3	46.60	5.40
−1	−4	−3	37.00	7.70
−1	−4	−3	45.90	9.10
−1	−4	−3	37.60	5.90
−1	−4	−3	35.00	8.10
−1	−4	−3	33.70	6.90
1	−4	3	41.90	5.10
1	4	3	40.40	6.50
1	4	3	47.80	5.90

1	4	3	36.40	5.80
−1	4	−3	39.60	5.30
−1	4	−3	42.50	7.40
1	4	3	36.60	5.90
−1	4	−3	39.10	6.80
−2	−4	−3	293.77	13.30
2	−4	3	283.97	8.70
−2	−4	−3	282.37	16.30
2	−4	3	289.97	12.20
−2	4	−3	323.97	18.10
2	4	3	316.07	15.50
2	4	3	284.27	18.60
2	4	3	318.07	13.60
2	4	3	334.97	14.00
2	4	3	324.97	19.80
2	4	3	293.67	16.50
−2	4	−3	300.57	12.30
3	−4	3	132.89	8.40
−3	−4	−3	153.48	13.90
−3	−4	−3	140.79	17.20
−3	−4	−3	134.69	11.80
−3	−4	−3	146.69	9.30
3	−4	3	142.79	15.10
3	4	3	132.49	14.00
−3	4	−3	148.09	5.90
3	4	3	158.68	9.80
3	4	3	134.39	10.10
−3	4	−3	130.19	7.70
3	4	3	144.79	13.70
−3	4	−3	133.69	7.70
3	4	3	131.49	15.60

−3	4	−3	139.19	5.70
3	4	3	146.59	9.70
−3	4	−3	140.49	14.00
4	−4	3	168.98	17.50
−4	−4	−3	157.18	19.10
4	−4	3	158.38	7.10
−4	−4	−3	144.19	10.90
4	−4	3	105.79	10.40
−4	−4	−3	144.69	15.70
4	4	3	138.39	11.00
4	4	3	140.99	11.70
4	4	3	138.49	15.40
4	4	3	147.89	14.90
−4	4	−3	142.89	15.40
−4	4	−3	161.88	11.20
−4	4	−3	163.28	8.50
−4	4	−3	155.58	7.80
−5	−4	−3	2.10	7.70
5	−4	3	−0.20	1.70
−5	−4	−3	3.40	4.10
−5	−4	−3	8.60	7.60
−5	4	−3	4.30	3.40
5	4	3	1.80	3.50
5	4	3	6.50	5.90
5	4	3	0.40	4.30
−5	4	−3	1.30	4.60
−6	−4	−3	170.48	12.70
−6	−4	−3	189.78	25.10
−6	−4	−3	205.08	25.30
6	4	3	202.48	14.20
−6	4	−3	178.68	19.40

−6	4	−3	156.68	22.10
6	4	3	170.38	13.30
−7	−4	−3	9.90	12.30
−7	−4	−3	15.60	7.10
7	−4	3	14.80	3.70
−7	−4	−3	14.70	11.20
−7	4	−3	14.20	12.70
7	4	3	18.90	7.20
−7	4	−3	18.20	8.30
8	−4	3	5.90	5.30
−8	−4	−3	−14.60	15.20
−8	−4	−3	11.90	13.00
8	4	3	6.20	6.80
−8	4	−3	15.00	10.60
−8	4	−3	21.70	12.40
−9	−4	−3	3.30	14.70
−9	−4	−3	29.40	15.50
9	−4	3	0.80	5.60
−9	4	−3	8.80	11.40
9	4	3	−7.30	7.50
−9	4	−3	−0.60	15.00
−10	−4	−3	−8.20	17.70
10	−4	3	1.60	7.10
−10	−4	−3	−1.50	16.50
−10	4	−3	21.50	18.10
10	4	3	−6.30	9.20
−10	4	−3	16.30	14.70
−10	4	−3	−4.10	19.00
−11	−4	−3	−2.60	19.80
−11	−4	−3	15.70	23.80
−11	4	−3	19.60	16.50

11	4	3	-2.30	11.60
-11	4	-3	-26.70	22.60
-11	-5	3	30.00	22.80
-11	-5	3	51.49	26.80
11	-5	-3	0.90	11.50
-11	5	3	35.70	21.20
-11	5	3	-3.90	21.60
-11	5	3	5.10	19.80
11	5	-3	1.40	10.80
-10	-5	3	18.40	22.20
-10	-5	3	-9.00	18.30
10	-5	-3	36.60	13.70
10	-5	-3	19.90	9.80
10	5	-3	7.40	9.40
-10	5	3	46.30	25.30
-10	5	3	-0.50	19.20
-10	5	3	18.80	17.40
-9	-5	3	-6.00	14.80
9	-5	-3	-1.70	10.00
-9	-5	3	6.60	15.60
9	-5	-3	-2.00	7.60
9	5	-3	0.70	7.40
-9	5	3	16.40	16.70
-9	5	3	-6.10	15.00
-9	5	3	-13.50	14.00
8	-5	-3	87.59	12.30
8	-5	-3	95.99	15.90
-8	-5	3	106.09	26.60
-8	-5	3	70.89	20.60
8	5	-3	118.19	15.00
-8	5	3	103.99	21.20

−8	5	3	95.39	21.50
−8	5	3	80.79	21.60
−7	−5	3	7.50	11.00
7	−5	−3	16.40	6.80
−7	−5	3	4.40	11.80
7	−5	−3	2.80	7.50
−7	5	3	3.40	10.00
−7	5	3	12.00	13.40
7	5	−3	7.20	7.50
−7	5	3	17.70	11.50
6	−5	−3	−7.50	5.50
−6	−5	3	−3.10	8.00
−6	−5	3	−5.80	9.80
−6	−5	3	0.50	8.40
−6	5	3	−7.90	7.10
6	5	−3	−3.90	4.50
−6	5	3	−3.10	6.80
−6	5	3	−7.90	8.20
5	−5	−3	90.69	14.90
5	−5	−3	96.59	10.90
5	−5	−3	90.29	13.60
−5	−5	3	100.49	12.60
−5	−5	3	62.89	13.00
−5	−5	3	113.99	20.10
−5	5	3	107.19	16.70
5	5	−3	89.99	11.20
−5	5	3	90.59	20.50
−5	5	3	83.99	15.40
4	−5	−3	977.10	37.40
−4	−5	3	934.81	45.50
−4	−5	3	877.91	28.60

−4	−5	3	974.70	41.20
−4	−5	3	971.90	32.20
4	−5	−3	991.50	31.10
4	−5	−3	921.91	37.00
−4	5	3	927.41	42.70
4	5	−3	933.01	30.50
−4	5	3	986.60	43.20
−4	5	3	1006.10	49.40
−3	−5	3	1.80	3.60
−3	−5	3	0.30	2.80
3	−5	−3	−1.10	4.60
3	−5	−3	−0.30	3.40
−3	−5	3	−1.30	4.60
−3	−5	3	0.20	3.50
3	−5	−3	−2.10	4.60
−3	−5	3	1.10	5.10
−3	5	3	−1.70	3.90
−3	5	3	12.30	7.70
−3	5	3	−3.80	5.90
−3	5	3	1.60	4.90
3	5	−3	3.50	3.20
3	5	−3	2.40	6.80
3	5	−3	7.40	5.30
−3	5	3	7.00	4.70
2	−5	−3	2094.99	67.09
−2	−5	3	2192.98	54.79
2	−5	−3	2065.69	52.89
−2	−5	3	2103.59	57.99
2	−5	−3	2124.09	61.39
−2	−5	3	2171.38	64.29
2	−5	−3	2062.29	53.19

2	−5	−3	1955.90	72.69
−2	−5	3	2109.49	48.70
−2	5	3	2034.70	56.99
2	5	−3	2071.39	50.69
−2	5	3	2022.50	73.29
2	5	−3	2158.28	66.69
−2	5	3	2151.38	61.59
2	5	−3	2146.19	58.09
2	5	−3	2074.99	50.89
−2	5	3	2140.69	67.99
2	5	−3	2110.09	58.59
1	−5	−3	333.67	13.80
−1	−5	3	322.47	16.40
1	−5	−3	332.47	21.40
1	−5	−3	314.87	12.00
−1	−5	3	355.46	15.20
1	−5	−3	300.07	17.70
−1	−5	3	334.67	8.80
−1	−5	3	310.27	15.50
1	−5	−3	359.16	25.20
−1	5	3	348.97	18.40
−1	5	3	366.56	25.90
1	5	−3	333.17	11.40
1	5	−3	334.97	15.30
1	5	−3	351.36	21.80
1	5	−3	320.77	13.80
−1	5	3	351.26	14.60
1	5	−3	347.77	16.80
−1	5	3	323.37	21.70
0	−5	−3	57.49	5.30
0	−5	−3	68.99	9.50

0	−5	−3	76.29	12.90
0	−5	3	83.09	10.30
0	−5	−3	60.99	11.90
0	5	3	59.59	6.50
0	5	−3	54.09	5.20
0	5	−3	63.39	7.70
0	5	3	48.50	11.80
0	5	−3	58.29	3.40
0	5	3	58.69	10.60
0	5	−3	50.09	8.20
0	5	3	61.99	8.70
−1	−5	−3	4.20	3.30
−1	−5	−3	2.30	3.00
−1	−5	−3	2.70	2.80
−1	−5	−3	5.00	5.60
−1	−5	−3	8.40	3.40
−1	−5	−3	3.70	4.60
−1	−5	−3	4.20	5.60
1	−5	3	−1.30	2.20
1	5	3	0.80	3.20
−1	5	−3	1.50	4.60
1	5	3	−1.40	2.60
1	5	3	5.10	5.80
1	5	3	3.90	3.10
−1	5	−3	6.60	3.00
1	5	3	7.10	3.60
−1	5	−3	0.70	4.80
−2	−5	−3	934.81	28.10
−2	−5	−3	944.41	29.40
−2	−5	−3	936.11	38.30
2	−5	3	950.00	28.70

−2	−5	−3	920.71	42.10
−2	−5	−3	903.81	37.60
−2	−5	−3	898.11	32.20
−2	−5	−3	943.71	32.80
2	5	3	974.30	39.60
2	5	3	930.41	29.40
2	5	3	985.80	33.00
−2	5	−3	949.31	28.40
2	5	3	906.61	28.60
−2	5	−3	966.30	35.30
2	5	3	924.61	32.50
−3	−5	−3	3.50	5.90
3	−5	3	3.30	2.80
−3	−5	−3	−8.80	4.20
3	−5	3	−1.50	2.40
−3	−5	−3	−3.20	3.40
3	5	3	−3.30	3.60
3	5	3	−0.60	4.20
−3	5	−3	−2.00	4.80
−3	5	−3	3.10	3.00
3	5	3	−1.70	2.90
−3	5	−3	1.80	5.90
3	5	3	5.20	6.60
3	5	3	0.60	5.20
−3	5	−3	2.50	1.60
4	−5	3	871.41	39.00
−4	−5	−3	848.02	38.30
−4	−5	−3	852.51	31.30
4	−5	3	885.11	25.10
−4	−5	−3	845.32	40.40
4	−5	3	833.22	27.30

−4	5	−3	819.72	23.00
4	5	3	847.42	29.40
−4	5	−3	868.51	26.50
4	5	3	885.41	31.60
−4	5	−3	785.72	46.80
4	5	3	813.82	37.10
−4	5	−3	858.81	37.30
−4	5	−3	827.62	31.20
−5	−5	−3	6.90	8.80
−5	−5	−3	11.10	9.10
−5	−5	−3	1.50	4.50
5	5	3	2.50	6.00
−5	5	−3	−0.10	4.20
5	5	3	1.80	3.70
−5	5	−3	−1.90	3.80
−5	5	−3	−6.30	6.30
−5	5	−3	−5.50	6.80
−6	−5	−3	86.69	19.20
6	−5	3	83.19	5.20
−6	−5	−3	75.49	12.40
−6	−5	−3	148.69	23.90
−6	5	−3	69.29	11.80
6	5	3	124.39	13.90
−6	5	−3	67.59	14.50
−7	−5	−3	5.50	9.40
−7	−5	−3	−4.60	10.60
−7	−5	−3	10.50	6.40
7	5	3	2.70	6.60
−7	5	−3	11.20	10.70
−7	5	−3	−10.50	9.50
−7	5	−3	10.00	7.90

−8	−5	−3	12.60	12.60
−8	5	−3	41.00	12.80
−8	5	−3	11.20	14.00
8	5	3	27.00	9.50
−8	5	−3	41.50	16.30
−9	−5	−3	−1.60	13.90
−9	−5	−3	−13.40	14.30
−9	5	−3	−12.30	15.70
−9	5	−3	−1.80	10.80
−9	5	−3	1.10	13.60
9	5	3	−1.10	8.70
−10	−5	−3	−8.90	17.60
−10	−5	−3	−4.50	21.50
−10	5	−3	4.30	16.30
−10	5	−3	15.80	20.50
10	5	3	31.20	12.20
−10	5	−3	29.90	14.50
−11	−5	−3	13.20	23.00
−11	−5	−3	−36.60	20.30
−11	5	−3	−2.80	22.20
−11	5	−3	−8.70	16.30
−11	5	−3	−28.50	21.10
−10	−6	3	11.00	20.60
10	−6	−3	3.30	10.50
−10	−6	3	−8.30	18.70
10	−6	−3	−3.30	12.90
−10	6	3	−1.10	17.10
10	6	−3	22.10	12.40
−10	6	3	−10.90	22.30
−10	6	3	−11.10	17.90
−9	−6	3	10.40	19.50

9	−6	−3	3.30	11.80
−9	−6	3	4.30	16.80
9	−6	−3	4.90	9.90
−9	6	3	9.20	16.80
−9	6	3	32.00	20.70
−9	6	3	3.70	19.70
9	6	−3	15.90	9.00
8	−6	−3	44.70	9.70
8	−6	−3	12.60	9.60
−8	−6	3	24.30	17.10
−8	−6	3	23.40	17.00
−8	6	3	34.60	17.10
−8	6	3	27.20	15.00
−8	6	3	52.09	17.50
8	6	−3	20.50	8.90
−7	−6	3	−9.00	10.40
7	−6	−3	9.10	7.10
−7	−6	3	4.20	11.90
7	−6	−3	9.40	9.00
7	6	−3	15.20	8.70
−7	6	3	−15.50	10.70
−7	6	3	3.20	10.50
−7	6	3	−2.10	11.40
6	−6	−3	215.68	19.10
−6	−6	3	241.98	20.10
−6	−6	3	275.47	25.80
6	−6	−3	245.58	15.50
−6	−6	3	280.87	28.40
−6	6	3	233.38	32.00
−6	6	3	251.87	25.50
−6	6	3	231.78	22.40

6	6	-3	240.08	17.10
-5	-6	3	65.79	10.80
-5	-6	3	56.49	16.30
5	-6	-3	69.19	8.90
-5	-6	3	44.40	9.80
5	-6	-3	35.60	9.70
5	-6	-3	49.00	12.30
-5	6	3	55.19	12.40
-5	6	3	50.39	16.90
-5	6	3	37.80	13.00
5	6	-3	59.09	10.10
-4	-6	3	103.89	13.10
4	-6	-3	105.39	14.40
-4	-6	3	142.39	12.60
4	-6	-3	133.49	14.50
-4	-6	3	126.99	23.80
-4	-6	3	144.39	18.90
-4	-6	3	135.09	9.80
4	-6	-3	120.09	9.70
-4	6	3	90.39	18.90
4	6	-3	122.79	10.40
-4	6	3	128.99	13.20
-4	6	3	109.79	17.40
3	-6	-3	221.98	14.30
-3	-6	3	235.88	15.90
3	-6	-3	214.28	13.20
-3	-6	3	219.58	14.60
-3	-6	3	223.58	18.10
-3	-6	3	220.58	10.80
-3	-6	3	189.98	19.60
3	-6	-3	203.48	16.40

3	6	−3	213.68	17.30
3	6	−3	181.28	19.30
−3	6	3	223.48	14.40
−3	6	3	190.78	26.00
3	6	−3	197.58	11.60
3	6	−3	227.08	12.50
−3	6	3	219.38	14.30
−3	6	3	200.58	26.50
−2	−6	3	189.98	8.40
−2	−6	3	197.58	13.80
−2	−6	3	200.68	15.00
−2	−6	3	205.58	13.70
2	−6	−3	157.78	12.10
2	−6	−3	185.68	15.00
2	6	−3	197.48	11.10
−2	6	3	187.88	18.20
−2	6	3	197.18	12.50
2	6	−3	205.08	15.00
2	6	−3	170.48	17.30
−2	6	3	251.77	24.90
1	−6	−3	248.08	27.10
−1	−6	3	245.78	14.80
−1	−6	3	231.18	13.90
1	−6	−3	241.68	16.40
−1	−6	3	216.58	14.10
1	−6	−3	196.28	17.60
1	6	−3	235.28	14.70
−1	6	3	264.47	23.60
−1	6	3	247.18	12.60
1	6	−3	214.28	17.40
−1	6	3	220.78	19.40

1	6	-3	227.08	13.10
-1	6	3	219.68	13.10
1	6	-3	224.78	9.70
1	6	-3	257.47	13.70
0	-6	3	1492.65	45.70
0	-6	-3	1476.95	51.69
0	-6	-3	1529.15	48.50
0	-6	-3	1523.65	40.30
0	-6	-3	1491.45	57.79
0	-6	3	1544.95	45.40
0	6	-3	1582.74	35.10
0	6	3	1497.65	41.00
0	6	-3	1468.65	49.30
0	6	3	1421.66	57.49
0	6	-3	1451.65	49.80
0	6	-3	1586.44	38.60
0	6	-3	1523.15	44.50
0	6	3	1494.55	42.50
-1	-6	-3	679.13	34.60
1	-6	3	700.93	18.40
-1	-6	-3	649.64	22.90
-1	-6	-3	695.53	23.40
-1	-6	-3	693.43	32.50
-1	-6	-3	724.83	29.00
1	-6	3	712.43	25.90
1	6	3	721.53	24.20
-1	6	-3	733.83	32.80
1	6	3	679.63	22.90
1	6	3	695.03	35.70
-1	6	-3	727.43	29.60
-1	6	-3	682.73	25.20

1	6	3	717.03	24.80
-2	-6	-3	762.92	24.50
-2	-6	-3	739.33	28.40
-2	-6	-3	722.53	34.30
-2	-6	-3	751.22	29.50
2	6	3	748.03	26.00
2	6	3	681.53	34.60
-2	6	-3	713.33	30.00
-2	6	-3	705.23	25.30
2	6	3	679.23	27.20
-2	6	-3	711.43	34.50
-3	-6	-3	56.69	9.50
-3	-6	-3	39.70	10.20
3	-6	3	35.50	4.20
3	-6	3	38.90	6.20
-3	-6	-3	40.80	10.30
-3	6	-3	50.89	12.60
-3	6	-3	42.90	6.40
3	6	3	21.00	8.80
3	6	3	49.00	10.70
3	6	3	33.50	6.70
3	6	3	37.90	9.60
-3	6	-3	20.10	8.20
-4	-6	-3	41.60	10.30
-4	-6	-3	55.39	15.20
4	-6	3	38.50	5.90
4	-6	3	73.39	14.00
4	-6	3	39.40	6.60
-4	6	-3	54.39	14.10
-4	6	-3	43.50	10.60
-4	6	-3	33.00	4.70

4	6	3	41.80	7.70
4	6	3	48.40	12.50
4	6	3	44.70	11.80
−4	6	−3	28.90	6.10
−4	6	−3	46.00	5.70
5	−6	3	7.80	4.00
−5	−6	−3	4.80	7.30
−5	−6	−3	−6.40	8.60
−5	−6	−3	5.90	5.80
5	6	3	3.20	3.70
−5	6	−3	0.80	7.90
−5	6	−3	17.30	11.90
−5	6	−3	11.40	6.00
5	6	3	12.50	9.40
−5	6	−3	12.70	4.80
−5	6	−3	11.80	4.50
−6	−6	−3	156.88	24.00
−6	−6	−3	180.78	25.60
6	−6	3	169.88	9.70
−6	−6	−3	194.98	16.00
6	6	3	162.58	14.40
−6	6	−3	201.28	21.10
−6	6	−3	163.68	15.60
6	6	3	168.58	23.30
−6	6	−3	229.48	29.60
−7	−6	−3	24.60	14.50
−7	−6	−3	27.60	16.10
−7	−6	−3	22.40	7.70
7	−6	3	25.30	3.80
−7	6	−3	15.40	12.60
−7	6	−3	34.20	10.10

−7	6	−3	4.10	10.00
7	6	3	33.70	8.90
−8	−6	−3	10.90	13.40
−8	−6	−3	29.90	15.70
−8	−6	−3	15.60	9.20
−8	6	−3	26.10	13.90
8	6	3	24.40	9.50
−8	6	−3	26.00	11.00
−8	6	−3	27.20	17.90
−9	−6	−3	24.20	19.10
−9	−6	−3	−20.20	14.00
−9	6	−3	12.40	13.00
9	6	3	−2.10	9.00
−9	6	−3	32.80	18.00
−9	6	−3	5.10	11.40
−10	−6	−3	−6.40	20.20
−10	−6	−3	20.90	19.70
−10	6	−3	16.00	18.50
−10	6	−3	−2.00	13.20
10	6	3	2.30	11.10
−10	6	−3	12.50	24.40
−10	−7	3	6.90	20.00
−10	−7	3	35.20	21.90
10	−7	−3	−5.70	14.80
−10	7	3	27.30	19.10
10	7	−3	10.80	10.70
−10	7	3	13.00	22.90
−10	7	3	13.20	21.10
−9	−7	3	−25.90	15.90
9	−7	−3	16.00	12.40
−9	−7	3	22.00	20.10

9	−7	−3	4.40	8.80
9	7	−3	2.50	9.20
−9	7	3	24.60	19.30
−9	7	3	16.30	18.60
8	−7	−3	6.70	7.60
−8	−7	3	24.10	17.20
8	−7	−3	6.60	10.20
−8	−7	3	−9.30	15.70
−8	7	3	10.90	16.90
8	7	−3	−0.70	8.10
−8	7	3	5.90	13.70
−8	7	3	14.30	16.20
−7	−7	3	56.19	20.20
−7	−7	3	32.00	15.40
7	−7	−3	42.00	8.80
7	−7	−3	65.79	14.40
−7	7	3	20.20	13.30
−7	7	3	33.60	16.20
−7	7	3	39.60	14.30
7	7	−3	34.50	14.80
6	−7	−3	33.10	10.50
−6	−7	3	9.40	11.60
−6	−7	3	20.20	12.30
−6	−7	3	15.30	16.00
6	−7	−3	35.60	7.20
6	7	−3	29.00	7.70
−6	7	3	−1.60	11.80
−6	7	3	28.10	13.70
−6	7	3	24.40	13.30
5	−7	−3	63.99	8.30
−5	−7	3	53.49	18.30

−5	−7	3	52.79	14.00
5	−7	−3	50.99	12.00
5	−7	−3	72.29	13.60
−5	7	3	88.89	19.10
−5	7	3	41.90	15.80
−5	7	3	67.99	11.90
5	7	−3	52.99	8.90
−4	−7	3	188.68	15.70
4	−7	−3	175.18	11.00
−4	−7	3	186.48	21.30
4	−7	−3	181.88	17.60
−4	−7	3	168.98	15.40
−4	−7	3	197.78	25.80
−4	−7	3	169.58	11.20
4	−7	−3	199.18	15.90
−4	7	3	162.88	14.50
−4	7	3	221.78	26.30
4	7	−3	200.78	13.50
−4	7	3	155.38	24.40
−3	−7	3	66.29	11.70
−3	−7	3	80.39	6.70
−3	−7	3	80.09	12.30
−3	−7	3	61.19	10.90
3	−7	−3	64.39	8.60
−3	−7	3	61.99	13.60
3	−7	−3	70.89	11.70
−3	7	3	77.29	15.50
3	7	−3	71.79	14.80
3	7	−3	73.49	8.70
3	7	−3	54.19	8.30
3	7	−3	86.59	13.40

−3	7	3	48.50	15.60
−3	7	3	71.89	9.60
−2	−7	3	47.10	4.70
−2	−7	3	42.70	9.60
−2	−7	3	58.19	10.20
−2	−7	3	33.70	9.10
2	−7	−3	43.50	9.20
2	7	−3	41.30	10.10
2	7	−3	47.50	6.30
2	7	−3	64.49	11.00
−2	7	3	61.19	15.70
2	7	−3	54.29	7.80
−2	7	3	53.09	14.80
1	−7	−3	3.80	4.40
1	−7	−3	−2.20	2.60
−1	−7	3	9.00	6.40
−1	−7	3	0.70	3.90
1	7	−3	3.60	2.70
−1	7	3	0.50	5.70
−1	7	3	3.20	3.90
1	7	−3	2.80	4.20
−1	7	3	0.10	3.90
1	7	−3	1.70	6.40
1	7	−3	4.40	4.20
0	−7	−3	162.18	14.20
0	−7	−3	182.38	20.50
0	−7	3	186.38	13.70
0	−7	−3	197.38	10.80
0	−7	3	191.88	15.40
0	7	−3	167.28	15.70
0	7	3	154.78	11.50

0	7	3	160.38	21.50
0	7	−3	174.58	8.60
0	7	−3	189.78	16.60
1	−7	3	11.50	5.00
1	−7	3	8.20	3.70
−1	−7	−3	9.20	5.00
1	−7	3	12.90	3.90
−1	−7	−3	10.20	3.80
1	7	3	17.60	5.60
−1	7	−3	19.20	8.90
−1	7	−3	3.10	1.80
−1	7	−3	14.70	5.00
1	7	3	4.20	7.50
−1	7	−3	9.20	5.60
−2	−7	−3	190.48	15.10
−2	−7	−3	184.78	18.50
2	−7	3	181.58	13.00
−2	−7	−3	191.78	13.70
−2	−7	−3	167.58	12.90
−2	7	−3	188.28	15.20
−2	7	−3	188.38	19.50
2	7	3	168.88	12.80
−2	7	−3	188.28	13.40
−3	−7	−3	18.90	10.20
3	−7	3	10.40	5.00
−3	−7	−3	10.70	6.00
−3	−7	−3	24.70	8.40
3	7	3	12.40	4.90
−3	7	−3	10.90	5.10
3	7	3	9.80	8.80
−3	7	−3	15.70	8.70

3	7	3	7.30	10.80
3	7	3	22.90	7.40
−3	7	−3	9.20	2.60
−3	7	−3	12.10	7.00
−4	−7	−3	14.40	8.20
−4	−7	−3	20.40	7.20
4	−7	3	15.40	4.90
−4	−7	−3	26.60	11.90
4	−7	3	16.70	5.00
−4	7	−3	26.60	12.80
4	7	3	14.80	9.30
4	7	3	11.30	4.80
4	7	3	3.20	9.60
−4	7	−3	9.10	3.80
−4	7	−3	22.80	9.00
−5	−7	−3	30.50	14.00
−5	−7	−3	18.60	8.90
−5	−7	−3	26.90	7.80
5	7	3	10.00	6.10
−5	7	−3	25.80	6.50
−5	7	−3	13.60	8.80
−5	7	−3	28.50	7.90
5	7	3	26.50	9.80
−5	7	−3	13.60	5.10
−5	7	−3	17.60	13.20
−6	−7	−3	0.90	11.90
−6	−7	−3	−9.60	5.50
−6	−7	−3	5.00	9.70
6	−7	3	−4.90	3.80
6	7	3	−1.70	8.80
−6	7	−3	−8.40	9.40

-6	7	-3	-0.10	6.40
-6	7	-3	1.90	9.20
-6	7	-3	5.30	6.80
6	7	3	-3.10	5.10
-7	-7	-3	9.00	14.10
-7	-7	-3	1.30	6.60
7	-7	3	7.40	4.40
-7	-7	-3	13.90	13.40
7	7	3	-4.90	6.80
-7	7	-3	4.00	8.10
-7	7	-3	6.00	10.60
-7	7	-3	12.80	15.50
-8	-7	-3	-15.90	14.30
-8	-7	-3	13.20	16.50
-8	-7	-3	12.50	8.80
8	7	3	2.20	7.70
-8	7	-3	-15.00	10.00
-8	7	-3	17.50	13.40
-8	7	-3	-5.80	14.90
-9	-7	-3	8.50	19.50
-9	-7	-3	3.00	18.10
-9	7	-3	-15.80	13.10
9	7	3	-1.20	9.00
-9	7	-3	-27.20	14.80
-9	7	-3	-6.00	15.10
-10	-7	-3	7.70	19.20
-10	7	-3	-38.90	21.00
-10	7	-3	-2.20	15.20
-10	7	-3	-15.20	17.90
10	7	3	1.80	11.70
-10	-8	3	-49.90	24.00

10	−8	−3	−23.70	16.10
−10	−8	3	−15.90	23.40
−10	8	3	−55.29	31.70
−10	8	3	9.10	27.30
−10	8	3	14.30	21.00
10	8	−3	11.40	13.30
−9	−8	3	4.20	19.00
−9	−8	3	−5.20	19.40
9	−8	−3	6.70	12.50
−9	8	3	12.50	20.10
9	8	−3	19.70	11.30
−9	8	3	7.20	16.60
−8	−8	3	2.00	17.30
8	−8	−3	−1.00	9.10
8	−8	−3	6.10	6.70
−8	−8	3	3.20	19.50
−8	8	3	−18.20	17.10
−8	8	3	18.50	14.20
8	8	−3	−2.70	7.80
−8	8	3	−7.50	13.60
7	−8	−3	6.40	9.40
7	−8	−3	27.80	7.20
−7	−8	3	15.30	18.30
−7	−8	3	−4.70	13.60
−7	8	3	26.40	12.60
−7	8	3	13.80	17.20
7	8	−3	2.80	8.10
−6	−8	3	−11.30	10.60
6	−8	−3	1.10	8.60
−6	−8	3	23.60	15.50
6	−8	−3	9.00	5.00

−6	8	3	13.80	12.60
6	8	−3	11.90	6.90
−6	8	3	16.60	24.10
−6	8	3	13.00	10.40
5	−8	−3	43.60	11.50
−5	−8	3	45.50	11.10
−5	−8	3	57.99	18.20
5	−8	−3	52.59	10.60
−5	−8	3	48.70	12.20
−5	8	3	28.40	12.80
−5	8	3	28.50	16.60
−5	8	3	41.70	11.60
5	8	−3	67.39	11.00
−4	−8	3	−4.50	6.20
−4	−8	3	−10.90	8.00
4	−8	−3	1.50	6.60
−4	−8	3	−3.70	4.00
4	−8	−3	−1.60	7.50
−4	−8	3	3.10	10.60
−4	−8	3	4.60	8.50
4	8	−3	17.00	11.50
−4	8	3	4.30	9.60
−4	8	3	9.30	12.10
−4	8	3	1.40	5.70
4	8	−3	3.20	4.80
−3	−8	3	2.40	5.80
−3	−8	3	11.00	7.60
−3	−8	3	0.80	3.50
−3	−8	3	−6.60	7.60
3	−8	−3	−3.70	6.50
−3	8	3	4.20	10.20

3	8	−3	0.60	4.40
3	8	−3	5.00	5.10
−3	8	3	6.00	8.40
3	8	−3	13.70	8.20
−2	−8	3	58.69	10.60
−2	−8	3	61.69	11.80
2	−8	−3	68.19	12.10
−2	−8	3	81.49	13.50
2	8	−3	60.49	10.30
2	8	−3	60.59	13.00
2	8	−3	77.59	10.60
−2	8	3	66.69	15.30
−2	8	3	44.50	14.90
2	8	−3	67.09	7.80
1	−8	−3	9.80	3.70
1	−8	−3	4.00	6.20
−1	−8	3	13.10	6.00
−1	−8	3	−3.90	7.20
−1	−8	3	2.80	5.00
1	8	−3	12.10	5.50
−1	8	3	−2.80	6.90
1	8	−3	8.40	5.90
1	8	−3	3.00	2.60
1	8	−3	−0.20	6.80
−1	8	3	2.70	4.30
0	−8	−3	195.98	17.00
0	−8	−3	179.78	21.60
0	−8	3	164.78	17.20
0	−8	3	190.48	15.40
0	8	3	188.88	13.30
0	8	3	164.78	23.70

0	8	-3	196.88	17.40
0	8	-3	204.38	9.20
1	-8	3	13.50	5.00
-1	-8	-3	9.00	6.50
1	-8	3	14.30	6.60
-1	-8	-3	11.90	4.10
-1	-8	-3	-4.70	6.30
1	-8	3	17.30	4.50
-1	8	-3	8.80	7.50
-1	8	-3	11.50	5.90
-1	8	-3	14.30	2.90
1	8	3	18.80	10.20
-2	-8	-3	59.79	9.20
-2	-8	-3	50.29	10.20
2	-8	3	40.70	7.10
-2	-8	-3	76.49	14.90
2	8	3	42.20	8.10
-2	8	-3	41.60	13.60
2	8	3	40.10	13.00
-2	8	-3	38.40	3.30
-2	8	-3	35.40	9.30
2	8	3	13.90	4.90
3	-8	3	110.99	11.00
-3	-8	-3	83.59	15.40
-3	-8	-3	109.99	16.90
-3	-8	-3	101.49	14.00
3	8	3	114.69	19.80
3	8	3	114.39	14.70
-3	8	-3	146.09	22.40
-3	8	-3	103.69	6.10
3	8	3	112.29	12.50

-3	8	-3	116.29	14.80
-4	-8	-3	118.59	16.00
-4	-8	-3	73.69	16.10
4	-8	3	91.89	11.00
4	-8	3	73.19	7.30
-4	-8	-3	85.49	16.40
4	8	3	90.99	12.20
-4	8	-3	75.69	7.40
-4	8	-3	99.59	20.50
4	8	3	95.49	17.20
-4	8	-3	98.99	14.40
-4	8	-3	77.99	11.50
-5	-8	-3	1.10	6.80
-5	-8	-3	-3.60	9.60
5	-8	3	6.40	5.20
-5	-8	-3	11.10	11.90
-5	8	-3	3.60	10.70
-5	8	-3	10.10	7.90
5	8	3	5.70	8.80
5	8	3	-6.50	5.00
-5	8	-3	-7.30	7.10
-6	-8	-3	30.10	15.70
-6	-8	-3	58.79	12.80
-6	-8	-3	48.50	17.30
6	-8	3	63.69	9.20
-6	8	-3	67.49	14.90
-6	8	-3	79.09	22.00
-6	8	-3	64.69	9.20
-6	8	-3	100.19	12.70
6	8	3	51.19	16.60
-7	-8	-3	0.90	8.00

7	−8	3	−5.10	5.60
−7	−8	−3	11.10	14.40
−7	8	−3	5.70	14.60
−7	8	−3	16.00	11.90
−7	8	−3	−5.00	8.80
7	8	3	−0.90	8.10
−8	−8	−3	10.30	9.10
−8	−8	−3	9.60	16.90
−8	−8	−3	29.20	17.70
−8	8	−3	−0.70	11.30
8	8	3	−5.70	7.50
−8	8	−3	−1.40	13.10
−8	8	−3	−9.70	14.90
−9	−8	−3	53.89	23.10
−9	−8	−3	18.80	20.40
−9	8	−3	12.20	13.60
−9	8	−3	26.30	16.80
9	8	3	4.20	10.00
−9	8	−3	23.20	20.90
−10	−8	−3	−5.30	22.70
−10	8	−3	12.70	17.70
−10	8	−3	−1.30	25.30
9	−9	−3	−13.60	13.50
−9	−9	3	2.50	21.50
−9	−9	3	−5.10	22.70
−9	9	3	−31.20	17.30
−9	9	3	−35.50	15.90
−9	9	3	−28.60	21.20
−8	−9	3	37.20	21.40
8	−9	−3	11.20	12.20
−8	−9	3	−2.20	19.20

8	9	-3	19.10	11.00
-8	9	3	-7.10	19.10
-8	9	3	26.30	18.40
-8	9	3	-4.30	15.30
-7	-9	3	1.80	19.70
-7	-9	3	10.40	15.50
7	-9	-3	3.90	10.10
-7	9	3	-11.70	12.00
7	9	-3	2.00	9.30
-7	9	3	17.90	16.90
-7	9	3	-3.30	14.50
6	-9	-3	2.30	3.40
-6	-9	3	6.80	14.30
6	-9	-3	-8.30	9.00
-6	-9	3	-22.30	12.30
6	-9	-3	1.60	17.60
-6	9	3	1.20	12.90
-6	9	3	0.90	14.50
6	9	-3	-1.90	8.20
-6	9	3	-13.10	9.20
-5	-9	3	28.20	13.90
-5	-9	3	35.30	14.90
5	-9	-3	22.30	10.70
5	-9	-3	14.90	4.20
5	-9	-3	42.60	13.00
-5	-9	3	-5.70	12.50
-5	-9	3	19.70	8.90
-5	9	3	21.40	16.90
5	9	-3	31.10	7.90
-5	9	3	34.80	14.60
4	-9	-3	170.48	20.50

-4	-9	3	175.18	11.80
-4	-9	3	202.58	18.80
-4	-9	3	244.68	25.70
-4	-9	3	191.18	23.10
4	-9	-3	193.28	9.40
4	-9	-3	261.47	16.40
4	9	-3	178.48	16.00
-4	9	3	182.18	30.40
-4	9	3	186.98	14.00
4	9	-3	191.08	23.80
-4	9	3	182.78	24.10
4	9	-3	189.88	14.60
-3	-9	3	8.30	10.10
3	-9	-3	8.70	7.00
-3	-9	3	-3.80	7.80
3	-9	-3	7.50	3.50
-3	-9	3	1.70	3.40
-3	-9	3	2.90	7.30
3	9	-3	-7.10	7.90
-3	9	3	5.00	9.80
-3	9	3	-2.90	12.00
3	9	-3	10.60	4.50
-2	-9	3	343.17	24.70
2	-9	-3	346.57	24.20
-2	-9	3	340.67	23.00
-2	-9	3	354.96	23.10
2	9	-3	360.56	22.90
2	9	-3	328.67	16.10
-2	9	3	442.06	29.90
2	9	-3	354.66	24.80
-2	9	3	337.77	32.20

-2	9	3	318.67	21.50
1	-9	-3	107.19	15.40
1	-9	-3	108.99	16.70
-1	-9	3	104.99	13.10
-1	-9	3	105.49	19.20
-1	-9	3	100.49	13.30
-1	9	3	85.19	19.90
1	9	-3	96.89	14.30
1	9	-3	95.09	14.30
1	9	-3	94.49	8.10
0	-9	-3	2.40	5.80
0	-9	3	3.20	5.90
0	-9	3	-0.60	9.40
0	9	-3	-7.20	8.10
0	9	3	10.30	6.00
0	9	3	3.20	11.00
0	9	-3	0.90	7.70
0	9	-3	10.00	3.30
1	-9	3	12.10	6.60
1	-9	3	5.70	5.50
-1	-9	-3	7.90	9.80
-1	-9	-3	-2.20	5.90
1	-9	3	9.00	4.50
-1	9	-3	12.10	3.10
1	9	3	5.70	4.80
-1	9	-3	12.00	8.10
-1	9	-3	3.20	8.10
1	9	3	2.80	9.20
-1	9	-3	-0.50	5.40
-2	-9	-3	93.49	14.10
-2	-9	-3	119.79	12.60

-2	-9	-3	97.29	17.30
2	-9	3	89.19	12.50
-2	9	-3	130.29	22.00
-2	9	-3	128.39	6.70
2	9	3	147.49	15.50
2	9	3	101.69	19.00
2	9	3	119.49	11.60
-3	-9	-3	8.10	6.40
3	-9	3	-2.40	4.40
-3	-9	-3	2.10	11.70
3	9	3	-19.90	7.20
3	9	3	5.90	5.70
-3	9	-3	-2.20	3.10
3	9	3	0.10	10.00
-3	9	-3	-0.60	6.00
-3	9	-3	-6.60	8.90
-4	-9	-3	142.69	18.60
-4	-9	-3	162.98	24.50
-4	-9	-3	206.18	21.80
4	-9	3	139.39	17.10
-4	9	-3	116.29	22.10
4	9	3	114.89	22.10
4	9	3	171.78	14.90
-4	9	-3	138.99	17.60
-4	9	-3	142.29	9.70
-5	-9	-3	6.90	13.00
-5	-9	-3	-1.80	7.10
-5	-9	-3	23.60	11.20
5	-9	3	-6.30	5.40
5	9	3	5.80	9.90
5	9	3	7.70	6.10

-5	9	-3	0.50	5.40
-5	9	-3	3.50	12.20
-5	9	-3	18.90	9.00
-6	-9	-3	38.00	11.00
6	-9	3	23.70	8.60
-6	-9	-3	72.69	19.70
-6	-9	-3	43.10	19.50
6	9	3	31.60	15.60
6	9	3	37.80	9.90
-6	9	-3	39.50	12.50
-7	-9	-3	43.50	18.10
7	-9	3	15.00	7.40
-7	-9	-3	24.20	16.90
-7	9	-3	20.90	19.90
7	9	3	23.60	11.10
-7	9	-3	14.80	12.90
-8	-9	-3	-12.40	17.00
-8	-9	-3	12.90	10.30
-8	-9	-3	4.00	17.70
-8	9	-3	14.00	10.20
8	9	3	-2.30	9.80
-8	9	-3	11.40	18.50
-8	9	-3	3.10	13.30
-9	-9	-3	-3.70	21.30
-9	-9	-3	-35.30	19.90
9	9	3	-2.30	10.80
-9	9	-3	11.90	13.80
-9	9	-3	27.40	19.00
9	-10	-3	18.40	17.00
-9	-10	3	-21.60	25.80
-9	-10	3	26.00	28.60

-9	10	3	-0.70	24.30
-9	10	3	0.80	18.80
9	10	-3	-2.60	12.30
-9	10	3	14.60	20.80
-8	-10	3	30.10	25.50
-8	-10	3	-10.20	19.60
8	-10	-3	19.30	16.50
-8	10	3	31.90	18.70
8	10	-3	-6.00	10.10
-8	10	3	-11.50	19.50
-8	10	3	9.90	15.90
7	-10	-3	2.10	10.60
-7	-10	3	9.50	18.30
-7	-10	3	-4.10	17.70
-7	10	3	-9.30	14.80
-7	10	3	3.70	12.10
-7	10	3	11.50	21.50
7	10	-3	9.80	8.00
6	-10	-3	4.10	10.20
-6	-10	3	30.30	16.80
-6	-10	3	15.90	18.50
-6	10	3	18.70	12.00
-6	10	3	3.60	17.80
6	10	-3	4.30	7.00
-5	-10	3	32.10	13.90
-5	-10	3	24.10	15.10
5	-10	-3	30.60	11.20
-5	-10	3	17.70	9.00
-5	10	3	16.00	17.30
5	10	-3	26.20	9.00
-5	10	3	29.90	9.80

-5	10	3	11.10	13.10
-4	-10	3	32.60	14.30
-4	-10	3	25.20	14.10
4	-10	-3	19.70	9.80
-4	-10	3	20.20	9.70
-4	-10	3	23.50	6.20
-4	10	3	20.40	15.70
-4	10	3	22.20	12.50
4	10	-3	6.40	13.30
4	10	-3	27.30	7.90
4	10	-3	31.90	9.30
-3	-10	3	54.99	14.20
-3	-10	3	40.40	12.80
-3	-10	3	48.40	5.90
-3	-10	3	63.39	13.40
3	-10	-3	42.60	12.50
-3	10	3	32.40	13.60
-3	10	3	56.99	20.80
3	10	-3	91.29	16.20
3	10	-3	45.10	8.90
3	10	-3	37.60	10.30
-2	-10	3	1.50	7.90
-2	-10	3	-0.60	6.00
-2	-10	3	-3.20	11.80
2	-10	-3	10.70	7.10
2	10	-3	0.30	3.60
-2	10	3	-9.70	5.00
2	10	-3	8.60	6.90
2	10	-3	-13.10	8.50
-2	10	3	-5.10	13.00
2	10	-3	-3.00	9.60

1	-10	-3	42.60	11.90
1	-10	-3	37.20	12.50
-1	-10	3	43.90	13.10
-1	-10	3	44.80	9.70
-1	-10	3	42.30	10.10
1	10	-3	43.70	6.80
-1	10	3	49.10	17.10
-1	10	3	41.80	9.30
1	10	-3	47.90	11.90
0	-10	-3	99.09	18.30
0	-10	3	91.59	14.10
0	-10	-3	101.99	16.30
0	10	-3	89.49	16.80
0	10	-3	116.19	8.40
0	10	-3	102.59	13.90
0	10	3	88.69	11.60
0	10	3	95.19	20.70
-1	-10	-3	70.69	16.10
-1	-10	-3	88.49	14.90
1	-10	3	69.09	11.30
1	-10	3	92.89	13.30
1	10	3	85.99	12.00
-1	10	-3	93.79	21.50
-1	10	-3	70.19	13.40
-1	10	-3	96.79	12.80
-1	10	-3	78.09	7.00
1	10	3	65.39	17.10
-1	10	-3	69.09	6.90
2	-10	3	108.19	15.10
-2	-10	-3	115.89	16.80
-2	-10	-3	141.79	22.40

2	10	3	100.09	13.70
−2	10	−3	107.29	21.20
2	10	3	78.49	19.20
−2	10	−3	86.19	20.40
−2	10	−3	97.49	7.90
−2	10	−3	112.79	7.40
2	10	3	103.89	11.10
−3	−10	−3	1.30	11.10
3	−10	3	0.70	6.00
−3	−10	−3	7.40	7.40
−3	10	−3	−5.20	6.60
−3	10	−3	−3.10	12.10
−3	10	−3	−0.80	4.10
3	10	3	14.00	8.80
3	10	3	7.10	5.70
3	10	3	−2.30	10.40
4	−10	3	−3.40	6.40
−4	−10	−3	12.50	10.00
−4	−10	−3	10.00	9.00
−4	−10	−3	9.10	12.30
−4	10	−3	−8.30	10.60
4	10	3	−5.20	11.10
−4	10	−3	−0.90	7.40
−4	10	−3	6.00	5.50
4	10	3	2.80	6.20
5	−10	3	−13.30	7.10
−5	−10	−3	5.40	14.90
−5	−10	−3	−6.60	11.10
−5	10	−3	2.20	8.50
−5	10	−3	−1.10	13.40
5	10	3	14.90	7.80

5	10	3	4.10	12.10
-5	10	-3	8.40	6.80
6	-10	3	4.20	7.60
-6	-10	-3	4.30	8.00
-6	-10	-3	18.10	18.40
-6	-10	-3	-1.40	14.50
-6	10	-3	19.60	11.40
6	10	3	13.20	7.80
-6	10	-3	18.20	17.80
-7	-10	-3	9.30	19.40
-7	-10	-3	4.20	9.50
7	-10	3	2.60	8.70
-7	-10	-3	16.40	19.60
7	10	3	16.40	10.20
7	10	3	29.70	16.50
-7	10	-3	-14.00	12.10
-7	10	-3	-1.80	16.20
-8	-10	-3	4.00	11.70
-8	-10	-3	-28.30	20.30
-8	-10	-3	53.99	24.40
-8	10	-3	-14.90	18.40
-8	10	-3	10.40	14.90
8	10	3	-4.40	9.10
-8	-11	3	0.40	23.90
8	-11	-3	-2.90	15.20
-8	-11	3	-5.70	23.90
-8	11	3	-5.30	18.50
-8	11	3	3.20	21.60
-7	-11	3	-15.30	20.20
7	-11	-3	20.30	13.80
-7	-11	3	-11.80	19.30

-7	11	3	4.40	12.90
-7	11	3	11.30	16.00
7	11	-3	31.40	11.30
-7	11	3	-13.20	20.30
6	-11	-3	-1.90	12.20
-6	-11	3	13.60	17.90
-6	-11	3	33.30	18.80
-6	11	3	19.80	14.60
6	11	-3	5.30	9.30
-6	11	3	13.70	21.30
-5	-11	3	23.90	17.40
5	-11	-3	19.70	12.30
-5	-11	3	4.60	9.60
-5	11	3	-2.30	12.20
-5	11	3	48.00	21.40
5	11	-3	-1.20	7.90
-4	-11	3	-6.70	12.80
4	-11	-3	-0.90	9.30
-4	-11	3	-2.10	7.90
-4	-11	3	3.40	6.50
4	11	-3	8.40	7.30
-4	11	3	-8.60	10.00
4	11	-3	17.90	15.40
4	11	-3	4.20	8.80
-4	11	3	-21.70	14.40
-3	-11	3	21.50	9.60
3	-11	-3	1.60	7.60
-3	-11	3	3.90	16.90
3	11	-3	17.20	7.80
3	11	-3	35.10	14.20
-3	11	3	8.30	7.20

3	11	-3	3.80	7.60
-3	11	3	8.30	18.50
-2	-11	3	27.30	10.20
2	-11	-3	30.80	14.20
-2	-11	3	25.00	9.70
2	-11	-3	22.80	11.40
-2	11	3	33.40	18.40
2	11	-3	-3.10	10.40
2	11	-3	28.60	10.70
2	11	-3	18.20	6.70
-2	11	3	20.90	8.10
-1	-11	3	10.40	8.00
1	-11	-3	7.60	7.90
-1	-11	3	6.70	8.50
-1	11	3	13.10	13.90
1	11	-3	-1.50	8.80
-1	11	3	0.10	7.00
1	11	-3	-0.70	4.00
0	-11	-3	29.80	10.60
0	-11	-3	20.70	12.50
0	-11	3	28.30	10.10
0	11	3	19.30	8.20
0	11	-3	39.80	9.40
0	11	-3	36.60	6.20
0	11	-3	33.40	9.70
0	11	-3	20.70	13.40
0	11	3	32.80	17.70
1	-11	3	15.40	8.20
-1	-11	-3	9.30	8.80
-1	-11	-3	7.90	11.10
-1	11	-3	11.00	4.60

-1	11	-3	6.90	6.90
-1	11	-3	1.10	9.40
-1	11	-3	22.80	4.90
-1	11	-3	3.10	11.00
1	11	3	10.20	11.60
1	11	3	24.20	8.90
2	-11	3	4.20	7.70
-2	-11	-3	5.60	12.60
-2	-11	-3	8.60	8.70
-2	11	-3	10.50	7.00
-2	11	-3	21.80	10.10
-2	11	-3	10.90	4.50
2	11	3	10.30	6.80
2	11	3	3.30	11.90
-2	11	-3	-1.10	13.50
-2	11	-3	15.30	5.50
3	-11	3	2.20	7.30
-3	-11	-3	3.70	12.60
-3	-11	-3	-0.80	8.90
-3	-11	-3	3.80	7.60
-3	11	-3	-2.70	7.10
-3	11	-3	-0.40	5.00
3	11	3	7.50	8.80
3	11	3	17.20	8.50
3	11	3	19.80	15.00
-3	11	-3	-7.60	13.40
-4	-11	-3	7.60	12.20
-4	-11	-3	8.30	9.50
4	-11	3	4.00	7.60
-4	-11	-3	10.00	15.30
4	11	3	14.40	13.50

-4	11	-3	17.40	7.10
-4	11	-3	27.80	9.90
-4	11	-3	13.90	17.30
4	11	3	19.50	8.40
-5	-11	-3	-1.60	8.70
-5	-11	-3	15.50	19.40
-5	-11	-3	-12.30	13.40
5	-11	3	-2.30	7.80
-5	11	-3	43.30	19.00
5	11	3	8.30	8.70
5	11	3	19.60	19.30
-6	-11	-3	-3.80	8.60
6	-11	3	-9.40	9.30
-6	-11	-3	18.80	17.10
6	11	3	22.10	15.50
-6	11	-3	-8.50	11.10
-6	11	-3	-20.80	14.80
6	11	3	-4.10	8.80
-7	-11	-3	13.30	20.10
-7	-11	-3	22.60	24.50
7	-11	3	19.40	11.60
-7	-11	-3	-2.60	11.60
-7	11	-3	15.00	14.50
7	11	3	3.90	14.90
7	11	3	-7.60	8.60
-7	11	-3	-7.50	22.00
-8	-11	-3	20.60	13.30
-8	-11	-3	8.00	24.10
-8	-11	-3	53.59	26.10
8	11	3	-7.60	11.30
-8	11	-3	-3.90	17.10

-7	-12	3	10.80	22.80
-7	-12	3	33.50	26.20
7	-12	-3	-2.60	14.70
7	12	-3	9.00	9.40
-7	12	3	-33.70	24.50
-7	12	3	4.30	15.70
6	-12	-3	0.80	13.30
-6	-12	3	-12.70	11.00
-6	-12	3	42.10	23.00
-6	12	3	22.30	20.30
-6	12	3	27.80	16.90
6	12	-3	10.70	10.00
-5	-12	3	11.60	11.70
-5	-12	3	1.00	16.70
5	-12	-3	1.00	12.40
-5	12	3	-7.50	12.70
5	12	-3	9.70	9.60
-5	12	3	-5.80	22.20
4	-12	-3	16.50	10.60
-4	-12	3	22.60	16.70
-4	-12	3	5.80	9.80
4	12	-3	21.40	15.10
4	12	-3	16.50	8.20
-4	12	3	-7.20	7.10
-4	12	3	8.10	17.30
3	-12	-3	6.90	15.40
3	-12	-3	4.30	9.80
-3	-12	3	-4.80	8.30
-3	12	3	12.40	17.40
-3	12	3	0.70	8.00
3	12	-3	2.20	6.70

2	-12	-3	-10.00	13.70
2	-12	-3	8.50	9.40
-2	-12	3	14.90	11.20
-2	-12	3	14.90	8.50
-2	12	3	7.00	7.20
2	12	-3	16.30	11.30
-2	12	3	7.10	17.00
2	12	-3	-3.30	5.80
2	12	-3	14.90	10.50
1	-12	-3	-1.70	12.10
-1	-12	3	10.20	10.10
-1	-12	3	-5.80	7.70
1	-12	-3	-13.70	7.70
-1	12	3	14.20	17.30
-1	12	3	8.00	7.10
1	12	-3	0.30	5.30
1	12	-3	14.00	11.60
0	-12	-3	7.00	11.10
0	-12	-3	-2.20	13.10
0	12	-3	-0.50	12.50
0	12	-3	0.40	7.60
0	12	-3	15.00	5.20
0	12	3	20.50	18.40
0	12	3	6.20	7.20
0	12	-3	-11.60	8.30
1	-12	3	24.10	10.40
-1	-12	-3	24.20	16.40
1	-12	3	19.40	8.60
-1	-12	-3	4.30	8.00
1	12	3	15.00	9.10
-1	12	-3	26.00	6.10

1	12	3	33.50	16.70
−1	12	−3	4.50	8.20
−1	12	−3	25.80	14.10
−1	12	−3	12.60	8.10
−2	−12	−3	−6.50	13.90
2	−12	3	21.20	10.40
−2	−12	−3	7.90	8.80
2	12	3	9.90	8.10
−2	12	−3	25.70	9.80
−2	12	−3	27.70	16.80
2	12	3	10.50	14.40
−2	12	−3	10.60	6.10
−2	12	−3	13.10	7.20
−2	12	−3	9.10	5.70
−3	−12	−3	32.90	12.60
−3	−12	−3	20.60	16.40
3	−12	3	36.60	11.50
3	12	3	28.70	9.30
−3	12	−3	10.40	12.30
−3	12	−3	10.00	14.40
−3	12	−3	34.30	7.10
−3	12	−3	27.00	8.90
3	12	3	37.10	9.80
3	12	3	56.89	18.50
−4	−12	−3	−8.60	16.00
−4	−12	−3	−4.00	10.30
4	−12	3	8.00	10.60
−4	−12	−3	5.30	12.40
4	12	3	6.70	15.30
−4	12	−3	12.80	8.80
4	12	3	−1.30	8.60

-5	-12	-3	3.30	12.50
-5	-12	-3	27.00	15.60
-5	-12	-3	-2.30	17.40
5	-12	3	-14.50	9.40
5	12	3	4.30	8.40
-5	12	-3	-6.30	17.50
-5	12	-3	-19.20	9.80
5	12	3	-5.10	14.60
-6	-12	-3	14.80	12.70
-6	-12	-3	3.00	20.90
6	-12	3	4.90	11.30
-6	-12	-3	-18.40	18.60
6	12	3	-14.20	9.50
-6	12	-3	21.30	23.10
6	12	3	-4.90	15.40
-6	12	-3	7.90	12.10
-7	-12	-3	-4.20	24.80
-7	-12	-3	11.50	13.00
7	-12	3	36.10	13.10
-7	12	-3	-21.20	13.90
7	12	3	10.00	11.60
-7	12	-3	-4.10	22.90
7	12	3	14.40	18.50
6	-13	-3	2.70	15.10
-6	-13	3	5.80	23.30
-6	-13	3	-8.30	12.80
6	13	-3	-2.60	10.20
-6	13	3	-16.90	23.00
5	-13	-3	9.40	14.10
-5	-13	3	-7.30	21.00
-5	-13	3	-8.20	12.00

-5	13	3	13.40	25.00
5	13	-3	9.80	12.50
5	13	-3	-5.60	8.90
-4	-13	3	29.20	19.10
4	-13	-3	4.20	11.60
-4	-13	3	5.60	10.30
-4	13	3	-2.40	17.60
4	13	-3	12.50	11.20
4	13	-3	13.30	9.70
-4	13	3	-9.30	9.20
-3	-13	3	13.70	25.80
3	-13	-3	12.50	17.10
-3	-13	3	19.00	12.70
3	-13	-3	12.40	12.50
-3	13	3	20.80	10.40
3	13	-3	26.40	9.60
-3	13	3	30.00	22.00
3	13	-3	14.30	11.70
2	-13	-3	-2.90	9.90
-2	-13	3	10.60	13.40
-2	-13	3	5.60	9.00
2	13	-3	-25.30	10.10
2	13	-3	1.20	6.90
-2	13	3	16.20	18.50
-2	13	3	-4.40	8.30
-1	-13	3	30.80	14.20
1	-13	-3	21.80	17.30
-1	-13	3	7.70	10.10
1	-13	-3	34.70	14.60
-1	13	3	41.80	10.20
1	13	-3	21.10	14.10

1	13	-3	23.20	8.30
-1	13	3	-1.00	17.50
0	-13	-3	-0.60	14.30
0	-13	-3	12.70	11.20
0	-13	3	10.40	9.10
0	13	-3	14.00	14.00
0	13	3	-5.60	7.90
0	13	-3	-5.10	7.10
0	13	3	-33.90	14.30
-1	-13	-3	-0.30	14.30
1	-13	3	14.20	10.80
-1	-13	-3	-4.60	9.20
-1	13	-3	-6.30	7.70
1	13	3	21.40	9.20
-1	13	-3	-7.20	5.50
1	13	3	-4.10	14.10
-1	13	-3	-2.10	16.00
-2	-13	-3	7.60	12.70
-2	-13	-3	-21.00	14.40
2	-13	3	36.40	10.80
2	13	3	13.00	15.70
-2	13	-3	16.00	6.30
-2	13	-3	-2.00	17.90
2	13	3	-5.90	7.00
3	-13	3	8.30	10.20
-3	-13	-3	9.40	17.50
-3	-13	-3	13.30	11.50
-3	13	-3	9.20	6.40
-3	13	-3	-13.30	19.30
-3	13	-3	5.90	6.80
3	13	3	-13.00	15.30

3	13	3	-8.60	8.00
-4	-13	-3	-8.50	12.70
-4	-13	-3	0.20	20.30
-4	-13	-3	-17.50	13.90
-4	13	-3	2.30	7.20
4	13	3	-5.30	8.30
-4	13	-3	-0.10	19.20
4	13	3	-34.10	14.30
5	-13	3	10.60	12.80
-5	-13	-3	0.60	10.90
-5	-13	-3	-6.10	23.50
-5	-13	-3	32.90	18.50
5	13	3	3.80	8.70
-5	13	-3	-3.80	22.40
5	13	3	30.40	19.20
-6	-13	-3	-17.00	20.90
-6	-13	-3	2.20	13.10
-6	-13	-3	11.90	25.90
6	-13	3	15.60	14.00
-6	13	-3	6.40	24.80
6	13	3	25.40	17.60
-5	-14	3	11.90	12.90
5	-14	-3	-10.30	16.00
-5	14	3	-28.50	10.60
5	14	-3	11.50	10.80
-5	14	3	6.70	24.40
4	-14	-3	-16.70	12.90
4	14	-3	18.70	12.80
4	14	-3	2.70	10.10
-4	14	3	-8.40	24.10
3	-14	-3	24.70	15.00

-3	-14	3	-4.50	12.80
-3	14	3	13.80	19.30
3	14	-3	27.00	14.30
-3	14	3	11.70	10.60
-2	-14	3	18.20	13.10
2	-14	-3	13.40	15.10
2	14	-3	-20.20	13.90
2	14	-3	-14.20	7.90
-2	14	3	38.90	12.00
-2	14	3	16.30	21.60
1	-14	-3	3.90	17.30
1	-14	-3	11.00	12.40
-1	-14	3	-26.60	9.60
-1	14	3	18.50	10.30
1	14	-3	-16.90	14.10
1	14	-3	6.60	7.50
-1	14	3	-2.50	19.10
0	-14	3	0.80	11.00
0	-14	-3	-2.50	18.50
0	-14	-3	-1.80	10.60
0	14	-3	-5.70	15.00
0	14	-3	6.70	6.70
0	14	3	-30.30	18.60
-1	-14	-3	11.70	20.10
1	-14	3	-8.60	11.20
-1	-14	-3	-7.10	11.10
-1	14	-3	-22.10	17.70
1	14	3	5.20	9.90
1	14	3	-1.30	18.10
-2	-14	-3	10.30	14.10
-2	-14	-3	-0.60	20.60

2	-14	3	0.30	10.40
2	14	3	17.70	9.90
-2	14	-3	8.50	7.10
-2	14	-3	18.00	24.20
2	14	3	10.40	17.10
3	-14	3	7.00	12.50
-3	-14	-3	-7.60	11.00
-3	-14	-3	13.50	20.90
-3	14	-3	40.50	22.30
3	14	3	29.60	19.80
3	14	3	-8.80	9.50
-4	-14	-3	-4.30	26.10
4	-14	3	4.60	12.70
-4	14	-3	0.50	23.90
4	14	3	-15.30	17.90
4	14	3	-6.50	9.90
-5	-14	-3	-11.50	24.40
-5	-14	-3	-3.10	13.90
5	-14	3	17.10	14.50
5	14	3	5.30	20.40
-5	14	-3	-29.60	21.60
-3	-15	3	-19.30	11.60
-3	15	3	0.50	12.60
3	15	-3	-15.90	9.50
-3	15	3	-14.80	29.10
3	15	-3	38.50	16.60
-2	-15	3	35.90	16.80
2	-15	-3	23.40	17.00
2	15	-3	-0.20	9.90
-2	15	3	-4.40	23.50
2	15	-3	40.20	18.00

-2	15	3	15.10	11.70
-1	-15	3	-12.90	12.60
1	-15	-3	12.10	16.40
-1	15	3	-21.00	20.60
1	15	-3	17.50	18.70
-1	15	3	3.20	11.60
1	15	-3	7.80	9.90
0	-15	3	3.00	12.00
0	15	-3	-6.80	8.20
0	15	3	-0.40	12.00
0	15	3	22.90	24.30
0	15	-3	-2.90	19.40
1	-15	3	4.90	11.80
-1	-15	-3	-4.60	13.80
-1	15	-3	32.80	23.30
1	15	3	22.80	23.60
1	15	3	16.30	11.00
-1	15	-3	-7.90	7.50
2	-15	3	4.40	13.60
-2	-15	-3	23.80	27.50
-2	-15	-3	27.30	17.00
2	15	3	7.40	23.50
2	15	3	8.40	12.40
-2	15	-3	28.70	23.10
-3	-15	-3	14.50	15.10
-3	-15	-3	-37.60	25.70
3	-15	3	-0.20	14.40
3	15	3	23.30	23.00
3	15	3	8.30	11.10
-3	15	-3	-41.70	23.90
11	0	-4	-16.00	14.10

-11	0	4	10.50	21.50
11	0	-4	-7.50	10.60
-11	0	4	6.90	21.80
10	0	-4	15.70	10.00
10	0	-4	31.40	11.20
-10	0	4	20.50	19.30
-10	0	4	21.40	18.70
-10	0	4	45.20	17.00
-9	0	4	4.30	11.50
-9	0	4	9.50	13.70
9	0	-4	9.00	7.80
-9	0	4	-3.60	11.50
8	0	-4	85.89	12.10
-8	0	4	74.69	17.80
-8	0	4	54.39	16.70
-8	0	4	85.39	19.70
-7	0	4	-4.00	9.50
7	0	-4	-1.20	5.40
-7	0	4	3.70	9.80
-7	0	4	4.00	9.10
6	0	-4	17.30	5.40
-6	0	4	25.90	10.50
-6	0	4	31.70	12.20
-6	0	4	25.80	10.50
-5	0	4	5.60	6.60
5	0	-4	-3.80	2.80
-5	0	4	8.80	8.70
4	0	-4	1062.99	44.90
-4	0	4	1124.19	43.50
-4	0	4	1045.00	44.50
-4	0	4	1026.80	39.70

4	0	-4	1018.30	32.10
4	0	-4	1007.60	42.30
-4	0	4	958.00	31.10
-3	0	4	-1.80	3.50
-3	0	4	3.50	5.10
-3	0	4	1.60	2.80
3	0	-4	0.90	2.30
3	0	-4	3.50	5.40
3	0	-4	2.60	5.30
-2	0	4	675.63	21.00
2	0	-4	655.53	25.50
2	0	-4	641.34	21.00
-2	0	4	690.03	26.60
2	0	-4	656.93	30.10
-2	0	4	651.33	28.20
-2	0	4	615.04	29.50
2	0	-4	677.23	29.00
1	0	-4	2.80	1.90
1	0	-4	1.20	3.30
-1	0	4	4.10	3.00
1	0	-4	1.20	3.00
-1	0	4	-1.30	1.40
-1	0	4	0.80	2.40
0	0	4	9143.88	204.48
0	0	4	9213.18	192.18
0	0	-4	9351.46	197.08
0	0	4	8990.90	192.68
0	0	-4	9178.18	203.68
0	0	-4	9112.79	193.78
0	0	4	8702.93	199.18
1	0	4	0.20	1.40

−1	0	−4	−2.30	2.90
−1	0	−4	−1.40	1.70
1	0	4	3.70	2.40
1	0	4	−0.60	1.60
1	0	4	−0.90	2.40
−1	0	−4	−0.20	1.40
−1	0	−4	−0.70	2.70
−1	0	−4	0.20	2.10
−1	0	−4	0.00	2.40
−2	0	−4	2.70	1.70
−2	0	−4	7.30	4.20
2	0	4	0.60	1.30
−2	0	−4	−0.20	2.60
−2	0	−4	−2.60	1.60
−2	0	−4	−2.40	1.40
−2	0	−4	0.60	2.70
−2	0	−4	3.50	3.50
2	0	4	−0.60	2.40
2	0	4	−4.10	3.40
2	0	4	6.40	2.40
−3	0	−4	2.60	3.50
−3	0	−4	0.80	1.50
−3	0	−4	3.90	4.60
3	0	4	1.40	2.00
3	0	4	1.80	2.10
−3	0	−4	−0.50	1.90
−3	0	−4	−0.20	1.80
3	0	4	−1.30	4.90
4	0	4	752.22	31.80
−4	0	−4	844.02	33.10
−4	0	−4	750.22	23.90

−4	0	−4	832.92	37.30
−4	0	−4	810.82	35.30
4	0	4	777.92	19.60
4	0	4	821.62	25.80
4	0	4	648.24	38.60
−4	0	−4	799.42	25.10
4	0	4	746.93	23.70
−4	0	−4	790.92	18.90
5	0	4	−0.80	3.20
−5	0	−4	−6.30	6.40
−5	0	−4	−4.10	5.30
5	0	4	−0.20	3.20
−6	0	−4	27.20	10.30
6	0	4	27.40	6.10
−6	0	−4	21.80	10.40
6	0	4	43.50	7.20
−7	0	−4	−4.20	7.70
−7	0	−4	−9.90	8.10
7	0	4	−3.00	4.30
−8	0	−4	67.69	18.80
8	0	4	54.59	9.20
−8	0	−4	48.00	15.80
9	0	4	0.60	6.50
−9	0	−4	−1.90	11.20
−9	0	−4	−22.20	11.80
−10	0	−4	9.30	16.00
−10	0	−4	−11.10	15.20
10	0	4	15.20	9.00
−11	0	−4	15.30	20.30
−11	0	−4	8.80	29.90
11	0	4	−0.90	10.90

11	−1	−4	−13.80	10.00
11	−1	−4	9.50	15.50
−11	−1	4	5.70	19.80
−11	−1	4	12.10	35.20
−11	1	4	−19.60	21.80
−11	1	4	1.70	19.30
11	1	−4	2.40	10.50
11	1	−4	44.90	17.10
−11	1	4	12.70	21.90
10	−1	−4	−7.50	8.50
−10	−1	4	1.20	15.50
−10	−1	4	−10.70	15.60
−10	−1	4	−7.30	15.30
10	−1	−4	14.60	13.00
−10	1	4	0.10	14.10
−10	1	4	6.10	17.30
10	1	−4	6.80	8.80
−10	1	4	−32.80	16.10
−9	−1	4	16.50	15.00
−9	−1	4	20.20	16.00
9	−1	−4	7.00	7.60
9	−1	−4	−4.10	10.00
−9	−1	4	17.60	13.90
−9	1	4	3.20	12.60
9	1	−4	1.60	6.50
−9	1	4	−18.10	12.00
−9	1	4	−5.00	11.80
−8	−1	4	148.09	27.10
−8	−1	4	203.78	30.30
8	−1	−4	143.49	18.30
8	−1	−4	213.58	16.70

−8	−1	4	156.78	23.70
−8	1	4	133.89	24.40
8	1	−4	163.58	14.60
−8	1	4	145.79	23.40
−8	1	4	187.28	29.40
−7	−1	4	12.30	8.50
−7	−1	4	0.70	10.70
7	−1	−4	−0.80	5.50
−7	−1	4	−0.20	10.20
−7	1	4	11.60	9.70
−7	1	4	−4.70	7.00
7	1	−4	16.10	6.10
−7	1	4	0.00	9.90
−6	−1	4	106.19	16.80
6	−1	−4	102.79	11.80
−6	−1	4	98.39	18.90
−6	−1	4	124.89	18.30
−6	1	4	148.39	21.60
−6	1	4	130.29	20.30
−6	1	4	99.39	19.20
6	1	−4	115.69	12.80
5	−1	−4	152.98	13.00
−5	−1	4	178.98	16.10
−5	−1	4	126.19	17.30
−5	−1	4	152.48	21.80
−5	1	4	157.38	17.00
−5	1	4	116.19	17.60
5	1	−4	155.38	11.30
−5	1	4	167.68	22.00
−4	−1	4	952.00	34.90
4	−1	−4	887.21	40.20

4	−1	−4	907.21	29.90
−4	−1	4	942.71	39.90
−4	−1	4	921.21	29.10
−4	−1	4	943.71	42.80
4	−1	−4	917.71	40.40
4	1	−4	910.71	29.60
4	1	−4	914.91	39.60
−4	1	4	957.40	39.70
−4	1	4	929.71	30.20
−4	1	4	971.60	41.40
4	1	−4	881.91	42.40
−4	1	4	899.11	39.70
−3	−1	4	200.48	15.90
3	−1	−4	184.58	15.20
−3	−1	4	175.88	16.90
3	−1	−4	181.18	16.30
−3	−1	4	203.18	10.50
3	−1	−4	195.78	11.00
3	−1	−4	197.78	17.90
−3	−1	4	200.58	12.90
−3	1	4	170.68	15.30
3	1	−4	211.38	19.80
3	1	−4	171.28	10.10
−3	1	4	166.48	10.30
−3	1	4	149.19	14.60
3	1	−4	207.08	16.70
−2	−1	4	350.76	13.10
−2	−1	4	364.36	15.90
2	−1	−4	361.26	21.30
−2	−1	4	312.07	20.60
−2	−1	4	330.27	18.80

2	-1	-4	362.56	14.60
2	-1	-4	340.07	20.00
2	-1	-4	344.87	20.30
2	1	-4	320.87	19.00
2	1	-4	327.07	21.20
-2	1	4	339.47	20.20
2	1	-4	353.56	13.50
2	1	-4	348.07	14.60
-2	1	4	338.97	20.40
-2	1	4	324.07	13.80
-2	1	4	342.77	20.00
1	-1	-4	39.70	8.00
-1	-1	4	33.40	4.10
1	-1	-4	34.30	7.20
1	-1	-4	35.20	5.00
-1	-1	4	41.40	7.60
1	-1	-4	30.90	6.70
1	-1	-4	34.50	6.00
-1	-1	4	42.70	4.60
1	1	-4	29.30	6.30
1	1	-4	40.70	4.50
1	1	-4	56.49	9.50
-1	1	4	23.10	6.10
-1	1	4	29.70	4.30
1	1	-4	39.50	5.60
-1	1	4	32.90	6.70
0	-1	-4	354.86	14.10
0	-1	-4	340.77	16.70
0	-1	4	375.76	17.90
0	-1	4	325.87	10.80
0	-1	-4	336.47	15.80

0	−1	4	326.77	12.90
0	−1	4	335.07	11.70
0	−1	−4	342.97	19.00
0	1	4	349.67	16.80
0	1	−4	346.37	16.90
0	1	−4	355.66	13.30
0	1	4	359.36	19.50
0	1	4	349.77	13.40
0	1	4	333.47	12.80
1	−1	4	396.36	11.20
−1	−1	−4	394.26	13.50
1	−1	4	398.46	16.60
1	−1	4	406.96	17.30
−1	−1	−4	391.66	16.20
−1	−1	−4	414.46	18.50
−1	−1	−4	416.06	20.10
−1	−1	−4	385.16	14.60
−1	−1	−4	379.96	19.90
−1	1	−4	398.26	21.00
1	1	4	407.56	18.70
−1	1	−4	418.66	16.70
−1	1	−4	430.36	18.60
−1	1	−4	391.56	13.50
1	1	4	392.36	13.60
−1	1	−4	405.66	12.20
1	1	4	379.36	16.10
1	1	4	386.16	19.70
1	1	4	400.26	14.60
−2	−1	−4	482.75	23.80
2	−1	4	474.15	15.50
−2	−1	−4	516.55	22.40

−2	−1	−4	522.25	16.30
−2	−1	−4	490.95	22.50
2	−1	4	506.75	24.20
−2	−1	−4	490.65	16.80
−2	−1	−4	453.55	22.50
2	−1	4	492.65	14.90
−2	1	−4	467.65	14.30
2	1	4	464.75	15.70
−2	1	−4	481.35	19.20
−2	1	−4	484.55	15.20
2	1	4	462.25	16.40
2	1	4	498.55	18.00
2	1	4	467.85	22.00
−2	1	−4	482.25	24.30
2	1	4	494.75	23.30
−2	1	−4	475.45	20.60
−2	1	−4	510.35	20.50
−2	1	−4	471.45	15.30
3	−1	4	249.38	17.90
−3	−1	−4	249.18	10.20
3	−1	4	253.57	10.40
−3	−1	−4	242.88	18.30
3	−1	4	248.48	9.20
−3	−1	−4	247.48	16.40
−3	−1	−4	221.88	12.50
−3	−1	−4	246.38	16.50
3	−1	4	245.18	14.50
−3	1	−4	265.27	10.40
−3	1	−4	260.17	10.90
−3	1	−4	268.17	19.10
3	1	4	245.98	16.40

3	1	4	253.57	20.70
-3	1	-4	253.97	7.10
3	1	4	292.97	18.40
3	1	4	251.87	11.40
-3	1	-4	275.27	27.00
3	1	4	276.87	11.70
4	-1	4	1171.58	31.90
-4	-1	-4	1201.48	34.10
4	-1	4	1220.08	31.40
-4	-1	-4	1148.79	46.80
-4	-1	-4	1228.98	35.50
-4	-1	-4	1290.87	45.20
4	-1	4	1197.08	41.00
-4	-1	-4	1223.38	30.80
-4	1	-4	1223.48	40.90
4	1	4	1099.79	51.69
4	1	4	1220.18	33.80
-4	1	-4	1190.48	45.60
4	1	4	1250.27	36.80
4	1	4	1267.97	32.30
-4	1	-4	1210.78	47.30
5	-1	4	0.80	3.20
-5	-1	-4	3.00	2.20
-5	-1	-4	-6.40	5.80
5	-1	4	4.60	2.70
-5	-1	-4	16.50	7.40
-5	1	-4	-0.30	6.60
5	1	4	0.40	3.50
5	1	4	7.00	3.10
5	1	4	0.70	3.90
-6	-1	-4	0.70	6.80

-6	-1	-4	4.40	7.60
6	-1	4	8.70	4.20
6	-1	4	1.10	4.10
-6	1	-4	-2.80	7.50
-6	1	-4	-6.00	5.30
6	1	4	4.60	3.10
6	1	4	0.90	4.00
7	-1	4	28.60	7.40
-7	-1	-4	18.30	10.80
-7	-1	-4	6.50	8.90
-7	1	-4	29.00	10.70
7	1	4	32.60	6.80
7	1	4	19.00	4.50
-7	1	-4	12.20	10.20
-8	-1	-4	118.49	20.60
-8	-1	-4	195.68	28.80
8	-1	4	178.98	13.30
8	1	4	182.18	9.90
-8	1	-4	132.59	24.30
8	1	4	176.78	14.40
-8	1	-4	140.69	21.30
-9	-1	-4	14.30	13.80
9	-1	4	18.40	7.60
-9	-1	-4	24.60	15.00
-9	1	-4	4.70	12.60
-9	1	-4	3.40	15.00
9	1	4	0.70	6.90
10	-1	4	18.70	8.40
-10	-1	-4	9.50	16.60
-10	-1	-4	10.00	14.90
-10	1	-4	22.10	18.10

10	1	4	-4.00	8.50
-10	1	-4	13.20	16.60
-11	-1	-4	24.30	23.30
-11	-1	-4	-8.60	19.40
11	1	4	-7.40	9.70
-11	1	-4	4.80	20.30
11	-2	-4	27.50	16.30
-11	-2	4	24.70	23.10
11	-2	-4	-6.90	10.10
-11	-2	4	15.80	38.00
-11	-2	4	-6.70	17.80
-11	2	4	-12.30	19.80
11	2	-4	-3.50	8.90
-11	2	4	14.30	24.70
-11	2	4	3.70	21.00
-10	-2	4	10.30	16.20
10	-2	-4	14.50	9.70
10	-2	-4	0.80	12.80
-10	-2	4	26.30	18.10
-10	-2	4	8.00	20.20
-10	2	4	11.70	16.10
-10	2	4	21.10	16.40
-10	2	4	30.30	19.70
10	2	-4	6.10	10.40
-9	-2	4	10.00	14.80
9	-2	-4	18.30	11.70
-9	-2	4	3.10	12.60
-9	-2	4	-3.80	12.20
-9	2	4	9.50	14.70
9	2	-4	12.90	7.70
-9	2	4	3.60	12.10

−9	2	4	21.60	15.00
−8	−2	4	−8.60	8.90
−8	−2	4	−3.30	10.70
8	−2	−4	6.50	9.90
8	−2	−4	0.80	5.90
−8	−2	4	3.60	12.50
−8	2	4	−13.90	10.70
−8	2	4	−1.00	9.80
−8	2	4	17.80	12.60
8	2	−4	2.10	5.60
7	−2	−4	12.20	6.30
−7	−2	4	−3.40	11.10
−7	−2	4	18.90	9.60
7	−2	−4	9.90	7.70
−7	−2	4	11.60	11.00
−7	2	4	21.40	11.80
7	2	−4	9.10	6.00
−7	2	4	16.20	11.50
−7	2	4	13.80	11.60
−6	−2	4	134.39	23.30
−6	−2	4	110.09	19.00
6	−2	−4	116.99	17.30
6	−2	−4	150.98	12.40
6	2	−4	132.59	11.80
−6	2	4	125.79	21.20
−6	2	4	159.38	23.40
−6	2	4	108.99	19.00
5	−2	−4	45.10	9.60
−5	−2	4	44.50	11.50
−5	−2	4	43.50	12.70
−5	2	4	56.09	12.50

−5	2	4	49.00	12.00
5	2	−4	48.60	8.10
−5	2	4	41.20	11.10
4	−2	−4	721.23	34.70
−4	−2	4	771.12	38.70
4	−2	−4	746.33	26.10
−4	−2	4	756.02	35.10
−4	−2	4	695.03	24.10
−4	−2	4	705.13	27.80
4	−2	−4	752.22	30.90
−4	2	4	765.32	36.00
4	2	−4	747.93	25.40
4	2	−4	675.03	33.70
−4	2	4	716.63	36.40
−4	2	4	734.23	35.10
−3	−2	4	344.87	24.50
3	−2	−4	371.46	24.00
3	−2	−4	354.36	22.10
−3	−2	4	368.76	14.70
3	−2	−4	376.16	16.30
−3	−2	4	377.66	21.90
−3	−2	4	366.36	16.30
3	−2	−4	347.97	23.20
3	−2	−4	380.16	19.80
−3	2	4	326.37	22.30
−3	2	4	376.46	22.90
3	2	−4	373.36	24.60
−3	2	4	383.16	16.60
3	2	−4	369.06	15.20
−3	2	4	431.26	29.50
3	2	−4	372.46	22.00

2	−2	−4	171.18	15.40
2	−2	−4	180.18	16.30
−2	−2	4	148.89	7.90
−2	−2	4	148.69	12.60
2	−2	−4	155.78	11.10
−2	−2	4	169.38	8.40
2	−2	−4	159.08	14.20
2	−2	−4	171.08	13.60
−2	2	4	133.89	17.80
2	2	−4	186.28	13.60
−2	2	4	171.78	14.10
2	2	−4	160.18	15.50
−2	2	4	149.19	9.90
−2	2	4	181.68	15.50
2	2	−4	165.18	8.70
−1	−2	4	246.38	14.10
1	−2	−4	229.28	17.00
1	−2	−4	227.78	14.50
1	−2	−4	262.17	15.60
1	−2	−4	238.38	12.10
−1	−2	4	248.38	8.90
1	−2	−4	245.98	17.30
1	2	−4	231.18	8.30
1	2	−4	253.87	15.60
−1	2	4	246.08	12.20
−1	2	4	247.68	18.00
−1	2	4	263.17	17.50
1	2	−4	241.68	9.50
−1	2	4	259.07	14.70
1	2	−4	230.08	17.30
0	−2	−4	1305.27	36.90

0	−2	−4	1314.87	45.00
0	−2	−4	1359.16	40.70
0	−2	4	1378.26	30.60
0	−2	−4	1436.46	44.30
0	−2	4	1356.16	30.50
0	−2	4	1366.06	39.80
0	2	−4	1388.96	33.90
0	2	−4	1368.76	32.30
0	2	4	1358.56	36.70
0	2	4	1273.17	43.10
0	2	4	1367.06	36.40
0	2	4	1435.06	46.90
0	2	4	1358.16	40.50
0	2	−4	1415.86	38.50
−1	−2	−4	4.90	2.40
−1	−2	−4	10.90	3.50
−1	−2	−4	7.20	3.50
−1	−2	−4	8.20	3.00
1	−2	4	0.30	2.00
−1	−2	−4	0.20	4.10
−1	−2	−4	6.20	3.90
−1	2	−4	9.60	1.80
1	2	4	10.60	3.80
1	2	4	9.20	2.70
−1	2	−4	14.60	2.50
1	2	4	3.60	3.40
−1	2	−4	17.10	3.20
1	2	4	7.80	4.30
−1	2	−4	11.30	3.30
1	2	4	9.70	3.00
−1	2	−4	5.60	2.60

−2	−2	−4	590.24	25.50
−2	−2	−4	535.25	25.00
−2	−2	−4	550.54	18.20
−2	−2	−4	556.84	19.50
−2	−2	−4	540.25	25.90
−2	2	−4	566.54	15.90
−2	2	−4	551.14	20.30
−2	2	−4	533.85	16.00
−2	2	−4	490.25	27.90
−2	2	−4	569.24	19.10
2	2	4	555.84	24.70
−2	2	−4	549.54	15.00
2	2	4	572.74	20.40
2	2	4	561.74	19.00
−3	−2	−4	165.58	9.90
−3	−2	−4	182.18	14.90
3	−2	4	165.88	6.30
−3	−2	−4	148.99	8.70
3	−2	4	157.68	14.60
−3	−2	−4	156.18	14.30
3	−2	4	151.98	5.50
3	−2	4	144.19	10.60
−3	−2	−4	149.39	13.50
−3	−2	−4	153.88	9.20
3	2	4	179.88	9.90
3	2	4	160.08	9.20
−3	2	−4	166.68	15.50
3	2	4	140.99	15.50
3	2	4	183.08	14.70
−3	2	−4	154.48	5.10
3	2	4	176.78	16.80

−3	2	−4	146.09	12.00
−3	2	−4	166.68	7.60
−3	2	−4	151.68	7.80
4	−2	4	87.89	11.00
−4	−2	−4	72.39	6.30
−4	−2	−4	67.99	12.60
4	−2	4	94.49	5.20
4	−2	4	81.59	5.70
−4	−2	−4	103.79	15.20
4	2	4	68.19	6.90
4	2	4	65.29	14.00
4	2	4	83.59	7.30
4	2	4	101.69	15.10
−4	2	−4	77.69	6.60
−4	2	−4	62.49	5.50
4	2	4	85.69	8.90
−4	2	−4	87.79	10.60
−4	2	−4	62.19	12.80
−5	−2	−4	72.99	6.80
−5	−2	−4	57.69	13.30
−5	−2	−4	74.89	13.90
5	−2	4	85.99	6.10
5	−2	4	75.09	6.70
5	2	4	77.49	9.80
−5	2	−4	70.39	7.70
−5	2	−4	73.89	14.70
5	2	4	93.99	8.10
−5	2	−4	67.89	11.30
−6	−2	−4	507.55	33.60
6	−2	4	573.84	19.70
−6	−2	−4	570.04	19.90

−6	−2	−4	538.65	33.40
6	−2	4	570.24	19.40
−6	2	−4	538.05	35.00
−6	2	−4	592.84	30.40
6	2	4	594.64	23.30
6	2	4	579.84	20.60
7	−2	4	6.00	4.30
−7	−2	−4	−5.50	7.70
−7	−2	−4	6.90	9.10
7	2	4	7.80	4.80
−7	2	−4	−5.30	7.30
7	2	4	3.20	5.20
−7	2	−4	3.40	9.80
8	−2	4	3.20	7.10
−8	−2	−4	2.80	10.80
8	−2	4	−0.50	8.10
−8	−2	−4	−1.20	12.80
8	2	4	−4.30	5.30
−8	2	−4	2.50	9.20
8	2	4	−4.90	5.20
−8	2	−4	2.90	10.80
−9	−2	−4	6.70	12.70
9	−2	4	17.50	7.10
−9	−2	−4	17.90	14.60
−9	2	−4	7.50	12.70
−9	2	−4	10.10	15.10
−10	−2	−4	46.20	20.40
−10	−2	−4	−3.10	16.20
10	−2	4	22.60	8.30
10	2	4	1.30	9.20
−10	2	−4	35.90	21.80

−10	2	−4	20.50	15.40
−11	−2	−4	−6.40	19.40
11	−2	4	37.10	11.00
−11	−2	−4	9.80	19.40
−11	2	−4	−17.50	20.00
−11	2	−4	−5.20	23.00
11	2	4	−15.30	10.90
−11	−3	4	−7.20	23.40
−11	−3	4	1.20	20.90
11	−3	−4	12.30	15.80
11	−3	−4	−13.60	10.00
−11	−3	4	9.00	23.30
11	3	−4	−9.50	10.80
−11	3	4	17.20	23.20
−11	3	4	3.10	21.50
−11	3	4	−3.20	18.50
10	−3	−4	−10.70	13.50
−10	−3	4	−4.20	18.90
10	−3	−4	−6.10	8.60
−10	−3	4	14.40	18.70
10	3	−4	1.00	9.80
−10	3	4	9.30	16.90
−10	3	4	24.80	20.50
−10	3	4	20.00	16.60
9	−3	−4	−5.50	8.10
9	−3	−4	14.40	11.60
−9	−3	4	45.90	14.60
−9	−3	4	24.90	18.30
−9	−3	4	29.70	18.50
−9	3	4	12.10	16.10
−9	3	4	40.20	17.70

9	3	−4	19.80	9.20
−9	3	4	0.80	13.00
−8	−3	4	104.49	22.40
8	−3	−4	93.59	12.20
8	−3	−4	112.99	17.10
−8	3	4	159.48	27.20
−8	3	4	125.69	24.40
7	−3	−4	28.20	8.00
7	−3	−4	28.60	9.30
−7	−3	4	14.50	12.90
−7	−3	4	3.70	12.70
−7	3	4	26.40	12.20
7	3	−4	15.70	6.60
−7	3	4	32.50	12.60
−7	3	4	20.80	12.20
−6	−3	4	78.49	18.20
6	−3	−4	46.60	13.30
6	−3	−4	83.79	11.50
−6	−3	4	95.29	13.50
−6	−3	4	65.89	16.00
−6	3	4	89.99	18.30
−6	3	4	80.49	17.20
−6	3	4	58.19	14.90
6	3	−4	62.79	9.70
−5	−3	4	6.40	5.60
5	−3	−4	5.60	6.60
−5	−3	4	4.10	7.40
−5	−3	4	15.30	9.30
−5	3	4	8.80	7.00
−5	3	4	9.10	7.20
−5	3	4	11.10	8.50

5	3	−4	7.90	4.30
−4	−3	4	522.75	32.90
−4	−3	4	523.65	20.90
4	−3	−4	484.95	25.40
4	−3	−4	550.34	28.90
−4	−3	4	533.65	29.40
−4	−3	4	512.95	19.40
4	−3	−4	541.45	21.70
4	3	−4	535.15	29.40
−4	3	4	510.25	28.40
−4	3	4	472.65	29.10
4	3	−4	464.35	19.40
−4	3	4	533.45	32.10
3	−3	−4	180.88	14.60
3	−3	−4	182.78	11.60
3	−3	−4	178.68	15.20
−3	−3	4	176.98	9.20
−3	−3	4	133.59	18.90
−3	−3	4	175.98	9.50
3	−3	−4	213.08	19.40
−3	−3	4	169.28	9.50
3	−3	−4	187.68	19.20
−3	−3	4	145.79	14.40
−3	3	4	173.48	11.90
3	3	−4	210.58	13.60
−3	3	4	185.18	15.60
3	3	−4	173.58	9.90
−3	3	4	164.08	16.80
3	3	−4	165.18	15.40
3	3	−4	204.98	18.70
−3	3	4	187.28	18.00

2	−3	−4	424.66	21.30
−2	−3	4	405.06	20.60
2	−3	−4	402.36	26.30
−2	−3	4	401.56	13.80
2	−3	−4	419.36	21.60
2	−3	−4	417.86	19.70
−2	3	4	409.76	26.50
−2	3	4	405.66	23.90
2	3	−4	389.86	20.10
2	3	−4	430.36	15.10
2	3	−4	428.86	26.00
−2	3	4	418.76	25.10
−2	3	4	403.96	19.60
−1	−3	4	581.54	23.10
1	−3	−4	575.54	30.00
−1	−3	4	593.64	15.90
1	−3	−4	612.34	24.30
1	−3	−4	618.44	30.00
1	−3	−4	616.34	21.60
−1	3	4	564.14	29.50
1	3	−4	595.64	17.40
−1	3	4	612.24	21.40
1	3	−4	603.44	22.50
−1	3	4	604.24	25.30
1	3	−4	620.44	22.30
1	3	−4	619.64	29.40
0	−3	−4	191.78	12.80
0	−3	−4	173.08	10.00
0	−3	−4	185.88	15.20
0	−3	−4	173.28	15.30
0	3	−4	185.88	9.50

0	3	4	176.08	10.00
0	3	4	184.88	12.40
0	3	−4	192.98	10.30
0	3	4	191.68	15.60
0	3	−4	191.18	6.80
0	3	−4	188.88	8.30
0	3	4	200.98	10.50
0	3	4	183.08	15.20
−1	−3	−4	312.97	19.60
−1	−3	−4	312.77	12.90
−1	−3	−4	299.67	16.60
−1	−3	−4	305.27	15.40
−1	−3	−4	328.37	18.90
−1	−3	−4	291.27	18.80
−1	3	−4	306.37	11.30
−1	3	−4	319.97	12.70
−1	3	−4	315.07	8.30
1	3	4	303.67	15.30
1	3	4	307.77	13.40
−1	3	−4	320.47	8.60
−1	3	−4	310.97	10.90
1	3	4	300.57	18.70
−2	−3	−4	468.55	23.80
−2	−3	−4	472.45	18.20
−2	−3	−4	437.26	23.90
−2	−3	−4	445.66	22.40
−2	−3	−4	443.86	18.30
−2	−3	−4	452.55	17.60
−2	−3	−4	467.75	24.80
2	3	4	465.35	17.40
−2	3	−4	462.45	14.40

-2	3	-4	443.46	14.30
2	3	4	418.16	23.20
2	3	4	451.45	18.60
-2	3	-4	447.66	14.70
2	3	4	440.96	21.70
-2	3	-4	440.36	12.70
-2	3	-4	439.36	16.10
-3	-3	-4	129.89	16.40
-3	-3	-4	117.89	12.80
3	-3	4	117.29	9.00
-3	-3	-4	157.38	14.10
-3	-3	-4	128.89	8.90
-3	-3	-4	128.09	9.20
-3	-3	-4	127.49	9.30
3	-3	4	113.69	15.10
3	3	4	108.69	8.70
-3	3	-4	98.09	8.50
3	3	4	126.39	15.80
3	3	4	124.29	16.80
3	3	4	119.09	8.50
3	3	4	114.39	12.50
-3	3	-4	121.69	5.90
3	3	4	139.99	9.80
-3	3	-4	117.39	6.20
-3	3	-4	124.99	8.40
-3	3	-4	112.09	6.10
-3	3	-4	135.09	13.60
-4	-3	-4	807.32	26.70
-4	-3	-4	830.72	38.20
4	-3	4	812.12	29.30
-4	-3	-4	824.52	37.60

4	3	4	823.42	37.60
−4	3	−4	828.52	38.00
−4	3	−4	809.02	23.30
4	3	4	826.62	27.30
−4	3	−4	809.62	28.80
4	3	4	803.82	27.20
−4	3	−4	817.02	23.40
5	−3	4	467.25	13.30
5	−3	4	469.85	12.80
−5	−3	−4	496.25	30.30
−5	−3	−4	467.95	29.90
−5	−3	−4	430.76	21.00
5	3	4	456.05	19.40
5	3	4	502.95	20.60
−5	3	−4	472.95	29.80
−5	3	−4	468.25	23.20
−5	3	−4	426.46	16.90
6	−3	4	−1.30	2.60
−6	−3	−4	−2.10	7.90
−6	−3	−4	6.30	7.30
6	−3	4	7.40	3.30
6	3	4	−3.50	3.50
−6	3	−4	−0.40	5.90
6	3	4	4.80	4.60
−6	3	−4	6.00	8.70
7	−3	4	72.29	7.60
−7	−3	−4	57.69	14.90
−7	−3	−4	79.99	18.10
−7	3	−4	81.49	18.20
−7	3	−4	76.39	14.80
7	3	4	47.20	8.90

8	−3	4	122.49	9.70
8	−3	4	129.29	11.60
−8	−3	−4	132.69	23.90
−8	3	−4	90.59	21.60
8	3	4	159.88	16.60
8	3	4	123.99	13.00
−8	3	−4	122.49	19.50
−9	−3	−4	−1.00	11.40
9	−3	4	5.80	5.90
−9	−3	−4	−23.40	13.60
−9	3	−4	−3.70	12.00
9	3	4	−4.20	7.30
−9	3	−4	−1.20	16.60
10	−3	4	8.00	8.00
−10	−3	−4	17.80	19.50
−10	−3	−4	18.70	16.80
−10	3	−4	−10.50	15.10
−10	3	−4	6.40	14.00
10	3	4	14.60	9.40
−11	−3	−4	24.40	19.50
−11	−3	−4	−12.30	18.80
−11	3	−4	18.90	21.20
−11	3	−4	7.60	17.70
11	3	4	9.80	11.20
−11	−4	4	−6.30	19.10
−11	−4	4	28.30	23.30
−11	−4	4	−7.20	20.60
11	−4	−4	−31.40	15.70
11	−4	−4	−8.60	11.50
−11	4	4	23.60	19.20
11	4	−4	4.90	12.00

-11	4	4	-50.89	22.90
-11	4	4	17.40	22.30
-10	-4	4	31.20	22.60
10	-4	-4	13.60	12.20
-10	-4	4	26.20	17.60
10	-4	-4	10.80	10.20
10	4	-4	12.20	9.60
-10	4	4	-8.40	18.00
-10	4	4	0.60	15.60
-10	4	4	26.60	19.30
-9	-4	4	17.10	17.90
-9	-4	4	9.60	16.10
9	-4	-4	9.90	11.30
9	-4	-4	-9.90	7.40
9	4	-4	6.30	7.60
-9	4	4	4.50	16.90
-9	4	4	26.50	15.80
-9	4	4	25.60	17.60
8	-4	-4	14.90	10.00
-8	-4	4	10.10	15.30
-8	-4	4	-13.30	10.80
8	-4	-4	9.60	8.40
-8	4	4	27.20	14.90
-8	4	4	9.90	12.60
-8	4	4	34.20	17.60
8	4	-4	40.50	10.30
-7	-4	4	32.50	15.80
7	-4	-4	16.10	6.70
7	-4	-4	0.00	8.50
-7	-4	4	10.90	10.00
-7	4	4	2.00	10.00

−7	4	4	5.20	9.50
7	4	−4	17.20	7.00
−7	4	4	−2.70	11.60
−6	−4	4	−3.10	9.30
6	−4	−4	−13.70	6.90
−6	−4	4	5.10	8.50
−6	−4	4	8.80	7.50
6	−4	−4	−1.90	5.00
6	4	−4	2.00	4.90
−6	4	4	1.60	8.20
−6	4	4	13.50	8.50
−6	4	4	6.50	9.70
5	−4	−4	171.78	16.80
−5	−4	4	151.38	23.40
−5	−4	4	142.89	19.30
−5	−4	4	128.69	12.80
−5	4	4	160.38	20.60
−5	4	4	148.19	22.30
−5	4	4	120.49	20.10
5	4	−4	129.79	10.80
−4	−4	4	605.44	22.20
−4	−4	4	610.94	32.10
4	−4	−4	622.84	24.40
4	−4	−4	612.84	29.70
−4	−4	4	595.74	36.40
4	−4	−4	622.24	33.60
−4	−4	4	604.44	21.60
4	4	−4	647.44	23.20
−4	4	4	593.54	30.10
−4	4	4	596.34	34.40
3	−4	−4	536.85	33.80

-3	-4	4	499.15	31.30
3	-4	-4	542.55	26.30
3	-4	-4	551.44	21.90
-3	-4	4	530.35	18.10
-3	-4	4	528.15	21.30
3	-4	-4	494.35	24.70
-3	-4	4	555.74	27.20
-3	4	4	487.75	31.80
3	4	-4	561.94	27.00
3	4	-4	542.35	19.60
-3	4	4	570.94	27.30
-3	4	4	546.65	31.00
-3	4	4	535.65	25.80
-3	4	4	557.34	22.10
3	4	-4	512.75	30.50
2	-4	-4	78.69	13.20
2	-4	-4	92.89	10.00
2	-4	-4	138.59	19.90
-2	-4	4	96.99	5.90
-2	-4	4	94.59	11.30
2	-4	-4	80.59	11.60
2	-4	-4	117.09	9.10
2	4	-4	95.49	6.80
-2	4	4	92.69	11.70
-2	4	4	90.89	10.10
2	4	-4	94.79	9.90
-2	4	4	96.69	15.40
-2	4	4	77.69	14.50
2	4	-4	103.39	11.80
1	-4	-4	51.49	9.30
1	-4	-4	53.19	11.70

-1	-4	4	42.40	7.30
1	-4	-4	55.29	11.10
1	-4	-4	60.09	8.30
-1	-4	4	59.69	3.30
1	-4	-4	63.09	8.50
1	4	-4	48.20	4.20
1	4	-4	55.69	7.70
-1	4	4	70.69	13.90
1	4	-4	47.90	9.80
-1	4	4	73.29	13.30
-1	4	4	61.09	9.60
0	-4	-4	4159.28	98.59
0	-4	-4	3791.32	96.39
0	-4	-4	3913.51	90.89
0	4	-4	3921.11	91.29
0	4	-4	3867.21	86.59
0	4	-4	3832.22	89.69
0	4	-4	3894.31	82.69
0	4	4	3850.91	105.19
0	4	4	3807.02	105.99
0	4	4	3805.32	91.19
-1	-4	-4	315.17	14.40
-1	-4	-4	304.67	16.40
1	-4	4	307.97	11.00
1	-4	4	288.37	13.10
-1	-4	-4	297.57	23.50
-1	-4	-4	297.77	22.50
-1	-4	-4	289.87	16.10
-1	-4	-4	301.87	19.70
1	4	4	346.27	17.50
1	4	4	314.27	14.10

1	4	4	307.27	13.90
-1	4	-4	296.57	13.40
1	4	4	285.47	19.90
2	-4	4	34.80	4.70
-2	-4	-4	29.30	4.90
-2	-4	-4	33.10	8.70
-2	-4	-4	38.40	9.00
-2	-4	-4	55.69	11.40
-2	-4	-4	32.60	5.80
-2	-4	-4	31.90	5.90
-2	-4	-4	32.20	7.10
2	4	4	29.50	5.50
2	4	4	29.50	4.80
-2	4	-4	42.00	5.20
2	4	4	31.80	7.20
2	4	4	43.40	8.60
2	4	4	30.70	8.40
-3	-4	-4	410.66	25.40
-3	-4	-4	435.16	24.40
-3	-4	-4	416.06	18.10
-3	-4	-4	401.66	18.60
3	-4	4	410.56	15.10
-3	4	-4	408.36	11.90
-3	4	-4	395.16	11.60
-3	4	-4	385.86	14.50
3	4	4	431.26	26.80
-3	4	-4	401.56	14.10
3	4	4	392.16	17.30
-3	4	-4	403.86	23.90
-3	4	-4	401.96	13.90
3	4	4	426.66	25.20

3	4	4	392.66	24.30
4	−4	4	338.77	16.30
−4	−4	−4	344.67	17.00
−4	−4	−4	314.47	23.10
4	4	4	350.36	24.40
−4	4	−4	317.17	23.80
−4	4	−4	332.97	15.90
4	4	4	351.96	16.90
4	4	4	350.56	17.40
−4	4	−4	346.67	12.40
−5	−4	−4	90.49	9.30
−5	−4	−4	94.99	15.50
−5	−4	−4	97.49	18.50
−5	4	−4	96.59	16.40
−5	4	−4	92.99	7.80
−5	4	−4	89.49	11.60
5	4	4	74.29	9.50
5	4	4	110.29	10.70
5	4	4	116.69	17.80
−6	−4	−4	35.20	12.90
−6	−4	−4	50.19	13.90
−6	−4	−4	48.20	8.10
6	4	4	32.00	7.90
−6	4	−4	74.09	17.10
6	4	4	65.29	9.60
−6	4	−4	70.19	12.10
−7	−4	−4	−10.70	8.40
−7	−4	−4	0.00	11.50
−7	−4	−4	6.20	5.10
7	4	4	−9.70	7.00
−7	4	−4	11.20	9.80

-7	4	-4	-7.80	6.50
7	4	4	0.10	5.50
-8	-4	-4	18.30	12.70
-8	-4	-4	21.80	17.30
-8	4	-4	35.20	12.00
-8	4	-4	31.40	15.80
-9	-4	-4	62.89	19.40
-9	-4	-4	14.70	15.60
9	-4	4	24.80	5.40
9	4	4	29.90	10.00
-9	4	-4	32.60	13.80
-9	4	-4	45.50	19.50
-10	-4	-4	-16.00	16.30
-10	-4	-4	19.80	18.00
-10	4	-4	18.90	14.70
-10	4	-4	-8.80	16.50
10	4	4	23.40	10.70
-11	-4	-4	-22.70	19.30
-11	-4	-4	34.50	24.20
11	4	4	-4.10	12.90
-11	4	-4	-12.30	19.30
-11	4	-4	-3.20	17.00
-11	4	-4	16.50	19.70
-10	-5	4	13.30	21.80
10	-5	-4	-7.90	10.70
-10	-5	4	-26.00	19.50
10	-5	-4	4.90	13.30
-10	5	4	32.70	17.60
10	5	-4	-0.60	11.20
-10	5	4	-16.20	18.80
-10	5	4	-7.10	16.60

9	−5	−4	−8.70	9.10
−9	−5	4	19.70	16.20
9	−5	−4	−8.30	7.60
−9	−5	4	−3.60	14.60
−9	5	4	−5.10	15.10
9	5	−4	4.30	7.60
−9	5	4	2.80	14.60
−9	5	4	23.20	16.90
8	−5	−4	39.40	13.20
−8	−5	4	67.39	17.60
−8	−5	4	79.49	20.70
8	−5	−4	78.29	12.30
−8	5	4	61.79	20.50
8	5	−4	63.09	12.70
−8	5	4	61.49	19.20
−8	5	4	28.70	16.00
−7	−5	4	48.20	17.90
−7	−5	4	67.79	19.20
7	−5	−4	38.00	12.20
7	−5	−4	42.50	9.60
7	5	−4	31.00	8.80
−7	5	4	28.00	13.20
−7	5	4	41.60	18.90
−7	5	4	58.19	16.80
6	−5	−4	34.70	8.50
−6	−5	4	43.80	11.00
−6	−5	4	61.69	15.60
6	−5	−4	53.59	12.00
−6	−5	4	44.20	16.60
−6	5	4	60.89	15.40
−6	5	4	63.09	17.40

−6	5	4	46.80	15.30
6	5	−4	52.49	10.20
−5	−5	4	217.28	15.20
−5	−5	4	213.78	21.40
−5	−5	4	213.48	29.00
5	−5	−4	222.88	21.60
5	−5	−4	243.68	17.10
5	−5	−4	199.88	17.60
−5	5	4	264.77	32.30
5	5	−4	234.88	15.10
−5	5	4	164.88	18.10
−5	5	4	204.68	25.60
4	−5	−4	283.67	21.20
−4	−5	4	331.37	23.70
−4	−5	4	326.77	14.60
−4	−5	4	287.27	14.90
−4	−5	4	332.97	27.70
4	−5	−4	315.77	16.90
4	−5	−4	316.17	22.00
−4	5	4	338.17	29.40
−4	5	4	285.37	24.90
4	5	−4	302.17	23.20
−4	5	4	338.17	21.90
−3	−5	4	109.49	17.00
−3	−5	4	134.29	8.20
3	−5	−4	117.29	12.00
3	−5	−4	148.39	10.90
3	−5	−4	126.19	13.10
−3	−5	4	111.49	14.40
3	5	−4	155.48	21.70
3	5	−4	149.29	16.80

-3	5	4	132.09	12.40
-3	5	4	102.49	22.20
-3	5	4	184.38	22.60
3	5	-4	107.19	8.60
2	-5	-4	259.77	18.20
2	-5	-4	277.77	15.30
2	-5	-4	272.47	14.30
-2	-5	4	271.87	19.20
-2	-5	4	290.87	9.80
-2	-5	4	281.17	10.50
-2	5	4	247.98	23.60
-2	5	4	295.17	19.30
-2	5	4	312.77	24.00
2	5	-4	292.27	12.10
2	5	-4	248.98	16.20
-2	5	4	274.07	15.10
2	5	-4	270.67	21.20
1	-5	-4	382.66	16.50
-1	-5	4	374.26	18.10
-1	-5	4	359.26	17.80
1	-5	-4	391.66	24.90
1	-5	-4	410.66	17.00
1	-5	-4	425.76	22.00
1	-5	-4	335.17	25.80
1	5	-4	387.36	13.10
1	5	-4	382.36	18.30
-1	5	4	390.06	17.70
-1	5	4	389.06	25.20
1	5	-4	335.67	23.20
-1	5	4	383.96	17.00
1	5	-4	367.96	16.90

-1	5	4	372.06	27.00
1	5	-4	389.56	13.00
-1	5	4	391.96	21.10
0	-5	-4	176.18	18.00
0	-5	4	166.18	11.60
0	-5	-4	177.48	10.40
0	-5	-4	187.28	17.40
0	-5	-4	178.58	11.00
0	-5	-4	188.58	14.10
0	5	4	149.88	10.70
0	5	4	169.18	16.90
0	5	-4	176.88	6.30
0	5	-4	175.48	8.30
0	5	4	148.59	16.30
0	5	4	163.58	10.60
0	5	-4	171.38	12.20
0	5	-4	153.58	12.50
0	5	4	135.19	12.30
-1	-5	-4	1587.64	41.80
-1	-5	-4	1503.65	52.69
-1	-5	-4	1660.23	59.99
-1	-5	-4	1533.05	44.80
1	-5	4	1503.75	46.00
-1	-5	-4	1441.56	46.80
-1	-5	-4	1535.25	42.50
-1	-5	-4	1529.55	55.39
1	5	4	1505.85	55.79
-1	5	-4	1495.75	47.50
1	5	4	1567.04	47.10
1	5	4	1560.24	43.20
-1	5	-4	1592.04	43.00

1	5	4	1525.25	42.20
−2	−5	−4	357.26	23.40
−2	−5	−4	347.77	19.30
−2	−5	−4	349.77	18.30
−2	−5	−4	315.77	14.80
2	−5	4	323.87	14.60
−2	−5	−4	301.97	15.20
2	5	4	366.96	23.60
2	5	4	326.97	15.90
−2	5	−4	341.07	14.80
−2	5	−4	308.47	20.70
2	5	4	306.07	17.80
2	5	4	315.27	17.90
−3	−5	−4	321.37	23.80
3	−5	4	303.27	11.00
−3	−5	−4	311.87	17.40
3	−5	4	311.77	13.30
−3	−5	−4	291.87	20.20
3	5	4	347.97	24.30
3	5	4	297.27	16.90
−3	5	−4	317.37	13.50
3	5	4	262.97	29.60
3	5	4	249.78	20.80
−3	5	−4	301.27	9.40
−3	5	−4	307.57	20.80
−3	5	−4	300.37	8.70
4	−5	4	677.03	23.50
−4	−5	−4	683.93	34.10
−4	−5	−4	665.13	36.40
4	−5	4	702.13	21.00
−4	−5	−4	709.03	28.10

4	5	4	754.62	37.10
−4	5	−4	710.63	22.00
−4	5	−4	680.73	21.90
−4	5	−4	695.83	35.20
−4	5	−4	736.63	19.90
4	5	4	674.03	31.80
4	5	4	692.83	26.30
−5	−5	−4	111.29	12.80
−5	−5	−4	101.99	18.40
−5	−5	−4	103.89	18.60
5	5	4	113.29	12.50
−5	5	−4	138.09	19.30
−5	5	−4	113.69	12.40
−5	5	−4	124.89	11.10
−5	5	−4	114.09	8.30
5	5	4	93.39	17.60
−6	−5	−4	35.10	14.80
−6	−5	−4	31.00	12.60
−6	−5	−4	28.00	7.80
−6	5	−4	27.10	7.70
6	5	4	9.20	6.40
−6	5	−4	42.80	13.60
−7	−5	−4	97.79	21.30
−7	−5	−4	91.49	12.30
−7	−5	−4	107.59	21.70
−7	5	−4	109.79	20.50
−7	5	−4	122.49	16.20
7	5	4	126.49	14.60
−8	−5	−4	49.90	18.40
−8	−5	−4	76.29	22.30
−8	5	−4	47.80	13.30

−8	5	−4	72.29	21.10
8	5	4	68.49	11.30
−9	−5	−4	21.40	17.30
−9	−5	−4	13.00	15.90
−9	5	−4	−7.00	14.10
−9	5	−4	15.50	12.80
−9	5	−4	18.50	19.10
9	5	4	1.70	9.20
−10	−5	−4	8.20	16.90
−10	−5	−4	13.20	20.40
−10	5	−4	−2.10	12.40
−10	5	−4	−6.30	18.50
10	5	4	−2.30	10.20
−10	5	−4	−5.60	16.60
−10	−6	4	13.10	21.20
10	−6	−4	−19.90	10.20
10	−6	−4	−7.60	13.40
−10	−6	4	−4.30	21.20
−10	6	4	15.80	20.40
−10	6	4	−0.80	24.60
−10	6	4	−13.80	18.40
−9	−6	4	17.20	17.60
9	−6	−4	13.70	10.30
−9	−6	4	−2.80	18.80
9	−6	−4	19.90	12.70
−9	6	4	25.90	20.40
−9	6	4	35.80	16.90
−9	6	4	1.40	17.80
9	6	−4	14.90	9.50
−8	−6	4	22.20	16.60
8	−6	−4	11.00	10.30

8	−6	−4	15.40	9.10
−8	−6	4	−9.30	13.90
8	6	−4	27.90	9.80
−8	6	4	−1.30	13.60
−8	6	4	16.20	13.00
−8	6	4	21.90	17.40
7	−6	−4	24.00	9.00
−7	−6	4	5.40	14.20
−7	−6	4	18.60	12.40
7	−6	−4	21.60	8.60
−7	6	4	24.50	12.60
−7	6	4	16.30	13.00
7	6	−4	25.40	7.70
−7	6	4	30.20	17.70
6	−6	−4	189.98	19.20
−6	−6	4	227.68	33.30
6	−6	−4	219.18	15.60
−6	−6	4	173.98	18.10
−6	−6	4	240.08	30.40
−6	6	4	207.58	31.40
−6	6	4	202.88	22.10
−6	6	4	201.28	28.60
6	6	−4	186.38	15.00
5	−6	−4	100.29	16.70
5	−6	−4	86.19	11.00
−5	−6	4	99.39	22.80
−5	−6	4	106.89	12.70
−5	−6	4	106.19	18.90
5	−6	−4	103.09	14.20
−5	6	4	104.49	15.60
−5	6	4	64.49	17.50

-5	6	4	97.09	18.40
5	6	-4	112.09	13.00
-4	-6	4	200.88	13.80
-4	-6	4	225.38	12.40
-4	-6	4	235.08	20.90
4	-6	-4	248.98	14.60
4	-6	-4	229.88	19.40
-4	-6	4	208.68	26.70
4	-6	-4	236.58	18.10
-4	6	4	229.68	30.70
4	6	-4	231.18	14.20
4	6	-4	214.88	25.60
-4	6	4	186.38	23.90
-3	-6	4	554.64	28.60
-3	-6	4	562.34	18.60
3	-6	-4	531.35	24.20
-3	-6	4	557.34	21.60
-3	-6	4	562.44	34.30
3	-6	-4	565.64	22.20
3	-6	-4	556.64	29.00
3	6	-4	559.74	20.80
-3	6	4	568.64	39.60
-3	6	4	548.55	34.40
3	6	-4	555.74	28.00
2	-6	-4	308.37	20.80
-2	-6	4	303.97	10.80
-2	-6	4	320.27	14.80
2	-6	-4	305.57	15.20
2	-6	-4	293.07	14.80
-2	6	4	345.47	29.20
2	6	-4	305.07	11.90

2	6	-4	290.17	17.70
-2	6	4	313.27	29.20
2	6	-4	346.77	24.90
-1	-6	4	179.18	13.00
1	-6	-4	201.78	15.80
1	-6	-4	161.78	10.30
-1	-6	4	159.78	11.90
1	-6	-4	175.28	9.60
1	-6	-4	151.78	10.50
1	6	-4	170.98	8.20
-1	6	4	174.38	21.80
-1	6	4	150.48	11.30
1	6	-4	150.78	11.10
1	6	-4	166.58	10.00
-1	6	4	163.98	13.30
-1	6	4	132.39	20.20
0	-6	-4	86.39	9.40
0	-6	-4	47.40	9.20
0	-6	4	69.49	9.10
0	-6	-4	67.79	7.10
0	-6	4	79.49	8.30
0	6	-4	76.39	4.50
0	6	-4	71.29	5.90
0	6	4	76.39	9.30
0	6	-4	57.69	8.50
0	6	-4	76.09	11.10
0	6	4	65.49	13.10
0	6	4	107.59	18.70
1	-6	4	76.39	9.30
-1	-6	-4	68.29	10.30
-1	-6	-4	82.89	9.00

−1	−6	−4	59.19	8.50
−1	−6	−4	69.89	7.90
−1	−6	−4	54.69	12.50
1	6	4	68.09	9.40
1	6	4	41.20	11.40
−1	6	−4	67.49	8.30
−1	6	−4	65.49	11.10
−2	−6	−4	334.27	18.80
−2	−6	−4	329.77	24.40
2	−6	4	336.47	16.30
−2	−6	−4	323.17	16.20
−2	−6	−4	298.37	18.10
−2	6	−4	329.27	16.40
2	6	4	336.77	19.30
2	6	4	318.37	18.10
2	6	4	366.36	28.90
−2	6	−4	335.87	22.50
−2	6	−4	311.07	21.40
3	−6	4	249.38	12.90
−3	−6	−4	241.08	18.00
−3	−6	−4	213.78	18.60
−3	−6	−4	256.17	23.20
3	6	4	236.28	21.00
−3	6	−4	256.57	22.10
−3	6	−4	262.87	13.50
3	6	4	238.98	14.40
3	6	4	262.07	18.70
−3	6	−4	222.48	18.60
4	−6	4	−0.30	3.00
4	−6	4	1.10	3.70
−4	−6	−4	−0.70	4.50

−4	−6	−4	−2.50	6.50
−4	−6	−4	2.90	8.80
−4	6	−4	1.80	2.30
−4	6	−4	12.40	7.80
−4	6	−4	12.30	4.00
4	6	4	0.50	4.80
−4	6	−4	−0.20	8.00
4	6	4	0.60	7.30
5	−6	4	151.98	9.00
−5	−6	−4	165.88	24.60
−5	−6	−4	150.68	13.80
−5	−6	−4	140.89	20.40
−5	6	−4	151.88	11.20
−5	6	−4	126.29	8.70
5	6	4	125.09	11.70
−5	6	−4	155.98	23.00
−5	6	−4	110.29	10.00
5	6	4	134.79	20.00
−5	6	−4	110.99	17.30
−6	−6	−4	362.06	21.50
−6	−6	−4	413.06	32.00
−6	−6	−4	369.96	33.00
−6	6	−4	381.86	29.10
−6	6	−4	358.56	33.00
6	6	4	368.96	30.00
6	6	4	375.66	21.00
−6	6	−4	340.57	19.80
−7	−6	−4	10.30	12.70
−7	−6	−4	7.70	7.90
−7	−6	−4	12.40	12.50
−7	6	−4	13.80	13.90

−7	6	−4	14.50	7.60
−7	6	−4	13.20	11.70
−8	−6	−4	10.00	12.30
−8	−6	−4	21.20	15.10
−8	−6	−4	16.50	8.50
−8	6	−4	−17.50	13.00
8	6	4	−6.10	7.70
−8	6	−4	2.70	8.70
−9	−6	−4	−7.20	16.80
−9	−6	−4	18.00	16.00
−9	6	−4	5.00	17.30
9	6	4	−0.20	8.70
−9	6	−4	52.09	19.50
−9	6	−4	−12.80	10.30
−10	−6	−4	21.80	22.30
−10	6	−4	39.20	24.10
10	6	4	8.00	12.20
−10	6	−4	14.80	14.30
−10	6	−4	49.30	22.30
−10	−7	4	−11.50	23.00
10	−7	−4	−9.40	11.40
10	−7	−4	43.10	17.30
−10	7	4	21.60	27.20
−10	7	4	−2.10	19.70
10	7	−4	7.50	12.60
−9	−7	4	−6.50	18.50
9	−7	−4	30.80	14.60
9	−7	−4	13.90	9.60
−9	−7	4	7.00	19.00
9	7	−4	31.00	11.90
−9	7	4	−2.90	19.90

-9	7	4	30.40	18.90
-9	7	4	-14.50	15.70
-8	-7	4	0.70	15.60
-8	-7	4	-7.80	17.10
8	-7	-4	23.40	9.40
8	-7	-4	18.00	12.20
8	7	-4	23.60	9.00
-8	7	4	5.10	17.80
-8	7	4	21.00	16.40
-8	7	4	-5.70	13.20
-7	-7	4	10.60	15.10
7	-7	-4	16.50	10.40
-7	-7	4	4.70	14.70
7	-7	-4	30.90	9.00
-7	7	4	4.80	12.00
7	7	-4	12.10	8.00
-7	7	4	-1.40	16.30
-6	-7	4	-7.70	11.50
6	-7	-4	-1.10	7.60
-6	-7	4	11.50	13.20
-6	-7	4	-14.00	8.50
6	-7	-4	13.80	6.40
-6	7	4	-6.90	9.80
6	7	-4	9.30	7.40
-6	7	4	-0.80	12.90
-6	7	4	-3.90	10.20
5	-7	-4	6.80	10.10
-5	-7	4	1.60	11.00
-5	-7	4	-7.90	7.20
-5	-7	4	-1.70	9.40
5	-7	-4	-5.70	7.00

5	−7	−4	13.70	5.80
−5	7	4	11.00	11.70
−5	7	4	0.60	6.40
5	7	−4	−0.80	5.00
−5	7	4	−9.60	7.90
−4	−7	4	25.80	7.40
−4	−7	4	15.00	9.80
4	−7	−4	25.60	8.30
4	−7	−4	15.50	8.50
−4	−7	4	2.40	9.40
−4	−7	4	9.90	5.00
4	−7	−4	22.60	5.80
−4	7	4	0.60	9.40
−4	7	4	17.80	10.40
−4	7	4	21.30	7.50
4	7	−4	12.30	4.70
4	7	−4	12.70	10.30
3	−7	−4	64.59	8.40
−3	−7	4	55.69	11.00
−3	−7	4	61.09	6.60
3	−7	−4	42.10	10.60
−3	−7	4	61.29	10.60
3	−7	−4	54.79	8.90
−3	−7	4	74.89	10.70
3	7	−4	76.49	9.20
−3	7	4	81.19	17.10
−3	7	4	67.59	18.40
3	7	−4	52.29	6.90
3	7	−4	67.69	11.40
2	−7	−4	431.16	15.50
2	−7	−4	426.86	25.30

−2	−7	4	425.46	22.90
2	−7	−4	427.56	18.20
−2	−7	4	450.15	19.20
2	7	−4	454.35	29.30
−2	7	4	432.36	29.50
2	7	−4	445.06	17.20
−2	7	4	416.66	34.70
2	7	−4	405.96	21.90
−1	−7	4	300.37	16.40
1	−7	−4	303.47	20.70
1	−7	−4	310.07	15.20
−1	−7	4	290.27	16.90
−1	−7	4	336.37	18.90
−1	7	4	365.16	26.90
1	7	−4	287.17	15.60
1	7	−4	315.97	12.30
1	7	−4	340.47	24.20
1	7	−4	312.97	16.20
−1	7	4	301.47	27.40
−1	7	4	323.27	17.80
0	−7	−4	29.80	8.00
0	−7	−4	54.89	7.60
0	−7	4	77.19	10.60
0	−7	−4	53.19	5.50
0	−7	−4	55.79	5.00
0	−7	−4	55.99	12.10
0	−7	4	49.00	7.10
0	7	−4	49.40	4.10
0	7	4	40.70	7.30
0	7	−4	34.70	9.20
0	7	−4	58.39	13.00

0	7	4	59.59	15.20
−1	−7	−4	234.68	13.60
−1	−7	−4	238.98	17.80
1	−7	4	230.58	15.00
−1	−7	−4	223.48	21.00
−1	7	−4	220.78	6.50
1	7	4	221.18	22.00
−1	7	−4	175.18	16.60
−2	−7	−4	430.56	21.70
−2	−7	−4	407.46	28.10
−2	−7	−4	398.46	22.60
2	−7	4	393.16	19.40
2	7	4	475.25	30.80
−2	7	−4	405.86	24.40
−2	7	−4	419.26	27.90
−2	7	−4	400.66	19.30
2	7	4	426.96	20.80
2	7	4	446.96	20.60
3	−7	4	26.30	6.10
−3	−7	−4	32.10	9.20
−3	−7	−4	45.70	9.90
−3	7	−4	31.60	11.20
−3	7	−4	20.10	8.10
3	7	4	39.30	11.30
3	7	4	25.40	10.80
−3	7	−4	21.70	5.10
3	7	4	39.90	8.60
−4	−7	−4	124.69	15.50
−4	−7	−4	167.78	24.40
4	−7	4	130.79	12.30
−4	−7	−4	144.59	19.30

4	7	4	134.59	11.90
−4	7	−4	126.49	18.30
−4	7	−4	111.89	6.60
−4	7	−4	127.19	21.30
4	7	4	130.79	21.10
−4	7	−4	126.69	11.10
−5	−7	−4	241.68	18.70
−5	−7	−4	333.67	26.50
5	−7	4	280.97	13.90
−5	−7	−4	265.77	31.20
−5	7	−4	259.37	27.60
−5	7	−4	255.87	15.90
−5	7	−4	265.67	12.10
−5	7	−4	280.17	24.00
5	7	4	260.87	17.10
5	7	4	254.97	25.20
6	−7	4	13.40	4.50
−6	−7	−4	3.80	6.80
−6	−7	−4	19.30	14.80
−6	−7	−4	−7.50	9.20
6	7	4	−11.00	5.30
−6	7	−4	5.80	9.80
−6	7	−4	−8.00	10.40
−6	7	−4	−16.40	5.90
−6	7	−4	4.00	5.60
6	7	4	−3.40	8.60
−7	−7	−4	25.60	9.30
−7	−7	−4	42.90	15.70
−7	7	−4	21.70	12.40
−7	7	−4	24.30	9.90
−7	7	−4	41.30	18.00

7	7	4	20.60	9.30
-8	-7	-4	20.30	15.00
-8	-7	-4	49.80	18.70
-8	-7	-4	39.90	10.40
-8	7	-4	17.20	19.90
8	7	4	19.80	10.30
-8	7	-4	13.20	11.30
-9	-7	-4	23.70	20.20
-9	-7	-4	-4.70	18.10
9	7	4	0.40	9.70
-9	7	-4	27.70	21.60
-9	7	-4	-2.10	15.60
-9	7	-4	0.50	13.00
-10	-7	-4	-0.80	26.70
-10	-7	-4	-24.60	22.60
10	7	4	11.80	12.90
-10	7	-4	23.50	22.20
-10	7	-4	-13.90	14.50
-10	7	-4	39.50	21.30
-9	-8	4	-10.00	21.80
9	-8	-4	11.10	9.70
-9	-8	4	-10.00	20.00
9	-8	-4	-18.10	12.90
-9	8	4	-17.60	15.50
9	8	-4	-1.70	10.70
-9	8	4	0.90	18.80
8	-8	-4	-5.40	10.70
8	-8	-4	6.00	7.30
-8	-8	4	-4.60	16.70
-8	8	4	-7.90	12.80
-8	8	4	19.70	21.80

8	8	−4	9.00	9.70
−8	8	4	−23.00	14.70
−7	−8	4	20.10	15.40
7	−8	−4	7.20	7.60
7	−8	−4	0.10	9.70
−7	−8	4	6.50	18.20
7	8	−4	−5.60	7.70
−7	8	4	11.20	16.20
−7	8	4	−3.00	10.90
−7	8	4	34.80	16.70
−6	−8	4	35.20	12.60
−6	−8	4	23.90	15.50
−6	−8	4	36.30	16.30
6	−8	−4	28.50	13.10
6	−8	−4	36.10	7.40
−6	8	4	18.60	14.40
6	8	−4	33.10	9.10
−6	8	4	40.10	14.80
−6	8	4	55.59	13.20
5	−8	−4	76.29	13.80
−5	−8	4	86.89	14.20
5	−8	−4	90.59	10.30
5	−8	−4	70.89	15.70
−5	−8	4	79.49	20.20
−5	−8	4	123.59	23.10
5	8	−4	99.29	12.70
−5	8	4	101.09	25.10
−5	8	4	87.79	15.20
−5	8	4	106.19	19.70
−4	−8	4	116.09	21.80
−4	−8	4	148.69	16.10

-4	-8	4	100.49	9.70
-4	-8	4	105.49	15.10
4	-8	-4	124.59	14.20
-4	-8	4	100.89	17.10
4	-8	-4	109.79	16.50
4	-8	-4	121.19	10.10
4	8	-4	122.59	11.30
-4	8	4	119.19	25.40
4	8	-4	143.09	23.00
-4	8	4	134.89	22.20
-3	-8	4	125.79	16.80
-3	-8	4	122.39	7.90
3	-8	-4	120.09	16.60
3	-8	-4	123.99	10.50
-3	-8	4	120.89	15.30
3	-8	-4	135.19	10.50
-3	-8	4	141.29	14.70
3	8	-4	149.59	11.50
-3	8	4	115.69	19.00
3	8	-4	145.79	18.60
3	8	-4	144.59	10.60
-3	8	4	137.99	25.30
2	-8	-4	0.40	5.50
-2	-8	4	0.40	4.60
-2	-8	4	-3.80	6.30
2	-8	-4	4.60	3.50
-2	8	4	9.20	10.10
2	8	-4	-5.30	2.90
-2	8	4	8.10	7.60
2	8	-4	-10.40	9.70
2	8	-4	8.30	6.90

2	8	-4	1.70	4.20
1	-8	-4	31.30	9.10
-1	-8	4	14.40	6.40
1	-8	-4	23.50	5.50
-1	-8	4	19.60	6.30
-1	-8	4	16.30	7.00
-1	8	4	12.90	11.50
1	8	-4	21.80	6.40
1	8	-4	27.70	7.60
-1	8	4	27.30	9.60
1	8	-4	17.60	4.10
1	8	-4	12.20	10.70
0	-8	-4	413.46	29.00
0	-8	4	459.95	23.80
0	-8	4	473.95	19.10
0	-8	-4	462.25	19.60
0	-8	-4	451.65	26.30
0	8	-4	414.66	29.90
0	8	-4	467.95	14.80
0	8	4	476.05	22.30
0	8	4	470.75	35.90
0	8	-4	457.05	26.50
-1	-8	-4	251.57	12.60
-1	-8	-4	275.07	24.80
1	-8	4	213.48	15.60
-1	-8	-4	223.48	18.70
1	8	4	251.67	15.40
-1	8	-4	252.17	19.00
1	8	4	263.47	25.60
-1	8	-4	255.27	8.40
-1	8	-4	218.38	21.10

−2	−8	−4	47.20	9.00
−2	−8	−4	39.70	11.40
−2	−8	−4	35.90	9.10
2	−8	4	18.10	6.60
−2	8	−4	25.50	10.80
−2	8	−4	41.10	10.30
2	8	4	37.90	13.00
2	8	4	19.10	7.40
2	8	4	53.19	9.30
−3	−8	−4	95.09	13.90
−3	−8	−4	119.59	17.00
3	−8	4	110.59	11.30
−3	−8	−4	107.49	18.40
3	8	4	128.49	17.10
−3	8	−4	111.69	16.10
3	8	4	88.79	11.70
3	8	4	103.69	19.10
−3	8	−4	118.49	20.90
4	−8	4	40.80	7.80
−4	−8	−4	35.50	9.70
−4	−8	−4	33.80	11.90
−4	−8	−4	52.89	15.80
−4	8	−4	63.69	18.40
−4	8	−4	41.90	9.00
−4	8	−4	29.00	10.80
−4	8	−4	43.40	5.00
4	8	4	41.00	9.00
4	8	4	15.00	10.70
−5	−8	−4	8.90	12.30
5	−8	4	5.00	6.00
−5	−8	−4	6.60	6.70

5	−8	4	15.50	6.20
−5	−8	−4	26.10	12.00
−5	8	−4	24.10	7.50
5	8	4	19.70	6.20
5	8	4	18.50	11.00
−5	8	−4	7.90	4.50
−5	8	−4	12.80	11.30
−5	8	−4	−6.80	7.80
−6	−8	−4	34.90	15.70
−6	−8	−4	29.50	9.60
−6	−8	−4	57.49	19.40
−6	8	−4	43.80	13.70
6	8	4	37.80	14.60
−6	8	−4	48.40	14.90
6	8	4	34.60	9.70
−6	8	−4	34.40	9.70
−7	−8	−4	21.80	16.70
−7	−8	−4	13.40	8.40
7	−8	4	−3.50	4.70
−7	−8	−4	2.70	14.10
7	8	4	5.30	8.20
−7	8	−4	−9.70	10.80
−7	8	−4	8.70	7.30
−7	8	−4	−13.60	13.90
−8	−8	−4	11.70	10.20
−8	−8	−4	48.00	21.40
−8	−8	−4	13.10	18.90
−8	8	−4	34.80	17.30
−8	8	−4	26.00	11.30
−8	8	−4	−9.40	15.00
8	8	4	6.10	9.50

−9	−8	−4	53.49	13.30
−9	−8	−4	47.50	23.70
−9	−8	−4	34.50	25.00
−9	8	−4	22.10	19.70
−9	8	−4	−6.00	16.50
−9	8	−4	2.80	13.10
9	8	4	0.30	10.50
−9	−9	4	−12.70	19.50
9	−9	−4	1.00	14.90
−9	−9	4	−6.60	24.70
9	9	−4	11.60	11.40
−9	9	4	13.10	20.40
−9	9	4	19.30	25.90
−9	9	4	−1.10	19.40
8	−9	−4	14.80	7.50
−8	−9	4	8.50	19.60
−8	−9	4	51.09	24.80
8	−9	−4	0.90	12.40
−8	9	4	45.10	20.00
8	9	−4	4.00	9.10
−8	9	4	2.80	21.50
−8	9	4	32.80	15.40
−7	−9	4	−2.80	15.40
7	−9	−4	3.10	6.10
7	−9	−4	17.30	10.50
−7	−9	4	−4.70	18.20
7	9	−4	3.00	9.90
−7	9	4	7.50	19.70
−7	9	4	3.30	11.80
−7	9	4	−21.30	13.70
−6	−9	4	−31.30	12.10

6	−9	−4	16.80	9.70
6	−9	−4	10.70	5.90
−6	−9	4	24.50	15.20
−6	−9	4	13.20	14.20
−6	9	4	−2.20	10.10
−6	9	4	−5.30	13.60
−5	−9	4	19.80	12.90
5	−9	−4	37.40	10.50
5	−9	−4	28.20	6.00
−5	−9	4	23.20	13.50
5	−9	−4	18.50	13.00
5	9	−4	33.30	8.80
−5	9	4	16.50	12.10
−4	−9	4	19.50	5.00
4	−9	−4	13.10	5.40
−4	−9	4	26.50	10.60
4	−9	−4	27.90	8.50
−4	−9	4	13.40	10.00
4	−9	−4	7.20	9.30
−4	−9	4	30.90	12.10
4	9	−4	18.10	6.90
−4	9	4	18.60	11.10
−4	9	4	4.10	13.70
4	9	−4	35.50	14.40
−3	−9	4	60.59	6.00
3	−9	−4	46.40	7.40
−3	−9	4	39.50	10.40
3	−9	−4	35.80	10.10
−3	−9	4	53.29	11.70
−3	−9	4	45.40	11.20
−3	9	4	37.90	15.80

3	9	-4	46.20	8.50
3	9	-4	51.19	8.30
-3	9	4	57.89	16.90
3	9	-4	47.60	13.00
-2	-9	4	161.38	16.70
-2	-9	4	161.08	15.80
2	-9	-4	110.29	15.50
2	-9	-4	178.68	11.40
-2	-9	4	201.98	29.80
2	9	-4	141.59	13.20
-2	9	4	131.19	21.00
2	9	-4	167.98	11.50
-2	9	4	159.98	25.90
-2	9	4	146.69	13.70
1	-9	-4	32.00	6.60
-1	-9	4	39.20	10.60
-1	-9	4	28.20	8.60
-1	-9	4	33.30	7.80
1	-9	-4	35.00	9.80
1	9	-4	48.20	12.10
1	9	-4	36.10	8.40
-1	9	4	35.20	9.10
1	9	-4	31.90	7.90
-1	9	4	31.20	14.00
0	-9	4	33.10	9.00
0	-9	-4	17.20	10.90
0	-9	4	15.10	6.00
0	-9	-4	14.50	8.30
0	9	-4	21.80	9.90
0	9	-4	34.00	4.70
0	9	-4	30.00	12.10

0	9	4	25.30	13.10
0	9	4	22.90	8.00
−1	−9	−4	306.17	26.80
−1	−9	−4	321.07	24.10
1	−9	4	329.97	18.70
1	−9	4	305.37	12.20
1	−9	4	298.97	19.60
1	9	4	304.27	19.00
1	9	4	339.07	31.30
−1	9	−4	304.77	24.50
−1	9	−4	335.67	27.00
−1	9	−4	320.27	21.90
−1	9	−4	326.67	11.30
−2	−9	−4	117.39	19.40
2	−9	4	111.69	14.00
−2	−9	−4	127.59	17.40
−2	−9	−4	153.68	13.20
−2	9	−4	152.88	18.20
2	9	4	115.29	13.40
2	9	4	138.79	24.10
3	−9	4	60.19	10.50
−3	−9	−4	66.39	13.10
−3	−9	−4	60.39	12.50
−3	−9	−4	64.29	15.20
3	9	4	117.69	14.30
−3	9	−4	94.99	15.20
−3	9	−4	68.69	5.00
−3	9	−4	97.39	20.50
3	9	4	99.79	20.10
−4	−9	−4	148.59	20.80
−4	−9	−4	133.89	18.30

-4	-9	-4	130.89	23.50
4	-9	4	123.49	15.60
-4	9	-4	111.79	21.50
-4	9	-4	123.69	8.40
-4	9	-4	115.59	20.60
4	9	4	136.59	23.70
4	9	4	98.99	13.00
-5	-9	-4	17.60	14.00
5	-9	4	20.90	7.30
-5	-9	-4	24.00	10.50
-5	-9	-4	35.50	15.40
-5	9	-4	10.30	10.80
-5	9	-4	15.90	5.70
-5	9	-4	10.00	14.40
5	9	4	28.10	14.50
-6	-9	-4	-5.90	13.20
6	-9	4	1.20	7.20
-6	-9	-4	8.40	15.00
-6	-9	-4	21.80	9.50
6	9	4	7.20	7.10
-6	9	-4	33.80	21.10
-6	9	-4	29.70	13.90
-6	9	-4	7.40	11.20
-7	-9	-4	14.10	17.10
-7	-9	-4	13.20	17.90
7	9	4	10.70	14.60
-7	9	-4	46.00	19.60
7	9	4	22.80	10.20
-7	9	-4	8.70	13.40
-8	-9	-4	26.60	20.30
-8	-9	-4	-12.90	10.30

-8	-9	-4	16.10	19.30
-8	9	-4	-1.80	20.90
-8	9	-4	30.20	17.50
8	9	4	14.80	9.10
-9	-9	-4	-23.70	11.50
-9	-9	-4	16.90	24.90
-9	-9	-4	9.20	22.50
9	9	4	1.90	10.00
-9	9	-4	13.70	9.50
-9	9	-4	47.70	26.30
-9	9	-4	23.80	19.10
-8	-10	4	24.40	20.70
8	-10	-4	1.70	14.40
-8	-10	4	4.60	23.90
-8	10	4	1.00	15.90
-8	10	4	16.30	17.80
8	10	-4	29.10	13.20
-7	-10	4	-29.10	16.70
7	-10	-4	11.40	12.60
-7	-10	4	-2.20	19.00
-7	10	4	-11.20	19.10
7	10	-4	15.20	9.80
-7	10	4	10.50	13.00
-7	10	4	-1.10	14.10
-6	-10	4	122.89	26.70
6	-10	-4	80.89	16.90
6	-10	-4	92.49	8.00
-6	-10	4	105.99	25.70
-6	10	4	124.69	31.30
6	10	-4	115.89	15.60
-6	10	4	167.38	28.00

-6	10	4	135.99	18.20
-5	-10	4	7.40	12.60
5	-10	-4	-6.10	9.20
5	-10	-4	25.60	11.80
-5	10	4	21.00	17.40
-5	10	4	1.30	11.90
5	10	-4	22.10	9.10
4	-10	-4	20.90	8.20
-4	-10	4	9.90	12.70
4	-10	-4	25.00	12.00
-4	-10	4	21.30	6.10
-4	-10	4	15.20	8.80
-4	-10	4	9.20	10.60
4	10	-4	49.50	16.10
-4	10	4	24.00	14.20
-4	10	4	10.00	15.50
4	10	-4	15.50	7.40
3	-10	-4	27.20	11.10
-3	-10	4	39.90	10.90
-3	-10	4	26.50	10.70
-3	-10	4	44.20	12.10
3	-10	-4	34.40	6.00
-3	10	4	29.60	16.80
3	10	-4	34.00	12.80
-3	10	4	44.60	14.20
3	10	-4	38.30	8.30
-2	-10	4	133.69	17.30
-2	-10	4	95.29	16.40
2	-10	-4	86.79	16.10
-2	-10	4	136.29	16.70
-2	10	4	100.19	23.90

2	10	−4	102.09	11.10
−2	10	4	115.99	14.10
2	10	−4	103.49	16.10
1	−10	−4	−5.90	5.50
−1	−10	4	3.40	5.60
1	−10	−4	−1.10	11.00
−1	−10	4	−0.50	6.70
−1	10	4	8.90	14.10
1	10	−4	−15.20	6.60
1	10	−4	−1.50	3.90
1	10	−4	−21.40	9.60
−1	10	4	−5.80	5.50
0	−10	4	4.50	6.90
0	−10	4	3.40	6.20
0	−10	−4	13.10	10.10
0	−10	−4	3.30	6.90
0	10	4	3.00	10.10
0	10	−4	0.70	3.30
0	10	4	6.50	6.00
0	10	−4	13.90	9.40
0	10	−4	−12.10	9.00
1	−10	4	7.70	5.30
1	−10	4	1.30	5.90
−1	−10	−4	3.10	6.10
1	−10	4	−8.10	4.50
−1	−10	−4	−0.30	9.60
−1	10	−4	4.10	10.50
−1	10	−4	−3.40	8.20
−1	10	−4	−3.00	10.00
1	10	4	4.70	6.70
1	10	4	1.10	13.00

-1	10	-4	7.60	3.30
-2	-10	-4	82.19	10.90
-2	-10	-4	48.20	12.20
2	-10	4	80.39	12.70
-2	-10	-4	75.29	17.40
2	10	4	71.79	19.60
-2	10	-4	77.89	5.90
-2	10	-4	93.89	6.20
-2	10	-4	67.59	12.80
-2	10	-4	105.39	21.30
2	10	4	85.19	13.00
3	-10	4	-1.90	6.50
-3	-10	-4	-5.90	11.90
-3	-10	-4	-8.70	5.70
3	10	4	13.30	9.30
3	10	4	-1.90	9.70
-3	10	-4	-8.00	3.50
-3	10	-4	0.00	10.80
3	10	4	-0.90	6.90
-3	10	-4	14.90	9.10
4	-10	4	4.80	7.20
-4	-10	-4	-1.70	7.10
-4	-10	-4	1.60	10.50
-4	-10	-4	-5.00	13.10
-4	10	-4	7.90	13.80
4	10	4	-11.70	5.90
-4	10	-4	5.70	4.70
-4	10	-4	25.80	15.90
4	10	4	-7.60	9.00
5	-10	4	13.00	8.10
-5	-10	-4	1.90	9.30

-5	-10	-4	4.50	17.10
-5	-10	-4	33.00	14.20
5	10	4	8.40	12.30
-5	10	-4	22.00	14.80
5	10	4	2.30	7.60
-5	10	-4	19.80	10.20
-6	-10	-4	122.09	22.90
-6	-10	-4	92.39	17.30
-6	10	-4	143.79	22.30
-6	10	-4	49.90	22.00
6	10	4	64.99	12.50
6	10	4	139.59	26.00
-7	-10	-4	29.90	12.90
-7	-10	-4	29.70	22.20
7	-10	4	-1.90	7.90
-7	-10	-4	30.10	18.40
-7	10	-4	-3.70	19.60
-7	10	-4	37.30	15.20
7	10	4	21.60	17.30
7	10	4	13.30	8.80
-8	-10	-4	30.00	22.50
-8	-10	-4	-13.30	11.00
-8	-10	-4	-11.80	19.00
-8	10	-4	-22.00	22.30
-8	10	-4	-33.90	15.40
8	10	4	-10.10	17.70
8	-11	-4	7.60	15.10
-8	-11	4	-14.10	26.10
-8	-11	4	-34.30	22.00
8	11	-4	4.10	12.10
-8	11	4	-18.80	20.30

-8	11	4	1.40	20.20
-7	-11	4	12.20	23.40
7	-11	-4	8.60	14.10
-7	-11	4	8.60	23.00
-7	11	4	-17.50	12.90
-7	11	4	6.70	17.40
-7	11	4	-10.10	21.10
7	11	-4	11.00	9.70
-6	-11	4	-17.50	16.40
6	-11	-4	-5.60	12.00
6	11	-4	-3.50	8.30
-6	11	4	3.00	14.50
-6	11	4	1.80	18.50
5	-11	-4	39.30	14.00
-5	-11	4	27.60	15.20
-5	-11	4	48.00	14.20
-5	11	4	10.80	14.70
-5	11	4	34.30	20.30
-4	-11	4	15.60	8.70
4	-11	-4	12.00	9.90
-4	-11	4	16.30	5.70
-4	-11	4	21.40	13.70
4	11	-4	10.80	7.70
4	11	-4	-6.60	7.40
4	11	-4	28.50	16.50
-4	11	4	12.50	12.10
-4	11	4	0.50	15.50
3	-11	-4	10.30	8.80
-3	-11	4	18.50	9.30
-3	-11	4	17.40	12.20
3	11	-4	17.20	7.40

-3	11	4	15.20	8.10
3	11	-4	12.70	8.20
-3	11	4	16.90	13.70
3	11	-4	5.70	13.00
2	-11	-4	223.98	28.80
-2	-11	4	227.38	34.60
2	-11	-4	195.28	24.30
-2	-11	4	210.48	22.80
-2	11	4	204.88	17.70
2	11	-4	197.68	14.10
2	11	-4	178.98	17.20
-2	11	4	195.38	32.30
1	-11	-4	-9.70	10.90
1	-11	-4	2.90	8.10
-1	-11	4	9.40	8.10
-1	-11	4	10.40	8.00
-1	-11	4	10.90	12.10
-1	11	4	-20.70	12.50
1	11	-4	8.60	8.50
1	11	-4	-3.50	4.40
1	11	-4	23.60	12.10
-1	11	4	1.70	6.50
0	-11	4	2.90	6.90
0	-11	4	0.00	7.60
0	-11	-4	2.10	8.60
0	-11	-4	-10.00	11.00
0	11	-4	3.90	4.50
0	11	-4	4.60	11.00
0	11	4	7.50	7.20
0	11	-4	-12.30	9.80
0	11	-4	-7.50	7.80

0	11	4	-5.50	12.90
1	-11	4	-7.10	5.90
-1	-11	-4	0.20	11.30
1	-11	4	-1.30	5.20
-1	-11	-4	14.20	10.30
1	11	4	-6.20	6.70
-1	11	-4	6.60	7.50
-1	11	-4	6.30	3.90
-1	11	-4	21.80	11.40
-1	11	-4	12.20	12.40
1	11	4	18.10	13.80
-2	-11	-4	139.09	24.60
-2	-11	-4	153.68	24.40
2	-11	4	118.39	16.80
-2	11	-4	110.69	23.10
2	11	4	131.09	16.20
-2	11	-4	148.49	8.60
-2	11	-4	123.89	16.20
-2	11	-4	155.88	8.90
-2	11	-4	110.69	18.80
2	11	4	147.49	28.00
-3	-11	-4	20.30	16.40
3	-11	4	9.70	6.90
-3	-11	-4	14.70	10.30
-3	-11	-4	37.40	10.20
3	11	4	10.70	9.20
-3	11	-4	10.10	8.70
-3	11	-4	9.10	4.70
3	11	4	11.70	7.00
3	11	4	1.70	12.90
-3	11	-4	3.50	15.00

-4	-11	-4	8.30	9.10
-4	-11	-4	-9.40	14.60
4	-11	4	3.50	7.40
-4	-11	-4	17.10	11.80
-4	11	-4	17.70	16.10
-4	11	-4	17.70	6.20
-4	11	-4	-12.20	9.50
4	11	4	-0.60	7.70
5	-11	4	25.30	9.70
-5	-11	-4	8.30	17.40
-5	-11	-4	26.90	16.20
-5	-11	-4	6.70	10.10
-5	11	-4	21.70	10.60
-5	11	-4	4.30	18.30
5	11	4	19.90	14.60
5	11	4	32.80	10.80
-6	-11	-4	-4.00	10.50
-6	-11	-4	-8.10	17.80
-6	-11	-4	10.50	20.40
6	-11	4	-7.70	9.00
6	11	4	33.90	16.00
6	11	4	-0.80	7.70
-6	11	-4	-4.30	11.50
-6	11	-4	-18.20	17.50
7	-11	4	7.50	10.50
-7	-11	-4	20.00	22.80
-7	-11	-4	21.70	11.80
7	11	4	14.00	10.70
-7	11	-4	-1.80	15.60
7	11	4	-12.80	18.00
-7	11	-4	3.40	20.00

-8	-11	-4	20.50	25.10
-8	-11	-4	-8.00	13.50
-8	-11	-4	-14.10	24.60
-8	11	-4	42.50	26.00
-8	11	-4	15.00	17.90
8	11	4	-11.80	11.00
-7	-12	4	-31.20	23.70
7	-12	-4	-3.50	16.00
-7	12	4	28.80	18.40
7	12	-4	-7.00	9.90
-7	12	4	29.90	24.70
-6	-12	4	29.60	21.30
6	-12	-4	6.00	13.60
6	12	-4	14.60	10.60
-6	12	4	15.90	24.70
-6	12	4	-10.60	16.30
5	-12	-4	2.90	11.90
-5	-12	4	-0.20	10.60
-5	-12	4	32.60	17.50
-5	12	4	8.00	15.20
5	12	-4	12.70	9.10
-5	12	4	13.30	20.00
-4	-12	4	7.80	10.60
4	-12	-4	-3.40	11.30
-4	-12	4	14.30	15.10
-4	12	4	6.00	8.70
4	12	-4	3.40	7.80
4	12	-4	-10.50	9.00
-4	12	4	24.60	21.70
3	-12	-4	-12.90	10.90
-3	-12	4	-5.80	11.80

-3	-12	4	-8.40	7.90
-3	12	4	1.10	17.70
3	12	-4	7.20	8.40
-3	12	4	-2.70	7.60
3	12	-4	2.90	6.50
-2	-12	4	-43.30	17.50
2	-12	-4	0.50	9.90
2	-12	-4	22.50	14.20
-2	-12	4	-0.50	7.90
-2	12	4	23.30	18.90
2	12	-4	-23.20	8.20
-2	12	4	-4.90	8.00
2	12	-4	6.30	6.50
-1	-12	4	18.80	9.50
1	-12	-4	50.99	14.30
-1	-12	4	53.79	13.80
1	-12	-4	50.79	17.60
1	12	-4	45.60	14.60
1	12	-4	47.20	13.60
-1	12	4	48.70	11.70
1	12	-4	49.60	13.50
-1	12	4	32.40	19.40
0	-12	-4	50.29	15.30
0	-12	4	38.90	10.80
0	-12	4	16.20	10.80
0	-12	-4	28.40	16.60
0	12	4	41.20	20.80
0	12	4	14.90	9.10
0	12	-4	9.40	12.20
0	12	-4	25.40	6.60
0	12	-4	61.39	16.40

0	12	-4	49.80	11.80
-1	-12	-4	31.60	11.80
1	-12	4	33.80	7.70
-1	-12	-4	63.39	19.10
1	-12	4	8.60	9.20
-1	12	-4	-2.30	14.30
-1	12	-4	11.20	9.70
1	12	4	22.90	9.10
-1	12	-4	17.00	12.50
-1	12	-4	23.70	6.20
-2	-12	-4	-5.10	15.50
-2	-12	-4	42.30	14.90
2	-12	4	4.40	7.90
-2	12	-4	34.40	9.40
-2	12	-4	16.70	5.00
2	12	4	27.10	9.70
-2	12	-4	-7.20	16.20
-2	12	-4	-7.20	12.40
-2	12	-4	26.00	6.30
2	12	4	24.90	20.30
-3	-12	-4	-41.40	14.60
-3	-12	-4	10.00	11.50
3	-12	4	12.10	9.70
-3	-12	-4	8.50	8.40
3	12	4	14.20	9.00
3	12	4	8.60	16.40
-3	12	-4	6.90	5.10
-3	12	-4	7.30	16.60
-3	12	-4	29.90	10.40
-4	-12	-4	19.80	13.80
-4	-12	-4	7.00	18.70

-4	-12	-4	21.00	12.40
4	-12	4	-1.10	9.60
4	12	4	12.20	9.20
-4	12	-4	-19.80	18.60
-4	12	-4	12.10	9.90
4	12	4	15.30	16.50
5	-12	4	-15.00	9.70
-5	-12	-4	-14.90	20.80
-5	-12	-4	-17.10	15.90
-5	-12	-4	2.00	12.60
5	12	4	8.10	10.10
-5	12	-4	1.50	10.50
-5	12	-4	15.80	19.60
5	12	4	21.20	19.30
-6	-12	-4	-24.40	10.80
-6	-12	-4	12.70	20.70
-6	-12	-4	8.40	24.60
6	-12	4	-9.20	11.00
-6	12	-4	-31.00	20.40
-6	12	-4	8.50	13.10
6	12	4	36.70	19.20
6	12	4	5.60	9.40
-7	-12	-4	4.10	25.80
-7	-12	-4	18.20	28.20
-7	-12	-4	17.50	14.70
-7	12	-4	-13.50	26.60
7	12	4	6.30	20.20
-7	12	-4	-8.60	15.90
7	12	4	4.10	12.60
-6	-13	4	40.90	23.30
6	-13	-4	-16.00	15.60

-6	13	4	-5.10	25.00
6	13	-4	18.00	11.40
-6	13	4	-35.70	15.60
-5	-13	4	-9.50	11.60
5	-13	-4	14.70	14.90
-5	-13	4	7.30	21.00
5	13	-4	5.10	10.00
-5	13	4	18.40	22.20
-5	13	4	-5.30	10.20
4	-13	-4	-7.60	12.70
-4	-13	4	2.80	10.70
-4	-13	4	17.70	18.00
-4	13	4	5.30	11.20
-4	13	4	-2.60	18.90
4	13	-4	8.20	9.00
4	13	-4	-22.30	10.70
-3	-13	4	13.30	15.50
-3	-13	4	10.70	12.10
3	-13	-4	27.50	13.60
3	-13	-4	28.90	19.70
3	13	-4	6.30	8.30
-3	13	4	11.60	10.60
-3	13	4	-12.10	20.00
3	13	-4	7.60	11.00
2	-13	-4	21.60	17.70
-2	-13	4	14.90	11.00
2	-13	-4	26.80	14.00
-2	13	4	14.00	9.80
2	13	-4	13.40	8.10
-2	13	4	10.40	16.80
-1	-13	4	16.50	11.30

1	-13	-4	19.30	16.50
1	-13	-4	3.00	10.60
-1	13	4	19.50	10.00
1	13	-4	10.70	12.90
1	13	-4	15.50	6.80
-1	13	4	11.50	19.10
0	-13	-4	-2.90	17.40
0	-13	-4	8.10	11.60
0	-13	4	12.20	10.30
0	13	-4	13.10	10.20
0	13	-4	-5.60	13.20
0	13	4	0.40	7.60
0	13	-4	3.60	6.10
0	13	4	-17.90	15.00
1	-13	4	-7.70	9.10
-1	-13	-4	-6.40	16.10
-1	-13	-4	17.40	12.30
1	13	4	8.70	15.50
-1	13	-4	19.00	16.50
-1	13	-4	-1.00	5.10
1	13	4	-9.40	8.40
2	-13	4	-8.90	9.00
-2	-13	-4	6.70	11.30
-2	-13	-4	-9.40	18.30
-2	13	-4	9.70	17.50
2	13	4	3.90	17.60
2	13	4	6.00	9.60
3	-13	4	-5.10	9.40
-3	-13	-4	-13.30	18.20
-3	-13	-4	-4.20	12.80
-3	13	-4	15.40	6.00

3	13	4	-8.00	8.60
3	13	4	-11.50	17.20
-3	13	-4	-19.00	21.90
-3	13	-4	3.90	9.00
-4	-13	-4	14.40	13.20
-4	-13	-4	-9.40	19.70
4	13	4	7.30	20.10
4	13	4	7.30	10.30
-4	13	-4	18.40	21.00
-4	13	-4	-5.90	10.10
5	-13	4	-27.30	11.30
-5	-13	-4	28.70	13.90
-5	-13	-4	-4.60	24.00
5	13	4	12.60	19.90
5	13	4	-19.70	10.40
-5	13	-4	-0.80	12.30
-5	13	-4	-34.40	19.70
-6	-13	-4	6.60	25.50
6	-13	4	-4.30	13.60
6	13	4	8.20	18.50
-6	13	-4	-13.60	23.80
6	13	4	-4.30	10.00
-5	-14	4	-6.80	13.70
5	-14	-4	36.80	17.90
-5	14	4	-8.00	24.80
-5	14	4	-32.30	11.60
5	14	-4	2.90	11.50
-4	-14	4	-20.40	12.80
4	-14	-4	9.10	14.10
-4	14	4	-4.30	26.30
-4	14	4	2.00	11.20

4	14	-4	12.80	10.90
-3	-14	4	-13.80	12.00
3	-14	-4	-11.50	13.50
3	14	-4	5.50	13.60
3	14	-4	9.00	10.40
-3	14	4	3.90	11.60
-3	14	4	0.20	23.30
2	-14	-4	19.90	15.90
-2	-14	4	14.00	12.10
2	-14	-4	-38.00	23.10
2	14	-4	15.10	13.40
-2	14	4	-28.20	22.80
2	14	-4	1.20	8.70
-2	14	4	10.60	10.80
-1	-14	4	9.60	11.10
1	-14	-4	-3.00	19.40
1	-14	-4	1.00	14.00
1	14	-4	5.10	7.20
-1	14	4	14.50	11.20
1	14	-4	0.20	13.50
1	14	-4	-12.80	11.30
-1	14	4	-5.40	20.10
0	-14	-4	-9.00	13.00
0	-14	4	1.90	11.10
0	-14	-4	-0.40	21.90
0	14	4	5.90	10.90
0	14	-4	-12.30	15.80
0	14	-4	-2.20	6.90
0	14	4	-2.80	21.10
-1	-14	-4	-7.10	10.40
-1	-14	-4	22.40	19.90

1	-14	4	4.60	11.80
-1	14	-4	-29.20	18.20
1	14	4	15.40	10.00
-1	14	-4	2.50	6.80
1	14	4	-12.70	19.20
2	-14	4	15.70	11.30
-2	-14	-4	-12.00	21.10
-2	-14	-4	14.00	15.90
2	14	4	8.90	9.80
-2	14	-4	-10.60	19.10
2	14	4	20.30	20.30
-3	-14	-4	26.70	15.60
-3	-14	-4	5.20	24.30
3	-14	4	35.80	13.40
3	14	4	37.70	12.60
3	14	4	36.40	23.00
-3	14	-4	75.59	26.30
-4	-14	-4	-18.50	12.90
-4	-14	-4	-17.20	26.30
4	-14	4	-17.40	11.80
4	14	4	-4.00	10.60
-4	14	-4	-29.60	24.90
4	14	4	-32.20	19.10
-5	-14	-4	-22.40	29.20
-5	14	-4	7.80	27.40
-3	-15	4	-4.70	12.20
3	-15	-4	-5.00	17.10
3	15	-4	8.80	10.90
-3	15	4	5.50	12.40
-3	15	4	-12.50	24.90
-2	-15	4	-3.80	13.10

2	-15	-4	18.40	18.60
-2	15	4	90.19	33.80
2	15	-4	20.10	11.80
-2	15	4	26.90	13.60
-1	-15	4	19.40	23.80
1	-15	-4	-12.50	16.10
1	15	-4	54.69	19.40
-1	15	4	-12.60	21.20
1	15	-4	0.10	9.70
-1	15	4	22.90	13.90
0	-15	-4	-3.30	13.20
0	-15	4	26.70	13.40
0	15	4	6.90	12.10
0	15	4	15.70	22.00
0	15	-4	-0.70	8.30
0	15	-4	18.90	20.10
-1	-15	-4	15.80	15.50
1	-15	4	12.70	12.20
-1	-15	-4	26.40	37.20
1	15	4	-9.50	19.20
1	15	4	13.40	13.00
-1	15	-4	58.59	24.00
-2	-15	-4	-45.50	22.40
2	-15	4	-9.00	13.30
-2	-15	-4	12.10	15.70
2	15	4	18.30	12.50
2	15	4	-20.40	22.10
-2	15	-4	36.50	26.50
-3	-15	-4	-37.60	29.60
3	-15	4	-30.10	12.60
-3	-15	-4	-8.50	13.80

-3	15	-4	7.40	26.00
3	15	4	48.70	25.60
3	15	4	-7.60	11.00
-11	0	5	59.79	27.30
11	0	-5	23.30	12.70
-11	0	5	66.49	25.70
11	0	-5	0.60	15.50
-11	0	5	41.00	24.80
-10	0	5	8.60	15.30
-10	0	5	-3.90	15.80
-10	0	5	11.40	17.10
10	0	-5	-7.60	12.00
10	0	-5	14.10	10.20
-9	0	5	49.00	16.80
-9	0	5	30.80	18.80
9	0	-5	41.80	11.80
-9	0	5	80.59	20.80
8	0	-5	9.00	6.30
-8	0	5	10.50	11.50
-8	0	5	-5.80	10.70
-8	0	5	-2.20	11.60
7	0	-5	-4.20	4.60
-7	0	5	14.70	10.80
-7	0	5	-1.80	8.20
-7	0	5	6.40	10.00
-6	0	5	0.20	8.90
-6	0	5	5.90	8.50
6	0	-5	3.80	4.10
-6	0	5	-4.60	8.10
5	0	-5	14.70	5.50
-5	0	5	25.50	9.30

−5	0	5	17.50	9.60
−5	0	5	18.40	8.40
−4	0	5	−1.20	5.50
4	0	−5	−0.70	7.30
−4	0	5	4.30	6.50
4	0	−5	−2.20	3.40
−3	0	5	1759.92	56.69
−3	0	5	1774.92	48.10
−3	0	5	1850.71	64.09
3	0	−5	1819.52	63.69
3	0	−5	1929.71	49.60
−3	0	5	1874.61	61.39
3	0	−5	1806.42	67.79
3	0	−5	1835.62	55.39
2	0	−5	−1.20	4.30
−2	0	5	−0.40	2.40
2	0	−5	1.00	6.50
−2	0	5	8.80	5.00
2	0	−5	2.70	5.10
−2	0	5	−1.50	3.30
1	0	−5	3293.97	87.49
1	0	−5	3188.08	82.59
1	0	−5	3311.77	76.39
−1	0	5	3149.98	79.49
−1	0	5	3227.48	74.99
−1	0	5	3282.27	88.39
0	0	−5	−5.00	3.50
0	0	5	0.40	1.70
0	0	5	0.50	2.20
0	0	5	2.20	2.40
0	0	−5	3.10	3.10

0	0	5	-0.90	3.40
0	0	-5	3.50	2.20
1	0	5	480.15	15.80
1	0	5	526.75	24.20
1	0	5	536.35	22.20
-1	0	-5	479.25	18.00
-1	0	-5	500.35	19.50
-1	0	-5	529.15	26.50
1	0	5	509.25	17.40
-1	0	-5	551.44	21.90
-1	0	-5	531.35	25.00
-1	0	-5	507.65	16.40
2	0	5	2.40	2.80
-2	0	-5	0.60	3.80
-2	0	-5	3.20	4.20
-2	0	-5	6.30	4.60
-2	0	-5	-1.40	4.20
2	0	5	0.40	3.80
-2	0	-5	-0.20	2.30
-2	0	-5	1.80	2.70
-2	0	-5	-0.30	2.20
2	0	5	-0.50	1.50
-3	0	-5	1977.00	65.59
-3	0	-5	2056.89	60.99
3	0	5	2024.60	50.29
-3	0	-5	2025.60	60.99
-3	0	-5	2062.29	59.79
3	0	5	2001.80	58.49
-3	0	-5	1949.10	52.49
3	0	5	1973.40	64.09
3	0	5	2079.39	48.70

-3	0	-5	2027.90	47.80
-4	0	-5	0.80	5.00
-4	0	-5	1.50	1.40
-4	0	-5	2.40	5.90
-4	0	-5	-0.20	3.00
4	0	5	2.30	2.20
4	0	5	5.50	3.30
-4	0	-5	0.80	3.00
-5	0	-5	236.88	19.40
-5	0	-5	213.58	22.40
5	0	5	240.88	12.10
5	0	5	244.88	12.90
-6	0	-5	7.50	6.50
6	0	5	1.80	3.60
6	0	5	5.90	5.40
-6	0	-5	-1.30	7.30
7	0	5	0.60	4.50
-7	0	-5	-5.80	7.90
-7	0	-5	-2.40	9.80
8	0	5	-3.10	5.70
-8	0	-5	-1.60	9.60
-8	0	-5	2.50	11.20
9	0	5	22.80	8.40
-9	0	-5	34.40	15.40
-9	0	-5	18.00	14.30
-10	0	-5	15.80	18.10
-10	0	-5	7.90	14.70
10	0	5	-3.20	8.40
-11	0	-5	-23.70	20.00
-11	0	-5	-22.70	18.40
11	0	5	18.00	11.30

-11	-1	5	-10.40	21.60
-11	-1	5	5.50	20.20
11	-1	-5	-8.80	11.00
-11	-1	5	-48.10	18.50
11	-1	-5	-20.60	14.70
11	1	-5	11.90	15.40
11	1	-5	9.50	12.30
-11	1	5	34.60	22.00
-11	1	5	16.00	18.70
-10	-1	5	8.40	16.90
10	-1	-5	0.90	9.60
-10	-1	5	-8.60	14.80
-10	-1	5	18.30	19.70
10	-1	-5	5.90	12.20
-10	1	5	0.60	15.60
-10	1	5	10.50	18.50
10	1	-5	-8.40	7.80
-10	1	5	-18.90	16.40
-9	-1	5	-11.20	12.80
-9	-1	5	2.60	13.60
9	-1	-5	-1.40	10.10
-9	-1	5	12.60	14.40
9	-1	-5	22.50	9.00
9	1	-5	5.20	7.40
-9	1	5	16.60	18.30
-9	1	5	-0.90	16.00
-9	1	5	1.80	13.50
8	-1	-5	-8.90	7.60
-8	-1	5	-0.20	10.00
-8	-1	5	-10.40	9.80
-8	-1	5	18.40	14.70

8	−1	−5	1.50	6.80
−8	1	5	10.70	11.60
8	1	−5	−7.00	5.60
−8	1	5	−7.80	12.40
−8	1	5	3.20	11.60
−7	−1	5	283.97	33.30
7	−1	−5	283.67	21.60
−7	−1	5	351.36	26.20
−7	−1	5	371.56	33.70
7	−1	−5	327.17	19.70
−7	1	5	329.87	29.30
−7	1	5	247.88	30.30
−7	1	5	316.47	31.10
7	1	−5	300.47	19.00
−6	−1	5	38.40	11.30
6	−1	−5	40.50	8.50
−6	−1	5	38.10	13.20
−6	−1	5	11.40	11.20
−6	1	5	36.60	13.60
−6	1	5	54.79	13.50
−6	1	5	49.40	12.20
6	1	−5	39.60	8.30
−5	−1	5	444.36	25.70
−5	−1	5	392.26	30.20
5	−1	−5	424.16	20.30
−5	−1	5	416.36	32.20
5	1	−5	454.05	20.50
−5	1	5	462.25	30.50
−5	1	5	431.16	29.80
−5	1	5	451.75	29.70
−4	−1	5	69.29	11.10

4	−1	−5	56.19	15.70
−4	−1	5	56.49	13.90
−4	−1	5	76.79	14.30
4	−1	−5	63.69	8.60
−4	1	5	62.69	13.50
−4	1	5	55.99	13.90
4	1	−5	58.09	8.10
4	1	−5	48.70	11.90
−4	1	5	68.39	12.80
3	−1	−5	468.35	26.30
3	−1	−5	497.05	28.70
−3	−1	5	460.35	29.30
3	−1	−5	464.95	27.60
−3	−1	5	484.45	22.60
−3	−1	5	477.25	18.20
3	−1	−5	487.85	19.80
3	1	−5	468.55	26.90
−3	1	5	478.25	27.60
3	1	−5	499.45	29.90
−3	1	5	520.95	19.90
3	1	−5	518.95	19.40
−3	1	5	457.65	27.10
−3	1	5	478.45	26.40
2	−1	−5	−8.00	5.50
2	−1	−5	1.60	5.20
−2	−1	5	3.50	2.80
−2	−1	5	0.60	3.60
2	−1	−5	−6.30	5.00
2	−1	−5	−1.10	2.50
2	−1	−5	−1.40	2.90
−2	−1	5	−0.10	3.00

2	1	−5	−1.10	3.60
−2	1	5	−6.20	4.00
−2	1	5	2.70	2.80
−2	1	5	−4.30	4.40
2	1	−5	−1.10	5.10
2	1	−5	1.70	7.00
1	−1	−5	455.75	25.40
−1	−1	5	452.25	16.50
−1	−1	5	431.76	15.00
−1	−1	5	458.25	23.10
1	−1	−5	440.56	23.20
1	−1	−5	449.46	17.80
1	−1	−5	479.05	21.40
−1	1	5	447.06	21.90
1	1	−5	466.35	16.30
1	1	−5	465.65	23.10
1	1	−5	462.35	18.80
−1	1	5	418.16	16.70
−1	1	5	449.36	25.10
0	−1	−5	236.98	12.50
0	−1	5	254.77	9.80
0	−1	−5	227.18	17.00
0	−1	5	263.07	16.90
0	−1	5	254.37	9.60
0	−1	−5	237.18	14.20
0	−1	−5	257.57	15.40
0	1	5	232.08	11.10
0	1	−5	242.58	11.00
0	1	−5	252.77	12.20
0	1	−5	236.48	15.90
0	1	5	231.08	14.60

0	1	5	236.28	11.80
0	1	5	240.58	17.60
−1	−1	−5	106.19	7.80
−1	−1	−5	96.39	6.80
1	−1	5	98.79	10.00
−1	−1	−5	101.29	11.10
−1	−1	−5	87.09	8.60
−1	−1	−5	89.99	12.10
−1	−1	−5	99.39	9.60
1	−1	5	109.29	6.10
1	−1	5	107.99	8.90
1	−1	5	86.99	5.00
1	1	5	93.59	9.90
1	1	5	95.29	7.70
1	1	5	116.59	14.70
1	1	5	85.39	6.50
−1	1	−5	100.29	5.90
−1	1	−5	94.49	6.90
−1	1	−5	97.79	8.60
−2	−1	−5	158.28	9.90
2	−1	5	140.09	7.60
2	−1	5	133.09	6.30
−2	−1	−5	139.39	12.40
−2	−1	−5	146.79	14.30
−2	−1	−5	171.48	13.90
−2	−1	−5	145.49	8.20
−2	−1	−5	128.89	13.00
−2	1	−5	173.88	16.00
2	1	5	138.59	8.10
−2	1	−5	152.88	11.70
−2	1	−5	151.08	8.20

-2	1	-5	152.18	8.60
-2	1	-5	162.88	7.70
2	1	5	150.48	12.90
2	1	5	149.59	9.20
2	1	5	136.49	12.90
2	1	5	148.99	10.00
-3	-1	-5	163.68	15.10
-3	-1	-5	141.59	9.00
3	-1	5	144.99	17.50
-3	-1	-5	122.69	13.00
-3	-1	-5	140.19	14.90
3	-1	5	132.19	11.80
-3	-1	-5	134.49	8.20
-3	-1	-5	148.59	14.50
3	-1	5	145.09	8.20
3	-1	5	136.79	6.80
-3	-1	-5	145.39	9.60
-3	1	-5	129.19	6.60
-3	1	-5	159.68	12.60
3	1	5	150.68	14.30
-3	1	-5	140.09	12.80
-3	1	-5	126.59	14.10
3	1	5	133.49	9.30
3	1	5	131.69	8.30
3	1	5	129.49	14.10
-3	1	-5	138.59	7.90
-3	1	-5	114.69	11.70
-3	1	-5	142.39	8.50
3	1	5	161.28	14.40
3	1	5	153.88	9.70
-4	-1	-5	21.20	8.00

−4	−1	−5	22.00	5.30
4	−1	5	33.90	4.60
−4	−1	−5	14.90	6.90
4	−1	5	17.70	4.30
−4	−1	−5	20.00	4.00
−4	1	−5	18.00	6.50
−4	1	−5	29.90	5.10
−4	1	−5	21.80	4.30
4	1	5	25.70	5.10
4	1	5	27.40	5.20
−4	1	−5	14.50	7.30
4	1	5	34.00	5.40
−5	−1	−5	66.49	12.80
5	−1	5	61.99	7.00
−5	−1	−5	60.39	8.70
5	−1	5	62.39	6.90
−5	−1	−5	66.99	14.60
−5	1	−5	57.09	13.50
5	1	5	83.99	7.30
5	1	5	54.49	7.60
5	1	5	66.79	8.30
−5	1	−5	43.80	9.90
6	−1	5	61.39	8.40
6	−1	5	58.49	7.10
−6	−1	−5	52.39	14.00
−6	−1	−5	76.19	15.20
6	1	5	61.79	8.20
−6	1	−5	51.09	12.50
6	1	5	53.89	6.30
−6	1	−5	84.09	18.10
−7	−1	−5	88.69	20.00

7	-1	5	107.49	11.20
-7	-1	-5	108.49	19.10
7	1	5	87.49	7.50
-7	1	-5	107.89	17.70
-7	1	-5	85.09	18.40
7	1	5	93.49	11.40
-8	-1	-5	6.00	11.10
8	-1	5	12.60	6.40
-8	-1	-5	-0.90	19.50
8	1	5	15.10	7.00
-8	1	-5	-2.30	10.20
-8	1	-5	9.10	13.30
9	-1	5	6.40	7.10
-9	-1	-5	8.10	14.60
-9	-1	-5	-13.10	14.30
-9	1	-5	-9.70	11.30
9	1	5	-1.70	6.80
-9	1	-5	-2.20	13.60
-10	-1	-5	-9.10	16.60
10	-1	5	17.60	8.60
-10	-1	-5	-5.40	14.90
10	1	5	9.80	9.20
-10	1	-5	-9.40	15.20
-10	1	-5	19.70	17.60
-11	-1	-5	-30.20	20.60
11	-1	5	12.90	11.30
-11	-1	-5	-24.10	17.00
11	1	5	4.60	10.20
-11	1	-5	-0.20	19.20
-11	1	-5	29.80	19.50
-11	-2	5	-11.60	19.20

−11	−2	5	33.60	22.60
11	−2	−5	36.00	17.30
11	−2	−5	−2.00	11.00
−11	−2	5	23.30	22.40
−11	2	5	23.50	22.00
11	2	−5	21.80	11.90
−11	2	5	38.90	23.40
−10	−2	5	12.20	19.00
10	−2	−5	−7.80	9.10
10	−2	−5	−18.90	11.80
−10	−2	5	19.90	19.20
−10	2	5	9.90	18.50
−10	2	5	36.00	17.50
10	2	−5	16.70	10.30
−10	2	5	−9.90	18.30
−9	−2	5	23.50	15.10
9	−2	−5	2.00	7.60
−9	−2	5	16.50	17.90
9	−2	−5	−11.20	10.00
−9	−2	5	22.30	15.90
−9	2	5	−3.60	14.00
9	2	−5	0.70	7.70
−9	2	5	−1.30	12.70
−9	2	5	−6.80	14.80
8	−2	−5	−8.90	6.70
−8	−2	5	10.00	9.90
8	−2	−5	5.70	9.00
−8	−2	5	13.70	13.80
−8	−2	5	−3.40	12.00
−8	2	5	6.30	12.80
−8	2	5	3.90	18.10

−8	2	5	8.50	14.20
−7	−2	5	129.79	23.00
−7	−2	5	134.19	24.70
7	−2	−5	116.39	14.30
7	−2	−5	158.28	19.00
−7	−2	5	129.59	19.50
−7	2	5	128.79	24.30
7	2	−5	136.39	12.70
−7	2	5	160.18	26.20
−7	2	5	138.09	24.40
−6	−2	5	25.10	9.40
6	−2	−5	10.70	5.90
−6	−2	5	11.40	10.30
6	−2	−5	19.60	8.30
−6	−2	5	15.70	10.50
−6	2	5	10.10	10.50
−6	2	5	17.10	12.00
−6	2	5	5.00	10.80
6	2	−5	3.60	4.80
5	−2	−5	9.10	4.60
−5	−2	5	8.50	10.40
−5	−2	5	1.30	8.40
5	2	−5	3.80	4.10
−5	2	5	−5.00	7.90
−5	2	5	3.10	8.20
−5	2	5	20.00	9.60
4	−2	−5	37.20	6.90
4	−2	−5	45.10	11.20
−4	−2	5	30.30	10.50
4	−2	−5	36.50	7.40
4	−2	−5	33.00	12.30

-4	-2	5	46.20	11.60
-4	-2	5	27.20	6.60
4	2	-5	36.30	6.50
-4	2	5	25.20	8.80
4	2	-5	9.70	9.00
-4	2	5	16.40	8.10
-4	2	5	31.40	10.10
-3	-2	5	56.49	7.40
3	-2	-5	56.59	7.90
-3	-2	5	54.69	8.00
-3	-2	5	60.09	13.20
3	-2	-5	87.99	16.20
3	-2	-5	64.29	10.70
-3	-2	5	54.09	10.00
3	2	-5	65.89	8.00
3	2	-5	85.09	14.20
-3	2	5	34.20	9.80
-3	2	5	80.39	14.00
-3	2	5	62.59	7.80
-3	2	5	78.29	13.50
3	2	-5	32.20	9.90
2	-2	-5	-1.90	5.20
2	-2	-5	0.90	3.70
2	-2	-5	3.10	5.40
-2	-2	5	-0.10	4.20
-2	-2	5	5.30	2.80
2	-2	-5	7.00	3.40
-2	-2	5	5.00	2.70
-2	2	5	0.80	4.90
-2	2	5	4.20	3.20
2	2	-5	4.50	2.40

-2	2	5	-0.30	6.90
2	2	-5	10.20	6.70
2	2	-5	6.00	5.10
1	-2	-5	397.86	19.70
-1	-2	5	406.16	13.30
-1	-2	5	397.96	12.50
1	-2	-5	405.06	24.90
1	-2	-5	449.95	24.40
-1	-2	5	384.36	20.30
-1	2	5	415.96	25.50
1	2	-5	382.26	14.40
1	2	-5	378.46	14.00
-1	2	5	379.56	16.60
-1	2	5	397.26	22.70
1	2	-5	383.36	19.90
0	-2	-5	134.09	16.60
0	-2	5	155.08	12.20
0	-2	5	138.99	6.20
0	-2	-5	133.89	11.20
0	-2	-5	122.59	11.90
0	-2	5	134.29	5.80
0	2	5	143.79	9.30
0	2	-5	132.49	7.20
0	2	5	129.29	10.70
0	2	-5	137.29	8.00
0	2	5	137.39	12.60
0	2	5	135.89	13.90
0	2	-5	145.39	11.20
1	-2	5	316.57	16.00
-1	-2	-5	295.47	15.80
-1	-2	-5	307.37	19.20

-1	-2	-5	280.97	12.60
1	-2	5	305.07	8.40
1	-2	5	300.67	8.20
-1	-2	-5	306.67	18.20
-1	-2	-5	286.57	19.50
1	2	5	333.07	21.20
1	2	5	275.47	15.40
1	2	5	310.47	14.00
-1	2	-5	302.17	16.40
-1	2	-5	296.97	14.60
-1	2	-5	308.67	10.40
2	-2	5	49.20	2.50
-2	-2	-5	39.90	6.20
2	-2	5	42.10	2.30
-2	-2	-5	45.30	9.80
-2	-2	-5	41.20	9.50
-2	-2	-5	53.49	14.30
-2	-2	-5	61.49	7.00
-2	-2	-5	37.70	9.10
-2	-2	-5	43.80	6.60
-2	2	-5	45.10	7.60
-2	2	-5	49.40	8.40
2	2	5	53.19	6.60
-2	2	-5	43.00	4.90
2	2	5	52.09	9.50
-2	2	-5	48.50	10.50
2	2	5	40.90	5.50
-2	2	-5	41.00	4.30
-2	2	-5	51.19	7.30
2	2	5	51.09	7.60
2	2	5	47.70	8.90

-2	2	-5	50.79	5.40
-3	-2	-5	213.68	11.40
3	-2	5	202.88	18.30
-3	-2	-5	206.18	16.90
-3	-2	-5	199.78	17.30
3	-2	5	201.68	7.40
-3	-2	-5	197.78	11.60
-3	-2	-5	178.78	16.40
3	-2	5	205.68	6.70
-3	-2	-5	198.28	17.30
3	-2	5	195.88	13.10
-3	-2	-5	206.78	11.10
3	2	5	225.18	11.20
-3	2	-5	229.28	13.40
-3	2	-5	202.38	9.50
-3	2	-5	194.08	7.50
-3	2	-5	193.98	13.90
3	2	5	201.88	17.00
3	2	5	180.38	11.80
3	2	5	202.88	11.20
-3	2	-5	203.78	18.30
3	2	5	176.08	16.30
3	2	5	209.18	17.40
-3	2	-5	171.88	14.50
-3	2	-5	204.88	8.60
-3	2	-5	200.28	8.90
-4	-2	-5	2.20	3.10
4	-2	5	1.70	2.70
-4	-2	-5	2.00	2.80
-4	-2	-5	1.40	5.70
4	-2	5	2.20	6.20

4	−2	5	2.70	2.20
−4	2	−5	6.40	5.00
−4	2	−5	−3.90	6.00
4	2	5	5.40	3.30
−4	2	−5	1.10	2.70
4	2	5	4.70	3.20
4	2	5	0.60	3.80
−5	−2	−5	73.69	14.90
−5	−2	−5	51.09	6.50
−5	−2	−5	44.90	11.70
5	−2	5	56.69	6.30
5	−2	5	56.89	5.30
−5	2	−5	47.10	7.80
−5	2	−5	44.40	13.70
5	2	5	58.79	7.60
−5	2	−5	60.69	10.90
5	2	5	64.39	8.40
−6	−2	−5	9.20	8.90
6	−2	5	8.60	4.60
−6	−2	−5	16.60	11.60
−6	−2	−5	1.70	3.40
6	2	5	2.20	4.30
6	2	5	1.90	4.60
−6	2	−5	8.90	8.30
−6	2	−5	−4.30	5.70
−7	−2	−5	21.20	11.40
−7	−2	−5	23.40	11.60
7	−2	5	36.10	6.60
7	−2	5	27.00	5.90
−7	2	−5	30.10	11.30
−7	2	−5	30.20	12.70

7	2	5	31.70	8.00
7	2	5	12.30	5.70
8	−2	5	9.70	5.90
8	−2	5	22.40	7.50
−8	−2	−5	25.70	14.60
−8	−2	−5	15.30	13.70
−8	2	−5	3.30	9.30
−8	2	−5	−2.10	13.50
8	2	5	18.60	6.60
8	2	5	0.10	6.80
9	−2	5	−5.80	6.00
−9	−2	−5	11.80	13.80
−9	−2	−5	1.80	14.50
−9	2	−5	−9.30	11.70
−9	2	−5	−4.60	12.50
−10	−2	−5	−22.60	17.30
−10	−2	−5	5.10	17.70
10	−2	5	−1.50	7.70
10	2	5	3.90	9.20
−10	2	−5	9.60	14.90
−10	2	−5	8.60	17.30
−11	−2	−5	2.60	19.40
−11	−2	−5	11.50	20.50
−11	2	−5	18.40	18.80
−11	2	−5	−23.30	18.30
11	2	5	13.10	11.80
11	−3	−5	−25.20	14.90
−11	−3	5	15.30	21.90
11	−3	−5	21.30	12.80
−11	−3	5	47.70	21.30
−11	−3	5	−11.50	22.50

11	3	-5	13.20	10.30
-11	3	5	-4.10	20.80
-11	3	5	-26.60	22.10
-11	3	5	-5.10	24.90
-10	-3	5	0.10	21.00
10	-3	-5	-3.60	12.70
-10	-3	5	13.10	16.20
10	-3	-5	6.30	10.40
-10	-3	5	4.70	19.60
10	3	-5	-8.20	9.00
-10	3	5	-17.10	19.20
-10	3	5	-0.90	16.60
-10	3	5	-0.30	16.70
-9	-3	5	3.40	15.20
9	-3	-5	19.30	9.10
9	-3	-5	11.90	11.60
-9	-3	5	-10.20	12.10
-9	-3	5	6.00	13.00
-9	3	5	17.00	15.10
-9	3	5	11.90	16.70
9	3	-5	31.40	10.50
-9	3	5	10.30	13.90
-8	-3	5	-9.30	9.80
8	-3	-5	-8.80	6.40
-8	-3	5	-3.80	12.90
-8	-3	5	3.30	13.60
8	-3	-5	9.80	9.80
-8	3	5	4.80	12.00
8	3	-5	10.70	7.90
-8	3	5	6.80	12.20
-8	3	5	-13.10	14.40

−7	−3	5	153.18	26.10
7	−3	−5	146.09	15.90
−7	−3	5	148.49	20.20
7	−3	−5	167.58	21.40
−7	−3	5	192.08	28.80
−7	3	5	208.98	29.50
7	3	−5	183.38	14.60
−7	3	5	186.78	26.60
−7	3	5	200.88	28.60
−6	−3	5	72.29	19.20
−6	−3	5	65.49	13.50
−6	−3	5	75.29	12.30
6	−3	−5	60.59	10.60
6	−3	−5	63.59	13.40
−6	−3	5	56.79	16.30
−6	3	5	96.09	19.70
6	3	−5	49.00	9.30
−6	3	5	93.49	21.40
−6	3	5	119.99	21.40
5	−3	−5	209.78	19.10
−5	−3	5	182.58	22.60
5	−3	−5	205.98	14.70
−5	−3	5	228.88	24.30
−5	−3	5	216.28	15.90
−5	3	5	216.58	24.00
−5	3	5	248.68	25.50
5	3	−5	217.08	13.90
−5	3	5	224.88	26.20
−4	−3	5	341.27	28.20
−4	−3	5	358.46	17.50
4	−3	−5	367.36	22.90

−4	−3	5	358.06	25.10
−4	−3	5	375.46	18.50
4	−3	−5	362.96	25.40
4	−3	−5	343.47	17.70
−4	3	5	319.37	25.40
4	3	−5	383.36	17.20
−4	3	5	386.46	28.90
4	3	−5	353.56	25.20
−4	3	5	353.26	25.00
3	−3	−5	454.05	20.10
−3	−3	5	457.65	16.80
−3	−3	5	474.25	30.70
−3	−3	5	428.16	18.60
−3	−3	5	454.85	25.80
3	−3	−5	457.25	26.20
3	−3	−5	408.66	24.00
−3	−3	5	461.05	14.90
3	−3	−5	426.26	29.20
3	3	−5	413.76	24.50
3	3	−5	440.06	17.80
−3	3	5	468.75	29.60
−3	3	5	457.45	30.00
−3	3	5	471.95	29.50
3	3	−5	446.16	36.40
2	−3	−5	71.69	15.60
2	−3	−5	90.79	15.10
−2	−3	5	102.49	13.20
2	−3	−5	82.59	9.90
−2	−3	5	95.09	6.40
2	−3	−5	79.29	11.50
2	−3	−5	76.59	11.90

2	3	−5	74.39	8.90
2	3	−5	87.09	14.80
2	3	−5	88.39	11.70
−2	3	5	102.09	16.40
−2	3	5	86.29	9.50
−2	3	5	64.39	12.30
2	3	−5	78.09	6.70
1	−3	−5	154.48	16.70
1	−3	−5	214.48	12.60
−1	−3	5	207.28	7.90
1	−3	−5	231.58	15.80
1	−3	−5	211.88	15.10
−1	−3	5	212.88	14.40
−1	3	5	195.48	18.40
1	3	−5	233.28	28.50
1	3	−5	227.28	15.20
−1	3	5	194.68	11.80
1	3	−5	200.28	9.30
−1	3	5	211.48	15.00
−1	3	5	199.98	17.60
0	−3	−5	284.47	21.10
0	−3	−5	258.37	16.80
0	−3	5	303.77	16.40
0	−3	−5	307.47	21.00
0	−3	−5	315.87	15.00
0	3	5	273.87	19.50
0	3	5	337.37	18.80
0	3	−5	322.47	15.00
0	3	−5	330.77	15.10
0	3	5	319.17	14.80
0	3	5	324.57	22.70

0	3	−5	305.77	13.00
−1	−3	−5	4.50	5.10
1	−3	5	1.00	3.10
−1	−3	−5	7.30	3.30
−1	−3	−5	18.40	7.90
−1	−3	−5	10.70	3.40
−1	−3	−5	4.20	4.90
1	3	5	5.20	3.10
−1	3	−5	13.10	4.50
1	3	5	6.30	3.80
1	3	5	13.40	6.30
1	3	5	7.30	5.20
−1	3	−5	10.70	2.10
−1	3	−5	16.30	3.80
−1	3	−5	12.40	3.50
−1	3	−5	11.20	2.30
1	3	5	5.20	4.50
−2	−3	−5	791.82	34.90
−2	−3	−5	804.72	27.00
−2	−3	−5	803.82	34.00
−2	−3	−5	769.92	24.80
−2	−3	−5	711.83	30.40
−2	3	−5	784.42	21.80
2	3	5	781.32	25.70
2	3	5	846.42	29.40
−2	3	−5	777.82	23.00
−2	3	−5	812.62	20.10
2	3	5	756.12	34.20
2	3	5	813.72	26.20
−2	3	−5	778.22	26.40
−2	3	−5	781.02	23.60

−2	3	−5	797.92	35.90
2	3	5	736.13	31.50
−3	−3	−5	118.39	16.80
−3	−3	−5	109.99	9.30
−3	−3	−5	95.19	8.70
−3	−3	−5	96.79	14.30
−3	−3	−5	133.79	17.80
3	3	5	105.39	14.80
3	3	5	93.69	8.30
3	3	5	118.59	16.10
−3	3	−5	98.29	6.30
−3	3	−5	96.89	5.70
3	3	5	117.89	10.00
−3	3	−5	110.19	9.80
−3	3	−5	105.99	6.10
−3	3	−5	104.69	15.70
3	3	5	82.49	9.10
−4	−3	−5	267.47	22.30
−4	−3	−5	301.27	23.20
−4	−3	−5	300.67	15.90
−4	−3	−5	259.77	15.80
4	−3	5	259.67	15.80
−4	3	−5	285.07	11.60
−4	3	−5	272.87	11.80
4	3	5	279.17	22.20
−4	3	−5	279.87	15.80
4	3	5	311.47	17.00
−4	3	−5	256.97	16.20
4	3	5	283.97	15.00
−5	−3	−5	5.80	8.50
−5	−3	−5	33.40	11.60

−5	3	−5	15.10	4.70
5	3	5	22.90	5.60
−5	3	−5	18.40	9.00
5	3	5	20.70	9.90
5	3	5	17.80	5.40
6	−3	5	4.20	5.40
−6	−3	−5	3.40	8.90
−6	−3	−5	3.70	9.20
6	3	5	1.60	4.20
6	3	5	9.10	5.20
−6	3	−5	6.90	7.70
−6	3	−5	0.00	6.20
7	−3	5	47.90	6.30
−7	−3	−5	60.89	15.90
−7	−3	−5	46.60	17.30
7	−3	5	48.50	5.70
−7	−3	−5	58.69	7.90
−7	3	−5	25.00	13.40
7	3	5	59.39	9.60
−7	3	−5	44.50	11.40
7	3	5	43.50	9.20
8	−3	5	61.39	8.70
−8	−3	−5	50.59	16.30
−8	−3	−5	43.20	15.90
−8	3	−5	66.79	20.30
−8	3	−5	44.40	14.60
8	3	5	51.29	10.20
8	3	5	55.19	11.00
9	−3	5	−8.40	5.00
−9	−3	−5	17.10	15.70
−9	−3	−5	3.70	13.60

-9	3	-5	2.40	12.70
9	3	5	2.40	7.50
-9	3	-5	4.20	12.30
-10	-3	-5	-5.90	19.30
-10	-3	-5	22.10	19.40
10	3	5	16.20	11.00
-10	3	-5	20.40	17.90
-10	3	-5	7.50	14.10
-11	-3	-5	-25.70	19.50
-11	-3	-5	8.70	20.70
11	3	5	0.90	11.30
-11	3	-5	-18.80	19.90
-11	-4	5	15.30	24.00
-11	-4	5	28.70	23.40
-11	-4	5	-11.90	20.90
11	-4	-5	14.20	16.50
11	-4	-5	0.10	12.20
-11	4	5	22.60	25.60
-11	4	5	-21.60	24.10
11	4	-5	-5.10	11.20
-11	4	5	26.80	20.90
-10	-4	5	13.80	20.20
-10	-4	5	-13.00	16.40
-10	-4	5	-5.20	15.80
10	-4	-5	23.00	14.40
10	-4	-5	-4.30	10.20
-10	4	5	-1.60	17.40
-10	4	5	26.80	18.20
10	4	-5	12.50	10.70
-10	4	5	-22.30	17.90
-9	-4	5	14.50	13.70

9	−4	−5	17.10	11.90
−9	−4	5	−7.10	14.50
−9	−4	5	21.10	18.80
−9	4	5	−11.50	14.40
−9	4	5	22.20	16.40
9	4	−5	17.80	9.40
−9	4	5	12.90	14.20
−8	−4	5	−8.10	13.40
−8	−4	5	30.70	14.70
8	−4	−5	1.30	6.50
8	−4	−5	−1.60	9.10
−8	−4	5	4.70	10.90
−8	4	5	3.10	12.70
−8	4	5	−6.40	11.40
8	4	−5	6.30	7.20
7	−4	−5	8.80	6.80
−7	−4	5	−1.30	8.10
−7	−4	5	18.30	12.80
−7	−4	5	−6.80	11.70
7	−4	−5	−19.20	7.90
−7	4	5	1.10	10.20
−7	4	5	−3.20	11.20
7	4	−5	−4.20	6.00
−7	4	5	7.00	9.40
−6	−4	5	49.10	14.00
−6	−4	5	77.19	18.90
6	−4	−5	57.29	12.40
−6	−4	5	57.29	10.90
−6	−4	5	41.30	10.40
6	−4	−5	59.09	10.40
−6	4	5	55.49	15.80

-6	4	5	30.50	13.30
6	4	-5	46.00	8.90
-6	4	5	62.39	19.60
-5	-4	5	18.40	6.60
5	-4	-5	3.70	6.50
-5	-4	5	19.90	12.10
-5	-4	5	17.80	11.00
-5	-4	5	17.50	6.00
5	-4	-5	10.40	6.10
-5	4	5	10.00	8.60
-5	4	5	3.70	7.70
5	4	-5	28.30	7.30
-5	4	5	17.10	9.60
-4	-4	5	32.60	5.50
-4	-4	5	33.10	10.10
4	-4	-5	26.80	8.20
-4	-4	5	41.30	12.80
-4	-4	5	38.80	5.80
4	-4	-5	9.40	7.90
-4	4	5	11.90	8.90
4	4	-5	21.20	10.30
-4	4	5	11.20	9.30
-4	4	5	24.80	9.60
3	-4	-5	445.16	20.50
3	-4	-5	424.26	24.30
-3	-4	5	420.16	29.60
-3	-4	5	398.96	24.30
3	-4	-5	416.26	24.20
-3	-4	5	407.16	15.80
-3	4	5	397.36	28.20
-3	4	5	436.06	31.20

3	4	-5	412.26	24.10
3	4	-5	484.85	18.40
2	-4	-5	154.98	21.70
2	-4	-5	164.88	14.70
2	-4	-5	145.59	22.50
2	-4	-5	128.69	10.00
-2	-4	5	143.49	13.90
2	-4	-5	145.99	13.60
-2	-4	5	158.08	7.70
2	4	-5	135.19	13.20
-2	4	5	147.79	20.60
-2	4	5	158.68	20.90
2	4	-5	175.18	22.80
2	4	-5	145.59	12.00
-2	4	5	132.89	11.00
-2	4	5	172.98	14.90
2	4	-5	133.59	9.50
1	-4	-5	1292.07	38.60
1	-4	-5	1248.18	44.60
-1	-4	5	1232.08	29.90
1	-4	-5	1326.67	41.80
-1	-4	5	1259.47	29.20
1	-4	-5	1253.57	49.50
-1	-4	5	1285.97	40.90
1	-4	-5	1227.38	51.29
1	4	-5	1283.27	50.19
-1	4	5	1369.36	38.80
1	4	-5	1267.37	39.10
-1	4	5	1211.28	50.89
-1	4	5	1291.77	44.50
-1	4	5	1253.37	38.70

1	4	−5	1275.17	32.60
−1	4	5	1357.76	52.79
1	4	−5	1239.08	39.00
0	−4	−5	577.54	22.00
0	−4	−5	572.24	31.00
0	−4	−5	569.04	25.90
0	−4	−5	602.84	31.30
0	−4	−5	577.44	25.50
0	4	5	534.75	30.60
0	4	−5	537.45	19.30
0	4	5	530.35	25.20
0	4	−5	541.75	17.90
0	4	5	575.44	21.80
0	4	−5	582.04	22.30
0	4	5	567.44	31.10
0	4	−5	574.24	15.90
0	4	−5	558.74	19.70
0	4	5	539.75	21.70
1	−4	5	169.28	10.10
−1	−4	−5	150.98	16.40
−1	−4	−5	155.08	9.80
−1	−4	−5	175.48	20.30
−1	−4	−5	166.78	13.80
−1	−4	−5	169.48	12.90
−1	−4	−5	189.38	21.10
−1	−4	−5	176.08	11.40
−1	4	−5	161.78	5.90
1	4	5	142.59	10.00
−1	4	−5	151.28	5.80
−1	4	−5	171.58	7.70
−1	4	−5	163.18	7.70

−1	4	−5	153.08	9.90
1	4	5	119.59	16.30
1	4	5	125.49	16.60
1	4	5	133.19	11.60
1	4	5	140.19	9.90
−2	−4	−5	59.99	12.20
−2	−4	−5	34.00	7.40
−2	−4	−5	52.69	7.90
−2	−4	−5	47.80	12.10
−2	−4	−5	37.80	7.50
−2	4	−5	38.70	2.80
−2	4	−5	48.80	11.50
−2	4	−5	42.20	3.70
2	4	5	39.40	7.80
2	4	5	55.89	11.40
−2	4	−5	39.10	5.60
2	4	5	69.69	11.60
2	4	5	45.50	7.20
−2	4	−5	37.70	3.40
−2	4	−5	43.80	4.00
−3	−4	−5	615.04	31.10
−3	−4	−5	648.04	33.50
−3	−4	−5	553.64	23.70
−3	4	−5	632.74	19.10
3	4	5	592.14	36.50
−3	4	−5	611.44	32.60
−3	4	−5	597.24	18.80
−3	4	−5	624.94	16.50
3	4	5	636.44	25.20
−3	4	−5	597.64	17.10
3	4	5	573.54	25.30

3	4	5	540.65	29.00
−3	4	−5	594.14	20.90
−4	−4	−5	67.79	9.60
−4	−4	−5	80.19	14.80
−4	−4	−5	86.59	15.80
4	−4	5	81.99	9.70
−4	−4	−5	77.29	9.90
−4	4	−5	77.39	6.10
4	4	5	90.89	10.20
4	4	5	71.39	9.90
−4	4	−5	67.09	9.30
−4	4	−5	63.59	5.70
−4	4	−5	93.49	18.00
4	4	5	49.90	12.40
−4	4	−5	66.09	8.60
−5	−4	−5	54.79	14.40
−5	−4	−5	70.99	14.90
−5	−4	−5	54.19	8.20
−5	4	−5	63.99	10.20
5	4	5	55.89	14.00
−5	4	−5	37.60	11.80
−5	4	−5	51.59	6.60
5	4	5	38.40	8.60
5	4	5	43.60	7.50
−6	−4	−5	85.49	17.60
−6	−4	−5	54.79	16.40
−6	−4	−5	58.39	9.00
−6	4	−5	51.89	11.20
−6	4	−5	66.39	15.80
6	4	5	50.99	9.00
6	4	5	64.49	10.20

−7	−4	−5	3.90	11.40
−7	−4	−5	−2.80	4.90
−7	−4	−5	−4.80	12.40
7	4	5	−14.70	6.70
7	4	5	6.00	6.10
−7	4	−5	7.10	12.40
−7	4	−5	6.70	7.20
−8	−4	−5	31.50	15.40
−8	−4	−5	30.20	16.50
−8	4	−5	10.40	10.10
8	4	5	16.70	8.60
−8	4	−5	19.60	14.40
−9	−4	−5	9.90	14.40
−9	−4	−5	−3.60	15.90
−9	4	−5	−5.50	12.00
9	4	5	1.20	7.10
−9	4	−5	6.70	11.20
−10	−4	−5	9.90	18.10
−10	−4	−5	−30.00	17.60
−10	4	−5	1.00	14.30
−10	4	−5	−6.60	15.40
10	4	5	−0.50	10.30
−10	−5	5	−9.60	18.20
10	−5	−5	−32.30	13.70
−10	−5	5	−7.90	15.20
−10	−5	5	−30.80	19.40
10	−5	−5	−7.90	10.40
10	5	−5	11.30	10.30
−10	5	5	−9.50	17.20
−10	5	5	−10.00	18.20
−10	5	5	−0.30	18.20

-9	-5	5	11.60	14.80
9	-5	-5	16.80	12.90
-9	-5	5	4.80	21.10
-9	-5	5	9.40	16.50
9	-5	-5	10.40	10.50
-9	5	5	13.20	15.60
-9	5	5	12.80	15.80
9	5	-5	19.30	8.30
-9	5	5	24.40	17.80
8	-5	-5	-10.90	9.20
-8	-5	5	5.20	16.30
-8	-5	5	16.50	16.40
-8	-5	5	3.80	10.00
8	-5	-5	4.00	7.90
-8	5	5	2.90	14.80
-8	5	5	-10.40	12.70
8	5	-5	5.10	7.50
-8	5	5	1.80	13.20
7	-5	-5	47.30	12.10
-7	-5	5	67.89	21.30
7	-5	-5	49.30	10.60
-7	-5	5	65.49	19.70
-7	5	5	50.59	16.90
7	5	-5	57.69	10.80
-7	5	5	67.49	22.90
-7	5	5	51.69	17.90
-6	-5	5	15.70	12.40
6	-5	-5	7.60	6.90
-6	-5	5	10.90	7.80
6	-5	-5	14.60	9.90
-6	-5	5	26.50	16.60

6	5	−5	19.90	7.00
−6	5	5	16.30	10.70
−6	5	5	6.60	11.20
−6	5	5	−2.70	12.10
−5	−5	5	120.79	11.00
5	−5	−5	81.59	14.90
−5	−5	5	106.59	20.90
−5	−5	5	128.69	20.70
5	−5	−5	128.79	14.10
5	5	−5	105.99	11.50
−5	5	5	118.69	18.20
−5	5	5	82.19	18.30
−5	5	5	114.19	20.40
−4	−5	5	1.00	4.10
−4	−5	5	4.60	6.90
−4	−5	5	0.30	3.70
−4	−5	5	1.50	7.50
4	−5	−5	−2.10	5.10
4	−5	−5	−10.20	7.20
−4	5	5	−4.40	6.70
4	5	−5	1.50	3.90
−4	5	5	3.60	8.80
−4	5	5	5.20	6.10
−3	−5	5	125.29	8.20
3	−5	−5	104.19	14.40
3	−5	−5	145.29	19.90
3	−5	−5	111.19	12.00
−3	−5	5	125.19	16.70
−3	5	5	122.09	21.10
3	5	−5	121.49	16.30
−3	5	5	106.59	19.30

3	5	-5	106.49	9.00
-2	-5	5	48.30	9.20
2	-5	-5	40.30	10.10
2	-5	-5	49.60	8.50
2	-5	-5	50.09	8.10
-2	-5	5	48.60	4.50
-2	5	5	70.49	16.70
-2	5	5	34.10	10.80
2	5	-5	41.60	9.00
2	5	-5	53.79	14.50
1	-5	-5	2.10	4.50
-1	-5	5	1.20	4.40
1	-5	-5	5.60	3.10
1	-5	-5	2.70	4.40
-1	5	5	-0.60	5.70
1	5	-5	-1.40	2.00
1	5	-5	9.30	4.00
1	5	-5	3.70	7.90
1	5	-5	2.10	4.10
-1	5	5	-3.40	5.50
-1	5	5	5.10	4.60
0	-5	-5	129.49	9.80
0	-5	-5	137.99	15.70
0	-5	-5	156.88	12.30
0	5	5	142.39	11.00
0	5	-5	139.29	6.30
0	5	-5	129.89	8.00
0	5	5	135.79	18.60
0	5	-5	119.29	10.10
0	5	5	116.69	17.60
0	5	-5	147.69	11.30

−1	−5	−5	6.20	4.30
−1	−5	−5	−0.40	3.20
1	−5	5	−2.50	2.60
−1	−5	−5	0.60	4.10
−1	−5	−5	−8.70	5.20
−1	−5	−5	−0.90	4.80
−1	5	−5	0.00	1.20
1	5	5	1.40	4.70
−1	5	−5	−1.10	3.50
−1	5	−5	1.90	1.10
−1	5	−5	1.80	3.40
1	5	5	3.60	3.50
1	5	5	0.60	6.00
−2	−5	−5	2.70	4.40
−2	−5	−5	−1.90	2.80
−2	−5	−5	6.60	6.40
2	5	5	−6.20	5.50
2	5	5	2.70	4.40
−2	5	−5	−1.10	5.30
2	5	5	0.80	4.60
2	5	5	4.40	5.50
−2	5	−5	−0.60	3.00
−3	−5	−5	197.28	14.50
−3	−5	−5	162.58	12.20
−3	−5	−5	197.08	17.80
−3	5	−5	183.98	6.80
3	5	5	201.88	12.90
3	5	5	166.38	17.00
3	5	5	198.18	19.40
−3	5	−5	177.48	18.90
−3	5	−5	191.48	7.30

−3	5	−5	199.18	10.70
−4	−5	−5	207.98	14.50
4	−5	5	206.18	12.30
−4	−5	−5	169.18	22.70
−4	−5	−5	178.68	19.30
−4	5	−5	212.78	9.00
4	5	5	232.88	21.70
−4	5	−5	198.98	20.60
−4	5	−5	200.08	8.80
−4	5	−5	189.78	10.80
4	5	5	200.68	13.90
−4	5	−5	208.78	10.30
−5	−5	−5	5.60	9.40
−5	−5	−5	15.20	8.30
−5	−5	−5	12.40	5.30
−5	5	−5	8.20	9.40
−5	5	−5	3.50	2.90
5	5	5	9.70	4.90
−5	5	−5	2.30	4.70
−5	5	−5	10.00	5.20
5	5	5	10.90	8.10
−6	−5	−5	61.29	16.80
−6	−5	−5	85.59	13.50
−6	−5	−5	72.99	16.60
6	5	5	106.99	19.20
6	5	5	50.79	9.30
−6	5	−5	77.89	18.40
−6	5	−5	91.79	12.70
−7	−5	−5	22.90	7.40
−7	−5	−5	−1.20	13.60
−7	−5	−5	0.00	11.30

−7	5	−5	23.90	8.30
−7	5	−5	27.40	13.70
7	5	5	8.10	6.50
−8	−5	−5	−7.30	6.10
−8	−5	−5	−4.90	14.30
−8	−5	−5	−3.00	12.70
8	5	5	−5.70	6.90
−8	5	−5	−9.00	12.70
−8	5	−5	7.10	9.40
−9	−5	−5	2.90	17.80
−9	−5	−5	−15.90	14.90
−9	5	−5	4.20	11.70
9	5	5	−1.00	8.00
−9	5	−5	20.70	16.60
−10	−5	−5	3.60	20.20
−10	5	−5	−1.30	18.70
−10	5	−5	7.30	14.10
10	5	5	13.20	11.10
10	−6	−5	27.60	15.50
−10	−6	5	−19.00	20.30
−10	−6	5	22.20	19.40
10	−6	−5	1.80	11.90
−10	6	5	−7.20	26.60
10	6	−5	18.20	10.30
−10	6	5	23.70	21.80
−9	−6	5	21.70	14.50
9	−6	−5	20.00	10.40
9	−6	−5	9.00	13.20
−9	−6	5	−6.20	18.90
−9	−6	5	−8.30	18.70
−9	6	5	2.00	16.30

9	6	−5	7.90	8.80
−9	6	5	28.10	18.50
−9	6	5	35.60	22.20
8	−6	−5	0.30	9.20
−8	−6	5	1.10	15.90
8	−6	−5	16.10	9.80
−8	−6	5	9.20	15.70
8	6	−5	1.90	7.60
−8	6	5	−0.60	15.10
−8	6	5	3.30	12.40
−8	6	5	−1.30	13.40
−7	−6	5	51.89	16.50
−7	−6	5	70.19	23.50
−7	−6	5	71.79	15.50
7	−6	−5	68.89	11.30
7	−6	−5	81.99	16.30
−7	6	5	68.49	19.20
−7	6	5	67.49	17.00
6	−6	−5	9.80	7.50
−6	−6	5	−3.30	11.00
−6	−6	5	−1.60	7.70
−6	−6	5	17.20	12.50
6	−6	−5	12.70	7.70
−6	6	5	5.90	11.40
−6	6	5	13.20	11.50
−6	6	5	3.70	9.30
6	6	−5	22.60	6.70
−5	−6	5	127.49	25.20
−5	−6	5	90.59	11.20
5	−6	−5	112.89	17.60
5	−6	−5	115.49	21.60

−5	−6	5	99.39	18.60
5	−6	−5	114.89	13.10
5	6	−5	116.19	13.00
−5	6	5	107.79	22.70
−5	6	5	116.99	18.40
−5	6	5	76.59	17.00
4	−6	−5	30.70	7.80
−4	−6	5	43.30	13.60
−4	−6	5	19.60	8.20
−4	−6	5	31.20	6.70
4	−6	−5	23.00	7.70
−4	−6	5	26.80	5.50
−4	6	5	17.20	13.80
−4	6	5	40.00	10.20
4	6	−5	21.20	6.40
4	6	−5	17.10	10.20
−4	6	5	10.60	9.40
−3	−6	5	102.29	15.50
−3	−6	5	119.79	8.30
3	−6	−5	131.09	11.50
3	−6	−5	120.99	13.40
3	−6	−5	141.79	18.00
−3	6	5	137.29	22.90
−3	6	5	112.59	20.70
3	6	−5	132.39	16.90
3	6	−5	123.09	9.60
−2	−6	5	29.90	4.00
2	−6	−5	33.90	9.40
2	−6	−5	33.20	7.60
2	−6	−5	38.80	7.60
−2	6	5	26.80	11.70

2	6	−5	20.20	6.60
−2	6	5	36.90	13.40
2	6	−5	36.30	5.50
−1	−6	5	0.70	4.10
1	−6	−5	1.30	5.40
1	−6	−5	3.40	3.50
1	−6	−5	5.30	4.70
1	−6	−5	0.70	4.30
−1	6	5	−0.70	8.90
1	6	−5	9.40	3.90
−1	6	5	2.90	7.70
−1	6	5	1.70	4.00
1	6	−5	−3.10	8.10
1	6	−5	4.70	2.70
1	6	−5	5.00	2.40
0	−6	5	285.97	16.60
0	−6	5	264.17	15.10
0	−6	−5	252.37	21.30
0	−6	−5	252.97	14.70
0	−6	−5	268.37	15.20
0	6	−5	270.67	11.00
0	6	5	242.58	22.50
0	6	−5	242.38	15.70
0	6	5	277.87	15.60
0	6	−5	270.37	9.40
0	6	5	251.07	24.20
1	−6	5	33.10	6.80
−1	−6	−5	22.90	5.60
1	−6	5	23.50	3.70
−1	−6	−5	28.00	10.80
−1	−6	−5	32.40	7.50

−1	6	−5	28.00	2.10
−1	6	−5	30.50	6.50
−1	6	−5	36.30	8.20
1	6	5	21.60	9.90
−2	−6	−5	38.50	12.00
−2	−6	−5	51.49	8.70
2	−6	5	27.90	5.50
−2	−6	−5	42.40	9.50
−2	−6	−5	31.30	9.20
−2	6	−5	36.20	7.30
2	6	5	38.20	8.80
2	6	5	27.90	9.70
2	6	5	44.70	8.60
−2	6	−5	31.60	9.20
−2	6	−5	53.99	13.00
−3	−6	−5	33.40	7.20
−3	−6	−5	15.80	7.20
3	−6	5	30.70	5.80
−3	−6	−5	17.30	9.20
−3	−6	−5	27.40	7.50
−3	6	−5	16.00	8.50
−3	6	−5	24.80	9.60
3	6	5	16.30	7.90
−3	6	−5	18.70	2.40
3	6	5	18.90	5.70
3	6	5	23.20	9.70
−3	6	−5	25.60	5.70
−4	−6	−5	0.00	9.20
−4	−6	−5	18.20	8.40
−4	−6	−5	9.10	6.40
−4	6	−5	9.20	2.90

4	6	5	3.60	5.10
-4	6	-5	8.00	2.80
-4	6	-5	-4.00	8.20
-4	6	-5	17.70	4.50
-4	6	-5	7.50	9.70
4	6	5	10.40	9.50
-5	-6	-5	114.69	23.10
-5	-6	-5	99.69	13.00
-5	-6	-5	81.09	17.40
5	6	5	95.69	12.20
-5	6	-5	130.89	19.90
-5	6	-5	94.49	9.30
-5	6	-5	115.49	8.20
-5	6	-5	83.69	7.80
-5	6	-5	84.59	18.00
-5	6	-5	100.69	11.50
5	6	5	104.19	18.70
-6	-6	-5	21.40	13.30
-6	-6	-5	29.30	8.30
-6	-6	-5	3.60	12.30
6	6	5	5.60	5.70
-6	6	-5	8.30	6.20
-6	6	-5	21.20	6.70
-6	6	-5	15.80	10.90
6	6	5	-0.60	9.90
-7	-6	-5	23.50	13.80
-7	-6	-5	4.60	7.40
-7	-6	-5	7.70	15.30
-7	6	-5	9.40	6.80
-7	6	-5	4.20	12.50
7	6	5	5.90	7.00

−8	−6	−5	−5.80	12.60
−8	−6	−5	29.10	15.00
−8	−6	−5	21.90	9.60
8	6	5	11.40	7.90
−8	6	−5	4.00	9.30
−8	6	−5	27.30	17.30
−8	6	−5	1.40	14.10
−9	−6	−5	16.30	16.90
−9	−6	−5	14.30	17.50
−9	6	−5	−4.10	18.90
9	6	5	4.20	10.00
−9	6	−5	35.20	17.70
−9	6	−5	−0.30	11.30
−10	−6	−5	−0.70	20.60
−10	−6	−5	22.50	24.10
−10	6	−5	−24.10	20.30
−10	6	−5	16.30	14.80
−10	6	−5	9.50	24.30
10	−7	−5	−3.40	13.20
−10	−7	5	−13.30	22.50
−10	−7	5	7.80	23.90
−10	−7	5	27.00	21.30
10	−7	−5	−26.60	14.30
10	7	−5	13.90	11.70
−10	7	5	31.50	21.90
−10	7	5	−10.50	24.80
−10	7	5	−12.90	19.70
9	−7	−5	8.70	10.40
−9	−7	5	19.40	20.40
−9	−7	5	8.70	19.00
9	7	−5	16.00	11.20

−9	7	5	18.80	16.80
−9	7	5	−8.70	18.40
−9	7	5	19.10	23.50
8	−7	−5	6.90	8.20
−8	−7	5	20.40	20.10
8	−7	−5	−5.60	10.70
−8	7	5	−20.80	14.00
8	7	−5	4.20	7.80
−8	7	5	22.80	15.00
−8	7	5	−3.50	18.70
−7	−7	5	7.60	16.50
7	−7	−5	4.40	7.30
−7	−7	5	−21.80	12.30
−7	−7	5	−5.00	12.50
7	−7	−5	−10.00	9.20
7	7	−5	10.30	7.60
−7	7	5	2.10	11.10
−7	7	5	−7.40	13.30
−7	7	5	−25.50	12.20
6	−7	−5	42.20	9.90
−6	−7	5	73.29	17.80
−6	−7	5	51.29	18.30
−6	−7	5	69.09	13.00
6	−7	−5	42.30	13.00
−6	7	5	60.79	18.20
−6	7	5	50.59	19.70
−6	7	5	49.99	14.00
6	7	−5	51.19	11.30
−5	−7	5	−3.00	7.10
5	−7	−5	0.80	7.70
5	−7	−5	3.60	6.40

5	−7	−5	−19.90	9.70
−5	−7	5	13.60	14.30
−5	−7	5	−1.30	9.00
−5	7	5	0.30	11.40
5	7	−5	11.10	5.60
−5	7	5	11.50	7.80
−5	7	5	7.30	11.20
−4	−7	5	144.59	20.40
4	−7	−5	162.48	18.00
−4	−7	5	137.09	10.60
−4	−7	5	163.58	12.30
4	−7	−5	168.38	12.90
4	−7	−5	146.79	19.70
−4	7	5	136.29	22.10
4	7	−5	160.58	12.60
−4	7	5	138.19	26.50
4	7	−5	150.28	20.60
3	−7	−5	140.59	11.40
−3	−7	5	108.89	15.50
3	−7	−5	114.19	12.60
3	−7	−5	175.98	18.20
−3	−7	5	136.89	8.20
−3	7	5	143.89	25.20
3	7	−5	134.59	16.30
3	7	−5	141.19	10.50
−3	7	5	104.19	20.30
2	−7	−5	6.70	5.10
−2	−7	5	8.40	2.00
2	−7	−5	16.30	5.70
2	−7	−5	7.20	7.00
2	7	−5	20.90	6.40

2	7	−5	13.80	4.20
−2	7	5	15.10	9.40
2	7	−5	8.70	2.40
−2	7	5	−3.30	9.10
−1	−7	5	57.69	9.20
−1	−7	5	70.29	11.00
1	−7	−5	72.79	8.60
1	−7	−5	52.09	7.50
1	−7	−5	51.79	11.60
1	−7	−5	59.59	7.60
1	7	−5	66.99	7.00
−1	7	5	31.50	10.90
1	7	−5	58.39	6.30
−1	7	5	60.69	10.10
1	7	−5	70.79	8.60
1	7	−5	76.69	15.80
−1	7	5	48.50	13.70
0	−7	−5	108.19	13.30
0	−7	5	105.39	10.20
0	−7	−5	83.79	13.60
0	−7	−5	127.59	9.70
0	7	5	139.59	22.40
0	7	5	133.79	11.90
0	7	−5	104.39	7.10
0	7	−5	107.39	14.10
0	7	−5	128.59	19.30
−1	−7	−5	207.08	25.10
−1	−7	−5	163.98	12.70
−1	−7	−5	153.88	15.80
1	−7	5	159.78	13.30
−1	−7	−5	161.88	12.40

1	7	5	135.19	12.60
-1	7	-5	148.09	5.30
-1	7	-5	143.49	15.70
-1	7	-5	166.18	16.70
1	7	5	136.89	21.50
-2	-7	-5	270.37	18.10
-2	-7	-5	244.18	18.40
-2	-7	-5	261.77	24.30
2	-7	5	273.07	15.80
-2	-7	-5	265.47	15.80
-2	7	-5	269.37	16.30
-2	7	-5	238.88	20.40
2	7	5	241.88	15.90
2	7	5	245.38	23.50
-2	7	-5	242.28	21.00
-3	-7	-5	11.90	6.60
3	-7	5	16.90	5.60
-3	-7	-5	12.70	10.40
-3	-7	-5	28.90	7.90
-3	7	-5	15.30	5.70
-3	7	-5	4.40	9.60
3	7	5	26.30	8.50
3	7	5	16.10	9.00
-3	7	-5	28.50	9.80
3	7	5	9.70	5.10
-4	-7	-5	145.79	15.10
-4	-7	-5	186.18	26.80
-4	-7	-5	148.19	20.60
-4	7	-5	154.78	24.50
4	7	5	175.88	14.30
-4	7	-5	156.08	12.00

−4	7	−5	148.69	6.90
4	7	5	124.69	20.50
−4	7	−5	154.18	21.50
−5	−7	−5	1.40	12.00
−5	−7	−5	8.00	11.10
−5	−7	−5	21.70	8.40
5	7	5	10.60	6.00
−5	7	−5	9.40	11.30
5	7	5	−12.00	8.80
−5	7	−5	10.30	3.60
−5	7	−5	−0.80	8.50
−5	7	−5	14.70	5.80
−6	−7	−5	0.60	11.90
−6	−7	−5	−5.90	13.40
−6	−7	−5	9.50	8.00
−6	7	−5	−1.00	12.50
−6	7	−5	−12.40	5.30
6	7	5	−2.10	10.30
−6	7	−5	−0.50	10.20
−7	−7	−5	−3.60	7.10
−7	−7	−5	−1.20	13.10
−7	−7	−5	−3.70	14.40
−7	7	−5	33.10	14.80
−7	7	−5	−5.50	7.00
−7	7	−5	6.00	12.90
7	7	5	3.80	7.10
−8	−7	−5	22.60	10.80
−8	−7	−5	35.40	17.60
−8	−7	−5	5.90	15.80
−8	7	−5	31.50	16.70
8	7	5	13.90	9.10

−8	7	−5	47.40	22.40
−8	7	−5	55.19	13.50
−9	−7	−5	57.69	23.00
−9	−7	−5	−2.70	9.80
−9	−7	−5	−9.60	19.60
−9	7	−5	45.00	19.20
9	7	5	−2.20	10.20
−9	7	−5	8.90	23.50
−10	−7	−5	28.40	24.10
−10	−7	−5	−6.50	32.40
−10	7	−5	−29.20	19.00
10	7	5	−5.40	11.90
−10	7	−5	26.90	16.20
−10	7	−5	−8.20	22.30
9	−8	−5	14.70	11.00
−9	−8	5	−2.80	22.60
9	−8	−5	14.00	14.50
−9	−8	5	−19.00	22.40
−9	8	5	−5.30	18.40
−9	8	5	43.70	28.70
9	8	−5	11.40	11.50
−9	8	5	25.00	18.40
8	−8	−5	1.60	8.80
8	−8	−5	14.40	14.90
−8	−8	5	9.50	21.40
−8	−8	5	10.10	17.40
−8	8	5	−20.70	13.60
8	8	−5	1.30	8.00
−8	8	5	−11.10	16.00
−8	8	5	−23.60	16.20
−7	−8	5	−6.90	14.00

7	-8	-5	11.80	11.70
-7	-8	5	-9.90	17.00
7	-8	-5	2.10	8.00
-7	8	5	2.90	12.10
-7	8	5	8.60	13.30
7	8	-5	-3.20	8.60
-7	8	5	15.10	15.60
-6	-8	5	3.10	13.90
6	-8	-5	31.30	11.70
-6	-8	5	27.10	14.80
6	-8	-5	16.80	7.30
-6	-8	5	-3.70	11.40
-6	8	5	19.30	15.20
6	8	-5	30.00	9.00
-5	-8	5	73.29	19.80
5	-8	-5	86.19	11.50
-5	-8	5	91.19	12.90
-5	-8	5	94.09	20.90
5	-8	-5	83.39	15.30
5	-8	-5	82.49	16.30
-5	8	5	70.39	14.30
-5	8	5	71.69	20.90
-5	8	5	60.79	16.60
5	8	-5	74.99	11.60
4	-8	-5	9.10	9.00
-4	-8	5	-0.70	10.00
-4	-8	5	-8.60	6.10
4	-8	-5	-2.70	4.80
-4	-8	5	-5.80	4.60
4	-8	-5	-1.30	8.30
4	8	-5	5.90	5.20

4	8	-5	-11.70	10.00
-4	8	5	4.40	13.70
-4	8	5	14.30	11.60
3	-8	-5	9.00	5.80
3	-8	-5	-13.10	7.10
-3	-8	5	-6.10	7.60
-3	-8	5	-2.10	3.00
-3	8	5	0.30	11.90
3	8	-5	-9.20	7.60
-3	8	5	-2.50	9.70
-2	-8	5	169.98	15.70
2	-8	-5	137.09	15.10
-2	-8	5	186.18	22.00
2	-8	-5	157.28	19.90
-2	8	5	142.29	22.10
-2	8	5	105.49	22.30
2	8	-5	140.09	10.10
2	8	-5	137.79	8.70
-2	8	5	149.09	13.80
-1	-8	5	140.09	14.00
1	-8	-5	120.99	17.30
-1	8	5	151.28	16.00
1	8	-5	99.49	10.00
-1	8	5	108.09	19.70
1	8	-5	129.29	11.80
1	8	-5	144.69	9.10
-1	8	5	122.69	24.90
0	-8	5	283.47	15.70
0	-8	-5	275.47	21.30
0	-8	-5	264.97	11.30
0	-8	-5	277.47	25.60

0	-8	5	230.68	17.50
0	-8	-5	256.27	10.90
0	-8	-5	264.47	15.50
0	8	5	236.48	30.80
0	8	5	273.47	17.20
0	8	-5	245.98	27.90
0	8	-5	270.57	19.80
0	8	-5	253.67	10.30
-1	-8	-5	4.60	5.60
-1	-8	-5	4.60	4.60
1	-8	5	-1.00	4.80
-1	-8	-5	-7.60	3.90
-1	-8	-5	4.30	8.90
1	8	5	2.90	4.90
-1	8	-5	-5.60	6.90
-1	8	-5	0.50	8.50
1	8	5	-2.70	6.80
-1	8	-5	1.50	1.70
2	-8	5	8.20	5.30
-2	-8	-5	-11.00	8.00
-2	-8	-5	4.60	5.50
-2	-8	-5	-0.40	6.90
-2	8	-5	-0.50	7.40
2	8	5	0.00	8.70
2	8	5	1.70	4.30
-2	8	-5	-4.20	7.00
3	-8	5	5.70	5.30
-3	-8	-5	-6.80	8.60
-3	-8	-5	9.10	7.40
-3	-8	-5	-4.20	5.20
3	8	5	34.30	9.50

3	8	5	7.10	6.30
−3	8	−5	−5.50	9.60
3	8	5	−1.90	9.50
−3	8	−5	0.00	6.90
−4	−8	−5	63.59	17.10
4	−8	5	90.69	11.00
−4	−8	−5	86.39	14.00
−4	−8	−5	68.29	14.70
4	8	5	44.90	12.80
−4	8	−5	89.29	15.50
−4	8	−5	66.89	5.10
−4	8	−5	79.49	11.20
−4	8	−5	84.69	17.80
4	8	5	91.49	12.90
−5	−8	−5	2.10	6.70
−5	−8	−5	−8.30	9.40
5	−8	5	−1.00	4.90
−5	−8	−5	−21.80	11.10
5	8	5	−2.30	5.70
−5	8	−5	2.00	9.60
−5	8	−5	−7.80	11.10
−5	8	−5	1.30	3.80
−5	8	−5	2.40	7.50
5	8	5	−2.70	8.50
−6	−8	−5	12.60	14.20
−6	−8	−5	25.70	10.50
−6	−8	−5	18.00	13.50
−6	8	−5	28.10	6.10
6	8	5	13.40	7.50
−6	8	−5	14.10	11.90
−6	8	−5	19.40	8.70

-6	8	-5	49.10	19.70
-7	-8	-5	41.30	17.00
-7	-8	-5	14.00	16.30
-7	-8	-5	24.00	9.90
7	8	5	5.80	7.20
-7	8	-5	21.30	15.00
7	8	5	21.40	13.80
-7	8	-5	3.60	15.60
-8	-8	-5	-11.00	17.00
-8	-8	-5	5.90	10.50
-8	-8	-5	1.70	17.20
8	8	5	12.30	8.80
-8	8	-5	-36.20	18.80
-8	8	-5	23.90	8.60
-8	8	-5	5.90	15.10
-9	-8	-5	-19.50	11.50
-9	-8	-5	-12.00	24.00
-9	-8	-5	19.80	21.10
9	8	5	11.40	12.50
-9	8	-5	3.70	12.20
-9	8	-5	-2.80	19.00
-9	8	-5	-14.60	22.30
-9	-9	5	0.10	27.70
9	-9	-5	-3.60	15.90
-9	-9	5	-12.70	22.30
9	9	-5	12.30	12.10
-9	9	5	44.70	26.50
-9	9	5	49.20	26.40
-8	-9	5	32.20	24.10
8	-9	-5	7.60	8.80
8	-9	-5	-2.80	12.90

−8	−9	5	5.70	20.30
−8	9	5	−25.50	14.40
−8	9	5	13.50	20.40
8	9	−5	1.20	9.50
−8	9	5	−24.00	16.30
7	−9	−5	2.20	19.80
7	−9	−5	3.20	7.00
−7	−9	5	−2.50	18.40
−7	−9	5	5.30	16.10
7	9	−5	−0.40	7.70
−7	9	5	20.40	13.10
−7	9	5	−2.90	14.90
−7	9	5	−31.30	18.40
6	−9	−5	14.40	7.10
−6	−9	5	23.30	18.60
6	−9	−5	0.20	10.20
−6	−9	5	−13.80	15.10
−6	−9	5	0.10	15.30
−6	9	5	1.80	10.10
6	9	−5	−8.60	6.60
−6	9	5	5.00	13.90
−6	9	5	−4.80	17.40
5	−9	−5	−14.10	12.60
5	−9	−5	4.60	6.00
5	−9	−5	−2.10	9.80
−5	−9	5	13.60	10.40
5	9	−5	−1.80	5.80
−5	9	5	−9.30	11.50
−5	9	5	6.70	15.80
4	−9	−5	12.30	9.50
−4	−9	5	2.30	9.40

-4	-9	5	-0.20	4.90
4	-9	-5	-6.20	8.30
-4	-9	5	-6.10	7.20
4	9	-5	10.40	11.60
4	9	-5	1.20	5.90
-4	9	5	-20.60	12.60
-4	9	5	-3.30	11.60
3	-9	-5	-1.80	7.30
-3	-9	5	12.30	6.70
-3	-9	5	6.00	9.50
-3	-9	5	13.40	8.10
-3	9	5	-5.10	10.40
3	9	-5	15.20	10.50
-3	9	5	7.80	13.50
3	9	-5	0.20	4.60
2	-9	-5	8.30	5.70
2	-9	-5	-9.30	7.60
-2	-9	5	9.40	7.50
-2	-9	5	-0.90	6.10
2	9	-5	12.00	7.90
2	9	-5	-1.20	4.10
-2	9	5	-4.50	5.90
-2	9	5	-1.30	8.40
-2	9	5	-15.60	10.10
2	9	-5	-6.60	4.00
1	-9	-5	143.39	11.80
1	-9	-5	95.49	16.40
-1	-9	5	104.29	14.90
-1	9	5	105.99	13.90
1	9	-5	112.49	13.10
1	9	-5	127.09	12.40

-1	9	5	121.29	24.50
0	-9	-5	28.50	7.10
0	-9	-5	19.10	9.30
0	-9	-5	19.60	12.00
0	-9	5	26.70	9.60
0	9	-5	31.30	5.00
0	9	5	41.50	9.70
0	9	5	41.40	15.50
-1	-9	-5	59.19	7.70
-1	-9	-5	70.79	12.90
1	-9	5	46.60	9.80
-1	-9	-5	40.10	16.50
-1	9	-5	64.79	14.90
1	9	5	37.30	15.50
1	9	5	43.40	9.80
-1	9	-5	46.10	4.60
-1	9	-5	50.29	12.40
-2	-9	-5	19.90	9.90
2	-9	5	22.40	7.10
-2	-9	-5	41.40	11.80
-2	-9	-5	31.20	7.50
2	9	5	24.70	12.40
-2	9	-5	23.30	9.70
2	9	5	18.90	8.00
-2	9	-5	23.70	12.10
-3	-9	-5	48.10	12.20
-3	-9	-5	70.59	13.20
3	-9	5	62.09	10.70
-3	-9	-5	63.49	16.50
-3	9	-5	52.79	14.60
3	9	5	54.09	12.30

3	9	5	64.79	19.10
3	9	5	63.19	11.70
−4	−9	−5	43.20	13.60
−4	−9	−5	18.10	9.60
−4	−9	−5	28.60	15.70
4	−9	5	32.40	8.20
−4	9	−5	20.70	16.10
4	9	5	33.00	14.90
−4	9	−5	19.00	4.00
4	9	5	18.20	7.80
−4	9	−5	58.19	14.30
−5	−9	−5	2.00	8.10
−5	−9	−5	18.70	14.30
5	−9	5	20.50	7.40
−5	−9	−5	49.50	16.80
5	9	5	25.20	9.50
−5	9	−5	21.40	16.60
−5	9	−5	41.30	12.40
5	9	5	14.90	13.60
−5	9	−5	4.20	4.40
6	−9	5	11.00	6.20
−6	−9	−5	15.00	20.20
−6	−9	−5	32.30	16.00
−6	−9	−5	3.50	8.90
−6	9	−5	8.20	12.00
6	9	5	18.80	9.60
−6	9	−5	24.90	17.20
−6	9	−5	31.60	13.20
−6	9	−5	17.30	7.10
6	9	5	19.40	15.60
−7	−9	−5	−0.70	8.70

-7	-9	-5	9.20	17.50
-7	-9	-5	-18.60	15.70
-7	9	-5	27.50	19.30
7	9	5	-8.50	7.90
-7	9	-5	-13.50	12.80
7	9	5	-1.20	15.50
-8	-9	-5	-9.90	10.50
-8	-9	-5	67.29	27.80
-8	-9	-5	-31.00	20.20
-8	9	-5	32.00	21.50
-8	9	-5	7.80	15.00
8	9	5	-0.40	8.40
-9	-9	-5	19.60	24.90
-9	-9	-5	15.60	12.40
-9	-9	-5	51.69	25.40
9	9	5	-5.30	10.90
-9	9	-5	25.30	27.00
-9	9	-5	-26.30	19.60
-8	-10	5	18.20	23.40
-8	-10	5	4.50	24.60
8	-10	-5	7.90	14.80
-8	10	5	48.80	17.50
-8	10	5	-34.50	23.50
8	10	-5	19.20	11.70
-8	10	5	-17.20	18.60
-7	-10	5	61.59	20.90
7	-10	-5	37.60	13.90
7	-10	-5	10.30	7.70
-7	-10	5	30.40	20.80
-7	10	5	-24.70	13.40
7	10	-5	17.90	11.40

-7	10	5	15.50	20.30
-7	10	5	18.00	17.50
6	-10	-5	15.30	13.00
-6	-10	5	3.00	16.70
6	-10	-5	0.00	6.20
6	10	-5	2.60	7.30
-6	10	5	1.30	15.40
-6	10	5	14.70	16.00
-5	-10	5	50.29	18.60
5	-10	-5	137.49	16.30
5	-10	-5	81.29	9.40
-5	-10	5	124.69	15.60
-5	10	5	89.89	20.70
5	10	-5	89.19	14.10
-5	10	5	56.09	21.80
-4	-10	5	2.10	8.90
-4	-10	5	2.70	5.00
-4	-10	5	1.40	10.90
4	-10	-5	-0.10	11.50
4	-10	-5	-3.00	5.20
4	10	-5	-8.70	12.40
-4	10	5	-4.10	15.30
4	10	-5	6.70	6.50
-4	10	5	12.50	12.50
-3	-10	5	128.39	18.10
3	-10	-5	134.79	20.30
-3	-10	5	145.49	14.60
3	-10	-5	115.49	15.40
-3	-10	5	189.18	22.80
-3	10	5	150.68	16.60
3	10	-5	123.09	18.60

-3	10	5	165.58	27.20
3	10	-5	138.29	12.70
-3	10	5	138.19	29.00
3	10	-5	135.99	11.60
-2	-10	5	1.60	8.30
-2	-10	5	9.60	8.50
2	-10	-5	7.10	8.80
2	-10	-5	5.80	5.60
2	10	-5	8.20	5.90
2	10	-5	5.30	4.50
2	10	-5	6.10	8.40
-2	10	5	5.00	12.90
-2	10	5	-5.90	13.00
-2	10	5	-2.60	6.80
1	-10	-5	14.60	9.50
1	-10	-5	42.40	16.00
-1	-10	5	17.20	8.60
-1	-10	5	-1.70	7.10
1	-10	-5	9.60	6.00
-1	10	5	7.90	7.10
-1	10	5	32.70	15.80
1	10	-5	16.80	8.00
1	10	-5	13.30	5.10
0	-10	5	3.80	6.90
0	-10	5	-4.00	6.00
0	-10	-5	11.90	8.90
0	-10	-5	-1.30	10.70
0	10	-5	9.30	4.20
0	10	5	1.50	6.80
0	10	5	22.50	11.40
0	10	-5	19.20	9.00

-1	-10	-5	-8.60	10.60
1	-10	5	5.70	6.00
-1	-10	-5	9.80	8.80
1	10	5	-1.90	7.10
-1	10	-5	1.40	2.80
1	10	5	4.90	12.60
-1	10	-5	-2.20	10.80
-1	10	-5	8.20	9.30
-2	-10	-5	5.60	12.50
-2	-10	-5	4.30	8.50
2	-10	5	-1.30	6.10
-2	-10	-5	4.00	6.50
2	10	5	16.40	8.30
-2	10	-5	-0.10	8.20
-2	10	-5	4.80	12.40
2	10	5	15.90	14.30
-2	10	-5	9.80	2.80
-3	-10	-5	76.99	15.60
-3	-10	-5	106.39	21.70
-3	-10	-5	130.19	17.50
3	-10	5	113.39	14.60
3	10	5	73.39	18.10
3	10	5	107.19	15.10
-3	10	-5	88.39	21.80
-3	10	-5	110.39	19.10
-4	-10	-5	2.10	15.50
-4	-10	-5	-6.30	8.70
4	-10	5	11.00	7.80
-4	-10	-5	3.30	11.50
-4	10	-5	-17.90	9.40
-4	10	-5	-3.40	14.50

-4	10	-5	9.40	4.00
4	10	5	-5.20	6.00
4	10	5	2.20	12.20
-5	-10	-5	79.49	22.70
5	-10	5	52.29	10.00
-5	-10	-5	32.20	12.80
-5	-10	-5	27.20	15.00
5	10	5	61.99	12.50
-5	10	-5	15.00	15.40
-5	10	-5	33.30	5.90
-5	10	-5	55.29	15.40
5	10	5	55.49	18.10
-6	-10	-5	7.70	9.60
-6	-10	-5	19.80	17.80
6	-10	5	4.30	7.90
-6	-10	-5	-20.30	16.20
-6	10	-5	-8.10	17.50
-6	10	-5	27.20	14.50
6	10	5	23.30	15.40
6	10	5	-5.80	7.10
-7	-10	-5	-2.90	19.60
-7	-10	-5	-8.00	10.00
-7	10	-5	14.20	20.00
-7	10	-5	-19.60	13.70
7	10	5	-19.20	16.00
7	10	5	3.80	9.50
-8	-10	-5	2.60	22.70
-8	-10	-5	-18.60	11.30
-8	-10	-5	25.60	24.40
-8	10	-5	-0.80	23.30
8	10	5	-0.10	11.90

-8	11	5	-14.50	22.50
-8	11	5	7.40	20.30
7	-11	-5	-13.00	13.60
-7	-11	5	-1.70	21.20
-7	11	5	0.50	17.30
7	11	-5	16.90	10.40
-6	-11	5	34.30	21.80
6	-11	-5	5.10	12.80
6	11	-5	15.10	10.00
-6	11	5	20.60	18.60
-6	11	5	28.10	22.40
5	-11	-5	30.50	14.30
5	-11	-5	31.30	6.70
-5	-11	5	33.60	16.50
-5	11	5	49.50	17.50
-5	11	5	57.69	23.10
4	-11	-5	4.90	5.40
4	-11	-5	6.80	10.50
-4	-11	5	27.20	14.10
4	11	-5	6.00	7.40
-4	11	5	1.90	14.90
4	11	-5	25.30	20.60
-4	11	5	20.90	19.50
-3	-11	5	4.30	11.50
3	-11	-5	39.90	7.70
-3	-11	5	19.10	10.10
-3	-11	5	12.10	10.10
3	-11	-5	25.90	11.40
3	11	-5	17.70	7.20
3	11	-5	15.10	12.70
3	11	-5	30.70	7.80

-3	11	5	31.40	18.20
-3	11	5	19.60	8.70
2	-11	-5	14.30	10.80
-2	-11	5	17.70	10.30
-2	-11	5	25.00	11.90
-2	11	5	15.00	16.70
-2	11	5	16.70	8.70
2	11	-5	17.80	10.70
2	11	-5	24.50	7.00
2	11	-5	34.60	9.50
1	-11	-5	131.59	23.90
-1	-11	5	115.59	17.10
1	-11	-5	65.29	14.10
1	11	-5	104.89	15.00
-1	11	5	101.79	31.40
1	11	-5	111.49	11.20
-1	11	5	125.49	16.30
0	-11	-5	70.29	14.30
0	-11	5	31.80	10.70
0	-11	5	35.90	11.30
0	-11	-5	25.40	14.80
0	11	5	44.70	11.70
0	11	-5	37.50	12.80
0	11	5	41.80	17.80
-1	-11	-5	124.39	19.80
1	-11	5	135.09	17.70
-1	-11	-5	117.69	21.80
-1	11	-5	141.59	25.30
-1	11	-5	128.99	19.70
1	11	5	133.59	16.30
-1	11	-5	142.99	8.50

1	11	5	118.69	25.20
-2	-11	-5	-12.50	14.20
-2	-11	-5	-2.70	8.80
2	-11	5	6.10	7.80
-2	11	-5	28.90	12.10
2	11	5	14.10	9.20
-2	11	-5	6.40	9.90
2	11	5	3.80	12.40
-2	11	-5	15.00	4.10
-2	11	-5	12.90	3.70
-3	-11	-5	-20.80	12.90
-3	-11	-5	-3.10	9.20
3	-11	5	4.80	6.70
-3	-11	-5	20.70	15.50
3	11	5	12.40	7.90
-3	11	-5	7.80	13.60
-3	11	-5	-7.40	3.70
-3	11	-5	10.40	10.40
3	11	5	1.00	17.10
-3	11	-5	1.00	3.60
-4	-11	-5	-1.00	11.70
-4	-11	-5	6.40	11.40
-4	-11	-5	17.50	16.00
4	-11	5	6.40	7.70
4	11	5	-2.40	12.00
4	11	5	-4.80	7.60
-4	11	-5	25.60	16.20
-4	11	-5	14.50	11.30
5	-11	5	4.70	8.60
-5	-11	-5	-5.40	14.50
-5	-11	-5	-7.30	9.50

-5	11	-5	-9.90	16.40
5	11	5	11.00	9.80
5	11	5	15.30	16.50
-5	11	-5	-20.00	10.40
-6	-11	-5	14.50	17.20
-6	-11	-5	28.80	21.80
6	-11	5	-4.50	9.80
-6	-11	-5	15.80	11.60
-6	11	-5	8.70	13.80
-6	11	-5	3.30	19.00
6	11	5	-7.90	8.30
6	11	5	1.20	18.10
-7	-11	-5	24.30	22.00
-7	-11	-5	20.40	12.20
-7	-11	-5	-16.20	23.50
7	11	5	-31.10	18.50
-7	11	-5	18.60	21.90
-7	11	-5	19.30	16.90
-7	-12	5	32.50	25.60
7	-12	-5	36.50	16.40
-7	12	5	6.50	18.70
7	12	-5	16.40	11.90
-7	12	5	19.30	25.00
6	-12	-5	1.70	13.60
-6	-12	5	13.90	22.90
6	12	-5	14.10	10.60
-6	12	5	-25.00	17.20
-6	12	5	-9.40	26.40
-5	-12	5	30.40	19.10
5	-12	-5	22.40	12.70
5	12	-5	23.00	10.30

-5	12	5	15.70	17.80
-5	12	5	22.50	20.40
-4	-12	5	6.40	16.30
4	-12	-5	20.60	13.10
-4	-12	5	5.40	11.00
4	12	-5	7.00	8.90
-4	12	5	4.50	11.00
-4	12	5	-9.60	15.40
-4	12	5	-13.70	20.50
-3	-12	5	5.30	9.40
3	-12	-5	9.50	10.80
-3	-12	5	11.80	13.40
-3	12	5	24.10	16.50
3	12	-5	7.50	8.00
-3	12	5	1.40	8.50
2	-12	-5	28.00	12.70
2	-12	-5	40.60	18.20
-2	-12	5	15.70	10.00
-2	-12	5	13.00	11.80
-2	12	5	30.20	10.40
2	12	-5	25.90	8.20
2	12	-5	20.00	8.80
-2	12	5	54.49	22.40
-1	-12	5	-3.80	7.70
1	-12	-5	-8.30	8.60
-1	12	5	17.50	8.40
1	12	-5	-8.90	9.30
1	12	-5	-1.50	5.70
-1	12	5	45.20	21.20
0	-12	5	18.70	10.30
0	-12	5	11.90	9.70

0	-12	-5	14.40	10.70
0	-12	-5	37.40	18.50
0	12	5	11.50	9.80
0	12	-5	12.80	10.90
0	12	5	10.40	15.70
0	12	-5	2.20	5.10
0	12	-5	16.20	12.50
1	-12	5	5.20	8.10
-1	-12	-5	-10.90	10.00
-1	-12	-5	-1.60	17.00
1	12	5	17.80	17.70
-1	12	-5	2.70	9.80
-1	12	-5	-3.30	4.00
-1	12	-5	-9.30	13.50
-1	12	-5	-11.10	5.20
-2	-12	-5	8.90	17.20
2	-12	5	34.50	10.50
-2	-12	-5	20.80	13.10
-2	12	-5	2.50	10.80
-2	12	-5	-7.30	15.00
2	12	5	25.60	10.70
2	12	5	21.90	18.80
-2	12	-5	14.40	4.30
-3	-12	-5	4.30	17.00
-3	-12	-5	-6.60	9.40
3	-12	5	6.80	9.40
-3	-12	-5	-1.80	8.90
3	12	5	-9.40	14.10
-3	12	-5	12.90	4.10
3	12	5	5.90	9.10
-3	12	-5	4.30	17.10

-4	-12	-5	2.80	13.80
-4	-12	-5	17.00	12.20
4	-12	5	28.30	9.30
-4	-12	-5	19.20	20.80
-4	12	-5	18.70	13.60
4	12	5	2.90	13.70
-4	12	-5	10.90	18.10
4	12	5	12.80	10.60
5	-12	5	2.60	9.70
-5	-12	-5	42.40	18.50
-5	-12	-5	-3.40	11.30
-5	-12	-5	-37.90	19.60
-5	12	-5	15.00	12.80
-5	12	-5	31.00	20.10
5	12	5	-1.90	8.70
5	12	5	13.90	16.60
-6	-12	-5	1.60	22.20
6	-12	5	4.70	11.10
-6	-12	-5	1.80	12.90
-6	-12	-5	-17.10	22.00
6	12	5	-26.90	16.60
-6	12	-5	-23.90	14.60
-6	12	-5	27.30	23.40
6	12	5	3.30	10.30
-7	-12	-5	-20.70	25.40
-7	-12	-5	40.00	25.30
-7	12	-5	-15.10	25.00
6	-13	-5	10.30	16.00
6	13	-5	3.30	10.80
-6	13	5	15.00	23.60
-6	13	5	-35.30	18.00

5	-13	-5	-12.00	14.20
5	13	-5	-2.30	9.80
-5	13	5	0.90	10.80
4	-13	-5	11.90	16.20
-4	-13	5	-3.50	11.90
-4	13	5	-6.30	21.80
-4	13	5	-0.70	11.50
4	13	-5	8.80	10.00
-3	-13	5	20.90	10.50
3	-13	-5	13.00	22.20
3	-13	-5	3.50	14.30
3	13	-5	19.50	9.60
-3	13	5	9.50	10.70
-3	13	5	0.60	23.20
3	13	-5	4.60	8.00
2	-13	-5	-9.80	11.60
-2	-13	5	24.20	12.30
-2	13	5	8.70	10.70
2	13	-5	14.00	10.10
2	13	-5	-6.10	6.80
-2	13	5	-28.30	17.60
-1	-13	5	15.10	11.70
1	-13	-5	22.20	21.50
1	-13	-5	25.10	13.90
1	13	-5	31.20	8.40
-1	13	5	30.40	19.40
1	13	-5	6.60	11.40
-1	13	5	45.50	12.70
0	-13	-5	-2.70	10.60
0	-13	-5	-10.70	19.20
0	-13	5	28.60	12.40

0	13	-5	-2.80	11.50
0	13	-5	-5.20	5.10
0	13	5	-19.00	18.10
0	13	5	-6.40	9.70
0	13	-5	-12.90	12.70
-1	-13	-5	12.60	12.50
1	-13	5	10.40	9.80
-1	-13	-5	11.80	18.00
-1	13	-5	1.60	5.40
-1	13	-5	-1.50	11.20
1	13	5	7.60	16.60
1	13	5	16.70	11.20
-1	13	-5	14.00	16.60
2	-13	5	29.20	11.20
-2	-13	-5	3.00	12.00
-2	-13	-5	5.20	19.50
-2	13	-5	1.00	5.00
-2	13	-5	8.00	18.90
-2	13	-5	20.60	11.90
2	13	5	23.90	9.70
2	13	5	24.90	18.00
-3	-13	-5	-5.10	12.60
3	-13	5	6.30	10.30
-3	-13	-5	21.70	21.50
-3	13	-5	-51.99	18.40
3	13	5	3.00	19.30
3	13	5	-2.80	8.40
-3	13	-5	35.30	13.30
-4	-13	-5	3.00	14.60
4	13	5	-9.00	9.70
4	13	5	-5.50	17.80

-4	13	-5	6.30	12.30
-4	13	-5	47.20	21.10
-5	-13	-5	11.40	13.60
5	-13	5	3.90	12.30
-5	-13	-5	3.80	24.20
-5	13	-5	-31.60	13.10
-5	13	-5	-19.30	22.70
5	13	5	7.50	19.70
5	13	5	1.70	12.90
-4	-14	5	-10.70	11.90
4	-14	-5	2.30	16.60
4	14	-5	12.90	11.20
-4	14	5	-7.50	11.40
-4	14	5	21.30	24.50
3	-14	-5	12.70	14.70
-3	14	5	20.40	13.00
3	14	-5	26.00	10.10
-3	14	5	45.40	25.00
-2	-14	5	9.40	12.10
2	-14	-5	6.90	15.90
2	-14	-5	14.90	22.90
2	14	-5	18.70	9.70
-2	14	5	0.80	11.50
2	14	-5	-32.90	11.30
-2	14	5	-6.70	19.60
-1	-14	5	14.60	13.40
1	-14	-5	8.20	15.30
1	-14	-5	48.20	25.60
-1	14	5	23.50	12.70
-1	14	5	3.60	19.20
1	14	-5	36.80	14.70

1	14	-5	9.00	8.00
0	-14	-5	13.00	21.70
0	-14	-5	11.20	15.20
0	-14	5	-13.00	11.40
0	14	5	39.60	20.70
0	14	5	1.10	10.40
0	14	-5	-7.40	15.40
0	14	-5	4.10	7.10
0	14	-5	24.70	13.30
-1	-14	-5	-14.90	22.30
-1	-14	-5	3.00	12.80
1	-14	5	3.00	11.70
-1	14	-5	18.80	19.20
-1	14	-5	-10.40	6.70
1	14	5	5.40	10.80
1	14	5	-2.80	20.60
2	-14	5	15.50	12.20
-2	-14	-5	-28.00	21.10
-2	-14	-5	3.70	14.20
2	14	5	2.70	10.30
-2	14	-5	-21.90	19.10
2	14	5	24.10	19.30
3	-14	5	27.20	12.80
-3	-14	-5	17.90	25.40
3	14	5	28.60	11.70
-3	14	-5	53.49	24.10
3	14	5	25.30	23.30
4	-14	5	31.70	14.70
-4	-14	-5	-18.40	14.60
-4	-14	-5	-28.10	25.30
4	14	5	-27.10	11.40

4	14	5	-31.20	21.60
-4	14	-5	-37.50	25.40
2	-15	-5	-17.10	14.70
-2	-15	5	-16.00	13.60
2	15	-5	6.30	10.30
-2	15	5	69.69	31.70
-2	15	5	11.10	14.70
1	-15	-5	31.00	19.30
-1	-15	5	-7.30	13.40
1	15	-5	15.70	10.20
-1	15	5	14.00	13.60
0	-15	5	3.90	13.20
0	-15	-5	-15.50	13.50
0	15	5	39.90	27.70
0	15	-5	48.50	19.30
0	15	5	2.70	12.50
-1	-15	-5	-22.90	26.10
-1	-15	-5	13.90	15.80
1	-15	5	14.80	14.40
-1	15	-5	50.79	23.50
1	15	5	-25.00	23.30
1	15	5	-2.90	12.80
-2	-15	-5	-13.40	15.80
-2	-15	-5	-30.30	29.40
2	-15	5	3.00	14.40
2	15	5	-55.29	22.60
2	15	5	0.90	11.50
-2	15	-5	7.30	27.20
-11	0	6	28.80	22.90
-11	0	6	-0.10	20.80
-11	0	6	10.20	19.80

11	0	−6	−18.10	15.50
−10	0	6	26.60	18.70
10	0	−6	−3.90	8.70
−10	0	6	−31.20	14.90
10	0	−6	−4.70	13.30
−10	0	6	21.70	17.10
9	0	−6	6.40	8.50
−9	0	6	−27.00	14.20
−9	0	6	−9.70	15.90
−9	0	6	20.30	15.10
8	0	−6	−2.50	7.50
−8	0	6	3.70	11.30
−8	0	6	2.10	23.00
7	0	−6	−5.60	4.90
−7	0	6	−4.10	10.50
−7	0	6	−4.10	10.40
−7	0	6	−2.70	9.50
6	0	−6	736.63	29.90
−6	0	6	664.93	39.40
−6	0	6	769.62	44.80
−6	0	6	695.93	42.20
−5	0	6	−0.90	7.30
5	0	−6	2.30	4.30
−5	0	6	7.80	8.60
−5	0	6	1.50	6.40
4	0	−6	962.80	32.90
−4	0	6	975.20	45.00
−3	0	6	−2.60	5.50
3	0	−6	−6.50	3.20
−3	0	6	3.70	4.40
3	0	−6	1.00	6.80

3	0	-6	8.70	8.10
-3	0	6	0.30	3.50
3	0	-6	-2.50	5.10
-2	0	6	1308.17	37.60
2	0	-6	1265.67	45.20
2	0	-6	1322.37	38.70
2	0	-6	1387.36	50.79
-2	0	6	1345.57	44.50
2	0	-6	1409.96	52.89
1	0	-6	-3.20	3.90
-1	0	6	-4.70	2.80
-1	0	6	-7.50	4.70
1	0	-6	3.40	5.80
-1	0	6	-7.90	2.40
1	0	-6	0.80	2.90
0	0	-6	1046.90	32.30
0	0	6	1078.69	30.70
0	0	-6	1134.59	42.90
0	0	6	1042.90	35.00
0	0	6	1052.49	42.30
0	0	6	1057.09	30.10
0	0	-6	1083.09	35.90
-1	0	-6	2.30	5.30
1	0	6	2.90	2.20
1	0	6	-2.00	3.00
-1	0	-6	1.60	5.90
-1	0	-6	-2.50	2.00
1	0	6	3.90	3.60
-1	0	-6	0.20	2.90
-1	0	-6	-1.90	2.60
1	0	6	0.30	4.20

−1	0	−6	−1.90	3.50
−2	0	−6	160.78	11.40
−2	0	−6	152.78	13.90
2	0	6	160.28	10.00
−2	0	−6	165.38	17.00
−2	0	−6	163.88	10.50
−2	0	−6	149.39	15.20
2	0	6	173.88	8.80
2	0	6	175.88	14.60
−3	0	−6	−0.70	2.50
−3	0	−6	5.60	5.60
−3	0	−6	−0.40	3.20
−3	0	−6	3.00	5.80
−3	0	−6	0.00	5.70
3	0	6	−3.30	3.40
−3	0	−6	−0.50	2.80
−3	0	−6	−1.90	6.50
−4	0	−6	527.55	32.50
−4	0	−6	491.05	19.90
4	0	6	520.15	19.50
−4	0	−6	480.15	15.10
−4	0	−6	449.26	28.10
4	0	6	491.75	17.80
−4	0	−6	441.16	26.60
−5	0	−6	−8.50	5.40
−5	0	−6	7.10	7.10
−5	0	−6	−2.70	1.40
5	0	6	0.10	4.40
5	0	6	−1.30	2.80
−6	0	−6	160.08	24.00
−6	0	−6	235.78	21.90

7	0	6	1.00	4.90
−7	0	−6	−3.20	8.70
−7	0	−6	−2.60	8.10
−8	0	−6	290.07	32.90
−8	0	−6	188.58	29.60
8	0	6	266.57	16.30
−9	0	−6	−17.90	11.90
9	0	6	1.20	7.20
−9	0	−6	−9.10	15.00
10	0	6	4.60	8.80
−10	0	−6	7.30	21.40
−10	0	−6	29.80	17.40
−11	0	−6	10.10	21.00
11	0	6	19.20	12.00
−11	0	−6	−41.60	20.70
11	−1	−6	7.60	12.70
−11	−1	6	11.70	21.80
−11	−1	6	−57.19	18.00
−11	−1	6	9.60	20.90
11	−1	−6	−18.10	15.10
−11	1	6	3.50	23.20
−11	1	6	8.80	21.10
11	1	−6	6.30	11.00
11	1	−6	24.50	16.60
−11	1	6	29.90	22.70
−10	−1	6	51.19	27.80
−10	−1	6	48.40	20.60
10	−1	−6	52.39	15.50
−10	−1	6	49.30	18.20
10	−1	−6	12.00	10.30
10	1	−6	19.60	10.00

-10	1	6	63.39	26.30
-10	1	6	45.80	25.40
-10	1	6	64.49	22.50
-9	-1	6	-1.80	14.80
-9	-1	6	24.30	16.30
9	-1	-6	5.00	7.80
9	-1	-6	13.30	10.10
-9	-1	6	-1.50	16.30
-9	1	6	11.20	15.30
-9	1	6	6.00	17.10
9	1	-6	-2.10	7.40
-9	1	6	-1.10	13.80
-8	-1	6	32.70	18.90
-8	-1	6	17.10	13.30
-8	-1	6	17.50	14.60
8	-1	-6	14.10	11.50
-8	1	6	46.80	22.00
-8	1	6	8.70	14.50
-8	1	6	19.50	14.40
8	1	-6	27.00	9.40
-7	-1	6	59.39	15.40
-7	-1	6	24.40	14.10
-7	-1	6	79.99	21.70
7	-1	-6	60.79	14.20
7	-1	-6	43.60	10.40
-7	1	6	44.10	14.40
-7	1	6	49.10	15.40
-7	1	6	57.59	14.90
7	1	-6	53.09	9.70
-6	-1	6	215.48	20.60
-6	-1	6	195.88	25.20

6	−1	−6	182.08	15.10
−6	−1	6	211.78	28.90
−6	1	6	215.98	27.60
−6	1	6	227.78	25.20
6	1	−6	174.68	14.00
−6	1	6	230.18	23.50
−5	−1	6	27.00	9.60
−5	−1	6	30.50	12.00
5	−1	−6	9.70	5.50
−5	−1	6	26.50	12.40
−5	1	6	36.00	11.90
−5	1	6	19.40	10.60
5	1	−6	24.00	6.20
−5	1	6	27.80	11.30
−4	−1	6	51.89	14.00
−4	−1	6	43.10	11.40
−4	−1	6	46.90	10.20
4	−1	−6	40.20	11.10
4	−1	−6	42.80	7.60
−4	1	6	51.49	12.80
−4	1	6	44.90	12.60
4	1	−6	50.49	8.10
−4	1	6	63.59	13.30
4	1	−6	41.80	13.30
−3	−1	6	25.70	5.20
3	−1	−6	31.30	10.90
−3	−1	6	12.20	5.20
3	−1	−6	31.90	6.60
3	−1	−6	22.80	8.80
3	−1	−6	26.50	10.30
−3	−1	6	21.50	8.90

−3	1	6	22.90	7.30
3	1	−6	17.90	5.10
2	−1	−6	562.24	29.80
2	−1	−6	552.04	36.00
−2	−1	6	650.03	31.30
2	−1	−6	613.54	32.10
2	−1	−6	588.64	26.80
−2	−1	6	582.34	22.90
−2	−1	6	597.34	20.40
2	−1	−6	583.84	22.80
2	1	−6	545.15	24.10
2	1	−6	543.55	31.70
2	1	−6	606.94	21.60
−2	1	6	619.64	32.70
−2	1	6	571.44	21.70
2	1	−6	610.14	31.00
−2	1	6	628.64	29.70
−1	−1	6	69.69	6.20
−1	−1	6	68.49	7.10
1	−1	−6	94.69	13.50
1	−1	−6	78.79	13.30
1	−1	−6	77.39	10.90
−1	−1	6	70.39	11.70
1	−1	−6	67.39	8.40
1	1	−6	78.09	7.10
1	1	−6	92.89	11.00
−1	1	6	88.19	11.80
−1	1	6	124.09	16.80
−1	1	6	77.59	8.30
1	1	−6	85.19	13.50
0	−1	−6	578.54	26.60

0	−1	−6	594.74	23.10
0	−1	6	594.14	22.50
0	−1	6	605.74	18.80
0	−1	−6	577.44	30.20
0	−1	6	653.93	30.10
0	−1	6	600.84	19.00
0	1	−6	635.44	21.40
0	1	6	556.24	25.60
0	1	−6	619.54	23.10
0	1	−6	668.43	29.80
0	1	6	627.84	22.30
0	1	6	622.54	23.60
0	1	6	618.24	31.70
1	−1	6	21.10	5.30
−1	−1	−6	21.70	7.70
−1	−1	−6	15.40	6.20
1	−1	6	16.80	3.10
−1	−1	−6	29.70	5.40
−1	−1	−6	20.10	4.50
−1	−1	−6	17.60	5.30
1	−1	6	11.30	5.80
−1	−1	−6	5.50	4.70
1	−1	6	20.50	3.70
−1	1	−6	17.40	4.30
−1	1	−6	19.50	5.60
1	1	6	20.40	4.80
−1	1	−6	16.00	6.50
1	1	6	22.70	7.80
1	1	6	21.20	4.50
−1	1	−6	13.10	3.20
−2	−1	−6	2313.97	67.49

2	-1	6	2427.16	55.89
-2	-1	-6	2241.78	61.09
2	-1	6	2341.67	53.59
-2	-1	-6	2483.15	70.89
-2	-1	-6	2323.27	57.29
-2	-1	-6	2361.16	70.69
-2	-1	-6	2347.67	59.89
-2	-1	-6	2443.06	74.99
2	1	6	2307.67	59.09
-2	1	-6	2467.35	65.79
-2	1	-6	2221.38	74.39
-2	1	-6	2300.07	58.29
2	1	6	2355.06	57.09
-2	1	-6	2348.17	55.59
2	1	6	2313.87	68.59
-2	1	-6	2340.77	59.39
-2	1	-6	2282.37	67.09
-2	1	-6	2375.16	65.79
3	-1	6	0.80	1.70
-3	-1	-6	-0.90	2.80
-3	-1	-6	5.10	3.50
-3	-1	-6	4.40	6.20
-3	-1	-6	2.50	5.80
-3	-1	-6	2.00	3.50
-3	-1	-6	8.40	6.80
3	-1	6	-1.70	3.00
-3	1	-6	-0.70	4.10
-3	1	-6	1.40	2.50
-3	1	-6	5.30	4.80
3	1	6	3.90	4.80
3	1	6	2.00	3.90

−3	1	−6	−3.60	5.00
−3	1	−6	1.10	6.20
3	1	6	2.80	2.70
4	−1	6	748.53	24.10
4	−1	6	774.52	22.20
−4	−1	−6	809.22	37.60
−4	−1	−6	738.63	22.90
−4	−1	−6	726.43	38.70
−4	−1	−6	763.52	27.20
−4	−1	−6	844.82	37.60
−4	1	−6	810.12	33.40
−4	1	−6	771.12	40.20
4	1	6	710.73	24.90
−4	1	−6	725.23	33.50
4	1	6	806.32	25.40
−4	1	−6	748.43	18.90
4	1	6	764.82	26.40
−4	1	−6	770.22	25.60
−4	1	−6	788.52	24.70
−5	−1	−6	16.90	3.70
5	−1	6	21.60	4.50
−5	−1	−6	17.70	9.40
−5	−1	−6	32.60	11.30
−5	−1	−6	13.20	4.70
5	−1	6	24.90	5.40
5	1	6	16.60	5.10
−5	1	−6	13.80	8.20
−5	1	−6	14.00	5.00
−5	1	−6	11.70	8.50
−6	−1	−6	186.58	8.50
−6	−1	−6	149.88	20.60

-6	-1	-6	141.19	22.70
6	-1	6	194.18	11.30
-6	1	-6	251.37	31.60
-6	1	-6	197.68	19.70
6	1	6	197.68	12.70
7	-1	6	5.10	7.50
-7	-1	-6	8.10	9.60
-7	-1	-6	1.40	10.10
7	-1	6	11.80	7.00
-7	1	-6	-0.20	12.10
-7	1	-6	3.80	9.10
7	1	6	12.00	5.60
8	-1	6	6.00	6.30
-8	-1	-6	-14.20	9.10
-8	-1	-6	-5.70	11.70
-8	1	-6	15.90	11.20
8	1	6	-0.60	6.50
9	-1	6	-5.00	6.80
-9	-1	-6	-0.50	13.80
-9	-1	-6	-10.50	12.70
9	1	6	8.30	7.00
-9	1	-6	-20.60	14.50
-9	1	-6	5.50	13.20
-10	-1	-6	1.90	18.70
10	-1	6	7.70	8.60
-10	-1	-6	8.90	17.00
-10	1	-6	5.20	19.20
-10	1	-6	-1.80	14.80
10	1	6	-4.50	8.90
11	-1	6	14.80	11.40
-11	-1	-6	25.90	20.60

−11	−1	−6	−13.40	23.50
−11	1	−6	41.60	20.90
11	1	6	2.30	11.60
−11	1	−6	−12.10	18.70
−11	−2	6	−13.80	22.50
11	−2	−6	−6.90	11.80
11	−2	−6	0.90	16.20
−11	−2	6	11.50	21.90
−11	−2	6	40.70	21.80
11	2	−6	−0.90	11.40
−11	2	6	−21.10	19.00
−11	2	6	18.30	20.00
−11	2	6	−13.90	21.50
10	−2	−6	2.80	9.60
−10	−2	6	20.60	17.60
10	−2	−6	0.90	13.10
−10	−2	6	13.50	20.80
10	2	−6	11.10	11.50
−10	2	6	1.30	21.10
−10	2	6	32.40	18.80
−10	2	6	−38.60	17.60
9	−2	−6	4.80	7.40
−9	−2	6	0.40	15.60
9	−2	−6	4.30	10.30
−9	−2	6	−3.20	15.40
−9	−2	6	−18.90	12.60
9	2	−6	6.30	8.50
−9	2	6	−9.40	15.50
−9	2	6	5.40	14.70
−9	2	6	−15.00	15.00
−8	−2	6	−1.00	11.60

8	−2	−6	3.50	10.10
−8	−2	6	7.80	12.30
8	−2	−6	10.50	7.10
−8	−2	6	8.00	14.70
−8	2	6	2.30	11.70
−8	2	6	8.40	11.40
−8	2	6	2.80	12.10
8	2	−6	−5.80	5.70
7	−2	−6	3.40	5.70
−7	−2	6	−1.00	10.80
−7	−2	6	10.20	15.70
7	−2	−6	3.70	8.10
−7	−2	6	4.60	12.80
−7	2	6	7.20	13.60
7	2	−6	1.00	5.40
−7	2	6	5.20	9.30
−7	2	6	−14.20	10.40
−6	−2	6	291.57	36.30
6	−2	−6	304.67	23.20
−6	−2	6	247.88	20.80
−6	−2	6	250.87	29.10
6	−2	−6	213.18	16.60
6	2	−6	250.57	16.70
−6	2	6	247.18	25.30
−6	2	6	240.28	24.80
−6	2	6	228.68	28.10
−5	−2	6	4.80	9.90
5	−2	−6	0.90	6.30
−5	−2	6	−10.60	7.00
5	−2	−6	5.50	4.70
−5	−2	6	3.10	7.40

−5	2	6	4.70	9.90
−5	2	6	8.80	9.50
−5	2	6	−4.10	7.40
5	2	−6	8.70	4.90
−4	−2	6	36.30	11.50
4	−2	−6	30.10	7.50
4	−2	−6	22.10	7.90
4	−2	−6	31.90	12.70
−4	−2	6	10.40	5.20
−4	−2	6	34.90	10.80
4	2	−6	15.80	5.30
−4	2	6	32.50	11.10
4	2	−6	13.70	9.10
−4	2	6	11.20	8.50
−4	2	6	22.00	9.70
3	−2	−6	12.50	6.20
−3	−2	6	12.80	5.50
3	−2	−6	16.90	9.60
−3	−2	6	15.30	9.10
3	−2	−6	10.00	8.30
3	−2	−6	22.40	6.40
−3	−2	6	16.60	4.70
−3	2	6	13.00	7.90
−3	2	6	16.10	8.90
3	2	−6	11.30	4.20
3	2	−6	12.00	7.30
2	−2	−6	2501.35	92.89
2	−2	−6	2868.61	72.69
2	−2	−6	2977.30	88.29
−2	−2	6	2818.12	66.09
−2	−2	6	2834.42	66.99

-2	-2	6	2822.72	81.29
2	-2	-6	2969.10	80.59
-2	2	6	2791.12	85.09
2	2	-6	2821.42	68.99
2	2	-6	2747.63	80.39
-2	2	6	2892.51	72.19
2	2	-6	2918.21	89.99
-2	2	6	2972.10	88.79
1	-2	-6	2.90	7.30
-1	-2	6	-5.10	3.90
-1	-2	6	1.30	2.40
-1	-2	6	3.00	2.70
1	-2	-6	-1.60	7.90
1	-2	-6	4.90	3.40
1	-2	-6	2.40	4.50
-1	-2	6	0.10	1.90
1	2	-6	9.90	5.80
1	2	-6	-1.00	3.30
-1	2	6	0.80	5.50
1	2	-6	-0.70	2.50
-1	2	6	6.10	3.60
-1	2	6	2.20	3.20
0	-2	6	350.16	17.20
0	-2	6	373.96	11.90
0	-2	6	369.96	21.30
0	-2	-6	321.67	23.40
0	-2	-6	328.77	16.50
0	-2	-6	339.37	21.10
0	-2	-6	319.87	19.10
0	-2	6	349.27	11.70
0	2	6	367.26	16.10

0	2	−6	381.66	20.50
0	2	−6	362.96	15.50
0	2	−6	338.87	13.80
0	2	6	346.37	16.10
0	2	6	308.17	20.30
−1	−2	−6	6.30	3.60
−1	−2	−6	4.60	6.80
1	−2	6	9.90	4.40
1	−2	6	7.90	1.70
−1	−2	−6	0.90	3.00
−1	−2	−6	6.90	5.60
−1	−2	−6	15.20	6.30
1	2	6	9.70	5.00
−1	2	−6	13.90	4.00
−1	2	−6	7.10	2.70
−1	2	−6	8.00	5.30
1	2	6	8.20	4.40
1	2	6	13.10	4.00
1	2	6	4.60	5.10
−1	2	−6	6.00	4.10
−2	−2	−6	282.87	21.10
2	−2	6	278.67	8.90
−2	−2	−6	305.97	15.30
−2	−2	−6	278.57	15.50
−2	−2	−6	283.47	19.50
−2	−2	−6	284.37	13.40
−2	−2	−6	301.97	21.70
−2	−2	−6	273.07	20.10
2	−2	6	295.07	8.50
−2	2	−6	292.77	17.30
2	2	6	291.27	20.00

2	2	6	299.97	14.00
2	2	6	281.67	16.40
−2	2	−6	301.07	11.40
2	2	6	282.67	14.20
−2	2	−6	264.07	17.90
−2	2	−6	248.38	15.10
−2	2	−6	287.77	22.60
−2	2	−6	265.97	12.50
−3	−2	−6	1.50	3.20
3	−2	6	2.40	1.90
−3	−2	−6	−2.10	5.60
−3	−2	−6	1.00	5.40
3	−2	6	−0.90	1.30
−3	−2	−6	4.40	3.20
−3	2	−6	−6.80	4.70
−3	2	−6	−1.30	5.20
−3	2	−6	1.30	2.50
3	2	6	−2.90	4.90
−3	2	−6	0.30	2.70
3	2	6	−0.40	4.00
3	2	6	6.20	3.70
3	2	6	−5.00	4.20
−3	2	−6	11.00	7.00
−3	2	−6	0.60	2.30
−3	2	−6	−1.30	3.90
−3	2	−6	−1.50	2.50
−4	−2	−6	25.30	6.00
−4	−2	−6	42.70	11.00
4	−2	6	39.60	4.80
−4	−2	−6	42.40	6.20
−4	−2	−6	21.80	9.60

−4	−2	−6	38.10	11.40
4	−2	6	44.70	4.30
−4	2	−6	46.70	9.00
4	2	6	55.89	7.90
−4	2	−6	41.20	5.60
−4	2	−6	37.30	9.70
−4	2	−6	38.80	12.00
−4	2	−6	50.49	7.20
4	2	6	45.80	7.30
4	2	6	32.60	10.60
−5	−2	−6	10.60	7.50
5	−2	6	7.50	3.90
−5	−2	−6	14.60	6.40
−5	−2	−6	3.60	8.00
5	−2	6	8.00	3.10
−5	−2	−6	4.60	3.60
5	2	6	17.30	5.70
−5	2	−6	7.60	6.30
−5	2	−6	12.10	7.50
5	2	6	17.40	4.80
−5	2	−6	23.40	10.10
−5	2	−6	12.30	4.60
−6	−2	−6	87.99	17.20
−6	−2	−6	125.29	21.80
6	−2	6	124.29	9.70
−6	−2	−6	106.69	8.50
−6	2	−6	125.29	16.20
−6	2	−6	102.09	20.60
6	2	6	134.19	11.70
6	2	6	105.09	11.50
7	−2	6	3.90	4.20

−7	−2	−6	13.70	10.70
−7	−2	−6	−8.20	10.50
7	−2	6	15.30	6.20
−7	2	−6	−6.80	8.30
7	2	6	6.40	5.10
−7	2	−6	−6.50	11.40
7	2	6	−4.10	5.90
−8	−2	−6	70.19	17.70
−8	−2	−6	86.29	19.90
8	−2	6	79.49	9.20
−8	2	−6	65.59	17.60
8	2	6	114.39	14.00
8	2	6	92.29	9.10
−8	2	−6	94.79	24.70
−9	−2	−6	17.40	15.00
−9	−2	−6	9.70	14.30
9	2	6	7.30	7.50
−9	2	−6	−17.80	11.20
−9	2	−6	−6.10	13.70
−10	−2	−6	30.60	20.40
−10	−2	−6	25.30	19.10
10	−2	6	39.40	9.50
−10	2	−6	32.00	22.20
−10	2	−6	26.70	18.00
10	2	6	1.20	10.20
−11	−3	6	−4.60	22.30
−11	3	6	5.40	25.30
−10	−3	6	−9.70	17.00
10	−3	−6	43.10	13.00
−10	−3	6	68.49	26.70
10	−3	−6	29.30	13.90

-10	-3	6	-0.40	20.00
-10	3	6	-3.20	16.70
-10	3	6	2.30	21.00
-10	3	6	34.00	19.40
10	3	-6	23.30	11.20
9	-3	-6	-4.30	8.40
-9	-3	6	-22.90	14.00
-9	-3	6	-0.70	17.20
9	-3	-6	-5.60	11.00
-9	-3	6	5.50	12.70
-9	3	6	6.10	15.90
-9	3	6	-16.00	14.10
-9	3	6	-0.80	16.60
9	3	-6	-10.40	8.00
-8	-3	6	-10.40	13.30
8	-3	-6	9.00	10.50
-8	-3	6	-2.00	10.70
-8	-3	6	-10.00	13.40
8	-3	-6	13.50	8.50
8	3	-6	2.10	6.50
-8	3	6	21.30	14.30
-8	3	6	-8.10	12.40
-8	3	6	17.90	14.50
-7	-3	6	30.40	16.00
7	-3	-6	24.00	11.10
7	-3	-6	-2.40	6.60
-7	-3	6	9.10	10.40
-7	-3	6	16.40	14.90
-7	3	6	10.20	11.60
-7	3	6	2.50	11.90
7	3	-6	17.60	8.20

−7	3	6	33.10	14.60
−6	−3	6	232.38	32.70
−6	−3	6	198.28	27.30
6	−3	−6	199.78	20.30
6	−3	−6	190.88	16.00
−6	−3	6	181.38	17.50
−6	3	6	167.28	25.70
6	3	−6	165.28	14.20
−6	3	6	226.48	24.10
−6	3	6	170.68	26.50
−5	−3	6	40.40	8.80
−5	−3	6	70.09	17.90
5	−3	−6	21.70	8.30
5	−3	−6	43.20	9.00
−5	−3	6	30.10	13.10
−5	3	6	32.50	13.20
5	3	−6	47.10	8.50
−5	3	6	48.20	14.60
−5	3	6	44.50	15.80
4	−3	−6	7.30	6.10
−4	−3	6	11.00	8.00
−4	−3	6	1.60	4.60
−4	−3	6	18.80	10.20
4	−3	−6	13.30	5.30
−4	3	6	4.90	8.60
4	3	−6	6.90	8.70
4	3	−6	2.40	3.40
−4	3	6	8.40	9.70
−4	3	6	6.00	7.40
3	−3	−6	17.30	6.00
3	−3	−6	22.60	8.20

−3	−3	6	9.00	3.70
−3	−3	6	22.60	3.70
3	−3	−6	25.30	11.30
3	−3	−6	10.30	8.20
3	3	−6	23.50	5.50
−3	3	6	10.10	8.70
3	3	−6	15.30	7.50
−3	3	6	8.80	8.40
2	−3	−6	232.98	14.90
−2	−3	6	259.87	11.00
−2	−3	6	232.38	18.10
2	−3	−6	258.77	19.70
2	−3	−6	235.98	23.00
2	−3	−6	270.27	23.90
2	−3	−6	204.78	17.40
−2	3	6	240.68	21.80
2	3	−6	244.68	18.90
2	3	−6	234.18	31.20
2	3	−6	289.07	13.30
−2	3	6	240.98	22.40
−1	−3	6	337.97	11.70
−1	−3	6	329.77	12.50
1	−3	−6	360.56	25.30
−1	−3	6	422.76	22.30
1	−3	−6	335.17	24.10
1	−3	−6	340.07	20.80
1	−3	−6	354.76	17.50
1	3	−6	339.07	13.80
−1	3	6	341.87	17.20
−1	3	6	325.37	24.90
−1	3	6	351.96	24.80

1	3	−6	330.67	19.30
1	3	−6	343.67	12.40
−1	3	6	380.46	21.90
−1	3	6	339.77	17.00
0	−3	6	515.35	13.10
0	−3	6	519.45	13.20
0	−3	−6	549.65	22.30
0	−3	−6	533.75	28.50
0	−3	6	503.55	24.30
0	−3	−6	453.75	23.80
0	−3	−6	506.85	29.90
0	3	6	511.15	21.00
0	3	6	528.95	30.90
0	3	−6	510.95	17.00
0	3	−6	544.55	21.70
0	3	−6	528.05	22.80
0	3	−6	495.95	17.90
0	3	6	505.05	28.10
0	3	6	550.44	25.30
0	3	6	495.25	20.70
−1	−3	−6	244.08	20.20
−1	−3	−6	196.98	17.20
−1	−3	−6	214.98	11.80
1	−3	6	176.08	13.40
−1	−3	−6	197.58	12.90
−1	−3	−6	231.78	15.90
1	3	6	239.88	13.60
−1	3	−6	242.28	9.40
1	3	6	212.88	17.70
−1	3	−6	219.38	13.00
1	3	6	226.28	20.40

−1	3	−6	214.78	10.00
−1	3	−6	230.18	13.80
1	3	6	231.18	12.70
−1	3	−6	223.78	12.30
1	3	6	205.18	15.30
−2	−3	−6	1360.36	43.80
−2	−3	−6	1379.66	52.09
−2	−3	−6	1445.36	53.39
−2	−3	−6	1522.45	53.19
−2	−3	−6	1409.86	41.90
2	3	6	1456.35	40.90
2	3	6	1392.06	41.10
2	3	6	1370.96	51.59
−2	3	−6	1431.16	38.00
−2	3	−6	1377.16	36.90
−2	3	−6	1421.06	34.60
−2	3	−6	1386.46	43.10
−2	3	−6	1440.26	55.39
−2	3	−6	1490.35	45.70
−3	−3	−6	−1.10	8.20
−3	−3	−6	3.20	4.00
−3	−3	−6	4.10	6.90
−3	3	−6	4.00	7.00
3	3	6	−1.30	8.50
−3	3	−6	2.00	3.50
−3	3	−6	0.70	4.80
−3	3	−6	4.00	2.60
3	3	6	2.40	3.70
−3	3	−6	2.20	2.30
3	3	6	−12.20	4.80
3	3	6	2.70	6.50

−3	3	−6	1.20	2.80
−3	3	−6	−1.60	6.00
−4	−3	−6	459.55	21.80
−4	−3	−6	501.15	30.90
−4	−3	−6	451.25	20.80
−4	−3	−6	484.35	30.60
4	3	6	451.45	19.50
−4	3	−6	422.66	22.20
4	3	6	426.66	28.60
−4	3	−6	431.96	30.00
4	3	6	451.85	21.40
−4	3	−6	420.06	20.50
−4	3	−6	438.16	11.80
−4	3	−6	424.56	13.00
−4	3	−6	422.36	16.30
−5	−3	−6	66.09	14.10
−5	−3	−6	70.19	16.90
−5	−3	−6	55.59	9.00
5	3	6	57.89	9.00
5	3	6	72.49	9.90
−5	3	−6	56.29	7.80
−5	3	−6	64.19	7.80
−5	3	−6	66.49	10.90
−5	3	−6	64.29	14.20
5	3	6	61.69	13.50
−6	−3	−6	163.68	24.50
−6	−3	−6	198.88	26.80
−6	−3	−6	164.38	13.20
6	3	6	157.98	13.20
6	3	6	192.58	13.90
−6	3	−6	140.69	22.70

−6	3	−6	174.98	17.30
7	−3	6	26.40	4.30
−7	−3	−6	38.40	16.10
−7	−3	−6	20.20	12.00
7	3	6	26.80	7.40
7	3	6	34.80	8.10
−7	3	−6	19.70	9.30
−7	3	−6	34.30	15.70
8	−3	6	0.70	3.60
−8	−3	−6	8.60	11.70
−8	−3	−6	−12.90	11.40
8	3	6	−18.20	8.60
−8	3	−6	−0.90	14.40
−8	3	−6	4.00	10.60
8	3	6	−1.20	6.20
−9	−3	−6	42.90	18.40
−9	−3	−6	7.60	17.30
9	−3	6	2.60	5.30
9	3	6	0.90	8.60
−9	3	−6	10.60	13.70
−9	3	−6	6.30	12.40
−10	−3	−6	−5.40	20.30
−10	−3	−6	4.90	18.10
−10	3	−6	13.00	16.30
−10	3	−6	0.40	16.10
10	3	6	4.50	10.20
−10	−4	6	12.70	19.70
−10	−4	6	−0.40	15.90
10	−4	−6	9.90	14.30
10	−4	−6	14.70	11.00
−10	−4	6	17.20	22.00

10	4	-6	-6.30	9.40
-10	4	6	-6.70	18.70
-10	4	6	5.70	22.00
-10	4	6	-0.20	17.90
-9	-4	6	11.20	19.50
-9	-4	6	15.30	17.80
9	-4	-6	10.50	9.90
-9	-4	6	7.50	14.90
9	-4	-6	-21.70	10.70
-9	4	6	23.30	17.30
-9	4	6	20.10	15.20
9	4	-6	19.70	8.30
-9	4	6	19.70	17.50
8	-4	-6	2.50	10.90
-8	-4	6	36.80	20.30
8	-4	-6	17.50	9.90
-8	-4	6	24.00	15.70
-8	-4	6	14.30	12.10
-8	4	6	26.80	18.90
-8	4	6	41.80	17.90
-8	4	6	53.49	17.30
8	4	-6	21.60	10.00
-7	-4	6	-9.80	12.30
-7	-4	6	6.20	8.40
-7	-4	6	21.30	10.30
7	-4	-6	11.80	6.60
7	-4	-6	7.30	9.60
-7	-4	6	11.40	13.00
-7	4	6	-0.70	11.70
-7	4	6	-9.20	10.50
-7	4	6	7.00	11.20

7	4	−6	−4.70	4.90
6	−4	−6	191.68	16.20
−6	−4	6	203.18	16.20
−6	−4	6	156.68	23.70
−6	−4	6	165.68	27.70
6	−4	−6	175.78	22.30
−6	4	6	205.28	29.60
−6	4	6	167.38	24.40
6	4	−6	219.88	16.30
−6	4	6	170.28	24.70
−5	−4	6	−6.20	8.90
−5	−4	6	4.10	4.90
5	−4	−6	6.10	8.40
−5	−4	6	2.30	5.00
5	−4	−6	3.20	5.20
−5	4	6	7.00	8.80
−5	4	6	4.30	8.50
5	4	−6	0.20	5.10
−5	4	6	5.70	11.00
−4	−4	6	384.66	16.60
−4	−4	6	399.06	27.40
−4	−4	6	429.56	19.60
4	−4	−6	426.56	28.10
4	−4	−6	408.96	21.00
−4	−4	6	377.56	32.00
4	−4	−6	400.66	26.40
−4	4	6	422.56	28.10
−4	4	6	376.06	30.60
4	4	−6	371.16	27.50
4	4	−6	408.06	19.20
−4	4	6	378.16	29.50

3	−4	−6	5.30	5.90
3	−4	−6	0.50	6.90
−3	−4	6	0.80	5.90
3	4	−6	−4.90	6.20
3	4	−6	0.70	3.20
−3	4	6	−8.90	6.30
2	−4	−6	470.15	26.30
2	−4	−6	516.95	22.20
2	−4	−6	487.55	26.00
−2	−4	6	463.85	25.40
−2	−4	6	452.95	15.30
−2	4	6	415.56	30.00
−2	4	6	459.75	32.00
2	4	−6	460.45	17.70
2	4	−6	460.65	24.50
−1	−4	6	17.00	4.00
−1	−4	6	13.70	3.80
−1	−4	6	11.70	6.10
1	−4	−6	8.00	4.70
1	−4	−6	24.50	7.30
1	−4	−6	16.50	7.30
1	−4	−6	8.40	10.10
−1	4	6	15.20	8.90
−1	4	6	15.60	5.70
−1	4	6	14.10	7.70
1	4	−6	9.10	3.30
1	4	−6	26.30	12.00
1	4	−6	1.60	4.40
1	4	−6	17.40	5.90
0	−4	−6	69.89	10.80
0	−4	−6	84.79	15.90

0	-4	-6	74.69	11.40
0	-4	-6	90.29	18.50
0	-4	6	92.99	11.80
0	-4	-6	82.29	10.20
0	4	6	76.89	9.60
0	4	6	83.99	14.80
0	4	6	105.39	13.90
0	4	-6	87.89	9.00
0	4	-6	92.19	6.70
0	4	6	94.99	17.00
0	4	-6	80.49	10.00
0	4	-6	91.69	5.90
-1	-4	-6	-1.80	3.50
-1	-4	-6	0.80	4.30
-1	-4	-6	-4.20	8.30
1	4	6	-4.00	4.80
-1	4	-6	0.20	3.90
-1	4	-6	4.90	3.50
-1	4	-6	1.50	2.60
-1	4	-6	0.00	1.70
-1	4	-6	-1.00	1.80
1	4	6	2.50	3.10
1	4	6	8.90	6.80
1	4	6	-3.70	5.20
-2	-4	-6	1.90	6.20
-2	-4	-6	-2.40	4.10
-2	-4	-6	2.40	4.40
-2	-4	-6	4.10	7.20
-2	4	-6	7.50	3.20
-2	4	-6	5.20	2.90
-2	4	-6	2.00	2.90

-2	4	-6	1.60	2.50
2	4	6	3.10	5.30
2	4	6	-0.20	6.10
-2	4	-6	5.00	2.10
2	4	6	1.60	3.90
-2	4	-6	7.50	7.00
-2	4	-6	-3.10	4.80
-3	-4	-6	48.40	8.60
-3	-4	-6	33.40	7.40
-3	-4	-6	39.10	11.80
-3	-4	-6	50.09	13.10
-3	4	-6	52.49	9.30
-3	4	-6	49.10	6.00
-3	4	-6	48.60	5.30
-3	4	-6	41.10	6.80
-3	4	-6	47.80	4.70
3	4	6	32.40	10.40
3	4	6	38.20	12.40
3	4	6	42.90	7.70
3	4	6	61.49	9.60
-4	-4	-6	349.57	18.60
-4	-4	-6	386.16	20.20
-4	-4	-6	372.96	28.30
-4	-4	-6	381.76	28.30
-4	4	-6	388.56	14.60
-4	4	-6	383.86	29.20
4	4	6	378.26	27.50
-4	4	-6	390.46	12.70
-4	4	-6	387.96	17.40
-4	4	-6	353.16	19.30
-4	4	-6	403.66	14.30

4	4	6	395.76	18.70
4	4	6	409.36	21.40
−5	−4	−6	49.30	8.40
−5	−4	−6	98.39	18.30
−5	−4	−6	43.60	13.90
−5	4	−6	53.09	6.70
5	4	6	49.20	10.00
−5	4	−6	63.29	15.30
5	4	6	73.69	16.00
−5	4	−6	61.49	7.40
−5	4	−6	41.00	10.30
5	4	6	53.29	9.00
−6	−4	−6	59.19	8.60
−6	−4	−6	52.39	16.60
−6	−4	−6	50.89	16.60
−6	4	−6	46.60	15.60
−6	4	−6	42.90	10.00
6	4	6	32.70	8.80
−6	4	−6	46.30	10.00
6	4	6	38.40	8.40
−7	−4	−6	0.70	5.10
−7	−4	−6	−7.20	10.60
−7	4	−6	−8.60	7.20
7	4	6	−8.60	8.10
7	4	6	−3.50	5.10
−7	4	−6	1.20	10.70
−8	−4	−6	144.89	25.10
−8	−4	−6	136.29	27.40
−8	−4	−6	112.09	13.00
8	4	6	142.69	18.30
−8	4	−6	114.99	23.50

8	4	6	94.59	14.10
−8	4	−6	147.09	21.10
−9	−4	−6	36.30	20.40
−9	−4	−6	13.30	17.70
9	4	6	4.60	9.70
−9	4	−6	2.10	15.00
−9	4	−6	−9.80	12.20
−10	−4	−6	0.30	18.30
−10	4	−6	17.60	15.70
−10	4	−6	−4.20	20.60
10	4	6	−13.60	9.10
−10	−5	6	12.90	21.40
10	−5	−6	−13.60	15.00
−10	−5	6	−15.30	17.50
10	−5	−6	1.40	11.10
−10	5	6	15.20	32.50
−10	5	6	−11.10	19.10
10	5	−6	22.60	11.20
−10	5	6	20.80	23.30
−9	−5	6	38.40	19.80
−9	−5	6	20.70	14.40
−9	−5	6	8.10	17.10
9	−5	−6	8.50	10.50
−9	5	6	−6.10	17.60
9	5	−6	15.50	9.70
−9	5	6	10.20	15.70
−9	5	6	−4.70	18.20
−8	−5	6	31.80	19.80
−8	−5	6	8.50	11.10
8	−5	−6	4.30	10.30
8	−5	−6	9.90	9.00

−8	−5	6	−11.80	16.70
−8	5	6	7.50	14.30
−8	5	6	4.90	12.90
8	5	−6	−6.70	6.00
−8	5	6	10.40	19.60
7	−5	−6	71.09	12.80
7	−5	−6	63.79	14.60
−7	−5	6	77.99	13.80
−7	−5	6	73.89	22.30
−7	−5	6	81.29	13.50
−7	5	6	67.19	18.40
−7	5	6	55.09	20.60
−7	5	6	105.39	25.40
6	−5	−6	44.60	10.20
−6	−5	6	48.50	17.80
−6	−5	6	65.49	10.30
−6	−5	6	46.60	10.10
−6	−5	6	86.59	18.70
6	−5	−6	55.29	12.20
6	5	−6	44.60	9.80
−6	5	6	19.20	13.90
−6	5	6	49.80	17.00
−6	5	6	44.60	14.90
5	−5	−6	7.50	6.30
5	−5	−6	8.20	7.90
−5	−5	6	8.70	10.50
−5	−5	6	9.00	5.30
−5	−5	6	7.20	5.30
−5	−5	6	15.70	11.40
−5	5	6	25.00	12.20
−5	5	6	11.00	9.70

5	5	−6	5.70	4.70
−5	5	6	1.20	9.80
−4	−5	6	60.79	8.10
4	−5	−6	57.89	10.10
−4	−5	6	47.80	12.10
−4	−5	6	74.79	17.50
4	−5	−6	68.49	13.90
4	−5	−6	50.99	12.80
−4	5	6	79.39	18.80
−4	5	6	35.90	14.00
4	5	−6	59.59	9.10
4	5	−6	85.09	15.30
3	−5	−6	16.50	7.30
3	−5	−6	8.60	7.60
−3	−5	6	30.80	8.60
−3	−5	6	27.40	4.80
3	−5	−6	35.00	7.90
−3	5	6	27.30	11.70
3	5	−6	24.90	5.60
−3	5	6	12.00	11.40
3	5	−6	13.40	7.50
2	−5	−6	12.90	6.40
2	−5	−6	5.60	6.00
−2	−5	6	32.80	8.40
−2	−5	6	26.20	4.00
2	−5	−6	22.10	7.70
2	5	−6	21.00	4.90
−2	5	6	25.00	12.60
2	5	−6	14.50	7.50
−2	5	6	4.50	8.50
−1	−5	6	19.80	6.30

1	−5	−6	24.10	7.10
1	−5	−6	30.90	6.20
1	−5	−6	15.90	7.90
1	5	−6	16.50	5.90
1	5	−6	21.60	4.10
−1	5	6	17.80	14.70
−1	5	6	16.00	6.10
0	−5	−6	514.55	26.90
0	−5	−6	528.95	25.80
0	−5	−6	508.75	22.30
0	5	−6	495.45	19.80
0	5	6	510.05	22.80
0	5	−6	514.65	15.90
0	5	−6	519.95	19.30
0	5	−6	491.45	22.30
0	5	−6	503.55	17.70
0	5	6	513.95	37.80
−1	−5	−6	9.40	5.70
1	−5	6	3.00	4.30
−1	−5	−6	−1.80	4.10
−1	−5	−6	−2.30	6.80
−1	5	−6	4.50	2.30
1	5	6	7.80	4.60
1	5	6	2.30	6.10
−1	5	−6	2.30	1.80
−1	5	−6	3.60	4.20
−1	5	−6	−1.40	2.20
−2	−5	−6	257.97	15.50
−2	−5	−6	240.78	30.30
−2	−5	−6	250.07	17.20
−2	−5	−6	266.27	20.10

2	5	6	273.97	15.80
−2	5	−6	269.97	22.30
−2	5	−6	253.37	13.80
−2	5	−6	246.08	9.30
2	5	6	234.18	21.40
−2	5	−6	258.77	8.40
−3	−5	−6	−3.60	4.60
−3	−5	−6	6.20	4.10
−3	−5	−6	2.30	6.80
−3	−5	−6	3.20	7.70
3	5	6	1.50	7.40
−3	5	−6	5.90	3.40
−3	5	−6	5.90	7.70
−3	5	−6	1.40	2.00
−3	5	−6	−1.00	2.40
3	5	6	0.30	3.70
3	5	6	0.20	6.70
−4	−5	−6	70.89	10.90
−4	−5	−6	78.59	11.00
−4	−5	−6	55.09	12.30
−4	−5	−6	57.69	15.00
4	5	6	75.99	10.20
−4	5	−6	63.69	8.20
−4	5	−6	66.69	6.20
4	5	6	89.79	18.20
−4	5	−6	79.59	16.00
−4	5	−6	63.79	6.00
−4	5	−6	74.59	6.80
−4	5	−6	74.39	6.80
−5	−5	−6	8.20	9.90
−5	−5	−6	3.60	8.90

-5	-5	-6	-5.50	4.70
5	5	6	11.10	5.40
-5	5	-6	5.00	3.90
5	5	6	6.40	10.40
-5	5	-6	3.10	5.20
-5	5	-6	13.80	9.00
-5	5	-6	8.10	5.00
-6	-5	-6	81.09	17.80
-6	-5	-6	92.99	17.80
6	5	6	118.69	21.90
6	5	6	119.69	14.10
-6	5	-6	90.89	12.90
-6	5	-6	85.69	12.70
-6	5	-6	102.49	20.40
-7	-5	-6	1.80	6.20
-7	-5	-6	4.60	12.90
-7	-5	-6	7.80	13.10
-7	5	-6	-4.90	8.30
-7	5	-6	-1.90	7.70
-7	5	-6	1.30	9.50
-8	-5	-6	25.90	18.80
-8	-5	-6	8.50	16.00
-8	-5	-6	17.40	8.40
-8	5	-6	17.20	9.70
-8	5	-6	26.30	16.40
8	5	6	10.80	8.80
-9	-5	-6	3.40	16.30
-9	-5	-6	-3.10	14.30
-9	5	-6	-10.10	16.40
-9	5	-6	-0.80	12.30
9	5	6	-2.60	8.90

-10	-5	-6	21.30	21.70
10	5	6	-16.70	10.20
-10	5	-6	30.50	20.90
-10	5	-6	8.40	14.10
-10	-6	6	5.80	21.30
-10	-6	6	26.70	24.20
-10	-6	6	42.20	21.10
10	-6	-6	42.50	18.30
10	-6	-6	-6.60	12.30
-10	6	6	19.10	22.30
10	6	-6	29.30	12.70
-10	6	6	-52.39	41.40
-10	6	6	-6.10	19.70
-9	-6	6	-18.60	17.60
-9	-6	6	12.90	21.50
9	-6	-6	9.90	12.90
9	-6	-6	3.50	10.40
-9	-6	6	19.10	15.70
-9	6	6	0.60	15.40
-9	6	6	-12.00	20.90
9	6	-6	-6.90	9.00
-9	6	6	-18.70	16.10
-8	-6	6	8.00	23.70
8	-6	-6	24.50	9.80
-8	-6	6	-7.00	17.60
-8	-6	6	3.10	10.50
8	-6	-6	8.70	11.40
8	6	-6	1.30	7.30
-8	6	6	16.00	15.00
-8	6	6	9.50	20.20
-8	6	6	-10.10	14.30

7	−6	−6	14.10	9.10
−7	−6	6	−7.60	8.20
−7	−6	6	13.20	17.30
−7	−6	6	24.00	15.60
7	−6	−6	8.00	10.00
−7	6	6	8.50	14.80
−7	6	6	9.00	13.30
−7	6	6	−3.90	10.30
7	6	−6	−4.90	7.30
6	−6	−6	8.00	6.90
−6	−6	6	18.40	12.40
6	−6	−6	−0.70	8.60
−6	−6	6	11.60	7.10
−6	−6	6	21.30	7.40
−6	−6	6	12.70	12.50
−6	6	6	2.90	9.90
−6	6	6	−2.60	12.80
−6	6	6	1.50	11.30
6	6	−6	0.50	7.10
−5	−6	6	13.30	11.00
5	−6	−6	2.30	6.80
5	−6	−6	−0.80	9.00
−5	−6	6	−1.00	10.00
−5	−6	6	−2.30	5.00
−5	−6	6	3.00	4.60
−5	6	6	−1.40	12.50
−5	6	6	9.70	8.70
−5	6	6	−12.50	9.20
5	6	−6	6.30	4.60
4	−6	−6	21.00	7.10
−4	−6	6	7.10	9.40

4	−6	−6	0.30	9.40
−4	−6	6	11.40	5.20
4	−6	−6	24.30	9.90
4	6	−6	15.00	5.80
−4	6	6	25.80	12.30
−4	6	6	14.00	11.50
3	−6	−6	41.90	8.60
3	−6	−6	17.50	8.70
3	−6	−6	28.70	9.60
−3	−6	6	41.20	11.30
−3	−6	6	39.30	5.50
−3	6	6	57.39	15.90
3	6	−6	33.90	6.80
3	6	−6	23.60	10.20
−3	6	6	19.30	13.10
−2	−6	6	526.95	27.90
2	−6	−6	531.55	30.20
−2	−6	6	549.85	15.50
2	−6	−6	566.54	24.50
2	−6	−6	548.45	26.30
−2	6	6	627.54	41.20
2	6	−6	560.04	26.50
−2	6	6	543.75	37.40
2	6	−6	547.65	20.10
1	−6	−6	11.80	6.70
1	−6	−6	10.70	5.40
−1	−6	6	3.40	6.10
1	−6	−6	7.40	4.60
−1	6	6	8.10	9.20
1	6	−6	8.00	3.10
−1	6	6	5.90	4.00

1	6	−6	12.90	4.80
−1	6	6	9.30	10.70
0	−6	−6	99.19	13.20
0	−6	−6	97.39	14.20
0	−6	−6	90.59	11.20
0	6	−6	85.59	7.50
0	6	6	106.59	18.20
0	6	−6	105.79	6.30
0	6	6	100.59	18.50
0	6	6	108.09	12.60
−1	−6	−6	9.30	4.50
1	−6	6	−2.50	6.20
−1	−6	−6	4.50	5.60
−1	−6	−6	7.70	6.30
−1	−6	−6	8.50	8.30
1	6	6	7.80	4.50
1	6	6	13.80	9.20
−1	6	−6	17.20	5.80
−1	6	−6	2.40	1.70
−1	6	−6	−2.80	4.70
−2	−6	−6	479.75	32.90
−2	−6	−6	510.55	27.10
−2	−6	−6	498.35	22.70
−2	−6	−6	453.35	25.50
2	6	6	478.25	32.60
−2	6	−6	487.55	27.70
−2	6	−6	466.85	12.50
−2	6	−6	515.75	31.90
−2	6	−6	518.75	21.40
2	6	6	482.95	22.60
−3	−6	−6	16.20	6.90

−3	−6	−6	−11.70	8.30
−3	−6	−6	2.60	6.90
−3	−6	−6	6.30	4.90
−3	6	−6	−2.20	1.80
3	6	6	5.20	5.40
−3	6	−6	−5.10	7.30
3	6	6	−3.70	6.80
−3	6	−6	0.30	7.20
−3	6	−6	−6.20	4.10
3	6	6	−8.20	7.40
−3	6	−6	1.00	2.10
−4	−6	−6	80.59	12.40
−4	−6	−6	66.89	17.20
−4	−6	−6	63.29	15.30
−4	6	−6	74.29	17.60
4	6	6	55.49	9.60
−4	6	−6	63.59	5.50
4	6	6	69.39	17.40
−4	6	−6	78.19	9.10
−4	6	−6	69.89	8.10
−5	−6	−6	32.20	11.90
−5	−6	−6	21.30	7.00
−5	−6	−6	25.30	13.10
−5	6	−6	18.70	5.20
−5	6	−6	18.10	10.30
5	6	6	24.60	7.40
5	6	6	19.50	11.40
−6	−6	−6	−1.50	6.10
−6	−6	−6	−7.80	9.40
−6	−6	−6	11.00	12.50
−6	6	−6	3.90	6.50

6	6	6	4.60	9.40
6	6	6	5.90	5.50
−6	6	−6	10.40	12.30
−7	−6	−6	−21.10	12.20
−7	−6	−6	−3.30	7.30
−7	−6	−6	12.80	13.70
−7	6	−6	4.40	7.10
7	6	6	5.10	6.30
−7	6	−6	7.60	8.60
−7	6	−6	1.20	12.60
−8	−6	−6	15.20	14.60
−8	−6	−6	7.90	8.50
−8	−6	−6	53.39	18.70
−8	6	−6	13.50	14.10
8	6	6	18.20	9.10
−8	6	−6	23.20	10.40
−9	−6	−6	−10.00	18.60
−9	−6	−6	−7.50	16.50
−9	6	−6	6.20	11.10
−9	6	−6	−23.70	15.90
9	6	6	−2.20	9.20
−9	6	−6	2.90	20.10
−10	−6	−6	27.60	22.20
−10	−6	−6	28.90	23.40
−10	6	−6	−10.20	25.10
−10	6	−6	−13.70	13.30
10	6	6	2.90	12.10
−10	6	−6	44.00	24.90
−9	−7	6	−14.20	21.50
9	−7	−6	−4.10	13.90
−9	−7	6	−7.80	14.80

-9	-7	6	8.70	23.20
9	7	-6	26.60	11.50
-9	7	6	-9.80	18.20
-9	7	6	-5.00	18.40
-9	7	6	-11.80	20.50
8	-7	-6	-5.10	13.70
-8	-7	6	-5.30	12.40
8	-7	-6	13.40	9.30
-8	-7	6	-11.60	18.30
-8	7	6	27.90	22.40
8	7	-6	-15.10	7.30
-8	7	6	5.30	15.90
-8	7	6	15.70	17.30
7	-7	-6	0.60	10.80
-7	-7	6	2.80	14.70
-7	-7	6	-12.30	17.30
-7	-7	6	-15.70	12.90
7	-7	-6	11.90	7.80
-7	7	6	-12.10	13.10
-7	7	6	4.00	12.10
-7	7	6	-1.10	15.30
7	7	-6	6.20	8.30
-6	-7	6	65.49	11.40
6	-7	-6	53.89	15.30
-6	-7	6	98.29	25.70
-6	-7	6	67.49	18.10
6	-7	-6	51.39	11.30
-6	-7	6	49.50	9.50
-6	7	6	93.69	18.00
6	7	-6	61.19	11.50
-5	-7	6	12.20	14.80

5	−7	−6	21.00	7.50
5	−7	−6	0.50	8.90
−5	−7	6	7.40	10.70
−5	7	6	5.40	12.50
5	7	−6	6.80	6.00
−5	7	6	1.50	14.00
−4	−7	6	129.79	10.40
4	−7	−6	84.49	15.90
4	−7	−6	87.99	11.60
4	−7	−6	127.69	25.20
−4	−7	6	120.89	20.20
−4	7	6	115.59	23.90
4	7	−6	129.89	11.40
−4	7	6	121.99	25.30
4	7	−6	102.09	18.20
−3	−7	6	18.10	8.80
−3	−7	6	2.60	3.50
3	−7	−6	11.20	8.70
3	−7	−6	13.60	6.10
3	−7	−6	17.30	8.30
−3	7	6	−5.70	10.20
−3	7	6	25.60	11.50
3	7	−6	7.00	8.10
3	7	−6	15.30	5.50
2	−7	−6	50.89	12.50
2	−7	−6	58.29	10.10
2	−7	−6	70.39	10.60
2	7	−6	45.20	9.70
−2	7	6	43.80	13.60
2	7	−6	56.49	7.40
−2	7	6	49.30	9.40

-2	7	6	59.59	16.70
1	-7	-6	165.88	19.80
1	-7	-6	174.08	13.10
-1	-7	6	152.88	14.10
1	-7	-6	183.28	13.30
1	-7	-6	217.28	14.60
-1	7	6	123.69	21.60
-1	7	6	161.28	13.80
1	7	-6	166.98	7.80
-1	7	6	146.79	25.40
1	7	-6	196.98	10.00
1	7	-6	171.28	12.70
0	-7	-6	125.39	11.60
0	-7	6	140.39	12.40
0	-7	-6	137.49	13.00
0	-7	-6	126.59	16.90
0	7	6	149.99	23.60
0	7	6	110.99	19.50
0	7	-6	117.59	7.60
0	7	-6	137.69	7.20
0	7	-6	124.59	12.50
0	7	6	131.79	14.50
-1	-7	-6	47.90	8.70
-1	-7	-6	49.90	10.80
-1	-7	-6	73.19	10.10
1	7	6	69.09	10.50
-1	7	-6	58.09	11.80
-1	7	-6	65.09	4.10
1	7	6	57.49	16.10
-2	-7	-6	45.90	9.50
-2	-7	-6	49.50	10.70

−2	−7	−6	78.59	18.10
−2	−7	−6	62.39	12.50
2	7	6	57.79	9.70
−2	7	−6	90.49	15.70
2	7	6	79.29	17.10
−3	−7	−6	22.90	8.20
−3	−7	−6	13.80	8.70
−3	−7	−6	24.60	13.10
−3	−7	−6	28.90	8.00
3	7	6	29.30	8.00
−3	7	−6	14.60	11.90
−3	7	−6	16.50	9.30
3	7	6	35.80	14.00
3	7	6	30.10	12.00
−3	7	−6	28.80	6.80
−4	−7	−6	15.20	10.10
−4	−7	−6	−2.20	11.80
−4	−7	−6	8.10	7.90
−4	7	−6	7.50	2.80
−4	7	−6	12.80	10.20
4	7	6	1.80	5.60
−4	7	−6	−12.70	4.40
−4	7	−6	4.30	9.10
−4	7	−6	0.60	2.40
4	7	6	−8.00	7.30
−5	−7	−6	52.19	18.10
−5	−7	−6	19.50	12.30
−5	−7	−6	38.30	10.60
5	7	6	44.30	10.20
−5	7	−6	39.00	15.20
−5	7	−6	29.50	7.00

−5	7	−6	44.80	14.80
5	7	6	32.70	12.60
−5	7	−6	45.30	6.00
−6	−7	−6	110.49	23.70
−6	−7	−6	70.49	21.00
−6	−7	−6	72.39	13.90
−6	7	−6	75.19	18.40
6	7	6	81.79	13.30
−6	7	−6	88.89	21.10
−7	−7	−6	−14.30	7.30
−7	−7	−6	−8.40	14.00
−7	−7	−6	16.00	16.60
−7	7	−6	2.00	6.50
7	7	6	−8.60	6.80
−7	7	−6	−18.30	12.60
−7	7	−6	7.00	6.50
−7	7	−6	10.20	17.40
−8	−7	−6	29.50	19.60
−8	−7	−6	8.50	9.90
−8	−7	−6	8.30	16.40
−8	7	−6	25.00	9.80
−8	7	−6	9.80	19.80
8	7	6	7.20	9.10
−8	7	−6	30.00	15.30
−9	−7	−6	45.00	20.50
−9	−7	−6	−0.50	10.10
9	7	6	−3.30	9.20
−9	7	−6	10.50	22.70
−9	7	−6	11.50	18.80
−9	7	−6	5.10	12.60
−9	−8	6	−29.40	23.10

9	−8	−6	−12.00	11.00
−9	−8	6	−13.20	20.70
9	−8	−6	6.40	14.90
−9	8	6	−24.70	30.10
−9	8	6	9.30	23.70
−9	8	6	−4.80	18.70
9	8	−6	9.70	10.80
−8	−8	6	−20.80	11.40
−8	−8	6	30.30	19.00
8	−8	−6	25.80	11.60
8	−8	−6	13.70	14.10
−8	−8	6	37.60	24.40
−8	8	6	27.90	17.10
−8	8	6	19.40	21.20
−8	8	6	53.49	22.50
8	8	−6	28.80	10.90
−7	−8	6	−15.30	8.50
−7	−8	6	38.70	21.40
7	−8	−6	17.30	8.30
−7	−8	6	−5.70	16.30
7	8	−6	7.20	9.10
−7	8	6	2.40	11.60
−7	8	6	0.30	17.00
−7	8	6	−3.70	14.20
−6	−8	6	−13.30	9.20
−6	−8	6	−4.00	16.30
−6	−8	6	−4.70	13.60
−6	−8	6	−13.50	9.60
6	−8	−6	8.50	11.20
6	−8	−6	−0.70	7.50
−6	8	6	0.80	15.20

−6	8	6	−0.50	10.20
6	8	−6	0.80	8.40
−6	8	6	28.00	15.50
5	−8	−6	4.50	13.50
5	−8	−6	25.80	7.60
−5	−8	6	30.00	15.30
5	−8	−6	6.80	8.90
5	8	−6	15.20	7.10
−5	8	6	18.60	15.60
−5	8	6	26.40	15.30
4	−8	−6	72.09	14.60
4	−8	−6	73.29	12.30
−4	−8	6	58.89	15.70
4	−8	−6	62.79	10.20
−4	8	6	49.00	18.80
−4	8	6	48.90	17.00
4	8	−6	52.09	13.70
4	8	−6	52.89	8.90
−3	−8	6	-1.80	2.80
3	−8	−6	-3.70	6.20
3	−8	−6	2.50	5.50
−3	−8	6	0.60	7.90
−3	8	6	18.20	14.40
3	8	−6	5.20	4.90
3	8	−6	6.50	8.50
−3	8	6	−2.70	9.40
2	−8	−6	−8.40	5.00
2	−8	−6	0.50	6.70
2	−8	−6	5.30	5.50
−2	−8	6	9.30	8.30
2	8	−6	1.60	4.00

-2	8	6	8.90	5.30
-2	8	6	-13.80	11.10
-2	8	6	10.90	11.10
2	8	-6	0.80	6.80
1	-8	-6	-4.40	4.70
-1	-8	6	0.00	5.60
1	-8	-6	2.20	4.70
1	-8	-6	-4.90	5.40
1	-8	-6	-5.80	5.60
-1	8	6	5.30	9.90
1	8	-6	6.00	3.90
-1	8	6	-0.50	4.60
1	8	-6	8.50	5.30
-1	8	6	-0.30	10.70
1	8	-6	0.90	3.50
0	-8	-6	127.29	12.50
0	-8	-6	148.29	27.90
0	-8	-6	158.28	12.10
0	-8	-6	135.69	12.70
0	-8	6	145.79	14.80
0	-8	-6	120.49	17.50
0	-8	6	152.88	13.80
0	8	6	150.68	13.70
0	8	-6	147.09	7.90
0	8	-6	114.89	15.30
0	8	6	144.09	24.30
-1	-8	-6	-3.50	5.90
-1	-8	-6	-2.50	5.10
-1	-8	-6	5.70	6.70
1	-8	6	3.60	5.60
-1	-8	-6	-0.40	8.80

-1	8	-6	-7.50	9.90
1	8	6	3.90	9.20
-1	8	-6	5.10	2.00
1	8	6	12.00	5.80
-1	8	-6	-0.80	8.50
-2	-8	-6	69.29	14.10
-2	-8	-6	52.39	9.70
-2	-8	-6	60.79	17.80
2	-8	6	57.69	11.20
-2	8	-6	61.19	14.00
2	8	6	65.59	16.50
2	8	6	54.09	10.80
-2	8	-6	45.30	12.80
-3	-8	-6	46.20	18.10
-3	-8	-6	23.00	10.00
-3	-8	-6	53.49	12.10
3	-8	6	49.00	8.80
3	8	6	32.30	8.80
3	8	6	25.00	12.40
-3	8	-6	48.50	13.90
-3	8	-6	24.60	11.70
-4	-8	-6	203.38	25.20
-4	-8	-6	205.48	28.40
-4	-8	-6	252.47	20.30
-4	8	-6	250.87	26.80
-4	8	-6	223.78	24.40
4	8	6	209.98	28.70
4	8	6	244.98	18.30
-4	8	-6	182.58	15.20
-4	8	-6	216.58	8.10
-5	-8	-6	48.40	14.40

−5	−8	−6	24.70	9.10
−5	−8	−6	32.80	17.60
5	8	6	25.70	15.60
−5	8	−6	39.30	5.30
−5	8	−6	27.50	13.70
−5	8	−6	25.60	13.50
5	8	6	30.60	9.70
−6	−8	−6	7.40	13.50
−6	−8	−6	−1.90	13.90
−6	−8	−6	4.70	7.70
6	8	6	−0.10	7.10
−6	8	−6	−6.50	14.60
6	8	6	1.50	11.90
−6	8	−6	11.50	5.70
−6	8	−6	−8.60	7.10
−6	8	−6	36.50	13.40
−7	−8	−6	−2.90	16.50
−7	−8	−6	−13.70	8.00
7	8	6	7.50	8.20
7	8	6	0.50	12.70
−7	8	−6	−5.10	13.40
−8	−8	−6	−10.10	18.90
−8	−8	−6	33.60	11.40
−8	−8	−6	29.30	20.10
−8	8	−6	12.40	15.90
−8	8	−6	9.80	22.40
8	8	6	16.60	10.40
−9	−8	−6	26.90	13.40
−9	−8	−6	9.30	23.20
−9	−8	−6	36.30	24.20
9	8	6	−19.00	8.80

-9	8	-6	17.30	19.50
9	-9	-6	-8.40	16.10
-9	-9	6	-17.80	25.50
-9	-9	6	-0.90	27.80
-9	9	6	12.20	20.40
-9	9	6	-2.90	21.70
-9	9	6	-33.70	19.40
8	-9	-6	5.90	10.80
8	-9	-6	5.60	14.10
-8	-9	6	-9.90	21.20
-8	-9	6	27.90	25.20
-8	9	6	-2.80	15.20
-8	9	6	-4.70	19.20
8	9	-6	-3.50	10.10
-8	9	6	-3.80	19.30
-7	-9	6	46.30	23.80
7	-9	-6	41.80	10.30
7	-9	-6	11.70	12.80
-7	-9	6	11.00	17.80
7	9	-6	39.90	11.30
-7	9	6	30.50	20.70
-7	9	6	26.90	15.00
-7	9	6	53.29	25.60
-6	-9	6	14.40	17.20
6	-9	-6	12.70	7.60
6	-9	-6	5.50	10.60
6	9	-6	7.10	8.20
-6	9	6	-5.50	16.40
-6	9	6	1.50	15.00
5	-9	-6	-4.00	10.00
5	-9	-6	10.60	7.00

5	-9	-6	-28.80	20.80
-5	9	6	7.10	15.20
-5	9	6	9.30	14.70
5	9	-6	15.20	7.70
-4	-9	6	175.78	10.70
4	-9	-6	179.38	17.70
4	-9	-6	148.29	22.10
-4	-9	6	194.28	25.70
4	-9	-6	203.08	15.40
-4	9	6	203.68	30.00
4	9	-6	261.77	28.30
4	9	-6	184.88	15.20
3	-9	-6	-2.60	5.10
3	-9	-6	-3.80	6.00
-3	-9	6	2.90	9.20
3	-9	-6	-3.70	8.10
-3	9	6	-1.10	10.90
3	9	-6	25.00	10.00
3	9	-6	5.10	5.30
-3	9	6	-1.00	14.00
-2	-9	6	74.69	14.00
2	-9	-6	49.40	13.10
2	-9	-6	98.49	12.80
-2	9	6	88.49	23.70
-2	9	6	61.59	11.40
-2	9	6	51.99	16.60
2	9	-6	86.39	9.70
2	9	-6	87.89	12.90
-1	-9	6	-8.90	7.40
1	-9	-6	13.70	6.00
-1	-9	6	18.10	7.40

1	−9	−6	0.50	7.90
−1	9	6	8.00	10.00
1	9	−6	8.70	5.90
1	9	−6	16.60	5.60
1	9	−6	12.00	4.70
−1	9	6	13.00	13.10
−1	9	6	17.60	7.60
0	−9	6	126.49	15.60
0	−9	−6	130.59	19.00
0	−9	−6	206.88	28.10
0	−9	6	169.68	16.20
0	−9	−6	163.28	14.00
0	9	6	152.38	16.80
0	9	−6	142.39	8.60
0	9	−6	159.58	18.30
0	9	6	130.29	24.90
−1	−9	−6	19.60	9.80
1	−9	6	34.40	8.70
−1	−9	−6	22.30	11.10
−1	−9	−6	29.10	7.30
−1	−9	−6	33.10	8.30
1	9	6	25.50	13.00
1	9	6	9.70	8.10
−1	9	−6	25.00	3.60
−1	9	−6	19.10	9.30
−1	9	−6	16.20	12.30
−2	−9	−6	23.70	9.90
−2	−9	−6	−1.70	10.40
2	−9	6	5.90	6.80
−2	−9	−6	15.50	7.60
2	9	6	20.40	13.40

2	9	6	21.60	7.70
-2	9	-6	20.00	11.00
-2	9	-6	18.40	12.70
-3	-9	-6	7.70	9.50
3	-9	6	12.40	6.90
-3	-9	-6	28.80	9.70
-3	-9	-6	-9.90	12.90
-3	9	-6	15.50	10.80
3	9	6	10.00	6.50
3	9	6	0.80	10.70
-3	9	-6	3.40	10.90
-4	-9	-6	14.30	8.90
4	-9	6	19.90	8.00
-4	-9	-6	10.80	10.70
-4	-9	-6	20.00	14.70
-4	9	-6	22.90	14.60
-4	9	-6	11.60	12.10
4	9	6	1.20	11.70
-4	9	-6	20.70	3.10
-5	-9	-6	12.60	9.30
-5	-9	-6	10.90	14.90
-5	-9	-6	-3.90	12.30
-5	9	-6	-12.50	11.90
5	9	6	-0.80	7.90
-5	9	-6	17.60	8.70
-5	9	-6	4.90	4.00
5	9	6	3.60	12.40
-5	9	-6	0.00	14.90
-6	-9	-6	21.30	11.20
-6	-9	-6	7.10	17.00
-6	-9	-6	16.60	15.40

-6	9	-6	26.00	15.30
6	9	6	43.30	18.40
-6	9	-6	53.19	20.40
6	9	6	28.20	10.90
-7	-9	-6	26.20	21.70
-7	-9	-6	-6.50	9.90
-7	-9	-6	31.20	18.60
7	9	6	-5.50	14.10
-7	9	-6	-8.90	19.60
-7	9	-6	49.80	18.50
7	9	6	8.50	10.00
-8	-9	-6	41.90	25.40
-8	-9	-6	-12.00	10.70
-8	-9	-6	25.70	20.10
-8	9	-6	-6.70	17.20
-8	9	-6	20.20	21.70
-8	-10	6	-4.70	23.40
8	-10	-6	6.90	16.60
-8	-10	6	-1.50	25.00
8	10	-6	36.40	19.40
-8	10	6	-13.80	20.50
-8	10	6	7.60	22.10
7	-10	-6	11.30	12.90
7	-10	-6	-5.70	8.90
-7	-10	6	-4.20	19.30
-7	10	6	-0.70	20.50
-7	10	6	30.50	19.70
7	10	-6	7.90	9.50
-6	-10	6	40.40	20.70
6	-10	-6	33.50	13.80
6	-10	-6	33.70	8.80

-6	10	6	40.30	24.60
6	10	-6	16.80	9.70
-6	10	6	10.80	16.50
5	-10	-6	5.10	10.00
5	-10	-6	5.80	7.10
-5	10	6	10.20	17.50
5	10	-6	-3.00	6.90
-5	10	6	2.20	14.90
4	-10	-6	10.00	7.10
-4	-10	6	12.90	13.20
4	-10	-6	-2.80	11.40
-4	-10	6	15.10	4.70
-4	10	6	2.20	14.00
4	10	-6	14.10	13.30
4	10	-6	8.60	7.10
-4	10	6	18.00	14.40
3	-10	-6	37.50	8.90
-3	-10	6	29.80	13.00
-3	10	6	44.10	11.40
3	10	-6	29.80	8.20
3	10	-6	53.79	13.30
-3	10	6	59.69	22.90
-3	10	6	57.19	20.70
-2	-10	6	8.50	10.00
2	-10	-6	16.30	7.20
2	-10	-6	27.20	11.10
2	10	-6	21.10	5.30
-2	10	6	19.10	9.50
-2	10	6	-13.90	10.40
2	10	-6	23.70	9.70
2	10	-6	17.70	6.60

-2	10	6	24.60	15.70
-1	-10	6	42.00	11.40
1	-10	-6	38.30	9.40
1	-10	-6	65.79	20.40
1	-10	-6	44.80	12.30
1	10	-6	32.60	5.80
-1	10	6	35.90	10.40
-1	10	6	37.00	19.10
1	10	-6	38.00	8.20
0	-10	-6	212.78	15.70
0	-10	-6	219.68	26.40
0	-10	-6	218.98	29.90
0	-10	6	257.97	19.90
0	-10	6	234.98	16.60
0	10	6	201.08	18.60
0	10	-6	191.08	10.70
0	10	6	219.18	32.50
-1	-10	-6	4.20	13.40
1	-10	6	-12.60	5.40
-1	-10	-6	13.20	10.20
-1	10	-6	-6.50	11.40
1	10	6	5.70	11.70
-1	10	-6	1.10	9.70
1	10	6	-0.40	7.40
2	-10	6	229.48	19.00
-2	-10	-6	217.68	26.00
-2	-10	-6	287.27	34.90
-2	-10	-6	269.57	18.30
2	10	6	198.08	30.30
-2	10	-6	211.28	7.60
-2	10	-6	276.67	24.90

2	10	6	203.78	18.30
−2	10	−6	180.88	26.70
−3	−10	−6	20.40	10.00
3	−10	6	−2.80	7.10
−3	−10	−6	−2.80	9.50
−3	−10	−6	4.20	14.80
−3	10	−6	−12.00	12.50
−3	10	−6	8.50	11.20
4	−10	6	25.00	9.40
−4	−10	−6	42.90	14.00
−4	−10	−6	26.00	17.70
−4	10	−6	43.90	17.10
4	10	6	26.20	17.60
4	10	6	33.70	10.80
−4	10	−6	45.10	15.00
−5	−10	−6	27.60	15.00
−5	−10	−6	−28.90	28.80
−5	−10	−6	13.20	8.90
−5	10	−6	14.00	12.00
5	10	6	−5.90	7.70
5	10	6	5.90	12.90
−5	10	−6	−33.90	14.80
−6	−10	−6	14.70	10.30
−6	−10	−6	9.30	19.00
−6	−10	−6	14.30	17.40
−6	10	−6	28.70	21.10
6	10	6	32.10	16.90
−6	10	−6	10.50	15.90
6	10	6	21.50	10.20
−7	−10	−6	−7.00	20.00
−7	−10	−6	−15.80	10.90

-7	-10	-6	40.30	27.30
7	10	6	-20.10	14.20
7	10	6	-2.80	10.60
-7	10	-6	1.10	17.80
-7	10	-6	-5.90	15.30
-8	-10	-6	18.40	26.60
-8	-10	-6	-2.20	11.70
-8	-10	-6	22.90	24.70
-8	10	-6	-3.90	21.10
-8	10	-6	-22.10	18.20
8	10	6	9.00	10.30
7	-11	-6	-17.80	14.70
-7	-11	6	-20.60	21.70
-7	11	6	11.80	20.30
7	11	-6	8.50	10.80
-7	11	6	-19.80	20.90
6	-11	-6	-11.50	14.10
6	-11	-6	11.20	14.00
-6	-11	6	20.20	18.50
-6	11	6	5.10	15.70
-6	11	6	24.10	24.20
6	11	-6	15.00	10.60
5	-11	-6	0.30	7.00
5	-11	-6	-37.30	12.00
-5	-11	6	-2.10	16.50
-5	11	6	5.40	15.70
5	11	-6	20.00	9.10
-5	11	6	23.10	20.70
-4	-11	6	80.09	18.90
4	-11	-6	62.49	16.00
4	-11	-6	77.89	10.90

-4	11	6	96.29	26.70
4	11	-6	95.59	13.90
-4	11	6	92.39	27.50
-3	-11	6	4.90	12.70
3	-11	-6	19.00	7.40
3	-11	-6	3.90	11.40
-3	11	6	11.10	18.30
-3	11	6	24.30	17.20
3	11	-6	19.10	13.40
-3	11	6	1.30	8.30
3	11	-6	7.00	6.90
-2	-11	6	99.89	17.10
2	-11	-6	94.39	19.00
2	-11	-6	94.79	12.00
-2	-11	6	112.59	17.10
2	11	-6	102.69	11.70
-2	11	6	104.59	26.40
2	11	-6	77.19	13.80
-2	11	6	80.89	13.70
2	11	-6	100.79	10.50
1	-11	-6	48.00	14.20
1	-11	-6	58.89	18.20
1	-11	-6	39.00	9.40
-1	11	6	62.19	21.50
1	11	-6	39.10	10.00
-1	11	6	38.00	12.40
1	11	-6	44.30	6.60
0	-11	6	2.40	9.10
0	-11	-6	-1.60	9.40
0	-11	6	11.20	7.60
0	-11	-6	4.50	14.90

0	11	6	7.20	15.30
0	11	-6	-8.10	9.00
0	11	6	12.70	9.30
-1	-11	-6	-2.40	13.60
-1	-11	-6	6.30	8.80
1	-11	6	-4.30	6.50
1	11	6	2.30	7.70
-1	11	-6	3.40	3.60
-1	11	-6	18.00	12.90
1	11	6	6.20	16.20
-1	11	-6	-4.60	10.60
-2	-11	-6	-4.40	8.30
-2	-11	-6	-4.50	13.30
2	-11	6	-13.20	7.00
2	11	6	11.30	13.90
-2	11	-6	-14.80	10.00
2	11	6	5.70	7.80
-2	11	-6	-2.10	2.70
-2	11	-6	20.70	13.40
-3	-11	-6	62.59	22.20
3	-11	6	28.00	10.90
-3	-11	-6	32.60	19.20
-3	-11	-6	26.60	12.20
-3	11	-6	28.00	13.90
-3	11	-6	39.00	18.20
3	11	6	10.00	15.00
3	11	6	33.40	10.40
-4	-11	-6	36.70	19.60
4	-11	6	8.20	9.20
-4	-11	-6	17.20	12.50
-4	-11	-6	33.40	13.60

-4	11	-6	16.80	13.60
4	11	6	29.50	11.70
4	11	6	6.60	16.80
-4	11	-6	-2.40	16.60
-5	-11	-6	21.00	12.00
5	-11	6	-2.00	9.30
-5	-11	-6	4.90	15.50
-5	-11	-6	3.20	20.40
-5	11	-6	-30.50	16.20
5	11	6	32.90	17.20
5	11	6	16.00	10.10
-5	11	-6	20.70	13.70
-6	-11	-6	50.79	27.60
-6	-11	-6	17.50	12.80
-6	-11	-6	56.79	21.80
-6	11	-6	44.30	24.30
6	11	6	-8.20	9.60
6	11	6	3.20	16.40
-6	11	-6	15.60	15.50
-7	-11	-6	-8.80	12.20
-7	-11	-6	61.49	25.40
-7	-11	-6	-11.60	24.50
-7	11	-6	-19.50	16.10
-7	11	-6	24.50	25.50
7	11	6	-14.90	10.10
7	11	6	-23.60	18.70
-6	-12	6	16.20	25.50
6	-12	-6	12.70	14.20
6	12	-6	-8.50	9.70
-6	12	6	-4.40	24.60
-6	12	6	15.90	17.90

5	-12	-6	45.50	15.20
-5	-12	6	15.00	20.50
5	12	-6	13.20	8.60
-5	12	6	42.90	23.00
-5	12	6	10.40	18.70
-4	-12	6	-10.70	15.50
4	-12	-6	13.90	13.60
4	12	-6	4.00	7.80
-4	12	6	-11.60	8.70
-4	12	6	5.50	16.40
-4	12	6	-6.20	18.60
-3	-12	6	10.10	14.20
3	-12	-6	-3.90	10.60
-3	12	6	-3.00	8.70
3	12	-6	7.70	7.10
-3	12	6	3.10	21.40
2	-12	-6	3.70	10.90
-2	-12	6	7.50	8.80
-2	-12	6	-7.20	12.10
2	-12	-6	-7.80	17.40
-2	12	6	-23.60	15.70
2	12	-6	19.70	7.30
2	12	-6	15.00	7.00
-2	12	6	2.70	8.80
-1	-12	6	-8.70	14.00
1	-12	-6	10.00	11.60
-1	-12	6	0.80	9.00
1	-12	-6	22.50	18.30
-1	12	6	17.70	10.70
-1	12	6	23.60	18.40
1	12	-6	8.60	9.50

1	12	-6	2.20	5.60
0	-12	-6	25.20	18.80
0	-12	6	38.30	10.90
0	-12	-6	31.90	13.80
0	12	6	44.90	11.10
0	12	6	60.19	22.20
0	12	-6	72.99	15.40
0	12	-6	54.29	8.00
0	12	-6	52.19	16.80
-1	-12	-6	-15.00	9.00
1	-12	6	12.00	11.00
-1	-12	-6	1.90	18.90
1	12	6	-3.50	17.40
-1	12	-6	-20.10	12.90
-1	12	-6	-2.00	4.40
-1	12	-6	1.40	11.60
1	12	6	1.80	8.60
-2	-12	-6	9.00	10.40
-2	-12	-6	-19.00	17.50
2	-12	6	21.40	10.70
2	12	6	43.60	19.50
2	12	6	20.40	10.30
-2	12	-6	24.60	17.60
-2	12	-6	36.70	13.10
3	-12	6	-6.10	9.10
-3	-12	-6	7.30	17.60
-3	-12	-6	-11.80	9.40
-3	-12	-6	-15.30	10.20
3	12	6	-3.70	18.40
-3	12	-6	1.50	11.60
-3	12	-6	13.10	18.60

3	12	6	7.90	9.00
-4	-12	-6	18.30	15.20
-4	-12	-6	56.29	17.90
-4	-12	-6	63.99	26.50
4	-12	6	31.20	12.70
4	12	6	37.90	13.10
-4	12	-6	21.20	13.90
-4	12	-6	57.79	23.30
4	12	6	61.79	23.30
-5	-12	-6	-24.90	17.70
-5	-12	-6	-3.50	12.20
5	-12	6	-2.00	10.60
-5	-12	-6	-20.50	21.30
-5	12	-6	-17.80	14.80
5	12	6	6.50	10.60
-5	12	-6	49.70	23.10
5	12	6	5.70	18.00
-6	-12	-6	-18.20	21.60
-6	-12	-6	0.20	25.70
-6	-12	-6	16.20	14.20
6	12	6	8.40	11.60
-6	12	-6	-12.50	24.90
-6	12	-6	23.50	17.80
6	12	6	-3.70	20.60
5	-13	-6	4.70	14.50
-5	13	6	19.10	21.70
-5	13	6	-20.40	17.20
5	13	-6	17.00	10.70
-5	13	6	-12.10	10.50
4	-13	-6	39.20	16.90
-4	13	6	46.40	14.90

4	13	-6	46.30	13.00
-4	13	6	28.70	24.00
3	-13	-6	35.30	26.60
3	-13	-6	-31.10	13.90
-3	13	6	31.20	23.20
-3	13	6	13.50	11.80
3	13	-6	8.10	7.70
2	-13	-6	29.10	14.70
2	-13	-6	8.40	23.30
-2	-13	6	4.50	11.80
-2	13	6	34.10	24.20
2	13	-6	20.80	8.30
2	13	-6	38.20	13.10
-2	13	6	5.10	12.10
-1	-13	6	-0.80	9.70
1	-13	-6	4.40	18.80
1	-13	-6	-8.60	11.90
1	13	-6	9.30	7.00
1	13	-6	1.40	9.70
-1	13	6	17.00	20.20
-1	13	6	10.50	11.40
0	-13	6	21.20	12.10
0	-13	-6	12.40	13.50
0	-13	-6	13.80	20.80
0	13	-6	42.00	8.00
0	13	-6	34.20	14.70
0	13	6	-13.40	17.70
0	13	6	23.70	11.50
-1	-13	-6	9.60	14.00
-1	-13	-6	8.00	19.40
1	-13	6	18.50	12.30

-1	13	-6	3.10	17.00
1	13	6	27.30	22.20
-1	13	-6	0.00	13.20
1	13	6	13.50	11.40
2	-13	6	0.50	10.70
-2	-13	-6	-1.90	21.80
-2	-13	-6	6.70	11.70
2	13	6	19.50	12.00
-2	13	-6	26.00	18.70
-2	13	-6	-6.40	13.50
2	13	6	-7.40	19.50
-3	-13	-6	-8.20	10.60
-3	-13	-6	-8.60	20.90
3	-13	6	-1.70	10.40
3	13	6	4.00	20.80
-3	13	-6	1.30	13.60
-3	13	-6	-38.90	19.40
3	13	6	-2.30	9.00
-4	-13	-6	22.40	14.50
-4	-13	-6	-31.70	23.70
-4	13	-6	21.10	21.70
4	13	6	18.40	11.70
4	13	6	7.70	20.00
-4	13	-6	10.00	13.90
5	-13	6	11.00	13.40
-5	-13	-6	-13.90	25.50
-5	-13	-6	-26.60	23.00
-5	13	-6	-12.80	22.80
5	13	6	-6.90	10.20
5	13	6	-3.20	22.50
-5	13	-6	27.90	17.10

4	-14	-6	-12.40	14.40
4	14	-6	-1.60	9.60
-4	14	6	-35.90	11.90
-4	14	6	10.50	25.60
-3	14	6	1.50	11.90
3	14	-6	10.20	17.40
-3	14	6	-39.30	20.50
2	-14	-6	-14.20	13.00
-2	-14	6	22.80	14.60
2	-14	-6	27.90	29.10
-2	14	6	7.50	12.00
-2	14	6	0.70	21.70
2	14	-6	6.10	8.80
1	-14	-6	-3.70	13.00
1	-14	-6	-0.20	24.90
-1	-14	6	12.90	13.30
1	14	-6	17.60	8.50
-1	14	6	17.20	13.20
1	14	-6	3.70	15.90
-1	14	6	11.80	20.30
0	-14	-6	8.20	24.40
0	-14	6	14.40	12.30
0	-14	-6	17.40	14.10
0	14	-6	48.10	15.40
0	14	6	63.29	28.80
0	14	-6	50.69	16.00
0	14	6	32.40	14.20
-1	-14	-6	0.80	21.70
-1	-14	-6	17.20	16.90
1	-14	6	-1.40	13.00
1	14	6	10.80	12.50

-1	14	-6	29.50	14.40
-1	14	-6	-9.90	17.20
1	14	6	-22.40	18.50
2	-14	6	31.10	14.00
-2	-14	-6	6.40	27.10
-2	14	-6	38.00	23.00
2	14	6	32.10	13.60
2	14	6	28.80	24.20
-3	-14	-6	25.00	25.70
-3	-14	-6	9.40	16.80
3	-14	6	-8.50	12.60
-3	14	-6	29.10	27.40
3	14	6	8.90	21.70
3	14	6	-17.20	11.80
4	-14	6	-20.40	13.30
-4	-14	-6	0.90	13.90
-4	-14	-6	-53.69	26.60
4	14	6	-8.40	12.20
-4	14	-6	20.60	26.00
-1	-15	6	8.70	15.50
1	-15	-6	-2.90	17.60
-1	15	6	-8.40	22.20
-1	15	6	32.60	15.30
0	-15	-6	15.00	16.70
0	15	6	-4.10	23.00
0	15	6	23.50	14.20
-1	-15	-6	-43.00	26.80
10	0	-7	-2.60	11.10
10	0	-7	-20.30	14.70
-10	0	7	-9.50	19.00
-10	0	7	-6.60	18.10

-10	0	7	27.80	20.60
-9	0	7	-6.00	14.40
9	0	-7	-6.30	7.10
-9	0	7	0.50	15.30
-9	0	7	6.80	15.40
9	0	-7	-3.40	11.40
-8	0	7	9.70	13.80
8	0	-7	-5.10	5.50
-8	0	7	-31.30	13.00
-8	0	7	-5.40	12.10
-7	0	7	186.98	30.20
-7	0	7	179.68	25.10
7	0	-7	174.38	15.70
-7	0	7	215.48	31.30
-6	0	7	-19.90	10.10
6	0	-7	-9.10	4.70
-6	0	7	22.50	12.20
-5	0	7	5.10	9.00
5	0	-7	1.70	4.90
-5	0	7	6.70	10.00
-5	0	7	29.20	12.50
-4	0	7	-11.50	6.10
4	0	-7	-1.20	4.70
-3	0	7	1515.45	51.79
3	0	-7	1409.06	47.50
3	0	-7	1372.16	57.29
-3	0	7	1436.66	57.39
3	0	-7	1413.76	50.19
2	0	-7	-6.20	4.90
2	0	-7	-2.60	3.00
-2	0	7	8.80	4.10

−2	0	7	−2.80	4.50
2	0	−7	3.10	9.20
−2	0	7	−2.30	5.30
2	0	−7	−0.30	8.30
1	0	−7	230.88	20.70
−1	0	7	254.57	16.40
1	0	−7	265.57	14.50
−1	0	7	281.97	14.30
−1	0	7	292.47	22.60
1	0	−7	274.37	18.70
0	0	7	2.10	6.10
0	0	−7	−3.80	6.00
0	0	−7	3.10	4.60
0	0	7	−0.90	3.30
0	0	−7	−0.40	3.80
0	0	−7	1.90	3.80
0	0	7	−1.10	3.40
0	0	7	−1.30	2.00
−1	0	−7	161.08	13.90
−1	0	−7	115.39	15.90
1	0	7	132.69	9.60
−1	0	−7	136.19	9.10
−1	0	−7	138.69	19.40
−1	0	−7	150.38	10.90
1	0	7	158.08	15.90
1	0	7	106.39	14.70
1	0	7	144.29	10.10
−2	0	−7	−4.70	3.00
2	0	7	0.20	4.40
−2	0	−7	−1.00	3.00
−2	0	−7	−0.80	6.30

-2	0	-7	-7.10	4.50
-2	0	-7	1.20	5.90
2	0	7	1.00	2.60
2	0	7	-2.20	3.60
-2	0	-7	3.10	4.20
-2	0	-7	0.40	5.40
-3	0	-7	20.40	8.50
3	0	7	8.30	5.40
3	0	7	10.70	4.90
-3	0	-7	17.60	4.90
3	0	7	10.00	3.60
-3	0	-7	10.50	7.90
-3	0	-7	24.40	9.40
-3	0	-7	10.40	4.90
4	0	7	2.40	3.10
-4	0	-7	6.10	7.10
-4	0	-7	13.40	9.30
-4	0	-7	-6.20	5.70
-4	0	-7	0.50	3.90
-4	0	-7	-2.00	2.40
5	0	7	91.49	9.30
-5	0	-7	78.89	18.20
5	0	7	85.79	10.10
-5	0	-7	75.39	9.90
-5	0	-7	78.59	14.50
-5	0	-7	85.99	17.40
-5	0	-7	90.99	5.50
6	0	7	4.90	4.20
-6	0	-7	2.90	10.20
-6	0	-7	0.40	7.90
-6	0	-7	3.10	11.30

7	0	7	25.50	7.30
-7	0	-7	31.90	14.60
-7	0	-7	23.50	11.20
8	0	7	-2.30	6.00
-8	0	-7	11.30	14.20
-8	0	-7	5.20	11.00
-9	0	-7	3.50	15.00
-9	0	-7	7.50	15.10
9	0	7	12.20	8.10
-10	0	-7	28.70	25.20
-10	0	-7	-4.60	16.20
10	0	7	2.30	8.80
-10	-1	7	38.80	24.30
-10	-1	7	-13.60	16.70
10	-1	-7	9.00	13.30
-10	-1	7	4.50	19.20
10	-1	-7	8.70	9.80
10	1	-7	11.60	9.60
-10	1	7	3.30	19.40
-10	1	7	36.90	22.40
-10	1	7	0.00	21.70
-9	-1	7	5.80	16.10
-9	-1	7	-2.10	17.10
9	-1	-7	19.20	10.10
9	-1	-7	13.60	12.50
-9	-1	7	15.50	16.10
-9	1	7	26.90	18.50
-9	1	7	-21.70	15.10
9	1	-7	15.60	9.40
-9	1	7	14.30	16.70
-8	-1	7	-1.00	13.80

8	−1	−7	39.40	10.70
−8	−1	7	22.50	15.60
8	−1	−7	6.10	12.30
−8	−1	7	22.30	16.40
8	1	−7	5.80	8.90
−8	1	7	2.60	13.60
−8	1	7	32.80	19.10
−8	1	7	17.50	14.40
7	−1	−7	171.48	15.00
−7	−1	7	209.98	27.40
7	−1	−7	178.28	21.70
−7	−1	7	190.38	28.80
−7	−1	7	186.28	30.10
−7	1	7	258.97	35.60
−7	1	7	244.78	31.20
7	1	−7	175.98	15.50
−7	1	7	178.18	28.00
−6	−1	7	18.70	10.40
6	−1	−7	3.10	8.40
−6	−1	7	−11.20	9.70
6	−1	−7	3.80	5.90
−6	−1	7	9.40	10.30
−6	1	7	−12.40	8.50
6	1	−7	−0.10	6.20
−6	1	7	−1.00	10.40
−6	1	7	−10.90	10.00
−5	−1	7	−0.70	9.40
−5	−1	7	14.70	9.30
−5	−1	7	9.20	9.50
5	−1	−7	8.60	5.00
−5	1	7	15.70	9.90

-5	1	7	-3.40	9.30
5	1	-7	15.00	6.50
-5	1	7	22.60	11.00
-4	-1	7	65.89	14.40
-4	-1	7	83.39	13.90
4	-1	-7	81.49	14.80
4	-1	-7	73.29	11.00
-4	1	7	68.69	15.40
-4	1	7	65.09	14.50
4	1	-7	59.49	8.90
3	-1	-7	14.40	7.30
-3	-1	7	18.90	9.30
3	-1	-7	16.20	6.20
-3	-1	7	33.30	8.40
3	-1	-7	26.60	10.00
3	1	-7	32.30	12.00
-3	1	7	17.30	8.30
3	1	-7	18.40	4.90
3	1	-7	22.00	6.90
-3	1	7	14.00	7.90
2	-1	-7	275.57	21.60
2	-1	-7	293.77	23.30
2	-1	-7	243.28	15.10
2	-1	-7	255.37	18.30
-2	-1	7	259.17	14.90
2	-1	-7	262.37	23.80
-2	-1	7	241.98	12.90
-2	1	7	273.97	23.20
2	1	-7	264.77	21.60
2	1	-7	227.48	16.60
2	1	-7	261.27	14.10

-2	1	7	235.68	13.90
-1	-1	7	47.70	7.40
-1	-1	7	33.80	9.20
1	-1	-7	40.10	10.30
1	-1	-7	45.50	7.70
1	-1	-7	42.10	9.20
-1	-1	7	42.40	5.90
1	-1	-7	37.70	11.70
-1	-1	7	39.00	5.60
-1	1	7	38.60	10.90
1	1	-7	33.00	8.40
-1	1	7	33.50	6.30
1	1	-7	57.39	7.70
-1	1	7	43.20	6.80
1	1	-7	43.30	9.80
0	-1	-7	101.39	15.90
0	-1	7	108.89	7.60
0	-1	7	92.79	9.30
0	-1	7	91.39	6.80
0	-1	-7	114.39	14.00
0	-1	-7	123.89	13.40
0	-1	-7	129.69	10.30
0	-1	7	120.69	16.80
0	1	-7	109.99	15.50
0	1	7	107.79	9.00
0	1	7	93.39	15.10
0	1	7	99.49	8.30
0	1	-7	107.09	9.90
-1	-1	-7	1291.67	44.20
-1	-1	-7	1413.06	54.89
-1	-1	-7	1312.67	37.70

-1	-1	-7	1280.77	51.29
1	-1	7	1336.77	48.20
1	-1	7	1398.36	36.00
-1	-1	-7	1336.57	40.90
-1	-1	-7	1404.96	47.30
1	-1	7	1291.77	43.30
1	-1	7	1344.27	34.50
1	1	7	1453.55	53.29
-1	1	-7	1377.46	43.40
1	1	7	1377.66	47.40
1	1	7	1347.07	37.80
1	1	7	1383.56	42.60
-1	1	-7	1337.37	49.40
2	-1	7	5.10	3.30
-2	-1	-7	7.60	5.00
2	-1	7	17.50	6.70
-2	-1	-7	9.80	8.70
-2	-1	-7	6.60	6.40
-2	-1	-7	16.00	8.30
-2	-1	-7	5.50	4.00
2	-1	7	7.50	2.70
-2	-1	-7	19.00	9.00
-2	-1	-7	6.90	4.60
-2	1	-7	4.10	5.20
2	1	7	3.50	4.40
-2	1	-7	24.30	10.80
-2	1	-7	15.40	5.50
-2	1	-7	7.20	3.40
-2	1	-7	2.10	6.80
2	1	7	9.70	5.80
2	1	7	8.80	3.80

−2	1	−7	9.00	3.80
3	−1	7	102.89	8.20
−3	−1	−7	88.19	15.20
−3	−1	−7	80.19	13.60
−3	−1	−7	100.39	11.30
−3	−1	−7	129.89	10.00
−3	−1	−7	91.19	19.00
3	−1	7	107.89	6.70
−3	1	−7	88.79	12.90
3	1	7	104.89	15.60
−3	1	−7	89.79	8.70
3	1	7	88.29	9.80
−3	1	−7	101.79	16.80
−3	1	−7	108.09	15.50
3	1	7	99.19	8.00
−3	1	−7	97.09	9.00
−3	1	−7	95.09	8.10
4	−1	7	63.99	8.20
−4	−1	−7	63.79	7.40
−4	−1	−7	71.09	13.50
−4	−1	−7	75.29	9.80
−4	−1	−7	66.19	15.30
4	1	7	65.29	7.80
−4	1	−7	77.79	13.60
−4	1	−7	72.69	12.90
−4	1	−7	64.99	15.80
4	1	7	76.89	9.90
−4	1	−7	66.69	5.80
−4	1	−7	70.89	9.20
5	−1	7	412.66	15.80
−5	−1	−7	448.86	30.20

−5	−1	−7	420.86	15.50
−5	−1	−7	388.06	29.80
−5	−1	−7	388.06	30.70
5	−1	7	398.76	17.60
−5	−1	−7	405.56	20.50
−5	1	−7	402.86	32.00
5	1	7	422.26	18.20
−5	1	−7	455.75	27.10
−5	1	−7	384.86	27.50
−6	−1	−7	15.50	9.60
−6	−1	−7	19.60	3.60
6	−1	7	23.50	5.40
−6	−1	−7	14.30	10.10
−6	1	−7	19.50	13.60
−6	1	−7	8.10	9.10
6	1	7	2.30	4.30
−6	1	−7	35.00	12.60
−7	−1	−7	67.39	18.50
7	−1	7	95.49	11.30
−7	−1	−7	86.19	19.10
7	−1	7	88.39	11.50
7	1	7	93.39	10.90
−7	1	−7	100.59	18.80
−7	1	−7	95.89	20.70
8	−1	7	9.80	5.90
−8	−1	−7	2.70	14.00
−8	−1	−7	−5.10	11.50
−8	1	−7	3.20	10.40
8	1	7	−5.50	6.40
−8	1	−7	0.70	14.40
9	−1	7	−3.20	6.90

-9	-1	-7	3.00	13.00
-9	-1	-7	14.80	14.20
9	1	7	0.40	7.00
-9	1	-7	-4.10	12.90
-9	1	-7	17.50	18.00
-10	-1	-7	12.80	24.70
-10	-1	-7	-38.10	16.00
10	-1	7	-6.80	9.00
-10	1	-7	-4.50	14.70
-10	1	-7	6.00	19.70
10	1	7	-1.60	9.80
-10	-2	7	-2.20	18.70
10	-2	-7	2.80	13.40
10	-2	-7	10.80	10.80
-10	-2	7	14.00	19.00
-10	-2	7	-3.40	20.30
-10	2	7	21.00	21.10
-10	2	7	11.90	18.20
-10	2	7	14.30	19.70
10	2	-7	4.40	8.90
9	-2	-7	-3.00	11.10
-9	-2	7	6.30	13.00
-9	-2	7	19.00	17.10
-9	-2	7	19.70	16.30
9	-2	-7	-3.10	8.90
-9	2	7	21.10	17.10
-9	2	7	18.90	16.00
-9	2	7	9.10	17.60
9	2	-7	-2.10	8.60
-8	-2	7	10.20	14.10
-8	-2	7	28.50	17.80

-8	-2	7	-10.20	10.70
8	-2	-7	15.50	10.00
8	-2	-7	-1.00	7.80
-8	2	7	5.10	14.20
-8	2	7	8.90	14.40
8	2	-7	0.80	6.40
-8	2	7	15.50	14.60
7	-2	-7	15.60	8.70
-7	-2	7	19.50	12.30
7	-2	-7	-0.40	5.90
-7	-2	7	12.20	13.90
7	2	-7	5.70	6.10
-7	2	7	22.40	13.80
-7	2	7	20.10	14.10
-7	2	7	1.10	10.40
6	-2	-7	30.90	8.40
-6	-2	7	15.70	13.50
6	-2	-7	26.50	9.00
-6	-2	7	22.10	9.80
-6	-2	7	11.60	10.60
-6	2	7	26.10	12.10
6	2	-7	24.60	7.80
-6	2	7	31.20	12.50
-6	2	7	25.10	13.20
-5	-2	7	139.39	18.60
5	-2	-7	112.39	14.20
-5	-2	7	127.69	19.30
5	-2	-7	148.89	19.40
-5	-2	7	140.59	23.70
-5	2	7	140.19	22.90
-5	2	7	139.29	21.70

5	2	−7	152.68	12.60
−5	2	7	117.99	20.90
4	−2	−7	11.10	5.90
−4	−2	7	10.70	6.70
4	−2	−7	15.80	8.40
−4	−2	7	4.70	9.00
−4	2	7	3.20	7.90
4	2	−7	7.80	9.80
−4	2	7	9.40	10.10
4	2	−7	13.80	5.30
−3	−2	7	1704.33	61.59
3	−2	−7	1651.33	63.99
3	−2	−7	1694.33	57.59
−3	−2	7	1632.44	45.80
3	−2	−7	1713.83	50.89
−3	−2	7	1693.43	50.39
−3	2	7	1715.43	64.49
−3	2	7	1719.33	66.29
3	2	−7	1772.62	48.90
2	−2	−7	7.40	5.90
2	−2	−7	13.30	5.60
2	−2	−7	4.60	8.80
2	−2	−7	16.80	8.40
−2	−2	7	10.40	3.10
−2	−2	7	11.60	4.00
−2	−2	7	8.20	6.20
2	−2	−7	19.10	8.70
2	2	−7	8.00	7.80
−2	2	7	9.40	7.80
2	2	−7	6.60	4.90
−2	2	7	20.60	9.10

2	2	-7	13.80	4.40
1	-2	-7	13.50	6.80
-1	-2	7	5.30	3.10
-1	-2	7	9.30	2.90
1	-2	-7	-9.10	7.80
1	-2	-7	15.40	5.70
-1	-2	7	11.20	6.10
-1	-2	7	7.50	3.20
1	-2	-7	5.80	7.00
-1	2	7	9.50	7.00
1	2	-7	8.30	6.70
1	2	-7	11.00	4.10
-1	2	7	13.00	8.30
-1	2	7	11.10	4.40
-1	2	7	10.40	4.70
1	2	-7	5.20	4.90
0	-2	-7	121.99	10.90
0	-2	7	104.39	8.70
0	-2	7	112.19	6.60
0	-2	-7	130.89	17.00
0	-2	7	104.99	6.80
0	-2	7	88.29	13.20
0	-2	-7	109.49	14.00
0	-2	-7	109.39	17.00
0	2	-7	114.89	8.60
0	2	7	107.89	15.20
0	2	-7	96.69	13.70
0	2	7	109.09	9.60
0	2	7	138.19	19.40
0	2	-7	102.89	9.40
-1	-2	-7	1503.95	51.59

−1	−2	−7	1424.86	55.69
−1	−2	−7	1395.56	47.00
1	−2	7	1449.26	34.70
1	−2	7	1463.45	49.30
−1	−2	−7	1489.85	44.40
1	−2	7	1473.05	35.20
−1	2	−7	1510.85	50.49
−1	2	−7	1466.15	44.50
1	2	7	1528.65	42.00
−1	2	−7	1420.46	40.40
1	2	7	1440.26	57.09
1	2	7	1505.75	43.10
1	2	7	1424.06	51.29
−1	2	−7	1458.55	38.20
−2	−2	−7	54.49	8.80
−2	−2	−7	55.79	11.00
−2	−2	−7	43.20	12.00
2	−2	7	54.19	6.60
−2	−2	−7	37.00	11.40
−2	−2	−7	51.59	8.30
2	−2	7	48.40	9.00
2	−2	7	50.49	4.00
−2	2	−7	61.19	6.20
−2	2	−7	54.39	9.20
2	2	7	32.30	9.10
2	2	7	66.99	8.60
2	2	7	57.09	11.30
−2	2	−7	49.50	9.70
2	2	7	58.89	7.80
−2	2	−7	68.59	13.90
−2	2	−7	42.80	6.70

−3	−2	−7	287.47	24.30
3	−2	7	273.37	10.20
−3	−2	−7	278.87	22.60
3	2	7	264.37	21.90
−3	2	−7	259.07	13.40
3	2	7	260.77	16.60
−3	2	−7	259.37	24.50
−3	2	−7	272.37	12.30
3	2	7	254.17	13.90
−3	2	−7	256.67	17.20
−3	2	−7	259.27	19.70
4	−2	7	−0.90	2.00
−4	−2	−7	−11.10	8.70
−4	−2	−7	3.30	3.90
4	−2	7	4.30	2.70
−4	−2	−7	−0.90	7.20
−4	−2	−7	−1.80	6.30
−4	−2	−7	1.40	4.60
4	2	7	11.10	8.30
−4	2	−7	6.70	5.40
−4	2	−7	22.00	10.10
−4	2	−7	7.90	6.50
−4	2	−7	4.40	3.80
4	2	7	1.20	5.20
−4	2	−7	−1.40	2.60
−4	2	−7	−2.10	3.30
4	2	7	−5.10	3.30
5	−2	7	47.70	4.90
−5	−2	−7	54.39	14.90
−5	−2	−7	42.20	13.10
−5	−2	−7	33.70	7.90

5	−2	7	41.30	5.80
−5	−2	−7	33.70	6.30
−5	2	−7	39.00	12.10
−5	2	−7	48.80	15.10
−5	2	−7	42.20	7.40
5	2	7	55.99	8.40
−5	2	−7	38.70	10.50
5	2	7	44.10	7.30
6	−2	7	3.80	3.40
6	−2	7	0.30	5.20
−6	−2	−7	5.40	10.00
−6	−2	−7	6.50	8.90
−6	−2	−7	5.10	4.10
6	2	7	11.70	6.40
−6	2	−7	6.30	11.30
−6	2	−7	−1.50	8.50
−6	2	−7	6.00	7.40
6	2	7	6.20	4.70
7	−2	7	−0.60	4.10
−7	−2	−7	−3.50	12.80
−7	−2	−7	5.40	3.60
7	−2	7	5.70	5.70
−7	−2	−7	−2.20	10.50
7	2	7	5.60	5.70
−7	2	−7	−2.70	11.70
−7	2	−7	−10.50	10.90
−7	2	−7	−4.10	8.50
7	2	7	−0.10	6.00
−8	−2	−7	5.40	12.40
−8	−2	−7	−5.90	11.10
8	−2	7	0.90	5.00

8	-2	7	6.80	8.30
-8	2	-7	-18.80	11.60
8	2	7	0.70	6.30
-8	2	-7	8.20	11.70
9	-2	7	49.70	8.30
-9	-2	-7	47.40	19.80
-9	-2	-7	38.30	20.40
-9	2	-7	46.30	16.90
9	2	7	12.50	8.40
-9	2	-7	42.20	20.70
-10	-2	-7	0.80	35.80
-10	2	-7	3.30	16.50
-10	2	-7	-4.60	24.50
10	2	7	-4.70	9.70
10	-3	-7	0.60	15.80
-10	-3	7	16.80	17.40
-10	-3	7	-1.20	22.20
10	-3	-7	-0.20	11.50
-10	-3	7	37.70	39.00
-10	3	7	5.60	18.20
10	3	-7	2.90	11.10
-10	3	7	2.10	25.60
-10	3	7	0.10	22.90
-9	-3	7	17.20	17.80
-9	-3	7	24.30	16.70
-9	-3	7	37.40	16.60
9	-3	-7	3.90	12.00
9	-3	-7	-3.00	10.10
-9	3	7	6.50	15.80
-9	3	7	10.40	15.80
9	3	-7	18.50	10.50

-9	3	7	23.00	18.80
8	-3	-7	-2.10	10.20
-8	-3	7	-9.40	21.00
-8	-3	7	2.70	12.10
8	-3	-7	-0.10	6.90
-8	-3	7	1.30	15.70
8	3	-7	-4.00	6.00
-8	3	7	-6.10	14.80
-8	3	7	-9.40	11.20
7	-3	-7	135.19	16.00
-7	-3	7	91.69	19.40
-7	-3	7	105.09	31.00
-7	-3	7	102.39	18.70
7	-3	-7	92.19	17.80
-7	3	7	164.88	29.30
-7	3	7	120.79	23.70
7	3	-7	128.69	15.60
-7	3	7	155.58	27.40
-6	-3	7	11.20	11.60
6	-3	-7	19.20	8.20
6	-3	-7	3.30	9.20
-6	-3	7	19.00	9.40
-6	-3	7	8.80	12.80
-6	3	7	5.10	10.80
-6	3	7	25.50	13.00
-6	3	7	29.10	13.10
6	3	-7	15.20	6.60
5	-3	-7	27.10	10.50
-5	-3	7	45.40	13.20
-5	-3	7	30.50	12.70
5	-3	-7	29.80	8.90

−5	−3	7	19.10	7.60
−5	3	7	30.10	12.20
−5	3	7	17.90	13.40
−5	3	7	40.90	14.60
5	3	−7	30.40	7.50
4	−3	−7	13.40	8.60
4	−3	−7	7.70	5.00
−4	−3	7	9.20	9.20
−4	3	7	5.40	10.70
4	3	−7	0.30	10.60
−4	3	7	0.40	8.80
4	3	−7	5.70	5.20
−3	−3	7	27.60	10.70
−3	−3	7	12.40	4.10
3	−3	−7	15.00	7.90
−3	−3	7	19.30	3.60
3	−3	−7	24.10	11.90
3	−3	−7	7.10	5.50
3	3	−7	20.70	5.40
−3	3	7	15.60	10.40
−3	3	7	29.20	10.70
3	3	−7	13.00	9.20
2	−3	−7	61.39	16.50
2	−3	−7	76.49	13.10
2	−3	−7	64.79	17.90
−2	−3	7	59.79	6.60
−2	−3	7	54.59	11.40
2	3	−7	73.69	8.60
−2	3	7	61.49	14.30
−2	3	7	63.79	14.50
2	3	−7	66.89	11.40

-1	-3	7	7.70	2.60
1	-3	-7	10.10	9.20
1	-3	-7	7.90	4.70
-1	-3	7	9.50	3.10
1	-3	-7	7.80	6.50
-1	-3	7	12.20	6.30
1	-3	-7	0.60	8.50
1	3	-7	8.10	3.70
-1	3	7	10.00	7.40
-1	3	7	1.60	8.10
1	3	-7	15.30	7.20
-1	3	7	1.00	3.80
0	-3	7	177.88	9.50
0	-3	7	157.98	15.60
0	-3	-7	198.18	24.10
0	-3	-7	165.48	14.60
0	-3	-7	173.78	18.50
0	-3	-7	168.78	12.60
0	3	-7	175.88	10.90
0	3	7	167.08	16.40
0	3	7	173.98	20.90
0	3	-7	180.78	10.10
0	3	7	184.18	12.60
0	3	7	195.88	24.30
-1	-3	-7	841.32	34.60
1	-3	7	847.22	33.80
-1	-3	-7	924.61	41.40
-1	-3	-7	846.72	42.00
-1	-3	-7	898.61	31.90
-1	3	-7	884.51	25.30
1	3	7	911.21	40.10

−1	3	−7	870.91	29.60
1	3	7	871.91	29.90
1	3	7	841.72	34.30
−1	3	−7	904.51	30.90
−1	3	−7	860.11	26.80
−1	3	−7	905.61	33.50
1	3	7	858.91	43.00
−2	−3	−7	10.70	7.20
−2	−3	−7	1.80	3.80
−2	−3	−7	−3.90	5.80
−2	−3	−7	9.70	10.00
−2	3	−7	3.10	5.50
−2	3	−7	2.50	3.30
−2	3	−7	0.90	3.90
−2	3	−7	5.00	2.40
2	3	7	4.80	4.40
2	3	7	−10.10	5.00
−2	3	−7	4.20	3.40
−3	−3	−7	52.59	16.40
−3	−3	−7	80.69	10.10
−3	−3	−7	64.79	15.50
−3	−3	−7	69.39	9.90
−3	3	−7	75.09	12.00
−3	3	−7	76.29	6.60
−3	3	−7	63.09	13.70
−3	3	−7	71.99	7.50
3	3	7	67.79	9.00
−3	3	−7	70.89	9.50
−3	3	−7	74.39	6.70
3	3	7	70.29	14.60
3	3	7	83.89	11.90

−3	3	−7	74.29	15.20
−4	−3	−7	47.70	12.40
−4	−3	−7	55.99	9.20
−4	−3	−7	37.10	12.70
−4	−3	−7	62.89	9.10
−4	3	−7	60.79	7.00
4	3	7	41.10	11.90
−4	3	−7	56.09	9.60
−4	3	−7	66.89	12.50
−4	3	−7	47.30	13.30
4	3	7	52.29	9.30
−4	3	−7	61.19	6.20
−4	3	−7	65.49	5.20
4	3	7	50.59	8.00
−4	3	−7	67.29	8.70
−5	−3	−7	242.98	26.10
−5	−3	−7	229.38	28.00
−5	−3	−7	274.37	15.10
−5	−3	−7	244.48	16.50
−5	3	−7	277.07	14.20
5	3	7	236.08	15.00
−5	3	−7	290.17	26.90
5	3	7	225.98	22.50
−5	3	−7	280.87	19.10
5	3	7	304.37	20.60
−6	−3	−7	12.30	5.10
−6	−3	−7	15.50	11.50
−6	−3	−7	26.00	13.30
−6	3	−7	5.30	10.20
−6	3	−7	20.90	9.50
6	3	7	20.10	6.30

6	3	7	20.90	9.10
−6	3	−7	9.10	7.60
−7	−3	−7	19.30	13.30
−7	−3	−7	35.80	16.90
−7	3	−7	25.50	11.70
−7	3	−7	35.60	11.60
7	3	7	42.20	9.20
−7	3	−7	51.29	20.10
7	3	7	54.59	11.90
−8	−3	−7	12.30	14.00
−8	−3	−7	24.80	14.60
−8	3	−7	−13.20	13.00
−8	3	−7	10.30	15.00
−9	−3	−7	1.70	18.00
−9	−3	−7	31.40	17.40
−9	3	−7	−5.10	15.00
9	3	7	1.50	8.30
−9	3	−7	−12.00	11.60
−10	−3	−7	−2.10	19.60
10	3	7	−3.20	10.00
−10	3	−7	−2.40	14.10
−10	−4	7	37.50	19.80
−10	−4	7	−25.30	24.50
10	−4	−7	−5.60	15.10
−10	−4	7	53.59	24.00
10	−4	−7	−2.00	11.80
−10	4	7	4.90	22.10
−10	4	7	9.00	19.10
10	4	−7	29.00	11.50
−10	4	7	2.80	21.70
−9	−4	7	0.40	18.10

-9	-4	7	19.80	14.20
9	-4	-7	-0.20	10.10
-9	-4	7	12.50	17.80
9	-4	-7	-7.30	11.90
-9	4	7	9.20	15.10
-9	4	7	11.10	15.90
9	4	-7	4.50	8.70
-9	4	7	7.00	16.10
8	-4	-7	7.60	12.20
-8	-4	7	26.70	12.40
-8	-4	7	13.60	18.20
8	-4	-7	2.60	9.50
8	4	-7	18.80	8.00
-8	4	7	0.40	15.80
-8	4	7	9.90	17.10
-8	4	7	12.10	14.60
-7	-4	7	66.69	21.80
-7	-4	7	78.19	14.40
7	-4	-7	77.09	17.40
-7	-4	7	79.59	15.10
7	-4	-7	100.99	14.70
-7	-4	7	79.89	23.90
-7	4	7	106.29	24.30
-7	4	7	43.20	16.80
7	4	-7	67.29	13.50
-7	4	7	77.99	21.20
-6	-4	7	33.00	9.20
-6	-4	7	15.90	14.60
-6	-4	7	13.80	10.60
6	-4	-7	17.30	8.10
-6	-4	7	0.90	7.30

6	−4	−7	11.60	11.60
−6	4	7	22.10	12.00
−6	4	7	17.20	12.60
−6	4	7	35.30	16.60
6	4	−7	9.70	6.00
5	−4	−7	58.59	14.40
−5	−4	7	42.30	13.80
5	−4	−7	26.40	8.80
−5	−4	7	29.90	8.10
−5	−4	7	64.39	18.50
−5	−4	7	56.79	7.90
5	4	−7	41.50	8.10
−5	4	7	36.70	12.50
−5	4	7	46.30	18.30
−5	4	7	13.90	11.30
−4	−4	7	2.80	2.60
4	−4	−7	−7.00	5.70
4	−4	−7	7.70	6.80
−4	−4	7	9.80	8.90
4	4	−7	10.10	10.70
−4	4	7	16.10	10.10
−4	4	7	5.20	8.30
4	4	−7	−1.30	3.90
−3	−4	7	400.06	16.30
3	−4	−7	426.46	27.40
3	−4	−7	389.26	21.20
3	−4	−7	352.56	25.30
3	4	−7	412.46	27.30
−3	4	7	389.26	31.90
−3	4	7	424.26	32.40
3	4	−7	419.86	18.80

2	-4	-7	35.90	10.60
2	-4	-7	15.60	5.70
-2	-4	7	26.40	4.20
-2	-4	7	24.20	8.30
2	-4	-7	30.40	8.80
2	4	-7	31.40	9.70
-2	4	7	26.50	12.10
2	4	-7	21.20	5.40
1	-4	-7	5.90	4.50
-1	-4	7	10.80	2.60
-1	-4	7	20.00	3.00
-1	-4	7	18.10	6.70
1	-4	-7	8.80	6.40
-1	4	7	9.20	10.30
-1	4	7	17.70	9.00
-1	4	7	19.20	5.90
1	4	-7	7.50	5.10
0	-4	7	117.79	14.20
0	-4	-7	109.29	13.40
0	-4	-7	133.79	17.30
0	-4	-7	94.89	14.50
0	4	7	113.99	19.80
0	4	7	101.19	12.70
0	4	-7	91.49	7.70
0	4	-7	130.39	13.90
0	4	-7	111.29	7.60
0	4	-7	102.69	10.30
0	4	7	138.99	20.10
-1	-4	-7	43.50	9.90
-1	-4	-7	38.80	7.30
-1	-4	-7	60.09	13.20

-1	4	-7	50.79	6.10
1	4	7	39.10	11.30
-1	4	-7	55.59	8.50
-1	4	-7	43.60	7.60
1	4	7	54.19	8.60
1	4	7	30.90	11.60
-1	4	-7	39.40	7.40
-1	4	-7	58.09	5.90
-2	-4	-7	14.90	6.40
-2	-4	-7	17.70	7.10
-2	-4	-7	8.80	7.50
-2	-4	-7	8.50	8.80
2	4	7	9.70	7.20
-2	4	-7	9.70	4.00
-2	4	-7	17.30	4.30
-2	4	-7	12.00	4.60
-2	4	-7	20.20	4.80
-2	4	-7	13.90	8.00
-2	4	-7	14.40	3.40
2	4	7	12.50	5.30
-3	-4	-7	20.70	9.40
-3	-4	-7	33.20	7.80
-3	-4	-7	24.10	6.20
-3	-4	-7	20.40	10.90
-3	4	-7	29.50	5.20
-3	4	-7	31.30	5.40
-3	4	-7	43.10	12.80
-3	4	-7	37.80	7.70
3	4	7	27.60	9.00
-3	4	-7	26.60	4.30
-3	4	-7	44.10	6.30

3	4	7	18.00	5.20
-4	-4	-7	36.20	8.30
-4	-4	-7	49.00	14.40
-4	-4	-7	48.50	8.20
-4	-4	-7	63.99	14.90
-4	4	-7	62.09	9.00
4	4	7	60.99	11.00
-4	4	-7	40.50	9.30
4	4	7	42.60	8.10
-4	4	-7	51.99	6.60
-4	4	-7	61.49	7.30
4	4	7	32.40	11.40
-4	4	-7	48.00	15.10
-4	4	-7	51.89	5.30
-5	-4	-7	20.00	5.90
-5	-4	-7	20.10	7.90
-5	-4	-7	8.60	10.40
-5	-4	-7	1.70	10.40
-5	4	-7	19.70	5.20
-5	4	-7	17.30	6.30
5	4	7	5.20	5.20
5	4	7	0.40	9.20
-5	4	-7	13.70	7.80
5	4	7	0.60	8.30
-5	4	-7	14.80	12.20
-6	-4	-7	0.20	10.90
-6	-4	-7	5.80	10.40
-6	-4	-7	-5.40	5.20
6	4	7	-5.00	8.90
6	4	7	12.00	7.20
-6	4	-7	7.10	7.20

−6	4	−7	−4.50	10.30
−6	4	−7	9.60	8.10
−7	−4	−7	20.60	16.60
−7	−4	−7	48.20	15.10
−7	−4	−7	25.80	7.20
−7	4	−7	20.10	10.10
−7	4	−7	24.00	12.40
7	4	7	22.70	8.50
7	4	7	22.10	11.30
−8	−4	−7	0.30	14.70
−8	−4	−7	19.90	7.50
−8	−4	−7	14.60	15.70
8	4	7	0.40	6.30
−8	4	−7	5.10	16.00
−8	4	−7	4.00	9.80
−9	−4	−7	−7.50	16.70
−9	−4	−7	−8.90	17.90
9	4	7	0.50	8.00
−9	4	−7	−14.40	13.90
−9	4	−7	6.50	12.50
−10	−4	−7	6.00	21.50
−10	−4	−7	−8.30	21.90
−10	4	−7	15.40	15.60
10	4	7	−3.20	11.00
−10	4	−7	−6.50	29.10
10	−5	−7	2.30	12.40
10	−5	−7	7.30	15.90
−10	−5	7	36.30	21.10
−10	−5	7	35.40	23.30
−10	−5	7	7.60	24.10
−10	5	7	1.50	22.00

-10	5	7	21.00	20.90
10	5	-7	9.90	11.60
-10	5	7	-10.90	20.90
9	-5	-7	25.00	11.20
-9	-5	7	-4.50	15.70
-9	-5	7	-14.40	18.60
9	-5	-7	4.70	13.50
-9	-5	7	-4.40	17.70
-9	5	7	20.30	21.00
9	5	-7	23.70	11.20
-9	5	7	16.10	17.50
-9	5	7	16.90	21.60
-8	-5	7	20.20	17.90
-8	-5	7	48.90	12.40
8	-5	-7	18.20	10.80
-8	5	7	78.39	25.20
8	5	-7	55.89	11.40
-8	5	7	35.00	19.60
-8	5	7	14.60	16.10
-7	-5	7	62.69	19.90
7	-5	-7	29.10	9.80
7	-5	-7	36.70	11.50
-7	-5	7	57.59	20.10
-7	-5	7	24.60	10.50
-7	5	7	38.80	18.60
-7	5	7	62.79	25.30
-7	5	7	53.29	17.50
7	5	-7	13.00	7.40
-6	-5	7	9.00	12.60
6	-5	-7	6.00	9.90
6	-5	-7	0.80	7.20

−6	−5	7	−8.80	7.00
−6	−5	7	−14.30	6.20
−6	−5	7	0.70	12.50
−6	5	7	12.80	11.70
−6	5	7	11.90	14.10
−6	5	7	0.40	11.00
6	5	−7	4.50	6.10
−5	−5	7	80.69	19.40
5	−5	−7	83.99	13.60
−5	−5	7	85.19	18.50
−5	−5	7	90.99	9.00
5	−5	−7	109.39	17.70
−5	5	7	77.69	21.60
−5	5	7	87.59	19.90
5	5	−7	70.49	10.80
−4	−5	7	241.88	24.00
4	−5	−7	234.68	17.00
4	−5	−7	240.58	21.60
4	5	−7	232.18	15.20
−4	5	7	210.68	28.80
−4	5	7	176.28	27.10
4	5	−7	208.48	25.10
−3	−5	7	1.50	7.50
3	−5	−7	−3.50	4.50
3	−5	−7	9.00	8.20
−3	−5	7	1.60	3.70
3	−5	−7	2.70	7.10
3	5	−7	−4.80	3.50
−3	5	7	−1.20	9.50
3	5	−7	−15.00	7.10
−3	5	7	6.50	9.70

2	−5	−7	299.27	23.10
2	−5	−7	326.27	18.90
−2	−5	7	342.27	23.00
−2	−5	7	315.37	11.70
−2	5	7	330.17	29.00
2	5	−7	286.47	20.50
−2	5	7	299.37	28.90
2	5	−7	289.97	14.40
1	−5	−7	183.38	17.00
1	−5	−7	176.48	17.40
1	−5	−7	177.38	13.70
−1	−5	7	183.18	16.60
1	5	−7	160.98	10.90
−1	5	7	164.78	24.40
−1	5	7	177.68	14.00
−1	5	7	236.38	29.20
1	5	−7	159.68	14.30
0	−5	−7	17.20	6.40
0	−5	−7	10.30	8.10
0	−5	−7	14.30	6.90
0	−5	7	−2.40	4.60
0	5	−7	19.90	4.00
0	5	−7	14.70	5.20
0	5	−7	16.20	4.50
0	5	7	26.20	10.90
0	5	7	17.00	6.60
0	5	−7	12.30	4.70
0	5	7	27.20	12.60
−1	−5	−7	67.09	10.00
−1	−5	−7	78.99	12.90
−1	−5	−7	58.39	14.40

−1	−5	−7	78.59	14.70
−1	5	−7	67.89	9.30
−1	5	−7	60.69	6.20
1	5	7	71.39	10.20
1	5	7	81.79	16.30
−1	5	−7	71.99	9.80
−1	5	−7	75.49	7.60
1	5	7	69.89	15.60
−2	−5	−7	41.70	9.40
−2	−5	−7	59.59	15.40
−2	−5	−7	36.10	7.80
−2	−5	−7	32.80	9.80
−2	5	−7	45.50	4.70
2	5	7	15.10	8.10
−2	5	−7	41.40	13.00
−2	5	−7	32.10	7.70
−2	5	−7	45.00	11.10
2	5	7	40.20	8.40
−2	5	−7	45.70	4.70
−2	5	−7	48.80	4.30
−3	−5	−7	14.00	10.30
−3	−5	−7	14.40	10.40
−3	−5	−7	31.90	7.90
−3	−5	−7	10.30	8.10
−3	5	−7	15.30	10.00
3	5	7	27.10	10.20
−3	5	−7	17.90	4.00
−3	5	−7	16.60	4.90
3	5	7	23.50	6.50
−3	5	−7	20.00	3.60
−4	−5	−7	51.79	10.00

−4	−5	−7	73.89	12.70
−4	−5	−7	81.99	17.80
−4	−5	−7	105.79	18.60
4	5	7	70.29	17.30
−4	5	−7	69.79	7.00
4	5	7	82.39	11.40
−4	5	−7	76.89	9.60
−4	5	−7	83.19	17.20
−4	5	−7	72.09	7.20
−4	5	−7	66.19	7.10
−4	5	−7	78.89	7.30
−5	−5	−7	59.89	10.70
−5	−5	−7	56.59	15.60
−5	−5	−7	32.00	13.20
−5	5	−7	48.00	7.70
5	5	7	36.80	8.30
5	5	7	47.10	14.00
−5	5	−7	47.40	10.20
−5	5	−7	41.20	9.30
−5	5	−7	41.80	6.50
−5	5	−7	50.69	16.20
−6	−5	−7	13.70	13.50
−6	−5	−7	10.40	13.10
−6	−5	−7	16.80	6.60
−6	5	−7	19.30	8.20
−6	5	−7	10.30	13.90
6	5	7	25.50	13.80
−6	5	−7	29.40	8.10
6	5	7	14.30	8.00
−7	−5	−7	−4.30	6.50
−7	−5	−7	5.10	13.70

−7	5	−7	2.80	8.70
−7	5	−7	−4.00	8.90
−7	5	−7	3.60	12.90
7	5	7	7.30	6.80
−8	−5	−7	27.40	17.70
−8	−5	−7	−5.60	7.10
−8	−5	−7	1.90	14.00
8	5	7	10.00	8.60
−8	5	−7	13.50	14.00
−8	5	−7	−15.00	9.30
−9	−5	−7	19.20	17.90
−9	−5	−7	31.70	19.60
9	5	7	−1.00	9.20
−9	5	−7	9.40	17.30
−9	5	−7	6.60	12.10
−10	−5	−7	−5.80	21.70
−10	−5	−7	−26.60	22.30
−10	5	−7	16.60	19.80
−10	5	−7	30.70	16.30
10	5	7	−8.80	12.30
10	−6	−7	5.20	14.90
−10	−6	7	−36.10	20.90
10	−6	−7	4.60	17.20
−10	−6	7	13.80	22.10
−10	6	7	−0.40	25.30
10	6	−7	1.90	12.40
−10	6	7	10.30	20.90
−10	6	7	−5.70	21.70
−9	−6	7	11.30	23.10
9	−6	−7	9.00	11.80
9	−6	−7	4.70	14.80

-9	-6	7	7.20	16.00
-9	-6	7	10.20	20.70
-9	6	7	-22.40	17.20
-9	6	7	50.29	24.90
9	6	-7	42.80	13.30
-9	6	7	32.70	20.40
-8	-6	7	37.30	21.40
8	-6	-7	-15.30	12.20
-8	-6	7	39.60	19.90
-8	-6	7	6.80	11.80
8	-6	-7	12.70	10.90
-8	6	7	-6.60	14.40
8	6	-7	-0.60	7.70
-8	6	7	20.00	18.30
-8	6	7	10.80	17.80
7	-6	-7	7.90	8.80
-7	-6	7	-8.30	13.70
7	-6	-7	-19.50	10.50
-7	-6	7	14.20	8.50
-7	-6	7	-4.50	17.20
-7	-6	7	-0.90	10.00
-7	6	7	19.00	12.90
-7	6	7	-5.40	13.30
-7	6	7	-0.40	15.60
7	6	-7	-2.70	7.10
-6	-6	7	39.00	8.00
6	-6	-7	46.90	11.40
6	-6	-7	47.40	15.70
-6	-6	7	30.40	14.40
-6	-6	7	54.09	20.00
-6	6	7	36.00	16.60

−6	6	7	48.50	15.40
6	6	−7	51.09	10.80
5	−6	−7	20.10	6.90
5	−6	−7	−6.00	9.40
−5	6	7	31.60	16.30
−5	6	7	3.00	14.50
5	6	−7	9.70	6.80
4	−6	−7	53.59	13.80
4	−6	−7	29.60	11.30
−4	−6	7	48.30	7.30
4	−6	−7	49.70	10.00
−4	−6	7	25.80	12.10
−4	6	7	34.20	17.10
4	6	−7	46.40	8.50
4	6	−7	38.00	14.00
−4	6	7	14.90	12.30
−3	−6	7	140.59	8.90
3	−6	−7	165.28	17.30
−3	−6	7	122.89	18.30
3	−6	−7	140.39	20.10
3	−6	−7	172.38	19.40
3	6	−7	140.09	19.40
−3	6	7	199.78	29.10
−3	6	7	162.68	26.20
−2	−6	7	3.60	2.50
−2	−6	7	8.70	7.10
2	−6	−7	4.70	6.10
2	−6	−7	−1.20	7.90
2	−6	−7	−5.80	5.90
2	6	−7	−6.70	5.90
2	6	−7	0.00	3.30

-2	6	7	-9.40	7.40
-2	6	7	2.20	5.30
-2	6	7	4.60	9.80
-1	-6	7	185.88	17.00
1	-6	-7	237.88	18.00
1	-6	-7	198.48	14.40
1	-6	-7	188.08	18.90
1	6	-7	169.48	15.30
1	6	-7	196.68	14.90
-1	6	7	229.88	25.00
-1	6	7	216.38	29.40
-1	6	7	190.38	14.90
0	-6	-7	195.78	14.40
0	-6	-7	193.88	18.60
0	6	7	161.28	14.00
0	6	-7	169.68	10.30
0	6	-7	195.58	11.50
0	6	7	220.58	29.50
0	6	-7	179.78	9.30
0	6	7	215.58	27.80
0	6	-7	191.38	19.60
-1	-6	-7	370.26	30.50
1	-6	7	361.86	17.50
-1	-6	-7	328.27	22.80
-1	-6	-7	334.47	23.00
-1	-6	-7	346.37	19.40
1	6	7	346.27	19.80
1	6	7	319.47	28.80
-1	6	-7	338.57	21.00
-1	6	-7	335.87	18.60
-2	-6	-7	116.99	13.50

-2	-6	-7	114.49	17.60
-2	-6	-7	112.49	15.20
-2	-6	-7	155.18	22.80
-2	6	-7	130.89	11.50
2	6	7	115.89	13.50
2	6	7	98.79	17.70
-2	6	-7	114.39	6.30
-2	6	-7	126.59	7.00
-2	6	-7	150.78	19.00
-2	6	-7	113.39	19.00
-3	-6	-7	9.60	8.70
-3	-6	-7	12.70	5.10
-3	-6	-7	10.40	12.00
-3	-6	-7	6.20	7.40
-3	6	-7	10.70	3.60
3	6	7	8.70	6.10
3	6	7	17.60	10.20
-3	6	-7	24.00	11.20
-3	6	-7	15.30	3.10
-4	-6	-7	13.80	10.00
-4	-6	-7	27.00	7.80
-4	-6	-7	19.40	12.00
-4	-6	-7	10.00	6.70
-4	6	-7	-7.20	4.20
-4	6	-7	4.60	3.50
-4	6	-7	4.00	10.50
4	6	7	26.80	11.00
-4	6	-7	4.60	3.10
4	6	7	9.10	5.60
-5	-6	-7	7.50	7.40
-5	-6	-7	13.10	14.00

−5	−6	−7	−7.10	9.40
−5	6	−7	2.10	4.30
5	6	7	1.40	5.50
−5	6	−7	13.50	4.30
−5	6	−7	2.40	4.30
−5	6	−7	22.90	12.80
5	6	7	1.90	10.60
−6	−6	−7	0.50	6.60
−6	−6	−7	1.80	10.20
6	6	7	−3.50	6.50
6	6	7	−5.40	9.20
−6	6	−7	0.00	12.90
−6	6	−7	7.50	7.10
−7	−6	−7	53.49	18.60
−7	−6	−7	5.70	8.40
−7	−6	−7	2.40	14.20
−7	6	−7	1.00	11.70
7	6	7	−1.70	7.80
−7	6	−7	1.80	8.70
−7	6	−7	7.80	7.10
−8	−6	−7	13.60	17.30
−8	−6	−7	−2.70	17.10
−8	−6	−7	−10.80	7.70
8	6	7	−14.00	8.60
−8	6	−7	14.40	16.00
−8	6	−7	7.80	9.30
−9	−6	−7	−30.80	17.30
−9	−6	−7	−22.40	16.80
−9	−6	−7	−19.90	8.90
−9	6	−7	14.00	11.70
−9	6	−7	17.90	16.80

9	6	7	-12.20	9.60
-9	-7	7	5.50	22.80
-9	-7	7	-4.20	25.20
9	-7	-7	21.10	15.40
9	-7	-7	1.20	12.20
-9	7	7	1.00	18.80
-9	7	7	26.50	22.00
-8	-7	7	11.70	22.40
-8	-7	7	15.40	12.60
8	-7	-7	23.20	14.30
8	-7	-7	-0.40	10.50
-8	-7	7	-21.50	16.10
-8	7	7	-1.80	22.80
-8	7	7	-8.90	17.90
-8	7	7	15.60	16.20
8	7	-7	30.00	11.10
7	-7	-7	16.30	9.20
7	-7	-7	26.70	12.20
-7	-7	7	5.50	9.20
-7	-7	7	-4.70	16.30
-7	-7	7	19.40	19.40
7	7	-7	11.50	9.00
-7	7	7	12.00	16.40
-7	7	7	-10.50	15.30
-7	7	7	-7.00	13.20
-6	-7	7	-21.50	12.10
6	-7	-7	11.00	7.30
6	-7	-7	11.90	10.80
-6	7	7	6.40	13.20
6	7	-7	12.00	8.00
-6	7	7	10.30	17.50

5	−7	−7	120.49	15.20
5	−7	−7	112.59	18.30
−5	7	7	103.79	23.60
5	7	−7	106.09	14.00
−5	7	7	92.39	24.90
4	−7	−7	10.00	6.70
4	−7	−7	6.60	8.90
−4	−7	7	21.40	12.10
−4	−7	7	30.00	6.10
4	−7	−7	14.80	10.90
−4	7	7	−6.70	11.00
4	7	−7	35.20	7.90
4	7	−7	15.20	12.60
−4	7	7	53.59	17.40
3	−7	−7	9.30	5.80
−3	−7	7	6.00	10.40
3	−7	−7	23.10	9.50
3	−7	−7	−14.10	8.30
−3	−7	7	8.60	4.00
3	7	−7	−0.10	8.80
−3	7	7	12.50	11.10
3	7	−7	3.60	4.80
−3	7	7	6.70	12.70
−2	−7	7	6.70	8.20
2	−7	−7	11.00	8.10
2	−7	−7	−11.80	6.80
2	−7	−7	−2.10	5.20
−2	7	7	−4.50	5.30
2	7	−7	3.20	3.70
−2	7	7	5.40	11.70
2	7	−7	−2.50	6.70

-2	7	7	-2.50	10.50
1	-7	-7	84.09	11.70
-1	-7	7	97.99	13.60
1	-7	-7	91.79	12.80
1	-7	-7	72.49	15.60
1	-7	-7	83.99	12.90
-1	7	7	102.19	13.30
-1	7	7	78.29	21.00
-1	7	7	51.79	15.50
1	7	-7	95.59	11.70
0	-7	-7	41.80	10.50
0	-7	-7	16.70	7.10
0	-7	7	33.00	8.50
0	-7	-7	17.20	8.10
0	7	7	28.20	13.60
0	7	7	26.10	12.20
0	7	-7	31.40	6.30
0	7	-7	38.30	5.60
0	7	-7	38.90	5.30
0	7	-7	27.90	7.80
0	7	7	28.00	7.60
-1	-7	-7	23.10	13.60
-1	-7	-7	21.80	9.00
1	-7	7	11.40	5.30
-1	-7	-7	25.00	6.90
1	7	7	15.00	7.90
-1	7	-7	8.70	6.90
-1	7	-7	13.70	2.90
1	7	7	17.90	12.60
-2	-7	-7	11.40	7.90
-2	-7	-7	6.80	5.50

-2	-7	-7	20.40	7.40
-2	-7	-7	-11.10	9.20
2	7	7	6.90	6.10
2	7	7	35.10	14.50
-2	7	-7	1.20	2.40
-2	7	-7	13.20	9.90
-2	7	-7	0.30	2.40
-2	7	-7	11.00	7.30
-3	-7	-7	3.50	8.90
-3	-7	-7	1.10	10.90
-3	-7	-7	3.70	6.30
-3	-7	-7	-2.50	5.60
-3	7	-7	-5.00	8.80
-3	7	-7	7.00	2.50
3	7	7	3.80	6.50
3	7	7	6.50	10.10
-3	7	-7	-8.30	5.80
-3	7	-7	-2.60	2.60
-4	-7	-7	7.10	11.60
-4	-7	-7	10.00	6.80
-4	-7	-7	6.60	13.00
4	7	7	9.90	9.50
-4	7	-7	13.90	10.30
-4	7	-7	12.30	9.90
4	7	7	17.20	7.50
-4	7	-7	13.30	3.50
-5	-7	-7	7.30	7.20
-5	-7	-7	13.70	11.80
-5	-7	-7	-6.30	12.90
-5	7	-7	23.90	13.00
5	7	7	20.90	8.50

5	7	7	6.60	11.20
−5	7	−7	7.10	12.80
−5	7	−7	20.70	5.60
−6	−7	−7	−7.50	12.00
−6	−7	−7	−5.40	8.00
−6	−7	−7	8.60	13.80
6	7	7	8.00	12.30
−6	7	−7	3.30	14.10
6	7	7	5.20	6.90
−6	7	−7	−3.60	12.70
−7	−7	−7	−8.30	15.80
−7	−7	−7	4.10	16.10
−7	−7	−7	3.40	8.30
7	7	7	6.20	14.70
−7	7	−7	0.50	15.20
−7	7	−7	−2.60	13.40
7	7	7	9.00	8.40
−8	−7	−7	33.30	22.20
−8	−7	−7	4.70	10.10
−8	−7	−7	−0.70	17.20
−8	7	−7	30.00	17.80
−8	7	−7	4.00	18.00
8	7	7	−0.10	10.30
−8	7	−7	−6.30	8.40
−9	−7	−7	−24.10	21.70
−9	−7	−7	8.20	11.60
−9	−7	−7	4.50	21.90
−9	7	−7	−10.40	18.90
−9	7	−7	−19.30	11.60
−9	7	−7	−16.60	22.80
9	−8	−7	2.10	12.70

-9	-8	7	-28.40	24.10
9	-8	-7	-23.90	14.60
-9	-8	7	57.39	27.40
9	8	-7	-2.80	12.20
-9	8	7	18.60	20.90
-9	8	7	-12.60	23.70
-9	8	7	-5.30	22.60
-8	-8	7	-11.70	19.20
-8	-8	7	-2.20	19.70
8	-8	-7	5.30	12.50
8	-8	-7	-23.50	13.20
-8	8	7	2.40	20.90
-8	8	7	-5.00	15.60
-8	8	7	-17.70	18.00
8	8	-7	4.40	10.00
-7	-8	7	6.10	17.80
-7	-8	7	18.60	18.70
7	-8	-7	8.60	13.40
7	-8	-7	-1.00	9.50
7	8	-7	10.10	8.70
-7	8	7	19.90	19.20
-7	8	7	10.30	18.40
-6	-8	7	-2.40	14.00
6	-8	-7	8.90	8.30
6	-8	-7	-10.10	9.60
-6	8	7	14.60	16.10
-6	8	7	25.30	20.20
6	8	-7	-0.60	7.30
5	-8	-7	62.69	12.00
5	-8	-7	63.39	17.60
5	8	-7	61.19	10.90

-5	8	7	42.00	19.10
-5	8	7	85.79	22.60
4	-8	-7	5.80	11.70
-4	-8	7	-7.00	10.30
4	-8	-7	-1.60	8.90
-4	-8	7	-3.20	4.90
4	-8	-7	6.40	6.40
-4	8	7	-34.70	11.40
4	8	-7	-4.30	12.10
-4	8	7	-1.40	12.90
4	8	-7	1.10	6.10
3	-8	-7	-19.10	8.00
3	-8	-7	0.90	5.50
-3	-8	7	3.30	9.70
3	-8	-7	5.90	8.70
3	8	-7	5.30	9.50
-3	8	7	-12.80	11.50
-3	8	7	-2.30	12.00
3	8	-7	2.00	5.10
2	-8	-7	-11.00	5.00
2	-8	-7	-0.50	6.70
-2	-8	7	-2.40	9.10
2	-8	-7	-1.20	8.90
2	-8	-7	11.50	8.00
-2	8	7	5.50	6.10
-2	8	7	7.50	12.00
2	8	-7	6.00	4.70
-2	8	7	9.80	10.80
2	8	-7	-0.90	6.70
1	-8	-7	58.09	10.40
-1	-8	7	5.80	7.10

1	-8	-7	29.20	8.60
1	-8	-7	48.10	9.50
1	-8	-7	39.50	12.10
-1	8	7	31.70	15.30
-1	8	7	42.70	9.90
1	8	-7	31.30	6.00
1	8	-7	33.20	7.70
-1	8	7	28.20	13.70
0	-8	-7	28.90	9.10
0	-8	-7	30.80	9.30
0	-8	-7	54.69	17.80
0	-8	-7	40.20	8.80
0	-8	7	35.00	9.00
0	-8	-7	32.50	8.70
0	8	-7	39.50	5.40
0	8	7	32.50	9.20
0	8	-7	19.20	8.50
0	8	7	32.00	14.70
-1	-8	-7	4.40	5.20
-1	-8	-7	15.10	12.10
-1	-8	-7	2.30	7.80
-1	-8	-7	26.50	9.70
1	8	7	25.00	14.30
-1	8	-7	18.00	3.10
1	8	7	-1.40	5.90
-1	8	-7	-5.60	9.10
-2	-8	-7	17.70	13.70
-2	-8	-7	11.10	9.20
-2	-8	-7	-2.30	6.00
-2	-8	-7	-8.00	6.50
2	8	7	-1.60	6.30

-2	8	-7	-8.60	8.90
-2	8	-7	-0.70	7.90
2	8	7	12.90	12.40
-2	8	-7	0.90	2.10
-3	-8	-7	232.68	20.90
-3	-8	-7	239.28	17.80
-3	-8	-7	233.48	22.10
-3	-8	-7	298.47	34.60
-3	8	-7	204.08	27.50
-3	8	-7	218.18	7.10
-3	8	-7	197.28	8.00
3	8	7	259.87	34.70
3	8	7	220.88	17.90
-3	8	-7	222.98	23.20
-4	-8	-7	58.19	15.20
-4	-8	-7	43.40	12.50
-4	-8	-7	6.10	12.80
-4	8	-7	46.10	4.60
-4	8	-7	39.10	9.20
-4	8	-7	22.60	14.60
-4	8	-7	35.10	4.50
4	8	7	52.59	17.50
-4	8	-7	47.60	16.00
4	8	7	35.60	10.20
-5	-8	-7	-11.50	8.00
-5	-8	-7	12.20	14.50
-5	-8	-7	-6.60	14.20
-5	8	-7	-4.80	11.30
5	8	7	3.30	6.90
5	8	7	1.40	13.30
-5	8	-7	-2.70	7.00

-5	8	-7	-0.40	4.10
-5	8	-7	34.40	18.00
-6	-8	-7	-4.90	16.20
-6	-8	-7	4.90	8.80
-6	-8	-7	4.40	14.30
6	8	7	-4.70	6.80
-6	8	-7	-0.90	12.50
-6	8	-7	13.20	7.40
6	8	7	1.20	14.40
-7	-8	-7	4.70	18.60
-7	-8	-7	38.40	21.20
-7	-8	-7	14.30	11.20
-7	8	-7	12.40	17.60
-7	8	-7	6.90	16.70
7	8	7	13.40	9.40
7	8	7	-1.90	14.70
-8	-8	-7	11.00	21.60
-8	-8	-7	7.30	11.60
-8	-8	-7	-13.80	19.70
8	8	7	0.20	9.20
-8	8	-7	22.30	22.30
-8	8	-7	42.50	17.90
-9	-8	-7	32.00	14.10
-9	-8	-7	8.60	25.50
-9	-8	-7	-26.20	23.30
-9	8	-7	7.70	20.70
9	8	7	-15.60	10.80
-9	8	-7	2.30	21.70
8	-9	-7	21.80	16.40
-8	-9	7	17.90	21.20
8	-9	-7	15.00	11.60

−8	9	7	8.80	20.10
8	9	−7	3.70	9.80
−7	−9	7	−16.20	17.70
7	−9	−7	16.40	14.60
7	−9	−7	5.00	9.40
7	9	−7	−5.40	9.30
−7	9	7	27.10	20.90
−7	9	7	−10.00	19.60
6	−9	−7	3.10	8.40
−6	−9	7	−21.20	16.50
6	−9	−7	1.40	10.30
−6	9	7	24.50	17.90
6	9	−7	1.00	8.20
−6	9	7	−13.80	14.80
5	−9	−7	73.79	18.90
5	−9	−7	93.79	13.30
5	−9	−7	112.39	19.90
−5	9	7	91.19	25.70
5	9	−7	79.29	12.40
−5	9	7	59.39	26.10
5	9	−7	74.49	22.70
4	−9	−7	33.80	13.20
4	−9	−7	90.69	12.90
−4	−9	7	84.19	18.50
−4	−9	7	75.39	7.30
4	9	−7	86.19	12.50
4	9	−7	76.29	17.30
−4	9	7	80.09	24.70
−4	9	7	72.29	20.50
3	−9	−7	−0.90	6.80
3	−9	−7	−4.50	9.90

-3	-9	7	-24.30	9.10
3	-9	-7	25.20	7.90
-3	9	7	31.70	15.40
-3	9	7	5.20	6.70
-3	9	7	-5.50	14.20
3	9	-7	10.40	6.00
3	9	-7	8.00	10.10
-2	-9	7	79.29	15.50
2	-9	-7	98.59	18.60
2	-9	-7	118.19	10.80
2	-9	-7	80.09	11.70
2	-9	-7	127.19	16.00
-2	9	7	116.79	26.50
2	9	-7	99.29	10.90
2	9	-7	107.19	15.00
-2	9	7	129.09	27.90
-2	9	7	132.49	17.00
1	-9	-7	330.57	19.70
1	-9	-7	309.97	15.20
1	-9	-7	294.27	25.60
1	-9	-7	319.17	14.90
-1	-9	7	211.28	28.40
-1	9	7	235.48	32.60
-1	9	7	294.57	39.10
-1	9	7	303.67	21.80
1	9	-7	285.77	16.40
1	9	-7	298.37	14.50
0	-9	-7	7.60	6.20
0	-9	-7	0.30	7.00
0	-9	-7	13.70	6.20
0	-9	-7	2.40	9.10

0	-9	-7	12.20	14.30
0	9	-7	-4.00	6.80
0	9	7	29.70	10.00
0	9	7	15.20	16.00
0	9	-7	11.60	3.90
-1	-9	-7	51.49	14.20
-1	-9	-7	40.90	10.00
-1	-9	-7	78.29	19.60
-1	-9	-7	57.89	11.60
1	9	7	34.20	10.50
-1	9	-7	47.20	14.10
-1	9	-7	26.20	14.00
1	9	7	54.79	20.20
-2	-9	-7	5.40	11.80
-2	-9	-7	10.20	10.00
-2	-9	-7	16.90	8.70
-2	-9	-7	15.40	9.20
2	9	7	4.50	6.60
-2	9	-7	3.40	11.60
-2	9	-7	0.80	1.90
2	9	7	10.00	15.30
-2	9	-7	-15.00	9.60
-3	-9	-7	5.00	8.80
-3	-9	-7	-7.90	12.20
-3	-9	-7	-14.20	9.00
3	9	7	17.10	9.80
-3	9	-7	-14.30	9.10
3	9	7	11.70	11.40
-3	9	-7	1.20	11.30
-4	-9	-7	18.00	12.80
-4	-9	-7	37.20	16.40

−4	−9	−7	21.00	10.80
4	9	7	21.00	15.00
−4	9	−7	20.70	3.60
4	9	7	29.20	10.40
−4	9	−7	34.30	15.40
−4	9	−7	54.79	16.80
−5	−9	−7	−1.20	8.10
−5	−9	−7	15.70	16.50
−5	−9	−7	5.00	13.40
5	9	7	1.90	8.80
−5	9	−7	22.90	14.10
−5	9	−7	7.00	8.80
−5	9	−7	20.20	14.60
5	9	7	−0.50	13.80
−5	9	−7	13.90	4.70
−6	−9	−7	54.79	22.50
−6	−9	−7	44.40	17.90
−6	−9	−7	5.50	12.10
6	9	7	32.10	18.80
−6	9	−7	16.50	18.60
6	9	7	21.00	9.60
−6	9	−7	29.40	16.40
−7	−9	−7	3.00	18.90
−7	−9	−7	37.10	25.10
−7	−9	−7	29.00	14.00
−7	9	−7	6.50	20.50
7	9	7	10.20	17.10
−7	9	−7	45.10	19.90
7	9	7	48.10	12.30
−8	−9	−7	18.30	12.80
−8	−9	−7	48.00	23.60

-8	-9	-7	5.00	18.70
-8	9	-7	-6.10	22.40
-8	9	-7	14.30	18.70
8	9	7	-10.50	10.10
-8	-10	7	-8.50	24.40
8	-10	-7	-15.50	15.30
8	-10	-7	28.90	18.30
-8	10	7	-5.00	20.80
8	10	-7	7.50	12.80
-8	10	7	-11.30	22.50
7	-10	-7	16.50	11.20
7	-10	-7	-9.80	13.40
-7	-10	7	-1.50	21.00
-7	10	7	14.50	17.80
7	10	-7	0.90	10.20
-7	10	7	12.80	26.20
6	-10	-7	2.50	13.20
6	-10	-7	-3.70	8.90
-6	-10	7	-5.40	18.90
-6	10	7	9.00	23.30
6	10	-7	21.80	10.10
-6	10	7	13.90	17.20
5	-10	-7	0.90	12.40
5	-10	-7	-2.30	7.50
-5	10	7	15.50	20.20
5	10	-7	5.20	8.10
-5	10	7	-14.70	15.80
4	-10	-7	41.60	11.40
4	-10	-7	25.80	9.00
4	-10	-7	41.30	13.90
-4	-10	7	24.60	15.20

4	10	-7	20.90	14.70
-4	10	7	8.10	16.00
-4	10	7	32.10	20.40
4	10	-7	28.50	9.00
3	-10	-7	-8.90	11.00
-3	-10	7	12.30	12.60
3	-10	-7	-0.10	6.90
-3	10	7	15.40	16.00
3	10	-7	2.90	5.70
-3	10	7	-7.10	8.10
-3	10	7	-8.90	13.80
3	10	-7	-1.10	11.10
2	-10	-7	8.10	7.60
2	-10	-7	-3.90	8.90
-2	-10	7	-12.90	9.50
-2	10	7	19.00	9.20
2	10	-7	12.60	6.80
-2	10	7	20.10	14.10
-2	10	7	33.50	18.80
2	10	-7	12.90	9.60
1	-10	-7	108.49	14.10
1	-10	-7	96.09	16.60
-1	10	7	124.19	18.10
1	10	-7	94.89	8.40
-1	10	7	112.89	28.60
1	10	-7	137.69	10.10
0	-10	7	56.99	10.80
0	-10	-7	41.00	9.30
0	-10	-7	49.00	16.70
0	-10	-7	49.10	20.00
0	10	-7	39.80	6.00

0	10	-7	39.50	10.20
0	10	7	48.10	12.70
-1	-10	-7	13.20	7.10
-1	-10	-7	-5.60	9.10
-1	-10	-7	17.60	8.10
-1	-10	-7	22.60	15.40
1	10	7	15.50	9.50
-1	10	-7	21.20	12.40
-1	10	-7	18.10	3.80
-1	10	-7	-14.00	10.80
-2	-10	-7	71.29	15.90
-2	-10	-7	63.29	20.80
-2	-10	-7	72.09	19.30
-2	10	-7	94.79	19.90
-2	10	-7	65.89	19.20
2	10	7	60.39	15.50
-2	10	-7	55.79	4.20
2	10	7	63.59	19.50
-3	-10	-7	117.59	19.00
-3	-10	-7	95.79	22.10
-3	-10	-7	93.99	17.80
3	10	7	97.29	24.80
-3	10	-7	82.69	19.90
-3	10	-7	108.39	24.20
3	10	7	106.89	16.30
-4	-10	-7	-11.20	12.30
-4	-10	-7	-15.20	15.70
-4	-10	-7	0.80	9.40
-4	10	-7	-2.20	13.30
-4	10	-7	15.10	15.30
4	10	7	-0.90	14.30

4	10	7	4.70	8.30
-5	-10	-7	16.80	17.50
-5	-10	-7	11.30	10.90
-5	-10	-7	23.20	17.50
-5	10	-7	25.40	19.30
-5	10	-7	15.80	14.90
5	10	7	42.00	21.00
5	10	7	15.30	9.90
-6	-10	-7	-25.00	10.40
-6	-10	-7	-0.50	22.10
-6	-10	-7	8.60	17.50
6	10	7	-0.80	7.90
6	10	7	26.30	17.60
-6	10	-7	-18.20	17.90
-7	-10	-7	-2.00	12.10
-7	-10	-7	14.30	23.30
-7	-10	-7	41.70	22.40
7	10	7	1.50	18.10
-7	10	-7	18.20	24.40
-7	10	-7	21.10	17.80
-7	-11	7	-43.90	24.70
7	-11	-7	23.80	15.30
7	11	-7	-19.40	10.50
-7	11	7	-4.30	22.30
6	-11	-7	16.90	15.60
6	-11	-7	12.20	9.90
-6	-11	7	-20.00	20.20
6	11	-7	10.40	9.60
-6	11	7	-27.90	19.30
-6	11	7	19.10	19.40
5	-11	-7	62.39	20.60

5	-11	-7	52.99	10.90
-5	11	7	55.09	25.60
-5	11	7	29.90	19.70
5	11	-7	56.89	12.30
-4	-11	7	3.70	14.80
4	-11	-7	-13.20	11.30
4	-11	-7	-1.40	7.70
-4	11	7	4.90	17.50
4	11	-7	-7.40	7.10
-4	11	7	1.60	9.10
-4	11	7	18.00	17.10
-3	-11	7	-9.50	13.60
3	-11	-7	0.50	7.50
3	-11	-7	-0.30	10.40
-3	11	7	3.20	16.00
-3	11	7	10.90	17.70
-3	11	7	6.00	8.70
3	11	-7	-9.00	5.90
2	-11	-7	12.40	11.90
2	-11	-7	-2.40	7.30
-2	-11	7	-4.00	13.30
2	11	-7	13.40	6.60
-2	11	7	2.90	9.80
2	11	-7	7.10	9.90
-2	11	7	-19.60	12.50
1	-11	-7	49.90	10.70
-1	-11	7	47.90	14.30
1	-11	-7	75.59	21.10
1	-11	-7	51.79	16.00
1	11	-7	40.30	7.90
-1	11	7	41.50	16.50

1	11	-7	48.30	7.90
0	-11	7	10.60	9.20
0	-11	-7	-11.80	8.30
0	-11	-7	8.30	7.60
0	-11	-7	17.30	16.50
0	11	7	13.40	9.60
0	11	7	2.60	17.70
-1	-11	-7	-16.60	15.10
-1	-11	-7	-1.50	10.40
-1	11	-7	6.20	12.80
1	11	7	-6.60	13.60
-1	11	-7	11.70	4.10
1	11	7	12.10	11.10
-1	11	-7	20.30	13.50
-2	-11	-7	42.20	20.20
-2	-11	-7	24.80	9.10
-2	-11	-7	31.40	12.00
-2	11	-7	55.79	16.90
-2	11	-7	23.00	3.70
-2	11	-7	43.30	16.40
2	11	7	12.30	9.60
2	11	7	45.60	20.90
-3	-11	-7	18.70	18.20
-3	-11	-7	23.60	12.00
-3	-11	-7	-4.50	10.40
3	11	7	3.90	17.50
-3	11	-7	30.10	16.10
3	11	7	6.30	10.00
-3	11	-7	29.00	13.80
-4	-11	-7	3.90	17.90
-4	-11	-7	8.20	9.70

-4	-11	-7	15.30	14.20
4	11	7	3.80	8.40
-4	11	-7	13.40	17.30
-4	11	-7	25.80	15.10
4	11	7	-6.80	18.60
-5	-11	-7	5.30	16.70
-5	-11	-7	13.60	13.20
-5	-11	-7	17.20	20.20
-5	11	-7	7.50	15.20
5	11	7	-4.50	18.10
5	11	7	11.10	10.60
-5	11	-7	-17.30	17.90
-6	-11	-7	-25.00	25.80
-6	-11	-7	5.10	12.40
-6	-11	-7	-0.60	20.10
-6	11	-7	-8.90	17.10
6	11	7	-10.50	20.00
-6	11	-7	27.00	21.80
-7	-11	-7	13.40	24.90
-7	-11	-7	10.10	14.60
-7	-11	-7	-23.60	28.50
7	11	7	-25.50	19.30
-7	11	-7	10.50	26.50
-7	11	-7	57.89	20.50
7	11	7	-5.00	11.60
6	-12	-7	1.20	17.80
6	12	-7	14.00	10.50
-6	12	7	7.00	26.00
-6	12	7	30.40	23.80
5	-12	-7	-9.10	15.70
5	-12	-7	19.50	9.90

-5	12	7	17.70	20.30
5	12	-7	31.80	10.70
-5	12	7	24.60	12.20
-5	12	7	-7.30	22.10
4	-12	-7	-9.20	11.50
4	-12	-7	-3.10	8.50
-4	12	7	-12.20	9.00
-4	12	7	-10.70	16.90
4	12	-7	-11.00	8.00
-4	12	7	16.00	21.80
3	-12	-7	17.90	8.70
3	-12	-7	22.50	13.60
-3	12	7	-0.60	9.10
3	12	-7	20.30	8.00
-3	12	7	27.60	21.00
2	-12	-7	6.30	20.60
2	-12	-7	12.90	12.80
2	-12	-7	4.90	8.40
-2	12	7	9.00	10.30
-2	12	7	-20.70	16.50
2	12	-7	10.60	7.30
1	-12	-7	-18.80	10.60
1	-12	-7	-7.10	18.20
1	12	-7	3.10	7.20
-1	12	7	18.50	18.30
-1	12	7	17.20	10.70
1	12	-7	-12.80	6.50
0	-12	-7	25.90	16.50
0	-12	-7	4.30	12.80
0	12	-7	-12.30	10.50
0	12	-7	-29.60	12.50

0	12	7	9.80	10.10
0	12	-7	7.00	5.40
0	12	7	-26.60	17.50
-1	-12	-7	11.80	12.40
-1	-12	-7	49.50	21.40
-1	12	-7	22.20	5.20
-1	12	-7	-10.40	13.40
1	12	7	15.20	11.00
1	12	7	-6.00	15.80
-2	-12	-7	16.10	11.10
-2	-12	-7	-9.40	17.70
2	12	7	-4.90	18.10
-2	12	-7	-7.90	13.10
2	12	7	18.60	11.60
-2	12	-7	-15.40	16.70
-2	12	-7	5.80	4.20
-3	-12	-7	75.69	18.30
-3	12	-7	95.69	22.20
-3	12	-7	81.99	22.70
3	12	7	74.29	14.30
3	12	7	62.69	23.50
-4	-12	-7	7.60	15.00
-4	-12	-7	-3.30	22.30
-4	-12	-7	22.70	13.50
4	12	7	15.70	11.90
-4	12	-7	11.10	20.30
4	12	7	17.70	19.80
-4	12	-7	-3.00	14.40
-5	-12	-7	8.60	12.30
-5	-12	-7	18.30	20.00
-5	-12	-7	-3.30	24.80

5	12	7	1.10	12.00
5	12	7	8.10	21.80
-5	12	-7	2.00	21.50
-5	12	-7	28.90	16.90
-6	-12	-7	-33.90	24.60
-6	-12	-7	4.30	15.30
-6	-12	-7	35.90	27.40
6	12	7	-39.30	19.70
6	12	7	5.40	11.40
-6	12	-7	1.30	17.50
-6	12	-7	-41.40	24.50
5	-13	-7	33.20	30.50
-5	13	7	-13.00	25.50
5	13	-7	5.00	10.50
-5	13	7	16.10	12.30
4	-13	-7	-15.00	15.90
4	13	-7	-10.20	9.20
-4	13	7	-0.60	10.30
-4	13	7	26.30	25.80
3	-13	-7	2.50	16.70
3	-13	-7	34.50	27.50
-3	13	7	-20.70	16.40
-3	13	7	28.50	13.90
3	13	-7	13.00	8.70
2	-13	-7	-16.40	11.40
2	-13	-7	-9.00	22.80
-2	13	7	-0.20	10.90
-2	13	7	-4.70	20.00
2	13	-7	7.00	7.20
1	-13	-7	60.99	23.70
1	-13	-7	68.29	20.10

-1	13	7	74.29	16.70
1	13	-7	63.99	10.40
-1	13	7	84.59	27.40
0	-13	-7	-8.00	12.30
0	-13	-7	20.00	23.30
0	13	-7	13.40	15.00
0	13	7	14.50	20.30
0	13	-7	20.60	11.40
0	13	7	6.70	10.30
0	13	-7	-3.10	5.50
-1	-13	-7	-9.40	13.10
-1	-13	-7	-24.50	20.30
1	-13	7	13.30	10.90
1	13	7	3.20	11.70
-1	13	-7	-16.80	14.80
1	13	7	-22.50	18.10
-1	13	-7	21.60	14.30
-2	-13	-7	8.30	13.30
-2	-13	-7	-27.80	20.40
-2	13	-7	17.80	14.40
-2	13	-7	9.40	19.30
2	13	7	1.20	10.70
2	13	7	4.10	20.80
-3	-13	-7	-41.10	21.10
-3	-13	-7	-9.10	11.80
-3	13	-7	12.80	15.60
3	13	7	25.60	24.20
-3	13	-7	-13.10	21.40
3	13	7	0.70	10.90
-4	-13	-7	-2.60	17.00
-4	-13	-7	2.70	25.70

4	13	7	-17.20	20.80
-4	13	-7	32.50	25.60
-4	13	-7	10.60	17.30
-5	-13	-7	-22.90	29.90
-5	13	-7	14.50	27.30
3	-14	-7	-1.30	17.80
-3	14	7	5.10	12.50
-3	14	7	-7.30	23.20
2	-14	-7	20.10	28.40
2	-14	-7	4.60	14.80
-2	14	7	11.20	13.70
-2	14	7	53.19	27.10
2	14	-7	9.80	9.30
1	-14	-7	7.40	15.20
1	-14	-7	16.60	29.10
1	14	-7	16.60	18.50
-1	14	7	24.40	14.10
1	14	-7	22.90	8.30
0	-14	-7	13.40	16.40
0	-14	-7	6.90	28.20
0	14	7	13.00	11.10
0	14	7	9.30	24.60
0	14	-7	-27.20	15.60
-1	-14	-7	1.10	15.50
-1	-14	-7	-17.60	25.30
-1	14	-7	36.80	18.30
1	14	7	1.40	11.60
1	14	7	-23.10	24.10
-1	14	-7	-14.10	16.20
-2	-14	-7	-0.40	27.00
-2	-14	-7	-5.20	15.20

-2	14	-7	30.60	21.70
-2	14	-7	-11.60	16.00
2	14	7	10.30	14.50
-3	-14	-7	-43.50	27.30
-3	-14	-7	-18.20	15.90
-3	14	-7	74.29	29.90
3	14	7	29.90	20.20
3	14	7	4.20	13.00
-10	0	8	26.10	23.80
-10	0	8	25.90	19.90
10	0	-8	-3.30	11.30
10	0	-8	-7.90	13.70
-10	0	8	16.00	23.00
-9	0	8	17.00	19.10
-9	0	8	12.90	18.60
9	0	-8	25.50	13.20
-9	0	8	-13.30	17.80
-8	0	8	0.10	16.90
-8	0	8	22.70	14.90
-8	0	8	4.10	14.60
8	0	-8	5.90	8.40
-7	0	8	7.80	13.10
-7	0	8	-5.60	10.70
-7	0	8	-4.50	10.70
6	0	-8	39.80	9.20
-6	0	8	51.19	17.40
-6	0	8	77.89	18.90
-6	0	8	70.29	16.50
5	0	-8	-2.70	5.30
-5	0	8	14.40	10.40
-5	0	8	-12.90	8.20

-4	0	8	68.79	15.30
-4	0	8	40.90	11.70
4	0	-8	47.00	10.90
4	0	-8	59.29	9.80
3	0	-8	4.60	7.80
3	0	-8	3.70	4.20
-3	0	8	4.00	9.10
2	0	-8	-4.90	8.60
2	0	-8	7.00	5.00
2	0	-8	6.40	6.90
-2	0	8	8.20	4.80
-2	0	8	6.70	8.00
1	0	-8	-11.30	8.10
-1	0	8	-4.50	4.20
1	0	-8	3.80	6.70
-1	0	8	2.40	4.20
-1	0	8	1.40	3.10
1	0	-8	3.40	4.00
-1	0	8	-1.00	6.40
0	0	8	196.98	11.70
0	0	8	174.58	11.50
0	0	-8	197.98	15.10
0	0	-8	198.98	13.90
0	0	-8	211.58	24.90
0	0	-8	163.58	14.90
0	0	8	170.78	17.30
0	0	8	215.88	26.90
-1	0	-8	-2.00	5.60
1	0	8	4.80	6.40
1	0	8	-1.10	4.50
-1	0	-8	8.00	7.90

1	0	8	-2.70	4.50
-1	0	-8	-1.60	4.50
1	0	8	-1.70	2.70
-1	0	-8	2.90	8.00
-1	0	-8	4.00	4.10
-2	0	-8	436.86	28.50
-2	0	-8	361.46	24.70
-2	0	-8	454.95	31.90
-2	0	-8	428.16	29.50
2	0	8	430.76	18.50
2	0	8	418.06	26.20
-2	0	-8	386.16	19.30
2	0	8	416.26	25.20
-2	0	-8	453.45	25.50
2	0	8	400.86	16.20
-3	0	-8	-5.80	4.10
3	0	8	-8.70	4.70
-3	0	-8	6.00	7.80
3	0	8	-1.70	2.80
-3	0	-8	4.70	4.10
-3	0	-8	-7.90	4.70
-3	0	-8	11.40	8.80
4	0	8	21.80	5.10
-4	0	-8	23.60	10.80
-4	0	-8	22.50	5.50
-4	0	-8	9.00	6.00
-4	0	-8	26.80	9.70
-4	0	-8	28.10	11.80
4	0	8	10.80	6.00
5	0	8	2.80	5.70
5	0	8	2.40	3.50

−5	0	−8	−18.20	7.60
−5	0	−8	−8.20	5.20
−5	0	−8	−1.30	6.90
−6	0	−8	144.09	25.90
−6	0	−8	200.48	25.90
−6	0	−8	173.58	25.90
6	0	8	156.38	11.40
−6	0	−8	124.99	14.30
−7	0	−8	9.00	12.60
7	0	8	−2.10	5.20
−7	0	−8	−16.50	9.90
−8	0	−8	−4.00	12.90
8	0	8	5.50	6.60
−8	0	−8	0.50	13.90
9	0	8	3.30	8.40
−9	0	−8	−9.10	13.70
−9	0	−8	8.10	16.00
10	0	8	7.70	10.50
−10	0	−8	10.00	31.70
−10	0	−8	−13.50	20.80
−10	−1	8	38.90	21.80
10	−1	−8	13.80	11.70
10	−1	−8	22.30	16.60
−10	−1	8	14.00	21.70
−10	1	8	−1.20	23.00
−10	1	8	29.50	20.40
−9	−1	8	−26.20	15.20
9	−1	−8	7.20	11.70
−9	−1	8	26.60	17.90
−9	−1	8	26.40	16.80
9	−1	−8	−4.80	9.90

9	1	-8	5.50	9.60
-9	1	8	-7.10	18.90
-9	1	8	-3.40	16.70
-8	-1	8	3.20	13.20
8	-1	-8	19.40	13.30
-8	-1	8	-6.30	15.30
-8	-1	8	8.80	15.70
8	-1	-8	14.20	9.90
-8	1	8	7.90	17.70
-8	1	8	6.20	14.70
8	1	-8	24.60	9.30
-8	1	8	-9.10	14.10
-7	-1	8	1.50	11.40
-7	-1	8	25.50	15.90
7	-1	-8	-1.80	9.10
7	1	-8	5.80	7.50
-7	1	8	30.50	13.80
-7	1	8	10.00	11.90
-7	1	8	7.90	10.70
-6	-1	8	22.40	13.60
6	-1	-8	-2.80	5.90
-6	-1	8	3.20	11.30
-6	-1	8	5.90	9.60
6	-1	-8	4.60	8.00
6	1	-8	3.70	6.50
-6	1	8	7.00	11.40
-6	1	8	0.10	12.10
-6	1	8	6.70	10.10
5	-1	-8	82.29	14.90
5	-1	-8	68.19	11.10
-5	-1	8	65.69	14.50

-5	-1	8	67.49	16.30
5	1	-8	65.99	10.70
-5	1	8	68.49	19.80
-5	1	8	57.59	17.30
4	-1	-8	5.30	7.00
-4	-1	8	12.80	8.20
4	-1	-8	2.60	10.80
-4	-1	8	15.90	10.00
4	-1	-8	14.60	7.20
-4	1	8	2.80	8.60
4	1	-8	23.30	7.40
-4	1	8	12.60	9.70
3	-1	-8	55.09	12.00
3	-1	-8	69.49	14.80
-3	-1	8	39.00	11.50
-3	-1	8	61.89	9.60
3	-1	-8	52.09	9.10
3	1	-8	55.59	9.20
-3	1	8	58.09	21.10
3	1	-8	36.70	13.10
-3	1	8	47.10	12.60
3	1	-8	62.89	12.50
-2	-1	8	627.24	22.90
2	-1	-8	539.35	32.30
-2	-1	8	593.24	35.00
2	-1	-8	599.54	37.60
2	-1	-8	647.74	26.60
-2	-1	8	583.24	25.00
2	-1	-8	652.23	31.30
2	1	-8	590.34	40.30
-2	1	8	662.63	38.70

2	1	-8	637.84	25.20
2	1	-8	579.34	28.60
-2	1	8	613.94	31.50
1	-1	-8	40.40	9.50
1	-1	-8	17.10	9.80
-1	-1	8	39.60	6.50
1	-1	-8	33.70	7.90
-1	-1	8	24.50	5.90
-1	-1	8	40.20	7.20
-1	-1	8	39.00	11.90
1	-1	-8	19.20	10.60
1	1	-8	34.60	8.70
1	1	-8	30.80	6.80
-1	1	8	37.00	9.10
1	1	-8	36.30	11.00
-1	1	8	24.70	6.30
-1	1	8	50.89	13.70
0	-1	8	144.59	9.30
0	-1	8	131.89	8.90
0	-1	8	152.88	12.50
0	-1	-8	151.68	18.00
0	-1	-8	172.48	15.50
0	-1	8	172.08	22.40
0	-1	-8	135.49	11.70
0	1	-8	141.69	12.90
0	1	8	167.18	15.50
0	1	-8	166.48	11.80
0	1	8	129.09	10.80
0	1	-8	131.59	18.40
0	1	8	148.49	21.40
0	1	8	138.99	10.60

1	−1	8	182.58	14.90
−1	−1	−8	197.88	20.50
1	−1	8	207.98	11.00
1	−1	8	250.87	20.60
−1	−1	−8	169.78	15.40
−1	−1	−8	194.88	17.30
−1	−1	−8	213.68	14.60
−1	−1	−8	193.08	24.10
1	1	8	267.47	29.00
−1	1	−8	187.38	18.30
1	1	8	195.98	12.10
−1	1	−8	217.38	11.90
1	1	8	194.58	13.20
−1	1	−8	190.48	13.10
1	1	8	214.48	17.30
−1	1	−8	194.98	19.70
−2	−1	−8	620.44	27.30
−2	−1	−8	619.64	35.20
2	−1	8	606.04	29.60
2	−1	8	591.54	30.00
−2	−1	−8	630.74	37.60
2	−1	8	650.83	21.20
−2	−1	−8	599.94	25.10
−2	−1	−8	629.34	34.10
2	1	8	690.33	35.20
−2	1	−8	551.04	32.40
−2	1	−8	553.84	36.60
2	1	8	586.64	23.60
−2	1	−8	641.14	31.40
2	1	8	632.54	32.00
−2	1	−8	614.24	28.80

−2	1	−8	615.84	21.70
−2	1	−8	595.04	24.20
2	1	8	596.34	22.10
−3	−1	−8	92.09	18.40
3	−1	8	67.29	8.10
−3	−1	−8	69.99	11.00
−3	−1	−8	74.79	15.00
3	1	8	73.49	8.50
3	1	8	60.99	12.30
−3	1	−8	75.99	9.60
−3	1	−8	73.29	16.70
−3	1	−8	86.59	10.40
−3	1	−8	44.60	12.40
3	1	8	71.29	10.00
−3	1	−8	50.89	11.30
−4	−1	−8	6.50	5.70
−4	−1	−8	10.90	9.40
4	−1	8	11.70	3.90
−4	−1	−8	9.90	10.00
−4	−1	−8	21.10	11.00
−4	−1	−8	4.90	4.20
4	−1	8	6.80	4.90
−4	1	−8	7.60	5.10
−4	1	−8	14.40	10.30
−4	1	−8	10.10	7.60
−4	1	−8	18.90	8.70
−4	1	−8	11.10	3.90
−5	−1	−8	−2.70	9.90
5	−1	8	3.30	3.60
−5	−1	−8	2.20	4.40
5	−1	8	0.40	5.60

-5	-1	-8	1.40	3.70
-5	-1	-8	0.60	8.00
-5	-1	-8	8.90	12.30
-5	1	-8	-3.70	8.70
-5	1	-8	3.20	5.20
-5	1	-8	-7.10	9.00
-5	1	-8	6.60	8.30
-5	1	-8	0.10	2.00
5	1	8	-0.50	3.80
-6	-1	-8	10.30	12.40
-6	-1	-8	28.80	12.10
6	-1	8	19.60	5.60
-6	-1	-8	37.70	9.00
-6	1	-8	20.20	12.40
6	1	8	13.50	5.90
-6	1	-8	4.00	11.80
-6	1	-8	33.40	7.70
-6	1	-8	21.80	10.80
-7	-1	-8	16.10	14.20
7	-1	8	5.90	5.80
7	-1	8	7.70	8.60
-7	-1	-8	16.30	12.20
-7	1	-8	19.80	13.30
-7	1	-8	9.10	14.70
-7	1	-8	-3.90	10.10
7	1	8	8.90	6.20
8	-1	8	-1.80	6.00
-8	-1	-8	6.20	13.00
-8	-1	-8	4.90	13.40
8	1	8	-14.20	6.50
-8	1	-8	-4.50	10.30

-8	1	-8	-9.80	13.90
-9	-1	-8	1.50	17.70
9	-1	8	1.90	7.40
-9	-1	-8	6.50	16.30
-9	1	-8	-18.90	13.20
9	1	8	16.00	8.50
-9	1	-8	-6.20	15.70
-10	-1	-8	-23.10	18.60
-10	-1	-8	45.80	27.20
10	1	8	10.00	10.40
-10	1	-8	13.20	21.10
-10	1	-8	31.40	18.30
10	-2	-8	-18.70	15.40
-10	-2	8	11.00	20.10
10	-2	-8	-1.00	10.30
-10	-2	8	19.70	27.10
-10	-2	8	-3.30	19.70
-10	2	8	-18.70	20.20
-10	2	8	-20.60	27.00
-10	2	8	22.20	22.40
10	2	-8	-15.20	10.10
-9	-2	8	-4.20	18.00
-9	-2	8	5.30	15.70
9	-2	-8	-0.30	13.30
9	-2	-8	2.00	10.10
-9	-2	8	34.70	20.10
9	2	-8	5.50	8.80
-9	2	8	-12.80	15.80
-9	2	8	5.70	18.90
-9	2	8	18.20	17.80
8	-2	-8	12.50	10.50

8	−2	−8	8.10	8.30
−8	−2	8	38.30	15.10
−8	−2	8	31.90	16.90
−8	−2	8	16.60	17.00
−8	2	8	1.70	15.40
−8	2	8	8.70	17.00
−8	2	8	13.90	16.00
8	2	−8	25.80	11.10
−7	−2	8	25.50	15.80
−7	−2	8	11.90	15.60
7	−2	−8	20.70	10.20
−7	−2	8	9.10	11.20
−7	2	8	15.60	12.20
−7	2	8	9.90	13.30
7	2	−8	2.40	6.00
6	−2	−8	233.88	23.30
−6	−2	8	219.88	32.50
−6	−2	8	237.58	21.80
6	−2	−8	163.78	15.50
−6	−2	8	218.78	29.60
6	2	−8	178.78	15.90
−6	2	8	178.98	27.50
−6	2	8	133.19	24.20
−6	2	8	162.38	26.80
5	−2	−8	45.20	9.90
−5	−2	8	46.00	14.80
−5	−2	8	20.90	11.80
5	−2	−8	13.60	7.80
5	2	−8	30.40	7.40
−5	2	8	8.20	11.60
−5	2	8	35.20	13.70

4	−2	−8	90.49	14.00
4	−2	−8	92.29	13.00
−4	−2	8	114.49	14.00
−4	−2	8	79.69	16.80
4	2	−8	84.99	11.70
−4	2	8	56.29	16.10
−4	2	8	78.99	16.10
3	−2	−8	119.09	13.70
−3	−2	8	99.59	16.30
3	−2	−8	123.09	17.60
−3	−2	8	102.29	9.70
−3	2	8	107.49	17.60
3	2	−8	122.79	11.20
3	2	−8	127.89	20.60
−3	2	8	100.69	19.10
−2	−2	8	378.96	15.90
2	−2	−8	321.57	26.70
2	−2	−8	358.26	23.90
2	−2	−8	342.27	20.00
−2	−2	8	334.57	15.40
−2	2	8	383.06	30.40
2	2	−8	311.77	16.70
2	2	−8	350.96	18.80
−2	2	8	322.77	24.90
2	2	−8	293.87	24.40
1	−2	−8	50.59	12.00
−1	−2	8	47.00	6.50
−1	−2	8	62.59	12.30
−1	−2	8	70.69	8.50
1	−2	−8	64.69	14.80
−1	−2	8	67.39	7.60

1	−2	−8	63.99	14.60
1	−2	−8	63.19	10.60
1	2	−8	50.99	8.30
−1	2	8	46.00	15.10
−1	2	8	38.80	9.70
1	2	−8	72.89	13.40
1	2	−8	61.99	10.00
0	−2	−8	84.49	12.90
0	−2	−8	80.49	17.10
0	−2	8	101.59	9.70
0	−2	8	90.49	6.70
0	−2	−8	86.79	15.90
0	−2	−8	102.39	14.70
0	−2	8	93.69	7.00
0	−2	8	94.59	15.50
0	2	−8	94.19	9.80
0	2	8	108.09	15.80
0	2	−8	93.99	15.10
0	2	8	85.89	10.10
0	2	−8	106.89	11.40
0	2	8	98.29	18.80
1	−2	8	5.20	2.90
−1	−2	−8	6.10	5.90
−1	−2	−8	3.30	8.20
1	−2	8	3.10	6.20
−1	−2	−8	35.50	11.60
1	−2	8	4.70	2.60
−1	2	−8	0.50	7.40
1	2	8	2.50	4.00
−1	2	−8	1.20	5.10
1	2	8	12.90	7.00

−1	2	−8	−0.90	3.20
−1	2	−8	2.40	4.00
1	2	8	13.30	9.40
−2	−2	−8	474.75	32.50
−2	−2	−8	495.25	22.70
2	−2	8	503.45	26.30
2	−2	8	470.75	15.00
−2	−2	−8	510.35	31.40
−2	−2	−8	480.85	24.60
2	−2	8	489.55	14.30
−2	2	−8	463.15	24.90
−2	2	−8	467.45	28.20
2	2	8	465.45	32.80
2	2	8	478.35	23.00
−2	2	−8	427.86	33.40
−2	2	−8	492.95	25.20
−2	2	−8	481.25	20.50
−2	2	−8	458.55	17.80
−3	−2	−8	35.10	11.70
−3	−2	−8	36.80	8.00
−3	−2	−8	53.89	14.20
−3	−2	−8	20.20	10.60
−3	−2	−8	44.10	8.50
3	−2	8	50.59	4.90
3	−2	8	35.60	3.80
−3	2	−8	36.10	9.90
−3	2	−8	33.70	14.00
−3	2	−8	42.50	6.90
−3	2	−8	50.69	6.80
−3	2	−8	50.79	8.40
3	2	8	58.69	8.50

3	2	8	24.60	9.90
−3	2	−8	47.30	11.60
−4	−2	−8	61.79	15.90
4	−2	8	59.09	5.70
4	−2	8	59.89	4.80
−4	−2	−8	48.00	9.00
−4	−2	−8	59.59	9.10
−4	−2	−8	82.29	16.90
−4	2	−8	73.49	18.70
4	2	8	47.60	12.70
−4	2	−8	76.69	14.70
−4	2	−8	56.29	8.40
−4	2	−8	56.89	7.10
4	2	8	56.09	8.60
−4	2	−8	58.19	6.10
−4	2	−8	56.69	11.20
−5	−2	−8	14.80	10.80
5	−2	8	11.10	3.40
−5	−2	−8	15.70	11.90
5	−2	8	6.40	4.20
−5	−2	−8	10.80	7.10
−5	2	−8	5.60	9.90
−5	2	−8	6.70	9.30
5	2	8	13.00	5.70
−5	2	−8	7.80	7.80
−6	−2	−8	447.16	18.10
6	−2	8	430.36	16.50
−6	−2	−8	418.86	37.40
6	−2	8	474.25	15.20
−6	−2	−8	440.06	34.20
−6	2	−8	464.65	29.50

-6	2	-8	475.45	37.40
-6	2	-8	457.55	22.00
-6	2	-8	395.86	29.70
6	2	8	434.86	21.10
-7	-2	-8	7.60	12.80
7	-2	8	-4.90	5.90
-7	-2	-8	6.80	13.50
-7	-2	-8	2.00	3.90
-7	2	-8	-10.40	8.70
-7	2	-8	-2.80	12.60
-7	2	-8	-18.70	12.80
7	2	8	2.40	5.30
8	-2	8	16.10	5.20
-8	-2	-8	-10.20	12.50
-8	-2	-8	-10.10	11.90
8	2	8	-3.80	6.30
-8	2	-8	-13.60	11.00
-8	2	-8	7.20	15.70
-9	-2	-8	10.50	16.80
-9	-2	-8	1.10	17.20
9	-2	8	5.40	7.70
-9	2	-8	5.90	15.40
9	2	8	-1.00	8.40
-9	2	-8	-2.20	15.20
-10	-2	-8	-20.10	21.10
-10	-2	-8	10.50	21.70
10	2	8	5.20	10.70
-10	2	-8	-9.40	19.00
-10	2	-8	-7.00	17.20
-10	-3	8	-3.00	19.30
-10	-3	8	-27.20	19.40

10	-3	-8	21.40	16.80
10	-3	-8	-12.50	11.40
-10	-3	8	-9.40	21.10
-10	3	8	30.70	23.70
-10	3	8	-16.70	23.40
-10	3	8	-11.30	20.80
10	3	-8	13.00	11.10
9	-3	-8	-11.50	8.90
-9	-3	8	33.00	21.60
-9	-3	8	4.10	17.70
-9	-3	8	13.60	18.00
9	-3	-8	4.10	12.80
-9	3	8	-1.70	16.70
-9	3	8	-2.80	17.10
-9	3	8	0.90	20.00
9	3	-8	1.70	10.10
8	-3	-8	-6.40	10.90
-8	-3	8	1.30	15.10
8	-3	-8	21.00	9.60
-8	-3	8	1.30	13.70
-8	-3	8	31.20	19.00
-8	3	8	1.80	16.20
-8	3	8	-2.50	14.00
8	3	-8	10.60	8.50
-7	-3	8	18.80	15.20
-7	-3	8	24.30	12.90
-7	-3	8	42.80	17.00
7	-3	-8	11.10	13.20
-7	3	8	37.40	15.90
-7	3	8	41.50	15.10
-7	3	8	19.10	16.00

7	3	−8	21.30	9.10
−6	−3	8	−2.10	12.20
6	−3	−8	1.40	6.60
−6	−3	8	7.00	12.00
6	−3	−8	−6.80	7.20
−6	3	8	0.50	11.00
6	3	−8	1.50	5.30
−6	3	8	1.40	11.70
−6	3	8	−6.60	11.30
5	−3	−8	−2.20	6.20
−5	−3	8	13.00	10.40
−5	−3	8	11.60	7.20
5	−3	−8	−0.10	7.90
−5	3	8	8.30	10.90
−5	3	8	−7.80	9.10
5	3	−8	−4.10	4.50
4	−3	−8	6.10	5.50
−4	−3	8	9.90	9.90
−4	−3	8	5.20	7.80
4	−3	−8	5.50	6.90
4	3	−8	5.40	12.70
4	3	−8	−1.90	4.70
−4	3	8	−0.40	9.20
−4	3	8	−3.30	9.30
−3	−3	8	51.59	13.10
−3	−3	8	69.09	5.60
−3	−3	8	60.79	7.70
3	−3	−8	60.99	13.60
3	−3	−8	70.79	11.80
−3	3	8	64.29	17.70
3	3	−8	48.80	8.40

−3	3	8	75.79	16.90
2	−3	−8	364.96	25.60
−2	−3	8	389.56	15.90
−2	−3	8	404.76	13.00
−2	−3	8	426.76	28.40
2	−3	−8	434.86	22.80
−2	3	8	461.25	30.70
2	3	−8	366.56	26.50
−2	3	8	372.06	30.90
2	3	−8	414.86	18.80
2	3	−8	364.16	14.50
1	−3	−8	121.69	17.20
−1	−3	8	134.99	17.00
−1	−3	8	127.69	7.80
−1	−3	8	153.18	8.60
1	−3	−8	133.49	12.20
−1	3	8	146.59	19.60
−1	3	8	159.18	12.70
−1	3	8	123.09	20.50
1	3	−8	134.79	17.60
1	3	−8	138.99	11.00
1	3	−8	149.19	10.80
0	−3	8	104.99	6.30
0	−3	8	104.39	5.90
0	−3	8	133.19	26.30
0	−3	−8	104.39	12.70
0	−3	−8	100.29	16.20
0	3	−8	131.89	16.70
0	3	−8	109.39	10.40
0	3	−8	122.69	10.50
0	3	8	118.59	18.40

0	3	8	128.59	11.40
0	3	8	108.29	19.00
−1	−3	−8	11.70	5.50
−1	−3	−8	9.70	5.20
1	−3	8	11.00	1.80
1	−3	8	15.90	1.70
1	−3	8	13.10	6.90
−1	−3	−8	12.20	9.30
−1	3	−8	6.70	4.20
−1	3	−8	7.20	5.30
1	3	8	1.30	8.70
1	3	8	14.20	5.20
1	3	8	12.10	9.20
−1	3	−8	9.40	6.70
−2	−3	−8	376.06	20.00
−2	−3	−8	303.37	30.30
−2	−3	−8	419.46	29.90
−2	−3	−8	378.36	22.10
2	−3	8	399.86	21.80
2	3	8	384.16	18.70
−2	3	−8	359.46	30.00
−2	3	−8	322.87	22.70
−2	3	−8	369.96	17.20
−2	3	−8	382.06	17.50
−2	3	−8	386.06	20.60
−2	3	−8	345.47	21.00
−2	3	−8	389.26	16.40
2	3	8	405.16	26.90
−3	−3	−8	58.29	14.60
−3	−3	−8	54.79	16.00
−3	−3	−8	68.89	10.90

-3	-3	-8	61.49	10.40
3	3	8	77.99	15.50
3	3	8	85.99	12.70
-3	3	-8	77.19	7.80
-3	3	-8	84.99	8.20
-3	3	-8	64.09	16.40
-3	3	-8	72.89	13.60
-3	3	-8	61.09	12.70
3	3	8	78.19	9.60
-3	3	-8	69.89	10.40
-4	-3	-8	24.70	7.20
-4	-3	-8	31.40	12.70
-4	-3	-8	17.00	11.60
-4	-3	-8	24.20	7.00
-4	3	-8	29.30	10.00
-4	3	-8	-0.20	8.60
-4	3	-8	37.80	5.10
4	3	8	23.30	9.60
4	3	8	45.30	9.40
-4	3	-8	24.70	5.00
-4	3	-8	20.30	6.00
-4	3	-8	37.40	8.70
4	3	8	26.70	7.20
-5	-3	-8	100.19	11.30
-5	-3	-8	99.89	21.40
-5	-3	-8	88.09	16.30
-5	-3	-8	81.39	11.90
-5	3	-8	92.29	13.20
-5	3	-8	58.69	17.90
5	3	8	93.79	12.20
-5	3	-8	91.69	15.70

5	3	8	94.19	13.10
−5	3	−8	86.99	10.30
5	3	8	106.09	20.30
−6	−3	−8	8.30	12.70
−6	−3	−8	21.60	6.10
−6	−3	−8	14.80	13.70
−6	3	−8	9.20	5.80
6	3	8	18.00	6.50
−6	3	−8	17.20	13.30
−6	3	−8	24.30	9.40
−6	3	−8	10.30	10.70
6	3	8	19.40	8.60
−7	−3	−8	−2.10	11.40
−7	−3	−8	−1.70	12.10
−7	−3	−8	4.40	5.50
−7	3	−8	4.30	9.30
−7	3	−8	4.80	12.20
7	3	8	2.00	9.80
7	3	8	−5.70	6.10
−7	3	−8	7.30	12.20
−8	−3	−8	52.19	19.50
−8	−3	−8	13.30	16.20
−8	3	−8	3.40	11.70
8	3	8	−3.60	7.60
−8	3	−8	21.30	16.40
−9	−3	−8	5.10	16.80
−9	−3	−8	−14.60	15.90
−9	3	−8	8.90	17.40
9	3	8	1.10	9.40
−9	3	−8	10.50	12.60
−10	−3	−8	19.60	29.40

−10	−3	−8	−7.40	22.00
−10	3	−8	−4.80	17.40
10	3	8	6.90	11.30
−10	3	−8	−10.60	16.40
−10	−4	8	−11.60	22.10
−10	−4	8	−6.00	21.90
10	−4	−8	−5.30	15.40
10	−4	−8	−8.20	11.30
−10	−4	8	15.70	23.10
−10	4	8	3.70	19.60
−10	4	8	5.70	22.80
10	4	−8	−13.40	11.00
−10	4	8	10.80	25.40
9	−4	−8	2.90	15.90
9	−4	−8	18.60	11.30
−9	−4	8	22.00	17.10
−9	−4	8	28.60	19.50
−9	−4	8	−4.20	18.80
−9	4	8	−10.00	18.00
−9	4	8	28.40	21.40
9	4	−8	13.30	11.30
−9	4	8	−3.50	18.80
−8	−4	8	21.50	13.30
−8	−4	8	5.60	15.70
8	−4	−8	−1.50	13.20
−8	−4	8	2.90	15.20
8	−4	−8	−6.60	9.20
−8	4	8	4.80	16.90
−8	4	8	21.30	15.90
−8	4	8	−6.60	16.40
8	4	−8	1.60	7.70

7	−4	−8	−7.60	11.30
−7	−4	8	−1.50	11.70
−7	−4	8	24.80	11.30
−7	−4	8	18.90	19.20
−7	−4	8	10.10	10.00
−7	4	8	3.40	14.30
7	4	−8	−4.00	6.90
−7	4	8	−5.20	13.20
−7	4	8	−15.40	13.50
−6	−4	8	9.80	12.80
6	−4	−8	17.70	11.10
6	−4	−8	4.10	7.90
−6	−4	8	19.60	8.20
−6	−4	8	11.80	15.70
−6	−4	8	8.40	8.80
6	4	−8	7.40	7.20
−6	4	8	−11.10	12.00
−6	4	8	−1.10	11.30
−6	4	8	15.60	12.90
−5	−4	8	97.49	20.30
−5	−4	8	95.79	11.10
−5	−4	8	98.59	9.00
5	−4	−8	118.99	19.90
5	−4	−8	89.89	13.50
5	4	−8	106.09	13.80
−5	4	8	60.09	17.10
−5	4	8	100.49	21.30
−4	−4	8	84.29	18.20
4	−4	−8	91.39	13.20
4	−4	−8	84.39	17.10
−4	4	8	131.79	23.10

4	4	−8	88.99	11.30
4	4	−8	79.89	17.50
−4	4	8	73.99	20.30
−3	−4	8	265.57	24.70
−3	−4	8	182.08	11.10
3	−4	−8	196.08	15.60
3	−4	−8	154.08	20.20
−3	4	8	229.58	29.70
3	4	−8	214.48	25.50
3	4	−8	237.78	14.50
−3	4	8	179.38	26.00
−2	−4	8	4.70	3.10
2	−4	−8	2.70	4.60
−2	−4	8	−2.70	7.50
2	−4	−8	13.30	7.20
2	−4	−8	11.60	9.50
−2	4	8	25.40	12.80
2	4	−8	11.60	8.10
−2	4	8	−6.00	8.80
2	4	−8	5.60	4.00
−1	−4	8	16.90	3.20
1	−4	−8	22.20	6.60
−1	−4	8	27.10	3.60
−1	−4	8	28.60	9.40
1	−4	−8	28.50	9.40
−1	4	8	20.40	9.10
−1	4	8	16.70	10.90
1	4	−8	17.80	8.20
1	4	−8	22.40	5.50
−1	4	8	23.30	6.80
1	4	−8	23.60	4.00

0	−4	−8	285.77	17.90
0	−4	8	272.07	20.90
0	−4	−8	305.27	24.20
0	−4	−8	260.07	20.80
0	4	8	302.67	28.00
0	4	−8	318.87	19.60
0	4	8	275.07	24.40
0	4	8	283.57	17.20
0	4	−8	267.17	14.10
1	−4	8	51.89	10.80
−1	−4	−8	45.30	10.80
−1	−4	−8	61.19	10.40
−1	−4	−8	75.29	15.80
−1	4	−8	58.69	9.00
−1	4	−8	70.19	9.70
1	4	8	41.40	14.20
1	4	8	59.09	9.40
−1	4	−8	61.99	7.60
1	4	8	53.59	12.80
−1	4	−8	72.49	9.90
−2	−4	−8	121.39	18.60
−2	−4	−8	138.69	20.30
−2	−4	−8	118.89	13.50
−2	−4	−8	113.59	17.10
−2	4	−8	117.89	10.00
−2	4	−8	124.79	20.70
−2	4	−8	140.59	8.60
−2	4	−8	131.59	11.40
2	4	8	157.68	12.30
−2	4	−8	144.29	15.10
2	4	8	137.29	20.80

-2	4	-8	136.19	12.10
-2	4	-8	127.39	12.80
-3	-4	-8	111.89	20.20
-3	-4	-8	83.39	12.00
-3	-4	-8	52.39	15.20
-3	-4	-8	86.69	12.20
3	4	8	73.29	15.70
-3	4	-8	93.59	20.40
-3	4	-8	75.89	12.50
-3	4	-8	88.79	10.30
-3	4	-8	89.49	9.70
3	4	8	108.69	12.10
-3	4	-8	84.69	9.20
-4	-4	-8	27.90	7.60
-4	-4	-8	24.20	11.70
-4	-4	-8	12.40	11.90
-4	-4	-8	16.70	5.90
4	4	8	38.00	8.50
-4	4	-8	25.10	5.20
-4	4	-8	21.40	5.00
-4	4	-8	49.20	15.90
4	4	8	28.20	11.50
-4	4	-8	29.10	10.10
-4	4	-8	27.50	5.90
-5	-4	-8	11.10	6.70
-5	-4	-8	-8.80	8.80
-5	-4	-8	8.70	6.70
-5	-4	-8	-9.10	11.20
-5	4	-8	15.60	8.80
-5	4	-8	18.10	7.20
-5	4	-8	-0.50	10.60

5	4	8	22.80	7.00
−5	4	−8	12.00	5.00
5	4	8	8.60	10.70
−6	−4	−8	49.90	20.20
−6	−4	−8	64.59	20.20
−6	−4	−8	55.69	10.10
6	4	8	34.50	8.60
−6	4	−8	35.10	8.90
−6	4	−8	45.10	12.10
−6	4	−8	36.20	15.00
−6	4	−8	73.19	14.40
−7	−4	−8	20.50	14.80
−7	−4	−8	−3.30	6.20
−7	−4	−8	10.50	14.80
−7	4	−8	−3.40	8.20
7	4	8	−10.00	17.80
−7	4	−8	8.00	13.20
7	4	8	0.60	7.40
−8	−4	−8	−4.70	13.30
−8	−4	−8	14.40	7.00
−8	−4	−8	20.10	18.70
−8	4	−8	17.20	15.80
8	4	8	13.40	8.60
−9	−4	−8	10.60	18.30
−9	−4	−8	22.00	19.20
−9	4	−8	21.20	13.80
−9	4	−8	25.30	21.20
9	4	8	7.20	9.80
−10	−4	−8	−28.50	19.60
−10	−4	−8	36.80	23.10
−10	4	−8	2.20	21.00

−10	4	−8	27.80	17.30
10	4	8	6.90	11.70
10	−5	−8	3.80	16.30
−10	−5	8	−26.60	23.00
−10	−5	8	6.70	21.20
−10	5	8	−19.00	20.50
−10	5	8	31.60	42.50
−10	5	8	−4.80	22.50
−9	−5	8	−4.50	20.80
9	−5	−8	30.30	14.80
−9	−5	8	−8.50	19.60
9	−5	−8	10.20	12.50
−9	−5	8	−5.00	16.50
9	5	−8	9.10	10.10
−9	5	8	7.20	20.00
−9	5	8	16.90	18.70
−9	5	8	27.20	21.20
−8	−5	8	32.80	13.50
8	−5	−8	47.50	14.00
8	−5	−8	18.70	10.10
−8	−5	8	36.90	20.50
−8	−5	8	30.90	17.10
−8	5	8	−2.20	15.00
−8	5	8	12.30	16.20
−8	5	8	27.50	20.60
8	5	−8	2.50	9.20
7	−5	−8	44.60	14.30
−7	−5	8	19.50	11.30
−7	−5	8	33.30	11.40
−7	−5	8	79.39	22.30
7	−5	−8	44.30	23.30

−7	−5	8	43.00	18.40
7	5	−8	46.80	11.00
−7	5	8	40.40	17.90
−7	5	8	65.99	20.40
−7	5	8	59.29	22.10
6	−5	−8	15.90	12.20
−6	−5	8	13.60	6.60
6	−5	−8	18.70	9.90
−6	−5	8	−10.30	11.70
−6	5	8	−8.30	13.00
−6	5	8	8.40	14.30
6	5	−8	2.50	7.30
−5	−5	8	141.29	23.00
5	−5	−8	171.98	19.60
5	−5	−8	177.18	23.60
5	5	−8	146.19	13.40
−5	5	8	183.28	30.50
−5	5	8	146.29	27.10
4	−5	−8	39.80	12.00
4	−5	−8	19.10	8.50
−4	−5	8	−1.00	12.70
−4	5	8	31.40	13.90
−4	5	8	36.70	15.70
4	5	−8	11.90	13.30
4	5	−8	25.30	7.10
3	−5	−8	38.80	9.40
3	−5	−8	39.10	11.50
−3	5	8	55.79	15.50
−3	5	8	25.10	13.40
3	5	−8	56.09	12.90
3	5	−8	29.00	6.30

2	−5	−8	56.69	14.00
−2	−5	8	60.39	6.60
2	−5	−8	58.89	12.90
−2	−5	8	61.19	5.80
2	−5	−8	63.59	10.90
−2	−5	8	65.09	13.70
−2	5	8	44.50	13.40
−2	5	8	66.39	10.90
−2	5	8	74.29	19.30
2	5	−8	58.19	12.20
2	5	−8	58.19	8.50
1	−5	−8	233.58	19.70
1	−5	−8	210.18	15.60
−1	−5	8	238.88	20.90
1	−5	−8	152.78	19.30
1	5	−8	219.08	12.10
−1	5	8	214.58	16.20
−1	5	8	210.48	28.30
−1	5	8	206.28	27.10
0	−5	−8	109.39	18.10
0	−5	8	89.89	14.20
0	−5	−8	100.79	13.00
0	−5	−8	104.69	16.00
0	5	8	51.69	14.10
0	5	8	75.49	11.60
0	5	8	66.79	17.10
0	5	−8	77.89	11.20
0	5	−8	80.59	8.10
0	5	−8	87.49	8.00
−1	−5	−8	477.65	24.00
−1	−5	−8	494.15	28.60

−1	−5	−8	478.65	30.20
−1	−5	−8	603.94	39.10
−1	5	−8	504.05	22.40
1	5	8	518.55	34.40
1	5	8	473.15	23.40
−1	5	−8	479.35	20.10
1	5	8	496.45	36.00
−1	5	−8	450.75	17.60
−2	−5	−8	45.80	10.70
−2	−5	−8	88.19	20.80
−2	−5	−8	92.19	17.10
−2	−5	−8	63.89	10.60
−2	5	−8	77.89	10.10
−2	5	−8	65.09	7.50
−2	5	−8	67.79	7.70
−2	5	−8	78.89	12.10
2	5	8	66.99	16.20
2	5	8	62.59	10.20
−2	5	−8	110.89	21.00
−2	5	−8	65.29	8.00
−2	5	−8	63.39	6.80
−3	−5	−8	50.99	10.20
−3	−5	−8	71.69	16.30
−3	−5	−8	63.19	16.30
−3	−5	−8	64.59	11.70
3	5	8	39.50	13.10
−3	5	−8	72.49	19.10
−3	5	−8	54.69	7.40
−3	5	−8	59.29	7.90
−3	5	−8	56.69	6.80
3	5	8	49.40	8.70

−3	5	−8	58.19	8.40
−3	5	−8	64.49	6.70
−4	−5	−8	201.78	29.80
−4	−5	−8	151.78	23.40
−4	−5	−8	181.78	16.20
−4	−5	−8	169.48	15.20
−4	5	−8	136.19	13.10
4	5	8	171.58	14.50
4	5	8	146.89	23.50
−4	5	−8	164.68	10.50
−4	5	−8	157.88	10.10
−4	5	−8	113.19	22.90
−4	5	−8	162.88	10.00
−4	5	−8	148.19	10.50
−5	−5	−8	36.10	14.20
−5	−5	−8	35.30	14.70
−5	−5	−8	47.50	10.00
−5	−5	−8	30.00	8.50
5	5	8	31.50	7.80
−5	5	−8	36.10	6.70
−5	5	−8	32.80	8.70
−5	5	−8	35.30	15.10
−5	5	−8	33.80	8.10
5	5	8	15.80	11.50
−6	−5	−8	3.40	12.60
−6	−5	−8	−3.90	13.70
−6	−5	−8	7.20	7.20
6	5	8	3.80	11.70
−6	5	−8	−7.40	5.90
6	5	8	3.10	7.70
−6	5	−8	15.60	12.90

−6	5	−8	9.40	9.60
−7	−5	−8	20.10	13.50
−7	−5	−8	19.10	13.20
−7	−5	−8	21.40	8.30
−7	5	−8	18.20	14.60
−7	5	−8	12.90	11.40
7	5	8	13.10	8.50
−7	5	−8	17.30	9.10
−8	−5	−8	15.60	8.70
−8	−5	−8	4.90	15.70
−8	−5	−8	27.40	20.70
−8	5	−8	19.90	12.30
8	5	8	13.30	9.60
−8	5	−8	2.20	14.80
−9	−5	−8	−9.50	18.70
−9	5	−8	−3.60	12.00
−9	5	−8	30.50	18.60
9	5	8	−21.70	9.10
9	−6	−8	26.50	13.80
9	−6	−8	−36.50	14.10
−9	−6	8	6.00	22.80
−9	−6	8	33.90	25.70
−9	6	8	1.80	19.20
−9	6	8	20.40	23.50
8	−6	−8	−21.90	12.00
8	−6	−8	1.40	10.10
−8	−6	8	32.30	23.20
−8	−6	8	27.10	20.30
8	6	−8	7.00	9.10
−8	6	8	5.10	16.80
−8	6	8	−0.10	19.60

7	−6	−8	−22.30	10.70
7	−6	−8	5.20	8.80
−7	−6	8	14.30	17.20
−7	−6	8	21.70	18.20
−7	−6	8	26.90	11.40
7	6	−8	−8.90	7.70
−7	6	8	4.60	15.70
−7	6	8	5.30	19.30
−6	−6	8	38.20	17.10
6	−6	−8	24.30	13.10
6	−6	−8	38.40	11.00
−6	6	8	20.60	15.60
6	6	−8	32.60	10.20
5	−6	−8	60.49	17.00
−5	−6	8	42.80	16.40
5	−6	−8	46.20	10.50
−5	6	8	46.40	18.80
−5	6	8	68.19	19.10
5	6	−8	44.50	10.20
4	−6	−8	102.69	17.40
−4	−6	8	94.69	18.80
−4	−6	8	96.59	9.80
4	−6	−8	77.79	12.30
4	6	−8	103.79	17.70
4	6	−8	86.89	11.10
−4	6	8	133.59	27.20
−4	6	8	124.19	23.10
−3	−6	8	210.68	29.40
3	−6	−8	197.48	20.90
3	−6	−8	242.78	17.80
−3	−6	8	198.08	10.70

3	-6	-8	211.58	21.80
3	6	-8	194.78	13.50
3	6	-8	187.28	23.00
-3	6	8	181.28	28.70
-3	6	8	186.48	30.90
2	-6	-8	85.59	15.50
-2	-6	8	87.19	5.40
2	-6	-8	114.09	16.80
2	-6	-8	70.29	11.40
-2	-6	8	76.39	14.30
-2	6	8	91.49	21.20
2	6	-8	71.19	12.70
-2	6	8	57.49	16.30
2	6	-8	78.49	9.70
-2	6	8	93.79	14.00
1	-6	-8	80.29	12.20
1	-6	-8	110.59	18.50
1	-6	-8	91.99	14.40
-1	-6	8	84.29	14.70
1	6	-8	89.79	9.60
-1	6	8	101.99	13.40
-1	6	8	103.99	21.70
1	6	-8	72.89	11.60
-1	6	8	82.89	19.90
0	-6	-8	9.90	6.40
0	-6	-8	26.00	8.40
0	-6	8	-2.70	6.40
0	-6	-8	16.00	9.40
0	6	8	18.20	7.70
0	6	-8	16.90	4.60
0	6	8	6.20	9.00

0	6	8	6.80	11.00
0	6	-8	7.10	5.10
0	6	-8	23.50	7.30
0	6	-8	19.80	4.70
-1	-6	-8	6.90	8.60
1	-6	8	1.90	5.60
-1	-6	-8	3.50	12.30
-1	-6	-8	-0.30	5.00
-1	-6	-8	10.00	7.50
1	6	8	14.50	7.30
-1	6	-8	7.80	3.50
1	6	8	-1.80	8.50
-1	6	-8	12.20	4.10
-1	6	-8	15.10	6.90
-1	6	-8	-0.30	5.80
-1	6	-8	5.20	3.60
-2	-6	-8	69.69	11.70
-2	-6	-8	58.59	12.00
-2	-6	-8	65.39	13.80
-2	-6	-8	73.39	18.30
-2	6	-8	48.70	11.70
-2	6	-8	66.19	17.70
-2	6	-8	69.99	7.30
-2	6	-8	59.79	6.20
2	6	8	53.19	9.90
2	6	8	64.59	16.90
-3	-6	-8	78.19	18.40
-3	-6	-8	80.69	17.30
-3	-6	-8	59.09	11.80
-3	-6	-8	64.69	11.40
3	6	8	76.49	17.90

3	6	8	73.19	11.90
−3	6	−8	74.79	8.20
−3	6	−8	61.69	9.40
−3	6	−8	87.59	18.30
−3	6	−8	72.29	6.20
−4	−6	−8	5.20	6.30
−4	−6	−8	16.90	10.40
−4	−6	−8	12.20	13.40
−4	−6	−8	14.80	7.90
4	6	8	39.90	14.90
4	6	8	4.30	6.50
−4	6	−8	9.80	3.80
−4	6	−8	12.40	5.50
−4	6	−8	3.30	11.10
−4	6	−8	11.60	4.30
−5	−6	−8	32.60	14.20
−5	−6	−8	69.79	11.70
−5	−6	−8	47.70	16.00
5	6	8	40.20	17.60
−5	6	−8	40.20	6.60
5	6	8	21.50	7.60
−5	6	−8	32.80	17.30
−5	6	−8	31.00	5.60
−6	−6	−8	98.09	21.50
−6	−6	−8	101.59	14.80
−6	−6	−8	121.09	25.00
−6	6	−8	67.59	9.90
6	6	8	80.29	21.70
−6	6	−8	73.29	24.10
6	6	8	100.29	13.60
−7	−6	−8	20.60	15.70

-7	-6	-8	-1.90	13.10
-7	-6	-8	14.80	8.60
-7	6	-8	2.80	6.90
-7	6	-8	1.70	17.90
7	6	8	7.60	7.90
-8	-6	-8	13.30	18.80
-8	-6	-8	-5.30	17.20
-8	-6	-8	11.90	9.50
8	6	8	-6.20	8.80
-8	6	-8	-5.00	8.80
-8	6	-8	4.60	16.00
-9	-6	-8	-7.40	10.40
-9	-6	-8	6.20	19.50
-9	-6	-8	58.49	25.00
-9	6	-8	-3.80	11.80
-9	6	-8	-19.10	18.00
9	6	8	-9.20	11.00
9	-7	-8	35.50	18.20
-9	-7	8	-6.00	23.40
9	-7	-8	13.30	13.90
-9	-7	8	15.90	26.70
9	7	-8	5.90	12.60
-9	7	8	-14.50	18.60
-9	7	8	9.20	20.20
8	-7	-8	-4.30	10.50
-8	-7	8	5.50	22.60
8	-7	-8	0.10	14.50
-8	-7	8	-14.30	18.20
-8	7	8	-43.20	18.10
-8	7	8	-8.50	23.40
8	7	-8	6.30	10.70

7	−7	−8	11.80	12.90
−7	−7	8	−24.70	15.80
7	−7	−8	31.60	11.20
−7	7	8	27.60	19.90
−7	7	8	14.20	20.80
7	7	−8	11.90	8.80
−6	−7	8	−12.60	12.90
6	−7	−8	14.80	11.60
6	−7	−8	2.90	8.70
6	7	−8	8.70	7.30
−6	7	8	−15.90	13.20
−6	7	8	−8.30	15.30
5	−7	−8	14.20	8.00
−5	−7	8	23.70	13.80
5	−7	−8	9.60	11.80
5	7	−8	0.10	6.10
−5	7	8	−6.00	12.90
4	−7	−8	20.50	8.20
4	−7	−8	20.10	9.80
−4	−7	8	20.10	5.90
−4	−7	8	26.70	14.50
4	7	−8	23.80	7.30
−4	7	8	32.20	16.50
−4	7	8	38.30	16.10
−3	−7	8	10.50	10.90
3	−7	−8	12.80	10.50
3	−7	−8	15.90	9.60
−3	−7	8	15.30	4.40
3	−7	−8	15.10	7.60
3	7	−8	−2.00	9.30
−3	7	8	2.10	12.40

3	7	-8	17.70	5.70
-3	7	8	-14.30	11.40
-2	-7	8	33.10	10.70
2	-7	-8	31.00	12.30
2	-7	-8	20.20	7.70
2	-7	-8	14.40	14.60
2	7	-8	52.09	11.70
-2	7	8	27.70	16.00
-2	7	8	31.60	13.50
2	7	-8	30.70	6.80
-2	7	8	25.90	9.40
1	-7	-8	111.39	15.10
1	-7	-8	99.19	17.20
1	-7	-8	133.49	18.40
-1	-7	8	115.69	16.90
1	-7	-8	130.29	18.20
1	7	-8	117.69	9.20
1	7	-8	101.29	12.70
-1	7	8	122.69	23.90
-1	7	8	155.18	26.70
-1	7	8	101.59	14.50
0	-7	-8	44.60	9.10
0	-7	-8	53.29	12.80
0	-7	-8	26.70	10.70
0	7	8	18.90	8.10
0	7	8	22.80	13.70
0	7	-8	33.90	6.00
0	7	-8	40.30	6.50
0	7	-8	38.90	5.80
0	7	8	33.30	14.40
-1	-7	-8	36.30	9.50

-1	-7	-8	50.19	16.70
-1	-7	-8	35.60	12.00
-1	-7	-8	53.19	12.50
-1	7	-8	52.69	10.90
1	7	8	33.10	9.70
1	7	8	45.90	16.30
-1	7	-8	43.70	8.60
-1	7	-8	36.60	5.40
-2	-7	-8	10.60	9.50
-2	-7	-8	28.20	9.40
-2	-7	-8	30.90	8.70
-2	-7	-8	15.40	13.50
2	7	8	39.40	13.30
-2	7	-8	11.00	9.10
-2	7	-8	-2.90	8.70
-2	7	-8	12.80	12.00
2	7	8	36.80	9.90
-3	-7	-8	7.80	9.00
-3	-7	-8	11.30	12.00
-3	-7	-8	7.00	10.20
-3	-7	-8	10.20	7.00
-3	7	-8	12.20	9.60
-3	7	-8	12.10	3.50
3	7	8	16.70	7.60
-3	7	-8	14.00	13.20
3	7	8	18.00	11.30
-4	-7	-8	127.59	22.40
-4	-7	-8	68.09	13.10
-4	-7	-8	84.99	14.80
-4	-7	-8	89.69	21.30
-4	7	-8	84.99	7.50

−4	7	−8	92.59	7.40
−4	7	−8	97.99	19.80
4	7	8	114.19	14.40
−4	7	−8	91.39	19.60
4	7	8	84.59	21.00
−4	7	−8	84.39	10.90
−5	−7	−8	49.70	11.90
−5	−7	−8	47.50	17.00
−5	7	−8	31.00	6.40
5	7	8	44.00	10.60
−5	7	−8	39.40	15.80
5	7	8	47.00	16.60
−5	7	−8	38.20	16.40
−5	7	−8	51.09	7.00
−5	7	−8	43.50	7.60
−6	−7	−8	−0.30	14.20
−6	−7	−8	8.50	13.90
−6	−7	−8	3.60	8.60
6	7	8	6.70	15.40
−6	7	−8	−5.20	11.90
6	7	8	−2.40	5.80
−7	−7	−8	−6.10	16.40
−7	−7	−8	−1.90	9.20
−7	−7	−8	−13.60	15.20
−7	7	−8	30.50	17.20
−7	7	−8	−5.80	17.90
7	7	8	4.70	8.40
7	7	8	13.40	17.60
−8	−7	−8	35.30	22.00
−8	−7	−8	17.90	22.80
−8	−7	−8	14.90	11.50

-8	7	-8	1.90	16.00
8	7	8	-1.30	9.40
-8	7	-8	8.30	19.00
-9	-7	-8	-12.70	20.80
-9	-7	-8	-21.20	22.80
-9	-7	-8	19.60	13.10
-9	7	-8	-14.40	22.20
-9	7	-8	19.70	21.90
9	7	8	16.70	10.00
-9	7	-8	-24.70	10.80
-8	-8	8	-39.10	20.90
8	-8	-8	-15.10	14.80
8	-8	-8	19.20	12.10
8	8	-8	-4.30	10.30
-8	8	8	6.90	19.90
-8	8	8	19.60	26.00
-7	-8	8	-37.00	17.00
7	-8	-8	-2.10	9.40
7	-8	-8	-10.80	12.50
-7	8	8	37.70	25.40
7	8	-8	-5.20	8.60
-7	8	8	-4.80	17.40
6	-8	-8	9.30	11.20
-6	-8	8	3.40	16.30
6	-8	-8	11.60	8.80
-6	8	8	2.10	17.80
6	8	-8	11.60	8.40
-6	8	8	5.70	16.40
5	-8	-8	39.40	11.00
-5	-8	8	41.10	18.00
5	-8	-8	55.29	15.30

5	8	-8	29.10	9.20
-5	8	8	54.69	19.80
-5	8	8	58.69	18.60
-4	-8	8	56.19	7.50
4	-8	-8	54.59	11.20
-4	-8	8	34.00	14.20
4	-8	-8	34.10	14.90
-4	8	8	75.49	24.90
4	8	-8	58.39	10.40
4	8	-8	50.39	16.40
-4	8	8	10.90	14.90
3	-8	-8	74.49	16.50
3	-8	-8	63.49	11.70
-3	-8	8	78.59	18.40
-3	8	8	61.89	20.40
-3	8	8	74.99	12.70
-3	8	8	71.09	19.20
3	8	-8	69.49	15.10
3	8	-8	39.80	8.20
2	-8	-8	17.00	10.00
2	-8	-8	31.50	12.20
2	-8	-8	22.90	9.40
-2	8	8	15.90	12.40
-2	8	8	16.10	7.30
2	8	-8	26.30	10.10
2	8	-8	18.70	6.20
-2	8	8	25.80	15.30
1	-8	-8	6.50	8.60
1	-8	-8	6.10	9.30
1	-8	-8	1.50	6.50
-1	-8	8	3.40	8.10

1	-8	-8	-6.60	7.80
-1	8	8	1.00	11.50
-1	8	8	-3.50	10.40
-1	8	8	-1.20	5.80
1	8	-8	2.40	3.90
1	8	-8	4.90	5.80
0	-8	-8	322.37	20.80
0	-8	8	358.86	21.30
0	-8	-8	287.17	24.80
0	8	8	317.07	39.30
0	8	8	349.57	22.70
0	8	8	323.77	39.00
0	8	-8	310.67	15.00
0	8	-8	322.47	14.00
1	-8	8	32.40	6.10
-1	-8	-8	20.50	10.70
-1	-8	-8	41.80	15.10
-1	-8	-8	42.40	10.20
-1	-8	-8	34.70	11.10
1	8	8	41.00	10.70
-1	8	-8	23.40	4.70
1	8	8	47.70	18.10
-1	8	-8	27.60	9.00
-1	8	-8	5.40	15.50
-1	8	-8	30.60	14.90
-2	-8	-8	-11.00	8.40
-2	-8	-8	1.80	7.10
-2	-8	-8	3.80	6.60
-2	-8	-8	0.10	12.20
-2	8	-8	-2.00	11.60
2	8	8	9.50	12.90

2	8	8	9.30	7.60
−2	8	−8	6.80	10.20
−2	8	−8	2.40	2.90
−3	−8	−8	4.70	14.50
−3	−8	−8	38.80	12.20
−3	−8	−8	27.30	9.80
−3	−8	−8	45.00	13.00
3	8	8	37.50	16.10
3	8	8	36.70	10.00
−3	8	−8	48.40	14.40
−3	8	−8	30.60	4.90
−3	8	−8	37.00	4.70
−3	8	−8	35.10	14.20
−4	−8	−8	22.30	16.70
−4	−8	−8	23.90	14.30
−4	−8	−8	22.10	10.80
4	8	8	15.60	11.90
−4	8	−8	30.10	4.60
−4	8	−8	8.00	13.20
−4	8	−8	26.30	10.30
4	8	8	15.30	8.10
−4	8	−8	33.30	13.70
−5	−8	−8	0.40	7.50
−5	−8	−8	−4.60	14.80
−5	−8	−8	11.60	15.50
−5	8	−8	9.30	15.30
5	8	8	−9.70	11.10
−5	8	−8	0.80	7.90
−5	8	−8	−8.10	4.90
−5	8	−8	8.90	13.30
5	8	8	7.80	8.10

−6	−8	−8	−0.20	9.10
−6	−8	−8	2.80	16.20
−6	−8	−8	−6.00	18.00
6	8	8	−5.40	15.20
−6	8	−8	15.40	17.50
6	8	8	7.30	7.60
−6	8	−8	−3.80	12.30
−7	−8	−8	0.60	10.10
−7	−8	−8	−2.20	21.30
−7	−8	−8	−0.80	16.90
−7	8	−8	18.10	17.40
−7	8	−8	27.10	18.70
7	8	8	−27.10	14.90
7	8	8	6.70	8.50
−8	−8	−8	6.60	32.40
−8	−8	−8	10.40	23.00
−8	8	−8	7.80	21.90
−8	8	−8	39.90	18.10
−8	−9	8	−3.00	24.50
8	−9	−8	18.50	13.40
8	−9	−8	10.70	16.90
−8	9	8	34.90	23.40
−8	9	8	49.20	32.70
7	−9	−8	2.50	14.50
7	−9	−8	4.40	13.70
−7	−9	8	0.70	18.00
7	9	−8	16.30	10.30
−7	9	8	13.20	19.00
−7	9	8	25.00	25.30
6	−9	−8	13.40	9.10
−6	−9	8	7.50	18.50

6	−9	−8	1.80	12.10
−6	9	8	23.70	16.90
−6	9	8	2.10	21.70
6	9	−8	17.60	9.90
−5	−9	8	34.20	18.60
5	−9	−8	73.59	18.50
5	−9	−8	38.70	10.30
−5	9	8	60.49	21.70
−5	9	8	41.50	21.80
5	9	−8	20.20	8.80
−4	−9	8	89.29	20.90
−4	−9	8	54.19	6.00
4	−9	−8	61.49	18.00
4	−9	−8	59.59	11.70
−4	9	8	58.29	19.60
−4	9	8	66.29	25.30
4	9	−8	59.49	11.10
4	9	−8	34.80	14.90
3	−9	−8	32.90	10.00
3	−9	−8	11.20	10.80
3	−9	−8	43.00	11.80
−3	−9	8	32.60	13.70
−3	9	8	19.40	15.80
−3	9	8	38.10	11.50
3	9	−8	40.00	12.50
3	9	−8	21.00	7.20
−3	9	8	18.20	17.90
−2	−9	8	−16.20	9.40
2	−9	−8	14.60	8.90
2	−9	−8	−8.00	6.50
2	−9	−8	−7.90	8.20

2	9	-8	6.30	8.60
-2	9	8	-0.90	14.20
2	9	-8	1.60	7.50
-2	9	8	5.40	7.30
1	-9	-8	38.80	9.10
1	-9	-8	18.90	12.20
1	-9	-8	54.59	9.90
1	-9	-8	42.40	11.20
-1	9	8	43.00	17.80
1	9	-8	25.20	7.30
-1	9	8	19.40	17.40
-1	9	8	17.40	9.30
1	9	-8	32.20	6.30
0	-9	-8	22.90	15.70
0	-9	-8	23.60	8.90
0	-9	-8	12.90	10.50
0	-9	-8	35.80	9.70
0	-9	-8	18.10	8.30
0	9	-8	10.20	6.60
0	9	-8	8.80	4.10
0	9	8	12.00	7.80
0	9	-8	14.00	6.80
0	9	8	24.80	14.80
-1	-9	-8	154.08	17.40
-1	-9	-8	164.58	29.40
-1	-9	-8	124.29	19.50
1	9	8	143.29	18.10
-1	9	-8	136.09	8.40
1	9	8	159.38	29.20
-1	9	-8	133.09	24.50
-1	9	-8	141.09	21.50

−2	−9	−8	3.30	8.40
−2	−9	−8	4.80	9.10
−2	−9	−8	19.30	15.00
−2	−9	−8	−12.70	8.50
−2	9	−8	−12.80	11.50
−2	9	−8	−0.80	10.60
2	9	8	3.90	13.30
−2	9	−8	0.90	3.20
−3	−9	−8	31.00	11.60
−3	−9	−8	50.69	14.20
−3	−9	−8	37.90	16.70
−3	9	−8	31.00	4.60
3	9	8	46.60	16.20
−3	9	−8	26.40	4.60
−3	9	−8	38.20	18.00
−3	9	−8	18.30	14.50
3	9	8	36.90	10.50
−4	−9	−8	149.69	24.40
−4	−9	−8	129.69	22.50
−4	9	−8	148.49	7.70
4	9	8	131.09	27.40
−4	9	−8	182.08	28.10
4	9	8	173.08	20.00
−4	9	−8	152.08	7.80
−4	9	−8	184.68	29.40
−5	−9	−8	−3.70	10.60
−5	−9	−8	−29.20	15.40
−5	−9	−8	4.70	13.60
5	9	8	21.60	10.00
−5	9	−8	28.30	16.10
5	9	8	2.70	15.30

-5	9	-8	19.50	13.70
-6	-9	-8	14.00	19.00
-6	-9	-8	16.80	11.20
-6	-9	-8	-7.40	21.40
-6	9	-8	-19.00	14.90
6	9	8	6.90	14.80
6	9	8	4.50	8.10
-6	9	-8	-25.70	17.40
-7	-9	-8	-11.00	11.30
-7	-9	-8	-6.00	19.40
-7	-9	-8	18.50	24.00
-7	9	-8	-11.00	16.20
7	9	8	-17.70	10.60
7	9	8	-7.70	20.10
-7	9	-8	-6.70	20.10
-8	-9	-8	20.60	14.10
-8	-9	-8	24.80	24.30
-8	-9	-8	-18.30	23.20
-8	9	-8	49.30	23.10
8	9	8	11.20	13.60
-8	9	-8	-28.90	24.00
7	-10	-8	5.80	17.90
7	-10	-8	-27.70	14.40
-7	-10	8	14.30	25.30
7	10	-8	7.90	11.20
-7	10	8	13.50	28.00
-6	-10	8	-21.80	18.90
6	-10	-8	-11.30	12.80
6	-10	-8	27.70	10.70
-6	10	8	9.80	22.00
6	10	-8	-12.90	8.80

-6	10	8	17.20	18.80
5	-10	-8	3.70	8.60
5	-10	-8	5.90	13.80
-5	-10	8	-2.40	16.50
-5	10	8	4.50	16.70
-5	10	8	3.30	20.20
5	10	-8	3.60	8.50
-4	-10	8	23.10	16.70
4	-10	-8	24.80	9.90
4	-10	-8	33.60	13.90
-4	10	8	22.00	10.70
-4	10	8	32.80	18.20
-4	10	8	12.50	18.60
4	10	-8	25.60	8.80
3	-10	-8	26.10	13.10
3	-10	-8	18.60	8.90
-3	-10	8	13.90	15.50
3	-10	-8	9.70	8.10
-3	10	8	22.50	17.70
-3	10	8	31.40	10.70
3	10	-8	12.80	11.70
-3	10	8	14.80	20.90
3	10	-8	17.20	7.70
-2	-10	8	-4.80	11.40
2	-10	-8	-5.10	10.30
2	-10	-8	7.30	7.80
-2	10	8	-8.10	12.50
2	10	-8	12.40	9.70
-2	10	8	0.80	9.00
2	10	-8	4.50	5.90
-2	10	8	10.50	16.10

1	-10	-8	-9.30	9.00
-1	-10	8	29.50	21.60
1	-10	-8	5.40	7.70
-1	10	8	30.30	15.40
1	10	-8	18.90	6.40
-1	10	8	16.30	16.30
1	10	-8	15.10	5.30
0	-10	8	79.59	11.90
0	-10	-8	51.69	11.60
0	-10	-8	56.49	14.30
0	-10	-8	89.99	19.70
0	-10	-8	46.50	9.80
0	10	8	59.49	14.30
0	10	-8	63.79	9.60
0	10	8	62.89	23.40
0	10	-8	44.70	9.70
-1	-10	-8	-2.00	9.60
-1	-10	-8	8.40	7.60
-1	-10	-8	10.00	7.60
-1	-10	-8	-2.30	15.90
1	10	8	-5.80	13.70
-1	10	-8	1.30	3.70
-1	10	-8	-7.40	11.50
-1	10	-8	-12.90	10.90
1	10	8	5.00	9.70
-2	-10	-8	11.50	9.10
-2	-10	-8	-4.50	16.00
-2	-10	-8	2.80	8.70
-2	-10	-8	-10.80	9.90
-2	10	-8	-1.60	12.10
2	10	8	0.80	7.20

-2	10	-8	-7.20	3.00
2	10	8	4.50	15.00
-3	-10	-8	-13.50	16.50
-3	-10	-8	-4.40	9.20
-3	-10	-8	3.40	11.50
-3	10	-8	-7.40	13.20
-3	10	-8	-9.60	12.10
3	10	8	9.80	15.00
3	10	8	7.70	8.20
-4	-10	-8	0.10	12.00
-4	-10	-8	11.60	14.30
-4	-10	-8	-45.50	15.20
-4	10	-8	-1.80	15.70
-4	10	-8	10.80	15.70
4	10	8	-31.10	14.40
4	10	8	-4.90	8.90
-5	-10	-8	14.50	16.10
-5	-10	-8	10.40	20.70
-5	-10	-8	-16.30	9.90
5	10	8	-14.30	15.10
-5	10	-8	1.40	14.30
5	10	8	12.80	10.80
-5	10	-8	13.20	18.60
-6	-10	-8	-14.60	18.10
-6	-10	-8	32.60	25.00
-6	-10	-8	-1.50	12.00
6	10	8	17.00	19.40
-6	10	-8	-1.90	19.40
6	10	8	1.90	11.30
-6	10	-8	28.90	17.80
-7	-10	-8	11.30	26.00

−7	−10	−8	12.70	13.90
−7	−10	−8	6.70	23.20
−7	10	−8	−5.10	18.70
−7	10	−8	20.20	23.30
7	10	8	−2.70	22.10
7	10	8	18.20	11.80
6	−11	−8	−6.90	10.50
6	−11	−8	22.50	16.90
−6	−11	8	−1.30	23.70
6	11	−8	4.90	10.30
−6	11	8	−15.20	19.20
−6	11	8	25.50	26.30
5	−11	−8	3.10	14.00
5	−11	−8	5.00	9.10
−5	11	8	1.80	18.90
5	11	−8	14.20	9.60
−5	11	8	5.50	22.80
−5	11	8	3.00	9.90
4	−11	−8	17.20	13.80
4	−11	−8	0.50	8.90
−4	−11	8	−18.40	17.00
−4	11	8	−4.20	19.50
4	11	−8	28.00	9.30
−4	11	8	6.20	19.30
−4	11	8	−2.90	9.70
3	−11	−8	0.50	14.30
3	−11	−8	−3.00	8.80
−3	−11	8	−18.00	14.20
−3	11	8	−3.80	9.50
−3	11	8	13.70	21.00
−3	11	8	7.20	18.30

3	11	-8	2.30	7.00
2	-11	-8	9.00	8.80
2	-11	-8	7.40	11.40
-2	-11	8	-0.70	15.90
-2	11	8	-9.00	14.90
2	11	-8	8.60	9.70
-2	11	8	5.10	8.90
-2	11	8	20.60	19.00
2	11	-8	8.80	6.20
1	-11	-8	17.30	18.00
1	-11	-8	15.20	11.90
1	-11	-8	14.10	8.40
-1	11	8	-4.80	9.90
1	11	-8	3.50	5.50
-1	11	8	-18.10	14.50
0	-11	-8	-4.30	9.60
0	-11	-8	4.10	8.50
0	-11	-8	6.60	17.80
0	11	8	5.90	10.00
0	11	-8	4.70	4.60
0	11	-8	7.60	8.10
0	11	8	-32.60	12.90
-1	-11	-8	-1.50	9.80
-1	-11	-8	-2.90	16.20
-1	11	-8	24.90	13.70
-1	11	-8	-2.50	3.90
1	11	8	-7.30	8.30
-1	11	-8	2.90	13.50
1	11	8	-4.80	16.40
-2	-11	-8	4.70	10.30
-2	-11	-8	-5.50	17.40

-2	-11	-8	1.90	9.60
-2	11	-8	28.60	15.80
-2	11	-8	-5.90	3.40
-2	11	-8	0.00	12.40
2	11	8	7.90	10.60
2	11	8	1.30	15.70
-3	-11	-8	7.30	12.50
-3	-11	-8	-11.30	17.80
-3	-11	-8	8.30	11.30
-3	11	-8	33.40	17.00
3	11	8	5.90	9.70
-3	11	-8	12.60	16.50
3	11	8	6.60	18.80
-4	-11	-8	19.00	21.80
-4	-11	-8	11.90	16.50
-4	-11	-8	18.30	12.40
4	11	8	21.50	11.10
4	11	8	7.60	20.80
-4	11	-8	5.70	16.90
-4	11	-8	-15.50	17.30
-5	-11	-8	17.00	18.00
-5	-11	-8	-29.20	21.00
-5	-11	-8	10.50	14.80
5	11	8	-19.30	17.70
-5	11	-8	40.80	22.00
-5	11	-8	-1.50	16.50
5	11	8	19.80	11.40
-6	-11	-8	0.20	13.50
-6	-11	-8	37.50	21.50
-6	-11	-8	-43.80	25.30
6	11	8	4.00	12.00

-6	11	-8	18.70	22.00
-6	11	-8	18.70	18.10
5	-12	-8	-0.50	16.30
5	-12	-8	0.30	10.70
5	12	-8	-1.00	9.70
-5	12	8	-4.50	25.80
-5	12	8	-13.40	10.90
-5	12	8	-13.90	22.00
4	-12	-8	-17.10	14.70
4	-12	-8	-2.30	9.70
-4	12	8	1.70	20.20
-4	12	8	-12.60	19.00
-4	12	8	12.10	10.90
4	12	-8	2.90	9.10
3	-12	-8	-7.50	8.50
3	-12	-8	-15.60	14.80
-3	12	8	-15.10	16.90
3	12	-8	2.40	8.00
-3	12	8	-17.10	9.90
-3	12	8	1.80	18.70
2	-12	-8	26.30	24.60
2	-12	-8	5.30	8.50
2	-12	-8	8.50	13.00
2	12	-8	21.90	7.90
-2	12	8	38.40	22.70
-2	12	8	13.30	11.60
1	-12	-8	33.10	22.30
1	-12	-8	-6.60	11.80
1	-12	-8	6.10	9.00
-1	12	8	-5.70	11.40
-1	12	8	-24.40	18.40

1	12	-8	3.70	6.20
0	-12	-8	66.19	24.90
0	-12	-8	92.59	22.00
0	12	8	84.29	16.50
0	12	-8	83.79	8.90
0	12	8	74.19	23.90
0	12	-8	58.69	20.20
0	12	-8	67.29	12.10
-1	-12	-8	-18.40	22.30
-1	-12	-8	5.50	12.40
1	12	8	-4.80	16.40
-1	12	-8	-10.10	4.30
1	12	8	3.60	11.80
-1	12	-8	-17.40	14.80
-1	12	-8	9.90	14.20
-2	-12	-8	-18.40	11.50
-2	12	-8	-12.10	14.00
-2	12	-8	-4.70	17.90
2	12	8	21.20	12.80
2	12	8	18.70	18.30
-3	-12	-8	-23.00	22.00
-3	-12	-8	1.40	13.40
-3	12	-8	5.80	16.80
3	12	8	-1.90	21.50
-3	12	-8	3.00	19.40
3	12	8	-4.90	9.60
-4	-12	-8	32.10	25.80
-4	-12	-8	-17.20	13.10
-4	12	-8	11.50	20.90
4	12	8	3.30	10.90
-4	12	-8	22.20	17.70

4	12	8	-6.80	21.30
-5	-12	-8	2.40	15.10
-5	-12	-8	-10.50	24.00
-5	-12	-8	-49.40	31.30
-5	12	-8	7.40	18.00
-5	12	-8	0.00	22.80
5	12	8	8.00	10.80
5	12	8	-12.20	36.70
4	-13	-8	-0.50	11.50
4	-13	-8	40.90	18.00
4	13	-8	21.10	10.20
-4	13	8	-56.89	21.40
-4	13	8	16.10	23.60
-4	13	8	19.30	13.60
3	-13	-8	2.30	10.70
3	13	-8	22.50	8.90
-3	13	8	50.99	27.90
-3	13	8	6.80	12.00
2	-13	-8	-6.70	26.80
2	-13	-8	-20.50	15.20
2	13	-8	-1.70	8.10
-2	13	8	7.50	11.10
-2	13	8	17.00	22.90
1	-13	-8	-7.90	22.40
1	-13	-8	-33.90	11.80
1	13	-8	5.40	6.80
-1	13	8	-21.00	18.70
-1	13	8	12.50	12.10
0	-13	-8	14.20	23.90
0	-13	-8	10.70	16.90
0	13	-8	6.50	6.80

0	13	8	1.70	12.80
0	13	8	0.70	18.90
0	13	-8	13.50	17.70
-1	-13	-8	0.40	14.60
-1	13	-8	14.50	18.50
-1	13	-8	2.60	25.30
1	13	8	-7.70	20.70
1	13	8	17.80	14.10
-2	-13	-8	1.00	23.80
-2	-13	-8	4.20	17.20
2	13	8	25.70	13.80
2	13	8	-9.90	20.10
-2	13	-8	-2.70	16.30
-2	13	-8	-30.60	18.10
-3	-13	-8	-43.90	23.70
-3	13	-8	-3.00	23.20
3	13	8	0.80	38.20
-3	13	-8	-41.20	17.20
-4	-13	-8	23.40	17.00
-4	-13	-8	19.30	29.00
4	13	8	-2.10	12.60
-4	13	-8	-1.10	19.90
-4	13	-8	25.60	25.10
4	13	8	-15.80	22.50
2	-14	-8	37.80	32.70
2	-14	-8	-0.90	17.70
-2	14	8	-13.40	13.00
-2	14	8	-14.00	20.90
2	14	-8	-4.90	8.70
1	-14	-8	8.20	31.60
1	-14	-8	6.90	16.80

1	14	-8	-13.40	19.60
-1	14	8	8.60	13.00
-1	14	8	-31.10	21.60
0	-14	-8	46.40	47.70
0	-14	-8	-16.40	14.20
0	14	8	11.00	13.70
0	14	-8	-24.10	19.50
0	14	8	-29.70	24.40
-1	-14	-8	14.60	29.20
-1	-14	-8	-6.40	16.50
-1	14	-8	-5.20	19.30
1	14	8	21.10	22.20
-2	-14	-8	-6.50	28.20
-2	-14	-8	11.60	15.70
2	14	8	-41.10	23.20
-2	14	-8	5.80	19.40
10	0	-9	18.00	13.20
-10	0	9	-1.20	22.70
10	0	-9	18.30	19.00
-10	0	9	-21.30	21.90
-10	0	9	8.50	22.30
9	0	-9	-8.30	9.70
9	0	-9	-23.30	13.60
-9	0	9	-0.30	20.00
-9	0	9	-20.90	17.80
-9	0	9	23.00	21.00
8	0	-9	1.40	11.90
-8	0	9	2.20	14.50
-8	0	9	3.10	15.80
-8	0	9	30.70	15.40
8	0	-9	-3.90	8.00

−7	0	9	10.70	14.10
7	0	−9	2.40	7.40
−7	0	9	21.90	14.70
−6	0	9	−5.00	10.70
6	0	−9	−14.00	5.80
−6	0	9	8.50	13.20
−5	0	9	72.19	19.20
5	0	−9	83.09	12.70
−5	0	9	77.49	16.70
−4	0	9	−7.20	7.20
4	0	−9	−2.40	5.90
−4	0	9	16.00	9.70
4	0	−9	12.20	10.30
3	0	−9	42.20	12.90
3	0	−9	60.09	10.70
−3	0	9	59.59	12.70
−2	0	9	6.20	8.50
−2	0	9	3.10	6.00
2	0	−9	−4.00	10.00
2	0	−9	7.00	10.00
2	0	−9	2.20	4.50
−1	0	9	442.56	32.90
1	0	−9	483.25	32.40
−1	0	9	414.26	23.50
1	0	−9	399.46	20.80
−1	0	9	414.56	19.80
1	0	−9	417.66	24.70
0	0	−9	2.40	4.70
0	0	9	5.60	4.70
0	0	−9	−1.90	9.50
0	0	−9	−0.10	6.60

0	0	-9	14.70	7.10
0	0	9	3.20	4.30
0	0	9	-6.30	7.50
0	0	9	0.20	3.60
-1	0	-9	118.29	15.10
-1	0	-9	73.79	12.90
1	0	9	77.49	17.10
-1	0	-9	86.19	15.70
-1	0	-9	118.79	13.30
-1	0	-9	89.09	19.00
1	0	9	109.29	11.00
1	0	9	97.39	8.60
1	0	9	95.49	13.60
2	0	9	-6.80	4.50
-2	0	-9	2.60	8.30
-2	0	-9	10.80	8.00
-2	0	-9	-10.20	9.50
-2	0	-9	-1.80	4.70
-2	0	-9	-10.40	4.80
2	0	9	1.20	3.70
2	0	9	1.40	6.30
-3	0	-9	23.10	7.20
-3	0	-9	34.20	7.90
3	0	9	30.80	6.60
3	0	9	31.80	10.80
3	0	9	21.10	5.30
-3	0	-9	27.90	11.40
-3	0	-9	22.70	13.30
-3	0	-9	32.70	12.70
-4	0	-9	3.00	6.90
-4	0	-9	3.20	5.30

4	0	9	-2.60	5.90
-4	0	-9	-0.90	4.80
-4	0	-9	-0.40	11.20
4	0	9	1.50	3.50
-4	0	-9	3.40	11.20
-5	0	-9	75.29	18.50
-5	0	-9	60.69	7.00
-5	0	-9	70.59	15.90
-5	0	-9	59.29	10.30
5	0	9	48.70	9.70
-5	0	-9	46.70	16.50
-6	0	-9	4.00	14.40
-6	0	-9	-1.60	5.80
-6	0	-9	-4.00	10.70
-6	0	-9	-7.20	10.10
6	0	9	1.00	4.40
-7	0	-9	-6.30	11.60
7	0	9	-3.40	5.40
-7	0	-9	-3.30	13.30
-8	0	-9	-14.80	12.60
-8	0	-9	0.30	16.50
8	0	9	-1.70	7.20
-9	0	-9	10.50	16.80
9	0	9	-2.40	8.40
-9	0	-9	4.20	16.40
10	0	9	-20.30	10.90
-10	0	-9	-6.50	19.40
-10	0	-9	-28.70	18.00
-10	-1	9	-22.30	20.10
10	-1	-9	4.50	12.60
10	-1	-9	-1.20	14.70

-10	-1	9	-2.30	20.50
10	1	-9	-12.40	10.00
-10	1	9	9.40	24.30
-10	1	9	52.59	38.60
-10	1	9	-3.00	22.90
10	1	-9	-22.80	15.40
9	-1	-9	2.00	10.90
-9	-1	9	-8.00	16.60
-9	-1	9	-7.50	19.10
9	-1	-9	2.90	12.70
-9	-1	9	17.80	18.60
-9	1	9	14.40	18.60
-9	1	9	9.30	19.70
9	1	-9	13.50	12.30
-9	1	9	11.60	18.80
8	-1	-9	-2.70	8.80
-8	-1	9	6.10	18.20
-8	-1	9	19.60	16.00
8	-1	-9	2.30	11.20
-8	-1	9	34.00	17.50
-8	1	9	4.50	17.50
-8	1	9	12.90	16.40
8	1	-9	10.30	8.70
-8	1	9	6.20	17.90
-7	-1	9	66.79	20.80
7	-1	-9	37.50	15.80
-7	-1	9	70.49	25.90
7	-1	-9	36.80	10.20
-7	-1	9	35.50	16.10
-7	1	9	37.80	20.30
-7	1	9	53.09	19.50

−7	1	9	28.80	16.20
7	1	−9	57.69	12.00
−6	−1	9	9.50	10.40
6	−1	−9	7.20	7.70
−6	−1	9	−0.50	12.10
6	−1	−9	1.70	8.80
−6	1	9	6.30	13.30
6	1	−9	9.20	8.00
−6	1	9	−16.70	11.50
5	−1	−9	23.80	12.00
−5	−1	9	31.60	14.90
−5	−1	9	4.10	10.20
5	−1	−9	26.80	8.30
5	1	−9	24.30	8.90
−5	1	9	13.30	10.60
−5	1	9	8.60	13.00
−4	−1	9	5.30	10.30
4	−1	−9	5.40	7.50
4	−1	−9	2.60	6.60
−4	1	9	10.40	10.60
4	1	−9	−2.20	4.90
−4	1	9	13.90	9.90
3	−1	−9	175.18	21.30
3	−1	−9	177.48	14.80
−3	−1	9	155.18	14.30
3	−1	−9	142.89	22.00
−3	−1	9	198.78	26.00
3	1	−9	179.88	17.60
−3	1	9	195.48	20.30
3	1	−9	157.18	13.90
−3	1	9	183.98	27.00

-2	-1	9	9.20	5.50
-2	-1	9	16.10	10.20
2	-1	-9	23.60	9.00
2	-1	-9	16.70	6.50
2	-1	-9	38.40	12.80
2	1	-9	15.30	7.90
-2	1	9	16.80	9.00
2	1	-9	8.40	10.80
-2	1	9	26.80	11.30
2	1	-9	21.10	6.90
-1	-1	9	169.18	13.70
1	-1	-9	188.48	25.60
1	-1	-9	165.48	14.40
-1	-1	9	158.38	22.80
-1	-1	9	172.78	12.00
-1	-1	9	181.28	11.90
1	-1	-9	216.18	25.00
1	-1	-9	165.88	15.90
1	1	-9	144.09	17.40
1	1	-9	159.28	21.90
-1	1	9	175.38	13.20
1	1	-9	169.98	13.50
-1	1	9	201.98	27.40
-1	1	9	175.48	17.00
0	-1	9	18.00	7.30
0	-1	9	11.20	9.30
0	-1	-9	27.90	10.20
0	-1	-9	18.90	9.90
0	-1	-9	37.50	13.10
0	-1	9	31.70	6.00
0	-1	-9	45.80	8.70

0	1	-9	40.90	10.80
0	1	-9	21.10	7.10
0	1	9	31.20	12.30
0	1	-9	17.10	7.80
0	1	9	20.40	8.10
0	1	9	27.20	7.30
-1	-1	-9	237.18	16.50
1	-1	9	242.28	18.00
1	-1	9	242.18	22.00
1	-1	9	268.87	13.20
1	-1	9	238.38	11.80
-1	-1	-9	222.38	18.20
-1	-1	-9	244.38	25.70
-1	-1	-9	259.57	25.70
1	1	9	264.57	25.20
-1	1	-9	239.68	15.70
1	1	9	237.08	19.60
1	1	9	242.18	13.90
-1	1	-9	248.58	18.00
-1	1	-9	276.77	18.80
-1	1	-9	185.18	22.80
2	-1	9	2.00	6.40
-2	-1	-9	4.50	11.50
2	-1	9	3.00	6.70
-2	-1	-9	1.90	5.20
-2	-1	-9	3.30	9.10
2	-1	9	-6.40	3.50
-2	-1	-9	3.90	7.10
-2	-1	-9	1.40	5.30
2	-1	9	-1.40	2.70
-2	1	-9	-7.50	9.00

2	1	9	2.80	5.80
2	1	9	-1.30	6.30
-2	1	-9	-3.10	4.70
-2	1	-9	3.80	9.30
-2	1	-9	-0.50	4.60
2	1	9	-0.80	4.00
-2	1	-9	-7.10	6.10
-3	-1	-9	119.69	19.90
-3	-1	-9	108.69	13.50
-3	-1	-9	158.68	22.50
3	-1	9	117.59	8.00
-3	-1	-9	108.19	13.30
-3	-1	-9	131.09	22.90
3	-1	9	99.19	9.00
3	1	9	117.79	10.50
3	1	9	138.89	19.30
-3	1	-9	129.89	23.30
-3	1	-9	98.39	12.00
-3	1	-9	110.69	16.10
-3	1	-9	133.59	21.60
-4	-1	-9	33.50	8.60
-4	-1	-9	30.50	14.30
-4	-1	-9	5.60	9.20
-4	-1	-9	23.30	10.50
-4	-1	-9	24.80	7.50
4	-1	9	17.70	5.70
4	-1	9	16.30	4.30
-4	1	-9	13.70	8.40
4	1	9	20.50	10.90
-4	1	-9	12.30	11.40
-4	1	-9	17.60	4.90

−4	1	−9	17.40	5.30
−4	1	−9	16.70	10.20
4	1	9	19.00	5.40
−5	−1	−9	15.00	7.10
5	−1	9	6.80	4.20
−5	−1	−9	10.20	4.80
5	−1	9	−2.30	5.80
−5	−1	−9	7.70	10.50
−5	−1	−9	−0.80	11.90
−5	1	−9	12.10	5.80
−5	1	−9	25.60	12.10
−5	1	−9	9.50	3.90
−5	1	−9	3.00	12.60
−5	1	−9	9.70	9.30
5	1	9	14.50	5.50
−6	−1	−9	12.50	4.20
−6	−1	−9	8.30	10.70
−6	−1	−9	−8.60	13.50
−6	−1	−9	0.90	6.30
6	−1	9	8.40	4.90
−6	1	−9	6.70	13.00
−6	1	−9	20.20	15.40
6	1	9	1.90	5.30
−6	1	−9	19.20	6.50
−6	1	−9	32.10	13.10
−7	−1	−9	−1.60	12.80
7	−1	9	−6.80	9.00
−7	−1	−9	1.80	11.70
7	−1	9	16.90	5.80
7	1	9	0.80	5.60
−7	1	−9	−8.70	15.10

−7	1	−9	14.80	11.80
−7	1	−9	9.90	14.20
8	−1	9	−5.80	5.80
−8	−1	−9	−3.40	13.60
−8	−1	−9	−25.60	14.50
8	1	9	−8.00	7.20
−8	1	−9	20.10	14.20
−8	1	−9	−4.00	12.80
−9	−1	−9	−14.20	16.00
−9	−1	−9	7.80	21.20
9	−1	9	4.20	8.10
−9	1	−9	30.00	16.40
−9	1	−9	−12.10	19.10
9	1	9	17.90	9.70
−10	−1	−9	−29.50	18.40
−10	−1	−9	−11.10	20.30
−10	1	−9	−5.00	18.80
−10	1	−9	−14.40	20.30
10	1	9	−13.10	10.10
−10	−2	9	−33.50	21.10
10	−2	−9	−3.90	12.50
−10	−2	9	−9.40	25.10
10	−2	−9	−2.20	17.60
−10	2	9	56.39	26.20
10	2	−9	7.50	13.10
−10	2	9	−6.20	20.30
−9	−2	9	−18.20	17.20
9	−2	−9	17.90	11.30
−9	−2	9	14.20	20.30
−9	−2	9	−1.40	17.70
9	−2	−9	−7.60	14.70

-9	2	9	33.00	22.30
-9	2	9	23.00	21.60
-9	2	9	17.50	18.50
9	2	-9	2.90	10.60
-8	-2	9	-17.90	15.50
-8	-2	9	20.50	18.20
8	-2	-9	-1.00	10.10
8	-2	-9	1.60	10.80
-8	-2	9	-10.00	15.20
-8	2	9	32.90	18.80
-8	2	9	11.90	14.70
8	2	-9	7.00	8.70
-8	2	9	21.40	16.50
-7	-2	9	42.20	19.30
-7	-2	9	54.49	19.70
-7	-2	9	57.59	16.90
7	-2	-9	53.09	12.30
7	-2	-9	45.20	17.00
-7	2	9	21.50	16.90
7	2	-9	43.40	10.20
-7	2	9	31.30	17.10
6	-2	-9	32.60	12.90
6	-2	-9	29.00	9.90
-6	-2	9	29.70	13.60
-6	-2	9	31.80	11.10
-6	2	9	47.90	19.20
-6	2	9	41.30	15.70
6	2	-9	59.59	11.70
5	-2	-9	144.39	17.80
5	-2	-9	161.68	21.40
-5	-2	9	143.39	24.10

5	2	-9	150.38	14.10
-5	2	9	144.69	24.90
-5	2	9	118.09	26.00
4	-2	-9	15.80	6.60
-4	-2	9	26.70	13.50
4	-2	-9	18.50	10.20
-4	-2	9	21.60	7.10
-4	2	9	15.30	11.40
-4	2	9	40.70	14.90
4	2	-9	19.30	7.00
3	-2	-9	15.70	8.10
-3	-2	9	27.30	13.40
3	-2	-9	13.60	6.70
-3	-2	9	13.90	6.20
-3	2	9	30.60	11.80
3	2	-9	39.10	13.70
-3	2	9	12.60	10.00
3	2	-9	20.10	7.20
3	2	-9	23.30	7.40
-2	-2	9	39.60	7.20
2	-2	-9	29.20	9.90
2	-2	-9	19.90	8.00
-2	-2	9	41.80	12.60
-2	-2	9	23.00	6.10
-2	-2	9	32.50	7.00
2	2	-9	54.59	15.60
-2	2	9	34.80	10.60
-2	2	9	25.20	7.50
-2	2	9	23.90	12.20
2	2	-9	45.10	9.40
2	2	-9	52.29	9.30

-1	-2	9	116.49	10.50
1	-2	-9	119.99	14.50
-1	-2	9	103.99	8.60
1	-2	-9	114.89	16.20
-1	-2	9	113.79	18.60
1	2	-9	118.79	18.60
1	2	-9	129.69	13.20
1	2	-9	111.79	13.90
-1	2	9	130.59	14.20
-1	2	9	106.09	17.10
-1	2	9	74.59	16.40
0	-2	-9	148.49	18.50
0	-2	9	161.48	9.70
0	-2	9	153.28	20.90
0	-2	-9	150.88	13.90
0	-2	9	159.08	12.90
0	-2	9	146.39	9.10
0	2	9	156.48	12.70
0	2	-9	137.39	12.00
0	2	-9	169.08	14.10
0	2	9	193.18	27.80
0	2	9	151.68	19.20
0	2	-9	126.29	16.60
-1	-2	-9	27.90	9.40
1	-2	9	36.60	11.30
1	-2	9	46.50	5.30
-1	-2	-9	61.59	16.40
-1	-2	-9	35.50	7.60
1	-2	9	43.20	5.60
-1	-2	-9	54.29	15.10
1	2	9	39.30	12.10

-1	2	-9	38.30	11.40
-1	2	-9	34.70	9.20
-1	2	-9	28.70	7.00
1	2	9	29.50	7.10
1	2	9	68.59	16.40
-2	-2	-9	15.80	7.10
2	-2	9	20.50	4.10
-2	-2	-9	25.50	8.50
2	-2	9	22.00	3.30
-2	-2	-9	19.40	10.40
2	-2	9	29.10	10.00
-2	-2	-9	32.70	12.00
-2	-2	-9	43.20	14.40
-2	2	-9	18.00	8.10
2	2	9	25.30	10.10
-2	2	-9	41.30	12.90
-2	2	-9	27.40	13.50
-2	2	-9	22.20	6.80
-2	2	-9	8.20	7.60
-2	2	-9	30.60	7.10
2	2	9	19.40	6.30
3	-2	9	21.20	3.40
3	-2	9	32.10	4.50
-3	-2	-9	25.90	8.00
-3	-2	-9	14.50	10.70
-3	-2	-9	19.60	11.70
-3	-2	-9	23.20	7.30
-3	2	-9	28.70	5.90
-3	2	-9	11.80	7.50
3	2	9	33.10	7.00
-3	2	-9	22.70	14.30

-3	2	-9	38.40	9.90
3	2	9	38.10	11.60
-3	2	-9	30.70	11.00
-4	-2	-9	19.70	12.10
-4	-2	-9	2.30	7.70
-4	-2	-9	10.10	6.50
4	-2	9	3.60	2.70
-4	-2	-9	3.30	4.90
4	-2	9	3.20	3.40
-4	2	-9	0.30	10.20
-4	2	-9	0.90	10.10
-4	2	-9	4.60	7.40
4	2	9	6.50	4.80
-4	2	-9	5.70	4.50
-4	2	-9	3.10	5.10
4	2	9	12.80	9.30
-4	2	-9	-6.00	4.40
-5	-2	-9	170.78	12.90
-5	-2	-9	196.98	28.10
-5	-2	-9	164.78	28.40
5	-2	9	173.88	9.10
-5	-2	-9	157.78	17.30
5	-2	9	170.38	8.10
5	2	9	165.38	12.80
-5	2	-9	219.08	24.00
-5	2	-9	156.38	28.40
-5	2	-9	174.18	23.50
-5	2	-9	160.68	14.10
-6	-2	-9	-0.10	7.00
-6	-2	-9	-2.10	11.70
6	-2	9	0.90	4.50

−6	−2	−9	0.90	5.30
6	−2	9	7.60	3.90
6	2	9	−1.00	5.40
−6	2	−9	6.60	5.90
−6	2	−9	−7.10	8.60
−6	2	−9	−9.30	11.90
7	−2	9	1.10	5.60
−7	−2	−9	1.40	13.60
−7	−2	−9	9.20	4.60
−7	−2	−9	17.80	16.10
7	−2	9	0.20	3.90
−7	2	−9	−3.30	13.80
−7	2	−9	10.10	14.60
−7	2	−9	1.20	11.60
7	2	9	5.50	6.70
−8	−2	−9	−0.50	16.80
8	−2	9	0.40	5.50
−8	−2	−9	9.50	16.20
−8	2	−9	−21.50	10.40
−8	2	−9	14.10	18.80
8	2	9	−10.50	7.10
−9	−2	−9	21.40	19.80
−9	−2	−9	68.19	24.60
9	2	9	17.20	10.10
−9	2	−9	15.30	17.80
−9	2	−9	9.50	15.20
10	−3	−9	−2.60	19.60
−10	−3	9	−38.60	22.20
10	−3	−9	−2.80	17.40
−10	−3	9	9.40	22.80
−10	3	9	75.69	30.20

−10	3	9	34.70	25.90
−10	3	9	17.00	23.90
10	3	−9	26.90	14.80
−9	−3	9	45.80	22.20
9	−3	−9	2.90	15.70
−9	−3	9	18.60	21.80
−9	−3	9	−18.30	18.90
9	−3	−9	−8.20	11.70
−9	3	9	−17.10	18.10
9	3	−9	−1.90	10.40
−9	3	9	17.40	19.70
−9	3	9	16.90	17.90
−8	−3	9	7.10	13.60
−8	−3	9	8.50	18.80
8	−3	−9	−2.00	8.60
8	−3	−9	−8.30	11.70
−8	−3	9	13.80	18.60
−8	3	9	−1.10	15.30
−8	3	9	−9.50	16.10
8	3	−9	0.70	7.70
−8	3	9	−16.80	15.80
−7	−3	9	32.60	18.90
−7	−3	9	53.49	20.90
7	−3	−9	30.00	13.00
−7	−3	9	37.20	14.40
7	−3	−9	36.20	10.90
−7	3	9	19.40	14.70
7	3	−9	16.70	9.80
−7	3	9	28.60	17.60
−7	3	9	60.59	19.90
6	−3	−9	38.80	13.50

6	−3	−9	55.69	11.60
−6	−3	9	25.10	16.30
−6	3	9	29.20	15.30
−6	3	9	48.80	16.50
6	3	−9	43.40	10.00
−5	−3	9	−0.30	6.90
5	−3	−9	13.10	9.70
−5	−3	9	−5.30	10.80
5	−3	−9	32.00	10.50
5	3	−9	19.30	7.70
−5	3	9	−13.00	10.70
−5	3	9	14.80	13.10
4	−3	−9	220.98	18.20
4	−3	−9	244.08	23.40
−4	−3	9	219.38	17.60
−4	3	9	225.28	30.90
4	3	−9	244.28	16.60
−4	3	9	257.17	27.30
−3	−3	9	187.28	25.70
3	−3	−9	175.38	16.30
3	−3	−9	197.98	24.40
−3	−3	9	171.18	8.90
−3	3	9	205.28	27.40
3	3	−9	143.19	21.70
3	3	−9	166.58	13.80
−3	3	9	198.78	30.00
−2	−3	9	108.99	9.50
−2	−3	9	107.49	8.80
−2	−3	9	109.39	7.50
2	−3	−9	85.79	15.80
2	−3	−9	86.09	12.80

2	3	−9	98.39	16.40
−2	3	9	96.89	13.20
2	3	−9	115.59	10.00
−2	3	9	105.79	21.30
2	3	−9	96.59	12.90
−2	3	9	88.89	18.20
1	−3	−9	90.89	15.00
−1	−3	9	92.69	16.70
−1	−3	9	74.19	7.30
−1	−3	9	64.69	7.10
1	−3	−9	54.19	10.20
1	3	−9	82.69	11.30
1	3	−9	85.69	15.70
−1	3	9	63.49	17.40
1	3	−9	88.99	10.60
−1	3	9	72.29	16.10
−1	3	9	75.19	11.80
0	−3	−9	28.20	7.80
0	−3	9	30.40	4.40
0	−3	9	32.30	4.50
0	−3	−9	35.40	10.40
0	−3	9	40.20	12.60
0	3	−9	17.90	5.90
0	3	−9	19.30	6.70
0	3	9	33.40	8.10
0	3	9	28.10	12.30
0	3	9	25.80	12.90
0	3	−9	24.00	9.60
1	−3	9	136.79	18.90
−1	−3	−9	121.99	16.40
−1	−3	−9	94.09	13.20

-1	-3	-9	121.09	20.80
1	-3	9	111.19	5.50
1	3	9	139.69	20.50
-1	3	-9	125.09	10.90
-1	3	-9	110.59	13.30
1	3	9	110.69	12.30
-1	3	-9	128.49	17.80
1	3	9	105.79	20.90
-2	-3	-9	256.57	17.60
2	-3	9	292.87	20.30
-2	-3	-9	229.18	22.30
-2	-3	-9	261.37	26.70
-2	-3	-9	230.98	25.80
-2	3	-9	248.88	18.60
-2	3	-9	246.28	31.90
-2	3	-9	251.07	13.30
2	3	9	281.87	16.90
-2	3	-9	283.87	19.50
-2	3	-9	236.78	15.20
2	3	9	272.67	23.10
-2	3	-9	261.27	15.60
-3	-3	-9	96.99	13.30
-3	-3	-9	108.59	22.30
-3	-3	-9	71.49	18.40
-3	-3	-9	108.49	13.70
-3	3	-9	94.29	15.60
3	3	9	112.19	19.30
-3	3	-9	156.78	15.10
-3	3	-9	114.99	12.20
-3	3	-9	78.19	18.60
-3	3	-9	104.39	9.90

3	3	9	121.89	11.10
−4	−3	−9	122.29	14.20
−4	−3	−9	131.69	22.80
−4	−3	−9	116.49	15.00
−4	−3	−9	140.89	24.50
−4	3	−9	130.69	16.30
−4	3	−9	105.09	11.10
−4	3	−9	120.09	10.90
4	3	9	139.69	21.90
−4	3	−9	116.29	23.10
−4	3	−9	124.29	9.60
4	3	9	147.09	12.90
−5	−3	−9	16.20	14.00
−5	−3	−9	10.00	7.10
−5	−3	−9	−2.30	5.60
−5	−3	−9	11.80	12.90
−5	3	−9	22.70	11.40
−5	3	−9	0.00	7.30
5	3	9	4.00	9.20
−5	3	−9	3.00	11.80
5	3	9	15.40	6.00
5	3	9	4.10	9.60
−5	3	−9	13.60	6.70
−6	−3	−9	1.20	6.80
−6	−3	−9	10.80	14.00
−6	−3	−9	−4.20	5.40
−6	−3	−9	2.80	13.90
−6	3	−9	5.20	7.40
−6	3	−9	9.10	11.90
−6	3	−9	11.10	15.30
6	3	9	6.10	6.30

−6	3	−9	1.60	6.30
−7	−3	−9	−4.30	14.30
−7	−3	−9	−3.10	6.00
−7	−3	−9	5.10	13.70
−7	3	−9	11.60	13.30
−7	3	−9	−8.50	13.20
−7	3	−9	0.10	10.10
7	3	9	17.60	8.90
−8	−3	−9	15.60	6.30
−8	−3	−9	16.50	19.70
−8	−3	−9	21.70	16.20
−8	3	−9	23.70	18.70
−8	3	−9	11.50	11.90
8	3	9	20.00	8.10
−9	−3	−9	4.10	17.40
−9	−3	−9	−11.60	18.50
−9	3	−9	−18.00	17.20
9	3	9	−3.90	9.00
−9	3	−9	4.60	12.60
9	−4	−9	−0.40	14.00
−9	−4	9	−2.50	20.60
−9	−4	9	3.70	21.40
9	−4	−9	4.90	12.50
−9	−4	9	−40.60	16.90
9	4	−9	0.40	10.10
−9	4	9	28.70	21.50
−9	4	9	−9.80	18.00
−9	4	9	16.30	21.20
−8	−4	9	37.90	15.20
−8	−4	9	14.20	19.80
8	−4	−9	9.70	9.60

-8	-4	9	8.50	21.90
8	-4	-9	4.60	13.70
-8	4	9	-14.90	15.00
-8	4	9	29.30	22.20
-8	4	9	27.00	20.20
8	4	-9	10.00	8.90
-7	-4	9	12.40	17.70
7	-4	-9	11.90	8.70
7	-4	-9	12.00	13.60
7	4	-9	1.10	6.70
-7	4	9	9.30	15.20
-7	4	9	-20.70	15.00
-6	-4	9	108.69	11.90
-6	-4	9	89.09	11.90
-6	-4	9	151.08	27.50
6	-4	-9	92.79	17.60
6	-4	-9	80.69	19.10
6	4	-9	73.49	15.50
-6	4	9	112.49	25.80
-6	4	9	135.99	29.00
5	-4	-9	86.59	18.60
-5	-4	9	66.39	19.10
5	-4	-9	82.49	13.70
-5	4	9	77.29	19.50
5	4	-9	81.29	13.10
-5	4	9	55.19	19.70
4	-4	-9	35.70	10.60
4	-4	-9	25.60	11.10
-4	-4	9	28.10	12.30
-4	4	9	12.20	11.80
4	4	-9	25.20	8.00

-4	4	9	35.90	14.00
3	-4	-9	63.59	11.40
-3	-4	9	55.59	14.30
-3	-4	9	52.19	7.50
3	-4	-9	41.00	12.00
-3	4	9	73.19	19.90
-3	4	9	29.10	13.90
3	4	-9	67.39	10.10
-2	-4	9	117.99	18.40
2	-4	-9	126.09	15.00
-2	-4	9	141.69	9.10
2	-4	-9	116.49	18.20
-2	-4	9	135.09	9.80
-2	4	9	164.08	25.10
-2	4	9	127.09	23.60
2	4	-9	118.89	17.70
-2	4	9	139.99	16.30
-1	-4	9	331.17	12.10
1	-4	-9	321.37	24.80
1	-4	-9	359.76	21.20
-1	-4	9	335.27	12.00
-1	-4	9	331.57	25.90
-1	4	9	336.37	29.00
1	4	-9	281.47	23.60
-1	4	9	316.37	19.60
1	4	-9	287.27	16.20
-1	4	9	429.36	36.20
0	-4	-9	458.75	32.40
0	-4	-9	393.16	22.60
0	-4	-9	407.66	27.60
0	4	-9	437.66	26.60

0	4	9	459.15	23.30
0	4	-9	422.16	18.70
0	4	9	461.85	36.20
0	4	-9	392.26	18.90
0	4	9	413.96	30.90
1	-4	9	52.09	11.60
-1	-4	-9	73.19	11.80
-1	-4	-9	62.89	15.60
-1	-4	-9	61.49	12.70
-1	4	-9	59.49	11.40
1	4	9	100.59	21.80
1	4	9	47.90	9.20
1	4	9	89.89	18.20
-1	4	-9	62.19	10.40
-1	4	-9	89.59	10.50
-2	-4	-9	42.40	15.30
-2	-4	-9	40.60	10.50
-2	-4	-9	49.30	10.00
2	-4	9	35.50	8.10
-2	-4	-9	35.60	13.70
-2	4	-9	55.59	9.20
-2	4	-9	46.80	9.90
-2	4	-9	29.20	9.50
-2	4	-9	45.80	9.00
-2	4	-9	33.90	9.90
2	4	9	43.80	8.40
2	4	9	39.80	11.30
-2	4	-9	21.60	13.20
-2	4	-9	34.60	5.90
-3	-4	-9	52.79	9.90
-3	-4	-9	51.69	10.60

−3	−4	−9	33.90	15.00
−3	−4	−9	20.20	12.70
3	4	9	26.90	7.60
3	4	9	31.20	12.50
−3	4	−9	42.40	7.90
−3	4	−9	49.70	16.60
−3	4	−9	48.80	7.90
−3	4	−9	53.19	7.50
−3	4	−9	65.29	10.70
−3	4	−9	14.80	8.40
−4	−4	−9	40.90	10.00
−4	−4	−9	18.50	11.90
−4	−4	−9	26.20	15.20
−4	−4	−9	29.20	9.70
4	4	9	26.30	12.40
−4	4	−9	41.00	8.70
−4	4	−9	34.00	16.70
−4	4	−9	37.80	7.60
−4	4	−9	17.10	5.20
−4	4	−9	41.00	7.60
−4	4	−9	15.40	9.90
4	4	9	32.50	7.90
−5	−4	−9	12.10	12.30
−5	−4	−9	9.30	12.30
−5	−4	−9	23.70	8.70
−5	4	−9	25.50	7.20
−5	4	−9	24.40	8.60
−5	4	−9	21.00	15.50
5	4	9	26.30	8.30
−5	4	−9	17.30	11.40
5	4	9	15.50	11.70

−6	−4	−9	44.80	16.70
−6	−4	−9	33.40	19.00
−6	−4	−9	21.80	8.30
−6	4	−9	20.00	9.30
−6	4	−9	37.00	13.50
−6	4	−9	22.30	8.10
−6	4	−9	10.30	13.90
6	4	9	37.00	9.60
−7	−4	−9	16.40	7.50
−7	−4	−9	7.60	13.90
−7	−4	−9	9.40	15.10
7	4	9	−4.10	6.90
−7	4	−9	−1.90	9.00
−7	4	−9	7.00	14.10
−7	4	−9	−17.90	14.10
−8	−4	−9	−0.60	7.40
−8	−4	−9	0.90	15.70
−8	−4	−9	3.40	17.00
8	4	9	4.40	8.40
−8	4	−9	−4.10	16.10
−8	4	−9	1.90	10.50
−9	−4	−9	2.50	20.40
−9	−4	−9	−0.80	19.10
9	4	9	−5.00	9.30
−9	4	−9	13.90	25.50
−9	4	−9	−15.20	13.50
9	−5	−9	22.30	17.00
9	−5	−9	9.80	12.40
−9	−5	9	21.50	25.70
−9	−5	9	35.10	24.40
−9	5	9	−6.60	20.20

−9	5	9	−11.80	21.70
−9	5	9	10.70	20.40
8	−5	−9	26.40	15.00
−8	−5	9	26.80	20.70
−8	−5	9	20.90	19.20
8	−5	−9	24.50	11.80
−8	5	9	−23.00	15.90
8	5	−9	14.90	10.30
−8	5	9	0.90	21.30
7	−5	−9	45.00	14.50
7	−5	−9	14.90	9.70
−7	−5	9	45.50	11.70
−7	−5	9	21.50	18.60
−7	5	9	23.60	18.70
7	5	−9	25.40	11.10
−7	5	9	59.79	24.00
−6	−5	9	12.10	16.00
6	−5	−9	11.50	12.70
6	5	−9	1.10	7.30
−6	5	9	−0.10	15.30
−6	5	9	−1.20	13.10
5	−5	−9	26.50	13.00
−5	−5	9	21.90	14.20
5	−5	−9	26.90	10.40
−5	5	9	20.70	14.80
−5	5	9	41.30	18.30
5	5	−9	7.50	7.50
4	−5	−9	82.39	13.60
−4	−5	9	115.19	22.70
4	−5	−9	90.89	16.60
4	5	−9	86.29	12.10

4	5	-9	76.39	18.90
-4	5	9	46.60	17.00
-4	5	9	80.09	19.90
3	-5	-9	48.00	12.90
3	-5	-9	40.80	10.20
-3	-5	9	43.70	6.90
-3	-5	9	32.70	13.80
3	5	-9	23.80	12.80
3	5	-9	58.09	9.40
-3	5	9	78.19	20.40
-3	5	9	51.29	18.10
2	-5	-9	157.48	21.40
2	-5	-9	158.58	15.10
-2	-5	9	173.78	22.20
-2	-5	9	163.08	8.50
-2	5	9	146.89	25.40
2	5	-9	140.49	19.00
-2	5	9	145.59	16.30
-2	5	9	140.89	23.60
-1	-5	9	59.39	13.20
1	-5	-9	56.29	12.80
1	-5	-9	70.49	11.40
1	5	-9	58.39	8.50
-1	5	9	59.29	11.40
-1	5	9	62.79	17.10
1	5	-9	35.70	9.90
-1	5	9	57.89	16.90
0	-5	-9	42.60	13.40
0	-5	-9	52.59	10.50
0	-5	-9	34.00	10.40
0	-5	9	56.89	12.30

0	5	−9	47.40	7.40
0	5	−9	50.49	7.80
0	5	9	58.19	15.20
0	5	9	28.90	8.80
0	5	−9	34.80	9.80
0	5	9	33.60	14.40
−1	−5	−9	24.50	11.80
−1	−5	−9	21.60	8.00
−1	−5	−9	19.30	9.80
1	−5	9	−2.40	5.90
1	5	9	18.90	9.00
−1	5	−9	16.90	7.00
1	5	9	15.10	11.90
1	5	9	17.20	6.80
−1	5	−9	34.20	7.70
−2	−5	−9	54.59	11.20
−2	−5	−9	65.29	16.10
−2	−5	−9	42.80	10.70
−2	−5	−9	45.90	15.90
−2	5	−9	57.99	7.30
−2	5	−9	69.29	12.20
−2	5	−9	73.49	9.00
−2	5	−9	21.80	14.90
2	5	9	59.79	10.30
−2	5	−9	68.79	9.30
−2	5	−9	54.59	9.60
2	5	9	42.60	13.70
−3	−5	−9	135.89	16.00
−3	−5	−9	165.08	24.40
−3	−5	−9	141.79	18.20
−3	−5	−9	167.28	27.30

−3	5	−9	148.29	11.10
−3	5	−9	157.38	10.40
−3	5	−9	147.69	12.70
3	5	9	162.18	14.70
−3	5	−9	102.79	22.30
−3	5	−9	164.48	12.90
3	5	9	138.09	21.70
−3	5	−9	146.99	13.10
−4	−5	−9	198.78	26.80
−4	−5	−9	151.78	26.20
−4	−5	−9	175.28	16.90
−4	−5	−9	195.18	17.00
4	5	9	212.78	17.00
−4	5	−9	194.28	13.40
−4	5	−9	125.49	25.30
4	5	9	207.18	28.10
−4	5	−9	161.08	11.70
−4	5	−9	141.99	11.40
−4	5	−9	152.78	15.00
−4	5	−9	165.98	21.70
−5	−5	−9	1.50	7.10
−5	−5	−9	−8.60	12.70
−5	5	−9	5.70	5.90
−5	5	−9	3.30	4.90
−5	5	−9	−1.50	4.20
−5	5	−9	13.40	14.10
5	5	9	−4.90	5.80
−5	5	−9	6.10	9.00
−6	−5	−9	6.40	14.20
−6	−5	−9	7.50	15.80
−6	−5	−9	−0.60	7.10

−6	5	−9	11.00	13.10
6	5	9	−7.20	10.80
−6	5	−9	16.90	11.20
6	5	9	10.60	7.80
−6	5	−9	10.30	6.10
−6	5	−9	−4.10	6.90
−7	−5	−9	6.80	8.60
−7	−5	−9	2.80	16.60
−7	−5	−9	8.70	17.80
−7	5	−9	18.90	12.90
7	5	9	−3.90	7.40
−7	5	−9	26.10	10.00
−7	5	−9	28.40	17.50
−8	−5	−9	11.10	18.80
−8	−5	−9	0.50	8.50
−8	−5	−9	25.50	19.80
8	5	9	−0.60	8.50
−8	5	−9	9.90	18.40
−9	−5	−9	5.00	36.00
−9	−5	−9	26.40	20.50
9	5	9	−10.90	10.20
−9	5	−9	12.50	12.80
−9	−6	9	11.50	23.60
9	−6	−9	−17.10	15.90
−9	−6	9	23.10	23.50
9	−6	−9	22.50	14.20
9	6	−9	9.60	12.00
−9	6	9	−57.79	22.20
−9	6	9	−22.70	22.00
8	−6	−9	0.30	12.00
−8	−6	9	0.50	17.90

8	−6	−9	−11.80	13.40
−8	6	9	19.80	19.30
−8	6	9	−47.00	19.50
8	6	−9	3.40	9.30
7	−6	−9	16.90	10.70
−7	−6	9	7.00	18.10
7	−6	−9	−0.50	11.40
7	6	−9	15.60	10.60
−7	6	9	11.00	17.10
−7	6	9	15.10	22.90
6	−6	−9	40.30	11.70
6	−6	−9	71.99	16.60
−6	−6	9	52.59	19.20
−6	6	9	19.80	16.70
−6	6	9	57.29	24.10
6	6	−9	68.79	12.70
−5	−6	9	63.69	19.90
5	−6	−9	80.89	13.40
−5	6	9	56.29	18.50
5	6	−9	73.49	12.10
−5	6	9	92.99	25.20
4	−6	−9	26.40	10.10
4	−6	−9	13.90	11.50
−4	−6	9	17.40	12.80
−4	6	9	−0.40	12.70
4	6	−9	27.20	7.40
−4	6	9	20.30	12.40
4	6	−9	46.50	15.90
3	−6	−9	4.30	7.70
3	−6	−9	−7.50	6.60
−3	−6	9	3.00	4.60

−3	−6	9	−0.70	10.00
−3	6	9	14.40	13.20
3	6	−9	−2.40	10.70
3	6	−9	13.30	6.40
−3	6	9	6.40	13.20
2	−6	−9	94.09	13.80
−2	−6	9	84.49	5.40
−2	−6	9	98.39	17.70
2	−6	−9	81.39	16.50
−2	6	9	91.89	14.50
−2	6	9	113.29	24.40
−2	6	9	95.09	22.70
2	6	−9	84.79	15.30
1	−6	−9	47.10	13.00
1	−6	−9	74.19	12.10
−1	−6	9	91.99	16.00
−1	6	9	53.29	15.20
1	6	−9	82.59	9.60
−1	6	9	74.89	12.70
1	6	−9	57.59	11.60
−1	6	9	110.29	24.50
0	−6	−9	259.97	23.10
0	−6	9	332.27	23.60
0	−6	−9	321.27	21.10
0	−6	−9	314.87	26.60
0	6	9	349.87	31.50
0	6	−9	289.67	14.80
0	6	9	335.27	21.80
0	6	9	270.37	35.80
0	6	−9	275.17	13.80
0	6	−9	297.87	18.50

-1	-6	-9	24.50	10.20
-1	-6	-9	51.49	17.10
-1	-6	-9	35.00	13.10
-1	-6	-9	20.10	8.10
1	6	9	24.60	9.00
-1	6	-9	33.80	8.30
-1	6	-9	22.70	6.40
-1	6	-9	19.90	5.30
1	6	9	23.00	12.70
-1	6	-9	16.30	5.20
-1	6	-9	17.60	7.20
-2	-6	-9	21.90	9.20
-2	-6	-9	31.60	13.90
-2	-6	-9	28.60	14.90
-2	-6	-9	33.00	8.70
2	6	9	24.00	12.20
2	6	9	25.00	8.20
-2	6	-9	20.80	6.00
-2	6	-9	23.20	9.10
-2	6	-9	29.20	12.60
-3	-6	-9	3.90	14.60
-3	-6	-9	6.20	7.30
-3	-6	-9	0.70	11.30
-3	-6	-9	7.80	7.40
-3	6	-9	9.00	6.10
-3	6	-9	7.70	7.20
-3	6	-9	-8.80	4.60
3	6	9	5.30	6.50
-3	6	-9	5.00	11.80
3	6	9	17.40	12.50
-4	-6	-9	12.90	7.60

-4	-6	-9	22.10	14.70
-4	-6	-9	30.40	13.40
-4	-6	-9	-1.50	8.50
-4	6	-9	22.90	15.00
4	6	9	23.90	13.50
-4	6	-9	25.10	7.00
4	6	9	25.10	8.80
-4	6	-9	23.80	6.00
-4	6	-9	18.30	7.90
-5	-6	-9	61.79	17.80
-5	-6	-9	70.09	21.00
-5	-6	-9	26.40	9.40
-5	-6	-9	24.30	10.10
-5	6	-9	16.80	13.70
-5	6	-9	46.40	7.60
5	6	9	79.69	20.60
-5	6	-9	29.60	5.90
5	6	9	44.10	11.00
-6	-6	-9	20.80	16.00
-6	-6	-9	10.70	8.00
6	6	9	20.80	16.90
-6	6	-9	8.50	7.40
6	6	9	5.20	7.80
-6	6	-9	7.60	16.00
-6	6	-9	18.30	9.20
-7	-6	-9	11.90	9.00
-7	-6	-9	10.70	17.90
-7	-6	-9	33.30	18.20
7	6	9	4.40	8.90
-7	6	-9	18.90	7.70
-7	6	-9	2.90	10.00

-7	6	-9	-7.80	16.10
-8	-6	-9	22.10	20.10
-8	-6	-9	-3.60	9.60
-8	-6	-9	-8.00	18.50
-8	6	-9	-1.70	9.50
8	6	9	-4.90	9.30
-8	6	-9	-5.50	17.50
-9	-6	-9	-3.70	20.70
-9	6	-9	-16.30	11.50
-9	6	-9	-17.90	21.80
9	6	9	7.60	11.70
-8	-7	9	-17.30	21.60
8	-7	-9	28.20	13.40
8	-7	-9	11.40	14.30
-8	7	9	6.40	24.50
8	7	-9	5.10	9.90
-8	7	9	-13.20	23.40
7	-7	-9	10.90	11.20
-7	-7	9	-9.00	18.90
7	-7	-9	12.20	12.50
-7	7	9	-17.60	15.20
-7	7	9	20.80	20.30
7	7	-9	32.00	12.30
6	-7	-9	14.50	9.80
-6	-7	9	-1.20	16.80
6	-7	-9	13.50	12.00
-6	7	9	2.10	20.00
-6	7	9	41.60	23.10
6	7	-9	14.80	8.50
5	-7	-9	16.60	13.10
-5	-7	9	-5.10	14.10

5	-7	-9	12.50	8.40
5	7	-9	1.10	7.10
-5	7	9	-16.90	14.70
-5	7	9	9.90	16.00
-4	-7	9	110.59	10.70
4	-7	-9	168.78	18.60
-4	-7	9	153.58	24.70
-4	7	9	137.59	28.80
4	7	-9	118.09	21.40
4	7	-9	124.59	14.60
-4	7	9	140.39	28.70
3	-7	-9	57.29	11.90
-3	-7	9	77.89	7.20
-3	-7	9	59.79	6.20
-3	-7	9	74.49	20.00
3	-7	-9	53.69	16.40
-3	7	9	54.39	19.20
3	7	-9	81.19	11.40
-3	7	9	72.99	21.90
3	7	-9	57.49	14.50
-3	7	9	76.49	13.10
2	-7	-9	58.69	15.20
2	-7	-9	75.69	15.30
2	-7	-9	44.00	10.20
-2	-7	9	58.09	15.10
-2	7	9	50.89	18.30
2	7	-9	55.59	9.70
-2	7	9	40.80	18.70
-2	7	9	78.59	13.20
2	7	-9	61.99	12.20
1	-7	-9	24.40	10.50

-1	-7	9	13.00	11.30
1	-7	-9	12.80	10.50
1	-7	-9	2.10	8.70
1	-7	-9	19.00	7.60
-1	7	9	2.50	12.10
1	7	-9	-0.80	4.00
-1	7	9	19.10	17.20
1	7	-9	-3.40	7.10
-1	7	9	13.30	7.50
0	-7	-9	2.30	9.60
0	-7	-9	-1.90	6.20
0	-7	-9	-2.20	7.30
0	7	9	21.50	14.10
0	7	-9	4.80	4.90
0	7	-9	6.70	5.60
0	7	9	7.40	6.80
0	7	-9	12.90	4.50
0	7	-9	8.40	5.80
0	7	9	10.80	12.10
-1	-7	-9	-1.60	6.40
-1	-7	-9	-1.40	11.60
-1	-7	-9	-28.30	8.50
1	-7	9	3.50	7.10
-1	-7	-9	5.60	7.10
-1	7	-9	9.90	7.80
1	7	9	11.90	12.20
-1	7	-9	2.50	4.10
1	7	9	-0.50	5.80
-1	7	-9	20.80	6.90
-2	-7	-9	96.59	16.50
-2	-7	-9	108.59	22.10

-2	-7	-9	156.88	22.60
-2	-7	-9	113.09	15.80
2	7	9	133.49	26.70
-2	7	-9	139.89	17.50
-2	7	-9	89.49	21.20
-2	7	-9	118.99	9.90
2	7	9	122.19	15.90
-2	7	-9	118.69	17.50
-3	-7	-9	63.69	19.40
-3	-7	-9	52.19	11.60
-3	-7	-9	52.99	15.30
-3	-7	-9	63.79	13.20
-3	7	-9	50.19	14.30
-3	7	-9	58.59	7.10
-3	7	-9	64.09	7.70
3	7	9	40.00	10.70
3	7	9	56.89	17.90
-3	7	-9	64.49	18.80
-4	-7	-9	103.69	16.30
-4	-7	-9	71.99	25.40
-4	-7	-9	87.19	20.30
-4	-7	-9	86.89	14.60
-4	7	-9	69.59	19.40
4	7	9	71.39	20.80
-4	7	-9	103.09	9.20
-4	7	-9	96.59	8.80
4	7	9	79.89	14.00
-4	7	-9	106.29	11.90
-5	-7	-9	-0.10	14.90
-5	-7	-9	-4.80	12.70
-5	-7	-9	7.70	8.60

5	7	9	-12.10	10.20
-5	7	-9	-3.70	5.90
-5	7	-9	7.30	5.30
5	7	9	-1.90	7.40
-6	-7	-9	4.20	17.20
-6	-7	-9	8.00	9.70
6	7	9	1.70	7.50
6	7	9	3.40	13.90
-6	7	-9	1.20	15.00
-7	-7	-9	-2.00	17.50
-7	-7	-9	46.60	21.00
-7	-7	-9	22.70	11.00
7	7	9	17.50	10.20
-7	7	-9	6.70	18.10
7	7	9	14.00	17.30
-8	-7	-9	3.40	21.10
-8	-7	-9	27.50	13.00
-8	7	-9	-19.90	16.50
-8	7	-9	-1.20	19.40
8	7	9	34.50	12.20
8	-8	-9	-7.50	15.90
-8	-8	9	6.00	24.40
8	-8	-9	4.60	13.40
-8	8	9	2.60	22.80
-7	-8	9	11.90	20.40
7	-8	-9	-12.50	12.60
7	-8	-9	1.30	10.70
-7	8	9	15.10	23.40
7	8	-9	-2.60	8.70
-7	8	9	-7.30	19.60
-6	-8	9	9.00	20.40

6	−8	−9	59.79	11.90
6	−8	−9	28.80	14.10
−6	8	9	33.50	24.30
6	8	−9	35.10	10.30
−6	8	9	33.60	19.80
−5	−8	9	2.40	16.00
5	−8	−9	18.20	12.00
5	−8	−9	48.00	18.60
−5	8	9	31.30	18.80
−5	8	9	−0.50	16.30
5	8	−9	27.70	8.60
4	−8	−9	16.20	10.90
−4	−8	9	10.10	5.80
4	−8	−9	18.20	8.60
−4	−8	9	12.20	13.90
4	8	−9	6.00	14.60
4	8	−9	5.60	7.00
−4	8	9	−2.20	14.10
−4	8	9	8.50	15.80
3	−8	−9	33.40	14.20
3	−8	−9	49.99	16.00
3	−8	−9	37.60	10.50
−3	−8	9	34.40	13.10
3	8	−9	35.30	8.90
3	8	−9	14.60	13.30
−3	8	9	81.79	26.10
−3	8	9	70.79	22.40
2	−8	−9	71.59	13.00
2	−8	−9	118.89	17.20
2	−8	−9	60.79	17.90
−2	−8	9	91.09	17.80

2	-8	-9	85.99	16.30
-2	8	9	65.09	25.10
-2	8	9	64.79	20.60
-2	8	9	56.69	12.90
2	8	-9	95.69	14.90
2	8	-9	72.09	9.40
-1	-8	9	135.39	19.20
1	-8	-9	138.99	17.40
1	-8	-9	126.99	17.80
1	-8	-9	159.48	21.00
1	-8	-9	111.09	19.40
-1	8	9	132.59	16.30
1	8	-9	124.09	10.80
1	8	-9	141.29	14.20
-1	8	9	115.29	25.30
-1	8	9	154.88	28.10
0	-8	9	191.48	18.60
0	-8	-9	201.18	18.20
0	-8	-9	197.18	21.30
0	-8	-9	167.98	23.80
0	8	-9	190.08	10.30
0	8	-9	174.28	12.40
0	8	-9	167.38	11.00
0	8	9	244.48	36.20
0	8	9	217.88	32.30
0	8	9	217.58	19.60
1	-8	9	43.10	8.60
-1	-8	-9	44.60	13.60
-1	-8	-9	37.50	17.40
-1	-8	-9	29.60	12.10
-1	-8	-9	34.20	11.40

-1	8	-9	31.80	10.60
1	8	9	38.10	16.80
-1	8	-9	43.70	10.00
-1	8	-9	36.90	16.10
-1	8	-9	35.10	6.00
1	8	9	28.30	11.10
-2	-8	-9	15.70	10.90
-2	-8	-9	-1.20	7.50
-2	-8	-9	22.00	16.00
-2	8	-9	1.40	10.00
-2	8	-9	-1.40	12.70
-2	8	-9	9.90	4.90
-2	8	-9	-4.90	9.60
-3	-8	-9	54.69	12.00
-3	-8	-9	38.90	18.10
-3	-8	-9	45.70	13.30
-3	-8	-9	30.40	14.10
-3	8	-9	39.60	6.80
-3	8	-9	38.90	16.40
3	8	9	66.89	21.10
-3	8	-9	15.50	12.60
3	8	9	62.49	13.40
-3	8	-9	42.20	6.30
-4	-8	-9	-0.30	14.40
-4	-8	-9	20.30	7.90
-4	-8	-9	16.40	9.00
-4	-8	-9	-4.30	13.60
-4	8	-9	23.50	5.00
4	8	9	-2.20	15.40
-4	8	-9	14.30	13.20
-4	8	-9	26.40	12.80

−4	8	−9	19.40	5.10
4	8	9	16.20	10.10
−5	−8	−9	0.20	14.90
−5	−8	−9	−20.40	15.80
−5	−8	−9	7.20	10.30
−5	8	−9	−6.20	13.00
−5	8	−9	5.80	14.10
−5	8	−9	−3.40	4.70
5	8	9	5.50	14.60
5	8	9	6.50	8.10
−6	−8	−9	43.20	21.30
−6	−8	−9	10.50	10.80
−6	−8	−9	−9.80	15.60
6	8	9	28.70	16.60
−6	8	−9	26.40	18.40
−6	8	−9	12.80	14.90
6	8	9	−10.50	8.80
−7	−8	−9	−3.60	20.20
−7	−8	−9	9.30	21.70
7	8	9	6.50	10.70
7	8	9	9.30	17.70
−7	8	−9	28.00	21.00
−7	8	−9	17.40	20.10
−8	−8	−9	18.50	14.10
−8	−8	−9	17.40	26.20
−8	−8	−9	42.20	24.90
−8	8	−9	−22.30	19.70
8	8	9	−6.00	9.90
−8	8	−9	33.30	26.10
7	−9	−9	23.10	11.90
7	−9	−9	20.70	14.80

−7	−9	9	4.60	23.20
7	9	−9	23.90	10.80
−7	9	9	−4.40	21.70
−6	−9	9	12.30	20.30
6	−9	−9	4.20	10.10
6	−9	−9	−22.00	12.80
−6	9	9	27.70	20.90
6	9	−9	−8.90	9.80
−6	9	9	−0.80	17.80
5	−9	−9	18.70	15.70
−5	−9	9	0.70	17.30
5	−9	−9	14.10	10.50
−5	9	9	−3.60	18.30
5	9	−9	−13.40	7.40
−5	9	9	−14.70	16.60
4	−9	−9	9.30	9.20
4	−9	−9	2.60	11.20
−4	−9	9	−12.70	14.70
4	9	−9	17.30	7.80
−4	9	9	6.40	16.20
−4	9	9	8.50	9.70
−4	9	9	−22.80	12.90
−3	−9	9	26.30	14.30
3	−9	−9	11.10	7.60
3	−9	−9	36.40	20.40
3	−9	−9	10.80	12.10
3	9	−9	−0.60	11.90
3	9	−9	10.80	7.00
−3	9	9	15.90	18.60
−3	9	9	3.60	8.00
−3	9	9	19.80	17.80

-2	-9	9	24.30	13.60
2	-9	-9	40.40	12.30
2	-9	-9	13.40	8.30
2	-9	-9	34.00	11.70
-2	9	9	22.00	10.20
2	9	-9	30.20	10.20
-2	9	9	75.09	24.00
2	9	-9	41.60	8.60
-2	9	9	27.00	18.40
1	-9	-9	-18.30	10.60
1	-9	-9	-1.50	8.20
-1	-9	9	16.70	12.40
1	-9	-9	-3.30	9.90
-1	9	9	10.70	8.50
-1	9	9	-5.40	13.60
1	9	-9	-3.20	4.70
-1	9	9	-23.40	13.60
1	9	-9	12.40	7.20
0	-9	-9	28.90	13.60
0	-9	-9	17.60	9.20
0	-9	-9	33.20	12.40
0	-9	-9	49.30	21.80
0	9	-9	33.90	6.40
0	9	9	49.30	20.20
0	9	9	10.20	9.80
0	9	-9	26.60	6.10
-1	-9	-9	12.90	9.80
-1	-9	-9	42.70	18.00
-1	-9	-9	12.40	8.70
-1	-9	-9	6.90	10.80
1	9	9	-5.70	8.20

-1	9	-9	22.70	8.40
-1	9	-9	-18.60	12.90
1	9	9	24.80	16.10
-1	9	-9	-1.70	4.20
-2	-9	-9	10.20	16.90
-2	-9	-9	6.00	11.90
-2	-9	-9	-3.40	9.20
-2	-9	-9	4.60	7.00
-2	9	-9	-8.70	12.90
-2	9	-9	-3.30	4.30
2	9	9	4.60	8.90
2	9	9	7.30	13.20
-2	9	-9	19.60	12.60
-3	-9	-9	51.39	17.20
-3	-9	-9	60.29	16.00
-3	-9	-9	54.29	13.20
-3	9	-9	68.79	20.40
-3	9	-9	42.30	6.50
3	9	9	82.89	15.10
-3	9	-9	27.30	15.10
3	9	9	47.30	18.80
-4	-9	-9	44.10	19.20
-4	-9	-9	70.19	19.20
-4	-9	-9	31.80	12.40
4	9	9	31.20	20.40
-4	9	-9	86.29	21.10
4	9	9	49.90	13.00
-4	9	-9	50.99	6.70
-4	9	-9	27.20	16.90
-5	-9	-9	-3.50	19.40
-5	-9	-9	-5.70	10.50

-5	-9	-9	-11.10	14.50
5	9	9	6.60	10.30
5	9	9	14.80	17.90
-5	9	-9	12.10	15.50
-5	9	-9	-15.80	15.30
-6	-9	-9	-11.40	16.90
-6	-9	-9	-0.10	20.80
-6	-9	-9	17.80	12.40
6	9	9	-6.70	17.80
-6	9	-9	-9.70	15.80
6	9	9	1.00	10.70
-6	9	-9	5.20	18.90
-7	-9	-9	13.80	30.70
-7	-9	-9	25.60	22.30
-7	-9	-9	14.10	12.50
-7	9	-9	-6.30	22.50
7	9	9	16.20	11.60
7	9	9	-20.70	19.40
-7	9	-9	5.90	20.00
7	-10	-9	8.10	16.50
-7	-10	9	-12.90	26.20
7	-10	-9	-6.70	11.60
-7	10	9	-9.90	22.40
-7	10	9	33.60	26.30
7	10	-9	2.00	10.40
6	-10	-9	12.30	17.00
-6	-10	9	-6.00	22.30
6	-10	-9	7.00	11.60
6	10	-9	11.50	10.20
-6	10	9	27.10	23.00
-6	10	9	27.60	23.80

5	-10	-9	30.00	10.80
-5	-10	9	40.00	23.70
5	-10	-9	42.80	19.50
-5	10	9	-3.40	19.50
-5	10	9	45.70	21.10
5	10	-9	0.70	9.50
-4	-10	9	-13.40	16.40
4	-10	-9	12.50	13.60
-4	10	9	2.40	10.00
-4	10	9	4.30	16.90
4	10	-9	-8.70	7.40
-4	10	9	-6.90	15.40
3	-10	-9	0.80	8.80
3	-10	-9	4.10	12.50
3	-10	-9	8.70	11.20
-3	-10	9	-2.50	16.80
-3	10	9	5.90	17.20
3	10	-9	-4.40	6.10
-3	10	9	-17.70	7.60
-3	10	9	-1.60	16.90
2	-10	-9	6.10	11.60
-2	-10	9	-4.00	15.30
2	-10	-9	-3.50	9.50
2	-10	-9	17.30	9.70
2	10	-9	2.60	9.60
-2	10	9	10.00	16.30
-2	10	9	-0.10	9.00
2	10	-9	-4.50	5.90
-2	10	9	6.30	17.70
-1	-10	9	3.20	14.70
1	-10	-9	15.20	7.90

1	-10	-9	10.40	8.90
1	-10	-9	0.90	10.90
1	-10	-9	23.80	10.50
-1	10	9	-16.10	13.60
1	10	-9	8.80	6.10
-1	10	9	12.40	9.60
1	10	-9	11.30	5.60
0	-10	-9	12.80	18.50
0	-10	-9	29.50	11.40
0	-10	-9	22.40	15.40
0	-10	-9	8.90	10.20
0	-10	9	26.50	10.70
0	-10	-9	14.70	12.70
0	10	9	-1.10	9.10
0	10	-9	8.60	5.40
0	10	-9	11.80	5.60
0	10	9	-0.50	14.90
-1	-10	-9	-3.60	11.10
-1	-10	-9	28.20	18.90
-1	-10	-9	10.40	9.20
-1	-10	-9	24.10	10.80
1	10	9	10.30	15.00
-1	10	-9	-15.40	9.50
-1	10	-9	2.50	4.60
1	10	9	14.50	10.80
-1	10	-9	-6.60	14.10
-2	-10	-9	-8.10	10.90
-2	-10	-9	-13.30	10.30
-2	-10	-9	3.20	19.80
-2	-10	-9	-2.80	8.30
-2	10	-9	19.00	13.30

-2	10	-9	-8.50	12.80
2	10	9	17.50	9.60
2	10	9	15.00	16.50
-2	10	-9	5.60	5.00
-3	-10	-9	8.30	10.30
-3	-10	-9	34.00	13.20
-3	-10	-9	-2.20	16.30
-3	10	-9	21.80	15.90
-3	10	-9	-18.90	13.30
-3	10	-9	-10.00	4.40
3	10	9	4.00	10.00
3	10	9	3.90	15.60
-4	-10	-9	-14.70	15.00
-4	-10	-9	-13.50	19.20
-4	-10	-9	-0.70	11.50
4	10	9	-10.50	14.40
4	10	9	-8.30	8.80
-4	10	-9	-23.50	15.40
-5	-10	-9	-7.40	19.30
-5	-10	-9	-34.70	10.80
-5	-10	-9	12.00	21.90
5	10	9	5.50	10.60
5	10	9	3.10	15.80
-5	10	-9	-4.50	16.00
-6	-10	-9	-1.30	13.00
-6	-10	-9	17.80	20.40
-6	-10	-9	-23.20	24.00
6	10	9	-12.00	10.20
-6	10	-9	9.10	22.60
-6	10	-9	-22.50	17.90
6	-11	-9	9.70	11.60

-6	11	9	-4.20	22.00
-6	11	9	-18.30	23.00
6	11	-9	-6.70	9.70
5	-11	-9	7.40	13.30
5	-11	-9	-6.10	10.00
5	11	-9	-6.10	9.00
-5	11	9	-1.10	17.90
-5	11	9	-3.20	10.20
4	-11	-9	41.10	19.10
4	-11	-9	11.70	10.10
-4	11	9	-0.80	20.10
-4	11	9	19.10	20.50
4	11	-9	-8.70	9.40
-4	11	9	3.10	10.10
3	-11	-9	14.20	16.20
3	-11	-9	49.20	17.10
-3	11	9	17.50	18.40
-3	11	9	1.10	17.60
3	11	-9	12.10	8.00
-3	11	9	11.60	12.80
2	-11	-9	-12.40	12.50
2	-11	-9	-9.50	9.70
-2	11	9	13.00	17.70
-2	11	9	11.10	10.60
-2	11	9	9.80	17.10
2	11	-9	-5.50	5.90
1	-11	-9	17.10	21.10
1	-11	-9	8.90	8.90
1	-11	-9	16.20	12.90
-1	11	9	-25.90	16.00
-1	11	9	1.50	10.70

0	-11	-9	-16.50	8.00
0	-11	-9	11.50	11.00
0	-11	-9	36.30	22.80
0	11	9	27.20	12.50
0	11	-9	-0.60	5.70
0	11	9	-6.10	16.50
-1	-11	-9	-1.10	11.20
-1	-11	-9	3.30	8.80
-1	-11	-9	3.40	9.70
-1	-11	-9	6.90	19.90
-1	11	-9	8.60	14.30
1	11	9	1.40	9.90
-1	11	-9	-22.60	11.40
1	11	9	-2.90	19.10
-1	11	-9	2.30	5.30
-2	-11	-9	23.60	13.30
-2	-11	-9	12.40	20.40
-2	-11	-9	4.40	13.00
-2	-11	-9	-2.00	8.90
2	11	9	7.60	10.70
-2	11	-9	-4.80	16.20
2	11	9	-1.30	18.40
-2	11	-9	17.90	16.10
-3	-11	-9	-15.20	11.90
-3	-11	-9	15.70	23.30
-3	-11	-9	-8.10	21.10
3	11	9	-6.10	19.10
3	11	9	17.10	11.90
-3	11	-9	4.60	17.40
-3	11	-9	-6.10	15.30
-4	-11	-9	-20.20	22.90

-4	-11	-9	-0.30	14.30
-4	-11	-9	7.50	17.40
4	11	9	-32.10	19.20
-4	11	-9	-15.90	19.20
-4	11	-9	-18.70	17.70
4	11	9	3.70	10.20
-5	-11	-9	-10.20	19.40
-5	-11	-9	-23.20	24.40
-5	-11	-9	3.80	11.30
-5	11	-9	10.10	18.70
-5	11	-9	28.40	23.50
5	11	9	22.90	13.70
-6	-11	-9	-31.00	21.20
-6	-11	-9	18.70	16.20
6	11	9	12.50	26.00
-6	11	-9	16.40	26.30
-6	11	-9	24.00	20.80
6	11	9	-15.10	11.50
5	-12	-9	-2.20	11.90
5	-12	-9	6.50	17.20
5	12	-9	-6.40	10.00
-5	12	9	20.60	23.20
-5	12	9	-6.00	14.00
-5	12	9	17.30	24.10
4	-12	-9	-8.20	10.00
-4	12	9	6.40	21.00
4	12	-9	-13.70	8.70
-4	12	9	2.70	12.70
3	-12	-9	7.10	11.10
3	-12	-9	7.60	16.80
-3	12	9	-6.40	19.50

-3	12	9	4.70	11.00
3	12	-9	8.20	8.80
-3	12	9	32.30	22.60
2	-12	-9	16.40	26.10
2	-12	-9	6.80	13.90
2	-12	-9	4.70	10.40
2	12	-9	-8.60	7.00
-2	12	9	4.00	11.20
-2	12	9	42.40	25.60
1	-12	-9	26.90	10.40
1	-12	-9	46.20	27.90
1	-12	-9	-22.40	12.10
1	12	-9	-3.30	6.50
-1	12	9	-24.00	20.20
-1	12	9	7.60	11.20
0	-12	-9	-1.90	10.60
0	-12	-9	-5.90	13.50
0	12	9	-2.60	20.30
0	12	-9	-12.80	17.10
0	12	9	36.70	13.30
0	12	-9	-7.40	5.50
-1	-12	-9	11.70	13.60
-1	-12	-9	13.60	22.80
-1	12	-9	-4.60	16.80
1	12	9	1.10	20.00
-1	12	-9	44.50	14.00
1	12	9	-2.00	11.60
-2	-12	-9	6.70	16.30
-2	-12	-9	16.70	23.50
2	12	9	2.00	12.10
-2	12	-9	14.50	18.80

2	12	9	-20.30	20.30
-2	12	-9	12.10	18.80
-3	-12	-9	8.60	25.20
-3	-12	-9	-14.60	11.70
3	12	9	29.10	23.30
3	12	9	7.00	12.40
-3	12	-9	25.10	21.60
-4	-12	-9	-9.10	27.70
-4	-12	-9	-14.80	12.50
4	12	9	-20.70	20.10
-4	12	-9	9.10	18.60
4	12	9	4.90	12.20
-4	12	-9	39.10	24.60
3	-13	-9	17.30	12.20
3	-13	-9	-3.00	16.40
-3	13	9	4.60	12.20
3	13	-9	18.00	11.30
-3	13	9	-10.40	22.30
2	-13	-9	-5.70	11.30
2	-13	-9	21.60	21.00
2	13	-9	-1.20	8.40
-2	13	9	-24.50	19.70
-2	13	9	8.90	11.90
1	-13	-9	-7.60	15.60
1	-13	-9	5.10	12.50
1	-13	-9	32.10	29.00
-1	13	9	0.60	11.80
1	13	-9	-0.50	7.30
-1	13	9	2.30	20.80
0	-13	-9	4.20	19.10
0	-13	-9	43.40	29.40

0	13	9	18.60	12.80
0	13	-9	-3.60	20.60
-1	-13	-9	-1.20	16.70
-1	-13	-9	23.00	27.10
1	13	9	25.40	24.50
-1	13	-9	1.70	19.50
1	13	9	4.20	14.60
-2	-13	-9	-15.70	27.90
-2	-13	-9	-18.40	16.70
2	13	9	16.30	15.70
2	13	9	2.90	23.90
-3	-13	-9	11.60	16.80
-3	-13	-9	67.89	31.20
-3	13	-9	-70.29	23.30
-3	13	-9	-23.00	19.40
3	13	9	26.00	25.10
3	13	9	17.10	13.20
-9	0	10	-12.00	23.00
-9	0	10	6.70	20.70
9	0	-10	-2.60	13.50
9	0	-10	-19.20	10.30
-9	0	10	-4.50	19.50
8	0	-10	-11.10	12.70
8	0	-10	-0.80	10.60
-8	0	10	-21.90	15.30
-8	0	10	-2.10	17.50
7	0	-10	-4.30	7.70
-7	0	10	26.40	16.00
7	0	-10	8.50	10.40
-7	0	10	0.90	14.90
6	0	-10	116.39	16.20

-6	0	10	79.39	18.70
-6	0	10	108.69	24.50
5	0	-10	1.00	7.30
-5	0	10	11.40	11.80
-5	0	10	3.60	10.10
4	0	-10	149.69	21.90
-4	0	10	213.18	30.60
-4	0	10	217.38	20.90
4	0	-10	174.18	15.80
3	0	-10	1.20	5.70
-3	0	10	-3.50	9.00
3	0	-10	-22.90	9.80
-3	0	10	6.40	7.20
2	0	-10	51.49	14.30
2	0	-10	34.20	10.20
-2	0	10	50.69	9.50
2	0	-10	46.70	10.70
-2	0	10	37.40	12.70
-2	0	10	42.50	10.30
-1	0	10	-8.30	9.40
1	0	-10	-12.40	8.70
1	0	-10	-4.90	10.40
-1	0	10	-0.10	6.40
1	0	-10	-1.90	6.00
1	0	-10	10.50	7.20
-1	0	10	-1.70	4.40
0	0	-10	17.90	9.10
0	0	-10	-1.40	11.20
0	0	-10	17.80	8.20
0	0	10	20.40	10.30
0	0	10	30.10	8.10

0	0	-10	24.10	7.40
1	0	10	-5.10	5.30
1	0	10	5.40	6.50
-1	0	-10	1.70	7.30
-1	0	-10	4.30	8.00
1	0	10	6.10	10.30
-1	0	-10	-5.40	4.70
-1	0	-10	6.60	12.60
1	0	10	-4.50	3.60
2	0	10	32.30	12.60
-2	0	-10	35.00	14.40
-2	0	-10	25.80	8.10
2	0	10	45.90	12.30
-2	0	-10	9.00	7.40
2	0	10	30.00	7.70
2	0	10	28.80	6.00
-2	0	-10	18.40	9.70
-3	0	-10	-12.00	9.40
-3	0	-10	5.50	9.10
-3	0	-10	-8.80	5.10
3	0	10	-2.30	5.70
-3	0	-10	-2.90	5.40
3	0	10	3.90	3.90
3	0	10	4.20	8.80
-4	0	-10	129.09	22.60
-4	0	-10	102.79	12.10
-4	0	-10	119.09	14.40
4	0	10	103.69	12.20
-4	0	-10	81.49	20.20
-4	0	-10	169.78	24.40
-5	0	-10	5.50	4.70

-5	0	-10	2.30	11.40
-5	0	-10	-6.10	5.80
5	0	10	-0.90	4.50
-5	0	-10	6.10	10.20
-5	0	-10	5.70	14.50
6	0	10	9.00	5.60
-6	0	-10	12.00	7.10
-6	0	-10	14.50	13.20
-6	0	-10	13.90	3.60
-7	0	-10	-15.30	14.00
-7	0	-10	2.60	13.30
7	0	10	-7.00	5.80
-8	0	-10	27.40	20.00
-8	0	-10	-3.30	14.30
8	0	10	14.40	8.30
-9	0	-10	-12.70	17.60
-9	0	-10	-25.00	17.10
9	0	10	-19.90	8.90
-9	-1	10	4.50	19.70
-9	-1	10	21.70	22.60
9	-1	-10	-10.90	9.30
-9	-1	10	-23.20	18.30
9	-1	-10	14.30	14.90
-9	1	10	6.90	23.00
9	1	-10	-14.80	10.40
-9	1	10	-21.80	18.40
-9	1	10	-9.30	17.90
-8	-1	10	-31.10	15.70
8	-1	-10	-0.80	13.80
-8	-1	10	11.50	18.30
8	-1	-10	-10.70	9.50

-8	1	10	8.50	17.80
8	1	-10	-5.50	9.10
-8	1	10	-10.00	15.20
7	-1	-10	13.20	9.60
-7	-1	10	7.60	14.20
-7	-1	10	19.00	16.00
7	-1	-10	8.40	13.00
-7	1	10	-19.30	15.90
7	1	-10	2.00	8.40
-7	1	10	25.70	16.40
6	-1	-10	24.10	12.90
-6	-1	10	8.30	12.20
6	-1	-10	20.60	8.60
-6	-1	10	33.10	17.50
-6	1	10	7.70	15.10
-6	1	10	8.50	13.80
6	1	-10	22.10	9.70
5	-1	-10	-6.70	6.80
-5	-1	10	7.60	13.40
5	-1	-10	11.60	8.60
-5	-1	10	-11.20	8.80
-5	1	10	-8.10	11.40
-5	1	10	-3.60	11.00
5	1	-10	3.10	7.30
4	-1	-10	-1.80	7.70
4	-1	-10	-0.10	6.30
-4	-1	10	4.10	10.80
-4	-1	10	6.10	7.90
-4	1	10	0.60	9.50
4	1	-10	-3.20	6.30
-4	1	10	14.90	11.70

3	-1	-10	-0.70	8.10
-3	-1	10	2.60	10.60
3	-1	-10	0.30	6.20
3	1	-10	12.90	9.40
-3	1	10	6.80	10.00
3	1	-10	5.60	6.00
-3	1	10	-5.90	7.70
-2	-1	10	56.59	14.80
2	-1	-10	55.29	12.40
-2	-1	10	34.90	7.60
2	-1	-10	51.69	10.30
2	1	-10	58.09	10.40
-2	1	10	55.49	13.40
2	1	-10	68.79	18.20
2	1	-10	50.49	12.10
-2	1	10	49.00	9.70
-2	1	10	46.60	14.40
-1	-1	10	8.80	5.60
1	-1	-10	1.30	7.00
-1	-1	10	9.90	9.90
1	-1	-10	14.70	6.40
-1	-1	10	6.20	5.20
1	1	-10	0.70	8.30
-1	1	10	2.10	5.10
1	1	-10	-11.20	11.50
-1	1	10	-4.60	6.20
-1	1	10	4.70	10.90
1	1	-10	7.50	5.40
0	-1	-10	84.19	14.40
0	-1	10	54.59	7.90
0	-1	10	98.19	8.60

0	−1	−10	39.00	16.70
0	−1	10	116.79	21.30
0	−1	10	100.19	13.40
0	−1	−10	84.69	13.10
0	1	10	87.89	11.40
0	1	10	61.99	13.20
0	1	−10	76.89	11.30
0	1	−10	90.79	14.10
0	1	10	68.89	18.30
0	1	−10	105.19	20.00
−1	−1	−10	14.10	11.60
−1	−1	−10	−3.80	6.20
−1	−1	−10	−2.50	5.70
−1	−1	−10	5.50	12.90
1	−1	10	11.80	5.30
1	−1	10	1.20	3.10
1	−1	10	3.80	6.80
−1	1	−10	−4.50	8.60
−1	1	−10	−9.70	9.70
−1	1	−10	8.00	6.20
−1	1	−10	−1.90	4.90
1	1	10	−2.00	5.90
1	1	10	−2.20	11.50
1	1	10	−0.30	4.40
−2	−1	−10	171.68	26.50
−2	−1	−10	162.98	26.10
2	−1	10	153.88	22.70
−2	−1	−10	149.49	17.70
−2	−1	−10	155.68	16.80
−2	−1	−10	131.69	20.80
2	−1	10	162.88	9.90

2	-1	10	132.29	10.50
-2	1	-10	138.49	15.30
2	1	10	157.78	12.20
-2	1	-10	178.48	27.00
-2	1	-10	172.48	15.90
2	1	10	175.38	18.70
-2	1	-10	170.58	27.80
2	1	10	134.79	20.30
-2	1	-10	208.68	21.20
-3	-1	-10	-6.20	11.20
3	-1	10	-1.70	3.40
-3	-1	-10	-16.30	5.60
-3	-1	-10	-0.30	5.80
-3	-1	-10	7.70	10.00
3	-1	10	-11.40	4.60
-3	-1	-10	4.40	13.70
3	1	10	5.90	10.30
-3	1	-10	1.10	6.30
-3	1	-10	-0.80	9.40
3	1	10	2.40	4.50
-3	1	-10	-0.10	6.00
-3	1	-10	-0.10	8.20
-3	1	-10	-8.30	11.50
-4	-1	-10	11.80	6.90
-4	-1	-10	2.60	5.60
-4	-1	-10	2.20	9.50
-4	-1	-10	16.50	12.40
4	-1	10	3.30	5.70
4	-1	10	3.00	3.60
4	1	10	0.10	4.40
-4	1	-10	-7.40	10.40

-4	1	-10	-5.70	8.70
-4	1	-10	1.70	9.00
-4	1	-10	11.00	5.80
-4	1	-10	-0.90	12.70
5	-1	10	1.30	3.80
-5	-1	-10	-5.50	10.20
5	-1	10	-1.10	6.30
-5	-1	-10	-1.90	6.20
-5	-1	-10	6.90	10.70
-5	1	-10	4.20	6.40
-5	1	-10	6.90	9.30
-5	1	-10	-9.10	12.90
-5	1	-10	23.70	13.30
5	1	10	2.70	4.80
-5	1	-10	-4.00	3.90
-6	-1	-10	54.19	18.70
-6	-1	-10	35.10	10.90
-6	-1	-10	29.60	12.80
-6	-1	-10	45.20	6.80
6	-1	10	59.09	7.90
-6	1	-10	47.40	11.00
-6	1	-10	70.89	20.80
6	1	10	64.69	9.60
-6	1	-10	49.90	20.00
-6	1	-10	63.19	15.70
-7	-1	-10	-15.50	15.80
-7	-1	-10	4.30	14.70
7	-1	10	1.60	5.10
-7	1	-10	3.20	13.00
7	1	10	8.30	6.30
-7	1	-10	-14.50	15.00

-8	-1	-10	10.10	18.20
-8	-1	-10	-6.30	15.30
8	1	10	3.80	8.90
-8	1	-10	-5.00	12.40
-8	1	-10	2.30	20.70
-9	-1	-10	18.30	21.90
-9	-1	-10	-20.50	17.40
-9	1	-10	-34.10	17.30
9	1	10	-3.80	9.60
-9	1	-10	34.40	18.50
-9	-2	10	-23.20	21.20
-9	-2	10	-13.50	21.50
9	-2	-10	-9.60	15.20
9	-2	-10	-15.80	11.60
-9	-2	10	-4.80	19.90
-9	2	10	-31.60	18.40
-9	2	10	-40.70	19.00
-9	2	10	19.60	20.30
9	2	-10	-3.90	20.60
-8	-2	10	-29.90	15.50
-8	-2	10	31.60	19.80
8	-2	-10	-5.50	14.10
8	-2	-10	2.40	9.90
-8	2	10	-7.30	17.60
8	2	-10	-6.70	8.80
-8	2	10	14.90	18.60
7	-2	-10	5.70	7.90
7	-2	-10	-1.20	12.70
-7	-2	10	5.00	12.10
-7	2	10	14.40	15.00
7	2	-10	1.60	8.70

-7	2	10	1.00	16.30
6	-2	-10	31.80	10.30
6	-2	-10	26.00	13.00
-6	-2	10	39.60	16.30
-6	-2	10	35.90	11.90
6	2	-10	30.40	9.00
-6	2	10	14.20	14.20
-5	-2	10	12.30	14.20
5	-2	-10	8.10	8.30
5	-2	-10	4.40	9.80
-5	2	10	20.00	14.60
5	2	-10	10.40	6.50
-5	2	10	9.60	13.10
-4	-2	10	14.20	7.60
4	-2	-10	-6.80	7.30
4	-2	-10	0.90	6.00
-4	-2	10	-4.80	10.10
-4	2	10	2.30	11.30
-4	2	10	-5.10	9.10
4	2	-10	5.10	6.40
3	-2	-10	50.19	10.90
-3	-2	10	52.19	16.00
3	-2	-10	37.50	11.60
-3	-2	10	36.10	7.60
-3	2	10	31.90	12.70
3	2	-10	39.00	9.00
-3	2	10	31.50	16.30
3	2	-10	39.80	10.10
-2	-2	10	271.57	15.80
-2	-2	10	268.87	15.60
2	-2	-10	270.97	23.70

2	-2	-10	288.07	19.60
-2	-2	10	238.48	28.50
2	2	-10	211.58	19.60
2	2	-10	253.07	17.30
2	2	-10	291.27	29.50
-2	2	10	320.07	37.40
-2	2	10	258.97	24.10
-2	2	10	250.77	18.10
-1	-2	10	3.30	11.00
1	-2	-10	16.50	8.20
-1	-2	10	17.10	5.50
-1	-2	10	22.50	5.60
-1	-2	10	24.60	7.10
1	-2	-10	32.40	10.50
-1	2	10	22.90	7.90
1	2	-10	32.20	8.40
-1	2	10	22.80	9.30
1	2	-10	20.30	12.40
1	2	-10	22.00	9.00
-1	2	10	1.10	10.40
0	-2	10	1.00	4.20
0	-2	10	11.50	6.90
0	-2	10	-1.10	8.40
0	-2	-10	-2.80	4.60
0	-2	-10	-1.30	7.10
0	-2	10	1.10	3.00
0	2	-10	7.20	6.00
0	2	-10	-0.70	9.90
0	2	10	-0.30	6.50
0	2	10	-4.40	4.10
0	2	-10	-12.90	6.50

0	2	10	12.90	9.40
-1	-2	-10	13.00	9.40
1	-2	10	9.70	5.00
1	-2	10	5.80	4.00
-1	-2	-10	22.50	9.40
-1	-2	-10	11.30	6.50
1	-2	10	16.10	10.40
-1	2	-10	28.30	8.50
-1	2	-10	11.80	6.70
1	2	10	19.50	10.20
1	2	10	11.20	6.20
-1	2	-10	-18.00	9.80
-2	-2	-10	49.90	17.60
-2	-2	-10	49.30	11.50
2	-2	10	37.10	10.40
-2	-2	-10	34.30	8.80
2	-2	10	56.69	6.40
2	-2	10	58.89	5.50
-2	-2	-10	46.30	14.50
2	2	10	50.59	9.80
2	2	10	37.50	14.00
-2	2	-10	46.80	12.20
-2	2	-10	63.09	17.50
-2	2	-10	59.19	10.20
-2	2	-10	43.80	15.50
-2	2	-10	45.10	8.90
-3	-2	-10	35.40	10.10
3	-2	10	43.80	4.80
-3	-2	-10	25.60	13.90
-3	-2	-10	37.60	9.20
3	-2	10	49.50	5.80

−3	−2	−10	66.39	19.60
−3	2	−10	56.09	18.90
3	2	10	33.60	13.30
−3	2	−10	69.59	16.00
−3	2	−10	55.89	10.20
−3	2	−10	44.10	11.90
3	2	10	47.40	8.40
−4	−2	−10	8.50	11.70
−4	−2	−10	18.60	14.50
−4	−2	−10	22.90	7.70
4	−2	10	17.20	3.50
4	−2	10	19.00	4.20
−4	−2	−10	25.00	8.50
−4	2	−10	12.30	7.10
4	2	10	9.40	11.30
−4	2	−10	−8.70	10.70
−4	2	−10	24.90	14.00
−4	2	−10	15.60	9.20
−4	2	−10	6.20	6.10
4	2	10	32.20	7.40
−4	2	−10	6.40	5.00
5	−2	10	6.40	3.50
−5	−2	−10	6.80	13.20
−5	−2	−10	−0.30	5.70
−5	−2	−10	6.90	7.70
−5	−2	−10	−1.40	12.30
−5	2	−10	−5.80	10.70
5	2	10	4.40	5.70
−5	2	−10	17.60	4.40
−5	2	−10	18.40	6.10
−5	2	−10	15.40	13.30

-5	2	-10	31.40	11.00
-6	-2	-10	28.00	14.50
6	-2	10	9.70	3.20
-6	-2	-10	12.80	15.50
-6	-2	-10	10.10	6.20
-6	-2	-10	5.00	8.70
-6	2	-10	16.80	15.90
6	2	10	4.20	6.30
-6	2	-10	13.20	10.70
-6	2	-10	25.60	16.40
-6	2	-10	15.10	7.00
-7	-2	-10	-9.30	14.10
-7	-2	-10	-3.80	5.00
-7	-2	-10	14.10	16.40
7	2	10	1.40	6.90
-7	2	-10	-5.90	10.30
-7	2	-10	14.10	15.30
-8	-2	-10	69.29	22.90
-8	-2	-10	-2.10	18.90
8	2	10	-0.20	12.40
-8	2	-10	6.20	18.30
-8	2	-10	-23.00	12.00
-9	-2	-10	20.60	19.30
-9	-2	-10	-31.20	16.40
-9	2	-10	-3.80	21.40
-9	2	-10	-4.20	15.50
9	2	10	-11.50	9.80
-9	-3	10	-0.50	22.10
9	-3	-10	8.10	16.40
9	-3	-10	-3.20	12.70
-9	-3	10	-27.20	19.00

9	3	-10	10.00	14.10
-9	3	10	-14.10	22.20
-8	-3	10	17.30	20.00
8	-3	-10	8.90	10.30
8	-3	-10	4.50	15.30
-8	3	10	26.20	20.00
8	3	-10	-4.10	9.70
-8	3	10	-6.20	16.80
7	-3	-10	55.29	17.20
-7	-3	10	38.10	14.80
-7	-3	10	59.89	24.30
7	-3	-10	33.20	10.80
-7	3	10	34.50	20.00
-7	3	10	89.99	23.20
7	3	-10	30.80	10.80
6	-3	-10	9.30	10.70
-6	-3	10	16.30	9.30
-6	-3	10	44.20	18.50
6	-3	-10	24.60	8.90
-6	3	10	10.70	16.80
6	3	-10	6.10	9.40
-6	3	10	34.10	16.40
5	-3	-10	70.69	13.10
5	-3	-10	113.49	21.30
-5	-3	10	53.09	9.80
-5	-3	10	62.19	18.00
-5	3	10	55.69	19.50
-5	3	10	49.99	18.80
5	3	-10	66.59	12.30
4	-3	-10	5.60	5.70
-4	-3	10	1.30	12.40

4	-3	-10	9.30	8.70
-4	-3	10	6.40	7.70
-4	3	10	-14.30	9.60
-4	3	10	0.20	12.90
4	3	-10	14.60	6.50
3	-3	-10	18.60	11.40
-3	-3	10	21.20	4.90
-3	-3	10	11.40	12.60
3	-3	-10	32.90	9.70
3	3	-10	7.90	6.40
-3	3	10	8.50	11.50
-3	3	10	24.60	11.00
3	3	-10	35.20	14.50
-2	-3	10	33.30	7.60
-2	-3	10	20.80	5.90
-2	-3	10	19.00	10.00
2	-3	-10	43.50	12.90
2	-3	-10	29.60	9.20
-2	-3	10	20.50	6.00
2	3	-10	46.50	16.30
2	3	-10	27.30	7.40
-2	3	10	35.60	11.90
-2	3	10	12.00	13.90
2	3	-10	18.80	7.20
-2	3	10	23.20	7.70
1	-3	-10	363.16	22.50
-1	-3	10	348.07	14.80
-1	-3	10	391.76	30.70
1	-3	-10	386.06	28.00
-1	-3	10	360.86	15.30
-1	3	10	384.16	21.70

1	3	-10	297.87	20.40
1	3	-10	344.37	19.30
1	3	-10	334.87	28.90
-1	3	10	417.66	31.00
-1	3	10	384.66	36.70
0	-3	10	54.89	6.10
0	-3	-10	55.79	12.50
0	-3	10	50.69	14.30
0	-3	-10	42.00	9.80
0	-3	10	49.50	5.80
0	3	10	71.39	14.80
0	3	-10	64.99	11.00
0	3	10	47.70	8.90
0	3	-10	72.19	15.00
0	3	10	24.00	13.10
0	3	-10	74.89	10.90
-1	-3	-10	269.97	29.00
-1	-3	-10	225.98	21.10
1	-3	10	315.87	25.60
1	-3	10	267.97	9.40
-1	-3	-10	244.28	18.70
1	3	10	254.07	17.30
1	3	10	278.67	34.00
-1	3	-10	300.67	19.90
-1	3	-10	229.08	25.20
1	3	10	274.97	25.70
-1	3	-10	271.57	17.20
2	-3	10	183.58	18.40
-2	-3	-10	145.19	16.70
-2	-3	-10	92.79	18.80
-2	-3	-10	126.39	17.00

-2	-3	-10	141.99	23.10
-2	3	-10	134.09	12.50
2	3	10	144.39	13.00
-2	3	-10	129.79	19.00
-2	3	-10	127.09	17.00
-2	3	-10	154.38	26.30
2	3	10	137.79	22.00
-3	-3	-10	-5.90	10.80
-3	-3	-10	-5.40	5.70
-3	-3	-10	0.60	9.70
-3	-3	-10	2.60	6.30
-3	3	-10	3.80	10.90
-3	3	-10	2.20	5.30
3	3	10	11.30	11.10
-3	3	-10	5.80	6.50
-3	3	-10	3.60	5.60
3	3	10	-1.40	5.40
-3	3	-10	4.20	7.30
-3	3	-10	12.90	11.20
-4	-3	-10	7.60	7.40
-4	-3	-10	20.50	13.50
-4	-3	-10	1.00	11.20
-4	3	-10	9.10	5.20
-4	3	-10	-1.20	11.20
4	3	10	8.90	5.60
4	3	10	11.10	13.20
-4	3	-10	6.60	6.80
-4	3	-10	2.90	7.20
-4	3	-10	-3.50	11.40
-5	-3	-10	53.09	17.60
-5	-3	-10	44.20	9.50

-5	-3	-10	67.09	13.00
5	3	10	48.90	15.60
5	3	10	50.19	9.90
-5	3	-10	43.70	11.30
-5	3	-10	64.29	17.90
-5	3	-10	67.29	10.40
-5	3	-10	49.90	5.70
-5	3	-10	67.09	6.60
-6	-3	-10	25.70	19.30
-6	-3	-10	33.30	16.70
-6	-3	-10	28.90	7.80
-6	-3	-10	36.40	11.60
-6	3	-10	33.20	8.80
-6	3	-10	37.50	15.30
-6	3	-10	45.50	13.00
6	3	10	40.10	9.30
-6	3	-10	38.30	20.00
-7	-3	-10	70.39	21.70
-7	-3	-10	21.90	16.20
-7	-3	-10	28.50	7.80
7	3	10	22.10	8.90
-7	3	-10	31.00	13.70
-7	3	-10	1.20	16.00
-8	-3	-10	-9.60	16.90
-8	-3	-10	-0.10	16.20
-8	3	-10	6.00	13.30
8	3	10	-9.40	7.70
-8	3	-10	-1.70	18.10
-9	-3	-10	11.40	21.90
-9	-3	-10	0.00	19.00
-9	3	-10	-12.50	15.00

-9	3	-10	-2.20	17.30
9	3	10	-16.10	10.00
-9	-4	10	5.20	23.20
9	-4	-10	-3.70	14.50
9	-4	-10	4.00	13.00
-9	4	10	-9.80	23.70
-9	4	10	-14.10	19.80
9	4	-10	14.90	11.50
8	-4	-10	6.30	12.20
-8	-4	10	-4.40	20.80
-8	-4	10	12.30	15.00
8	-4	-10	-23.60	14.20
-8	4	10	-0.10	19.40
-8	4	10	3.10	20.50
8	4	-10	-13.60	9.20
-7	-4	10	18.40	10.60
7	-4	-10	3.40	8.90
-7	-4	10	11.90	18.50
7	-4	-10	-13.10	10.90
7	4	-10	2.50	8.80
-7	4	10	21.30	18.00
-7	4	10	-15.90	15.90
6	-4	-10	29.90	15.10
6	-4	-10	32.40	11.30
-6	-4	10	19.50	7.90
-6	-4	10	26.70	16.10
-6	4	10	25.10	17.40
-6	4	10	34.10	20.50
6	4	-10	18.30	9.00
-5	-4	10	3.70	15.00
5	-4	-10	36.50	12.50

-5	4	10	33.60	16.70
-5	4	10	51.09	18.20
5	4	-10	43.40	10.80
-4	-4	10	125.39	23.60
4	-4	-10	109.89	20.10
4	-4	-10	117.69	16.60
4	4	-10	96.09	13.30
-4	4	10	118.49	25.10
-4	4	10	68.99	19.10
-3	-4	10	172.88	26.60
3	-4	-10	157.88	22.50
3	-4	-10	147.29	18.50
-3	4	10	258.67	33.10
-3	4	10	206.98	31.80
3	4	-10	204.08	25.70
3	4	-10	189.18	15.50
2	-4	-10	21.30	8.10
-2	-4	10	23.60	5.70
-2	-4	10	11.90	5.50
-2	-4	10	29.10	13.90
2	-4	-10	17.70	9.50
2	4	-10	7.20	12.00
-2	4	10	9.80	12.90
-2	4	10	17.10	12.20
2	4	-10	20.60	7.00
-2	4	10	-2.30	6.50
1	-4	-10	88.09	16.20
-1	-4	10	113.89	20.50
1	-4	-10	82.99	13.20
-1	-4	10	80.49	6.60
-1	-4	10	79.19	6.70

1	4	-10	96.09	16.80
-1	4	10	45.80	16.90
1	4	-10	120.59	11.30
-1	4	10	85.59	12.80
-1	4	10	89.39	19.90
0	-4	-10	-4.30	9.70
0	-4	-10	-0.80	5.30
0	-4	-10	-0.60	8.30
0	-4	10	5.30	1.70
0	-4	10	2.00	1.80
0	-4	10	2.70	10.20
0	4	-10	1.00	4.90
0	4	-10	1.30	9.00
0	4	10	1.80	9.00
0	4	-10	5.60	5.80
0	4	10	3.30	6.20
0	4	10	19.90	14.00
-1	-4	-10	17.80	8.00
-1	-4	-10	15.90	8.90
-1	-4	-10	40.30	13.70
1	-4	10	42.50	13.00
-1	4	-10	37.20	8.30
1	4	10	33.00	8.60
1	4	10	33.20	15.90
-1	4	-10	38.90	11.80
1	4	10	33.90	13.10
2	-4	10	28.60	9.00
-2	-4	-10	17.50	8.50
-2	-4	-10	17.90	11.40
-2	-4	-10	14.50	12.90
-2	-4	-10	29.30	9.70

2	4	10	2.50	6.40
-2	4	-10	14.70	6.30
-2	4	-10	6.20	12.60
-2	4	-10	13.80	9.20
2	4	10	11.10	10.20
-2	4	-10	1.20	6.30
-3	-4	-10	180.38	19.70
-3	-4	-10	188.68	28.60
-3	-4	-10	167.38	19.10
-3	-4	-10	190.48	28.30
-3	4	-10	163.38	15.90
-3	4	-10	154.18	13.10
-3	4	-10	179.98	30.10
3	4	10	138.39	22.70
-3	4	-10	163.18	20.80
-3	4	-10	208.78	14.40
3	4	10	189.18	15.90
-4	-4	-10	108.49	15.80
-4	-4	-10	89.09	20.40
-4	-4	-10	82.99	12.80
-4	-4	-10	94.19	22.70
-4	4	-10	117.59	10.00
-4	4	-10	94.19	13.80
-4	4	-10	58.09	10.10
-4	4	-10	95.89	15.80
4	4	10	111.99	14.20
-4	4	-10	116.59	13.30
-4	4	-10	35.80	17.40
4	4	10	83.79	16.20
-5	-4	-10	44.20	12.40
-5	-4	-10	40.30	17.20

-5	-4	-10	57.69	11.30
-5	-4	-10	39.20	17.30
-5	4	-10	57.69	11.30
5	4	10	42.10	9.40
-5	4	-10	58.09	10.00
-5	4	-10	44.40	6.20
5	4	10	28.30	13.70
-5	4	-10	33.40	15.90
-5	4	-10	51.89	15.30
-5	4	-10	43.60	7.30
-6	-4	-10	17.90	8.80
-6	-4	-10	33.40	19.10
-6	-4	-10	0.10	15.10
-6	-4	-10	12.90	9.10
6	4	10	15.80	8.30
-6	4	-10	25.80	14.90
-6	4	-10	14.50	15.30
-6	4	-10	-4.50	7.80
-6	4	-10	12.30	7.30
-7	-4	-10	-13.00	17.20
-7	-4	-10	-1.30	17.50
-7	-4	-10	10.20	7.70
7	4	10	7.20	7.70
-7	4	-10	-2.10	9.90
-7	4	-10	-24.80	17.80
-8	-4	-10	4.90	18.20
-8	-4	-10	34.10	20.60
-8	-4	-10	10.30	9.00
-8	4	-10	7.90	11.90
-8	4	-10	46.20	24.20
8	4	10	2.80	8.60

-9	-4	-10	46.10	22.40
-9	-4	-10	31.60	27.20
-9	4	-10	13.80	24.10
-9	4	-10	7.10	15.00
9	4	10	-4.60	9.70
9	-5	-10	-5.30	14.50
9	-5	-10	17.00	18.70
-9	-5	10	16.50	23.60
9	5	-10	-4.70	12.10
-9	5	10	-28.20	21.00
-9	5	10	13.40	26.40
8	-5	-10	0.30	11.90
8	-5	-10	10.30	14.00
-8	-5	10	-9.50	20.60
-8	5	10	45.00	25.10
8	5	-10	8.30	9.20
-8	5	10	2.50	22.70
7	-5	-10	11.90	10.60
7	-5	-10	7.60	12.50
-7	-5	10	-12.70	17.40
-7	5	10	5.20	17.10
7	5	-10	2.30	9.50
-7	5	10	-3.00	19.60
6	-5	-10	8.10	10.30
-6	-5	10	3.50	17.10
6	-5	-10	14.20	12.60
-6	5	10	5.00	17.00
6	5	-10	-11.50	7.50
-6	5	10	-2.60	16.60
5	-5	-10	27.10	14.60
5	-5	-10	12.70	9.20

-5	-5	10	4.20	14.50
-5	5	10	28.40	16.40
5	5	-10	18.30	9.00
-5	5	10	26.10	17.10
4	-5	-10	-5.60	7.30
4	-5	-10	7.90	10.70
-4	-5	10	15.30	11.90
-4	5	10	4.80	14.80
4	5	-10	2.90	7.30
-4	5	10	2.50	15.50
-3	-5	10	-0.50	11.20
-3	-5	10	-4.10	5.00
-3	-5	10	0.10	4.90
3	-5	-10	-6.00	7.40
3	-5	-10	-2.50	5.80
3	5	-10	-1.90	5.40
-3	5	10	8.30	11.90
-3	5	10	6.90	12.20
-3	5	10	1.20	6.80
-2	-5	10	15.20	4.50
-2	-5	10	26.90	12.20
2	-5	-10	10.40	10.00
2	-5	-10	-2.60	5.80
-2	5	10	21.30	13.90
2	5	-10	16.40	6.50
2	5	-10	22.50	11.50
-2	5	10	11.20	12.90
-2	5	10	12.30	6.70
1	-5	-10	86.79	14.60
1	-5	-10	60.19	14.20
-1	-5	10	64.49	15.10

1	5	-10	65.69	13.50
-1	5	10	56.99	11.20
-1	5	10	57.49	19.90
1	5	-10	63.69	10.30
-1	5	10	63.59	17.60
0	-5	-10	115.09	22.10
0	-5	-10	109.29	14.70
0	-5	10	121.79	18.70
0	-5	-10	118.69	17.70
0	5	10	125.79	25.00
0	5	10	110.49	14.80
0	5	-10	111.89	11.10
0	5	-10	86.19	10.80
0	5	10	77.89	16.90
0	5	-10	114.99	16.30
-1	-5	-10	72.39	12.60
1	-5	10	70.09	13.80
-1	-5	-10	80.39	14.90
-1	-5	-10	94.69	20.50
-1	-5	-10	72.59	18.80
1	5	10	111.69	22.00
1	5	10	79.29	12.40
1	5	10	82.99	19.30
-1	5	-10	89.79	12.20
-1	5	-10	96.39	11.20
-1	5	-10	84.89	12.90
-2	-5	-10	27.60	14.50
-2	-5	-10	5.40	6.90
-2	-5	-10	18.70	8.60
-2	-5	-10	34.40	14.00
-2	5	-10	19.80	8.20

-2	5	-10	9.40	9.30
-2	5	-10	27.10	7.40
2	5	10	32.40	14.20
-2	5	-10	11.00	6.50
-2	5	-10	18.50	12.90
-3	-5	-10	3.90	6.60
-3	-5	-10	26.80	12.90
-3	-5	-10	-9.30	12.20
-3	-5	-10	4.10	7.00
-3	5	-10	7.40	6.90
-3	5	-10	-5.80	5.50
-3	5	-10	21.20	15.20
-3	5	-10	4.40	5.30
3	5	10	4.50	6.20
3	5	10	10.50	10.40
-3	5	-10	8.30	6.40
-4	-5	-10	-15.60	11.00
-4	-5	-10	-10.30	15.10
-4	-5	-10	1.60	7.80
-4	-5	-10	-5.70	8.60
4	5	10	-2.40	10.50
-4	5	-10	-15.60	7.40
-4	5	-10	-9.30	13.20
-4	5	-10	5.70	5.80
4	5	10	2.10	6.70
-4	5	-10	0.00	5.60
-5	-5	-10	12.70	8.50
-5	-5	-10	3.80	18.90
-5	-5	-10	4.70	16.00
-5	-5	-10	16.20	9.20
-5	5	-10	11.40	11.80

5	5	10	5.40	6.30
-5	5	-10	9.10	7.00
5	5	10	16.90	14.50
-5	5	-10	20.90	14.50
-5	5	-10	15.50	4.70
-5	5	-10	10.80	6.00
-6	-5	-10	-3.70	8.50
-6	-5	-10	10.90	16.60
-6	-5	-10	33.60	19.40
6	5	10	-1.30	14.20
-6	5	-10	30.20	8.40
-6	5	-10	4.80	15.40
-6	5	-10	4.40	8.00
6	5	10	-0.40	7.80
-6	5	-10	15.50	12.10
-7	-5	-10	8.10	9.60
-7	-5	-10	13.80	17.50
-7	-5	-10	-6.00	19.20
-7	5	-10	10.70	19.60
-7	5	-10	4.10	9.70
7	5	10	7.40	8.30
-8	-5	-10	33.00	10.20
-8	-5	-10	-17.50	23.20
-8	-5	-10	11.00	22.90
-8	5	-10	16.90	18.60
-8	5	-10	6.50	11.50
8	5	10	19.20	10.60
-8	-6	10	-17.50	23.10
8	-6	-10	-21.30	14.00
8	-6	-10	14.90	13.60
-8	6	10	41.00	24.90

8	6	-10	1.70	10.80
7	-6	-10	-14.50	12.90
7	-6	-10	0.80	11.00
-7	-6	10	6.70	21.70
-7	6	10	-14.10	18.30
7	6	-10	-1.20	8.50
-7	6	10	-37.70	18.00
-6	-6	10	1.10	18.30
6	-6	-10	-2.10	12.90
6	-6	-10	9.40	9.00
-6	6	10	-12.90	16.50
-6	6	10	-17.80	14.50
6	6	-10	5.20	8.30
5	-6	-10	14.00	15.00
5	-6	-10	19.40	10.40
-5	-6	10	14.10	16.70
-5	6	10	7.10	15.30
5	6	-10	27.10	9.70
-5	6	10	-3.70	15.30
4	-6	-10	-13.30	9.50
-4	-6	10	-1.50	13.60
4	-6	-10	15.30	8.70
-4	6	10	-6.80	13.60
4	6	-10	-0.60	15.60
-4	6	10	-4.90	14.30
4	6	-10	2.40	5.70
3	-6	-10	60.29	13.30
-3	-6	10	62.19	7.60
3	-6	-10	91.29	18.80
-3	-6	10	66.09	18.70
-3	-6	10	53.99	6.90

-3	6	10	54.59	21.00
-3	6	10	87.69	22.00
3	6	-10	62.19	10.00
-3	6	10	63.99	12.70
3	6	-10	56.19	16.90
2	-6	-10	57.19	11.60
-2	-6	10	74.09	17.90
2	-6	-10	70.49	16.40
-2	-6	10	52.59	4.30
2	6	-10	46.60	9.10
-2	6	10	68.09	21.00
2	6	-10	32.70	13.90
-2	6	10	67.89	18.70
-2	6	10	42.70	10.90
1	-6	-10	32.40	9.20
1	-6	-10	11.60	8.90
-1	-6	10	39.50	12.40
1	-6	-10	36.80	14.90
-1	6	10	17.50	8.70
-1	6	10	36.40	18.00
1	6	-10	22.10	6.10
-1	6	10	7.70	11.50
0	-6	-10	24.50	10.10
0	-6	10	12.30	10.00
0	-6	-10	8.00	11.00
0	-6	-10	14.00	7.40
0	6	10	18.90	13.20
0	6	-10	4.60	5.00
0	6	-10	6.60	7.60
0	6	-10	12.30	5.20
0	6	10	2.90	7.70

0	6	10	28.10	17.20
-1	-6	-10	31.30	14.10
-1	-6	-10	38.50	12.40
-1	-6	-10	34.70	9.60
1	-6	10	-2.70	7.10
-1	-6	-10	27.40	13.00
1	6	10	19.70	13.00
1	6	10	32.80	15.10
-1	6	-10	21.50	8.60
-1	6	-10	22.70	7.20
1	6	10	20.80	9.10
-1	6	-10	15.40	7.50
-1	6	-10	25.40	6.90
-2	-6	-10	44.60	16.30
-2	-6	-10	43.40	13.30
-2	-6	-10	27.30	15.20
-2	-6	-10	39.40	11.00
2	6	10	39.90	10.40
-2	6	-10	36.70	8.00
2	6	10	30.80	15.10
-2	6	-10	31.70	6.00
-2	6	-10	55.29	18.10
-2	6	-10	36.40	10.60
-3	-6	-10	96.79	17.80
-3	-6	-10	93.19	22.20
-3	-6	-10	107.29	22.80
-3	-6	-10	116.49	18.20
3	6	10	103.79	22.30
-3	6	-10	79.39	10.70
3	6	10	87.49	13.90
-3	6	-10	110.89	26.00

-3	6	-10	102.49	10.60
-4	-6	-10	10.80	8.50
-4	-6	-10	5.80	15.00
-4	-6	-10	-1.60	12.90
-4	-6	-10	2.40	8.40
4	6	10	16.40	7.80
-4	6	-10	20.10	6.80
4	6	10	30.70	16.70
-4	6	-10	36.30	18.70
-4	6	-10	15.30	7.20
-5	-6	-10	7.10	15.20
-5	-6	-10	17.10	9.20
-5	-6	-10	15.30	16.30
-5	-6	-10	-4.20	8.10
-5	6	-10	6.80	6.90
-5	6	-10	17.10	5.70
5	6	10	12.90	8.00
-5	6	-10	37.00	8.10
-5	6	-10	43.00	19.10
5	6	10	28.10	17.30
-6	-6	-10	-18.10	14.50
-6	-6	-10	-9.70	8.10
-6	-6	-10	24.50	17.00
6	6	10	-9.90	8.30
-6	6	-10	4.50	6.90
-6	6	-10	-4.60	15.40
6	6	10	-0.20	14.10
-7	-6	-10	-12.40	9.50
-7	-6	-10	-7.90	17.50
-7	-6	-10	-9.70	20.00
7	6	10	11.00	8.70

-7	6	-10	5.70	7.60
-7	6	-10	16.10	17.70
-8	-6	-10	5.30	10.60
-8	-6	-10	0.90	19.80
-8	-6	-10	16.60	23.00
-8	6	-10	15.90	21.70
-8	6	-10	-7.10	9.70
8	6	10	7.70	11.00
8	-7	-10	1.90	13.60
8	-7	-10	-5.00	14.30
-8	-7	10	-48.90	24.20
-8	7	10	14.90	21.80
-8	7	10	-13.80	23.30
-7	-7	10	0.70	20.60
7	-7	-10	33.50	15.90
7	-7	-10	20.80	13.40
7	7	-10	13.70	10.80
-7	7	10	5.10	20.20
-7	7	10	6.20	24.50
6	-7	-10	3.40	11.50
6	-7	-10	12.40	10.70
-6	-7	10	6.80	18.00
6	7	-10	14.10	9.90
-6	7	10	4.60	17.60
-6	7	10	28.10	19.00
-5	-7	10	40.50	19.30
5	-7	-10	49.40	15.50
5	-7	-10	39.90	12.30
-5	7	10	53.19	21.90
-5	7	10	20.10	19.50
4	-7	-10	4.20	9.00

4	-7	-10	3.00	10.90
-4	-7	10	2.20	16.20
-4	-7	10	1.30	7.80
-4	7	10	9.10	14.70
-4	7	10	5.00	16.40
4	7	-10	-2.80	6.50
4	7	-10	13.30	16.70
-3	-7	10	25.80	4.50
3	-7	-10	24.60	9.20
-3	-7	10	15.00	4.30
-3	-7	10	21.80	16.20
3	-7	-10	12.00	11.00
-3	7	10	23.70	18.20
-3	7	10	19.90	10.00
-3	7	10	18.50	16.90
3	7	-10	17.70	6.90
3	7	-10	26.30	13.10
-2	-7	10	9.80	12.90
2	-7	-10	-4.20	7.10
2	-7	-10	12.60	12.80
-2	7	10	-13.00	12.60
-2	7	10	3.90	9.10
-2	7	10	15.40	15.10
2	7	-10	0.00	9.90
2	7	-10	9.70	5.80
1	-7	-10	216.88	27.50
1	-7	-10	215.68	26.20
1	-7	-10	203.48	21.40
-1	-7	10	268.87	28.60
1	7	-10	218.08	14.30
-1	7	10	254.67	35.80

-1	7	10	147.19	18.60
-1	7	10	166.28	29.60
1	7	-10	205.28	20.60
0	-7	-10	23.00	10.60
0	-7	-10	25.30	8.80
0	-7	10	-15.30	8.40
0	7	-10	17.00	6.00
0	7	10	13.30	16.10
0	7	-10	3.30	6.10
0	7	10	30.40	10.10
0	7	-10	23.10	6.80
0	7	10	18.50	12.10
1	-7	10	143.59	16.10
-1	-7	-10	157.48	19.90
-1	-7	-10	127.29	20.00
-1	-7	-10	131.69	22.60
-1	-7	-10	145.29	29.40
-1	7	-10	147.59	14.20
-1	7	-10	130.89	16.00
-1	7	-10	118.79	8.80
-1	7	-10	102.19	21.70
-1	7	-10	118.79	10.60
1	7	10	109.69	16.10
-1	7	-10	109.69	8.60
1	7	10	107.89	21.80
-2	-7	-10	66.69	21.20
-2	-7	-10	35.50	15.60
-2	-7	-10	14.50	11.30
-2	7	-10	24.20	16.10
-2	7	-10	47.90	11.90
-2	7	-10	32.60	7.90

-2	7	-10	50.49	13.20
-3	-7	-10	3.00	8.10
-3	-7	-10	9.10	9.30
-3	-7	-10	3.80	13.20
3	7	10	-2.80	12.20
-3	7	-10	9.70	12.80
-3	7	-10	3.80	5.80
3	7	10	0.00	6.90
-3	7	-10	-12.60	5.10
-4	-7	-10	14.10	9.40
-4	-7	-10	-7.10	15.40
-4	-7	-10	2.40	14.30
-4	-7	-10	17.40	10.90
4	7	10	15.00	8.90
-4	7	-10	-19.90	7.70
4	7	10	10.40	12.80
-5	-7	-10	44.20	20.90
-5	-7	-10	31.00	12.70
-5	-7	-10	36.50	13.60
-5	-7	-10	68.69	19.50
5	7	10	60.69	12.50
-5	7	-10	45.80	8.30
5	7	10	38.30	18.40
-5	7	-10	51.49	21.00
-6	-7	-10	-40.70	16.00
-6	-7	-10	-14.40	10.10
-6	-7	-10	-10.60	18.80
6	7	10	28.20	19.00
-6	7	-10	20.90	17.00
6	7	10	16.70	10.00
-7	-7	-10	14.50	20.70

-7	-7	-10	-10.50	11.00
-7	-7	-10	-12.20	21.30
7	7	10	17.40	10.90
-7	7	-10	0.20	17.20
7	7	10	15.40	20.40
-8	-7	-10	-18.20	20.10
-8	-7	-10	-12.30	12.10
-8	-7	-10	-5.90	22.00
8	7	10	1.20	11.10
7	-8	-10	6.40	12.20
7	-8	-10	-4.60	14.70
-7	-8	10	-35.90	22.50
7	8	-10	20.20	11.20
-7	8	10	-0.50	23.30
-6	-8	10	0.40	19.00
6	-8	-10	-5.00	9.90
6	-8	-10	-17.10	12.90
-6	8	10	-15.20	17.30
6	8	-10	-11.20	8.60
-6	8	10	26.00	22.10
-5	-8	10	11.10	17.90
5	-8	-10	39.90	16.90
5	-8	-10	22.30	10.00
-5	8	10	-6.70	18.80
-5	8	10	7.70	17.30
5	8	-10	29.00	10.20
4	-8	-10	22.30	12.20
-4	-8	10	34.20	8.00
-4	-8	10	6.70	18.30
4	-8	-10	31.80	11.10
-4	8	10	27.20	12.30

-4	8	10	32.50	20.80
-4	8	10	19.20	17.50
4	8	-10	27.50	9.70
3	-8	-10	57.59	15.10
-3	-8	10	66.89	17.90
3	-8	-10	89.99	16.80
-3	8	10	66.09	23.20
-3	8	10	36.70	18.40
3	8	-10	69.29	10.60
-3	8	10	82.09	15.70
3	8	-10	46.60	16.00
2	-8	-10	18.00	12.40
2	-8	-10	-3.40	9.40
2	-8	-10	4.30	9.40
-2	-8	10	10.80	13.60
-2	8	10	-1.60	13.10
-2	8	10	8.60	9.40
2	8	-10	-9.40	11.20
-2	8	10	8.30	15.00
2	8	-10	-8.80	5.50
1	-8	-10	27.50	10.80
1	-8	-10	22.10	12.90
-1	-8	10	17.60	12.60
1	-8	-10	80.79	17.20
-1	8	10	28.30	17.20
-1	8	10	24.50	18.70
1	8	-10	26.60	8.00
1	8	-10	6.30	8.70
-1	8	10	25.50	11.00
0	-8	10	-5.60	13.20
0	-8	-10	19.30	12.50

0	-8	-10	22.40	13.50
0	8	-10	2.80	4.90
0	8	10	1.30	14.10
0	8	10	11.60	14.40
0	8	10	-8.30	7.40
0	8	-10	3.30	4.90
1	-8	10	25.70	9.60
-1	-8	-10	24.10	10.80
-1	-8	-10	33.80	14.00
-1	-8	-10	12.30	20.00
-1	-8	-10	40.70	15.50
1	8	10	14.80	10.10
-1	8	-10	10.50	8.60
1	8	10	0.80	11.70
-1	8	-10	33.60	10.90
-1	8	-10	15.80	15.80
-2	-8	-10	-0.60	11.70
-2	-8	-10	-5.30	7.00
-2	-8	-10	7.50	10.30
-2	-8	-10	0.20	16.90
-2	8	-10	10.10	5.90
2	8	10	12.70	9.50
2	8	10	-17.30	11.30
-2	8	-10	20.40	12.10
-2	8	-10	2.90	11.70
-3	-8	-10	81.69	16.70
-3	-8	-10	78.49	20.30
-3	-8	-10	50.39	12.20
-3	-8	-10	90.99	23.20
3	8	10	80.19	21.80
-3	8	-10	67.09	9.60

3	8	10	79.59	15.00
-3	8	-10	61.19	19.00
-3	8	-10	57.59	20.00
-4	-8	-10	32.00	22.10
-4	-8	-10	39.60	15.20
-4	-8	-10	66.79	19.50
-4	-8	-10	39.80	13.00
-4	8	-10	73.59	17.90
-4	8	-10	24.60	18.90
4	8	10	45.50	11.40
4	8	10	31.20	18.20
-4	8	-10	22.60	6.80
-5	-8	-10	26.60	16.90
-5	-8	-10	51.29	21.00
5	8	10	36.50	11.30
-5	8	-10	46.40	19.60
5	8	10	2.00	15.00
-5	8	-10	-6.60	17.00
-5	8	-10	16.60	6.10
-6	-8	-10	-1.70	21.60
-6	-8	-10	-15.80	11.00
-6	-8	-10	37.50	19.30
6	8	10	-3.50	9.40
-6	8	-10	23.70	20.50
-6	8	-10	3.80	18.00
6	8	10	31.00	22.20
-7	-8	-10	-35.30	20.10
-7	-8	-10	-7.20	12.30
-7	8	-10	-2.30	21.90
-7	8	-10	14.00	19.80
7	8	10	3.10	20.70

7	−9	−10	9.10	12.30
−7	−9	10	−25.80	25.80
7	−9	−10	−24.00	25.90
−7	9	10	−11.80	21.00
7	9	−10	−9.30	10.30
−7	9	10	49.50	28.00
6	−9	−10	3.60	16.00
−6	−9	10	−12.60	22.30
6	−9	−10	6.20	11.80
6	9	−10	−3.90	9.70
−6	9	10	11.70	24.30
−6	9	10	11.00	21.10
5	−9	−10	15.40	10.70
−5	−9	10	−8.40	19.00
5	−9	−10	13.60	13.00
−5	9	10	−22.50	17.30
5	9	−10	6.90	9.30
−5	9	10	−8.20	18.70
4	−9	−10	20.90	13.30
4	−9	−10	4.90	10.00
−4	−9	10	10.20	18.20
−4	9	10	30.60	11.30
4	9	−10	15.20	8.50
−4	9	10	42.40	17.20
−4	9	10	21.70	18.40
−3	−9	10	−14.10	15.00
3	−9	−10	6.50	8.50
3	−9	−10	−7.80	12.60
3	−9	−10	4.80	13.60
−3	9	10	9.60	17.40
−3	9	10	−19.20	8.40

3	9	-10	-15.00	5.80
-3	9	10	-27.30	15.40
2	-9	-10	7.10	12.00
2	-9	-10	-13.90	10.20
2	-9	-10	-3.00	7.30
-2	-9	10	7.70	16.80
-2	9	10	2.30	16.20
2	9	-10	-3.10	10.20
2	9	-10	-1.70	5.90
-2	9	10	29.50	17.20
-2	9	10	6.90	9.60
-1	-9	10	11.40	13.60
1	-9	-10	-13.30	6.50
1	-9	-10	28.50	13.70
1	-9	-10	19.70	12.70
1	-9	-10	14.20	11.30
1	9	-10	-1.60	7.30
-1	9	10	-7.20	6.60
-1	9	10	7.50	14.40
-1	9	10	19.60	16.90
0	-9	-10	59.69	15.60
0	-9	-10	53.29	21.40
0	-9	-10	66.79	17.10
0	-9	-10	30.10	12.30
0	-9	-10	53.19	13.50
0	-9	10	22.20	21.40
0	9	-10	37.00	7.40
0	9	10	44.70	13.20
0	9	10	90.89	22.70
-1	-9	-10	0.00	12.10
-1	-9	-10	-10.80	9.50

-1	-9	-10	25.50	18.10
-1	-9	-10	-1.70	7.90
1	9	10	7.30	17.30
1	9	10	-6.30	9.40
-1	9	-10	2.90	8.30
-1	9	-10	-23.90	11.90
-1	9	-10	-15.90	10.20
-2	-9	-10	-1.90	15.40
-2	-9	-10	-12.10	13.60
-2	-9	-10	2.70	10.60
-2	-9	-10	8.60	9.80
-2	9	-10	10.90	5.60
-2	9	-10	27.10	13.70
2	9	10	-3.80	16.80
2	9	10	4.60	9.90
-2	9	-10	-7.30	13.60
-3	-9	-10	-11.50	16.40
-3	-9	-10	19.40	15.00
-3	-9	-10	13.70	9.00
-3	-9	-10	6.70	10.30
-3	9	-10	-28.70	12.70
3	9	10	-1.10	17.60
-3	9	-10	-2.80	5.70
3	9	10	3.10	9.40
-4	-9	-10	8.70	17.10
-4	-9	-10	1.40	11.50
-4	-9	-10	6.90	11.50
-4	-9	-10	-4.00	20.90
-4	9	-10	17.30	5.50
-4	9	-10	-9.20	15.30
4	9	10	-3.40	8.60

4	9	10	-8.10	17.10
-4	9	-10	9.40	15.10
-5	-9	-10	14.20	13.20
-5	-9	-10	-1.00	20.90
-5	-9	-10	7.60	17.00
-5	9	-10	-12.00	18.30
5	9	10	0.00	17.10
-5	9	-10	-34.00	16.30
5	9	10	4.30	10.50
-6	-9	-10	-7.10	22.80
-6	-9	-10	3.90	24.20
-6	-9	-10	-8.80	12.50
-6	9	-10	-6.80	17.90
-6	9	-10	-38.80	18.90
-7	-9	-10	13.70	27.90
-7	-9	-10	-16.30	24.60
-7	-9	-10	-19.30	14.30
-7	9	-10	-8.50	23.50
-7	9	-10	22.10	22.00
7	9	10	-27.10	18.70
7	9	10	-7.40	11.50
6	-10	-10	-13.10	10.80
-6	-10	10	-11.90	26.80
6	10	-10	14.60	11.80
-6	10	10	32.30	20.70
-6	10	10	33.40	29.80
-5	-10	10	-9.60	24.50
5	-10	-10	16.70	11.00
5	-10	-10	10.50	17.60
-5	10	10	8.70	19.10
5	10	-10	6.50	9.80

-5	10	10	17.10	12.80
-5	10	10	-0.60	22.10
4	-10	-10	-3.00	9.60
4	-10	-10	2.30	13.40
-4	-10	10	-11.30	20.00
4	10	-10	17.10	8.40
-4	10	10	-3.10	17.90
-4	10	10	0.70	19.90
-4	10	10	8.10	10.80
3	-10	-10	-11.60	12.50
-3	-10	10	-19.90	17.10
3	-10	-10	12.30	16.80
3	-10	-10	-11.20	9.00
3	10	-10	-3.50	7.20
-3	10	10	31.40	23.30
-3	10	10	-13.90	18.00
-3	10	10	13.00	9.90
2	-10	-10	-10.20	8.10
2	-10	-10	-5.60	11.70
-2	10	10	-3.00	17.20
-2	10	10	1.00	9.40
2	10	-10	-8.10	6.00
-2	10	10	9.10	17.20
1	-10	-10	5.10	9.10
1	-10	-10	-0.70	14.00
-1	10	10	-24.10	16.50
1	10	-10	9.70	6.60
-1	10	10	-2.30	10.00
1	10	-10	-8.80	5.90
-1	10	10	15.80	17.90
0	-10	-10	9.60	11.10

0	-10	-10	57.79	14.30
0	-10	-10	-10.90	22.40
0	-10	-10	28.20	10.80
0	10	10	33.10	17.90
0	10	10	32.00	11.50
0	10	-10	16.30	6.70
-1	-10	-10	-3.70	12.10
-1	-10	-10	1.50	9.40
-1	-10	-10	9.70	21.20
-1	-10	-10	18.80	11.30
-1	10	-10	-7.50	14.60
1	10	10	-11.90	8.40
1	10	10	9.10	18.00
-1	10	-10	0.40	11.70
-2	-10	-10	17.40	14.00
-2	-10	-10	25.10	22.00
-2	-10	-10	16.60	10.20
-2	-10	-10	25.50	24.60
2	10	10	-3.50	16.50
-2	10	-10	13.90	6.00
2	10	10	8.70	10.90
-2	10	-10	37.30	16.50
-3	-10	-10	12.50	12.50
-3	-10	-10	13.10	21.20
-3	-10	-10	-7.10	9.90
-3	-10	-10	-15.60	14.50
-3	10	-10	24.70	16.40
3	10	10	0.00	21.50
3	10	10	-0.60	9.40
-3	10	-10	-11.60	17.40
-4	-10	-10	-5.60	11.90

-4	-10	-10	4.40	17.10
-4	-10	-10	23.00	23.50
4	10	10	1.60	11.50
4	10	10	12.90	17.70
-4	10	-10	9.80	17.10
-4	10	-10	12.10	18.80
-5	-10	-10	13.80	19.50
-5	-10	-10	-27.40	20.90
-5	-10	-10	7.10	14.10
-5	10	-10	20.30	18.60
-5	10	-10	-7.50	20.40
5	10	10	8.70	22.00
-6	-10	-10	8.10	23.60
-6	-10	-10	-14.30	49.40
-6	-10	-10	10.20	14.90
6	10	10	20.50	13.30
6	10	10	-1.00	22.60
-6	10	-10	23.20	24.10
-6	10	-10	-42.90	18.20
5	-11	-10	-8.90	14.70
5	-11	-10	-8.50	11.50
5	11	-10	-12.80	10.40
-5	11	10	-34.50	18.30
-5	11	10	12.60	19.80
-5	11	10	24.50	25.90
4	-11	-10	18.20	14.90
4	-11	-10	-6.40	10.20
-4	11	10	-1.60	20.80
-4	11	10	-6.00	12.20
4	11	-10	-12.80	9.10
-4	11	10	28.70	28.30

3	-11	-10	9.20	13.90
3	-11	-10	12.00	10.80
-3	11	10	6.80	21.50
-3	11	10	21.50	11.20
-3	11	10	-5.50	19.40
3	11	-10	-4.10	8.10
2	-11	-10	1.60	10.80
2	-11	-10	-18.60	11.70
-2	11	10	12.90	11.60
-2	11	10	11.70	21.10
2	11	-10	12.00	8.30
-2	11	10	20.60	21.50
1	-11	-10	33.40	23.50
1	-11	-10	29.60	11.50
1	-11	-10	-12.20	11.80
-1	11	10	7.30	18.80
-1	11	10	9.00	12.90
1	11	-10	7.60	6.70
0	-11	-10	-6.00	12.20
0	-11	-10	4.80	9.90
0	-11	-10	-21.40	20.40
0	11	-10	12.20	17.00
0	11	10	3.80	18.90
0	11	-10	-15.10	5.90
0	11	10	12.70	12.90
-1	-11	-10	-6.40	8.80
-1	-11	-10	-18.30	11.00
-1	-11	-10	42.40	25.60
-1	11	-10	-31.00	12.40
1	11	10	25.70	20.50
1	11	10	1.20	10.20

-1	11	-10	61.69	20.30
-2	-11	-10	-6.60	21.80
-2	-11	-10	34.30	13.00
-2	-11	-10	13.60	12.90
-2	-11	-10	-13.30	15.50
2	11	10	14.40	18.40
-2	11	-10	43.70	27.20
2	11	10	7.30	10.50
-2	11	-10	45.40	21.80
-3	-11	-10	-3.00	23.40
-3	-11	-10	-35.30	16.40
-3	-11	-10	-18.30	12.80
-3	11	-10	-0.10	19.30
-3	11	-10	13.90	18.90
3	11	10	2.70	19.50
3	11	10	-2.10	12.30
-4	-11	-10	23.20	19.40
-4	-11	-10	7.30	24.80
-4	-11	-10	18.60	14.30
4	11	10	1.00	14.50
-4	11	-10	-34.70	20.00
-4	11	-10	-2.20	19.60
-5	-11	-10	-8.80	13.10
-5	-11	-10	11.80	23.70
-5	-11	-10	9.40	30.70
5	11	10	30.10	26.50
5	11	10	-4.40	12.20
-5	11	-10	22.10	19.80
-5	11	-10	35.00	24.50
4	-12	-10	-13.80	11.70
4	-12	-10	-2.50	15.50

-4	12	10	-10.40	25.20
-4	12	10	-12.70	22.00
-4	12	10	-10.80	13.50
4	12	-10	0.60	10.40
3	-12	-10	7.20	11.20
3	-12	-10	-16.30	14.30
-3	12	10	4.10	13.00
-3	12	10	-26.20	21.00
-3	12	10	11.80	24.20
3	12	-10	-10.30	9.00
2	-12	-10	-4.40	10.60
2	-12	-10	-14.30	29.80
2	12	-10	-16.70	8.00
-2	12	10	13.00	13.90
1	-12	-10	10.20	27.00
1	-12	-10	7.20	17.40
1	-12	-10	13.00	11.00
-1	12	10	1.70	11.20
1	12	-10	-0.30	7.40
-1	12	10	-16.40	17.70
0	-12	-10	-14.30	25.40
0	-12	-10	-3.20	11.30
0	-12	-10	-23.60	11.80
0	12	10	12.10	14.50
0	12	10	11.40	22.70
0	12	-10	-37.10	17.00
-1	-12	-10	-10.40	16.20
-1	-12	-10	13.50	26.10
-1	-12	-10	11.30	11.80
1	12	10	3.00	11.90
1	12	10	-4.00	22.40

-1	12	-10	-9.50	18.80
-2	-12	-10	10.90	15.60
-2	-12	-10	21.50	29.50
-2	12	-10	18.60	18.40
-3	-12	-10	-49.50	27.00
-3	-12	-10	-6.10	15.50
-3	12	-10	32.30	23.60
-3	12	-10	-10.20	19.70
3	12	10	3.80	23.90
3	12	10	29.90	13.80
-4	-12	-10	-29.80	28.00
-4	12	-10	5.70	23.40
2	-13	-10	-30.90	31.10
-2	13	10	-46.10	22.90
1	-13	-10	11.90	28.60
1	-13	-10	18.90	13.60
1	-13	-10	-23.60	16.90
-1	13	10	21.20	23.90
-1	13	10	8.60	14.70
0	-13	-10	5.80	18.10
0	-13	-10	50.59	29.50
0	13	-10	-0.20	22.00
0	13	10	-23.30	21.60
0	13	10	7.50	14.20
-1	-13	-10	20.00	16.70
-1	-13	-10	0.70	28.90
1	13	10	6.00	23.70
-1	13	-10	5.60	20.90
1	13	10	4.40	15.00
9	0	-11	-18.50	16.40
-9	0	11	-9.20	22.10

-9	0	11	11.50	22.20
9	0	-11	-1.00	12.90
-8	0	11	11.20	19.60
8	0	-11	-5.60	9.10
-8	0	11	9.00	17.70
8	0	-11	-17.70	12.10
-7	0	11	96.19	26.50
7	0	-11	45.90	14.20
-7	0	11	62.19	20.10
7	0	-11	94.59	20.10
6	0	-11	-11.90	8.50
-6	0	11	-0.20	12.50
-6	0	11	-3.00	14.90
6	0	-11	12.60	11.10
-5	0	11	20.40	13.60
5	0	-11	-7.90	12.20
5	0	-11	3.20	10.10
4	0	-11	3.30	7.60
-4	0	11	-5.20	8.30
-4	0	11	11.80	13.20
4	0	-11	-4.80	8.00
-3	0	11	491.65	39.50
3	0	-11	601.74	29.20
3	0	-11	574.14	34.70
-3	0	11	543.45	31.70
3	0	-11	467.35	34.00
-2	0	11	3.70	6.40
2	0	-11	7.50	7.10
-2	0	11	-7.60	7.50
-2	0	11	7.70	11.00
2	0	-11	0.10	6.10

2	0	-11	-2.30	12.30
-1	0	11	14.90	12.70
-1	0	11	37.00	8.30
1	0	-11	40.00	10.90
1	0	-11	53.09	10.80
-1	0	11	19.20	9.20
0	0	11	-3.40	11.60
0	0	11	5.80	5.20
0	0	-11	-15.50	13.90
0	0	-11	15.20	7.90
0	0	-11	-2.80	7.40
0	0	11	3.60	7.60
-1	0	-11	19.60	7.90
1	0	11	23.60	10.20
-1	0	-11	38.50	17.90
-1	0	-11	45.80	11.80
1	0	11	38.40	7.40
1	0	11	43.10	16.70
-1	0	-11	70.39	20.10
2	0	11	22.80	9.90
-2	0	-11	1.00	12.70
2	0	11	1.60	4.80
-2	0	-11	-7.60	6.30
-2	0	-11	-4.80	8.80
2	0	11	5.30	9.50
-2	0	-11	1.40	5.80
-2	0	-11	-8.10	14.50
-3	0	-11	20.90	17.10
3	0	11	9.90	4.60
3	0	11	10.90	10.00
-3	0	-11	2.00	7.20

-3	0	-11	12.10	13.00
-3	0	-11	7.00	10.60
-3	0	-11	7.60	8.00
-4	0	-11	12.80	14.20
-4	0	-11	5.80	7.10
-4	0	-11	14.00	16.60
4	0	11	-3.60	4.60
-4	0	-11	4.50	6.50
-4	0	-11	3.80	9.90
-5	0	-11	23.60	17.10
-5	0	-11	55.29	20.40
5	0	11	18.50	6.10
-5	0	-11	26.30	16.50
-5	0	-11	22.80	9.40
6	0	11	4.70	5.80
-6	0	-11	-9.80	16.00
-6	0	-11	-2.90	7.70
-6	0	-11	2.10	12.50
-7	0	-11	8.90	14.70
-7	0	-11	4.20	9.10
7	0	11	8.70	7.20
-7	0	-11	3.80	16.20
-8	0	-11	-33.30	18.20
-8	0	-11	-8.20	15.60
8	0	11	-10.30	7.90
-9	0	-11	-28.30	18.40
-9	0	-11	-26.70	19.40
-9	-1	11	11.00	22.90
9	-1	-11	17.40	16.20
-9	-1	11	44.70	25.00
9	-1	-11	16.00	14.50

9	1	-11	6.80	12.40
9	1	-11	-17.00	13.40
-9	1	11	42.60	24.80
-8	-1	11	2.30	20.50
8	-1	-11	0.70	14.20
-8	-1	11	-15.90	16.30
8	-1	-11	-10.60	11.30
-8	1	11	9.90	21.80
-8	1	11	23.00	19.50
8	1	-11	-9.80	9.60
-7	-1	11	83.59	23.90
7	-1	-11	76.49	19.00
7	-1	-11	103.49	17.40
-7	-1	11	73.99	18.60
7	1	-11	76.69	15.40
-7	1	11	38.50	21.20
-7	1	11	66.09	20.90
-6	-1	11	-3.40	15.40
6	-1	-11	-2.40	9.40
6	-1	-11	17.50	11.20
-6	-1	11	-17.80	11.50
-6	1	11	14.90	16.40
-6	1	11	5.60	13.50
6	1	-11	8.70	9.30
5	-1	-11	5.60	8.00
-5	-1	11	0.70	14.20
-5	-1	11	4.90	10.00
5	-1	-11	10.50	11.20
-5	1	11	8.40	12.60
-5	1	11	12.80	13.40
-4	-1	11	17.30	9.00

4	-1	-11	5.00	9.00
4	-1	-11	3.40	7.70
-4	-1	11	-4.40	12.60
4	1	-11	9.90	7.80
-4	1	11	5.50	11.50
-4	1	11	17.80	13.80
4	1	-11	3.40	11.40
-3	-1	11	-6.50	11.80
3	-1	-11	-0.50	9.10
-3	-1	11	12.50	8.40
3	-1	-11	13.60	8.60
-3	1	11	0.90	10.30
3	1	-11	4.30	10.60
3	1	-11	13.00	8.10
-3	1	11	17.80	13.00
2	-1	-11	3.10	7.50
-2	-1	11	0.60	5.30
2	-1	-11	12.00	7.40
-2	-1	11	10.20	7.70
2	1	-11	12.60	7.80
2	1	-11	16.50	11.70
-2	1	11	-3.20	11.90
-2	1	11	24.90	11.80
-2	1	11	2.60	5.60
1	-1	-11	0.40	9.00
-1	-1	11	3.90	13.20
-1	-1	11	3.10	5.00
1	-1	-11	5.40	7.50
-1	-1	11	-7.10	6.00
1	1	-11	-9.60	13.30
-1	1	11	7.10	6.30

1	1	-11	-4.20	5.30
1	1	-11	-6.30	9.60
-1	1	11	0.40	11.80
-1	1	11	5.10	8.50
0	-1	11	6.20	11.80
0	-1	11	2.10	6.10
0	-1	11	8.40	7.50
0	-1	-11	2.90	7.10
0	-1	-11	5.50	6.90
0	1	11	-1.30	5.70
0	1	-11	0.40	6.40
0	1	11	15.50	8.70
0	1	-11	22.00	10.00
0	1	-11	-12.20	12.00
0	1	-11	16.40	7.90
0	1	11	-2.70	10.70
-1	-1	-11	415.16	27.40
-1	-1	-11	361.46	34.80
1	-1	11	430.46	18.10
1	-1	11	416.36	27.00
1	-1	11	416.56	18.50
1	-1	11	497.45	36.10
-1	-1	-11	406.76	23.60
1	1	11	426.46	36.90
1	1	11	388.76	19.70
-1	1	-11	385.06	37.80
-1	1	-11	397.46	22.90
1	1	11	391.56	28.80
-1	1	-11	415.76	26.40
-1	1	-11	418.36	35.80
2	-1	11	0.30	4.30

-2	-1	-11	11.50	12.60
-2	-1	-11	-2.40	9.90
2	-1	11	5.40	5.40
2	-1	11	-5.00	10.20
-2	-1	-11	-2.70	7.10
-2	-1	-11	3.20	6.50
-2	1	-11	2.00	13.40
-2	1	-11	-4.10	8.20
2	1	11	19.40	12.70
-2	1	-11	1.90	6.20
-2	1	-11	22.40	13.30
2	1	11	4.50	5.10
2	1	11	1.20	8.20
-3	-1	-11	14.30	8.00
-3	-1	-11	28.10	12.50
-3	-1	-11	-0.10	7.80
3	-1	11	10.40	4.70
-3	-1	-11	12.10	13.00
-3	1	-11	-5.00	14.80
-3	1	-11	8.50	6.90
-3	1	-11	9.00	13.60
-3	1	-11	18.90	8.60
3	1	11	17.90	5.70
3	1	11	16.30	10.10
-3	1	-11	-2.90	9.00
4	-1	11	3.90	4.00
-4	-1	-11	-2.20	13.30
-4	-1	-11	-8.30	7.20
4	-1	11	-1.60	6.10
-4	-1	-11	-9.70	9.90
-4	-1	-11	-3.20	6.90

-4	1	-11	2.00	6.70
-4	1	-11	-3.50	12.30
-4	1	-11	5.90	9.10
-4	1	-11	-6.10	6.40
-4	1	-11	27.30	16.40
4	1	11	1.00	5.40
5	-1	11	141.79	10.00
-5	-1	-11	144.19	27.90
-5	-1	-11	145.99	13.20
-5	-1	-11	120.79	21.50
-5	-1	-11	123.69	17.10
5	-1	11	171.78	15.20
5	1	11	142.89	12.50
-5	1	-11	113.59	28.00
-5	1	-11	126.29	15.80
-5	1	-11	109.09	11.40
-5	1	-11	142.29	21.00
-6	-1	-11	24.30	18.00
6	-1	11	-4.00	4.90
-6	-1	-11	-5.60	13.10
-6	-1	-11	-5.70	7.90
-6	-1	-11	10.80	5.70
6	1	11	-14.90	5.50
-6	1	-11	-21.20	12.40
-6	1	-11	11.20	10.80
-6	1	-11	9.80	7.70
-6	1	-11	24.50	19.90
7	-1	11	4.70	6.10
-7	-1	-11	4.20	9.80
-7	-1	-11	41.30	18.60
-7	-1	-11	15.90	17.50

-7	1	-11	-2.00	8.40
-7	1	-11	-6.10	12.40
7	1	11	-8.60	6.60
-7	1	-11	-10.40	16.70
-8	-1	-11	18.70	23.40
-8	-1	-11	-15.70	15.90
-8	1	-11	19.70	20.10
8	1	11	-7.70	7.80
-8	1	-11	-6.20	14.00
-9	-1	-11	11.90	19.70
-9	-1	-11	-43.30	23.50
9	1	11	-2.60	17.00
-9	1	-11	27.60	25.60
9	-2	-11	9.90	18.50
9	-2	-11	-5.50	13.20
-9	-2	11	11.10	34.20
-9	-2	11	-11.50	21.50
-9	2	11	40.90	25.70
-9	2	11	27.30	25.80
9	2	-11	6.50	13.10
8	-2	-11	-3.70	11.60
8	-2	-11	-14.00	15.90
-8	-2	11	-7.20	20.10
-8	-2	11	-39.00	17.40
-8	2	11	45.90	24.30
-8	2	11	24.80	19.80
8	2	-11	-13.00	9.20
-7	-2	11	18.80	17.40
7	-2	-11	7.50	10.10
7	-2	-11	-7.60	14.60
-7	-2	11	28.20	14.10

-7	2	11	30.80	18.40
-7	2	11	-8.40	15.90
7	2	-11	1.60	9.10
-6	-2	11	5.40	17.60
6	-2	-11	-5.10	9.60
-6	-2	11	4.00	10.90
6	-2	-11	2.20	10.50
-6	2	11	6.10	16.00
6	2	-11	3.80	8.50
-6	2	11	16.30	15.10
-5	-2	11	42.60	18.30
5	-2	-11	49.10	14.60
5	-2	-11	31.70	11.10
-5	2	11	52.49	19.20
-5	2	11	59.09	19.00
-4	-2	11	3.80	6.90
4	-2	-11	-5.60	8.30
-4	-2	11	1.30	13.30
4	-2	-11	-0.90	7.90
-4	2	11	7.90	12.00
-4	2	11	1.20	13.10
4	2	-11	2.80	8.20
3	-2	-11	432.56	32.60
-3	-2	11	377.66	21.30
-3	-2	11	537.05	38.60
3	-2	-11	405.86	25.40
-3	-2	11	405.46	21.30
-3	2	11	445.26	25.20
3	2	-11	364.16	24.20
-3	2	11	489.25	33.30
-3	2	11	467.95	40.00

3	2	-11	452.45	25.30
2	-2	-11	10.50	10.20
-2	-2	11	2.40	5.10
-2	-2	11	1.70	5.60
2	-2	-11	7.10	7.20
-2	-2	11	13.80	10.70
-2	2	11	19.70	7.50
2	2	-11	3.20	14.20
2	2	-11	-1.60	6.20
-2	2	11	9.90	13.20
-2	2	11	6.30	10.40
2	2	-11	-13.90	9.10
-1	-2	11	20.70	6.80
1	-2	-11	27.30	10.60
-1	-2	11	37.20	8.20
-1	-2	11	31.00	14.10
1	-2	-11	28.50	9.80
-1	2	11	41.30	17.80
-1	2	11	26.60	7.60
-1	2	11	30.90	11.80
1	2	-11	4.30	11.00
1	2	-11	47.90	9.70
1	2	-11	6.50	14.00
0	-2	11	28.70	8.60
0	-2	11	9.40	5.70
0	-2	-11	19.40	8.50
0	-2	-11	11.30	8.20
0	-2	11	19.60	5.20
0	-2	11	16.20	14.10
0	2	-11	16.10	7.70
0	2	11	8.40	12.50

0	2	-11	20.20	9.50
0	2	11	0.30	8.90
0	2	-11	-4.50	12.20
0	2	11	37.80	8.50
-1	-2	-11	245.78	18.30
-1	-2	-11	229.78	20.70
1	-2	11	236.08	12.80
1	-2	11	232.88	11.80
-1	-2	-11	272.67	30.00
1	2	11	262.67	17.10
-1	2	-11	246.28	20.40
1	2	11	246.18	23.50
-1	2	-11	234.48	28.10
-1	2	-11	247.88	35.30
-1	2	-11	229.68	17.60
1	2	11	237.18	32.30
-2	-2	-11	3.10	8.00
-2	-2	-11	7.20	7.70
-2	-2	-11	7.10	11.90
-2	-2	-11	12.50	10.90
2	-2	11	12.50	10.70
2	-2	11	11.30	4.70
2	2	11	-1.80	7.90
-2	2	-11	6.50	8.70
-2	2	-11	-2.10	12.40
-2	2	-11	1.00	7.90
2	2	11	-3.30	10.60
-2	2	-11	-3.80	6.40
-2	2	-11	14.10	12.70
2	2	11	2.40	4.90
-3	-2	-11	-3.40	6.10

-3	-2	-11	-1.30	10.20
3	-2	11	2.90	4.20
-3	-2	-11	-1.70	7.60
-3	-2	-11	7.20	13.50
3	-2	11	0.20	2.80
-3	2	-11	-16.80	13.00
3	2	11	6.90	9.80
3	2	11	5.20	5.60
-3	2	-11	-1.60	7.10
-3	2	-11	-6.30	12.80
-3	2	-11	-5.30	7.00
-3	2	-11	1.40	8.20
4	-2	11	0.20	4.00
-4	-2	-11	24.30	17.50
-4	-2	-11	-6.10	6.60
-4	-2	-11	1.10	11.90
4	-2	11	-1.30	3.00
4	2	11	1.50	5.80
-4	2	-11	-3.70	5.70
-4	2	-11	-1.10	9.10
-4	2	-11	-10.60	6.50
-4	2	-11	11.50	13.10
-4	2	-11	4.70	15.30
4	2	11	-4.90	9.60
-5	-2	-11	0.70	15.50
-5	-2	-11	12.00	8.80
-5	-2	-11	21.90	13.80
5	-2	11	2.20	4.50
-5	-2	-11	-2.40	6.70
-5	2	-11	-6.90	6.60
-5	2	-11	3.10	5.20

-5	2	-11	53.59	17.20
5	2	11	9.90	6.40
-5	2	-11	35.10	18.50
-5	2	-11	-2.00	9.60
-5	2	-11	-2.30	4.70
-6	-2	-11	1.00	12.90
-6	-2	-11	6.70	8.90
-6	-2	-11	24.40	7.50
-6	-2	-11	22.80	16.30
6	2	11	9.90	6.70
-6	2	-11	4.60	8.10
-6	2	-11	-15.30	16.90
-6	2	-11	22.90	13.20
-7	-2	-11	6.80	15.90
-7	-2	-11	15.50	6.10
-7	-2	-11	-1.40	16.70
7	2	11	0.70	7.50
-7	2	-11	-14.40	7.70
-7	2	-11	3.60	11.20
-7	2	-11	14.60	21.70
-8	-2	-11	4.40	22.70
-8	-2	-11	19.80	19.00
-8	2	-11	34.40	24.00
-8	2	-11	14.60	14.90
8	2	11	-15.60	8.00
-9	-2	-11	1.30	19.20
-9	-2	-11	-4.80	21.00
-9	2	-11	6.30	17.20
-9	2	-11	-11.50	20.70
9	2	11	-13.50	11.00
9	-3	-11	19.20	20.20

−9	−3	11	−30.20	24.20
9	−3	−11	−4.80	14.10
−9	3	11	40.30	25.50
9	3	−11	0.00	13.70
−9	3	11	3.50	25.70
−8	−3	11	14.80	20.30
8	−3	−11	−6.10	10.50
8	−3	−11	19.30	17.20
−8	−3	11	−4.10	19.70
−8	3	11	−9.70	19.90
−8	3	11	9.10	18.80
8	3	−11	9.40	10.70
7	−3	−11	24.60	13.50
−7	−3	11	26.50	14.70
−7	−3	11	22.70	20.30
7	−3	−11	52.79	19.70
−7	3	11	47.60	26.00
7	3	−11	55.29	12.40
−7	3	11	66.39	22.00
−6	−3	11	−27.60	8.10
6	−3	−11	−1.90	12.00
6	−3	−11	−11.10	9.10
−6	−3	11	38.00	19.10
−6	3	11	−6.70	15.80
6	3	−11	−3.90	7.30
−6	3	11	29.80	17.10
5	−3	−11	4.00	8.90
5	−3	−11	5.90	10.60
−5	−3	11	18.00	15.30
−5	−3	11	26.00	7.20
−5	3	11	−10.90	13.20

-4	-3	11	78.49	22.60
4	-3	-11	70.89	17.20
4	-3	-11	73.19	13.60
-4	3	11	81.89	20.00
-4	3	11	77.19	21.10
4	3	-11	48.10	11.10
3	-3	-11	-1.20	7.80
-3	-3	11	11.40	13.10
-3	-3	11	3.50	4.70
3	-3	-11	7.40	11.10
-3	-3	11	-1.90	5.70
-3	3	11	-6.40	9.50
3	3	-11	-1.10	6.10
-3	3	11	6.20	7.00
-3	3	11	3.10	13.20
-2	-3	11	114.69	12.90
-2	-3	11	164.58	26.70
-2	-3	11	136.19	19.20
2	-3	-11	125.89	17.10
2	-3	-11	158.18	23.40
-2	-3	11	136.79	11.00
2	3	-11	176.98	26.90
2	3	-11	126.79	14.70
2	3	-11	130.89	15.20
-2	3	11	141.79	16.90
-2	3	11	143.49	28.40
-2	3	11	151.68	23.60
-1	-3	11	4.00	11.40
-1	-3	11	3.30	3.80
1	-3	-11	5.80	7.60
1	-3	-11	12.30	9.80

1	3	-11	-1.90	5.00
1	3	-11	-7.60	12.10
1	3	-11	5.40	8.20
-1	3	11	-2.80	9.20
-1	3	11	-2.90	6.00
-1	3	11	-7.40	11.90
0	-3	11	5.20	11.70
0	-3	-11	7.60	7.50
0	-3	-11	14.60	8.10
0	-3	11	10.40	4.30
0	-3	11	0.00	5.90
0	3	-11	20.20	11.40
0	3	11	-0.90	9.30
0	3	11	20.80	14.40
0	3	-11	-1.90	7.70
0	3	-11	9.70	6.50
0	3	11	6.90	6.30
1	-3	11	277.57	10.70
-1	-3	-11	334.27	26.40
-1	-3	-11	305.47	30.70
-1	-3	-11	294.77	21.40
1	-3	11	336.87	27.60
1	-3	11	286.17	10.40
1	3	11	237.28	31.80
1	3	11	270.97	17.90
1	3	11	280.97	26.80
-1	3	-11	289.57	19.10
-1	3	-11	258.37	22.10
-1	3	-11	277.97	27.50
-2	-3	-11	74.49	14.60
-2	-3	-11	120.09	24.10

2	-3	11	99.09	15.90
-2	-3	-11	107.59	21.70
-2	-3	-11	96.99	14.90
-2	3	-11	125.99	14.40
-2	3	-11	94.99	24.00
2	3	11	104.39	13.70
-2	3	-11	106.49	17.80
2	3	11	88.29	17.60
-2	3	-11	84.59	17.70
-3	-3	-11	9.00	7.80
-3	-3	-11	-7.80	7.40
-3	-3	-11	12.40	12.90
-3	-3	-11	11.80	12.00
3	3	11	1.90	5.70
-3	3	-11	-2.00	13.30
-3	3	-11	12.70	7.90
3	3	11	5.50	9.70
-3	3	-11	11.00	7.50
-3	3	-11	19.60	13.70
-3	3	-11	9.10	9.00
-3	3	-11	-2.00	6.40
-4	-3	-11	90.09	14.20
-4	-3	-11	72.69	13.90
-4	-3	-11	79.09	22.10
-4	3	-11	120.69	18.30
-4	3	-11	68.29	20.00
4	3	11	85.59	20.70
-4	3	-11	90.79	13.00
4	3	11	92.09	12.20
-4	3	-11	81.99	11.80
-5	-3	-11	82.59	13.40

-5	-3	-11	87.89	23.80
-5	-3	-11	97.39	16.60
-5	-3	-11	128.79	27.30
-5	3	-11	111.99	14.30
-5	3	-11	109.29	10.50
5	3	11	85.39	12.20
-5	3	-11	92.59	20.70
5	3	11	57.09	16.50
-5	3	-11	82.59	8.30
-5	3	-11	108.99	17.40
-5	3	-11	58.19	22.60
-6	-3	-11	10.50	17.70
-6	-3	-11	11.50	10.50
-6	-3	-11	20.40	7.60
-6	-3	-11	23.60	15.00
-6	3	-11	-1.90	7.50
-6	3	-11	-6.70	9.20
6	3	11	11.30	7.70
-6	3	-11	14.90	16.60
-7	-3	-11	-7.80	7.30
-7	-3	-11	-9.10	17.40
-7	-3	-11	18.50	21.50
-7	3	-11	-11.90	10.70
7	3	11	3.90	8.00
-7	3	-11	2.50	17.30
-7	3	-11	0.20	9.20
-8	-3	-11	1.10	19.50
-8	-3	-11	-33.00	21.20
-8	3	-11	-26.10	12.10
-8	3	-11	-19.20	23.00
8	3	11	-8.00	8.40

-8	-4	11	-1.50	22.50
8	-4	-11	-10.10	11.10
8	-4	-11	-12.20	16.10
8	4	-11	0.20	10.60
-8	4	11	17.40	20.90
-8	4	11	-0.70	20.80
7	-4	-11	11.30	15.10
-7	-4	11	70.49	25.50
7	-4	-11	39.90	14.30
-7	4	11	-5.60	18.00
7	4	-11	34.80	14.00
-7	4	11	12.70	19.80
6	-4	-11	9.30	16.00
-6	-4	11	29.40	19.70
6	-4	-11	11.10	12.00
-6	4	11	45.30	20.50
-6	4	11	22.70	18.90
6	4	-11	17.80	11.20
5	-4	-11	-5.00	7.00
5	-4	-11	-6.40	8.90
-5	-4	11	6.40	15.80
-5	4	11	22.60	16.80
-5	4	11	4.50	14.30
-4	-4	11	-5.40	13.30
4	-4	-11	-0.10	9.70
4	-4	-11	0.30	8.90
-4	4	11	10.90	13.00
4	4	-11	0.50	7.10
-4	4	11	2.80	13.80
3	-4	-11	220.38	19.10
-3	-4	11	197.68	14.90

-3	-4	11	251.77	32.70
3	-4	-11	219.18	28.10
3	4	-11	201.58	28.60
3	4	-11	200.78	17.40
-3	4	11	254.97	19.80
-3	4	11	213.38	30.80
-3	4	11	263.67	38.90
2	-4	-11	22.30	11.60
-2	-4	11	46.80	13.90
-2	-4	11	36.70	7.20
-2	-4	11	35.80	7.00
2	-4	-11	28.30	10.70
2	4	-11	32.90	15.30
-2	4	11	24.40	11.30
-2	4	11	36.90	9.80
2	4	-11	40.60	8.80
-2	4	11	24.40	17.00
-1	-4	11	4.70	3.80
1	-4	-11	-0.20	6.50
-1	-4	11	5.10	10.30
1	-4	-11	-6.90	7.60
-1	4	11	-4.70	13.20
-1	4	11	-3.90	10.30
-1	4	11	-3.80	5.20
1	4	-11	4.60	7.20
1	4	-11	0.00	6.30
1	4	-11	14.60	12.70
0	-4	11	30.90	12.50
0	-4	-11	43.90	13.60
0	-4	-11	38.50	10.40
0	-4	11	67.99	4.10

0	-4	-11	88.29	20.70
0	4	-11	73.79	16.20
0	4	11	71.89	17.50
0	4	-11	55.29	11.20
0	4	11	35.40	17.20
0	4	-11	69.49	11.30
0	4	11	67.89	11.30
-1	-4	-11	-3.50	10.70
1	-4	11	16.40	10.80
-1	-4	-11	5.40	9.40
-1	-4	-11	-5.50	6.90
1	4	11	0.20	11.10
-1	4	-11	14.30	7.40
-1	4	-11	-1.30	10.40
1	4	11	7.70	7.30
1	4	11	6.50	11.30
-1	4	-11	12.50	8.20
-2	-4	-11	36.40	14.70
-2	-4	-11	29.20	10.60
-2	-4	-11	24.00	13.30
-2	-4	-11	24.40	10.80
2	4	11	18.70	11.50
-2	4	-11	24.10	11.80
-2	4	-11	17.00	13.90
2	4	11	13.30	7.30
-2	4	-11	24.60	8.10
-2	4	-11	26.10	7.80
-3	-4	-11	21.90	14.30
-3	-4	-11	19.40	12.60
-3	-4	-11	12.00	9.20
-3	-4	-11	20.60	10.20

3	4	11	50.99	18.00
-3	4	-11	2.30	11.30
-3	4	-11	4.10	7.40
3	4	11	21.60	7.70
-3	4	-11	18.00	8.50
-3	4	-11	24.80	17.40
-4	-4	-11	5.80	8.60
-4	-4	-11	-3.60	14.20
-4	-4	-11	0.30	14.50
-4	-4	-11	-4.00	7.80
4	4	11	-13.40	5.70
4	4	11	-0.50	11.70
-4	4	-11	-9.00	7.30
-4	4	-11	-9.50	12.40
-4	4	-11	-3.10	6.60
-4	4	-11	18.30	7.80
-4	4	-11	-11.90	14.20
-5	-4	-11	17.90	14.80
-5	-4	-11	27.80	11.20
-5	-4	-11	16.70	17.60
-5	-4	-11	6.00	8.10
-5	4	-11	22.40	9.50
-5	4	-11	7.60	15.00
-5	4	-11	21.20	15.00
-5	4	-11	12.20	5.30
5	4	11	21.80	12.70
-5	4	-11	15.40	6.30
-5	4	-11	25.00	8.70
-6	-4	-11	19.30	19.70
-6	-4	-11	6.00	9.00
-6	-4	-11	13.90	18.90

−6	−4	−11	37.50	9.80
−6	4	−11	19.10	10.80
6	4	11	17.90	8.70
−6	4	−11	27.70	15.40
−6	4	−11	18.90	8.60
−7	−4	−11	43.20	20.50
−7	−4	−11	0.10	9.20
−7	−4	−11	28.40	21.20
−7	4	−11	−2.10	11.50
7	4	11	13.40	9.40
−7	4	−11	15.70	23.10
−8	−4	−11	−27.30	20.80
−8	−4	−11	−5.20	20.60
8	4	11	−3.20	9.50
−8	4	−11	4.50	13.10
−8	−5	11	−2.90	22.40
8	−5	−11	8.10	16.30
8	−5	−11	4.50	12.50
−8	5	11	−25.20	22.10
−8	5	11	−1.00	22.80
7	−5	−11	24.50	16.00
−7	−5	11	0.40	31.20
7	−5	−11	17.60	13.00
−7	5	11	25.60	20.00
−7	5	11	21.50	22.40
7	5	−11	18.30	10.10
−6	−5	11	11.00	18.90
6	−5	−11	8.00	10.50
6	−5	−11	−14.80	11.80
−6	5	11	9.60	14.70
6	5	−11	−8.20	8.90

-6	5	11	1.20	16.00
5	-5	-11	18.00	10.60
-5	-5	11	21.60	15.50
5	-5	-11	37.70	13.80
-5	5	11	26.00	13.70
-5	5	11	1.40	17.10
5	5	-11	7.00	8.70
4	-5	-11	7.40	9.30
4	-5	-11	31.40	12.40
-4	-5	11	17.90	17.00
-4	5	11	4.80	14.70
4	5	-11	-6.70	6.50
-4	5	11	18.70	13.60
3	-5	-11	5.50	10.20
-3	-5	11	18.80	6.90
-3	-5	11	8.50	5.80
-3	-5	11	19.90	15.80
3	5	-11	20.50	7.90
-3	5	11	-9.80	13.00
3	5	-11	20.50	15.90
-3	5	11	13.10	12.10
2	-5	-11	6.70	11.20
-2	-5	11	12.50	4.30
-2	-5	11	11.60	12.60
-2	-5	11	10.30	4.40
2	-5	-11	7.10	7.70
2	5	-11	20.70	7.80
2	5	-11	22.70	14.00
-2	5	11	33.30	16.10
-2	5	11	14.50	14.30
-2	5	11	-1.70	7.80

1	-5	-11	40.80	10.70
-1	-5	11	15.30	14.00
1	-5	-11	58.19	15.20
-1	5	11	23.90	16.90
-1	5	11	25.70	16.10
-1	5	11	39.30	11.30
1	5	-11	22.80	8.00
1	5	-11	54.29	15.40
0	-5	-11	-7.40	7.50
0	-5	11	-10.10	10.30
0	-5	-11	-9.30	10.20
0	-5	-11	-4.70	7.10
0	5	11	-12.00	10.60
0	5	-11	-18.40	6.40
0	5	11	-8.10	6.10
0	5	11	9.80	14.10
0	5	-11	-2.20	10.00
-1	-5	-11	76.79	13.80
1	-5	11	122.39	17.40
-1	-5	-11	106.79	23.40
-1	-5	-11	90.09	24.10
-1	-5	-11	99.19	18.50
-1	5	-11	78.49	14.10
1	5	11	91.29	14.30
-1	5	-11	92.59	12.00
1	5	11	99.09	24.20
1	5	11	94.69	21.40
-2	-5	-11	53.59	13.10
-2	-5	-11	56.19	19.30
-2	-5	-11	40.30	14.90
-2	-5	-11	28.80	10.90

-2	5	-11	36.60	9.60
-2	5	-11	46.60	9.50
-2	5	-11	46.60	11.60
2	5	11	56.79	12.00
-2	5	-11	42.60	17.80
2	5	11	31.60	14.50
-3	-5	-11	1.50	14.50
-3	-5	-11	5.80	8.50
-3	-5	-11	-0.40	8.80
-3	5	-11	16.00	7.00
3	5	11	-3.20	15.10
-3	5	-11	-6.70	8.60
-3	5	-11	5.40	6.70
3	5	11	10.30	8.30
-3	5	-11	20.20	17.40
-4	-5	-11	3.10	14.60
-4	-5	-11	14.90	16.80
-4	-5	-11	19.60	10.10
-4	-5	-11	25.70	11.00
4	5	11	6.40	11.70
4	5	11	24.70	9.50
-4	5	-11	21.90	7.70
-4	5	-11	16.70	10.50
-4	5	-11	2.40	7.30
-4	5	-11	1.10	14.70
-4	5	-11	12.30	8.20
-4	5	-11	18.40	8.30
-5	-5	-11	-5.00	16.80
-5	-5	-11	17.50	9.90
-5	-5	-11	23.90	10.50
-5	-5	-11	45.80	19.80

-5	5	-11	7.90	7.50
5	5	11	21.80	10.30
5	5	11	21.30	17.10
-5	5	-11	15.60	8.60
-5	5	-11	38.00	8.10
-5	5	-11	11.40	7.90
-5	5	-11	35.20	13.70
-5	5	-11	23.30	16.50
-6	-5	-11	-4.40	15.70
-6	-5	-11	21.90	9.80
-6	-5	-11	11.30	10.30
-6	-5	-11	4.80	19.10
-6	5	-11	-8.30	7.20
6	5	11	-13.00	7.60
-6	5	-11	-10.30	8.30
-6	5	-11	27.00	19.20
6	5	11	7.60	14.60
-7	-5	-11	11.20	18.00
-7	-5	-11	0.70	9.70
-7	-5	-11	-19.30	19.70
7	5	11	-3.10	8.70
-7	5	-11	3.40	10.00
-7	5	-11	20.30	23.20
-8	-5	-11	20.50	23.10
-8	-5	-11	-14.60	22.40
8	5	11	-2.90	10.40
-8	5	-11	19.40	19.40
-8	5	-11	14.50	11.90
-8	-6	11	-10.50	26.30
8	-6	-11	10.50	13.70
8	-6	-11	2.40	18.70

-8	6	11	-36.30	23.00
-8	6	11	-19.10	21.40
8	6	-11	-6.70	11.90
7	-6	-11	-4.00	13.90
7	-6	-11	-7.00	10.20
-7	6	11	-4.10	18.90
-7	6	11	-2.80	21.50
7	6	-11	17.00	11.40
6	-6	-11	24.60	12.30
-6	-6	11	-1.30	21.50
6	-6	-11	-10.00	13.80
6	6	-11	10.70	8.50
-6	6	11	10.40	17.70
-6	6	11	-11.80	19.90
5	-6	-11	5.90	9.20
-5	-6	11	3.70	17.00
5	-6	-11	-5.40	11.70
5	6	-11	5.60	8.60
-5	6	11	-13.90	12.30
-5	6	11	0.90	17.40
4	-6	-11	-13.70	11.00
4	-6	-11	4.60	9.10
4	6	-11	2.70	7.50
-4	6	11	25.80	17.30
-4	6	11	-4.40	15.00
3	-6	-11	38.20	11.70
-3	-6	11	61.89	7.80
-3	-6	11	61.39	7.90
-3	-6	11	58.39	16.60
3	-6	-11	54.19	16.40
-3	6	11	31.90	10.70

3	6	-11	69.79	18.00
3	6	-11	56.79	11.30
-3	6	11	32.60	20.90
-3	6	11	16.10	14.60
-2	-6	11	33.90	14.30
-2	6	11	10.20	14.60
-2	6	11	26.90	9.40
2	6	-11	30.10	8.20
-2	6	11	9.90	14.80
2	6	-11	32.90	14.10
1	-6	-11	65.29	18.30
1	-6	-11	61.49	12.20
1	-6	-11	67.19	15.70
-1	-6	11	68.89	17.60
-1	6	11	70.99	14.90
-1	6	11	32.30	17.50
1	6	-11	50.39	9.70
1	6	-11	57.79	15.60
-1	6	11	49.20	18.50
0	-6	11	49.80	12.40
0	-6	-11	25.00	11.70
0	-6	-11	39.80	15.00
0	-6	-11	25.90	9.20
0	6	-11	28.50	7.70
0	6	-11	12.10	7.00
0	6	11	18.20	10.70
0	6	-11	29.90	10.70
0	6	11	7.20	13.70
0	6	11	31.20	17.50
-1	-6	-11	54.89	20.30
-1	-6	-11	62.69	16.30

-1	-6	-11	88.79	25.40
1	-6	11	17.40	10.70
-1	6	-11	59.39	10.60
-1	6	-11	60.09	11.00
-1	6	-11	45.10	19.10
-1	6	-11	45.20	9.30
-1	6	-11	58.69	10.00
1	6	11	47.40	16.30
1	6	11	35.30	17.80
1	6	11	36.80	10.90
-2	-6	-11	2.90	9.60
-2	-6	-11	-2.90	13.00
-2	-6	-11	6.30	8.70
-2	-6	-11	-17.30	16.50
-2	6	-11	7.60	6.20
-2	6	-11	-7.50	6.00
-2	6	-11	15.30	14.50
-2	6	-11	7.60	6.80
2	6	11	9.70	7.70
-3	-6	-11	57.29	23.60
-3	-6	-11	2.90	10.90
-3	-6	-11	23.60	10.80
-3	-6	-11	42.40	17.50
-3	6	-11	31.60	6.30
-3	6	-11	5.70	6.90
-3	6	-11	16.20	18.70
3	6	11	26.70	10.20
3	6	11	-5.60	13.00
-4	-6	-11	0.10	16.00
-4	-6	-11	1.80	8.20
-4	-6	-11	12.90	14.60

−4	−6	−11	3.00	9.00
−4	6	−11	−8.60	13.60
−4	6	−11	7.20	7.20
−4	6	−11	−20.10	7.70
4	6	11	17.10	9.30
4	6	11	−4.90	13.90
−5	−6	−11	−15.10	9.20
−5	−6	−11	−17.90	15.90
−5	−6	−11	−25.90	15.80
−5	−6	−11	−7.80	9.10
−5	6	−11	6.10	7.60
5	6	11	13.90	15.50
−5	6	−11	−1.60	16.80
5	6	11	12.70	8.80
−6	−6	−11	20.20	17.20
−6	−6	−11	51.49	24.60
−6	−6	−11	24.90	11.10
6	6	11	0.30	15.90
−6	6	−11	0.80	7.80
6	6	11	29.80	10.70
−6	6	−11	−5.50	18.70
−7	−6	−11	−9.20	10.40
−7	−6	−11	−2.40	21.60
−7	−6	−11	22.10	24.00
−7	6	−11	−21.50	19.70
7	6	11	−1.90	10.10
−7	6	−11	12.10	7.40
−8	−6	−11	40.30	26.70
−8	6	−11	4.40	10.00
−8	6	−11	45.10	27.80
7	−7	−11	23.20	12.40

7	-7	-11	19.10	17.00
-7	7	11	7.80	21.20
7	7	-11	-2.40	10.80
6	-7	-11	16.10	17.20
6	-7	-11	-6.70	12.10
-6	-7	11	23.30	23.10
6	7	-11	-12.70	8.10
-6	7	11	-16.80	18.60
-6	7	11	6.40	20.60
-5	-7	11	55.29	24.40
5	-7	-11	26.00	10.30
5	-7	-11	57.99	20.40
5	7	-11	11.20	10.20
-5	7	11	9.20	18.10
-5	7	11	8.10	18.00
4	-7	-11	28.00	13.90
-4	-7	11	22.80	7.10
-4	-7	11	25.50	16.90
4	-7	-11	30.80	11.80
-4	7	11	54.79	21.60
-4	7	11	22.80	11.60
-4	7	11	44.90	21.00
4	7	-11	28.50	9.90
3	-7	-11	-5.30	10.10
-3	-7	11	1.90	3.00
-3	-7	11	3.60	15.90
3	-7	-11	9.10	9.50
3	7	-11	0.20	6.70
-3	7	11	3.90	15.60
3	7	-11	-8.00	14.10
-3	7	11	-5.90	15.60

-3	7	11	-0.30	8.30
-2	-7	11	62.59	16.30
2	-7	-11	44.00	11.50
2	-7	-11	61.49	17.20
-2	7	11	58.99	19.50
-2	7	11	58.89	12.40
2	7	-11	32.20	8.60
2	7	-11	48.00	15.30
-2	7	11	121.89	26.90
1	-7	-11	21.60	14.90
1	-7	-11	25.40	10.60
1	-7	-11	27.90	12.80
-1	7	11	17.30	19.10
-1	7	11	19.50	9.40
-1	7	11	32.80	19.30
1	7	-11	18.20	11.10
1	7	-11	30.60	8.10
0	-7	-11	1.80	8.00
0	-7	-11	-10.50	11.00
0	-7	11	-6.50	10.60
0	7	-11	-1.60	8.00
0	7	-11	3.00	6.00
0	7	11	-9.20	12.70
0	7	11	-4.50	16.90
0	7	11	-4.00	9.10
-1	-7	-11	33.60	15.90
-1	-7	-11	27.40	10.20
-1	-7	-11	31.40	19.70
-1	-7	-11	49.60	15.10
-1	7	-11	19.40	10.40
1	7	11	28.40	10.90

-1	7	-11	31.10	16.80
-1	7	-11	38.80	8.40
1	7	11	18.30	15.30
-1	7	-11	37.20	8.80
-1	7	-11	30.70	6.90
-1	7	-11	40.80	10.00
-2	-7	-11	51.69	15.80
-2	-7	-11	95.39	16.40
-2	-7	-11	117.19	29.80
-2	-7	-11	84.59	21.20
2	7	11	76.79	20.60
2	7	11	87.39	15.10
-2	7	-11	109.69	18.30
-2	7	-11	88.39	12.00
-3	-7	-11	-10.80	17.10
-3	-7	-11	5.10	10.60
-3	-7	-11	10.00	8.90
-3	-7	-11	-9.30	15.10
-3	7	-11	-3.60	6.70
3	7	11	-9.00	12.60
3	7	11	-0.60	8.70
-3	7	-11	-7.30	15.60
-4	-7	-11	47.60	21.10
-4	-7	-11	9.30	11.70
-4	-7	-11	43.60	19.40
-4	-7	-11	55.29	14.50
4	7	11	53.49	20.20
4	7	11	48.90	12.60
-4	7	-11	45.00	20.20
-4	7	-11	36.70	8.70
-5	-7	-11	14.40	10.40

-5	-7	-11	9.00	18.70
-5	-7	-11	13.60	11.00
-5	-7	-11	16.30	18.30
-5	7	-11	5.10	16.70
-5	7	-11	27.50	8.70
5	7	11	11.50	10.00
5	7	11	-6.90	16.30
-6	-7	-11	-20.00	11.70
-6	-7	-11	19.60	22.00
-6	-7	-11	12.60	19.40
6	7	11	-10.40	9.00
6	7	11	15.20	18.30
-6	7	-11	-11.60	7.20
-6	7	-11	31.30	20.70
-7	-7	-11	-7.20	12.50
-7	-7	-11	-30.00	22.70
-7	-7	-11	4.60	19.40
-7	7	-11	-35.70	19.80
7	7	11	-4.00	18.40
7	7	11	-18.70	10.80
7	-8	-11	27.60	14.00
-7	8	11	-12.80	20.80
7	8	-11	27.30	12.20
6	-8	-11	5.20	12.50
-6	-8	11	-19.20	23.90
6	-8	-11	-22.50	15.00
6	8	-11	4.60	9.90
-6	8	11	-5.80	22.90
-6	8	11	-5.40	19.40
5	-8	-11	16.60	10.80
5	-8	-11	-13.80	11.70

-5	-8	11	34.60	23.30
-5	8	11	-5.50	18.80
5	8	-11	1.90	9.60
-5	8	11	21.20	19.50
4	-8	-11	9.20	9.50
-4	-8	11	8.10	17.80
4	-8	-11	-5.90	10.80
4	8	-11	-3.40	7.60
-4	8	11	10.10	16.50
-4	8	11	-3.40	16.20
-4	8	11	-4.40	9.70
3	-8	-11	26.90	13.30
3	-8	-11	6.20	9.90
-3	8	11	29.00	17.40
-3	8	11	-16.50	17.30
3	8	-11	8.00	7.70
-3	8	11	1.00	9.80
2	-8	-11	44.10	17.50
-2	-8	11	25.10	17.90
2	-8	-11	33.10	13.90
2	-8	-11	9.50	8.90
2	8	-11	14.50	8.00
-2	8	11	-1.10	15.20
-2	8	11	12.20	17.70
-2	8	11	16.90	10.60
-1	-8	11	33.80	15.60
1	-8	-11	-7.00	10.60
1	-8	-11	21.10	15.40
1	-8	-11	5.40	7.90
-1	8	11	2.90	9.50
-1	8	11	-15.00	12.80

-1	8	11	-7.00	16.60
1	8	-11	3.20	6.40
1	8	-11	6.80	10.00
0	-8	-11	43.10	16.70
0	-8	-11	36.00	10.70
0	-8	-11	7.60	11.40
0	8	11	15.80	17.70
0	8	-11	12.10	7.40
0	8	11	32.00	16.50
0	8	11	42.60	13.60
0	8	-11	15.00	10.90
-1	-8	-11	7.10	11.00
-1	-8	-11	14.40	18.10
-1	-8	-11	6.20	14.40
-1	-8	-11	1.30	9.80
-1	8	-11	-3.70	9.70
-1	8	-11	3.20	8.20
1	8	11	-7.40	14.60
-1	8	-11	10.90	6.40
-1	8	-11	6.80	15.40
1	8	11	-5.40	8.30
-2	-8	-11	-16.00	13.50
-2	-8	-11	0.50	17.00
-2	-8	-11	2.10	12.50
-2	-8	-11	-4.60	8.90
2	8	11	5.10	8.10
-2	8	-11	8.60	7.00
-2	8	-11	22.20	11.50
2	8	11	-11.10	13.60
-2	8	-11	21.40	17.90
-3	-8	-11	73.49	19.30

-3	-8	-11	31.80	20.70
-3	-8	-11	28.50	13.00
-3	-8	-11	34.40	12.40
-3	8	-11	30.80	8.80
3	8	11	51.59	13.40
3	8	11	29.60	18.00
-3	8	-11	29.80	16.60
-3	8	-11	75.89	22.70
-4	-8	-11	13.10	12.00
-4	-8	-11	-5.20	11.00
-4	-8	-11	-10.70	16.30
-4	-8	-11	-24.30	18.20
-4	8	-11	-21.40	16.40
4	8	11	-14.80	15.90
4	8	11	2.50	10.60
-4	8	-11	10.70	7.10
-4	8	-11	37.00	16.10
-5	-8	-11	-12.60	22.80
-5	-8	-11	3.60	11.40
-5	-8	-11	-3.70	17.00
-5	-8	-11	6.80	12.70
-5	8	-11	13.50	7.00
-5	8	-11	6.90	19.10
5	8	11	-9.60	18.90
-5	8	-11	24.70	19.50
5	8	11	13.20	11.50
-6	-8	-11	-1.70	23.80
-6	-8	-11	-2.10	13.50
-6	-8	-11	6.50	21.50
-6	8	-11	14.20	19.80
6	8	11	-16.40	10.20

-6	8	-11	30.90	19.10
-7	-8	-11	-22.80	14.10
-7	-8	-11	5.00	24.30
-7	-8	-11	-0.30	25.90
-7	8	-11	36.00	24.00
-7	8	-11	2.60	37.60
-6	-9	11	-33.60	26.40
6	-9	-11	1.60	13.30
6	9	-11	-28.60	10.20
-6	9	11	-18.00	20.80
5	-9	-11	33.70	12.90
-5	-9	11	-2.20	21.90
5	-9	-11	15.90	17.10
-5	9	11	39.40	13.20
-5	9	11	47.80	24.00
-5	9	11	36.50	24.80
5	9	-11	28.30	11.90
4	-9	-11	3.50	10.40
4	-9	-11	6.40	13.80
-4	-9	11	10.90	22.50
-4	9	11	-6.10	9.20
-4	9	11	-15.10	17.90
-4	9	11	11.50	21.90
3	-9	-11	5.70	9.80
-3	-9	11	11.80	19.80
3	-9	-11	17.50	16.10
3	-9	-11	-3.40	12.80
-3	9	11	-2.30	9.80
3	9	-11	7.20	7.60
-3	9	11	-0.20	17.50
-3	9	11	-24.00	16.60

2	-9	-11	13.40	23.60
2	-9	-11	6.70	11.90
2	-9	-11	-2.20	9.70
2	9	-11	-4.00	7.10
-2	9	11	12.60	19.80
-2	9	11	-26.60	15.00
-2	9	11	-6.90	9.50
1	-9	-11	86.19	20.90
1	-9	-11	101.89	23.60
1	-9	-11	60.09	12.90
-1	-9	11	10.70	16.80
-1	9	11	102.89	27.80
1	9	-11	53.69	10.40
-1	9	11	35.20	21.10
-1	9	11	71.09	14.40
1	9	-11	58.29	10.40
0	-9	-11	1.50	10.30
0	-9	-11	4.90	10.30
0	-9	-11	7.50	20.10
0	-9	-11	3.50	14.70
0	9	-11	-2.10	6.40
0	9	11	1.50	9.80
0	9	11	2.20	16.90
-1	-9	-11	-27.30	10.40
-1	-9	-11	-10.30	9.50
-1	-9	-11	9.30	19.50
-1	-9	-11	-28.70	12.80
-1	9	-11	-6.00	16.60
1	9	11	-6.10	9.80
1	9	11	1.40	19.00
-1	9	-11	11.60	6.70

-1	9	-11	-16.10	10.00
-2	-9	-11	-28.20	20.00
-2	-9	-11	15.40	13.70
-2	-9	-11	26.80	16.10
-2	-9	-11	-9.80	10.40
2	9	11	12.70	10.90
2	9	11	12.40	17.50
-2	9	-11	3.20	7.30
-2	9	-11	7.80	14.20
-2	9	-11	-16.20	14.50
-3	-9	-11	1.30	17.60
-3	-9	-11	-7.30	12.10
-3	-9	-11	11.80	11.50
-3	-9	-11	3.40	20.70
3	9	11	-1.30	15.60
-3	9	-11	5.00	16.00
3	9	11	-7.60	10.10
-3	9	-11	2.00	18.20
-3	9	-11	-13.50	6.80
-4	-9	-11	0.60	12.00
-4	-9	-11	-8.40	20.70
-4	-9	-11	27.60	17.30
-4	-9	-11	14.10	11.90
4	9	11	-18.70	18.70
-4	9	-11	4.10	17.00
4	9	11	26.40	12.20
-4	9	-11	14.00	19.40
-5	-9	-11	19.60	19.90
-5	-9	-11	5.10	12.90
-5	-9	-11	-31.20	21.40
5	9	11	2.00	10.20

5	9	11	-11.20	23.50
-5	9	-11	-4.20	17.40
-5	9	-11	-23.00	20.60
-6	-9	-11	-48.40	24.70
-6	-9	-11	1.80	23.70
-6	-9	-11	7.80	14.40
-6	9	-11	11.80	21.30
6	9	11	13.80	24.60
6	9	11	-4.70	12.70
5	-10	-11	4.90	16.40
5	-10	-11	20.60	13.30
-5	10	11	9.90	25.60
-5	10	11	-12.50	21.50
5	10	-11	-3.80	9.80
4	-10	-11	5.50	12.20
4	-10	-11	9.20	13.10
-4	10	11	31.70	24.00
-4	10	11	-8.50	22.40
-4	10	11	8.20	12.20
4	10	-11	1.30	9.40
3	-10	-11	15.00	10.10
3	-10	-11	22.80	18.10
3	-10	-11	-10.60	12.40
-3	10	11	-3.80	9.70
3	10	-11	-4.70	8.10
-3	10	11	-9.70	18.30
-3	10	11	24.80	20.50
2	-10	-11	8.20	11.20
2	-10	-11	11.50	16.40
2	-10	-11	23.10	15.40
-2	10	11	-3.90	17.80

-2	10	11	9.50	12.30
-2	10	11	13.40	21.40
2	10	-11	14.50	9.00
1	-10	-11	46.40	13.10
1	-10	-11	32.30	16.40
1	-10	-11	33.30	18.00
1	10	-11	52.59	10.00
-1	10	11	36.30	22.70
-1	10	11	71.19	26.30
-1	10	11	69.09	17.00
0	-10	-11	-3.90	10.30
0	-10	-11	-18.00	11.70
0	-10	-11	-2.10	23.60
0	-10	-11	-21.80	14.50
0	10	11	-25.70	18.30
0	10	-11	4.70	7.00
0	10	11	11.20	10.70
-1	-10	-11	9.60	11.20
-1	-10	-11	6.30	12.90
-1	-10	-11	-24.60	24.50
-1	-10	-11	4.70	25.10
-1	10	-11	11.90	7.10
1	10	11	10.90	12.70
-1	10	-11	-2.90	15.90
1	10	11	4.00	18.10
-2	-10	-11	7.60	17.20
-2	-10	-11	-9.20	11.30
-2	-10	-11	-5.20	9.90
-2	-10	-11	11.80	23.50
-2	10	-11	12.70	19.90
-2	10	-11	20.50	16.00

2	10	11	7.20	11.70
2	10	11	-14.10	17.10
-3	-10	-11	20.60	17.30
-3	-10	-11	16.00	20.40
-3	-10	-11	41.70	14.90
-3	-10	-11	10.20	24.00
-3	10	-11	16.70	19.80
-3	10	-11	48.10	19.70
3	10	11	26.10	14.40
3	10	11	31.90	21.20
-4	-10	-11	4.90	18.80
-4	-10	-11	30.20	25.20
-4	-10	-11	-7.30	14.30
-4	10	-11	28.20	22.10
-4	10	-11	24.70	18.80
-5	-10	-11	23.10	22.90
-5	-10	-11	22.80	15.80
-5	-10	-11	3.70	33.90
-5	10	-11	-22.20	19.60
-5	10	-11	-21.30	19.50
5	10	11	8.50	21.80
5	10	11	-2.80	12.60
4	-11	-11	-2.20	14.70
4	-11	-11	-1.70	10.30
-4	11	11	13.50	20.40
4	11	-11	6.50	11.20
-4	11	11	7.80	13.90
-4	11	11	0.90	22.80
3	-11	-11	-5.20	11.10
3	-11	-11	35.10	23.00
3	11	-11	-9.90	9.30

-3	11	11	21.10	37.40
-3	11	11	-50.69	35.10
-3	11	11	-9.60	11.50
2	-11	-11	26.20	15.20
2	-11	-11	-17.90	11.10
-2	11	11	27.80	22.30
2	11	-11	6.20	8.60
-2	11	11	-16.90	12.20
-2	11	11	-25.80	21.50
1	-11	-11	12.20	14.20
1	-11	-11	55.19	27.40
1	-11	-11	13.80	11.90
-1	11	11	17.30	25.40
1	11	-11	5.50	8.20
-1	11	11	11.90	12.80
0	-11	-11	5.40	25.00
0	-11	-11	21.60	12.80
0	-11	-11	8.70	13.70
0	11	-11	5.90	19.30
0	11	11	10.10	13.10
0	11	11	2.50	23.60
0	11	-11	7.10	13.00
-1	-11	-11	-0.90	14.30
-1	-11	-11	-3.00	10.90
-1	-11	-11	-12.30	23.70
1	11	11	4.90	21.70
-1	11	-11	41.80	21.90
1	11	11	9.40	11.90
-2	-11	-11	16.40	13.20
-2	-11	-11	50.89	27.10
-2	-11	-11	19.70	16.40

2	11	11	13.00	12.40
2	11	11	-23.10	20.60
-2	11	-11	4.20	17.90
-3	-11	-11	30.90	19.40
-3	-11	-11	14.00	14.40
-3	-11	-11	-33.70	24.90
-3	11	-11	-18.70	19.50
-3	11	-11	20.50	20.30
3	11	11	12.00	12.60
3	11	11	35.90	25.40
-4	-11	-11	-31.20	21.70
-4	-11	-11	-41.30	28.20
-4	-11	-11	-12.60	15.50
-4	11	-11	2.70	23.20
4	11	11	-40.80	19.20
4	11	11	-4.80	11.80
-3	12	11	14.40	22.70
2	-12	-11	61.89	32.80
2	-12	-11	2.40	15.40
2	-12	-11	11.80	13.10
-2	12	11	17.50	13.50
2	12	-11	-12.70	9.30
-2	12	11	14.10	25.50
1	-12	-11	5.50	17.90
1	-12	-11	10.60	13.70
1	-12	-11	8.30	27.80
1	12	-11	5.90	9.50
-1	12	11	23.40	15.10
-1	12	11	-7.80	21.50
0	-12	-11	10.20	28.10
0	-12	-11	-20.70	11.60

0	-12	-11	36.60	18.80
0	12	11	19.90	28.50
0	12	11	30.50	14.50
0	12	-11	-11.20	21.40
-1	-12	-11	15.20	13.60
-1	-12	-11	-22.40	14.10
-1	-12	-11	-18.50	27.10
-1	12	-11	-42.30	19.30
1	12	11	15.60	15.10
1	12	11	25.90	25.50
-2	-12	-11	-10.60	17.40
-2	-12	-11	6.40	31.50
-2	12	-11	25.90	20.90
2	12	11	-30.70	22.60
2	12	11	11.50	14.80
8	0	-12	-4.50	15.30
-8	0	12	-6.00	23.50
-8	0	12	15.60	19.50
7	0	-12	41.70	17.00
-7	0	12	12.50	18.50
-7	0	12	13.50	15.90
7	0	-12	-1.00	9.90
-6	0	12	101.29	26.10
-6	0	12	94.59	21.20
6	0	-12	67.89	14.30
6	0	-12	96.89	20.00
5	0	-12	-4.60	11.30
-5	0	12	10.00	14.80
-5	0	12	-6.60	11.80
5	0	-12	14.20	9.30
-4	0	12	-19.80	15.10

4	0	-12	24.10	12.70
-4	0	12	5.30	15.30
-3	0	12	9.40	9.20
3	0	-12	-10.20	8.20
-3	0	12	4.80	14.70
3	0	-12	3.10	7.10
-3	0	12	0.00	7.00
2	0	-12	36.60	11.50
2	0	-12	42.20	13.40
-2	0	12	12.50	14.30
-2	0	12	48.00	12.30
-2	0	12	47.30	9.50
1	0	-12	1.30	6.90
-1	0	12	2.00	13.00
-1	0	12	2.60	5.90
-1	0	12	3.60	12.40
1	0	-12	2.10	8.80
0	0	12	95.89	24.90
0	0	12	62.89	13.50
0	0	-12	76.39	13.70
0	0	12	47.60	9.60
0	0	-12	65.59	15.00
1	0	12	3.60	7.80
-1	0	-12	3.30	9.00
1	0	12	2.40	12.30
1	0	12	-1.60	5.40
-1	0	-12	1.60	7.50
-1	0	-12	-3.80	11.80
2	0	12	116.99	20.00
-2	0	-12	130.59	27.70
-2	0	-12	95.69	15.70

-2	0	-12	106.39	15.90
-2	0	-12	153.98	28.20
-2	0	-12	75.59	18.60
2	0	12	94.79	11.10
2	0	12	126.29	24.20
-3	0	-12	2.30	13.60
-3	0	-12	6.90	7.80
-3	0	-12	0.20	7.60
-3	0	-12	-4.00	11.10
3	0	12	-3.40	4.90
-3	0	-12	1.90	18.70
-4	0	-12	15.40	8.80
-4	0	-12	-29.80	13.60
-4	0	-12	6.20	8.00
4	0	12	5.00	5.30
-5	0	-12	6.50	8.50
-5	0	-12	-18.40	15.30
-5	0	-12	11.10	14.00
5	0	12	4.50	5.90
-5	0	-12	-6.70	6.50
6	0	12	34.80	8.30
-6	0	-12	17.90	15.00
-6	0	-12	31.60	6.80
-6	0	-12	45.00	22.80
-6	0	-12	45.10	12.40
-7	0	-12	-14.80	14.60
-7	0	-12	-9.50	17.80
7	0	12	1.10	7.40
-7	0	-12	-12.40	8.90
-8	0	-12	-9.90	16.50
-8	0	-12	14.40	27.50

8	0	12	-14.50	9.20
-8	-1	12	48.30	20.60
8	-1	-12	4.80	22.20
-8	-1	12	16.90	21.20
8	-1	-12	3.10	14.10
8	1	-12	-15.50	15.90
-8	1	12	20.60	22.30
-8	1	12	5.50	20.00
7	-1	-12	11.80	13.10
-7	-1	12	-7.30	19.20
7	-1	-12	1.00	11.00
-7	-1	12	-22.50	14.70
-7	1	12	2.10	20.30
-7	1	12	3.40	16.60
7	1	-12	-1.10	9.70
6	-1	-12	7.70	13.00
6	-1	-12	12.40	10.60
-6	-1	12	-11.10	19.30
-6	1	12	-1.30	18.30
6	1	-12	-11.50	10.00
-6	1	12	-3.90	15.70
5	-1	-12	7.20	10.50
5	-1	-12	10.30	13.60
-5	-1	12	6.40	13.80
-5	-1	12	26.00	12.60
-5	1	12	-16.20	14.00
-5	1	12	8.00	12.70
5	1	-12	6.90	8.20
4	-1	-12	48.80	13.60
-4	-1	12	85.09	15.60
4	-1	-12	64.19	16.70

-4	-1	12	40.70	19.70
-4	1	12	55.49	18.50
-4	1	12	56.49	17.30
4	1	-12	90.09	18.10
4	1	-12	104.59	19.30
3	-1	-12	-0.90	9.00
-3	-1	12	-4.60	12.90
-3	-1	12	7.80	7.10
-3	-1	12	16.10	7.70
3	-1	-12	4.10	9.30
3	1	-12	-2.30	12.90
-3	1	12	-8.50	13.10
-3	1	12	8.50	7.70
-3	1	12	-4.90	10.10
3	1	-12	5.90	8.50
2	-1	-12	179.78	24.30
-2	-1	12	178.78	14.70
-2	-1	12	141.89	25.70
-2	-1	12	169.48	18.00
2	-1	-12	179.88	20.10
2	1	-12	184.28	16.70
-2	1	12	163.48	15.00
-2	1	12	177.48	22.70
-2	1	12	151.78	28.70
2	1	-12	165.68	22.80
-1	-1	12	0.50	5.40
-1	-1	12	2.10	8.00
1	-1	-12	-3.70	9.50
-1	-1	12	-12.10	12.30
1	-1	-12	-0.60	7.40
1	1	-12	1.60	8.80

-1	1	12	-17.00	7.50
-1	1	12	-5.10	5.70
-1	1	12	19.00	15.70
1	1	-12	6.10	11.90
1	1	-12	13.10	8.40
0	-1	12	16.30	9.20
0	-1	-12	15.90	8.70
0	-1	12	8.70	5.70
0	-1	-12	2.70	9.70
0	-1	12	-7.50	12.40
0	1	-12	5.80	8.10
0	1	-12	12.40	10.30
0	1	12	18.50	11.00
0	1	12	21.80	7.90
0	1	12	17.60	13.10
0	1	-12	-22.00	15.40
-1	-1	-12	3.70	13.00
1	-1	12	-1.50	4.80
1	-1	12	-4.70	11.00
-1	-1	-12	11.50	8.00
1	-1	12	4.20	7.60
-1	-1	-12	0.00	8.80
1	-1	12	7.10	10.20
1	1	12	7.10	9.50
-1	1	-12	-1.00	6.80
-1	1	-12	-6.10	7.50
-1	1	-12	3.60	14.60
-1	1	-12	-34.60	15.20
1	1	12	-2.70	5.40
1	1	12	3.90	12.80
2	-1	12	157.98	13.00

-2	-1	-12	115.69	22.30
-2	-1	-12	172.78	20.80
2	-1	12	148.29	11.10
-2	-1	-12	153.08	20.90
2	-1	12	131.69	23.70
-2	-1	-12	139.39	29.00
-2	1	-12	114.09	25.90
2	1	12	147.89	12.60
-2	1	-12	141.49	24.40
-2	1	-12	155.98	18.40
2	1	12	130.59	21.30
-2	1	-12	134.49	18.40
2	1	12	136.09	24.20
-2	1	-12	115.29	24.90
-3	-1	-12	1.70	7.70
-3	-1	-12	12.70	14.20
3	-1	12	5.00	6.80
-3	-1	-12	-11.70	8.50
3	-1	12	4.80	4.50
-3	-1	-12	2.90	11.20
-3	1	-12	5.40	9.50
-3	1	-12	16.70	18.60
-3	1	-12	-17.70	16.20
3	1	12	7.80	10.30
-3	1	-12	8.30	8.50
-3	1	-12	-7.60	11.50
3	1	12	8.90	6.00
4	-1	12	6.10	4.80
-4	-1	-12	20.50	13.00
-4	-1	-12	24.40	10.50
-4	-1	-12	25.70	11.40

-4	-1	-12	17.20	17.50
-4	1	-12	25.40	16.70
-4	1	-12	41.30	21.60
-4	1	-12	17.30	8.60
-4	1	-12	18.00	11.60
4	1	12	21.00	6.90
-4	1	-12	14.70	9.10
5	-1	12	-4.50	4.60
-5	-1	-12	-4.40	12.90
-5	-1	-12	8.30	7.80
-5	-1	-12	4.10	15.40
5	1	12	-3.30	6.20
-5	1	-12	20.80	16.30
-5	1	-12	-4.40	11.50
-5	1	-12	7.80	9.10
-5	1	-12	33.30	20.70
6	-1	12	4.00	5.50
-6	-1	-12	22.10	10.20
-6	-1	-12	9.10	15.30
-6	-1	-12	-5.20	15.50
6	1	12	-3.90	6.40
-6	1	-12	12.60	17.20
-6	1	-12	21.30	13.60
-7	-1	-12	18.00	22.10
7	-1	12	-8.50	6.50
-7	-1	-12	-11.40	15.60
-7	-1	-12	-1.70	9.10
-7	1	-12	-7.10	9.40
7	1	12	3.90	8.00
-7	1	-12	35.90	17.50
-7	1	-12	13.50	18.60

-8	-1	-12	9.90	19.40
-8	1	-12	-20.00	14.20
-8	1	-12	40.90	26.30
8	1	12	-13.20	8.80
-8	-2	12	39.10	23.90
8	-2	-12	5.30	17.50
-8	2	12	18.70	22.40
-8	2	12	-0.40	22.50
7	-2	-12	3.80	11.10
-7	-2	12	2.40	15.50
-7	-2	12	-0.30	19.70
7	-2	-12	1.00	15.20
-7	2	12	-10.90	18.70
7	2	-12	-15.40	9.60
-7	2	12	16.50	18.20
-6	-2	12	104.49	17.90
6	-2	-12	69.09	20.90
-6	-2	12	46.60	20.50
6	-2	-12	73.29	15.30
-6	2	12	85.49	24.80
-6	2	12	57.39	20.70
6	2	-12	86.29	15.40
5	-2	-12	-7.70	7.90
5	-2	-12	-10.20	12.60
-5	-2	12	-17.10	15.40
-5	2	12	-17.50	14.80
-5	2	12	15.00	15.40
5	2	-12	-1.60	9.50
-4	-2	12	6.00	8.20
4	-2	-12	-5.30	9.30
4	-2	-12	-1.00	8.90

-4	-2	12	5.10	15.60
4	2	-12	1.30	8.90
-4	2	12	6.70	14.00
-4	2	12	7.20	13.00
-3	-2	12	9.20	6.30
-3	-2	12	11.90	7.90
3	-2	-12	14.80	8.00
-3	-2	12	26.30	15.70
3	-2	-12	8.10	10.30
3	2	-12	-2.00	10.40
-3	2	12	-2.70	6.70
-3	2	12	-5.40	13.30
3	2	-12	10.90	8.90
-3	2	12	2.20	11.60
2	-2	-12	87.09	17.90
-2	-2	12	92.99	12.50
2	-2	-12	104.59	16.60
-2	2	12	53.79	18.20
-2	2	12	87.39	18.80
-2	2	12	115.19	15.30
2	2	-12	78.99	17.40
2	2	-12	109.99	15.20
1	-2	-12	-0.60	8.20
-1	-2	12	8.60	13.70
-1	-2	12	6.70	7.30
1	-2	-12	-4.20	7.40
-1	-2	12	-7.00	4.50
-1	2	12	2.00	10.00
1	2	-12	-2.60	14.90
1	2	-12	6.60	11.50
1	2	-12	6.80	7.20

-1	2	12	6.90	6.50
-1	2	12	9.50	14.60
0	-2	-12	108.69	19.30
0	-2	12	132.49	25.10
0	-2	12	110.49	9.90
0	-2	12	108.69	11.40
0	-2	-12	105.79	15.60
0	-2	12	125.19	16.20
0	2	12	129.49	24.80
0	2	-12	152.48	19.50
0	2	-12	119.19	16.00
0	2	12	115.59	13.90
0	2	12	100.99	19.00
-1	-2	-12	-29.70	12.70
1	-2	12	4.10	5.70
-1	-2	-12	8.50	8.40
1	-2	12	2.40	4.30
1	-2	12	-5.10	12.60
-1	-2	-12	6.10	6.50
1	2	12	-4.90	9.00
-1	2	-12	11.20	7.20
-1	2	-12	15.60	14.10
-1	2	-12	20.20	9.50
1	2	12	6.70	5.70
-1	2	-12	-24.10	12.80
1	2	12	1.60	14.20
-2	-2	-12	50.89	12.00
-2	-2	-12	81.99	26.00
2	-2	12	83.39	7.30
2	-2	12	101.49	19.90
-2	-2	-12	120.09	24.50

2	-2	12	88.99	8.80
-2	-2	-12	72.29	14.90
-2	2	-12	112.29	27.40
2	2	12	90.49	19.30
-2	2	-12	49.40	11.60
-2	2	-12	66.89	16.10
-2	2	-12	47.30	18.60
-2	2	-12	85.69	15.90
2	2	12	96.99	12.90
-3	-2	-12	24.20	10.60
-3	-2	-12	8.50	8.70
3	-2	12	-6.90	4.40
-3	-2	-12	17.20	18.30
-3	2	-12	-44.00	15.40
-3	2	-12	-13.10	23.50
-3	2	-12	5.90	8.00
3	2	12	3.90	11.60
-3	2	-12	19.00	11.90
3	2	12	14.10	7.10
-4	-2	-12	17.60	11.30
-4	-2	-12	26.20	10.00
4	-2	12	23.90	3.70
-4	-2	-12	30.30	18.00
-4	-2	-12	41.20	18.80
4	2	12	35.30	9.20
4	2	12	28.60	19.00
-4	2	-12	29.50	18.40
-4	2	-12	46.10	19.10
-4	2	-12	28.60	13.10
-4	2	-12	42.40	10.40
-4	2	-12	23.20	8.80

−5	−2	−12	−3.10	14.50
−5	−2	−12	−18.30	13.70
−5	−2	−12	5.50	9.30
5	2	12	−7.50	5.90
−5	2	−12	−6.30	7.70
−5	2	−12	−12.00	6.40
−5	2	−12	18.80	18.40
−5	2	−12	−11.30	7.40
−5	2	−12	4.10	21.20
−5	2	−12	15.80	17.20
−6	−2	−12	59.39	15.30
−6	−2	−12	61.39	24.50
−6	−2	−12	60.89	19.50
−6	−2	−12	50.09	10.60
−6	2	−12	90.59	14.60
−6	2	−12	61.09	24.70
−6	2	−12	102.89	20.50
6	2	12	57.89	10.90
−7	−2	−12	−25.50	21.70
−7	−2	−12	3.00	18.00
−7	−2	−12	−4.40	9.40
−7	2	−12	8.40	12.90
−7	2	−12	−6.90	9.50
−7	2	−12	6.20	19.00
−8	−2	−12	29.80	23.00
−8	−2	−12	11.60	19.70
8	2	12	−3.10	9.20
−8	2	−12	−4.50	21.60
−8	2	−12	−25.90	14.20
−8	−3	12	−25.40	23.80
8	−3	−12	0.00	15.70

8	3	-12	-15.80	12.10
-8	3	12	12.10	22.40
-7	-3	12	8.30	21.90
7	-3	-12	11.90	13.90
7	-3	-12	0.50	11.50
7	3	-12	2.70	10.90
-7	3	12	11.60	19.50
-7	3	12	36.10	18.70
-6	-3	12	4.30	8.60
6	-3	-12	-0.20	10.60
-6	-3	12	23.20	20.80
6	-3	-12	-0.40	13.40
-6	3	12	3.20	18.40
-6	3	12	21.20	19.60
6	3	-12	14.70	10.00
5	-3	-12	8.10	12.80
-5	-3	12	-6.20	17.80
5	-3	-12	4.10	9.20
5	3	-12	-1.10	8.50
-5	3	12	6.30	14.60
-5	3	12	-3.50	16.60
4	-3	-12	23.40	10.40
4	-3	-12	10.60	11.90
-4	-3	12	28.30	17.30
-4	3	12	19.20	18.10
4	3	-12	24.00	10.10
-4	3	12	8.70	14.20
3	-3	-12	0.30	8.40
-3	-3	12	3.60	6.10
-3	-3	12	-5.80	13.40
-3	3	12	21.30	14.50

-3	3	12	0.60	14.40
-3	3	12	9.50	8.90
3	3	-12	19.70	8.70
2	-3	-12	124.49	16.60
-2	-3	12	108.89	22.90
2	-3	-12	100.79	19.10
-2	-3	12	106.69	12.20
-2	3	12	126.29	16.50
2	3	-12	112.59	15.10
-2	3	12	124.09	21.30
2	3	-12	95.29	14.50
2	3	-12	147.09	25.10
-2	3	12	143.99	28.30
-1	-3	12	-0.70	4.40
-1	-3	12	3.20	5.70
1	-3	-12	-0.70	7.80
1	-3	-12	9.40	7.90
-1	-3	12	-0.50	13.50
-1	3	12	6.90	7.10
-1	3	12	7.90	11.80
1	3	-12	8.40	7.80
-1	3	12	-3.50	13.20
1	3	-12	10.10	10.10
0	-3	12	-7.20	11.90
0	-3	12	8.10	4.70
0	-3	-12	0.30	7.30
0	-3	-12	9.20	9.20
0	-3	-12	11.40	13.40
0	-3	12	-0.50	3.70
0	3	12	0.50	11.40
0	3	12	4.60	15.10

0	3	-12	17.20	14.90
0	3	12	-5.80	6.50
0	3	-12	7.20	9.10
0	3	-12	4.30	7.00
-1	-3	-12	28.40	9.20
1	-3	12	27.90	15.60
1	-3	12	38.40	4.60
-1	-3	-12	53.19	21.20
1	-3	12	26.90	4.80
-1	-3	-12	36.30	12.30
-1	3	-12	20.70	18.10
1	3	12	40.00	9.70
1	3	12	19.90	13.50
-1	3	-12	43.40	15.20
-1	3	-12	25.30	9.20
1	3	12	29.50	14.80
2	-3	12	73.89	16.50
-2	-3	-12	51.89	12.20
-2	-3	-12	85.49	21.20
-2	-3	-12	86.29	23.40
-2	-3	-12	41.30	13.30
-2	3	-12	52.79	13.90
-2	3	-12	43.00	11.20
-2	3	-12	30.70	17.00
2	3	12	87.79	13.20
2	3	12	101.29	21.00
-2	3	-12	66.29	13.30
-2	3	-12	37.40	17.10
-3	-3	-12	9.60	9.90
-3	-3	-12	8.00	13.40
-3	-3	-12	2.00	8.60

-3	-3	-12	10.40	13.50
-3	3	-12	15.20	18.10
3	3	12	35.10	16.30
-3	3	-12	29.30	16.60
-3	3	-12	19.50	11.90
-3	3	-12	3.10	7.20
3	3	12	27.70	8.70
-4	-3	-12	28.70	20.50
-4	-3	-12	1.30	9.30
-4	-3	-12	12.20	8.50
-4	-3	-12	-8.30	13.90
-4	3	-12	13.20	9.70
-4	3	-12	-6.30	17.10
4	3	12	3.30	6.40
4	3	12	2.70	13.70
-4	3	-12	9.60	15.20
-4	3	-12	6.50	9.70
-4	3	-12	3.30	8.20
-4	3	-12	0.20	7.40
-5	-3	-12	-14.70	8.10
-5	-3	-12	-4.30	15.10
-5	-3	-12	-13.80	14.90
-5	-3	-12	-8.20	9.30
5	3	12	-0.30	13.60
5	3	12	2.50	7.60
-5	3	-12	6.50	17.40
-5	3	-12	-0.40	6.60
-5	3	-12	5.10	9.80
-5	3	-12	2.60	8.10
-6	-3	-12	-0.50	8.00
-6	-3	-12	12.20	12.20

-6	-3	-12	5.10	18.60
-6	-3	-12	30.80	21.10
-6	3	-12	-20.90	18.30
-6	3	-12	1.90	8.30
-6	3	-12	6.90	11.00
6	3	12	11.50	8.10
-7	-3	-12	-12.00	18.10
-7	-3	-12	-5.60	20.40
-7	-3	-12	0.70	10.70
-7	3	-12	26.20	19.60
-7	3	-12	-1.70	12.50
-7	3	-12	1.80	9.20
7	3	12	-2.40	7.50
-8	-3	-12	-8.20	23.60
-8	-3	-12	-37.50	20.00
-8	3	-12	-27.60	14.00
8	3	12	-10.90	9.80
-8	3	-12	-24.80	23.60
8	-4	-12	-23.90	13.00
-8	-4	12	0.00	21.20
8	4	-12	10.70	11.50
-8	4	12	40.60	26.10
-8	4	12	-3.70	22.90
7	-4	-12	24.20	13.50
7	-4	-12	-2.90	14.40
-7	-4	12	-26.80	19.50
-7	4	12	7.40	20.60
7	4	-12	-1.70	11.80
-7	4	12	-14.20	18.80
6	-4	-12	-10.10	14.80
6	-4	-12	22.60	12.10

-6	-4	12	24.70	20.10
6	4	-12	28.70	10.90
-6	4	12	-16.40	16.30
-6	4	12	10.70	21.40
5	-4	-12	-15.20	10.50
5	-4	-12	0.10	10.00
-5	-4	12	-9.10	15.50
-5	4	12	1.30	15.20
-5	4	12	-3.00	16.30
5	4	-12	-3.80	7.90
4	-4	-12	-1.10	11.90
-4	-4	12	20.10	15.70
4	-4	-12	2.30	8.50
-4	4	12	-17.10	13.80
4	4	-12	7.90	8.80
-4	4	12	-17.10	13.40
-3	-4	12	9.40	6.50
3	-4	-12	4.00	8.70
3	-4	-12	2.00	10.80
-3	4	12	11.40	9.20
-3	4	12	12.60	13.70
3	4	-12	-5.20	7.20
-3	4	12	-10.90	14.20
2	-4	-12	5.20	9.40
-2	-4	12	-8.20	14.20
-2	-4	12	6.40	5.50
-2	-4	12	2.60	5.10
2	4	-12	13.60	8.50
2	4	-12	8.90	15.30
-2	4	12	4.10	16.50
-2	4	12	-0.40	12.80

-2	4	12	-0.30	6.60
-1	-4	12	-7.70	11.70
-1	-4	12	-3.60	3.30
1	-4	-12	3.10	7.70
-1	-4	12	4.80	4.30
1	-4	-12	-11.70	8.30
-1	4	12	-8.50	14.30
-1	4	12	-4.60	9.70
1	4	-12	26.00	13.80
1	4	-12	-23.00	8.60
1	4	-12	-0.70	6.80
-1	4	12	-12.10	6.20
0	-4	12	7.60	13.80
0	-4	12	1.40	2.30
0	-4	-12	17.90	9.10
0	-4	-12	3.00	9.70
0	-4	-12	0.90	14.30
0	4	-12	7.90	13.30
0	4	12	16.80	12.20
0	4	12	0.80	7.30
0	4	12	12.10	13.80
0	4	-12	17.70	9.80
0	4	-12	0.10	6.90
-1	-4	-12	-1.70	8.80
-1	-4	-12	9.60	14.30
-1	-4	-12	13.10	10.80
1	-4	12	3.70	13.90
-1	4	-12	-16.20	11.80
-1	4	-12	-9.30	9.60
-1	4	-12	7.10	7.00
1	4	12	13.00	14.30

1	4	12	6.50	8.00
1	4	12	-2.50	12.00
-1	4	-12	5.80	13.10
-2	-4	-12	22.60	17.60
2	-4	12	48.00	12.50
-2	-4	-12	54.39	14.20
-2	-4	-12	31.40	17.20
-2	4	-12	46.80	12.70
2	4	12	21.70	9.20
2	4	12	33.70	17.10
-2	4	-12	41.00	14.70
-2	4	-12	34.00	10.40
-3	-4	-12	11.20	16.30
-3	-4	-12	13.60	9.70
-3	-4	-12	12.20	14.90
-3	-4	-12	4.40	10.00
3	4	12	6.70	14.60
-3	4	-12	8.30	10.20
-3	4	-12	-5.80	8.30
-3	4	-12	20.80	8.90
-3	4	-12	-5.30	14.30
-3	4	-12	-10.50	12.70
3	4	12	7.10	7.80
-4	-4	-12	-0.90	17.70
-4	-4	-12	-6.80	8.20
-4	-4	-12	-10.90	8.60
-4	-4	-12	-1.20	15.50
-4	4	-12	-5.10	8.60
4	4	12	-4.80	11.30
4	4	12	-5.90	7.80
-4	4	-12	5.80	8.20

-4	4	-12	-16.10	7.40
-4	4	-12	-14.80	13.70
-4	4	-12	-4.10	8.20
-4	4	-12	-4.80	14.50
-5	-4	-12	-16.40	16.60
-5	-4	-12	-5.90	9.00
-5	-4	-12	4.70	9.50
-5	-4	-12	1.40	18.10
5	4	12	19.10	15.90
-5	4	-12	12.50	7.20
-5	4	-12	14.40	10.70
-5	4	-12	47.90	18.60
5	4	12	9.20	7.90
-5	4	-12	13.50	8.00
-6	-4	-12	-10.30	10.30
-6	-4	-12	28.90	20.10
-6	-4	-12	1.50	9.70
-6	-4	-12	0.90	17.30
-6	4	-12	-3.70	7.60
-6	4	-12	3.00	17.80
6	4	12	13.30	8.60
-7	-4	-12	-3.70	20.60
-7	-4	-12	29.90	25.50
-7	-4	-12	8.90	9.40
-7	4	-12	-8.00	9.60
-7	4	-12	7.10	21.40
7	4	12	-3.50	9.00
-7	4	-12	-7.20	11.90
-8	-4	-12	-0.90	24.70
-8	-4	-12	-32.20	23.60
-8	4	-12	2.80	13.80

-8	4	-12	28.30	23.40
8	4	12	-18.70	10.20
-8	-5	12	17.40	28.90
8	-5	-12	-11.50	15.10
8	-5	-12	-16.10	13.90
8	5	-12	-9.40	11.10
-8	5	12	21.90	24.20
-8	5	12	-5.10	23.40
-7	-5	12	2.90	22.80
7	-5	-12	-7.60	12.20
7	-5	-12	9.50	16.40
7	5	-12	-10.70	10.00
-7	5	12	-8.90	19.60
-7	5	12	-16.20	19.50
6	-5	-12	26.10	16.40
6	-5	-12	6.10	12.00
-6	-5	12	-0.10	20.00
-6	5	12	-16.60	16.90
6	5	-12	7.30	11.40
-6	5	12	-5.80	16.50
5	-5	-12	24.10	15.10
5	-5	-12	-7.00	8.80
-5	-5	12	-7.50	18.40
5	5	-12	18.10	9.30
-5	5	12	11.30	16.80
-5	5	12	9.80	16.30
4	-5	-12	-0.50	8.80
-4	-5	12	2.80	6.80
-4	-5	12	-9.00	17.40
4	-5	-12	3.60	13.00
-4	5	12	-1.10	8.80

-4	5	12	-18.60	14.70
4	5	-12	-4.00	8.50
-4	5	12	6.40	14.00
-3	-5	12	5.80	7.10
3	-5	-12	-3.60	11.10
-3	-5	12	8.40	16.80
3	-5	-12	21.20	10.50
-3	-5	12	15.20	7.90
-3	5	12	14.10	16.50
-3	5	12	2.20	8.20
3	5	-12	28.80	18.90
-3	5	12	-10.70	13.60
3	5	-12	2.10	6.80
2	-5	-12	26.30	9.90
-2	-5	12	19.30	5.50
2	-5	-12	-12.60	10.90
-2	-5	12	14.80	4.70
-2	-5	12	27.30	14.20
-2	5	12	13.40	9.20
2	5	-12	23.90	15.80
-2	5	12	5.00	15.30
-2	5	12	21.60	16.30
2	5	-12	11.90	8.10
1	-5	-12	12.60	8.90
-1	-5	12	-6.00	13.00
1	-5	-12	5.40	12.80
1	5	-12	-6.10	6.50
-1	5	12	-17.30	14.20
-1	5	12	6.30	8.30
-1	5	12	-7.50	11.60
1	5	-12	2.70	13.50

0	-5	-12	16.40	10.30
0	-5	-12	6.00	8.90
0	-5	-12	1.50	15.50
0	-5	12	-10.70	12.00
0	5	12	0.60	16.10
0	5	-12	6.10	7.30
0	5	12	2.90	11.70
0	5	-12	15.60	12.50
0	5	-12	9.60	8.20
-1	-5	-12	-15.70	12.80
-1	-5	-12	7.90	9.50
-1	-5	-12	-3.00	7.40
1	-5	12	13.30	16.40
-1	-5	-12	-1.00	14.80
-1	5	-12	2.90	8.00
-1	5	-12	12.10	7.90
1	5	12	-14.30	11.10
1	5	12	12.90	14.80
-1	5	-12	0.60	9.40
-1	5	-12	22.10	11.50
-2	-5	-12	-2.70	8.40
-2	-5	-12	3.30	11.20
-2	-5	-12	11.80	14.30
-2	-5	-12	13.20	18.80
-2	5	-12	11.40	7.50
2	5	12	5.40	15.00
-2	5	-12	14.10	8.30
2	5	12	7.70	9.00
-2	5	-12	31.10	16.10
-2	5	-12	16.40	11.50
-3	-5	-12	-5.40	9.30

-3	-5	-12	-12.50	14.80
-3	-5	-12	-0.40	9.30
-3	-5	-12	-8.60	13.40
3	5	12	22.00	16.10
-3	5	-12	6.40	17.70
-3	5	-12	-0.40	6.40
3	5	12	0.80	7.20
-3	5	-12	4.40	10.90
-3	5	-12	-7.60	7.60
-4	-5	-12	22.50	12.20
-4	-5	-12	22.10	9.20
-4	-5	-12	26.90	18.80
-4	-5	-12	-21.60	15.70
-4	5	-12	7.30	15.80
-4	5	-12	0.40	8.60
4	5	12	2.40	13.00
-4	5	-12	-9.40	11.10
-4	5	-12	0.60	7.50
4	5	12	-6.90	7.10
-4	5	-12	4.70	8.70
-5	-5	-12	8.20	10.70
-5	-5	-12	13.10	16.10
-5	-5	-12	-3.40	10.00
-5	-5	-12	-7.20	17.70
5	5	12	28.30	17.70
-5	5	-12	26.60	17.30
-5	5	-12	2.10	7.70
-5	5	-12	-2.30	8.10
5	5	12	0.90	7.80
-5	5	-12	-12.00	8.00
-6	-5	-12	-22.80	18.00

−6	−5	−12	23.10	11.20
−6	−5	−12	0.10	20.10
−6	−5	−12	13.20	11.10
6	5	12	10.90	16.40
−6	5	−12	−1.30	19.60
6	5	12	7.20	9.40
−6	5	−12	−7.40	8.20
−6	5	−12	5.80	10.10
−7	−5	−12	0.40	21.00
−7	−5	−12	−4.00	10.40
−7	−5	−12	12.40	24.60
−7	5	−12	4.00	18.10
−7	5	−12	19.20	28.40
7	5	12	2.90	10.20
−7	−6	12	−9.70	22.60
7	−6	−12	13.60	14.10
7	−6	−12	−4.90	19.10
7	6	−12	−7.90	11.10
−7	6	12	20.50	22.40
−7	6	12	1.00	22.40
−6	−6	12	36.40	23.40
6	−6	−12	4.20	12.90
6	−6	−12	−0.80	15.50
6	6	−12	24.10	11.10
−6	6	12	−1.50	20.60
−6	6	12	6.40	19.40
−5	−6	12	−5.60	18.10
5	−6	−12	−27.10	14.80
5	−6	−12	5.80	11.00
5	6	−12	−6.30	8.70
−5	6	12	29.80	19.50

-5	6	12	-31.70	16.70
-4	-6	12	3.20	17.40
4	-6	-12	26.20	10.40
4	-6	-12	11.90	14.00
-4	-6	12	7.30	7.80
-4	6	12	-4.80	9.10
-4	6	12	9.90	17.40
-4	6	12	4.90	17.90
3	-6	-12	10.30	12.10
-3	-6	12	18.80	18.40
3	-6	-12	-0.40	8.40
-3	-6	12	5.40	5.30
3	6	-12	14.30	18.70
-3	6	12	9.80	14.70
3	6	-12	-2.90	7.00
-3	6	12	6.30	9.90
-3	6	12	-5.50	15.00
2	-6	-12	19.90	13.40
2	-6	-12	19.50	10.50
-2	-6	12	10.40	15.30
2	6	-12	24.20	15.70
-2	6	12	16.60	10.30
-2	6	12	28.30	19.50
2	6	-12	23.40	8.70
-2	6	12	35.20	18.70
-1	-6	12	8.60	13.80
1	-6	-12	-8.00	8.50
1	-6	-12	-2.30	9.40
1	-6	-12	14.70	13.40
-1	6	12	-10.10	8.00
-1	6	12	8.20	16.50

1	6	-12	6.50	11.50
1	6	-12	2.20	7.30
0	-6	-12	3.60	11.80
0	-6	-12	1.90	6.90
0	-6	-12	6.50	14.30
0	6	-12	-6.70	7.50
0	6	12	-2.40	6.90
0	6	12	-9.60	14.80
0	6	-12	-0.30	10.60
0	6	-12	7.20	7.90
-1	-6	-12	5.40	12.30
-1	-6	-12	6.60	15.70
1	-6	12	11.00	12.30
-1	-6	-12	6.00	8.50
-1	-6	-12	16.00	18.10
1	6	12	5.20	8.70
-1	6	-12	13.30	15.30
-1	6	-12	-3.00	7.10
-1	6	-12	9.70	10.60
1	6	12	-14.30	11.50
-1	6	-12	11.10	7.70
-2	-6	-12	8.10	9.80
-2	-6	-12	10.20	16.40
-2	-6	-12	9.20	10.00
-2	-6	-12	22.30	17.30
-2	6	-12	12.60	8.60
2	6	12	10.60	9.10
-2	6	-12	30.20	17.50
2	6	12	17.20	13.40
-2	6	-12	19.50	7.80
-3	-6	-12	9.60	13.00

−3	−6	−12	9.30	21.00
−3	−6	−12	−12.60	8.10
−3	−6	−12	−2.40	15.40
3	6	12	12.30	9.80
−3	6	−12	23.90	7.60
3	6	12	12.90	15.80
−3	6	−12	8.50	6.30
−3	6	−12	9.40	15.60
−4	−6	−12	3.20	10.30
−4	−6	−12	13.90	20.80
−4	−6	−12	11.50	10.20
−4	6	−12	16.60	6.90
4	6	12	3.60	13.10
−4	6	−12	−3.10	7.10
−4	6	−12	−5.30	5.30
−4	6	−12	15.60	20.00
4	6	12	−0.80	7.60
−5	−6	−12	−2.70	11.50
−5	−6	−12	−10.60	10.00
−5	−6	−12	11.10	21.00
−5	−6	−12	5.90	19.40
−5	6	−12	−13.10	9.10
−5	6	−12	−13.30	17.90
5	6	12	−5.00	17.00
5	6	12	−3.00	9.00
−5	6	−12	−19.50	7.80
−6	−6	−12	20.40	12.40
−6	−6	−12	15.80	22.00
−6	−6	−12	1.30	12.40
−6	−6	−12	14.80	22.50
−6	6	−12	−0.30	8.60

6	6	12	-8.30	15.50
6	6	12	-8.80	8.10
-6	6	-12	2.20	20.40
-7	-6	-12	-18.60	21.80
-7	-6	-12	20.50	13.40
-7	-6	-12	2.70	24.70
-7	6	-12	-19.80	23.50
7	6	12	-4.60	11.60
7	-7	-12	1.10	18.80
-7	-7	12	32.80	26.30
7	-7	-12	2.90	13.10
-7	7	12	-29.80	24.30
-7	7	12	-20.20	24.00
6	-7	-12	25.50	13.80
-6	-7	12	20.70	23.30
6	-7	-12	10.40	17.90
6	7	-12	2.80	9.70
-6	7	12	-0.10	22.70
-6	7	12	-1.60	19.60
5	-7	-12	4.10	10.70
-5	-7	12	-7.00	19.80
-5	-7	12	7.30	9.00
5	-7	-12	-7.90	13.50
-5	7	12	9.20	11.20
5	7	-12	0.50	9.80
-5	7	12	17.00	22.20
-5	7	12	-10.20	17.70
-4	-7	12	26.20	20.30
-4	-7	12	24.10	7.50
4	-7	-12	10.80	10.00
4	-7	-12	25.30	15.00

-4	7	12	11.60	9.90
-4	7	12	-16.60	16.60
-4	7	12	-29.80	15.60
4	7	-12	-2.10	9.50
3	-7	-12	-1.20	9.60
-3	-7	12	17.10	18.40
3	-7	-12	2.00	12.00
-3	7	12	14.90	17.30
3	7	-12	1.50	8.20
-3	7	12	-13.10	17.00
-3	7	12	-13.40	9.70
2	-7	-12	4.70	8.30
2	-7	-12	13.70	12.80
-2	-7	12	1.50	16.10
-2	7	12	5.70	16.60
-2	7	12	-4.20	9.00
2	7	-12	7.20	7.90
2	7	-12	-9.00	15.90
-2	7	12	-9.60	15.70
-1	-7	12	-13.60	14.90
1	-7	-12	3.40	11.40
1	-7	-12	14.40	16.40
1	-7	-12	-0.30	8.10
-1	7	12	13.20	14.80
-1	7	12	-15.80	18.10
1	7	-12	24.50	12.80
1	7	-12	11.10	7.80
-1	7	12	-1.80	8.70
0	-7	-12	-5.60	13.40
0	-7	-12	1.60	13.30
0	-7	-12	-2.20	8.10

0	-7	12	-10.60	13.80
0	7	-12	1.20	10.20
0	7	12	-12.50	14.70
0	7	12	-5.50	8.90
0	7	12	3.40	14.20
0	7	-12	0.80	7.20
-1	-7	-12	20.90	10.90
-1	-7	-12	23.90	12.30
-1	-7	-12	18.90	19.50
-1	-7	-12	0.20	14.80
-1	7	-12	11.60	19.00
1	7	12	19.60	16.00
-1	7	-12	1.90	6.60
-1	7	-12	20.80	9.20
1	7	12	-1.20	8.40
-2	-7	-12	1.60	10.20
-2	-7	-12	0.20	12.20
-2	-7	-12	-16.10	14.80
-2	-7	-12	1.10	17.60
-2	7	-12	-1.30	12.00
-2	7	-12	-6.20	14.30
-2	7	-12	-14.10	7.20
2	7	12	-3.50	8.60
2	7	12	15.60	16.30
-3	-7	-12	-9.30	19.80
-3	-7	-12	28.70	19.40
-3	-7	-12	7.60	9.40
-3	-7	-12	13.20	10.70
-3	7	-12	-14.40	7.60
3	7	12	-4.40	14.10
-3	7	-12	5.70	18.50

3	7	12	7.90	8.50
-4	-7	-12	6.30	12.30
-4	-7	-12	0.90	11.10
-4	-7	-12	2.00	19.20
4	7	12	27.10	12.30
4	7	12	2.60	17.30
-4	7	-12	10.80	8.60
-4	7	-12	-18.00	17.50
-5	-7	-12	2.80	12.30
-5	-7	-12	7.20	12.90
-5	-7	-12	4.10	20.90
-5	-7	-12	-3.30	20.70
-5	7	-12	23.80	8.90
5	7	12	3.10	10.30
5	7	12	17.60	19.10
-5	7	-12	10.80	21.50
-6	-7	-12	15.80	13.90
-6	-7	-12	-23.60	20.10
-6	-7	-12	5.60	48.10
6	7	12	26.30	11.60
-6	7	-12	-12.60	8.50
6	7	12	-43.40	16.00
-6	7	-12	10.90	21.90
6	-8	-12	-0.40	16.20
-6	-8	12	-9.50	28.10
6	-8	-12	15.10	13.80
6	8	-12	-13.60	11.30
-6	8	12	-1.00	23.40
-6	8	12	-32.20	21.40
5	-8	-12	-1.60	14.00
-5	-8	12	-13.60	26.90

5	-8	-12	12.40	11.30
-5	-8	12	4.00	12.80
-5	8	12	25.20	21.40
5	8	-12	-0.10	10.00
-5	8	12	-3.20	11.00
-5	8	12	14.60	21.10
4	-8	-12	28.80	12.10
-4	-8	12	9.60	23.60
4	-8	-12	-16.60	13.80
-4	8	12	-8.90	19.20
-4	8	12	13.60	11.70
-4	8	12	10.70	17.00
4	8	-12	11.10	9.80
3	-8	-12	11.00	10.20
3	-8	-12	16.90	14.20
-3	-8	12	29.50	23.40
-3	8	12	3.80	20.60
-3	8	12	-9.70	8.00
-3	8	12	6.90	16.30
3	8	-12	3.20	9.00
2	-8	-12	24.80	15.90
2	-8	-12	-4.50	9.10
-2	-8	12	27.40	19.70
2	-8	-12	13.80	12.70
2	8	-12	8.90	8.40
-2	8	12	13.50	18.50
-2	8	12	16.90	11.30
-2	8	12	-7.00	15.70
1	-8	-12	33.40	17.30
-1	-8	12	-5.00	15.50
1	-8	-12	5.40	11.60

1	-8	-12	-5.70	9.70
-1	8	12	-8.70	9.00
-1	8	12	5.40	16.70
1	8	-12	0.80	7.50
-1	8	12	11.40	17.60
1	8	-12	-20.60	10.10
0	-8	-12	59.49	13.30
0	-8	-12	78.79	25.60
0	-8	-12	26.10	14.00
0	-8	-12	93.29	23.20
0	8	12	64.29	24.30
0	8	-12	47.90	9.90
0	8	12	71.59	23.10
0	8	12	40.90	13.30
-1	-8	-12	0.70	9.00
-1	-8	-12	24.40	21.10
-1	-8	-12	-3.20	13.30
-1	-8	-12	1.40	16.60
1	8	12	23.90	19.20
1	8	12	1.40	10.20
-1	8	-12	-1.10	7.80
-1	8	-12	-0.70	11.00
-1	8	-12	-19.20	15.30
-2	-8	-12	12.70	11.10
-2	-8	-12	2.70	15.00
-2	-8	-12	-17.70	19.90
-2	-8	-12	-18.10	12.40
-2	8	-12	-1.50	7.60
-2	8	-12	17.80	14.40
-2	8	-12	26.00	13.70
2	8	12	8.90	11.80

2	8	12	-6.70	15.40
-3	-8	-12	13.30	18.20
-3	-8	-12	10.10	24.00
-3	-8	-12	11.20	11.90
-3	-8	-12	-0.70	11.60
-3	8	-12	1.20	7.70
-3	8	-12	3.80	16.10
-3	8	-12	12.90	20.60
3	8	12	7.70	16.00
3	8	12	14.70	10.40
-4	-8	-12	4.10	23.50
-4	-8	-12	-20.20	9.20
-4	-8	-12	16.50	13.00
-4	8	-12	-10.20	20.60
4	8	12	1.60	10.40
4	8	12	5.50	16.80
-4	8	-12	-1.70	8.10
-4	8	-12	43.10	18.90
-5	-8	-12	27.10	21.70
-5	-8	-12	-19.50	25.50
-5	-8	-12	-2.40	14.20
-5	8	-12	4.00	21.90
5	8	12	3.20	10.50
-5	8	-12	30.50	20.50
-6	-8	-12	-45.00	25.50
-6	-8	-12	-3.20	14.00
-6	-8	-12	-34.60	21.40
-6	8	-12	8.30	21.40
6	8	12	24.20	21.50
-6	8	-12	33.40	23.90
6	8	12	-5.40	12.40

5	-9	-12	9.90	16.90
5	-9	-12	7.30	13.20
-5	9	12	6.40	21.70
5	9	-12	-6.20	10.60
-5	9	12	14.50	26.10
-5	9	12	5.50	13.40
4	-9	-12	38.10	13.50
4	-9	-12	19.70	15.50
-4	9	12	18.10	12.20
4	9	-12	-1.30	10.20
-4	9	12	33.60	22.50
-4	9	12	7.40	19.80
3	-9	-12	-9.20	9.80
3	-9	-12	39.60	18.80
3	-9	-12	2.10	13.10
-3	9	12	-4.90	9.60
-3	9	12	35.80	21.10
-3	9	12	16.40	20.60
3	9	-12	3.50	8.90
2	-9	-12	27.80	19.70
2	-9	-12	5.30	10.40
2	-9	-12	17.20	14.20
-2	9	12	-48.90	18.10
-2	9	12	26.70	21.80
2	9	-12	7.60	8.80
-2	9	12	6.60	9.30
1	-9	-12	1.00	16.60
1	-9	-12	-7.10	9.00
1	-9	-12	9.90	15.90
-1	9	12	8.00	18.40
1	9	-12	1.90	8.20

-1	9	12	19.50	14.00
-1	9	12	-14.00	16.90
0	-9	-12	-0.60	22.90
0	-9	-12	-0.80	10.50
0	-9	-12	12.80	15.40
0	-9	-12	-11.50	16.90
0	9	12	6.00	11.00
0	9	-12	-0.40	7.80
0	9	12	-12.20	17.20
-1	-9	-12	10.40	23.20
-1	-9	-12	-4.00	12.80
-1	-9	-12	-0.30	17.60
-1	-9	-12	4.50	10.70
-1	9	-12	14.20	19.00
1	9	12	3.30	19.90
-1	9	-12	22.00	8.40
1	9	12	-13.50	10.50
-2	-9	-12	22.20	15.00
-2	-9	-12	23.50	12.20
-2	-9	-12	49.60	27.10
-2	-9	-12	9.00	16.60
2	9	12	17.80	13.40
-2	9	-12	6.70	8.70
-2	9	-12	-24.80	17.50
2	9	12	2.10	18.00
-2	9	-12	37.10	15.30
-3	-9	-12	-4.00	22.70
-3	-9	-12	-18.40	13.20
-3	-9	-12	25.80	14.50
-3	-9	-12	-38.00	17.50
-3	9	-12	1.20	16.50

3	9	12	11.90	12.10
-3	9	-12	-1.90	17.20
3	9	12	-1.80	18.30
-3	9	-12	1.60	9.10
-4	-9	-12	9.70	15.70
-4	-9	-12	31.70	16.30
-4	-9	-12	-15.50	25.00
-4	9	-12	10.30	19.90
-4	9	-12	42.80	22.20
4	9	12	52.49	17.10
-5	-9	-12	-40.60	21.00
-5	-9	-12	4.80	28.80
-5	-9	-12	10.70	15.20
-5	9	-12	-34.30	20.50
5	9	12	-26.30	11.40
-5	9	-12	-27.00	23.10
5	9	12	-8.30	22.50
4	-10	-12	8.20	13.10
4	-10	-12	-16.00	13.50
-4	10	12	-7.80	11.70
-4	10	12	-13.60	24.30
4	10	-12	-5.00	9.50
-4	10	12	33.10	22.50
3	-10	-12	5.80	10.80
-3	10	12	25.90	22.20
-3	10	12	17.70	14.10
2	-10	-12	6.90	15.00
2	-10	-12	-1.60	11.00
-2	10	12	-8.10	21.30
2	10	-12	4.30	9.40
-2	10	12	-0.30	11.50

-2	10	12	-28.10	21.00
1	-10	-12	17.50	27.50
1	-10	-12	5.80	12.80
1	-10	-12	-41.60	21.30
1	-10	-12	0.10	10.50
-1	10	12	0.70	23.00
1	10	-12	9.90	8.40
-1	10	12	-6.50	12.10
0	-10	-12	19.30	12.00
0	-10	-12	12.80	18.70
0	-10	-12	31.20	15.40
0	-10	-12	30.10	28.30
0	10	-12	12.70	8.80
0	10	12	11.90	21.40
0	10	-12	10.30	19.30
0	10	12	12.40	14.30
-1	-10	-12	4.40	11.50
-1	-10	-12	1.30	25.00
-1	-10	-12	22.30	15.90
-1	-10	-12	34.20	21.00
-1	10	-12	-27.70	15.40
1	10	12	-14.60	18.20
1	10	12	21.10	13.90
-1	10	-12	-2.00	8.40
-2	-10	-12	38.20	26.40
-2	-10	-12	-13.70	18.80
-2	-10	-12	-12.30	14.10
-2	-10	-12	-0.70	12.10
2	10	12	-10.90	12.30
-2	10	-12	18.90	21.40
2	10	12	-10.60	20.70

-3	-10	-12	44.80	21.50
-3	-10	-12	-10.60	15.20
-3	-10	-12	-2.80	12.20
-3	-10	-12	-17.20	28.30
-3	10	-12	6.80	22.90
-3	10	-12	15.70	20.40
3	10	12	8.50	14.50
3	10	12	5.50	20.70
-4	-10	-12	-17.20	14.00
-4	-10	-12	-56.69	21.10
-4	-10	-12	-16.20	14.20
-4	10	-12	15.60	21.60
4	10	12	0.40	14.30
4	10	12	-12.30	23.10
-4	10	-12	11.60	23.30
3	-11	-12	11.70	13.20
3	-11	-12	7.30	17.30
-3	11	12	-8.40	13.90
-3	11	12	19.10	23.80
-3	11	12	9.10	22.90
3	11	-12	-26.10	9.90
2	-11	-12	11.10	16.70
2	-11	-12	-15.50	11.50
-2	11	12	-1.00	11.40
-2	11	12	-22.70	21.20
-2	11	12	-36.90	21.30
2	11	-12	6.80	10.30
1	-11	-12	8.10	12.90
1	-11	-12	101.59	29.70
1	-11	-12	-4.20	16.40
-1	11	12	9.90	13.30

1	11	-12	-12.90	8.50
-1	11	12	-8.10	22.30
0	-11	-12	-9.40	12.30
0	-11	-12	-46.80	26.60
0	11	-12	-9.20	21.00
0	11	12	2.90	13.30
0	11	12	-6.90	22.80
-1	-11	-12	26.30	27.40
-1	-11	-12	24.50	12.80
1	11	12	12.90	22.20
-1	11	-12	-18.60	21.50
1	11	12	11.60	13.80
-2	-11	-12	10.40	27.80
2	11	12	7.10	14.30
2	11	12	-12.20	22.10
-2	11	-12	-0.50	23.30
-3	-11	-12	-4.10	35.20
-3	11	-12	3.20	23.00
3	11	12	7.10	24.90
3	11	12	0.70	13.50
8	0	-13	-6.40	13.40
8	0	-13	20.50	21.70
-8	0	13	-32.60	23.20
-8	0	13	-11.40	23.60
7	0	-13	20.80	15.20
7	0	-13	-1.10	13.10
-7	0	13	26.00	24.00
-7	0	13	37.10	19.00
-6	0	13	-10.40	14.30
6	0	-13	0.80	10.60
6	0	-13	9.80	15.00

-6	0	13	33.00	19.80
5	0	-13	88.39	16.10
5	0	-13	70.69	17.00
-5	0	13	107.19	20.10
-4	0	13	17.30	12.80
-4	0	13	4.50	16.50
4	0	-13	-5.40	7.40
-4	0	13	8.40	8.50
4	0	-13	3.20	11.20
-3	0	13	19.90	8.00
3	0	-13	28.10	11.80
3	0	-13	11.80	12.50
-3	0	13	34.70	20.50
-3	0	13	15.80	11.40
2	0	-13	-2.40	8.60
2	0	-13	12.60	11.80
-2	0	13	-7.70	15.70
-2	0	13	-1.00	6.70
-2	0	13	4.60	10.00
-1	0	13	12.80	7.10
1	0	-13	6.30	8.90
1	0	-13	22.20	12.00
-1	0	13	21.50	11.10
-1	0	13	15.60	16.00
0	0	13	1.40	6.20
0	0	-13	2.50	7.80
0	0	-13	4.90	11.00
0	0	13	18.20	15.10
0	0	13	9.40	10.70
-1	0	-13	0.00	8.30
1	0	13	-0.10	10.60

1	0	13	2.80	6.00
-1	0	-13	5.10	14.10
-1	0	-13	-17.10	8.90
1	0	13	5.20	16.70
-2	0	-13	11.40	9.80
2	0	13	-0.10	5.70
2	0	13	6.20	13.90
-2	0	-13	1.90	7.20
-2	0	-13	5.50	16.20
2	0	13	3.40	11.60
-3	0	-13	53.99	12.70
-3	0	-13	31.70	16.30
-3	0	-13	49.60	16.20
-3	0	-13	32.50	12.30
3	0	13	39.70	8.10
-4	0	-13	-9.90	12.40
-4	0	-13	12.30	9.90
-4	0	-13	-8.00	15.30
4	0	13	-0.30	5.50
-4	0	-13	2.00	10.10
-5	0	-13	99.19	22.10
-5	0	-13	40.00	11.00
5	0	13	44.50	8.40
-5	0	-13	43.60	11.90
-5	0	-13	86.79	27.70
-6	0	-13	-11.20	17.30
6	0	13	-11.40	6.20
-6	0	-13	-6.00	9.60
-6	0	-13	0.80	15.30
-7	0	-13	8.40	18.00
-7	0	-13	0.60	11.60

7	0	13	0.00	8.20
8	-1	-13	-4.60	13.50
-8	-1	13	7.90	26.60
8	-1	-13	1.00	17.20
8	1	-13	-21.10	14.00
8	1	-13	2.40	17.20
7	-1	-13	9.50	16.70
-7	-1	13	5.90	21.40
-7	-1	13	-5.10	18.40
7	-1	-13	10.20	13.60
-7	1	13	-8.20	21.20
7	1	-13	-6.90	10.60
7	1	-13	-18.50	14.00
-7	1	13	-4.40	18.70
-6	-1	13	36.40	16.90
6	-1	-13	10.20	11.00
6	-1	-13	-6.10	13.70
-6	-1	13	27.90	22.80
-6	1	13	-5.10	15.70
6	1	-13	-7.00	12.20
-6	1	13	-3.30	19.30
6	1	-13	-0.10	10.30
5	-1	-13	26.40	15.00
5	-1	-13	8.60	10.00
-5	-1	13	32.40	17.20
-5	-1	13	14.80	12.10
5	1	-13	19.20	11.30
5	1	-13	9.10	14.50
-5	1	13	31.20	16.70
-5	1	13	22.40	19.80
-4	-1	13	-14.80	15.60

4	-1	-13	7.40	11.00
4	-1	-13	2.00	8.50
-4	-1	13	15.80	8.60
-4	-1	13	-4.20	10.10
-4	1	13	1.20	8.00
4	1	-13	-10.20	10.50
4	1	-13	3.90	9.70
-4	1	13	17.70	16.10
-4	1	13	-11.70	12.70
3	-1	-13	24.80	12.80
-3	-1	13	22.60	8.70
-3	-1	13	7.70	10.60
-3	-1	13	-2.70	15.60
3	-1	-13	33.70	11.80
-3	1	13	10.80	16.90
-3	1	13	26.40	9.50
3	1	-13	24.00	13.90
-3	1	13	25.10	13.80
2	-1	-13	2.60	11.00
-2	-1	13	-6.90	14.90
-2	-1	13	5.20	8.40
2	-1	-13	-5.80	15.30
-2	-1	13	9.40	6.80
-2	1	13	-2.40	11.20
2	1	-13	-3.40	9.50
-2	1	13	5.10	16.50
-2	1	13	8.80	7.60
2	1	-13	-2.30	7.20
-1	-1	13	284.57	38.30
-1	-1	13	289.27	23.30
1	-1	-13	268.07	22.30

-1	-1	13	246.48	16.70
1	-1	-13	309.87	28.20
-1	1	13	259.17	18.70
1	1	-13	323.97	27.00
-1	1	13	265.27	27.40
1	1	-13	277.47	22.10
-1	1	13	199.08	34.20
0	-1	-13	-8.30	10.40
0	-1	-13	-3.70	8.00
0	-1	13	0.00	5.70
0	-1	13	-10.90	14.30
0	-1	13	5.60	9.30
0	1	13	4.90	7.10
0	1	13	-23.50	13.20
0	1	13	3.70	10.40
0	1	-13	5.40	8.40
0	1	-13	4.60	9.90
-1	-1	-13	142.49	18.80
-1	-1	-13	171.08	32.10
1	-1	13	177.38	23.30
1	-1	13	162.88	30.90
1	-1	13	180.48	13.40
-1	-1	-13	228.68	23.50
-1	1	-13	154.08	22.00
-1	1	-13	161.28	19.10
1	1	13	184.68	26.30
1	1	13	137.99	27.80
-1	1	-13	154.18	28.10
1	1	13	157.08	14.60
-2	-1	-13	-11.40	11.70
-2	-1	-13	-17.60	8.40

2	-1	13	10.00	5.40
2	-1	13	15.00	13.40
2	-1	13	-2.70	8.60
-2	-1	-13	-7.00	8.90
2	1	13	2.90	15.60
-2	1	-13	4.80	16.30
-2	1	-13	-18.30	8.20
-2	1	-13	-38.90	18.80
2	1	13	9.20	7.10
-2	1	-13	6.30	8.70
-2	1	-13	18.50	12.60
2	1	13	-2.90	11.00
-3	-1	-13	49.70	20.90
-3	-1	-13	21.20	12.00
3	-1	13	39.40	9.20
-3	-1	-13	38.90	10.90
-3	-1	-13	35.10	19.10
-3	1	-13	33.40	12.00
3	1	13	23.80	8.30
-3	1	-13	43.80	17.00
-3	1	-13	54.49	22.70
-3	1	-13	13.20	16.30
3	1	13	11.40	11.50
-3	1	-13	35.10	10.60
-4	-1	-13	-14.20	9.20
-4	-1	-13	-0.60	15.70
-4	-1	-13	-13.10	8.80
-4	-1	-13	-7.20	13.90
4	-1	13	-0.40	5.00
-4	1	-13	-10.30	15.10
-4	1	-13	11.50	9.00

-4	1	-13	-1.00	13.90
4	1	13	-0.10	6.00
-4	1	-13	-9.70	27.40
5	-1	13	7.40	5.40
-5	-1	-13	16.70	10.50
-5	-1	-13	7.40	16.90
-5	-1	-13	11.60	15.50
-5	1	-13	26.30	20.60
5	1	13	22.40	7.90
-5	1	-13	20.30	14.60
-5	1	-13	30.20	10.10
6	-1	13	1.90	5.60
-6	-1	-13	0.70	16.80
-6	-1	-13	-11.40	7.70
-6	-1	-13	-2.10	8.30
-6	-1	-13	6.70	23.40
6	1	13	-2.70	7.00
-6	1	-13	-8.90	18.90
-6	1	-13	-10.90	13.20
-6	1	-13	12.70	11.80
-7	-1	-13	11.50	11.90
-7	-1	-13	-4.50	17.20
-7	1	-13	3.20	33.40
-7	1	-13	7.80	16.20
7	1	13	-1.90	8.30
-7	1	-13	-3.90	10.30
-8	-2	13	-5.90	26.00
8	-2	-13	-2.70	12.90
8	-2	-13	26.80	19.60
8	2	-13	-3.90	14.40
-8	2	13	0.00	23.50

-8	2	13	-7.10	23.30
7	-2	-13	26.90	14.50
7	-2	-13	17.30	17.40
-7	-2	13	-21.70	23.80
-7	2	13	-16.60	22.20
-7	2	13	47.40	23.70
7	2	-13	22.90	13.40
-6	-2	13	6.00	20.60
6	-2	-13	1.60	13.50
-6	-2	13	-10.20	12.20
6	-2	-13	6.20	9.60
-6	2	13	-20.20	19.20
-6	2	13	23.30	19.30
6	2	-13	12.80	11.30
-5	-2	13	70.59	22.60
-5	-2	13	102.99	24.80
5	-2	-13	92.79	21.50
5	-2	-13	108.49	19.50
5	2	-13	111.19	17.80
-5	2	13	107.09	24.60
-5	2	13	101.99	27.00
-4	-2	13	16.50	16.30
4	-2	-13	5.20	9.90
4	-2	-13	11.90	14.10
-4	-2	13	6.60	8.60
-4	2	13	1.50	9.20
4	2	-13	17.70	11.00
-4	2	13	0.10	13.60
-3	-2	13	74.69	12.90
-3	-2	13	72.19	24.00
3	-2	-13	74.19	17.90

3	-2	-13	83.69	16.50
-3	2	13	62.09	22.50
-3	2	13	61.19	12.70
3	2	-13	39.20	15.70
3	2	-13	60.09	16.40
-3	2	13	63.19	18.30
-2	-2	13	3.80	6.30
2	-2	-13	7.10	16.80
2	-2	-13	-3.50	7.50
-2	-2	13	-8.70	14.50
-2	-2	13	1.90	7.90
-2	2	13	-11.40	11.40
2	2	-13	6.50	9.20
2	2	-13	-29.50	13.00
-2	2	13	-9.30	14.60
-2	2	13	0.60	8.20
-1	-2	13	-1.30	5.50
-1	-2	13	-4.70	7.10
1	-2	-13	0.20	9.90
1	-2	-13	-3.00	7.30
-1	2	13	5.20	15.20
1	2	-13	1.70	13.20
-1	2	13	4.20	6.80
-1	2	13	-3.80	10.50
1	2	-13	6.40	7.90
0	-2	13	5.90	5.30
0	-2	-13	-5.00	15.90
0	-2	13	-4.80	14.30
0	-2	13	-13.90	6.60
0	-2	-13	-0.20	10.20
0	-2	-13	2.10	9.10

0	2	-13	3.20	8.80
0	2	-13	6.00	9.80
0	2	-13	19.90	18.90
0	2	13	21.70	11.90
0	2	13	4.40	7.00
0	2	13	-6.80	15.30
-1	-2	-13	-10.20	8.10
1	-2	13	4.80	4.90
1	-2	13	-12.30	14.50
-1	-2	-13	-15.70	8.70
-1	-2	-13	-13.20	17.60
1	2	13	4.70	7.40
1	2	13	1.50	10.60
-1	2	-13	-5.80	16.60
-1	2	-13	21.40	18.10
1	2	13	-22.00	15.90
-1	2	-13	-2.60	8.00
-1	2	-13	-15.70	10.00
-2	-2	-13	-18.70	12.40
2	-2	13	7.10	12.10
2	-2	13	1.80	4.20
-2	-2	-13	-20.40	13.80
2	-2	13	-3.70	6.00
-2	-2	-13	-12.70	9.60
-2	-2	-13	2.40	9.30
-2	2	-13	12.40	13.70
2	2	13	-15.00	5.80
-2	2	-13	-2.10	12.00
-2	2	-13	-29.20	18.40
-2	2	-13	-0.30	10.20
2	2	13	12.00	12.10

-2	2	-13	1.20	8.10
-3	-2	-13	60.29	15.10
3	-2	13	72.89	7.90
-3	-2	-13	78.69	14.20
-3	-2	-13	120.59	28.10
-3	-2	-13	58.39	22.60
-3	2	-13	64.69	14.00
-3	2	-13	84.39	25.00
3	2	13	88.49	12.80
-3	2	-13	81.39	15.10
-3	2	-13	67.79	17.00
-3	2	-13	85.49	24.00
3	2	13	77.89	19.60
-4	-2	-13	18.80	10.40
-4	-2	-13	-6.60	15.00
-4	-2	-13	-10.00	19.60
-4	-2	-13	11.90	11.10
4	2	13	0.00	7.00
-4	2	-13	2.00	15.90
4	2	13	-3.20	12.70
-4	2	-13	3.20	11.80
-4	2	-13	5.60	9.10
-4	2	-13	-2.70	9.30
-5	-2	-13	75.09	13.60
-5	-2	-13	36.00	12.60
-5	-2	-13	46.50	24.00
-5	-2	-13	70.79	23.00
-5	2	-13	40.90	24.60
-5	2	-13	78.89	14.80
-5	2	-13	46.00	10.20
5	2	13	66.49	11.40

-5	2	-13	72.59	17.20
-6	-2	-13	16.40	20.60
-6	-2	-13	5.10	10.30
-6	-2	-13	-20.20	16.30
-6	-2	-13	-8.40	8.40
-6	2	-13	18.40	14.20
6	2	13	-5.50	8.50
-6	2	-13	9.10	10.50
-6	2	-13	-9.30	20.70
-7	-2	-13	9.90	12.90
7	2	13	-5.80	9.40
-7	2	-13	-3.60	10.90
-7	2	-13	4.20	20.20
-7	2	-13	9.50	15.70
7	-3	-13	11.00	15.00
7	-3	-13	-6.40	11.80
-7	-3	13	-12.00	21.30
7	3	-13	-1.50	11.70
-7	3	13	-13.50	22.70
-7	3	13	-9.20	22.90
6	-3	-13	0.60	12.10
-6	-3	13	12.70	21.20
6	-3	-13	-15.10	14.30
-6	3	13	6.00	22.00
6	3	-13	-8.90	10.00
5	-3	-13	3.40	10.90
5	-3	-13	14.20	13.90
-5	-3	13	5.40	18.40
-5	3	13	-3.20	16.20
-5	3	13	35.70	20.80
5	3	-13	-0.30	10.60

-4	-3	13	25.00	9.90
-4	-3	13	-1.10	17.90
4	-3	-13	7.00	9.20
-4	-3	13	5.30	8.80
4	-3	-13	-9.50	12.30
-4	3	13	9.70	14.20
-4	3	13	0.50	16.30
4	3	-13	8.40	10.80
-4	3	13	9.80	9.80
3	-3	-13	34.80	14.50
3	-3	-13	24.50	11.40
-3	-3	13	44.40	9.90
-3	-3	13	51.09	18.20
-3	3	13	63.59	23.00
-3	3	13	42.10	17.40
-3	3	13	23.00	8.40
3	3	-13	57.79	13.30
-2	-3	13	5.40	6.10
2	-3	-13	10.70	11.30
2	-3	-13	-2.00	8.40
-2	-3	13	2.30	14.60
-2	3	13	3.80	15.40
2	3	-13	1.70	9.00
-2	3	13	-3.90	12.30
-2	3	13	1.90	8.50
2	3	-13	0.00	10.50
1	-3	-13	173.08	26.10
-1	-3	13	117.69	12.00
1	-3	-13	121.59	19.30
-1	-3	13	154.98	29.90
-1	3	13	122.19	22.90

-1	3	13	124.29	16.90
-1	3	13	107.59	26.50
1	3	-13	140.29	21.90
1	3	-13	94.79	24.60
1	3	-13	142.69	17.80
0	-3	-13	29.70	16.90
0	-3	-13	-0.50	7.70
0	-3	13	13.80	15.30
0	-3	-13	3.50	12.60
0	-3	13	6.60	4.90
0	-3	13	-4.00	5.30
0	3	13	2.50	11.50
0	3	-13	-0.70	8.00
0	3	-13	-19.30	12.20
0	3	-13	17.10	17.30
0	3	13	-4.30	15.80
0	3	13	8.60	8.80
1	-3	13	80.69	21.40
-1	-3	-13	79.29	15.60
1	-3	13	95.99	7.20
-1	-3	-13	78.49	21.80
-1	-3	-13	90.69	18.80
1	-3	13	85.39	7.70
1	3	13	105.99	15.20
1	3	13	78.79	24.90
1	3	13	91.39	20.30
-1	3	-13	75.49	23.60
-1	3	-13	93.29	14.90
-1	3	-13	66.59	21.00
-1	3	-13	113.09	17.90
-2	-3	-13	1.00	10.80

-2	-3	-13	8.00	18.10
-2	-3	-13	-2.40	9.40
-2	-3	-13	13.10	16.20
-2	3	-13	31.60	17.80
-2	3	-13	5.90	9.30
-2	3	-13	0.90	7.60
-2	3	-13	-1.10	11.10
2	3	13	2.50	12.60
2	3	13	20.70	8.40
-2	3	-13	-0.20	16.40
-3	-3	-13	24.90	17.90
-3	-3	-13	54.59	21.70
-3	-3	-13	16.10	11.70
-3	-3	-13	34.70	12.20
-3	3	-13	21.60	12.00
3	3	13	14.70	9.00
-3	3	-13	25.20	15.40
-3	3	-13	25.70	10.40
-3	3	-13	56.69	13.20
3	3	13	15.30	12.20
-3	3	-13	15.60	19.50
-4	-3	-13	5.50	9.50
-4	-3	-13	-19.60	16.00
-4	-3	-13	15.90	10.10
-4	-3	-13	32.70	21.80
-4	3	-13	0.00	12.40
-4	3	-13	-7.10	9.10
-4	3	-13	5.50	15.50
4	3	13	-16.00	11.90
-4	3	-13	-5.60	9.50
-5	-3	-13	-9.90	9.70

-5	-3	-13	-1.20	20.20
-5	-3	-13	28.00	13.10
-5	3	-13	13.60	9.30
-5	3	-13	23.60	20.10
5	3	13	5.80	14.10
-5	3	-13	14.60	12.50
5	3	13	6.40	8.20
-6	-3	-13	8.20	19.90
-6	-3	-13	2.80	11.10
-6	-3	-13	10.30	23.30
-6	-3	-13	5.70	10.30
-6	3	-13	-8.90	22.60
-6	3	-13	9.60	13.60
6	3	13	4.80	8.70
-7	-3	-13	-32.30	12.00
-7	-3	-13	0.60	20.80
-7	3	-13	1.10	13.00
7	3	13	4.30	9.80
-7	3	-13	42.80	25.80
-7	3	-13	-15.50	9.90
-7	-4	13	-0.60	23.90
-7	4	13	-41.80	40.90
7	4	-13	-1.70	20.30
-7	4	13	-7.00	20.50
6	-4	-13	-19.80	13.30
6	-4	-13	17.50	12.30
-6	4	13	1.10	19.90
-6	4	13	39.80	20.90
6	4	-13	19.30	11.90
5	-4	-13	19.30	14.50
-5	-4	13	83.09	24.40

5	-4	-13	40.80	13.70
-5	4	13	25.60	18.00
-5	4	13	10.40	20.60
5	4	-13	43.50	14.20
-4	-4	13	10.00	19.00
4	-4	-13	6.30	8.40
-4	-4	13	-0.10	8.10
4	-4	-13	18.60	15.60
4	4	-13	19.70	10.10
-4	4	13	16.20	19.50
-4	4	13	-2.10	13.50
-4	4	13	9.40	8.60
-3	-4	13	20.60	17.70
3	-4	-13	2.00	9.50
-3	-4	13	-7.90	6.70
3	-4	-13	-6.40	11.40
3	4	-13	-7.30	8.00
-3	4	13	-21.70	13.70
-3	4	13	3.20	9.10
-3	4	13	3.60	17.30
-2	-4	13	-17.70	16.10
2	-4	-13	15.10	13.20
-2	-4	13	-0.20	7.40
2	-4	-13	14.20	8.90
2	4	-13	1.20	19.30
2	4	-13	7.50	8.60
-2	4	13	6.50	15.60
-2	4	13	5.90	13.10
-2	4	13	6.90	9.30
-1	-4	13	24.40	5.60
1	-4	-13	34.60	12.00

1	-4	-13	24.20	13.30
-1	-4	13	22.10	6.10
-1	-4	13	29.00	18.00
-1	4	13	40.60	16.20
-1	4	13	33.40	10.60
1	4	-13	-4.90	10.60
-1	4	13	32.40	15.90
1	4	-13	57.19	19.10
1	4	-13	11.80	9.20
0	-4	-13	11.00	13.70
0	-4	-13	7.20	9.70
0	-4	13	5.40	27.70
0	-4	-13	15.60	12.40
0	4	13	10.90	8.10
0	4	13	26.20	14.60
0	4	13	15.70	18.90
0	4	-13	11.40	15.10
0	4	-13	8.20	8.40
-1	-4	-13	18.00	12.70
-1	-4	-13	13.80	16.00
-1	-4	-13	1.70	8.90
1	-4	13	16.10	14.60
-1	-4	-13	47.60	20.90
-1	4	-13	16.20	15.50
1	4	13	0.60	19.10
1	4	13	32.90	16.90
1	4	13	5.20	9.20
-1	4	-13	25.60	9.60
-1	4	-13	13.20	17.50
-2	-4	-13	-4.70	17.10
-2	-4	-13	-1.20	9.10

-2	-4	-13	10.20	11.40
-2	-4	-13	-9.80	15.70
-2	4	-13	15.10	16.50
-2	4	-13	-8.60	9.70
-2	4	-13	-8.10	7.80
-2	4	-13	-2.40	9.60
-2	4	-13	-4.30	14.60
2	4	13	1.90	8.90
2	4	13	-13.40	11.10
-3	-4	-13	2.30	11.00
-3	-4	-13	-4.60	16.10
-3	-4	-13	21.20	18.60
-3	-4	-13	-12.40	8.80
-3	4	-13	10.50	10.10
-3	4	-13	2.90	10.10
-3	4	-13	-9.10	7.30
-3	4	-13	14.90	18.20
3	4	13	3.10	7.10
3	4	13	14.20	16.20
-4	-4	-13	-3.40	9.90
-4	-4	-13	2.00	10.10
-4	-4	-13	4.20	17.90
-4	-4	-13	-14.40	18.00
-4	4	-13	36.50	21.50
-4	4	-13	5.30	9.50
-4	4	-13	6.80	9.10
-4	4	-13	-17.20	9.80
4	4	13	-9.80	13.90
4	4	13	8.70	9.20
-5	-4	-13	11.40	12.10
-5	-4	-13	-6.50	10.70

-5	-4	-13	-1.70	19.30
-5	-4	-13	1.20	16.40
-5	4	-13	12.60	9.60
5	4	13	31.30	10.30
-5	4	-13	28.90	18.90
5	4	13	-6.60	17.80
-6	-4	-13	-11.00	10.50
-6	-4	-13	25.60	19.90
-6	-4	-13	-8.10	11.40
-6	-4	-13	19.80	21.20
6	4	13	4.10	9.30
-6	4	-13	2.80	10.20
-6	4	-13	-30.80	17.90
-6	4	-13	-7.70	11.00
-7	-4	-13	4.10	24.30
-7	4	-13	0.70	23.20
-7	4	-13	-15.70	13.20
7	4	13	-19.50	9.90
-7	4	-13	-5.20	11.50
-7	-5	13	29.40	28.20
7	-5	-13	24.40	15.40
7	-5	-13	25.10	17.80
7	5	-13	13.30	14.10
-7	5	13	-29.30	20.80
-7	5	13	15.80	24.20
-6	-5	13	12.70	22.40
6	-5	-13	-6.60	14.40
6	-5	-13	-17.70	12.20
-6	5	13	11.30	22.00
-6	5	13	-17.40	21.40
6	5	-13	-1.60	10.30

5	−5	−13	7.00	16.50
5	−5	−13	14.50	12.50
−5	−5	13	10.50	23.80
5	5	−13	−5.70	9.40
−5	5	13	11.70	22.40
−5	5	13	−5.10	7.80
−5	5	13	−15.40	16.40
−4	−5	13	2.40	8.10
4	−5	−13	9.70	11.00
4	−5	−13	28.90	17.20
−4	−5	13	9.70	19.30
−4	5	13	−19.00	16.70
−4	5	13	25.50	18.50
4	5	−13	0.20	9.20
−4	5	13	22.50	11.10
3	−5	−13	10.90	13.70
−3	−5	13	10.60	6.50
−3	−5	13	15.20	18.80
3	−5	−13	11.20	8.60
−3	5	13	2.90	9.80
−3	5	13	−4.90	17.30
−3	5	13	29.40	16.00
3	5	−13	11.70	9.50
−2	−5	13	8.00	16.50
2	−5	−13	5.00	10.00
−2	−5	13	3.10	4.60
2	−5	−13	−10.70	11.10
2	5	−13	21.60	9.70
−2	5	13	5.50	8.60
−2	5	13	−3.90	17.10
−1	−5	13	42.10	18.60

1	-5	-13	24.90	11.20
1	-5	-13	37.10	19.00
1	-5	-13	30.10	14.10
1	5	-13	52.29	11.80
-1	5	13	29.30	19.30
-1	5	13	-0.10	9.00
1	5	-13	57.79	17.40
0	-5	-13	4.00	8.60
0	-5	-13	3.60	10.30
0	-5	13	10.30	14.60
0	-5	-13	9.40	17.20
0	5	13	17.40	15.00
0	5	-13	15.50	9.50
0	5	13	14.60	8.60
0	5	-13	21.30	15.40
0	5	-13	13.30	10.50
-1	-5	-13	14.40	9.40
-1	-5	-13	30.10	18.80
1	-5	13	-1.80	14.50
-1	-5	-13	-5.10	19.80
1	5	13	6.90	16.90
-1	5	-13	9.70	9.40
-1	5	-13	7.70	15.60
-1	5	-13	42.90	12.90
1	5	13	1.50	9.40
-1	5	-13	4.20	9.80
1	5	13	0.40	12.00
-2	-5	-13	18.00	17.00
-2	-5	-13	-9.20	8.50
-2	-5	-13	-6.90	10.60
-2	-5	-13	-23.40	16.40

-2	5	-13	17.10	13.70
-2	5	-13	19.60	15.70
2	5	13	3.90	15.30
-2	5	-13	-4.40	9.00
2	5	13	11.20	9.00
-2	5	-13	9.70	8.80
-3	-5	-13	37.50	19.90
-3	-5	-13	25.10	22.90
-3	-5	-13	50.79	14.30
-3	-5	-13	24.90	12.50
3	5	13	42.20	19.40
-3	5	-13	50.59	19.80
-3	5	-13	49.00	15.30
3	5	13	52.59	13.10
-3	5	-13	21.60	9.80
-3	5	-13	38.40	13.10
-3	5	-13	51.49	12.00
-4	-5	-13	1.40	10.40
-4	-5	-13	-16.80	18.70
-4	-5	-13	-0.10	11.90
-4	-5	-13	2.60	14.40
-4	5	-13	39.20	22.10
-4	5	-13	30.80	12.50
-4	5	-13	-6.90	8.30
4	5	13	-11.50	7.90
-4	5	-13	12.90	10.00
4	5	13	-29.50	15.70
-5	-5	-13	2.40	11.40
-5	-5	-13	-2.70	18.70
-5	-5	-13	7.60	11.40
-5	-5	-13	26.20	22.60

5	5	13	4.30	9.90
-5	5	-13	6.20	18.20
-5	5	-13	9.20	9.50
5	5	13	-2.30	17.90
-5	5	-13	22.50	10.20
-6	-5	-13	-2.60	20.70
-6	-5	-13	-9.80	12.80
-6	-5	-13	3.60	12.60
-6	5	-13	9.90	10.20
-6	5	-13	-19.20	11.90
-6	5	-13	-34.60	19.90
6	5	13	0.60	10.30
-7	-5	-13	-14.00	24.20
-7	5	-13	6.90	12.10
6	-6	-13	18.20	13.70
-6	-6	13	0.10	26.30
-6	6	13	-1.30	22.10
6	6	-13	-1.70	12.30
-5	-6	13	29.70	26.40
5	-6	-13	14.40	15.00
-5	-6	13	39.00	10.90
5	-6	-13	11.00	13.50
-5	6	13	39.40	11.60
-5	6	13	8.80	21.60
-5	6	13	37.10	24.90
5	6	-13	7.00	11.00
-4	-6	13	8.90	21.30
4	-6	-13	13.30	11.90
-4	-6	13	13.70	7.70
4	-6	-13	7.20	13.80
-4	6	13	24.30	19.50

-4	6	13	10.60	17.60
4	6	-13	10.30	10.20
-4	6	13	4.60	10.70
3	-6	-13	16.90	10.60
3	-6	-13	6.70	12.10
-3	-6	13	6.80	5.50
-3	-6	13	12.20	19.10
-3	6	13	-6.70	17.40
3	6	-13	-2.90	8.30
-3	6	13	-10.10	8.50
-3	6	13	6.90	17.40
2	-6	-13	5.40	12.90
2	-6	-13	-6.70	8.70
-2	-6	13	9.00	18.10
-2	6	13	-7.70	15.50
-2	6	13	-2.30	7.90
2	6	-13	4.60	8.70
-2	6	13	6.90	16.90
1	-6	-13	20.30	11.40
1	-6	-13	53.39	19.90
-1	-6	13	19.00	18.50
1	-6	-13	2.50	11.80
-1	6	13	14.60	19.10
1	6	-13	38.50	9.90
-1	6	13	-3.70	15.40
-1	6	13	4.70	8.80
0	-6	13	1.00	16.10
0	-6	-13	4.60	9.50
0	-6	-13	1.20	17.90
0	-6	-13	28.90	11.70
0	6	-13	16.10	13.60

0	6	13	2.40	9.10
0	6	13	7.00	18.20
0	6	-13	5.20	9.80
0	6	-13	9.00	8.50
0	6	13	10.80	14.30
1	-6	13	0.10	14.50
-1	-6	-13	-11.90	17.10
-1	-6	-13	2.20	10.20
-1	-6	-13	1.90	9.30
-1	-6	-13	15.60	19.40
-1	6	-13	12.80	9.40
-1	6	-13	8.50	8.70
1	6	13	-22.00	15.00
1	6	13	-9.60	9.90
-1	6	-13	19.30	11.90
-1	6	-13	29.20	22.40
-2	-6	-13	1.50	9.80
-2	-6	-13	-3.30	19.50
-2	-6	-13	-6.60	16.00
-2	-6	-13	4.80	12.50
2	6	13	-15.40	14.00
-2	6	-13	8.90	9.00
-2	6	-13	3.40	9.80
2	6	13	-2.30	8.20
-2	6	-13	8.40	7.90
-2	6	-13	-3.00	14.80
-3	-6	-13	3.90	10.20
-3	-6	-13	6.70	18.40
-3	-6	-13	-1.60	20.00
-3	-6	-13	-3.50	11.20
-3	6	-13	7.80	7.20

-3	6	-13	-17.20	17.90
3	6	13	-1.20	17.00
-3	6	-13	-8.20	9.10
3	6	13	5.50	9.40
-4	-6	-13	-28.40	19.90
-4	-6	-13	-9.30	19.20
-4	-6	-13	-3.90	12.20
-4	6	-13	10.50	9.60
4	6	13	1.10	18.20
-4	6	-13	-18.80	20.20
-4	6	-13	-24.00	10.80
-4	6	-13	-13.90	6.40
-4	6	-13	-0.90	10.40
4	6	13	15.40	11.40
-5	-6	-13	40.60	16.10
-5	-6	-13	9.20	24.30
-5	-6	-13	19.90	24.10
-5	-6	-13	20.40	12.30
5	6	13	29.70	11.10
-5	6	-13	11.00	9.70
5	6	13	3.30	22.20
-5	6	-13	12.20	23.90
-6	-6	-13	30.70	13.60
-6	-6	-13	6.90	13.30
-6	-6	-13	32.00	26.60
-6	-6	-13	41.40	27.00
-6	6	-13	-28.40	31.40
6	6	13	-16.50	17.30
6	6	13	-2.10	11.00
-6	6	-13	-6.00	10.10
6	-7	-13	22.40	18.00

-6	-7	13	37.50	29.40
-6	7	13	27.40	22.90
6	7	-13	-5.70	11.80
-6	7	13	11.30	22.80
-5	-7	13	7.60	27.10
5	-7	-13	0.80	12.50
5	-7	-13	29.80	16.20
-5	-7	13	-3.50	9.90
5	7	-13	-7.20	10.40
-5	7	13	10.10	22.50
-5	7	13	-3.50	22.70
-5	7	13	1.20	11.80
-4	-7	13	8.40	25.00
4	-7	-13	-0.40	11.60
4	-7	-13	15.90	14.20
-4	-7	13	-1.00	7.00
4	7	-13	3.00	9.80
-4	7	13	5.30	22.10
-4	7	13	-4.90	18.40
-4	7	13	-2.60	11.30
-3	-7	13	20.40	24.90
3	-7	-13	-8.50	13.40
3	-7	-13	5.20	11.20
-3	7	13	10.30	18.60
-3	7	13	12.70	11.10
-3	7	13	31.60	20.80
2	-7	-13	9.70	11.40
2	-7	-13	-2.70	11.60
2	-7	-13	9.40	16.00
-2	7	13	-22.60	15.90
-2	7	13	17.00	20.10

2	7	-13	12.40	8.50
-2	7	13	7.30	11.30
1	-7	-13	9.30	17.40
1	-7	-13	15.20	15.70
-1	-7	13	-36.70	15.80
1	-7	-13	-3.60	9.40
-1	7	13	-6.30	17.30
-1	7	13	11.20	10.20
-1	7	13	-9.30	18.90
1	7	-13	3.60	8.40
0	-7	13	-12.90	14.60
0	-7	-13	3.80	11.20
0	-7	-13	-11.50	9.40
0	-7	-13	-11.90	18.30
0	7	13	-6.70	14.50
0	7	-13	14.10	9.40
0	7	13	-12.30	8.20
0	7	13	0.30	18.40
-1	-7	-13	-2.10	8.10
-1	-7	-13	31.90	18.60
-1	-7	-13	16.50	12.20
-1	-7	-13	-14.60	19.50
-1	7	-13	4.40	8.00
1	7	13	-8.50	16.80
1	7	13	0.50	8.90
-1	7	-13	0.90	11.10
-1	7	-13	-7.30	8.60
-1	7	-13	-20.80	18.00
-2	-7	-13	5.40	21.40
-2	-7	-13	-0.50	11.60
-2	-7	-13	-2.20	18.40

-2	-7	-13	16.20	11.30
-2	7	-13	6.40	5.20
-2	7	-13	24.80	10.00
-2	7	-13	7.80	13.30
2	7	13	-3.00	9.00
2	7	13	11.80	19.30
-2	7	-13	-8.40	19.80
-3	-7	-13	11.50	11.90
-3	-7	-13	10.30	21.90
-3	-7	-13	3.80	21.90
-3	-7	-13	19.20	13.30
-3	7	-13	9.80	19.90
3	7	13	-2.80	17.00
-3	7	-13	-12.90	8.70
3	7	13	3.90	11.80
-4	-7	-13	-27.10	12.10
-4	-7	-13	-7.00	23.30
-4	-7	-13	5.20	12.80
-4	-7	-13	11.10	19.40
-4	7	-13	-22.50	20.40
4	7	13	8.10	16.80
-4	7	-13	7.40	9.90
4	7	13	0.90	11.20
-5	-7	-13	57.99	59.59
-5	-7	-13	-35.50	12.00
-5	-7	-13	14.10	14.60
-5	-7	-13	1.00	23.30
-5	7	-13	-6.20	9.70
5	7	13	-39.40	19.80
5	7	13	11.10	12.10
-5	7	-13	41.50	24.40

-6	-7	-13	28.30	28.30
-6	-7	-13	-3.00	14.80
-6	-7	-13	1.10	24.50
-6	7	-13	-16.90	24.20
6	7	13	2.70	12.10
-5	-8	13	1.30	38.00
5	-8	-13	7.60	12.90
5	-8	-13	-31.60	13.60
-5	8	13	-2.40	20.90
-5	8	13	9.90	11.70
5	8	-13	-4.10	10.90
-5	8	13	-3.80	20.20
4	-8	-13	-4.90	14.80
-4	-8	13	25.30	29.80
4	-8	-13	4.10	11.60
-4	8	13	16.90	22.30
4	8	-13	-4.10	10.80
-4	8	13	1.70	18.60
-4	8	13	-2.80	12.20
3	-8	-13	49.60	12.90
3	-8	-13	19.90	17.90
-3	8	13	18.30	21.80
-3	8	13	41.80	13.60
-3	8	13	46.90	24.80
3	8	-13	21.50	12.80
2	-8	-13	18.50	16.40
2	-8	-13	13.00	11.00
2	-8	-13	-7.80	18.60
-2	8	13	18.10	21.00
-2	8	13	-16.00	19.70
2	8	-13	32.80	10.30

-2	8	13	15.20	10.50
1	-8	-13	34.60	13.20
1	-8	-13	68.39	21.60
1	-8	-13	29.20	16.70
-1	8	13	50.49	28.30
-1	8	13	56.99	14.80
-1	8	13	73.69	28.10
1	8	-13	26.20	10.50
0	-8	-13	13.90	13.60
0	-8	-13	7.30	19.40
0	-8	-13	-10.20	11.50
0	-8	-13	-3.30	22.70
0	8	13	16.70	23.60
0	8	13	9.60	11.40
0	8	13	-11.50	19.40
0	8	-13	15.10	8.80
0	8	-13	19.80	10.40
-1	-8	-13	2.50	19.10
-1	-8	-13	37.50	23.00
-1	-8	-13	1.30	10.80
-1	-8	-13	23.70	17.00
-1	8	-13	26.30	10.20
1	8	13	34.20	13.20
-1	8	-13	11.20	19.20
1	8	13	48.20	21.80
-2	-8	-13	2.00	24.00
-2	-8	-13	32.90	18.30
-2	-8	-13	-5.80	11.00
-2	-8	-13	-5.90	14.30
2	8	13	12.10	22.00
-2	8	-13	-2.10	9.30

-2	8	-13	40.30	15.70
-2	8	-13	-3.20	19.30
2	8	13	3.80	11.70
-3	-8	-13	27.10	13.50
-3	-8	-13	27.70	27.60
-3	-8	-13	7.80	16.00
-3	8	-13	17.90	18.20
3	8	13	6.30	12.30
-3	8	-13	15.90	10.00
3	8	13	-1.80	17.50
-3	8	-13	27.30	22.40
-4	-8	-13	-13.90	25.40
-4	-8	-13	13.60	13.20
-4	-8	-13	12.20	14.20
-4	-8	-13	-30.60	23.90
4	8	13	13.80	14.40
-4	8	-13	41.00	21.10
-4	8	-13	-12.90	22.40
-5	-8	-13	-3.50	24.20
-5	-8	-13	-14.40	29.00
-5	-8	-13	-17.60	15.20
-5	-8	-13	38.10	15.10
-5	8	-13	39.90	22.40
5	8	13	-54.39	21.00
5	8	13	9.90	12.00
4	-9	-13	23.90	13.20
4	-9	-13	34.20	18.20
4	9	-13	-3.80	10.80
-4	9	13	-10.30	12.40
-4	9	13	-11.50	21.30
3	-9	-13	-6.00	11.60

3	-9	-13	-3.70	22.20
-3	9	13	18.60	22.00
3	9	-13	5.50	11.40
2	-9	-13	42.50	22.00
2	-9	-13	23.20	12.90
2	-9	-13	-18.80	14.80
2	9	-13	-2.60	9.30
-2	9	13	11.90	12.30
-2	9	13	14.20	19.80
-2	9	13	28.40	20.80
1	-9	-13	-5.00	14.50
1	-9	-13	2.30	11.90
1	-9	-13	-14.80	19.90
-1	9	13	5.80	19.90
-1	9	13	15.80	21.10
-1	9	13	-7.30	12.70
1	9	-13	4.40	9.70
0	-9	-13	-22.40	18.50
0	-9	-13	32.80	24.90
0	-9	-13	-0.10	15.70
0	-9	-13	-1.40	12.30
0	9	13	22.90	12.10
0	9	13	7.20	21.60
0	9	-13	38.00	10.80
-1	-9	-13	12.40	12.20
-1	-9	-13	24.60	23.50
-1	-9	-13	7.80	15.20
-1	-9	-13	25.70	19.60
-1	9	-13	29.50	21.70
-1	9	-13	5.90	10.30
1	9	13	8.50	12.80

1	9	13	-3.20	23.00
-2	-9	-13	-14.60	10.50
-2	-9	-13	-21.00	24.40
-2	-9	-13	24.40	20.30
-2	-9	-13	11.50	15.50
2	9	13	12.60	21.50
-2	9	-13	-15.20	10.20
-2	9	-13	-14.90	20.40
2	9	13	3.10	14.10
-3	-9	-13	19.50	15.50
-3	-9	-13	-14.20	25.40
-3	-9	-13	-6.00	14.30
-3	-9	-13	17.00	25.30
-3	9	-13	41.30	23.60
3	9	13	22.80	13.00
3	9	13	0.20	18.70
-3	9	-13	-24.50	18.90
-4	-9	-13	12.10	15.10
-4	-9	-13	17.60	24.40
-4	-9	-13	37.10	15.40
-4	-9	-13	-6.50	31.40
-4	9	-13	2.30	24.00
4	9	13	-4.60	11.50
4	9	13	9.50	25.10
-4	9	-13	44.90	24.00
3	-10	-13	26.20	13.20
3	-10	-13	4.30	18.30
3	10	-13	-1.30	10.90
-3	10	13	-7.80	26.00
-3	10	13	5.40	12.30
-3	10	13	11.10	25.90

2	-10	-13	19.40	13.60
2	-10	-13	20.80	18.00
-2	10	13	14.80	24.90
2	10	-13	1.30	10.70
-2	10	13	-6.50	24.50
-2	10	13	-16.40	12.50
1	-10	-13	31.80	29.20
1	-10	-13	28.90	17.40
1	-10	-13	-14.00	12.10
1	10	-13	28.00	10.80
-1	10	13	-14.40	26.10
-1	10	13	3.80	13.90
0	-10	-13	1.70	27.90
0	-10	-13	-6.30	12.30
0	-10	-13	32.00	21.90
0	10	-13	16.60	10.10
0	10	13	-35.50	24.40
0	10	13	25.80	16.50
0	10	-13	7.90	20.80
-1	-10	-13	-17.70	27.10
-1	-10	-13	33.30	23.40
-1	-10	-13	16.60	15.40
1	10	13	1.60	22.40
1	10	13	-2.50	14.50
-1	10	-13	-0.30	22.70
-2	-10	-13	4.00	22.90
-2	-10	-13	-13.90	27.20
-2	-10	-13	22.30	13.40
-2	-10	-13	-5.80	15.00
-2	10	-13	11.20	23.10
2	10	13	-1.60	13.40

2	10	13	-7.70	22.00
-3	-10	-13	-2.50	17.10
-3	-10	-13	10.30	30.60
-3	-10	-13	-4.20	15.90
-3	-10	-13	30.70	25.30
-3	10	-13	26.60	26.10
3	10	13	-14.20	21.80
3	10	13	13.70	15.30
-7	0	14	12.40	23.00
7	0	-14	-5.70	12.70
7	0	-14	16.00	17.30
-7	0	14	-19.40	19.00
6	0	-14	-7.00	13.10
-6	0	14	-10.90	18.70
-6	0	14	-7.60	17.30
6	0	-14	7.10	14.10
-5	0	14	-10.30	15.00
5	0	-14	10.30	10.00
-5	0	14	0.50	19.70
5	0	-14	-15.30	11.70
4	0	-14	-10.30	10.90
-4	0	14	5.80	14.00
4	0	-14	3.70	13.40
-4	0	14	-30.40	15.80
-4	0	14	3.20	9.40
3	0	-14	14.40	11.10
-3	0	14	-4.20	8.50
3	0	-14	-8.40	12.70
-3	0	14	3.50	12.30
-3	0	14	-6.40	17.80
-2	0	14	16.40	17.90

-2	0	14	35.60	14.00
2	0	-14	-5.20	12.10
2	0	-14	19.30	11.40
-2	0	14	3.10	7.50
1	0	-14	-0.60	8.60
-1	0	14	-2.70	7.10
-1	0	14	0.10	10.50
-1	0	14	-13.30	17.40
0	0	14	28.70	13.40
0	0	14	22.20	8.00
0	0	14	16.90	19.50
0	0	-14	20.00	12.90
-1	0	-14	4.20	11.30
1	0	14	-9.10	11.60
-1	0	-14	5.90	9.90
1	0	14	-5.60	6.60
1	0	14	27.70	19.00
-1	0	-14	16.60	20.40
-2	0	-14	7.00	17.10
2	0	14	6.00	6.70
2	0	14	7.30	17.50
-2	0	-14	5.00	14.20
2	0	14	8.30	12.30
-2	0	-14	-1.90	10.70
-3	0	-14	-6.20	9.70
-3	0	-14	3.70	14.90
-3	0	-14	3.40	10.10
3	0	14	-5.20	6.00
-3	0	-14	1.90	18.50
-4	0	-14	32.40	11.70
-4	0	-14	10.50	11.00

4	0	14	19.80	7.20
-4	0	-14	38.40	19.10
-4	0	-14	2.20	15.00
-5	0	-14	28.30	17.30
-5	0	-14	5.00	18.50
-5	0	-14	-5.70	10.60
5	0	14	4.00	6.60
6	0	14	-5.50	8.00
-6	0	-14	11.20	17.00
-6	0	-14	-24.80	22.40
-6	0	-14	11.10	12.90
-7	0	-14	2.00	24.80
-7	0	-14	-16.90	12.20
-7	0	-14	17.40	19.80
7	-1	-14	-16.00	16.00
7	-1	-14	1.70	13.00
-7	-1	14	17.90	25.30
7	1	-14	22.10	14.40
7	1	-14	11.20	18.50
-7	1	14	30.00	25.80
-7	1	14	-3.10	21.20
6	-1	-14	-8.60	15.40
6	-1	-14	-1.00	12.80
-6	-1	14	30.90	23.00
-6	-1	14	-23.00	14.80
-6	1	14	-17.40	20.20
-6	1	14	3.60	18.00
6	1	-14	-2.00	11.70
6	1	-14	-6.70	14.10
5	-1	-14	6.10	10.90
-5	-1	14	-12.90	13.50

-5	-1	14	-5.00	20.20
5	-1	-14	9.90	14.10
-5	1	14	22.00	19.60
5	1	-14	7.80	11.40
-5	1	14	-5.70	15.80
5	1	-14	3.40	13.00
-4	-1	14	14.50	12.00
4	-1	-14	8.00	10.80
4	-1	-14	17.00	13.80
-4	-1	14	12.00	9.70
-4	-1	14	43.90	22.20
4	1	-14	26.30	16.60
-4	1	14	-1.30	18.00
-4	1	14	12.10	11.20
-4	1	14	17.20	15.70
4	1	-14	19.10	12.00
-3	-1	14	12.70	12.20
-3	-1	14	11.80	8.30
3	-1	-14	2.10	9.60
-3	-1	14	10.90	19.80
3	-1	-14	7.40	12.20
-3	1	14	-6.80	12.80
3	1	-14	11.00	9.90
3	1	-14	-19.90	10.80
-3	1	14	17.50	9.30
-3	1	14	-5.90	18.30
2	-1	-14	13.10	14.40
-2	-1	14	14.70	19.70
-2	-1	14	20.80	8.00
2	-1	-14	26.30	10.20
-2	-1	14	-8.00	18.10

-2	1	14	37.60	15.30
2	1	-14	24.50	12.40
-2	1	14	31.10	18.50
2	1	-14	28.60	14.00
-2	1	14	24.90	10.90
-1	-1	14	-6.80	15.80
1	-1	-14	15.30	10.80
-1	-1	14	0.60	6.70
-1	-1	14	7.10	9.70
1	-1	-14	5.60	8.90
-1	1	14	-11.00	7.20
-1	1	14	-6.00	16.00
-1	1	14	8.10	11.10
1	1	-14	-5.40	15.90
0	-1	14	17.10	12.30
0	-1	-14	23.80	19.50
0	-1	14	-7.40	6.60
0	-1	14	-2.70	17.70
0	-1	-14	12.00	11.20
0	-1	-14	-7.00	11.10
0	1	14	0.70	10.90
0	1	14	-2.90	7.50
0	1	-14	9.20	9.70
0	1	-14	3.10	11.90
0	1	14	11.90	16.90
1	-1	14	-5.10	6.10
1	-1	14	-0.90	11.30
-1	-1	-14	5.50	9.50
-1	-1	-14	9.50	11.10
1	-1	14	6.20	16.40
-1	-1	-14	2.80	16.70

1	1	14	7.90	17.90
-1	1	-14	7.90	11.10
-1	1	-14	9.70	8.50
-1	1	-14	-11.40	15.90
1	1	14	-0.50	7.10
1	1	14	3.00	12.00
-2	-1	-14	9.30	11.70
-2	-1	-14	10.10	15.70
2	-1	14	10.50	6.20
2	-1	14	50.59	18.30
-2	-1	-14	16.80	11.90
-2	-1	-14	21.00	16.30
-2	1	-14	40.10	20.10
2	1	14	6.70	14.10
-2	1	-14	22.60	14.50
-2	1	-14	19.40	13.50
-2	1	-14	6.90	10.40
2	1	14	11.40	7.70
-3	-1	-14	-16.60	11.00
3	-1	14	-0.60	5.30
-3	-1	-14	-31.70	16.90
-3	-1	-14	-0.30	10.50
-3	-1	-14	-10.50	15.70
3	1	14	3.50	15.70
-3	1	-14	-8.00	8.60
-3	1	-14	13.20	19.60
-3	1	-14	2.00	14.60
-3	1	-14	-7.30	11.70
3	1	14	4.50	7.00
-4	-1	-14	22.40	12.60
-4	-1	-14	22.90	13.00

-4	-1	-14	34.10	19.80
-4	-1	-14	13.20	18.30
4	-1	14	32.10	9.70
-4	1	-14	31.10	22.80
4	1	14	30.30	8.50
-4	1	-14	16.10	11.20
-4	1	-14	27.20	12.50
-4	1	-14	27.30	16.20
-5	-1	-14	-11.60	10.70
-5	-1	-14	5.00	17.00
-5	-1	-14	-4.70	10.40
-5	-1	-14	24.60	22.90
5	-1	14	-2.30	5.70
5	1	14	3.80	8.10
-5	1	-14	43.00	18.70
-5	1	-14	20.10	23.40
-5	1	-14	16.70	10.20
-5	1	-14	8.70	11.10
-6	-1	-14	34.20	20.70
-6	-1	-14	-6.20	24.30
-6	-1	-14	7.10	10.60
-6	1	-14	17.40	17.10
-6	1	-14	3.00	11.00
6	1	14	-2.00	8.60
-7	-1	-14	-22.10	11.90
-7	-1	-14	17.60	26.20
-7	-1	-14	17.50	20.30
-7	1	-14	-23.60	10.90
7	1	14	3.50	9.90
-7	1	-14	28.40	24.60
-7	1	-14	15.70	18.80

7	-2	-14	-9.50	11.60
-7	-2	14	-16.00	24.70
7	-2	-14	1.40	20.10
-7	2	14	-14.70	25.60
7	2	-14	-8.40	11.70
-7	2	14	-18.30	20.40
6	-2	-14	22.70	17.50
6	-2	-14	14.70	13.50
-6	-2	14	20.90	23.10
6	2	-14	11.10	12.80
-6	2	14	30.80	19.80
-6	2	14	-16.50	22.70
-5	-2	14	13.60	10.70
5	-2	-14	-2.10	10.90
5	-2	-14	7.80	13.90
-5	2	14	15.30	19.10
-5	2	14	11.40	11.50
-5	2	14	15.30	21.40
5	2	-14	11.60	11.90
-4	-2	14	-17.50	16.40
4	-2	-14	-1.10	14.40
4	-2	-14	7.20	11.00
-4	2	14	-14.70	8.00
-4	2	14	-5.10	14.80
4	2	-14	-7.20	9.80
-4	2	14	-21.20	15.00
-3	-2	14	35.60	20.70
3	-2	-14	8.80	10.60
-3	-2	14	21.80	9.60
-3	-2	14	18.80	9.50
3	-2	-14	-5.10	13.40

-3	2	14	12.00	9.60
-3	2	14	13.90	13.30
3	2	-14	12.80	12.20
-2	-2	14	-12.20	8.80
2	-2	-14	-13.10	10.90
-2	-2	14	-4.00	6.90
2	-2	-14	0.40	7.90
-2	-2	14	12.20	17.80
-2	2	14	9.40	12.50
-2	2	14	16.10	9.00
2	2	-14	2.00	12.40
2	2	-14	-31.70	15.30
2	2	-14	20.60	10.40
-2	2	14	-1.70	16.90
1	-2	-14	7.00	8.90
-1	-2	14	23.00	11.10
1	-2	-14	1.00	12.50
-1	-2	14	0.00	6.30
-1	-2	14	-8.70	17.30
1	2	-14	-6.10	8.40
-1	2	14	-13.60	12.30
-1	2	14	-4.20	19.20
0	-2	14	60.39	24.90
0	-2	-14	52.99	13.10
0	-2	-14	44.60	20.00
0	-2	-14	44.70	15.90
0	-2	14	35.50	8.20
0	2	-14	30.90	12.00
0	2	14	46.90	15.90
0	2	-14	63.89	17.10
0	2	14	37.30	11.70

0	2	14	50.29	21.10
-1	-2	-14	3.00	9.30
1	-2	14	-1.90	9.80
1	-2	14	-7.70	15.50
1	-2	14	0.10	8.80
-1	-2	-14	-19.00	11.50
-1	-2	-14	22.30	18.00
1	2	14	0.80	12.20
-1	2	-14	-9.60	21.60
-1	2	-14	-7.00	10.40
-1	2	-14	2.00	8.30
1	2	14	14.00	16.00
-1	2	-14	-8.90	16.00
1	2	14	-4.10	7.10
2	-2	14	43.40	14.50
-2	-2	-14	31.80	12.80
-2	-2	-14	34.80	20.30
-2	-2	-14	4.20	11.40
-2	-2	-14	3.40	19.70
2	-2	14	22.80	5.50
2	2	14	20.20	9.10
-2	2	-14	37.70	11.90
-2	2	-14	11.60	11.00
-2	2	-14	28.70	14.90
2	2	14	6.30	15.40
-2	2	-14	14.50	20.10
-3	-2	-14	23.90	12.40
-3	-2	-14	11.20	11.60
-3	-2	-14	5.10	18.30
-3	-2	-14	9.60	17.60
-3	2	-14	4.00	11.60

3	2	14	13.00	14.80
-3	2	-14	1.90	12.90
3	2	14	1.50	7.30
-3	2	-14	-6.00	9.00
-3	2	-14	19.80	18.40
-4	-2	-14	-17.10	17.30
-4	-2	-14	2.10	12.70
-4	-2	-14	12.50	11.90
-4	-2	-14	-22.80	16.10
-4	2	-14	11.90	11.00
-4	2	-14	13.20	11.10
4	2	14	20.40	16.00
-4	2	-14	8.00	13.80
-4	2	-14	-12.50	19.80
4	2	14	10.40	8.40
-5	-2	-14	21.30	20.50
-5	-2	-14	11.50	22.00
-5	-2	-14	-12.60	11.00
-5	-2	-14	17.00	10.80
-5	2	-14	-20.20	8.90
-5	2	-14	3.60	12.10
-5	2	-14	3.80	24.20
-5	2	-14	4.60	12.80
-6	-2	-14	44.90	26.40
-6	-2	-14	37.20	15.20
-6	-2	-14	-7.70	21.50
-6	2	-14	-29.00	31.30
6	2	14	19.80	10.10
-6	2	-14	10.50	11.70
-6	2	-14	10.60	14.80
7	-3	-14	37.30	16.60

-7	-3	14	33.00	26.30
7	-3	-14	17.50	21.90
-7	3	14	29.00	23.20
7	3	-14	2.00	14.20
-7	3	14	-9.30	23.80
6	-3	-14	7.70	19.60
-6	-3	14	9.50	22.70
-6	3	14	18.70	22.10
6	3	-14	-5.30	10.30
-6	3	14	-14.40	20.40
5	-3	-14	8.10	13.10
5	-3	-14	-10.50	17.10
-5	-3	14	0.20	22.10
-5	-3	14	24.40	10.80
-5	3	14	17.30	22.70
-5	3	14	11.50	10.40
-5	3	14	-28.40	19.70
5	3	-14	-7.00	10.30
4	-3	-14	-20.60	14.20
4	-3	-14	14.00	11.70
-4	-3	14	16.40	20.40
-4	-3	14	24.10	9.80
-4	3	14	-4.00	19.60
4	3	-14	22.80	11.70
-4	3	14	-6.50	14.80
-4	3	14	1.90	10.10
3	-3	-14	9.10	10.80
3	-3	-14	6.20	14.60
-3	-3	14	8.20	18.20
-3	-3	14	1.60	7.80
-3	3	14	10.80	15.70

3	3	-14	2.90	9.80
-3	3	14	3.10	18.10
-3	3	14	1.90	9.90
2	-3	-14	6.30	11.20
-2	-3	14	28.50	18.80
2	-3	-14	8.80	13.80
-2	-3	14	7.70	7.00
-2	3	14	8.30	9.50
-2	3	14	2.20	15.60
-1	-3	14	26.70	7.60
1	-3	-14	37.60	16.10
1	-3	-14	32.50	11.50
-1	3	14	42.20	15.80
1	3	-14	25.90	18.60
1	3	-14	8.10	23.30
-1	3	14	43.50	12.10
-1	3	14	58.99	23.70
1	3	-14	35.10	11.90
0	-3	14	2.10	5.00
0	-3	-14	0.60	9.30
0	-3	14	3.30	16.90
0	-3	-14	32.80	18.00
0	-3	-14	-9.30	10.30
0	3	-14	-8.30	10.00
0	3	14	42.20	21.70
0	3	-14	-18.00	19.60
0	3	14	-0.40	7.60
0	3	14	7.60	14.40
0	3	-14	19.00	10.30
1	-3	14	26.50	7.50
-1	-3	-14	15.90	9.90

-1	-3	-14	55.69	20.60
1	-3	14	36.40	18.60
-1	-3	-14	31.00	14.00
-1	-3	-14	44.50	23.10
-1	3	-14	32.90	12.40
1	3	14	14.20	8.70
1	3	14	19.90	18.40
-1	3	-14	5.10	9.00
-1	3	-14	-6.20	17.40
1	3	14	-7.80	15.00
-2	-3	-14	-12.40	10.40
-2	-3	-14	-16.00	18.00
-2	-3	-14	-11.40	16.60
-2	-3	-14	9.90	11.20
2	3	14	1.80	7.90
-2	3	-14	-10.70	17.50
2	3	14	20.90	15.10
-2	3	-14	-5.10	9.10
-2	3	-14	-2.60	11.80
-2	3	-14	21.40	12.50
-3	-3	-14	-7.20	10.30
-3	-3	-14	-22.70	16.70
-3	-3	-14	3.10	17.00
-3	-3	-14	-6.20	11.20
3	3	14	-14.30	13.80
-3	3	-14	-13.50	16.90
-3	3	-14	-0.90	12.40
-3	3	-14	-1.90	12.40
3	3	14	2.30	8.70
-3	3	-14	-7.00	9.90
-4	-3	-14	-1.30	12.00

-4	-3	-14	15.20	17.80
-4	-3	-14	42.50	13.60
-4	-3	-14	-1.80	20.30
4	3	14	32.30	19.10
-4	3	-14	-12.10	10.00
-4	3	-14	-13.20	11.20
-4	3	-14	19.30	13.70
-4	3	-14	-1.60	21.40
4	3	14	15.40	8.80
-5	-3	-14	6.50	11.30
-5	-3	-14	-0.30	11.40
-5	-3	-14	-17.30	22.00
-5	-3	-14	-19.50	22.10
-5	3	-14	-1.90	12.50
-5	3	-14	16.00	22.20
5	3	14	9.70	9.40
-6	-3	-14	-25.00	11.40
-6	-3	-14	11.60	12.20
-6	-3	-14	2.50	24.50
-6	-3	-14	17.00	24.10
-6	3	-14	3.80	10.60
-6	3	-14	-11.20	21.10
-6	3	-14	-1.20	13.50
6	3	14	22.70	10.40
6	-4	-14	-3.60	14.90
6	-4	-14	35.70	17.40
-6	-4	14	61.39	26.00
-6	4	14	-47.30	21.30
-6	4	14	-30.40	20.50
6	4	-14	-5.00	12.80
-5	-4	14	-17.00	21.10

5	-4	-14	-12.90	12.20
5	-4	-14	5.10	15.80
-5	-4	14	9.40	10.10
-5	4	14	17.40	20.50
-5	4	14	6.50	16.80
5	4	-14	19.00	11.50
-5	4	14	25.40	11.50
-4	-4	14	15.70	9.10
4	-4	-14	19.30	17.70
4	-4	-14	19.80	13.40
-4	-4	14	20.50	20.80
-4	4	14	-11.10	18.00
-4	4	14	16.10	10.90
-4	4	14	7.50	18.70
4	4	-14	19.60	11.80
-3	-4	14	40.70	9.70
3	-4	-14	51.99	17.60
3	-4	-14	30.30	12.00
-3	-4	14	33.00	24.10
3	4	-14	30.90	12.00
-3	4	14	47.40	14.40
-3	4	14	22.20	21.70
-2	-4	14	6.30	6.10
-2	-4	14	-0.20	18.10
2	-4	-14	4.10	10.20
2	-4	-14	-13.40	10.90
2	4	-14	8.30	18.40
-2	4	14	10.50	18.00
-2	4	14	0.10	7.80
-2	4	14	-9.20	18.70
1	-4	-14	31.40	11.80

-1	-4	14	-4.10	16.80
1	-4	-14	22.70	15.30
1	4	-14	29.70	10.90
-1	4	14	20.40	17.20
-1	4	14	17.50	10.40
0	-4	-14	-11.70	24.40
0	-4	-14	1.40	8.10
0	-4	14	-19.60	16.60
0	-4	-14	-3.20	11.50
0	4	-14	-9.10	8.00
0	4	14	-9.10	13.80
0	4	14	7.90	8.70
-1	-4	-14	-1.70	12.40
-1	-4	-14	19.90	20.30
-1	-4	-14	24.20	21.00
-1	-4	-14	6.30	10.40
-1	4	-14	2.80	16.60
-1	4	-14	5.00	9.50
1	4	14	-8.80	11.90
1	4	14	-6.80	19.00
1	4	14	-10.70	8.60
-2	-4	-14	-5.30	8.60
-2	-4	-14	11.00	19.70
-2	-4	-14	-1.10	21.50
-2	4	-14	15.90	11.90
-2	4	-14	5.60	9.80
-2	4	-14	-8.00	19.20
2	4	14	10.10	15.60
2	4	14	14.80	9.80
-3	-4	-14	2.40	11.80
-3	-4	-14	-5.00	18.40

-3	-4	-14	18.30	20.30
-3	-4	-14	0.80	12.30
-3	4	-14	12.30	11.40
3	4	14	22.90	10.70
3	4	14	-6.30	16.40
-3	4	-14	12.00	11.40
-3	4	-14	14.50	19.90
-3	4	-14	14.60	11.90
-4	-4	-14	-12.20	20.30
-4	-4	-14	29.30	21.10
-4	-4	-14	11.40	11.40
-4	-4	-14	-10.50	11.90
-4	4	-14	8.50	12.40
-4	4	-14	22.80	19.00
4	4	14	15.90	20.20
-4	4	-14	29.60	12.80
-4	4	-14	11.10	11.30
4	4	14	7.70	9.50
-5	-4	-14	-6.30	13.40
-5	-4	-14	-20.90	12.10
-5	-4	-14	-10.90	20.50
-5	-4	-14	-4.50	21.70
-5	4	-14	-5.30	11.00
-5	4	-14	-2.90	11.40
-5	4	-14	7.90	22.90
5	4	14	-1.20	19.50
5	4	14	4.80	10.60
-6	-4	-14	-11.00	11.60
-6	-4	-14	18.10	23.80
-6	-4	-14	28.60	24.20
-6	-4	-14	11.30	13.20

6	4	14	-6.50	10.40
-6	4	-14	-24.50	23.00
-6	4	-14	-1.50	12.90
-6	4	-14	-7.70	11.50
6	-5	-14	25.00	17.00
-6	-5	14	27.80	28.20
6	-5	-14	19.50	15.70
-6	5	14	5.90	22.40
6	5	-14	10.30	14.70
-6	5	14	1.40	22.90
5	-5	-14	-0.20	13.10
5	-5	-14	29.20	19.20
-5	-5	14	9.00	10.50
-5	-5	14	37.60	25.60
-5	5	14	-14.60	19.50
-5	5	14	-2.20	11.40
5	5	-14	-4.80	12.60
-5	5	14	21.80	23.30
4	-5	-14	-12.20	11.40
-4	-5	14	36.60	25.90
4	-5	-14	9.30	13.50
-4	-5	14	15.70	9.00
4	5	-14	8.60	12.50
-4	5	14	-13.30	10.20
-4	5	14	-35.70	19.20
-4	5	14	11.30	21.50
-3	-5	14	-17.00	6.30
-3	-5	14	14.70	20.90
3	-5	-14	-8.90	12.00
3	-5	-14	7.00	11.60
-3	5	14	-1.40	11.00

3	5	-14	12.80	10.20
-3	5	14	-9.30	16.20
-3	5	14	-20.20	18.10
-2	-5	14	-6.30	4.90
2	-5	-14	0.30	10.40
2	-5	-14	-2.10	12.10
-2	-5	14	-14.80	17.10
-2	5	14	10.00	20.20
-2	5	14	8.90	8.80
2	5	-14	-7.90	9.60
-2	5	14	-5.00	17.00
1	-5	-14	11.30	19.70
-1	-5	14	30.10	20.60
1	-5	-14	19.80	10.80
1	-5	-14	15.90	13.60
-1	5	14	-10.70	16.20
-1	5	14	-6.50	18.60
-1	5	14	-1.30	8.80
1	5	-14	17.40	10.50
0	-5	-14	-3.00	9.40
0	-5	-14	6.90	13.50
0	5	14	7.80	9.30
0	5	-14	18.60	11.00
0	5	14	1.90	17.50
0	5	14	13.60	15.70
1	-5	14	-13.00	16.90
-1	-5	-14	-10.00	18.90
-1	-5	-14	43.60	14.40
-1	-5	-14	6.40	20.50
-1	-5	-14	18.80	12.70
1	5	14	25.80	11.40

1	5	14	14.40	19.90
1	5	14	25.90	18.70
-1	5	-14	-27.70	17.50
-1	5	-14	5.60	11.80
-1	5	-14	21.30	11.90
-2	-5	-14	-4.00	9.10
-2	-5	-14	-0.50	19.40
-2	-5	-14	-23.40	21.10
-2	-5	-14	-4.90	12.60
-2	5	-14	2.00	10.10
-2	5	-14	-3.70	20.10
2	5	14	17.00	11.20
2	5	14	-0.50	14.90
-2	5	-14	-16.60	8.80
-3	-5	-14	-13.50	19.40
-3	-5	-14	3.20	11.40
-3	-5	-14	4.20	21.30
-3	-5	-14	21.20	13.70
-3	5	-14	-12.00	11.40
-3	5	-14	11.90	10.20
3	5	14	10.60	10.70
-3	5	-14	-4.80	17.90
-3	5	-14	-6.70	9.80
3	5	14	13.70	17.80
-4	-5	-14	5.80	12.00
-4	-5	-14	-11.60	23.00
-4	-5	-14	15.70	18.60
4	5	14	7.30	18.20
4	5	14	0.10	10.00
-4	5	-14	-0.90	9.40
-4	5	-14	9.40	11.20

-4	5	-14	-25.80	13.00
-4	5	-14	13.70	21.20
-5	-5	-14	21.80	29.70
-5	-5	-14	-5.00	20.20
-5	-5	-14	14.50	24.40
-5	-5	-14	8.40	13.70
5	5	14	-16.40	17.40
-5	5	-14	6.60	11.00
5	5	14	19.80	11.50
-5	5	-14	6.40	25.40
-6	-5	-14	-17.10	13.90
-6	-5	-14	-21.00	27.30
-6	-5	-14	-36.70	26.80
-6	-5	-14	-2.60	13.80
6	5	14	14.20	11.50
-6	5	-14	-47.60	24.30
-6	5	-14	-6.50	12.20
6	-6	-14	-32.60	16.80
-6	-6	14	0.60	29.50
-6	-6	14	2.70	12.80
-6	6	14	-14.90	23.70
-6	6	14	7.50	12.30
-6	6	14	-38.60	22.80
-5	-6	14	10.10	11.50
-5	-6	14	44.40	29.70
5	-6	-14	-22.90	16.60
-5	6	14	-3.40	22.00
5	6	-14	7.80	11.90
-5	6	14	-7.60	10.70
4	-6	-14	5.20	12.30
-4	-6	14	20.40	24.40

4	−6	−14	10.60	15.50
−4	−6	14	5.70	8.40
−4	6	14	−18.50	19.70
−4	6	14	10.10	10.50
−4	6	14	5.00	20.70
4	6	−14	−14.90	10.00
−3	−6	14	20.80	23.90
3	−6	−14	28.80	13.40
3	−6	−14	21.40	15.20
3	6	−14	21.80	11.80
−3	6	14	−2.00	20.00
−3	6	14	24.50	12.30
−3	6	14	7.70	18.80
2	−6	−14	7.70	10.40
−2	−6	14	11.00	21.60
2	−6	−14	4.40	11.30
−2	6	14	−10.80	17.70
2	6	−14	15.50	12.00
−2	6	14	−6.40	19.00
−2	6	14	−2.50	8.50
1	−6	−14	−13.50	10.90
1	−6	−14	2.10	16.00
1	−6	−14	30.30	21.40
−1	6	14	−34.00	15.10
1	6	−14	22.10	11.30
−1	6	14	43.60	22.30
−1	6	14	22.40	11.60
0	−6	−14	−6.70	9.10
0	−6	−14	−16.70	10.60
0	−6	14	2.10	18.50
0	−6	−14	40.60	19.80

0	6	14	-6.60	10.60
0	6	14	3.90	19.80
0	6	14	15.40	17.60
0	6	-14	-0.70	9.90
-1	-6	-14	2.00	11.80
-1	-6	-14	29.20	21.90
-1	-6	-14	2.00	12.00
1	6	14	8.60	17.50
1	6	14	11.60	11.90
-1	6	-14	11.00	11.20
-1	6	-14	2.70	9.60
-1	6	-14	-30.40	19.60
-2	-6	-14	17.50	15.50
-2	-6	-14	11.20	18.80
-2	-6	-14	-25.90	20.20
-2	-6	-14	-1.50	10.10
-2	6	-14	-8.50	9.10
-2	6	-14	2.10	10.00
-2	6	-14	7.60	23.10
2	6	14	-5.40	18.00
2	6	14	4.80	12.30
-3	-6	-14	-5.20	20.80
-3	-6	-14	-21.50	12.40
-3	-6	-14	-28.60	21.50
-3	-6	-14	-8.20	11.30
-3	6	-14	9.00	10.20
-3	6	-14	-17.80	21.40
-3	6	-14	1.60	8.40
3	6	14	2.00	16.50
3	6	14	5.30	10.80
-3	6	-14	37.20	14.60

-4	-6	-14	-24.00	21.40
-4	-6	-14	24.70	14.90
-4	-6	-14	-0.90	25.50
-4	-6	-14	-0.40	13.20
-4	6	-14	-4.00	23.10
-4	6	-14	-6.00	7.70
-4	6	-14	0.50	12.60
4	6	14	16.50	12.70
4	6	14	-8.70	18.60
-4	6	-14	11.90	10.90
-5	-6	-14	30.90	24.80
-5	-6	-14	42.30	28.00
-5	-6	-14	0.60	12.30
-5	-6	-14	24.30	14.40
5	6	14	9.10	12.40
-5	6	-14	-3.20	23.20
5	6	14	21.90	22.00
-5	6	-14	16.10	11.90
5	-7	-14	33.90	18.80
-5	-7	14	11.80	29.10
5	-7	-14	21.30	15.10
-5	7	14	-49.20	20.80
-5	7	14	26.10	11.80
-5	7	14	-40.20	22.10
5	7	-14	4.60	12.60
4	-7	-14	4.30	19.30
4	-7	-14	4.80	12.40
-4	-7	14	30.10	29.60
-4	7	14	-6.50	10.20
-4	7	14	9.70	22.00
4	7	-14	4.70	11.80

-4	7	14	0.40	25.00
-3	-7	14	-18.90	28.00
3	-7	-14	-2.70	10.50
3	-7	-14	2.10	15.30
-3	7	14	10.00	12.20
-3	7	14	8.50	20.50
-3	7	14	-8.10	20.90
3	7	-14	-15.20	10.20
2	-7	-14	-15.50	15.30
2	-7	-14	-0.10	11.50
2	-7	-14	31.00	22.80
-2	7	14	-1.60	10.40
-2	7	14	3.60	20.40
-2	7	14	20.90	20.80
1	-7	-14	-9.10	20.50
1	-7	-14	11.90	11.10
1	-7	-14	45.70	18.50
1	7	-14	29.30	12.70
-1	7	14	13.00	23.50
-1	7	14	22.40	20.70
-1	7	14	-2.30	10.40
0	-7	-14	10.30	25.40
0	-7	-14	-18.40	19.50
0	-7	-14	-15.10	11.00
0	-7	-14	4.90	14.10
0	7	14	-12.40	18.10
0	7	14	2.80	10.40
0	7	14	32.50	21.00
0	7	-14	-1.60	10.30
-1	-7	-14	-24.00	22.50
-1	-7	-14	21.60	11.20

-1	-7	-14	39.70	16.40
1	7	14	15.70	21.00
-1	7	-14	-5.10	18.90
-1	7	-14	1.70	10.70
1	7	14	7.20	10.50
-1	7	-14	24.10	9.90
-2	-7	-14	-9.20	12.00
-2	-7	-14	-6.40	23.50
-2	-7	-14	-15.80	21.50
-2	-7	-14	-17.70	13.50
-2	7	-14	12.40	19.80
-2	7	-14	6.40	10.40
2	7	14	-1.40	12.40
2	7	14	31.90	22.10
-2	7	-14	11.90	6.70
-3	-7	-14	-10.70	20.80
-3	-7	-14	0.90	13.80
-3	-7	-14	-8.00	11.90
-3	-7	-14	26.60	28.10
-3	7	-14	29.10	20.80
3	7	14	16.30	13.70
-3	7	-14	-0.60	11.00
3	7	14	-2.90	21.20
-4	-7	-14	12.80	26.90
-4	-7	-14	11.00	14.10
-4	-7	-14	-36.10	14.20
-4	-7	-14	40.10	26.00
4	7	14	0.00	13.00
4	7	14	20.50	21.90
-4	7	-14	9.10	28.90
-4	7	-14	1.20	10.70

-4	7	-14	-11.90	14.90
4	-8	-14	25.30	13.60
4	-8	-14	-13.20	17.10
-4	8	14	-6.20	25.60
-4	8	14	2.70	25.10
4	8	-14	19.10	13.80
-4	8	14	3.40	11.90
3	-8	-14	18.60	19.90
3	-8	-14	40.60	25.00
3	-8	-14	16.90	15.10
-3	8	14	-4.00	21.80
-3	8	14	9.30	12.50
3	8	-14	26.00	12.00
2	-8	-14	-29.70	11.40
2	-8	-14	39.50	18.50
2	-8	-14	-14.20	19.50
-2	8	14	27.70	21.00
-2	8	14	21.10	26.00
-2	8	14	-14.10	20.00
1	-8	-14	22.40	21.50
1	-8	-14	-16.90	11.40
1	-8	-14	-16.40	15.00
1	8	-14	16.90	10.90
-1	8	14	-2.70	11.10
-1	8	14	-11.20	20.20
-1	8	14	-13.60	19.90
0	-8	-14	19.30	25.20
0	-8	-14	-8.30	23.70
0	-8	-14	13.40	12.80
0	-8	-14	16.60	14.70
0	8	14	16.10	12.10

0	8	-14	20.60	11.60
0	8	-14	-0.90	9.10
0	8	14	77.09	27.80
-1	-8	-14	-9.10	22.40
-1	-8	-14	-5.70	11.10
-1	-8	-14	-5.70	12.90
-1	-8	-14	-7.00	25.80
-1	8	-14	15.00	19.60
1	8	14	7.90	24.30
-1	8	-14	6.50	11.20
1	8	14	-11.60	9.30
-2	-8	-14	31.80	26.10
-2	-8	-14	9.90	15.60
-2	-8	-14	43.90	24.20
2	8	14	19.70	22.30
-2	8	-14	24.20	23.70
-2	8	-14	6.80	10.80
2	8	14	11.80	12.70
-3	-8	-14	24.30	18.70
-3	-8	-14	-30.00	32.60
-3	-8	-14	1.90	23.80
-3	-8	-14	1.10	15.00
-3	8	-14	9.10	24.30
-3	8	-14	0.90	19.80
-3	8	-14	15.40	12.60
3	8	14	-36.70	20.40
3	8	14	-7.50	12.60
-4	-8	-14	-5.30	25.90
-4	-8	-14	-24.10	28.50
-4	-8	-14	-19.80	15.20
-4	-8	-14	-5.50	13.50

-4	8	-14	-25.90	23.50
-4	8	-14	27.90	22.50
4	8	14	16.10	14.90
4	8	14	16.70	23.80
3	-9	-14	17.00	14.20
3	-9	-14	36.10	28.20
3	-9	-14	-23.00	17.50
-3	9	14	-22.70	24.10
3	9	-14	23.40	12.20
-3	9	14	1.90	13.00
-3	9	14	-3.40	24.70
2	-9	-14	-23.10	16.50
2	-9	-14	20.50	24.00
2	-9	-14	8.20	12.50
-2	9	14	16.10	14.50
-2	9	14	43.10	26.40
-2	9	14	-1.10	24.60
2	9	-14	6.00	12.60
1	-9	-14	-18.60	22.90
1	-9	-14	4.50	16.10
-1	9	14	-8.00	26.00
-1	9	14	-19.30	13.10
1	9	-14	-1.10	11.80
0	-9	-14	-4.90	11.60
0	-9	-14	0.00	27.50
0	-9	-14	-4.80	21.30
0	9	-14	4.90	12.10
0	9	-14	22.60	20.70
0	9	14	-2.50	12.40
0	9	14	39.50	45.60
-1	-9	-14	2.80	10.90

-1	-9	-14	-19.40	25.90
-1	-9	-14	19.20	17.90
-1	-9	-14	49.80	24.20
1	9	14	0.70	22.10
-1	9	-14	-7.20	20.80
-1	9	-14	-18.30	11.10
1	9	14	2.10	14.60
-2	-9	-14	-24.90	29.20
-2	-9	-14	-25.90	26.70
-2	-9	-14	23.20	17.70
-2	-9	-14	8.80	12.90
2	9	14	10.00	14.10
-2	9	-14	-5.90	21.30
2	9	14	-6.50	24.40
6	0	-15	-15.10	14.50
-6	0	15	-12.70	23.10
-6	0	15	-6.20	25.50
6	0	-15	1.50	12.50
-5	0	15	-13.00	17.30
-5	0	15	-2.30	11.30
5	0	-15	24.00	16.90
5	0	-15	18.20	14.30
-5	0	15	14.30	22.80
-4	0	15	-0.60	10.90
-4	0	15	6.70	16.10
-4	0	15	-4.50	19.80
4	0	-15	-12.50	14.60
4	0	-15	6.10	11.80
-3	0	15	-22.20	20.10
-3	0	15	0.40	14.40
-3	0	15	9.10	10.20

3	0	-15	24.40	13.60
3	0	-15	7.50	16.80
2	0	-15	7.50	10.80
-2	0	15	18.30	20.20
-2	0	15	5.90	9.00
-2	0	15	-7.10	12.60
2	0	-15	0.10	13.50
-1	0	15	-3.60	12.90
1	0	-15	16.90	13.10
-1	0	15	6.00	7.90
-1	0	15	-15.50	18.40
1	0	-15	-1.50	10.40
0	0	-15	-15.50	12.30
0	0	-15	5.30	17.20
0	0	15	2.80	19.10
0	0	-15	17.50	10.30
0	0	15	-2.30	8.40
0	0	15	-14.20	11.80
-1	0	-15	3.70	13.40
-1	0	-15	10.00	12.10
1	0	15	4.20	13.70
1	0	15	4.10	8.20
-1	0	-15	-22.70	15.70
-1	0	-15	7.30	16.50
1	0	15	19.30	22.20
2	0	15	-4.20	7.40
2	0	15	-12.70	13.10
-2	0	-15	14.80	20.90
-2	0	-15	2.80	17.00
-2	0	-15	9.20	13.00
-2	0	-15	-6.80	9.90

-3	0	-15	-6.60	17.00
-3	0	-15	41.40	14.70
-3	0	-15	36.60	16.10
3	0	15	53.09	10.40
-3	0	-15	41.10	24.30
-4	0	-15	8.40	12.90
-4	0	-15	-10.00	15.40
-4	0	-15	-3.80	24.90
-4	0	-15	-3.80	12.90
4	0	15	3.80	7.60
-5	0	-15	-9.80	11.50
-5	0	-15	1.50	12.20
-5	0	-15	3.80	17.80
5	0	15	7.00	8.10
-6	0	-15	-18.10	20.80
-6	0	-15	14.10	12.90
-6	0	-15	11.30	20.50
6	-1	-15	-5.40	14.40
-6	-1	15	20.80	27.60
6	-1	-15	10.40	17.50
-6	1	15	2.20	26.50
6	1	-15	-1.10	15.00
-6	1	15	-34.50	19.90
6	1	-15	4.90	15.80
-5	-1	15	6.20	22.20
5	-1	-15	26.60	12.60
-5	-1	15	1.50	11.60
5	-1	-15	12.30	18.90
-5	1	15	-12.50	11.10
5	1	-15	39.10	18.40
5	1	-15	30.90	13.30

-5	1	15	29.40	24.40
-5	1	15	39.40	19.60
4	-1	-15	11.10	11.80
-4	-1	15	5.70	10.80
4	-1	-15	12.60	17.20
-4	-1	15	-9.10	21.10
-4	1	15	-11.50	16.40
4	1	-15	26.70	12.20
4	1	-15	7.70	14.40
-4	1	15	-10.60	19.50
-4	1	15	-7.20	9.40
-3	-1	15	-15.90	19.50
3	-1	-15	15.40	11.00
-3	-1	15	12.00	9.70
-3	-1	15	7.40	12.80
3	-1	-15	28.90	17.20
3	1	-15	22.40	15.80
-3	1	15	15.30	15.50
-3	1	15	7.10	9.50
-3	1	15	-17.00	18.50
3	1	-15	8.10	11.70
-2	-1	15	4.50	8.40
2	-1	-15	15.00	13.80
-2	-1	15	-26.00	18.90
-2	-1	15	40.10	13.10
2	-1	-15	0.00	11.10
-2	1	15	-4.20	14.70
-2	1	15	-6.50	8.30
-2	1	15	2.60	21.00
2	1	-15	-1.70	13.40
2	1	-15	-11.60	10.30

1	-1	-15	31.70	11.50
-1	-1	15	49.00	14.40
-1	-1	15	31.90	21.60
1	-1	-15	8.50	14.90
-1	-1	15	1.70	7.70
-1	1	15	15.70	8.60
-1	1	15	15.20	14.40
-1	1	15	34.90	24.80
1	1	-15	5.80	13.70
1	1	-15	51.19	14.00
0	-1	-15	9.50	20.90
0	-1	-15	6.70	11.20
0	-1	-15	-13.50	8.90
0	-1	15	0.60	6.60
0	-1	15	13.20	25.10
0	-1	15	13.70	13.10
0	1	-15	-3.30	9.20
0	1	15	-0.80	8.00
0	1	15	17.60	14.60
0	1	15	-7.50	19.20
0	1	-15	-7.90	11.50
-1	-1	-15	6.10	10.50
1	-1	15	5.30	7.90
1	-1	15	17.40	21.60
-1	-1	-15	4.70	20.50
-1	-1	-15	3.80	16.70
1	1	15	30.00	15.80
-1	1	-15	1.70	16.60
-1	1	-15	-18.70	9.40
-1	1	-15	4.70	16.90
-1	1	-15	7.40	14.10

1	1	15	18.40	18.40
1	1	15	7.70	9.10
-2	-1	-15	-10.30	15.90
-2	-1	-15	11.90	23.70
-2	-1	-15	38.30	12.90
2	-1	15	5.30	6.60
-2	-1	-15	-13.30	10.40
-2	1	-15	18.60	12.30
2	1	15	-3.30	7.90
-2	1	-15	13.00	16.20
-2	1	-15	5.00	13.00
-2	1	-15	0.20	17.10
3	-1	15	1.60	6.30
-3	-1	-15	-9.60	10.50
-3	-1	-15	12.40	25.20
-3	-1	-15	-16.60	12.50
-3	-1	-15	5.50	19.30
-3	1	-15	-18.00	17.90
-3	1	-15	17.50	18.10
3	1	15	9.10	7.90
3	1	15	-12.10	14.60
-3	1	-15	-5.50	12.20
-3	1	-15	-2.50	10.20
-4	-1	-15	-29.80	12.70
-4	-1	-15	15.40	19.10
-4	-1	-15	-15.30	18.10
-4	-1	-15	-10.70	11.40
-4	1	-15	14.80	12.20
4	1	15	4.10	8.40
-4	1	-15	-12.00	11.80
-4	1	-15	14.10	24.40

-4	1	-15	6.40	15.50
-5	-1	-15	15.90	20.50
-5	-1	-15	44.70	33.70
-5	-1	-15	-23.30	11.30
-5	-1	-15	10.90	12.00
5	1	15	19.40	9.10
-5	1	-15	-0.50	39.80
-5	1	-15	19.70	13.90
-5	1	-15	20.70	17.30
-6	-1	-15	1.40	19.80
-6	-1	-15	3.20	14.10
-6	-1	-15	12.70	28.00
-6	1	-15	-10.10	18.40
6	1	15	13.70	10.60
-6	1	-15	-3.30	12.70
-6	1	-15	-18.00	23.20
6	-2	-15	-13.30	17.00
6	-2	-15	16.60	14.00
-6	-2	15	14.60	12.70
-6	-2	15	-10.60	25.90
-6	2	15	31.60	14.10
-6	2	15	35.70	27.00
-6	2	15	11.90	25.20
6	2	-15	11.30	12.90
6	2	-15	6.30	16.80
5	-2	-15	17.30	15.00
-5	-2	15	-23.20	22.60
5	-2	-15	37.80	21.80
-5	-2	15	-17.50	10.60
5	2	-15	-2.40	10.70
-5	2	15	9.80	12.10

5	2	-15	-12.10	15.00
-5	2	15	-15.30	21.60
-5	2	15	-41.90	17.90
-4	-2	15	2.00	9.90
4	-2	-15	-10.70	12.00
4	-2	-15	4.70	17.50
-4	-2	15	5.60	22.60
4	2	-15	-4.00	13.60
-4	2	15	6.20	9.60
-4	2	15	28.80	18.10
-4	2	15	6.70	22.60
4	2	-15	-0.70	13.00
-3	-2	15	-9.60	11.20
3	-2	-15	10.50	17.70
-3	-2	15	20.90	10.00
3	-2	-15	10.90	13.30
-3	-2	15	3.00	21.90
-3	2	15	-15.10	16.20
-3	2	15	9.00	9.50
3	2	-15	-1.80	11.90
3	2	-15	25.00	15.00
-3	2	15	-12.50	17.00
2	-2	-15	17.00	10.70
2	-2	-15	-14.00	12.40
-2	-2	15	11.20	23.40
-2	-2	15	11.50	8.50
-2	-2	15	7.00	11.50
2	2	-15	33.10	16.40
2	2	-15	8.40	11.40
-2	2	15	-13.00	15.10
-2	2	15	-0.80	9.40

-2	2	15	1.90	20.00
-1	-2	15	40.70	21.80
1	-2	-15	23.90	11.60
1	-2	-15	53.59	17.00
-1	-2	15	22.50	9.10
-1	2	15	25.40	10.90
-1	2	15	27.40	21.20
1	2	-15	36.80	17.20
-1	2	15	29.90	17.30
1	2	-15	33.80	13.30
0	-2	-15	30.80	21.00
0	-2	-15	5.00	11.30
0	-2	-15	35.30	15.60
0	-2	15	6.60	7.30
0	-2	15	-11.50	18.50
0	2	-15	13.40	11.00
0	2	15	35.90	22.00
0	2	15	13.90	9.30
0	2	-15	0.10	10.50
0	2	15	12.90	16.80
-1	-2	-15	-10.90	18.20
-1	-2	-15	9.00	11.20
-1	-2	-15	35.70	24.10
1	-2	15	-6.20	19.40
-1	-2	-15	0.60	9.60
1	2	15	0.00	8.20
-1	2	-15	8.10	10.40
1	2	15	-3.50	19.20
-1	2	-15	15.80	12.90
-1	2	-15	11.80	19.80
-1	2	-15	-6.40	14.20

2	-2	15	-3.00	5.60
-2	-2	-15	-1.60	12.30
-2	-2	-15	3.40	19.30
-2	-2	-15	-10.80	20.30
-2	-2	-15	-0.50	10.70
2	2	15	-12.60	13.80
-2	2	-15	-19.30	13.10
-2	2	-15	-29.50	11.20
2	2	15	-5.90	8.00
-2	2	-15	19.30	20.90
-2	2	-15	15.70	11.10
-3	-2	-15	24.40	20.40
-3	-2	-15	49.00	27.90
-3	-2	-15	48.50	16.40
-3	-2	-15	22.20	13.20
3	2	15	43.60	11.60
-3	2	-15	16.30	14.20
-3	2	-15	24.80	24.00
-3	2	-15	46.30	14.40
3	2	15	42.60	17.20
-3	2	-15	54.39	19.20
-4	-2	-15	-2.90	13.60
-4	-2	-15	-8.60	11.90
-4	-2	-15	-54.59	20.90
-4	-2	-15	2.50	20.50
-4	2	-15	5.10	11.90
-4	2	-15	15.30	13.20
-4	2	-15	7.30	22.10
4	2	15	15.60	8.60
-4	2	-15	-6.90	14.00
-5	-2	-15	-14.50	23.50

-5	-2	-15	20.10	12.90
-5	-2	-15	10.00	13.00
-5	2	-15	16.10	15.30
5	2	15	1.80	9.40
-5	2	-15	6.30	13.30
-5	2	-15	-24.90	24.60
6	-3	-15	-10.20	14.90
-6	-3	15	27.00	28.50
6	-3	-15	-26.20	20.10
-6	-3	15	32.80	14.40
-6	3	15	31.90	23.10
6	3	-15	-2.90	14.70
-6	3	15	19.90	14.30
-5	-3	15	-36.40	23.50
-5	-3	15	-5.60	11.30
5	-3	-15	-8.60	13.00
-5	3	15	1.00	10.80
-5	3	15	-3.90	18.50
-5	3	15	3.60	23.00
5	3	-15	2.40	11.90
-4	-3	15	11.40	22.50
4	-3	-15	21.00	14.30
4	-3	-15	-9.40	17.30
-4	3	15	36.60	25.40
-4	3	15	32.60	18.90
4	3	-15	13.40	11.70
-4	3	15	20.10	12.10
3	-3	-15	-12.40	12.90
-3	-3	15	19.50	9.80
-3	-3	15	-10.20	21.60
3	-3	-15	16.10	12.50

-3	3	15	-5.40	20.80
-3	3	15	-23.20	18.10
-3	3	15	-5.40	10.10
3	3	-15	28.60	12.70
2	-3	-15	28.80	17.10
-2	-3	15	27.70	9.00
-2	-3	15	-10.80	22.00
2	-3	-15	22.00	12.70
-2	3	15	61.29	19.80
-2	3	15	9.40	20.00
-2	3	15	12.80	10.30
2	3	-15	18.00	11.60
-1	-3	15	1.30	6.30
-1	-3	15	10.10	20.30
1	-3	-15	37.10	18.60
1	-3	-15	19.50	11.30
-1	3	15	12.40	19.40
-1	3	15	1.80	10.10
1	3	-15	17.70	13.50
1	3	-15	21.80	12.10
0	-3	-15	4.20	14.00
0	-3	-15	-9.20	10.50
0	-3	-15	-41.90	18.00
0	-3	15	-0.80	5.60
0	3	-15	5.40	13.40
0	3	15	1.50	15.00
0	3	15	13.10	23.50
0	3	15	2.90	10.20
0	3	-15	-0.20	10.70
-1	-3	-15	30.80	23.40
-1	-3	-15	8.40	12.40

1	-3	15	3.80	23.20
-1	-3	-15	-4.50	19.40
1	-3	15	10.10	4.00
-1	-3	-15	26.90	14.90
1	3	15	10.50	17.10
-1	3	-15	13.10	13.20
-1	3	-15	9.40	18.80
-1	3	-15	15.10	16.30
1	3	15	9.20	9.70
-2	-3	-15	38.40	15.40
-2	-3	-15	30.40	15.00
-2	-3	-15	19.90	23.60
-2	-3	-15	27.80	22.70
2	3	15	37.50	12.10
-2	3	-15	27.80	21.30
-2	3	-15	4.00	13.20
-2	3	-15	29.30	14.40
2	3	15	29.20	19.00
-2	3	-15	60.29	17.00
-3	-3	-15	-41.70	19.90
-3	-3	-15	-14.20	9.40
-3	-3	-15	15.20	22.60
-3	-3	-15	-15.30	14.60
3	3	15	-20.30	16.50
-3	3	-15	0.00	13.90
-3	3	-15	-20.40	12.90
-3	3	-15	-14.90	24.70
3	3	15	15.70	9.50
-4	-3	-15	12.90	21.60
-4	-3	-15	29.90	22.30
-4	-3	-15	10.80	14.40

-4	-3	-15	41.30	15.80
-4	3	-15	2.70	12.00
4	3	15	19.70	18.70
-4	3	-15	34.50	22.80
-4	3	-15	36.40	15.60
4	3	15	-5.30	9.70
-4	3	-15	-0.90	13.30
-5	-3	-15	0.00	22.70
-5	-3	-15	-15.00	19.60
-5	-3	-15	2.10	13.80
-5	-3	-15	20.70	14.30
-5	3	-15	13.10	12.60
5	3	15	7.00	11.10
-5	3	-15	10.00	15.30
-5	3	-15	18.80	26.10
5	-4	-15	-3.90	13.30
5	-4	-15	-12.20	17.20
-5	-4	15	22.40	12.40
-5	-4	15	-1.70	42.30
5	4	-15	3.10	13.60
-5	4	15	9.10	21.90
-5	4	15	-0.80	23.00
-5	4	15	-2.40	12.40
4	-4	-15	-9.50	16.10
-4	-4	15	24.20	24.50
4	-4	-15	-2.30	11.00
-4	-4	15	4.70	10.20
-4	4	15	-0.50	20.40
4	4	-15	14.80	12.20
-4	4	15	21.60	11.40
-4	4	15	11.20	23.40

-3	-4	15	12.10	8.40
3	-4	-15	42.20	15.60
-3	-4	15	16.20	25.40
3	-4	-15	8.70	11.50
3	4	-15	10.80	14.10
-3	4	15	15.50	22.50
-3	4	15	13.20	12.40
-3	4	15	33.80	21.20
2	-4	-15	-12.00	13.70
-2	-4	15	-1.60	24.40
-2	-4	15	9.00	7.10
2	-4	-15	8.60	10.70
-2	4	15	33.60	12.90
2	4	-15	-1.70	10.70
-2	4	15	24.00	21.70
-2	4	15	1.20	17.20
1	-4	-15	1.60	10.40
1	-4	-15	-6.20	11.90
1	-4	-15	-15.60	19.20
-1	-4	15	0.50	5.00
-1	4	15	-5.90	10.50
-1	4	15	17.70	16.80
-1	4	15	14.20	19.30
1	4	-15	-0.20	10.00
0	-4	-15	45.50	17.40
0	-4	-15	15.70	20.90
0	-4	15	2.30	22.10
0	-4	-15	0.40	10.30
0	4	15	35.40	12.40
0	4	15	21.20	25.80
0	4	15	25.20	18.60

-1	-4	-15	-4.30	22.40
-1	-4	-15	-12.40	11.80
-1	-4	-15	12.40	21.00
1	-4	15	13.00	20.80
-1	-4	-15	16.30	12.10
-1	4	-15	-26.30	12.10
-1	4	-15	11.40	20.80
1	4	15	-9.90	15.60
-1	4	-15	12.30	14.50
-1	4	-15	5.90	10.60
1	4	15	13.10	20.90
1	4	15	19.60	11.60
-2	-4	-15	22.50	12.60
-2	-4	-15	11.00	25.00
-2	-4	-15	-9.80	12.40
-2	-4	-15	-9.60	20.90
-2	4	-15	-16.60	12.90
2	4	15	-16.30	10.00
2	4	15	19.20	15.40
-2	4	-15	8.70	13.50
-2	4	-15	13.90	12.40
-2	4	-15	10.90	23.80
-3	-4	-15	31.30	14.10
-3	-4	-15	-3.20	13.80
-3	-4	-15	-21.30	22.30
-3	-4	-15	18.10	22.90
-3	4	-15	14.30	13.00
3	4	15	35.40	12.60
-3	4	-15	-10.20	19.50
-3	4	-15	13.70	14.10
3	4	15	-17.90	15.40

-3	4	-15	5.20	14.90
-4	-4	-15	-7.70	15.10
-4	-4	-15	51.79	29.10
-4	-4	-15	4.40	12.20
4	4	15	3.20	10.30
-4	4	-15	-4.00	13.70
-4	4	-15	16.30	13.00
4	4	15	40.60	21.00
-5	-4	-15	16.20	26.60
-5	-4	-15	-6.20	26.70
-5	-4	-15	1.40	13.10
-5	-4	-15	-3.30	14.70
5	4	15	9.60	11.90
5	4	15	-10.80	20.30
-5	4	-15	6.30	12.90
-5	4	-15	-13.30	11.90
-5	4	-15	52.99	28.50
-5	-5	15	8.80	29.60
5	-5	-15	26.00	12.10
5	-5	-15	4.20	17.20
-5	5	15	-4.80	25.10
5	5	-15	6.90	14.30
-5	5	15	9.60	13.00
-5	5	15	-9.60	23.70
4	-5	-15	-3.60	19.00
-4	-5	15	21.40	10.10
4	-5	-15	-18.20	23.30
-4	-5	15	15.90	29.60
-4	5	15	4.80	24.50
-4	5	15	-29.60	20.40
4	5	-15	-1.70	14.00

-3	-5	15	0.60	7.60
-3	-5	15	-6.70	27.60
3	-5	-15	5.20	13.50
3	-5	-15	-14.10	16.60
-3	5	15	2.70	10.80
-3	5	15	-14.80	20.80
3	5	-15	-1.70	11.20
-3	5	15	38.40	22.90
-2	-5	15	28.40	24.60
2	-5	-15	17.30	14.50
2	-5	-15	7.60	10.30
-2	5	15	3.30	19.30
2	5	-15	22.50	12.40
-2	5	15	-7.70	20.50
-2	5	15	2.90	13.10
1	-5	-15	22.90	11.40
1	-5	-15	3.50	16.10
-1	-5	15	-17.80	22.90
1	-5	-15	24.50	21.00
-1	5	15	19.90	22.70
1	5	-15	16.20	11.50
-1	5	15	35.90	18.40
-1	5	15	3.10	12.70
0	-5	-15	2.40	12.30
0	-5	-15	9.60	13.40
0	-5	-15	11.60	24.10
0	-5	15	-9.20	21.10
0	5	15	3.00	23.60
0	5	15	-3.90	16.40
0	5	15	-4.70	10.20
0	5	-15	10.30	11.30

-1	-5	-15	13.40	13.50
-1	-5	-15	19.10	15.90
-1	-5	-15	32.60	25.50
-1	-5	-15	-16.10	20.40
1	5	15	26.50	19.80
1	5	15	12.70	10.90
-1	5	-15	22.60	13.50
-1	5	-15	4.90	11.30
-1	5	-15	-16.20	18.20
-2	-5	-15	13.10	24.10
-2	-5	-15	7.00	14.10
-2	-5	-15	27.80	23.90
-2	-5	-15	-11.30	11.30
-2	5	-15	43.80	14.50
-2	5	-15	16.60	12.80
2	5	15	-0.10	20.00
-2	5	-15	3.40	12.40
2	5	15	32.30	13.40
-2	5	-15	42.40	24.90
-3	-5	-15	22.80	15.50
-3	-5	-15	9.70	24.90
-3	-5	-15	8.20	12.80
-3	-5	-15	-4.10	24.30
3	5	15	-8.80	11.10
3	5	15	12.00	20.60
-3	5	-15	2.60	11.30
-3	5	-15	-0.70	14.90
-3	5	-15	-6.20	11.20
-3	5	-15	5.70	22.10
-4	-5	-15	-1.70	26.20
-4	-5	-15	7.40	15.30

-4	-5	-15	-0.40	26.10
-4	-5	-15	-12.60	14.70
-4	5	-15	-8.90	23.00
4	5	15	9.40	12.10
-4	5	-15	8.50	11.60
-4	5	-15	23.30	13.40
-4	5	-15	9.60	14.70
4	5	15	2.30	18.90
-5	-5	-15	-17.50	24.90
4	-6	-15	11.00	18.30
4	-6	-15	4.70	14.40
-4	-6	15	-14.70	29.10
4	6	-15	-3.10	12.50
-4	6	15	-7.00	11.50
-4	6	15	5.40	23.80
-3	-6	15	-26.50	27.40
3	6	-15	16.60	12.30
-3	6	15	5.60	23.50
-3	6	15	20.00	22.90
2	-6	-15	17.40	21.50
2	-6	-15	25.00	19.10
2	-6	-15	20.10	12.50
2	6	-15	22.90	12.60
-2	6	15	-15.60	21.40
-2	6	15	6.80	20.50
-2	6	15	3.00	12.70
1	-6	-15	2.40	10.90
1	-6	-15	-36.50	21.30
1	-6	-15	-1.30	16.40
-1	6	15	4.20	18.40
-1	6	15	4.80	11.40

1	6	-15	-13.10	10.60
-1	6	15	15.80	25.90
0	-6	-15	50.89	21.10
0	-6	-15	5.50	11.70
0	-6	-15	37.80	17.60
0	6	15	0.10	13.90
0	6	-15	20.80	12.10
0	6	15	6.70	22.90
0	6	15	4.10	21.10
-1	-6	-15	-10.70	14.70
-1	-6	-15	-6.80	20.90
-1	-6	-15	25.70	25.80
-1	-6	-15	15.10	12.90
-1	6	-15	-2.10	11.00
-1	6	-15	-15.50	18.70
1	6	15	26.80	20.90
-1	6	-15	3.70	11.50
1	6	15	1.90	12.10
-2	-6	-15	25.60	25.40
-2	-6	-15	19.70	23.80
-2	-6	-15	16.40	14.70
-2	-6	-15	27.50	15.70
-2	6	-15	31.30	24.80
2	6	15	15.70	23.30
2	6	15	18.90	13.80
-2	6	-15	3.80	12.30
-3	-6	-15	-22.10	14.80
-3	-6	-15	-15.80	12.00
-3	-6	-15	27.10	24.20
-3	-6	-15	-4.20	25.50
-3	6	-15	-41.30	15.00

-3	6	-15	3.90	10.30
3	6	15	19.10	22.10
3	6	15	-2.90	11.60
-3	6	-15	1.00	12.70
-4	-6	-15	4.30	27.60
-4	-6	-15	23.90	16.10
-4	-6	-15	10.20	27.30
-4	-6	-15	15.20	14.60
4	6	15	-37.80	19.50
-4	6	-15	-8.50	26.30
4	6	15	22.40	14.60
-4	6	-15	17.40	12.80
-4	6	-15	-3.00	9.60
-4	7	15	26.70	26.30
3	-7	-15	6.90	17.20
3	-7	-15	-27.30	12.40
-3	7	15	1.50	10.20
-3	7	15	24.60	24.10
-3	7	15	10.50	24.80
3	7	-15	28.10	14.10
2	-7	-15	3.10	11.70
2	-7	-15	48.60	23.00
2	7	-15	27.30	12.50
-2	7	15	32.00	27.10
-2	7	15	27.30	22.20
-2	7	15	34.10	14.60
1	-7	-15	25.70	16.80
1	-7	-15	-2.40	24.00
1	7	-15	12.60	13.20
-1	7	15	-7.60	23.60
-1	7	15	-8.60	19.30

0	-7	-15	-34.20	23.60
0	-7	-15	-28.50	14.60
0	-7	-15	6.50	29.70
0	7	15	8.50	21.80
0	7	-15	7.10	11.90
0	7	-15	-12.80	21.90
0	7	-15	-6.80	11.60
0	7	15	-11.70	12.10
0	7	15	10.70	23.70
-1	-7	-15	60.49	25.90
-1	-7	-15	42.80	39.40
-1	-7	-15	19.70	14.50
-1	-7	-15	28.70	15.60
1	7	15	35.60	13.10
-1	7	-15	21.00	11.50
-1	7	-15	-17.50	12.70
-1	7	-15	27.20	28.40
1	7	15	21.70	20.50
-2	-7	-15	17.00	15.60
-2	-7	-15	14.20	16.50
-2	-7	-15	26.40	28.10
-2	-7	-15	26.10	26.70
-2	7	-15	-6.80	12.40
2	7	15	33.50	38.50
-2	7	-15	19.40	9.00
-3	-7	-15	-4.10	28.10
-3	-7	-15	8.10	16.00
-3	-7	-15	-15.10	24.20
-3	-7	-15	-8.50	14.00
3	7	15	-1.60	20.00
-3	7	-15	37.20	16.70

-3	7	-15	10.60	12.70
-3	7	-15	12.90	24.30
3	7	15	10.50	13.60
2	-8	-15	28.90	18.80
2	-8	-15	3.70	13.30
2	-8	-15	-31.80	23.40
-2	8	15	21.00	26.30
2	8	-15	12.60	12.40
-2	8	15	4.40	14.10
1	-8	-15	-22.20	13.80
1	-8	-15	-16.50	22.80
1	-8	-15	-8.90	13.00
-1	8	15	-22.70	23.70
-1	8	15	-16.40	11.30
1	8	-15	9.10	12.70
0	-8	-15	19.70	25.60
0	-8	-15	6.00	11.30
0	-8	-15	46.90	38.50
0	8	-15	8.10	31.50
0	8	15	-12.80	40.30
0	8	15	-11.60	13.20
0	8	-15	28.00	13.50
-1	-8	-15	-20.40	23.90
-1	-8	-15	7.60	15.80
-1	-8	-15	1.70	28.40
-1	-8	-15	-20.00	13.30
-1	8	-15	2.20	13.80
1	8	15	29.50	25.50
1	8	15	9.00	14.20
-1	8	-15	7.20	28.20
-2	-8	-15	37.10	25.50

-2	-8	-15	55.39	29.20
-2	-8	-15	-18.10	17.60
-2	-8	-15	11.10	14.60
2	8	15	47.00	26.50
-2	8	-15	6.90	28.80
-2	8	-15	-28.70	12.70
2	8	15	13.80	15.50
5	0	-16	-18.80	15.90
-5	0	16	14.60	12.60
-5	0	16	-8.20	26.40
5	0	-16	-4.00	13.10
-4	0	16	-9.90	19.20
-4	0	16	8.80	12.50
-4	0	16	-19.50	23.50
4	0	-16	-18.10	30.10
4	0	-16	-13.20	14.10
-3	0	16	-7.80	22.50
3	0	-16	8.20	18.50
3	0	-16	2.10	13.40
-3	0	16	12.30	16.60
-3	0	16	-4.10	10.10
-2	0	16	-6.70	9.00
2	0	-16	40.10	17.90
-2	0	16	6.10	23.20
2	0	-16	9.10	13.60
-2	0	16	-7.40	15.80
-1	0	16	9.90	15.60
1	0	-16	-2.30	11.70
-1	0	16	-1.00	10.00
1	0	-16	14.30	15.10
-1	0	16	-32.60	21.50

0	0	16	-4.00	25.10
0	0	16	-14.30	8.90
0	0	-16	-0.70	10.50
0	0	16	-16.70	15.90
0	0	-16	-6.90	14.40
0	0	-16	36.30	27.10
1	0	16	-5.10	8.60
1	0	16	11.00	14.90
-1	0	-16	16.10	22.50
-1	0	-16	-24.50	18.20
-1	0	-16	9.50	15.00
1	0	16	10.40	23.10
-1	0	-16	-12.50	11.10
-2	0	-16	-9.60	19.10
-2	0	-16	-8.30	11.30
2	0	16	9.60	8.60
-2	0	-16	47.30	25.10
-2	0	-16	-0.80	14.50
3	0	16	10.00	9.10
-3	0	-16	-7.30	18.90
-3	0	-16	6.60	13.10
-3	0	-16	-4.40	22.00
-3	0	-16	10.30	15.00
4	0	16	0.40	8.90
-4	0	-16	-6.10	19.60
-4	0	-16	-24.50	26.30
-4	0	-16	-0.60	15.50
-4	0	-16	-7.80	13.20
-5	-1	16	-8.20	11.80
5	-1	-16	6.80	19.30
-5	-1	16	-24.60	27.50

5	-1	-16	17.20	15.20
5	1	-16	-7.60	16.90
-5	1	16	-12.80	12.10
5	1	-16	19.80	15.00
-5	1	16	-37.20	21.30
-5	1	16	11.50	25.70
-4	-1	16	-3.80	11.90
-4	-1	16	-23.20	26.60
-4	1	16	39.40	21.40
4	1	-16	-15.90	14.40
-4	1	16	0.00	22.40
-4	1	16	22.00	11.50
4	1	-16	3.00	32.00
-3	-1	16	1.40	10.60
3	-1	-16	2.30	14.10
3	-1	-16	18.20	15.90
-3	-1	16	0.70	15.60
-3	1	16	-43.70	15.20
-3	1	16	-0.60	11.40
-3	1	16	-6.10	20.40
3	1	-16	-36.30	11.90
3	1	-16	4.10	12.40
2	-1	-16	11.40	18.20
-2	-1	16	-37.20	20.90
2	-1	-16	-10.00	11.90
-2	-1	16	1.30	14.30
-2	-1	16	5.70	9.60
2	1	-16	-9.10	14.30
-2	1	16	-28.20	19.20
-2	1	16	-17.60	16.50
2	1	-16	5.00	12.10

-2	1	16	-2.50	10.40
1	-1	-16	1.10	10.50
-1	-1	16	-22.50	21.80
-1	-1	16	-1.90	8.90
-1	-1	16	25.90	14.40
1	-1	-16	13.50	17.10
-1	1	16	1.50	9.90
1	1	-16	6.00	12.50
1	1	-16	11.10	14.40
-1	1	16	-4.60	16.10
0	-1	-16	-9.80	11.40
0	-1	16	-8.90	7.60
0	-1	-16	17.10	15.90
0	-1	16	15.30	25.00
0	-1	16	-4.10	14.00
0	-1	-16	-2.50	20.80
0	1	16	-20.10	24.20
0	1	16	27.10	15.70
0	1	16	18.30	10.20
0	1	-16	9.80	12.40
0	1	-16	18.80	15.00
0	1	-16	1.50	18.60
-1	-1	-16	3.60	10.90
-1	-1	-16	-6.30	19.80
-1	-1	-16	-17.20	17.80
-1	-1	-16	-31.70	13.30
1	-1	16	-21.20	7.20
1	-1	16	2.30	23.90
1	1	16	-0.40	9.20
-1	1	-16	-29.60	17.90
-1	1	-16	-15.50	13.80

-1	1	-16	-0.90	16.60
-1	1	-16	-22.20	10.50
1	1	16	-3.70	18.30
1	1	16	6.90	21.50
-2	-1	-16	1.60	19.90
-2	-1	-16	4.70	13.10
-2	-1	-16	-7.40	14.30
-2	-1	-16	-26.00	25.30
2	-1	16	2.10	8.00
-2	1	-16	-6.10	17.10
2	1	16	10.50	17.50
-2	1	-16	-7.50	19.00
-2	1	-16	-1.90	11.80
3	-1	16	7.20	7.50
-3	-1	-16	-4.40	24.90
-3	-1	-16	-18.50	20.60
-3	-1	-16	-10.30	16.80
-3	-1	-16	-22.60	11.50
-3	1	-16	2.80	14.20
-3	1	-16	-16.20	25.80
3	1	16	-12.20	9.40
-3	1	-16	10.90	13.40
-3	1	-16	-35.00	16.50
-4	-1	-16	-17.30	14.80
-4	-1	-16	10.50	13.50
-4	-1	-16	5.00	27.30
-4	1	-16	9.00	15.70
-4	1	-16	-4.80	13.40
-4	1	-16	23.70	17.90
4	1	16	8.90	9.40
-4	1	-16	-27.30	24.30

5	-2	-16	0.00	15.40
-5	-2	16	-8.20	12.60
5	-2	-16	7.00	18.10
-5	-2	16	12.50	28.60
-5	2	16	0.00	11.80
-5	2	16	-11.90	25.40
5	2	-16	15.70	15.20
-5	2	16	37.60	23.60
5	2	-16	8.50	17.20
-4	-2	16	-8.50	10.60
4	-2	-16	-3.20	20.90
4	-2	-16	-9.50	11.80
-4	-2	16	11.40	27.70
-4	2	16	17.20	12.20
-4	2	16	-30.20	21.70
4	2	-16	3.30	12.20
-4	2	16	-26.30	21.80
3	-2	-16	-10.80	11.20
-3	-2	16	1.80	10.00
3	-2	-16	-1.00	14.70
-3	-2	16	1.00	23.60
-3	2	16	24.30	27.20
3	2	-16	-4.40	13.30
-3	2	16	13.10	12.30
-3	2	16	49.20	20.30
3	2	-16	29.70	17.20
2	-2	-16	21.30	12.60
-2	-2	16	2.20	8.50
-2	-2	16	1.80	23.60
2	-2	-16	1.00	15.80
-2	2	16	5.60	25.00

2	2	-16	-2.80	12.10
-2	2	16	15.70	18.40
-2	2	16	-13.40	9.10
2	2	-16	21.50	16.70
-1	-2	16	0.40	23.60
1	-2	-16	19.50	16.80
-1	-2	16	-7.70	8.30
1	-2	-16	-1.30	11.00
1	2	-16	-21.40	13.40
-1	2	16	-5.90	23.90
1	2	-16	5.70	12.20
-1	2	16	0.90	10.60
0	-2	-16	3.60	18.80
0	-2	16	-27.90	21.90
0	-2	-16	-2.60	10.80
0	-2	16	10.70	8.10
0	-2	-16	-6.60	14.50
0	2	-16	-14.40	10.40
0	2	16	-16.30	15.00
0	2	16	-11.80	22.90
0	2	-16	34.50	22.80
0	2	-16	-4.40	14.60
0	2	16	-3.00	10.00
-1	-2	-16	23.10	16.10
1	-2	16	0.50	7.30
-1	-2	-16	-16.80	22.00
-1	-2	-16	1.60	12.40
1	-2	16	55.59	25.60
-1	-2	-16	-25.10	20.00
1	2	16	14.40	17.00
-1	2	-16	6.20	13.20

1	2	16	3.60	11.20
-1	2	-16	-24.30	22.00
-1	2	-16	-12.90	15.30
-1	2	-16	15.50	16.80
-2	-2	-16	-23.60	20.80
-2	-2	-16	7.70	26.90
-2	-2	-16	-17.00	10.20
-2	-2	-16	-4.40	14.00
-2	2	-16	-9.90	11.80
-2	2	-16	27.50	27.50
2	2	16	6.70	19.90
-2	2	-16	-2.70	16.40
-2	2	-16	-2.40	25.20
2	2	16	6.20	9.70
-3	-2	-16	28.60	16.50
-3	-2	-16	-5.40	21.40
-3	-2	-16	-7.40	12.20
-3	-2	-16	34.20	24.90
3	2	16	16.50	19.60
-3	2	-16	-12.70	15.20
-3	2	-16	-3.60	15.90
-3	2	-16	-20.30	11.80
3	2	16	-9.10	9.60
-4	-2	-16	35.80	16.40
-4	-2	-16	-4.30	43.60
-4	-2	-16	-13.30	21.10
-4	-2	-16	-26.30	13.80
-4	2	-16	12.10	14.50
-4	2	-16	11.20	25.20
4	2	16	22.70	11.00
-4	2	-16	0.80	17.30

-4	2	-16	-13.90	15.00
4	-3	-16	-9.20	17.00
-4	-3	16	18.40	11.30
4	-3	-16	-10.50	13.00
4	3	-16	19.10	14.70
-4	3	16	-11.40	11.40
-4	3	16	-34.60	22.10
-4	3	16	-1.50	21.00
4	3	-16	-1.60	17.10
3	-3	-16	38.80	20.80
-3	-3	16	23.30	26.30
-3	-3	16	2.40	9.70
3	-3	-16	-2.30	20.30
3	3	-16	9.70	12.50
-3	3	16	-40.00	23.30
3	3	-16	-21.70	15.60
-3	3	16	1.00	10.30
-3	3	16	19.60	20.40
2	-3	-16	13.80	16.70
-2	-3	16	-2.80	8.30
2	-3	-16	-5.70	12.10
-2	-3	16	-10.10	21.80
-2	3	16	-21.10	21.20
-2	3	16	-28.60	17.30
-2	3	16	1.00	10.20
2	3	-16	1.30	16.60
2	3	-16	13.30	12.60
-1	-3	16	7.70	25.30
1	-3	-16	21.90	13.30
-1	-3	16	9.40	8.30
1	-3	-16	54.29	27.00

1	-3	-16	30.80	17.50
1	3	-16	28.40	15.00
-1	3	16	19.70	24.40
-1	3	16	37.50	18.80
1	3	-16	31.60	13.20
-1	3	16	16.10	11.30
0	-3	-16	-12.20	23.20
0	-3	16	-39.50	25.40
0	-3	-16	12.30	14.90
0	-3	16	-5.50	11.20
0	-3	-16	6.20	11.90
0	3	16	-10.20	17.60
0	3	-16	-9.90	14.30
0	3	-16	0.10	20.20
0	3	-16	-4.30	11.90
0	3	16	15.10	24.40
0	3	16	-6.90	10.70
-1	-3	-16	52.39	25.50
1	-3	16	53.49	27.80
-1	-3	-16	21.40	14.30
-1	-3	-16	21.00	22.40
-1	-3	-16	43.40	18.30
1	3	16	13.70	10.70
1	3	16	31.40	22.40
-1	3	-16	8.50	22.20
-1	3	-16	17.90	16.40
-1	3	-16	32.60	16.50
1	3	16	46.40	20.80
-1	3	-16	45.70	14.60
-2	-3	-16	-6.00	12.00
-2	-3	-16	-26.30	21.20

-2	-3	-16	12.90	16.10
-2	-3	-16	2.20	27.40
-2	3	-16	0.90	20.10
-2	3	-16	8.20	12.50
2	3	16	6.60	11.80
2	3	16	-21.20	17.80
-2	3	-16	-3.00	14.60
-3	-3	-16	0.00	13.30
-3	-3	-16	-26.30	14.50
-3	-3	-16	-25.30	25.00
-3	-3	-16	-18.80	24.90
-3	3	-16	-8.20	16.10
-3	3	-16	30.20	17.10
-3	3	-16	11.90	13.70
3	3	16	49.40	19.60
-3	3	-16	-20.20	21.60
3	3	16	-23.90	9.60
-4	-3	-16	-4.90	13.00
-4	-3	-16	3.60	24.60
-4	-3	-16	-17.40	15.50
4	3	16	12.90	19.90
-4	3	-16	38.10	16.90
-4	3	-16	-14.20	12.90
4	3	16	2.00	11.90
-4	3	-16	24.40	15.40
-4	3	-16	-9.70	23.10
-4	-4	16	-5.80	31.90
-4	-4	16	6.20	12.20
4	-4	-16	13.80	13.70
4	-4	-16	-5.80	18.00
-4	4	16	-2.00	12.30

-4	4	16	9.80	23.70
-4	4	16	6.20	26.50
4	4	-16	-15.90	14.00
3	-4	-16	12.80	14.30
-3	-4	16	2.50	10.20
-3	-4	16	-17.30	29.10
-3	4	16	19.40	22.20
-3	4	16	1.10	12.80
-3	4	16	32.10	23.60
3	4	-16	38.90	15.60
2	-4	-16	-11.50	14.40
-2	-4	16	-21.60	26.70
-2	-4	16	-6.40	9.50
2	-4	-16	0.20	12.50
-2	4	16	-11.30	23.80
-2	4	16	-4.00	11.20
-2	4	16	33.40	20.90
2	4	-16	13.50	12.00
-1	-4	16	-28.80	25.20
1	-4	-16	27.80	14.70
1	-4	-16	18.90	22.40
1	-4	-16	-16.40	11.20
-1	4	16	4.20	24.80
-1	4	16	-21.60	20.00
1	4	-16	-7.50	12.40
-1	4	16	22.40	11.70
1	4	-16	12.10	16.90
0	-4	-16	-12.50	24.90
0	-4	-16	-7.50	15.60
0	-4	-16	13.10	12.00
0	4	16	7.70	24.10

0	4	16	-11.20	11.20
0	4	16	21.90	19.20
0	4	-16	24.00	22.80
0	4	-16	8.40	12.90
0	4	-16	12.90	15.20
-1	-4	-16	8.90	16.60
-1	-4	-16	23.10	26.40
-1	-4	-16	16.10	24.90
-1	-4	-16	19.90	13.80
-1	4	-16	14.80	16.80
-1	4	-16	-12.40	14.60
-1	4	-16	-9.70	22.90
1	4	16	-4.00	11.30
1	4	16	-5.10	18.70
1	4	16	21.50	24.00
-1	4	-16	-4.10	12.00
-2	-4	-16	17.20	16.20
-2	-4	-16	15.80	25.10
-2	-4	-16	-0.60	14.20
-2	4	-16	-10.60	13.90
2	4	16	-3.40	11.60
2	4	16	29.50	19.70
-2	4	-16	0.70	12.40
-2	4	-16	23.40	18.00
-3	-4	-16	-18.90	26.90
-3	-4	-16	34.00	17.20
-3	-4	-16	-15.80	13.40
-3	-4	-16	8.40	23.70
-3	4	-16	-4.20	13.50
3	4	16	-21.40	23.00
3	4	16	18.20	12.70

-3	4	-16	-2.50	25.20
-3	4	-16	46.30	16.70
-3	4	-16	-12.40	17.40
-4	-4	-16	16.30	15.70
-4	-4	-16	-32.50	15.70
-4	-4	-16	-25.30	26.90
-4	4	-16	13.20	14.70
4	4	16	5.80	12.80
-4	4	-16	-23.40	13.50
4	4	16	13.20	23.10
-4	4	-16	-17.60	26.90
-3	-5	16	-16.30	29.00
3	-5	-16	-1.40	13.60
3	-5	-16	-8.70	14.80
3	5	-16	17.70	13.50
-3	5	16	-17.70	23.00
-3	5	16	-20.80	20.70
-3	5	16	-11.20	13.10
2	-5	-16	-1.10	12.90
2	-5	-16	1.40	25.50
-2	5	16	-2.80	22.70
2	5	-16	15.00	13.60
-2	5	16	1.70	13.40
-2	5	16	-5.60	26.20
1	-5	-16	18.30	14.20
1	-5	-16	21.50	15.90
1	-5	-16	-24.90	23.50
-1	5	16	3.60	19.80
-1	5	16	29.20	26.20
1	5	-16	28.80	13.60
-1	5	16	11.90	12.70

0	−5	−16	50.99	26.10
0	−5	−16	−36.20	14.60
0	5	16	23.90	26.50
0	5	−16	−15.30	12.20
0	5	16	−6.10	22.60
0	5	16	−5.90	12.50
−1	−5	−16	15.70	12.90
−1	−5	−16	−25.00	24.70
−1	−5	−16	−8.50	27.30
−1	−5	−16	35.50	15.50
−1	5	−16	38.80	16.50
−1	5	−16	13.20	23.30
−1	5	−16	12.30	12.50
−1	5	−16	63.49	18.10
1	5	16	−26.20	17.00
1	5	16	17.90	13.30
−2	−5	−16	−5.00	13.80
−2	−5	−16	7.80	23.80
−2	−5	−16	−14.10	15.10
−2	−5	−16	−9.40	23.70
2	5	16	5.70	22.00
−2	5	−16	−34.80	15.60
−2	5	−16	18.10	25.10
−2	5	−16	−14.00	12.10
−2	5	−16	15.00	15.10
2	5	16	6.30	12.90
−3	−5	−16	−7.10	26.20
−3	−5	−16	8.90	16.40
−3	−5	−16	1.40	16.30
−3	−5	−16	11.10	28.30
−3	5	−16	24.40	13.60

-3	5	-16	0.70	24.80
3	5	16	-9.70	12.00
-3	5	-16	16.40	18.30
-3	5	-16	-6.90	13.00
-3	6	16	8.80	25.80
2	-6	-16	1.90	16.30
2	-6	-16	17.70	14.60
2	-6	-16	34.00	27.70
-2	6	16	12.20	14.50
-2	6	16	-8.00	28.00
2	6	-16	4.40	13.80
-2	6	16	9.20	25.40
1	-6	-16	6.70	12.30
1	-6	-16	-10.70	26.00
-1	6	16	14.60	19.40
-1	6	16	-38.50	24.40
1	6	-16	4.70	13.00
-1	6	16	-11.80	13.30
0	-6	-16	12.30	15.80
0	-6	-16	13.10	27.30
0	-6	-16	-5.50	13.30
0	-6	-16	16.10	26.50
0	6	-16	12.10	26.70
0	6	16	6.70	25.20
0	6	-16	-3.60	12.60
0	6	-16	-19.50	14.60
0	6	16	0.60	23.90
-1	-6	-16	-25.90	16.30
-1	-6	-16	-5.60	25.20
-1	-6	-16	6.20	14.40
-1	-6	-16	-0.20	25.30

-1	6	-16	-0.10	13.00
1	6	16	29.30	23.90
-1	6	-16	5.30	27.70
-1	6	-16	4.40	13.60
-2	-6	-16	25.30	27.10
-2	-6	-16	9.30	14.90
-2	-6	-16	21.40	17.40
-2	-6	-16	32.10	30.60
-2	6	-16	29.10	16.70
-2	6	-16	-22.60	13.40
2	6	16	12.70	14.70
2	6	16	-35.70	41.60
-2	6	-16	13.30	27.30
-2	6	-16	32.60	13.40
-1	7	16	40.30	27.20
0	-7	-16	-12.60	19.40
0	-7	-16	12.20	27.00
0	-7	-16	-34.90	25.30
0	7	-16	-6.70	24.90
0	7	16	26.20	29.10
0	7	16	3.30	14.00
0	7	16	-6.30	25.40
0	7	-16	-18.60	13.70
3	0	-17	11.50	14.90
-3	0	17	-4.70	26.20
-3	0	17	-8.10	19.90
3	0	-17	41.50	21.80
-3	0	17	4.60	11.40
-2	0	17	27.40	20.70
2	0	-17	-7.70	16.50
-2	0	17	9.10	25.40

-2	0	17	-1.30	10.80
2	0	-17	3.80	13.30
-1	0	17	-41.60	18.00
-1	0	17	-4.40	10.90
-1	0	17	9.90	26.00
1	0	-17	3.70	13.60
1	0	-17	-16.30	16.20
0	0	-17	-19.40	17.00
0	0	17	-1.30	10.80
0	0	17	5.00	19.70
0	0	17	-0.40	27.70
0	0	-17	-35.90	20.40
0	0	-17	-0.10	11.70
-1	0	-17	-18.20	16.80
-1	0	-17	-6.10	12.90
1	0	17	-3.00	10.60
-1	0	-17	-8.70	26.30
1	0	17	8.20	26.10
1	0	17	12.90	19.00
-2	0	-17	26.10	30.90
-2	0	-17	9.30	16.50
2	0	17	9.50	9.70
-2	0	-17	3.60	14.50
-3	0	-17	-3.20	24.20
-3	0	-17	-13.00	21.40
-3	0	-17	-36.80	16.00
-3	0	-17	0.70	15.60
3	0	17	9.60	11.50
3	-1	-17	-28.60	16.80
3	-1	-17	33.80	16.60
-3	-1	17	5.20	29.20

-3	-1	17	-0.20	10.90
3	1	-17	-35.50	16.90
-3	1	17	15.60	21.70
-3	1	17	-19.40	24.00
-3	1	17	-15.30	11.80
2	-1	-17	-18.20	18.30
-2	-1	17	-17.00	29.50
-2	-1	17	-30.40	10.20
-2	-1	17	13.60	27.80
2	1	-17	1.80	12.10
-2	1	17	-30.00	25.80
2	1	-17	41.20	18.70
-2	1	17	-2.20	19.00
-2	1	17	-0.10	11.80
1	-1	-17	53.09	29.50
1	-1	-17	37.80	19.40
-1	-1	17	58.89	21.40
1	-1	-17	10.30	13.70
-1	-1	17	63.89	30.80
-1	1	17	40.20	28.70
1	1	-17	18.90	13.20
-1	1	17	15.00	11.90
1	1	-17	35.70	17.80
0	-1	-17	-12.90	11.10
0	-1	17	-5.60	29.20
0	-1	-17	-10.20	16.40
0	-1	17	-7.20	10.00
0	-1	-17	1.30	26.70
0	1	17	-27.80	9.80
0	1	17	26.70	31.90
0	1	-17	7.50	12.60

0	1	-17	-44.90	15.00
0	1	17	11.10	20.70
1	-1	17	34.30	30.30
1	-1	17	-5.00	10.00
-1	-1	-17	11.80	24.40
-1	-1	-17	-50.39	29.00
-1	-1	-17	24.80	12.90
-1	-1	-17	-6.60	17.00
1	1	17	18.70	21.70
-1	1	-17	5.10	23.10
-1	1	-17	-8.90	18.50
-1	1	-17	36.40	19.60
-1	1	-17	5.50	13.70
1	1	17	22.90	12.20
1	1	17	15.00	26.00
-2	-1	-17	7.00	15.00
-2	-1	-17	34.70	17.70
-2	-1	-17	-57.89	26.10
-2	-1	-17	2.70	23.60
-2	1	-17	-25.40	16.60
-2	1	-17	27.30	29.90
2	1	17	6.20	10.70
-2	1	-17	-3.50	20.30
2	1	17	0.00	19.60
-2	1	-17	-12.40	13.50
-3	-1	-17	19.40	18.10
-3	-1	-17	35.00	32.50
-3	-1	-17	-52.69	22.20
-3	1	-17	-1.50	24.30
-3	1	-17	9.40	31.50
-3	1	-17	19.70	18.10

-3	1	-17	25.10	15.50
3	1	17	8.60	11.50
3	-2	-17	8.30	14.50
-3	-2	17	-15.90	11.40
-3	-2	17	-10.80	26.50
3	-2	-17	-12.40	17.50
-3	2	17	2.00	13.30
-3	2	17	1.70	26.60
-3	2	17	-31.30	20.30
3	2	-17	-16.10	16.00
-2	-2	17	7.30	30.00
-2	-2	17	-9.80	11.50
2	-2	-17	39.20	21.80
2	-2	-17	-5.90	12.50
2	2	-17	8.30	18.30
2	2	-17	-3.20	13.90
-2	2	17	18.10	21.50
-2	2	17	-31.70	23.20
-2	2	17	1.80	12.30
1	-2	-17	10.50	22.10
1	-2	-17	16.80	17.40
-1	-2	17	17.70	30.40
-1	-2	17	-13.40	10.10
-1	2	17	-18.00	19.50
-1	2	17	-12.00	27.30
-1	2	17	-6.90	11.30
1	2	-17	-5.50	12.80
1	2	-17	-29.30	15.60
0	-2	17	-8.40	9.30
0	-2	17	-27.90	28.80
0	-2	-17	12.90	15.90

0	-2	-17	-20.30	16.40
0	-2	-17	-15.80	28.70
0	2	-17	2.90	24.60
0	2	17	1.80	11.40
0	2	17	24.10	21.50
0	2	17	15.70	29.20
0	2	-17	20.40	13.50
0	2	-17	41.90	19.80
-1	-2	-17	23.00	26.60
1	-2	17	9.80	30.10
-1	-2	-17	29.50	17.50
-1	-2	-17	-3.80	13.70
-1	-2	-17	-18.70	24.10
-1	2	-17	-11.30	19.30
1	2	17	10.30	21.10
-1	2	-17	7.00	28.10
-1	2	-17	2.20	17.70
1	2	17	7.90	25.10
1	2	17	0.60	11.30
-1	2	-17	-21.40	13.40
-2	-2	-17	-5.80	24.30
-2	-2	-17	-42.80	16.30
2	2	17	16.70	21.50
-2	2	-17	-0.10	14.40
-2	2	-17	52.99	18.70
-2	2	-17	-11.80	18.10
2	2	17	14.30	12.30
-2	2	-17	-15.00	24.80
-3	-2	-17	6.40	26.40
-3	2	-17	19.40	21.50
3	-3	-17	33.10	20.90

-3	3	17	29.20	24.50
-3	3	17	-3.40	26.70
3	3	-17	22.40	19.40
-2	-3	17	16.70	11.10
-2	-3	17	8.00	31.00
2	-3	-17	10.80	17.70
2	-3	-17	-0.90	12.50
2	3	-17	-6.20	16.30
-2	3	17	15.40	29.60
-2	3	17	5.30	22.00
-2	3	17	-3.30	12.30
2	3	-17	20.40	16.30
-1	-3	17	-50.29	28.10
1	-3	-17	-2.10	14.80
1	-3	-17	47.10	29.00
1	-3	-17	7.80	18.30
-1	3	17	13.70	21.50
1	3	-17	-11.30	12.90
1	3	-17	8.00	18.30
-1	3	17	15.20	13.20
-1	3	17	-3.50	29.80
0	-3	-17	-11.00	12.50
0	-3	-17	-11.70	22.10
0	-3	-17	11.60	17.20
0	3	-17	24.80	27.50
0	3	-17	9.00	14.30
0	3	17	22.00	30.40
0	3	17	25.30	24.20
0	3	-17	-22.50	16.70
0	3	17	6.10	12.30
-1	-3	-17	-29.20	14.00

-1	-3	-17	-12.60	24.70
-1	-3	-17	-1.50	12.30
-1	-3	-17	-1.30	27.30
-1	3	-17	12.70	18.00
-1	3	-17	33.80	26.10
1	3	17	35.30	20.30
1	3	17	83.59	27.90
1	3	17	10.90	13.00
-1	3	-17	-4.00	12.10
-1	3	-17	27.00	17.70
-2	-3	-17	20.70	17.80
-2	-3	-17	11.40	26.40
-2	-3	-17	-8.60	12.70
-2	3	-17	-18.70	13.50
-2	3	-17	-22.90	18.40
2	3	17	20.40	12.90
-2	3	-17	-17.60	26.40
2	3	17	-17.10	21.00
-2	3	-17	-2.90	18.10
2	-4	-17	-7.80	26.60
2	-4	-17	6.70	19.10
2	-4	-17	20.20	14.30
-2	4	17	0.90	13.50
-2	4	17	-6.10	26.40
-2	4	17	-16.60	20.50
2	4	-17	-13.90	13.90
1	-4	-17	-9.90	13.20
1	-4	-17	17.60	26.20
-1	4	17	-27.30	27.10
-1	4	17	2.10	13.10
-1	4	17	28.60	25.60

1	4	-17	-42.10	13.20
1	4	-17	33.00	17.30
0	-4	-17	12.30	18.80
0	-4	-17	40.00	29.50
0	-4	-17	2.90	14.70
0	4	-17	7.50	15.70
0	4	17	28.70	30.20
0	4	17	3.50	13.70
0	4	17	-16.30	22.60
0	4	-17	11.20	27.60
0	4	-17	2.30	19.10
-1	-4	-17	-14.50	16.40
-1	-4	-17	-18.00	25.90
-1	-4	-17	-4.00	27.20
-1	-4	-17	2.40	15.90
-1	4	-17	-8.60	26.50
-1	4	-17	-8.00	16.90
1	4	17	23.50	13.90
1	4	17	34.60	22.20
-1	4	-17	-20.60	14.10
-1	4	-17	7.60	17.80
0	0	0	0.00	0.00

_computing_structure_solution   'SHELXT 2014/5 (Sheldrick, 2014)'

;

_shelx_hkl_checksum   90228
